# Global incidence and mortality trends of gastric cancer and predicted mortality of gastric cancer by 2035

**DOI:** 10.1186/s12889-024-19104-6

**Published:** 2024-07-02

**Authors:** Ju-Li Lin, Jian-Xian Lin, Guang-Tan Lin, Chang-Ming Huang, Chao-Hui Zheng, Jian-Wei Xie, Jia-bin Wang, Jun Lu, Qi-Yue Chen, Ping Li

**Affiliations:** 1 Department of Gastric Surgery, Fujian Medical University Union Hospital, No.29 Xinquan Road, Fuzhou , Fujian Province, 350001 China; 2 Department of General Surgery, Fujian Medical University Union Hospital, Fuzhou, Fujian Province China; 3 Key Laboratory of Ministry of Education of Gastrointestinal Cancer, Fujian Medical University, Fuzhou, Fujian Province, China

**Keywords:** Stomach Neoplasms, Incidence, Mortality, Global trends, Predict mortality

## Abstract

**Objective:**

To study the historical global incidence and mortality trends of gastric cancer and predicted mortality of gastric cancer by 2035.

**Methods:**

Incidence data were retrieved from the Cancer Incidence in Five Continents (CI5) volumes I-XI, and mortality data were obtained from the latest update of the World Health Organization (WHO) mortality database. We used join-point regression analysis to examine historical incidence and mortality trends and used the package NORDPRED in R to predict the number of deaths and mortality rates by 2035 by country and sex.

**Results:**

More than 1,089,000 new cases of gastric cancer and 769,000 related deaths were reported in 2020. The average annual percent change (AAPC) in the incidence of gastric cancer from 2003 to 2012 among the male population, South Korea, Japan, Malta, Canada, Cyprus, and Switzerland showed an increasing trend (*P* > 0.05); among the female population, Canada [AAPC, 1.2; (95%Cl, 0.5–2), *P* < 0.05] showed an increasing trend; and South Korea, Ecuador, Thailand, and Cyprus showed an increasing trend (*P* > 0.05). AAPC in the mortality of gastric cancer from 2006 to 2015 among the male population, Thailand [3.5 (95%cl, 1.6–5.4), *P* < 0.05] showed an increasing trend; Malta Island, New Zealand, Turkey, Switzerland, and Cyprus had an increasing trend (*P* > 0.05); among the male population aged 20–44, Thailand [AAPC, 3.4; (95%cl, 1.3–5.4), *P* < 0.05] showed an increasing trend; Norway, New Zealand, The Netherlands, Slovakia, France, Colombia, Lithuania, and the USA showed an increasing trend (*P* > 0.05). It is predicted that the mortality rate in Slovenia and France’s female population will show an increasing trend by 2035. It is predicted that the absolute number of deaths in the Israeli male population and in Chile, France, and Canada female population will increase by 2035.

**Conclusion:**

In the past decade, the incidence and mortality of gastric cancer have shown a decreasing trend; however, there are still some countries showing an increasing trend, especially among populations younger than 45 years. Although mortality in most countries is predicted to decline by 2035, the absolute number of deaths due to gastric cancer may further increase due to population growth.

**Supplementary Information:**

The online version contains supplementary material available at 10.1186/s12889-024-19104-6.

## Introduction

Gastric cancer is one of the most common cancers worldwide [1]. In 2020, more than 1,089,000 new cases and 769,000 patients died of gastric cancer worldwide. Although the incidence of gastric cancer has been steadily declining over the past few decades [2], the decrease is much less marked in some populations, such as Canada, Brazil, Denmark, India, and Israel [3]. The recognized etiology of gastric cancer includes *Helicobacter pylori* infection (a major confirmed cause of gastric cancer) [4]; obesity [5]; smoking [6]; alcohol [7], high salt food [8]; coffee [9]; gastric ulcer disease [10]; gastroesophageal reflux disease [11, 12]; gastric surgery [13] and radiation exposure [14] are associated with the risk of gastric cancer.

Knowledge of gastric cancer’s global and regional epidemiology is essential for personalized decision-making in risk stratification, screening, and prevention. Owing to the high heterogeneity of epidemiological trends in different regions, using data from high-quality population-based cancer registries to predict future mortality trends is extremely important.

To date, few studies have assessed gastric cancer’s global incidence and mortality trends. A previous study [15] predicted the incidence rate of gastric cancer by 2035 but did not predict the mortality rate by 2035. We hypothesized that gastric cancer incidence and mortality rates have decreased over the past decade. In this study, we used the GLOBOCAN 2020 database, the latest data on cancer incidence in Five Continents Plus (CI5plus) database, and UN World Population Prospects 2019 Revision. We further investigated whether global trends varied by age, sex, and region. The global incidence and mortality trends of gastric cancer will be analyzed, and the mortality rate up to 2035 will be predicted.

## Method

### Data

A total of 41 countries participated in the analysis of incidence and mortality rates. The estimated global incidence and mortality for 2020 were retrieved from the GLOBOCAN database (http://globocan.iarc.fr). Incidence data were retrieved from country-specific registries based on the Cancer Incidence in Five Continents (CI5) volumes I-XI [16]. Mortality data from malignant neoplasms of the stomach (International Classification of Diseases (ICD)-10 C16) were obtained from the latest update of the World Health Organization (WHO) mortality database. We excluded databases with less than 15 calendar years of incidence/mortality, as this does not allow joinpoint regression analysis to be performed. The updated databases of incidence and mortality in the European Cancer Observatory and Nordic cancer registries will replace the data in the CI5 I-XI volume and WHO databases. The incidence and mortality rates for each country have been standardized (using the Segi's standard world population and the revised standard population issued by the WHO for 2020–2025).

In the database, the incidence rates for most countries range from 2003 to 2012 (with some countries' data from 1998 to 2012), and the mortality rates for most countries range from 2006 to 2015 (with some countries' data from 2001 to 2015). Therefore, in order to present a more comprehensive view of the historical trends in incidence rates and mortality rates, the data did not only show the overlapping time period (2006–2012) for both incidence and mortality rates.

### Joinpoint regression analysis to examine incidence and mortality trends

We used joinpoint regression analysis (https://surveillance.cancer.gov/joinpoint/) to examine the historical incidence and mortality trends. We specified the maximum number of three joinpoints as the analysis options. To determine the direction and magnitude of recent trends, the average annual percent change (AAPC) and the corresponding 95% confidence interval (CI) were evaluated for the most recent 10-year period available. The trends of incidence and mortality rates in this study both follow a Poisson distribution. Therefore, a logarithmic linear model analysis was used.

### NORDPRED predicted mortality in 2035

Age-standardized mortality rates per 100 000 person-years were calculated using the world standard population. To predict the number of deaths and mortality rates by 2035 by country and sex, an age–period–cohort model was fitted to recent trends in mortality rates. The model, implemented in R through the NORDPRED package, has been shown to perform well empirically in projecting current trends in cancer mortality in the future [17]. The three or four most recent 5-year observed periods (depending on data availability) were extrapolated using a power function to level off the growth, with a projection of the recent linear trend for the last 10 years that was attenuated (or accentuated in the case of negative trends) by 25% and 50% in the second and third prediction periods, respectively, and by 75% for both the fourth and fifth prediction periods. The number of deaths was predicted up to 2035 by taking a weighted average of the projected mortality rates for the last two prediction periods, centering on 2035, and then applying the rates to the UN World Population Prospects 2019 Revision forecasts available for each country for that year. Predicted ASRs (Age-standardised rate) were then analyzed in light of the mortality threshold of 6 per 100 000 person-years, below which cancer can have low mortality.

### Ethic

As this was a retrospective study involving the examination of secondary cancer data only, patients were not involved in the design and conduct of this research. This study was approved by the Ethics Committee of Fujian Union Hospital of Fujian Medical University.

### Statistical analysis

Stata.14 was used for data management and plotting observed and modeled trends. Modeling analyses were performed using R 4.10, and the functions available in the Epi package version 1.1.36, R Studio, and the NORDPRED package (http://www.kreftregisteret.no/en/Research/ Projects/ Nordpred/Nordpred-software/).

## Results

### New cases and deaths of gastric cancer in 2020

**Fig. 1:**
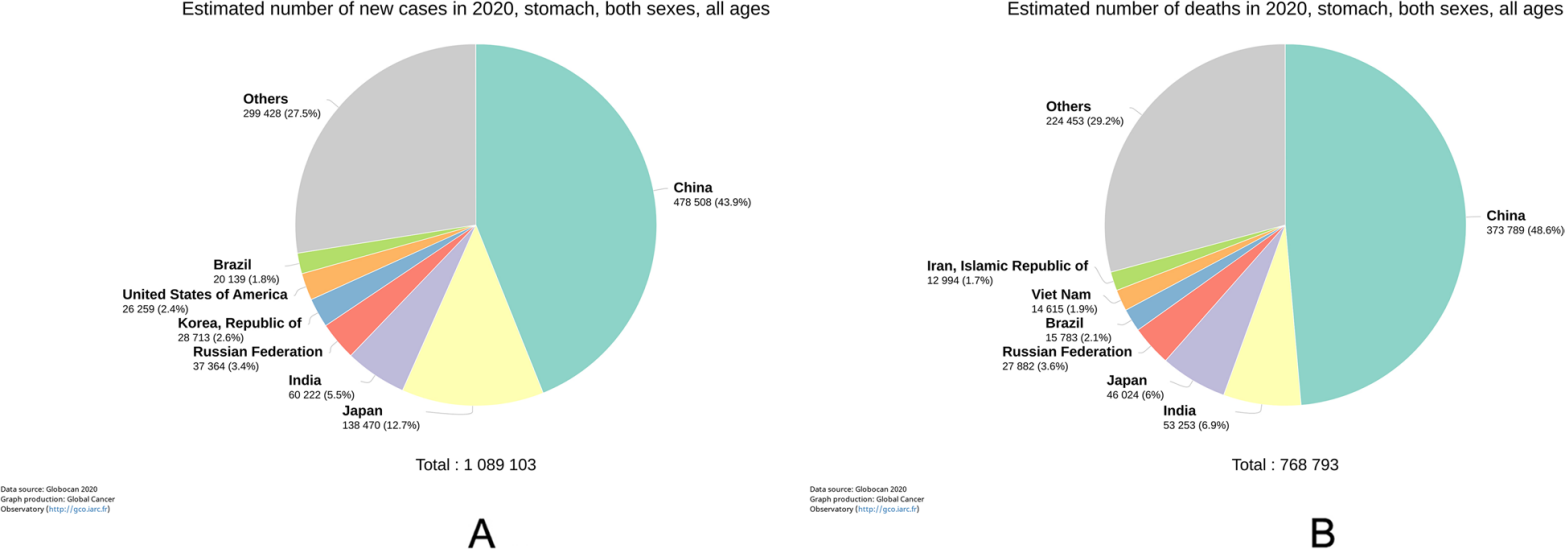
**A** in 2020, there were 1,089,000 new cases of gastric cancer worldwide. **B** in 2020, there were 769,000 deaths of gastric cancer worldwide

In 2020, there were more than 1,089,000 new cases of gastric cancer and 769,000 related deaths. China, Japan, India, Russia, and South Korea were the top five countries for new cases of gastric cancer. The top five countries in terms of deaths were China, India, Japan, Russia, and Brazil. (Fig. 1A, B).

### Global incidence and mortality in 2020

**Fig. 2:**
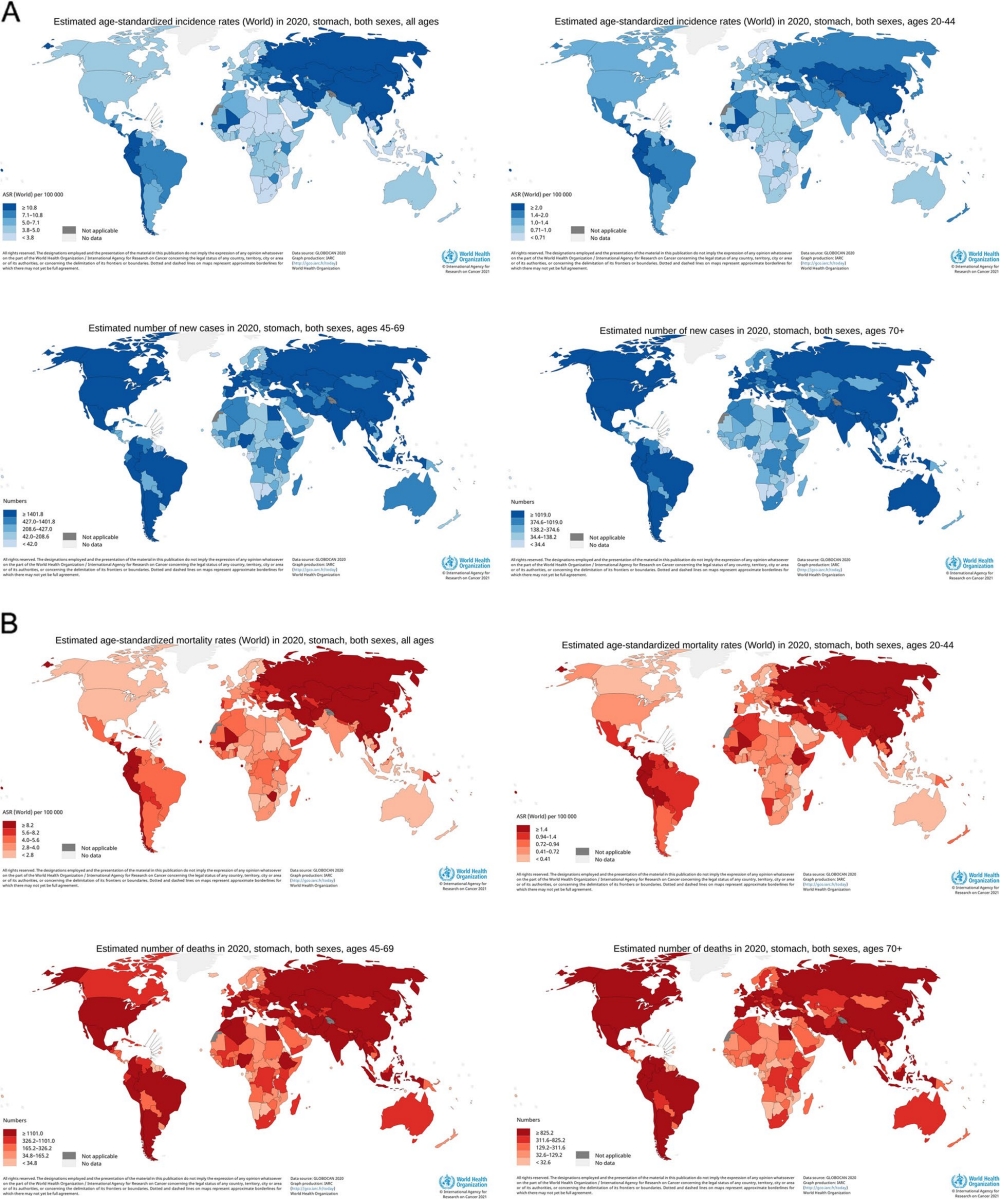
**A** Map of incidence rate of gastric cancer. **B** Map of mortality rate of gastric cancer

The estimated incidence of gastric cancer in Asia was the highest (Fig. 2A). Regarding the total population, Mongolia, Japan, South Korea, Tajikistan and China had the highest incidence rate.

The estimated mortality rate of gastric cancer was the highest in Asia (Fig. 2B). Regarding the total population, Mongolia, Pakistan, Bhutan, China and Kyrgyzstan have the highest mortality rate.

**Fig. 3:**
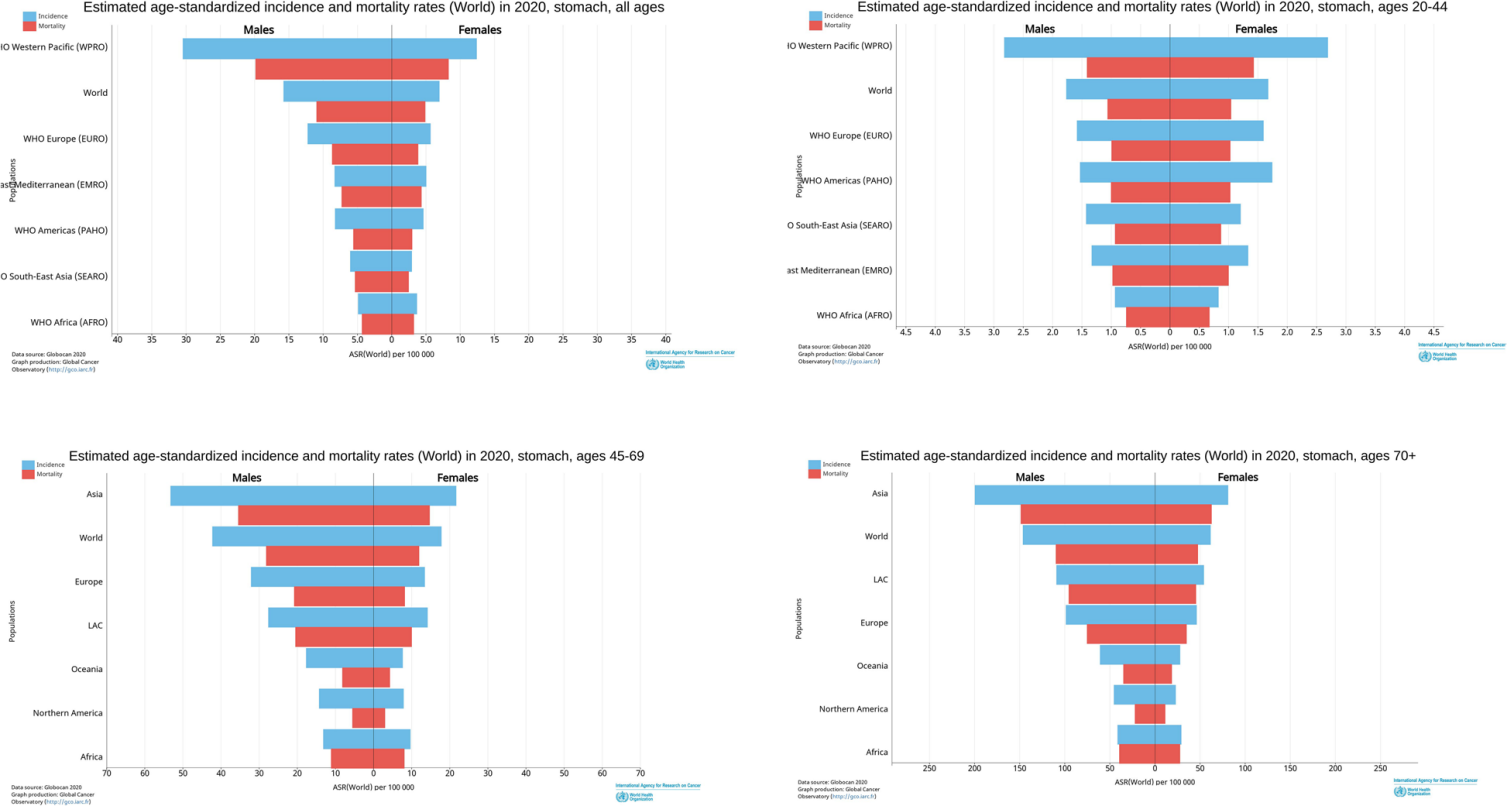
Incidence and mortality of gastric cancer by gender and age

The estimated incidence and mortality rates of gastric cancer also differed between the sexes. In the total population, gastric cancer incidence and mortality rates were higher in men than women. Stratified analysis showed that gastric cancer incidence and mortality rates in men aged 45–74 and 70–85 + were higher than those in women; in the population aged 20–44, the incidence and mortality rates in men were similar to those in women (Fig. 3).

### Average annual percent change in the incidence of gastric cancer from 2003 to 2012

The average annual percent change in incidence in 41 countries from 2003 to 2012 is shown in Table 1.

**Table 1:** AAPC of the Incidence of Gastric Cancer in Individuals 0 to 85 Years or Older

	Males			Females		
Country	APCC	-95% CI	95% CI	APCC	-95% CI	95% CI
South America						
Brazil	-4.6	-9	0.1	-7.4*	-11.8	-2.7
Chile	-2.3	-6	1.6	-0.9	-2.7	1
Colombia	-3.8*	-5.9	-1.7	-4.8*	-6.1	-3.4
Ecuador	-0.8	-4.5	3.1	0.9	-3	4.9
Northern America						
Canada	0.3	-0.2	0.8	1.2*	0.5	2
USA	-1.0*	-1.5	-0.5	-0.4	-0.9	0.2
Eastern Asia						
China	-3.0*	-3.6	-2.4	-3.1*	-3.7	-2.4
Japan	0.2	-0.6	1	-0.2	-1	0.6
Philippines	-8.7*	-11.2	-6.1	-7.4*	-10.1	-4.7
India	-0.3	-3.3	2.8	0	-2.9	3
South Korea	0.2	-0.7	1.1	0.1	-0.7	0.8
Southeastern Asia						
Thailand	0.4	-2.1	3	2.2	-0.6	5.2
Eastern Europe						
Belarus	-1.9*	-2.6	-1.2	-1.8*	-2.5	-1.1
Bulgaria	-2.5*	-3.6	-1.4	-2.2*	-3.6	-0.8
Czech Republic	-3.4*	-4.4	-2.3	-2.1*	-2.9	-1.3
Poland	-4.0*	-6	-1.9	-1.5	-5.7	2.9
Slovakia	-1.9*	-3.1	-0.7	-1.2	-3	0.5
Northern Europe						
Denmark	-1.3	-3.2	0.8	0	-2	2.1
Estonia	-2.4*	-4.6	-0.2	-3.7*	-6.5	-0.9
Iceland	-3.8	-10.3	3.2	-2.6	-9.7	5.1
Ireland	-0.1	-1	0.9	-0.9	-3.2	1.3
Lithuania	-2.1*	-3.6	-0.6	-1.5	-3.2	0.4
Sweden	-1.7	-3.6	0.2	-2.6*	-3.9	-1.2
Norway	-2.5*	-4.3	-0.6	-4.2*	-6.3	-2
Finland	-4.0*	-5.4	-2.5	-3.1*	-4.2	-1.9
UK	-3.4*	-3.8	-2.9	-2.9*	-3.8	-1.9
Western Asia						
Cyprus	0.5	-3.2	4.2	3.7	-2.1	9.7
Southern Europe						
Croatia	-3.6*	-4.7	-2.6	-2.5*	-4	-1
Italy	-4.3*	-5.1	-3.5	-2.7*	-3.9	-1.5
Malta	0.3	-3.6	4.3	-1.3	-8.4	6.4
Slovenia	-2.8*	-3.8	-1.9	-1.4*	-2.8	-0.1
Spain	-2.5*	-3.2	-1.8	-2.0*	-3.3	-0.7
Israel	-2.4*	-4.1	-0.8	-2.6*	-3.9	-1.3
Turkey	-0.5	-1.7	0.7	-1.1	-3.1	1
Western Europe						
Austria	-2.8*	-3.4	-2.3	-3.8*	-4.7	-2.9
France	-2.1*	-2.8	-1.4	-1.8*	-3.5	0
Germany	-1.5*	-2.8	-0.3	-1.3	-3.4	0.9
The Netherlands	-2.6*	-3.3	-1.9	-1.3*	-2.4	-0.2
Switzerland	0.6	-1	2.3	0	-2.9	3
Oceania						
Australia	-1.6*	-2.2	-1	-1.1*	-1.7	-0.4
New Zealand	-2.0*	-3.4	-0.6	-1.8*	-3.4	-0.3

*P < 0.05

**Fig. 4:**
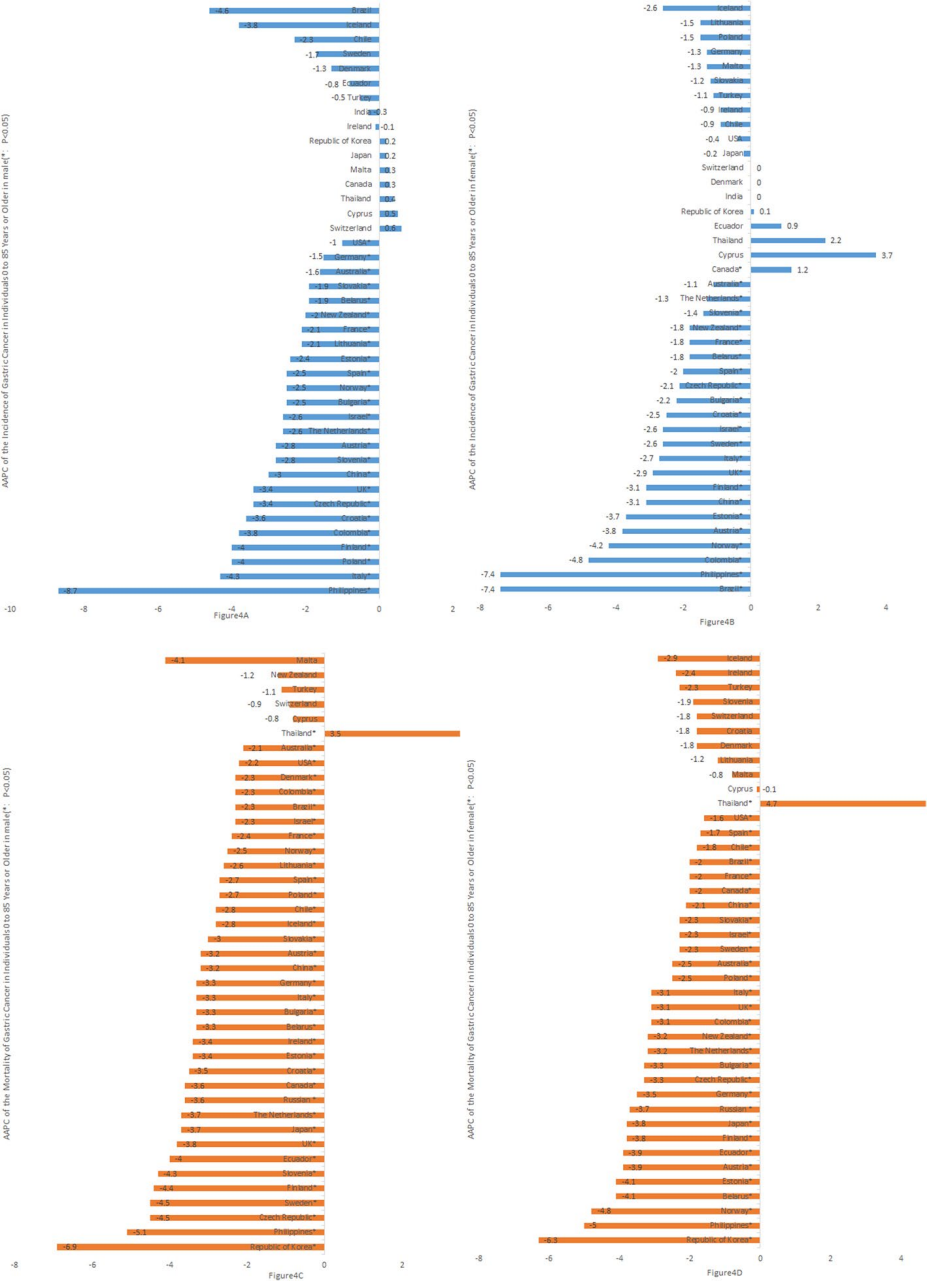
**A** AAPC of the Incidence of Gastric Cancer in Individuals 0 to 85 Years or Older in male (*: *P* < 0.05). **B** AAPC of the Incidence of Gastric Cancer in Individuals 0 to 85 Years or Older in female (*: *P* < 0.05). **C** AAPC of the Mortality of Gastric Cancer in Individuals 0 to 85 Years or Older in male (*: *P* < 0.05). **D** AAPC of the Mortality of Gastric Cancer in Individuals 0 to 85 Years or Older in female (*: *P* < 0.05)

Among the male population, South Korea, Japan, Malta, Canada, Thailand, Cyprus, and Switzerland showed an increasing trend (*P* > 0.05). Brazil, Iceland, Chile, Sweden, Denmark, Ecuador, Turkey, India, and Ireland showed a decreasing trend (*P* > 0.05), while the other 25 countries showed a decreasing trend (*P* < 0.05) (Fig. 4A).

Among the female population, Canada [AAPC, 1.2; (95%Cl, 0.5–2), *P* < 0.05] showed an increasing trend; South Korea, Ecuador, Thailand, and Cyprus showed an increasing trend (*P* > 0.05); Iceland, Lithuania, Poland, Germany, Malta, Turkey, Ireland, Chile, the USA, and Japan showed a decreasing trend (*P* > 0.05), while the other 23 countries showed a decreasing trend (*P* < 0.05) (Fig. 4B).

### Average annual percent change in the mortality of gastric cancer from 2006 to 2015

The average annual percent change in the mortality of the 41 countries from 2006 to 2015 is shown in Table 2.

**Table 2:** AAPC of the mortality of Gastric Cancer in Individuals 0 to 85 Years or Older

Region	Males			Females		
	APCC	-95% CI	95% CI	APCC	-95% CI	95% CI
South America						
Brazil	-2.3*	-2.7	-2	-2.0*	-2.7	-1.3
Chile	-2.8*	-4	-1.7	-1.8*	-2.7	-0.8
Colombia	-2.3*	-3.1	-1.4	-3.1*	-4.2	-1.9
Ecuador	-4.0*	-5.1	-2.9	-3.9*	-4.7	-3.2
Northern America						
Canada	-3.6*	-4.2	-3	-2.0*	-3	-1.1
USA	-2.2*	-2.5	-1.9	-1.6*	-2.2	-1.1
Eastern Asia						
China	-3.2*	-4.5	-1.9	-2.1*	-3.7	-0.4
Japan	-3.7*	-3.9	-3.4	-3.8*	-4	-3.5
South Korea	-6.9*	-7.3	-6.6	-6.3*	-6.7	-5.9
Southeastern Asia						
Philippines	-5.1*	-5.7	-4.6	-5.0*	-5.7	-4.2
Thailand	3.5*	1.6	5.4	4.7*	3.6	5.9
Eastern Europe						
Belarus	-3.3*	-3.8	-2.9	-4.1*	-4.7	-3.5
Bulgaria	-3.3*	-4.3	-2.3	-3.3*	-4.5	-2.1
Czech Republic	-4.5*	-5.6	-3.3	-3.3*	-4.2	-2.3
Russian	-3.6*	-4.1	-3.2	-3.7*	-4.2	-3.3
Poland	-2.7*	-3	-2.4	-2.5*	-3.6	-1.5
Slovakia	-3.0*	-4.3	-1.7	-2.3*	-4.1	-0.5
Northern Europe						
Denmark	-2.3*	-3.8	-0.7	-1.8	-4.5	1
Estonia	-3.4*	-5.6	-1.2	-4.1*	-6.5	-1.7
Finland	-4.4*	-6	-2.9	-3.8*	-6	-1.6
Iceland	-2.8*	-5.2	-0.4	-2.9	-10.2	5
Ireland	-3.4*	-5.6	-1.2	-2.4	-5	0.3
Lithuania	-2.6*	-4	-1.2	-1.2	-3.7	1.4
Sweden	-4.5*	-5.6	-3.3	-2.3*	-3.9	-0.7
Norway	-2.5*	-4	-1	-4.8*	-6.3	-3.3
UK	-3.8*	-4.1	-3.5	-3.1*	-3.8	-2.4
Western Asia						
Cyprus	-0.8	-5.6	4.2	-0.1	-7.1	7.5
Israel	-2.3*	-3.5	-1.1	-2.3*	-4.3	-0.4
Turkey	-1.1	-5.5	3.5	-2.3	-6.1	1.7
Southern Europe						
Croatia	-3.5*	-4.6	-2.4	-1.8	-3.6	0
Italy	-3.3*	-3.7	-2.9	-3.1*	-3.7	-2.4
Malta	-4.1	-10.5	2.7	-0.8	-6.2	4.9
Slovenia	-4.3*	-5.9	-2.7	-1.9	-4.4	0.7
Spain	-2.7*	-3.3	-2.2	-1.7*	-2.1	-1.2
Western Europe						
Austria	-3.2*	-4.3	-2.1	-3.9*	-5.4	-2.3
France	-2.4*	-2.9	-1.9	-2.0*	-2.8	-1.3
Germany	-3.3*	-3.5	-3	-3.5*	-3.9	-3.1
The Netherlands	-3.7*	-5	-2.4	-3.2*	-4.4	-2
Switzerland	-0.9	-1.8	0	-1.8	-4.3	0.7
Oceania						
Australia	-2.1*	-3.1	-1	-2.5*	-4	-0.9
New Zealand	-1.2	-2.8	0.4	-3.2*	-5.9	-0.4

*P < 0.05

Among the male population, Thailand [3.5 (95%Cl, 1.6–5.4), *P* < 0.05] had an increasing trend; Malta Island, New Zealand, Turkey, Switzerland, and Cyprus had an increasing trend (*P* > 0.05); 35 countries had a decreasing trend (*P* < 0.05) (Fig. 4C).

Among the female population, Thailand [4.7 (95%Cl, 3.6–5.9], *P* < 0.05) had an increasing trend (Iceland, Ireland, Turkey, Slovenia, Switzerland, Croatia, Denmark, Lithuania, Malta, and Cyprus had an increasing trend (*P* > 0.05); and 30 countries had a decreasing trend (*P* < 0.05) (Fig. 4D).

### Predicting mortality trends and absolute number of deaths by 2035

**Fig. 5:**
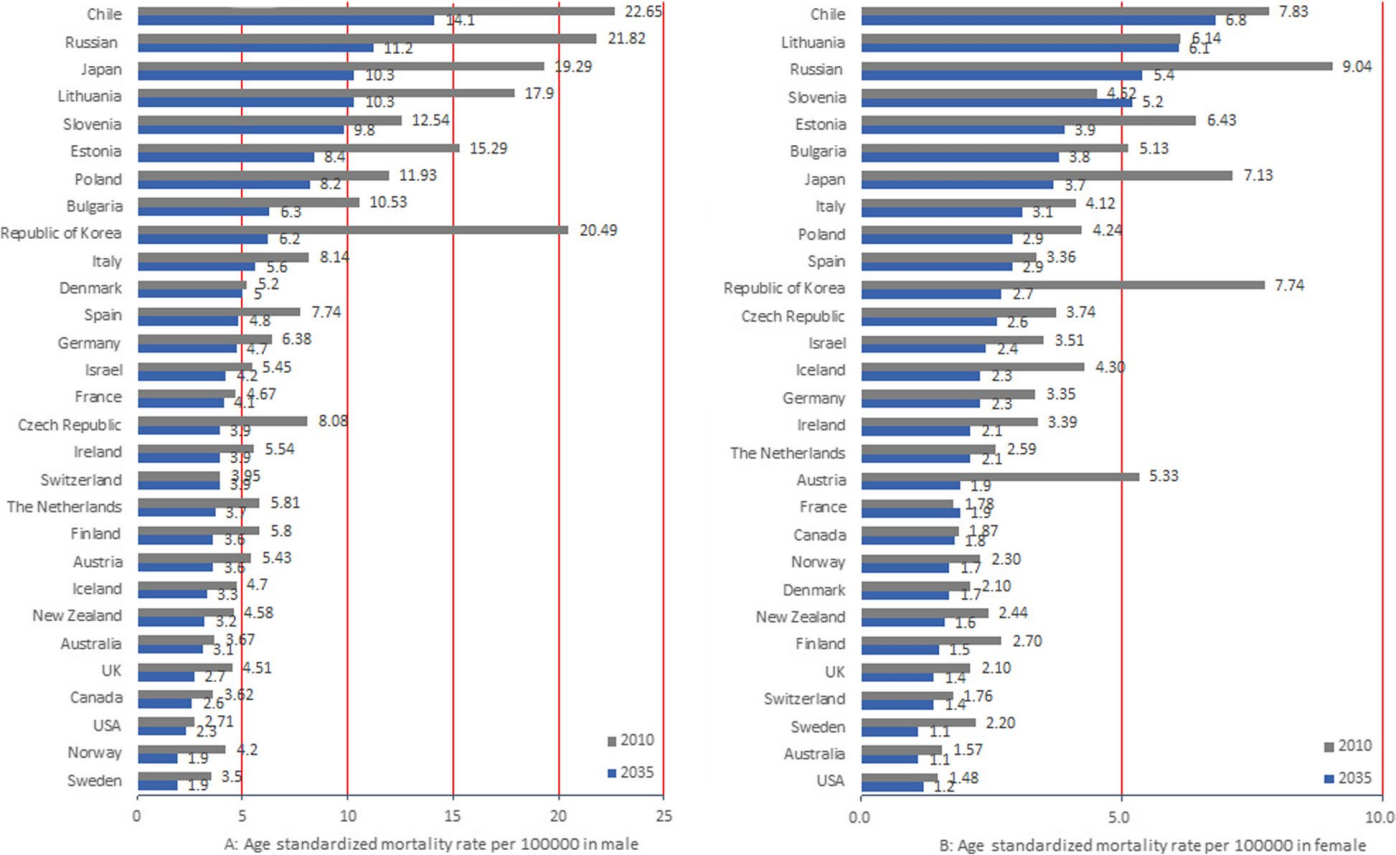
**A** Age standardized mortality rate per 100,000 in male. **B** Age standardized mortality rate per 100,000 in female

Among the male population, it is predicted that the mortality rate in 29 countries will show a decreasing trend by 2035(Fig. 5A). Among the female population, it is predicted that the mortality in 27 countries will show a decreasing trend in 2035, and the mortality in two countries (Slovenia and France) will show an increasing trend until 2035 (Fig. 5B).

Using a mortality threshold of 6 per 100 000, 20 of 29 countries will have reached this threshold by 2035 (Fig. 5A). Using the mortality threshold of 6 per 100 000, 27 out of 29 countries will reach this threshold by 2035 (Fig. 5B). Generally, the mortality rate in male patients is higher than in female patients.

Although mortality rates continue to decrease, the absolute number of deaths is expected to increase further in some countries. Among the male population, it is predicted that the number of deaths in 28 countries will decrease by 2035, but the absolute number of deaths in Israel will increase by 2035 (Table 3). Among the female population, it is predicted that the absolute number of deaths in 26 countries will decrease by 2035, but the absolute number of deaths in Chile, France, and Canada will increase by 2035 (Table 4).

**Table 3:** Number of new gastric cancer cases, age-standardised mortality rates and percentage change in cases due to population and risk in male

	Population (annual, million)		Number of new cases		Age-standardised rate	
	2010	2035	2010	2035	2010	2035
South America						
Chile	8.39	9.61	1900	1183	22.65	14.1
Northern America						
Canada	16.94	21	613	440	3.62	2.6
USA	152.61	177.52	4136	3510	2.71	2.3
Eastern Asia						
Japan	62.87	58.84	12,128	6476	19.29	10.3
South Korea	24.82	25.55	5086	1539	20.49	6.2
Eastern Europe						
Bulgaria	3.62	2.99	381	229	10.53	6.3
Czech Republic	5.17	5.29	418	206	8.08	3.9
Russian	66.54	65.54	14,519	7340	21.82	11.2
Poland	18.53	17.52	2211	1520	11.93	8.2
Northern Europe						
Denmark	2.76	3.02	144	138	5.2	5
Estonia	0.62	0.6	95	52	15.29	8.4
Finland	2.63	2.8	153	101	5.8	3.6
Iceland	0.16	0.18	8	5	4.7	3.3
Ireland	2.26	2.67	125	88	5.54	3.9
Lithuania	1.44	1.1	258	148	17.9	10.3
Sweden	4.68	5.44	164	103	3.5	1.9
Norway	2.44	3.09	102	46	4.2	1.9
UK	31.18	35.54	1406	841	4.51	2.7
Western Asia						
Israel	3.63	5.35	198	225	5.45	4.2
Southern Europe						
Italy	28.7	28.5	2336	1607	8.14	5.6
Slovenia	1.01	1.02	127	99	12.54	9.8
Spain	23.19	22.49	1795	1113	7.74	4.8
Western Europe						
Austria	4.09	4.57	222	147	5.43	3.6
France	30.46	32.49	1422	1248	4.67	4.1
Germany	39.62	41.09	2528	1862	6.38	4.7
The Netherlands	8.28	8.74	482	307	5.81	3.7
Switzerland	3.84	4.67	152	150	3.95	3.9
Oceania						
Australia	11.08	14.61	407	343	3.67	3.1
New Zealand	2.16	2.61	99	70	4.58	3.2

**Table 4:** Number of new gastric cancer cases, age-standardised mortality rates and percentage change in cases due to population and risk in female

	Population (annual, million)		Number of new cases		Age-standardised rate	
	2010	2035	2010	2035	2010	2035
South America						
Chile	8.67	10.05	679	683	7.83	6.8
Northern America						
Canada	17.21	21.24	322	382	1.87	1.8
USA	156.4	181.17	2314	2174	1.48	1.2
Eastern Asia						
Japan	65.67	60.14	4682	2225	7.13	3.7
South Korea	24.72	25.41	1913	686	7.74	2.7
Eastern Europe						
Bulgaria	3.81	3.15	195	120	5.13	3.8
Czech Republic	5.36	5.41	200	141	3.74	2.6
Russian	76.94	75.6	6955	4082	9.04	5.4
Poland	19.8	18.65	840	541	4.24	2.9
Northern Europe						
Denmark	2.81	3.08	59	52	2.1	1.7
Estonia	0.71	0.64	46	25	6.43	3.9
Finland	2.73	2.81	74	42	2.7	1.5
Iceland	0.16	0.18	7	4	4.3	2.3
Ireland	2.31	2.72	78	57	3.39	2.1
Lithuania	1.69	1.26	104	77	6.14	6.1
Sweden	4.71	5.39	104	59	2.2	1.1
Norway	2.44	3.02	56	51	2.3	1.7
UK	32.28	36.18	678	507	2.1	1.4
Western Asia						
Israel	3.72	5.3	131	127	3.51	2.4
Southern Europe						
Italy	30.62	29.63	1262	918	4.12	3.1
Slovenia	1.03	1.02	47	53	4.52	5.2
Spain	23.73	23.29	797	675	3.36	2.9
Western Europe						
Austria	4.31	4.64	230	88	5.33	1.9
France	32.42	34.74	577	660	1.78	1.9
Germany	41.21	41.55	1380	956	3.35	2.3
The Netherlands	8.4	8.76	218	184	2.59	2.1
Switzerland	3.96	4.72	70	66	1.76	1.4
Oceania						
Australia	11.08	14.8	174	163	1.57	1.1
New Zealand	2.24	2.7	55	43	2.44	1.6

## Discussion

This study aimed to further investigate the global incidence and mortality trend of gastric cancer and predict the mortality by 2035 using global data. Several key findings were obtained in this cohort study. First, East Asia has the highest incidence and mortality rates of gastric cancer. Second, in the past decade, gastric cancer incidence and mortality in major countries have decreased; however, the decreasing trend in populations younger than 45 years is not obvious. Third, mortality in Thailand has been increasing over the past decade. Finally, the absolute number of deaths due to gastric cancer in some countries is predicted to increase by 2035.

This study found that the incidence rate in East Asia was the highest, whereas that in Western Europe and Northern America was lower. This finding is consistent with those of previous studies [3, 18]. Different risk factors related to gastric cancer result in this phenomenon. Gastric cancer is divided into non-cardiac cancer (NCGC) and cardiac cancer (CGC). Cardiac cancer is related to obesity and reflux esophagitis in the Western population, whereas most non-cardiac cancers are related to *Helicobacter pylori* infection. The World Health Organization classifies H pylori as a class I carcinogen, which is the most important risk factor for gastric cancer [19]. Nearly 90% of distal gastric cancers can be attributed to H pylori infection [20]. Countries with a high incidence of gastric cancer are related to high H pylori seroprevalence rates, such as South Korea [21], China [22] and Japan [23]. The incidence rate of gastric cancer is relatively low in some countries, such as South Asia and Africa, whereas the seroprevalence of H pylori is very high [3]. Better food preservation practices and refrigeration during the transportation and storage of food have decreased the risk of developing gastric cancer [24]. Other risk factors for gastric cancer include smoking, low intake of fruits and vegetables, and a high intake of salt-preserved foods and possibly alcohol [25, 26].

In contrast to the overall declining incidence, an increasing incidence has been observed in populations younger than 50 years in the USA [27, 28] and the UK [29]. The historical incidence trends in these countries are mainly affected by cardiac cancer. The increase in cardiac cancer is related to an increase in the obesity level. The USA and UK are high-income countries with the highest obesity rates in adults, children, and adolescents, which is a public health concern [30]. This study found that, contrary to the declining trend of the overall incidence rate, the incidence rate of people younger than 45 years in some countries showed an increasing trend, especially in countries with a low incidence rate, including the UK, USA, and the Netherlands.

A recent global study showed that the mortality rate decreased faster than the incidence rate owing to improved socioeconomic status and better access to diagnostics and treatment [31]. Early gastric cancer rarely causes symptoms, and early screening still faces many challenges. Although Japan and South Korea are two high-risk countries, their survival rates are relatively high (> 60%) owing to their great efforts in early screening. Although the implementation of population-based screening in high-risk areas has shown some benefits, whether it will translate into a real reduction in mortality need to be further confirmed [32, 33]. We observed an increasing trend in populations younger than 45 years in some countries, including the USA, the UK, and other developed countries. Moreover, the mortality rate in Thailand has increased significantly in the past decade, which may be related to the low level of economic development and screening rate for early gastric cancer. Therefore, more attention should be paid to the population with gastric cancer in Thailand and those younger than 45 years of age.

A previous study [15] has used global high-quality data to predict whether gastric cancer may become a "rare" disease. This rare cancer threshold was defined in previous studies as cervical cancer (set at less than six cases per 100,000 population) [34]. To our knowledge, this study is also the first attempt to characterize future trends in gastric cancer mortality from a global perspective. Based on the mortality threshold of 6 cases per 100,000 population, those above this threshold can be regarded as high-mortality cancer. Although it is predicted that mortality will continue to decrease in most countries until 2035, gastric cancer is still a high mortality cancer in some countries. The absolute number of deaths is expected to further increase by 2035 as a result of population growth and aging, making gastric cancer a major public health challenge in some world regions.

The prevention of gastric cancer mainly focused on primary prevention and secondary prevention. Primary prevention strategies that can reduce the risk of gastric cancer include dietary modification such as decreasing the intake of salty foods, increasing the intake of fruits and vegetables, avoiding smoking, high alcohol consumption [1, 35–38], and refrigeration or chemical preservation of foods [36]. *Identifying Helicobacter pylori*-infected individuals at high risk for gastric cancer presents an opportunity for primary prevention. A meta-analysis of twenty-four studies [39] demonstrated that *Helicobacter pylori eradication* reduces gastric cancer incidence by 47%. A randomized controlled trial in South Korea [40] demonstrated a 55% reduction in incidence of gastric cancer after *Helicobacter pylori* eradication. Secondary prevention mainly involves endoscopic screening. Four studies [41–44] demonstrated endoscopic screeningr educe gastric cancer-specific mortality ranging from 42–67%.Two studies [42, 43] from Japan found endoscopic screening were superior in reducing gastric cancer-specific mortality compared with radiographic screening. The odds ratio (OR) of gastric cancer specific mortality among screened subjects compared to never-screened individuals was 0.53 (95% CI: 0.51–0.56) in a South Korea study [33].

Our study had several limitations. First, it is very important to distinguish non-cardiac cancer (NCGC) and cardiac cancer (CGC) from etiology; however, the existing data do not support a stratified analysis of incidence and mortality trends. Second, as the database lacks data on the risk factors associated with gastric cancer, we are unable to provide one table of risk factors that are associated with the emergence of gastric cancer.The influence of *Helicobacter pylori* infection, obesity, smoking, and other risk factors on gastric cancer incidence and mortality were not analyzed. Finally, the model used in this study to predict mortality was based on past data; therefore, there was a certain degree of uncertainty.

In the past decade, gastric cancer incidence and mortality have shown a decreasing trend, but there are still some countries showing an increasing trend, especially among populations younger than 45 years of age. Although mortality in most countries is predicted to decline by 2035, the absolute number of deaths due to gastric cancer may further increase due to population growth, making gastric cancer a major public health challenge in some countries. Changes in the epidemiology of gastric cancer require further analysis for cancer control. This study will also aid in the planning and decision-making related to gastric cancer control strategies.

### Supplementary Information

Supplement Figure 1: AAPC of the Incidence of Gastric Cancer in Individuals aged 20-44 in male (*: *P*<0.05).

Supplement Figure 2: AAPC of the Incidence of Gastric Cancer in Individuals aged 45-69 in male (*:*P*<0.05).

Supplement Figure 3: AAPC of the Incidence of Gastric Cancer in Individuals aged 70-85+ in male(*:*P*<0.05).

Supplement Figure 4: AAPC of the Incidence of Gastric Cancer in Individuals aged 20-44 in female (*: *P*<0.05).

Supplement Figure 5: AAPC of the Incidence of Gastric Cancer in Individuals aged 45-69 in female (*:*P*<0.05)

Supplement Figure 6: AAPC of the Incidence of Gastric Cancer in Individuals aged 70-85+ in female (*:*P*<0.05).

Supplement Figure 7: incidence joinpoint.

Supplement Figure 8: mortality joinpoint.

Supplementary material 9.

Supplementary material 10.

## Data Availability

All data were retrieved from country-specific registries based on the Cancer Incidence in Five Continents (CI5) volumes I-XI (16)(https://gco.iarc.fr/overtime/en) and the World Health Organization (WHO) mortality database (https://platform.who.int/mortality). This public database is open, and the use of data does not require additional consent.

## Abbreviations

WHO: World Health Organization
CI5plus: Five Continents Plus
AAPC: The average annual percent change
WHO: World Health Organization
