# Supplementary figures and images for: Global incidence and mortality trends of gastric cancer and predicted mortality of gastric cancer by 2035 (part 1 of 4)
Source: BMC Public Health. 2024 Jul 2;24:1763. doi: 10.1186/s12889-024-19104-6 (PMC11221210; doi:10.1186/s12889-024-19104-6)

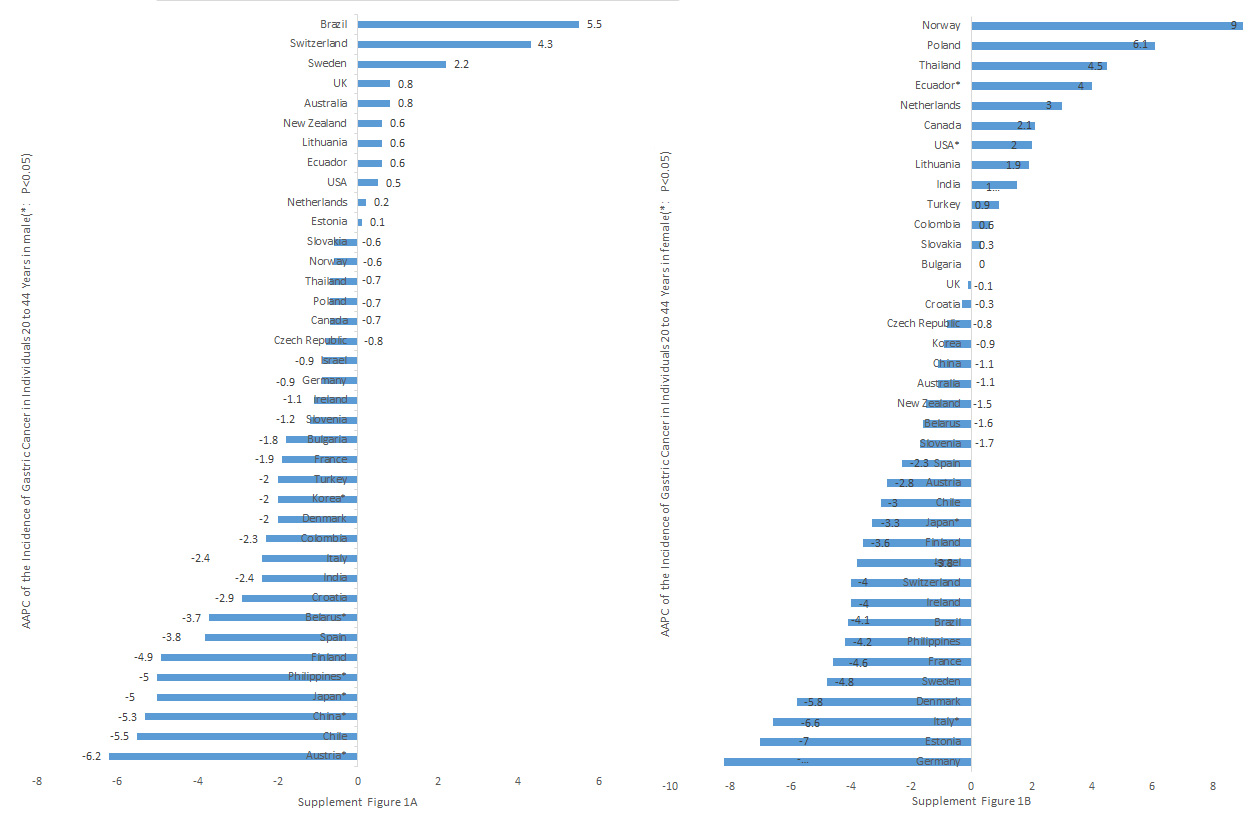

Supplement: Supplementary file 1 — Supplement Figure 1: AAPC of the Incidence of Gastric Cancer in Individuals aged 20-44 in male (*: P<0.05). [file 12889_2024_19104_MOESM1_ESM.jpg]

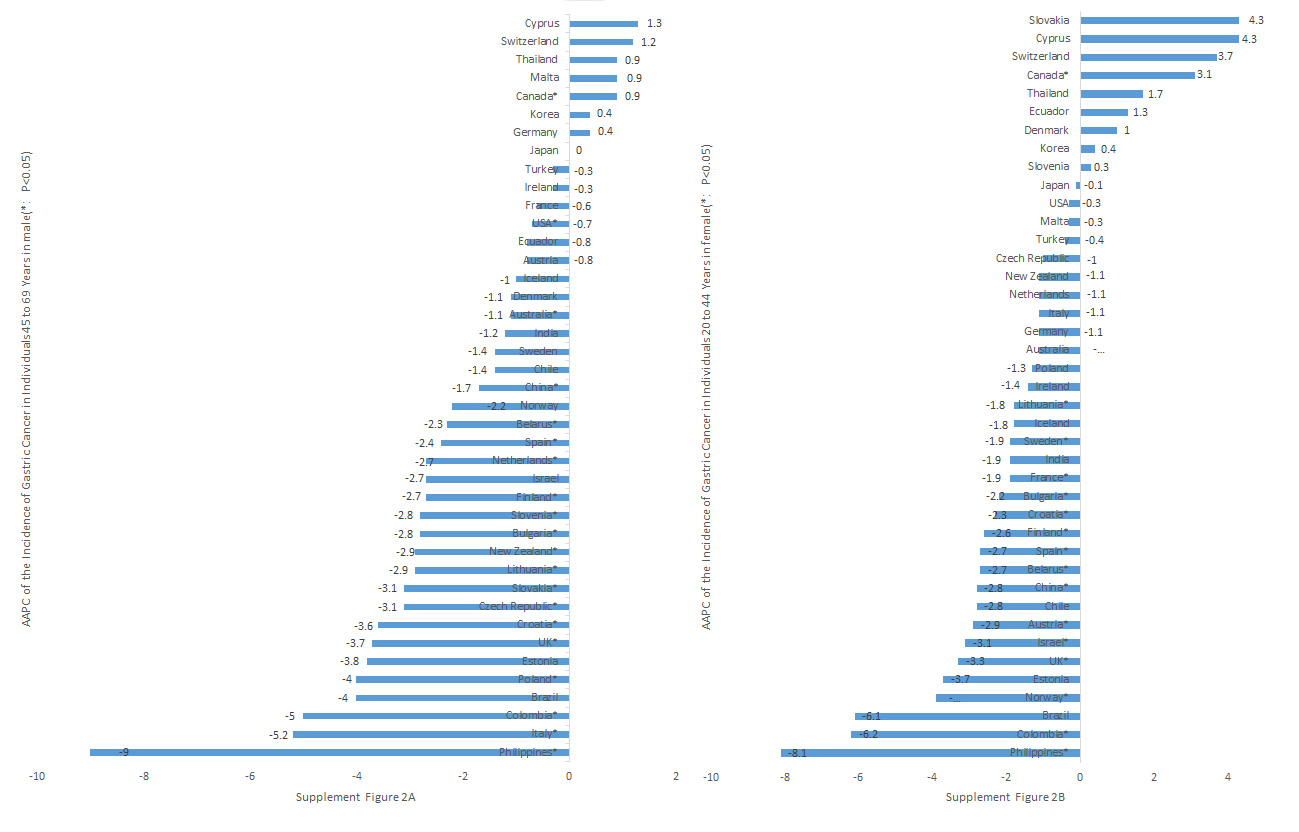

Supplement: Supplementary file 2 — Supplement Figure 2: AAPC of the Incidence of Gastric Cancer in Individuals aged 45-69 in male (*:P<0.05). [file 12889_2024_19104_MOESM2_ESM.jpg]

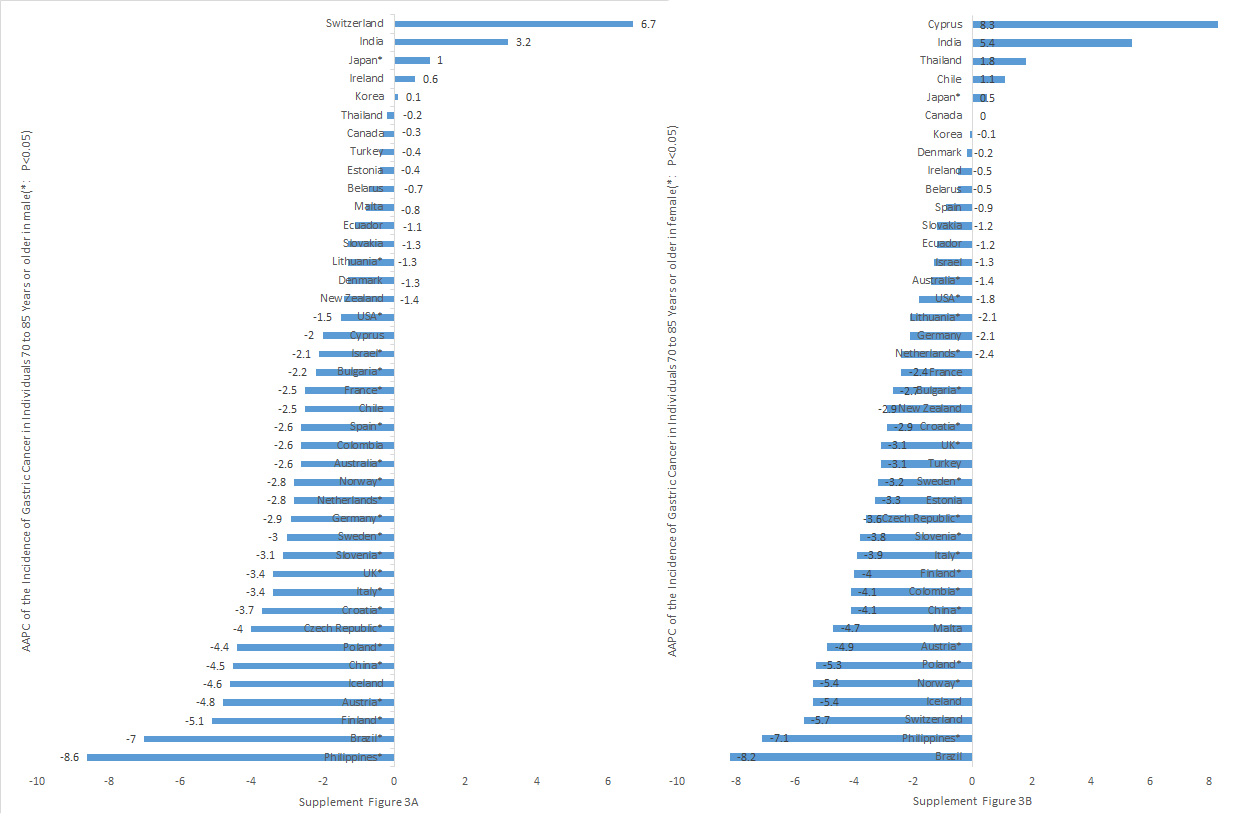

Supplement: Supplementary file 3 — Supplement Figure 3: AAPC of the Incidence of Gastric Cancer in Individuals aged 70-85+ in male(*:P<0.05). [file 12889_2024_19104_MOESM3_ESM.jpg]

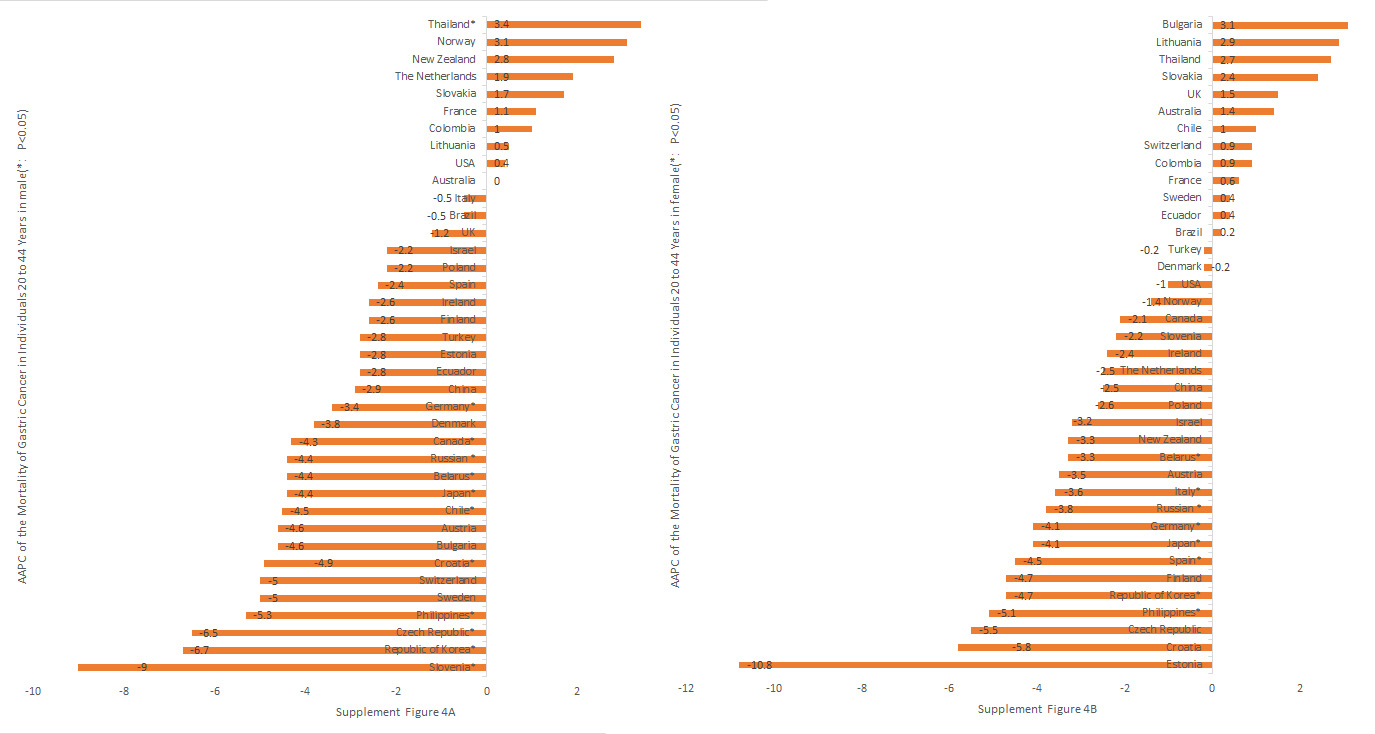

Supplement: Supplementary file 4 — Supplement Figure 4: AAPC of the Incidence of Gastric Cancer in Individuals aged 20-44 in female (*: P<0.05). [file 12889_2024_19104_MOESM4_ESM.jpg]

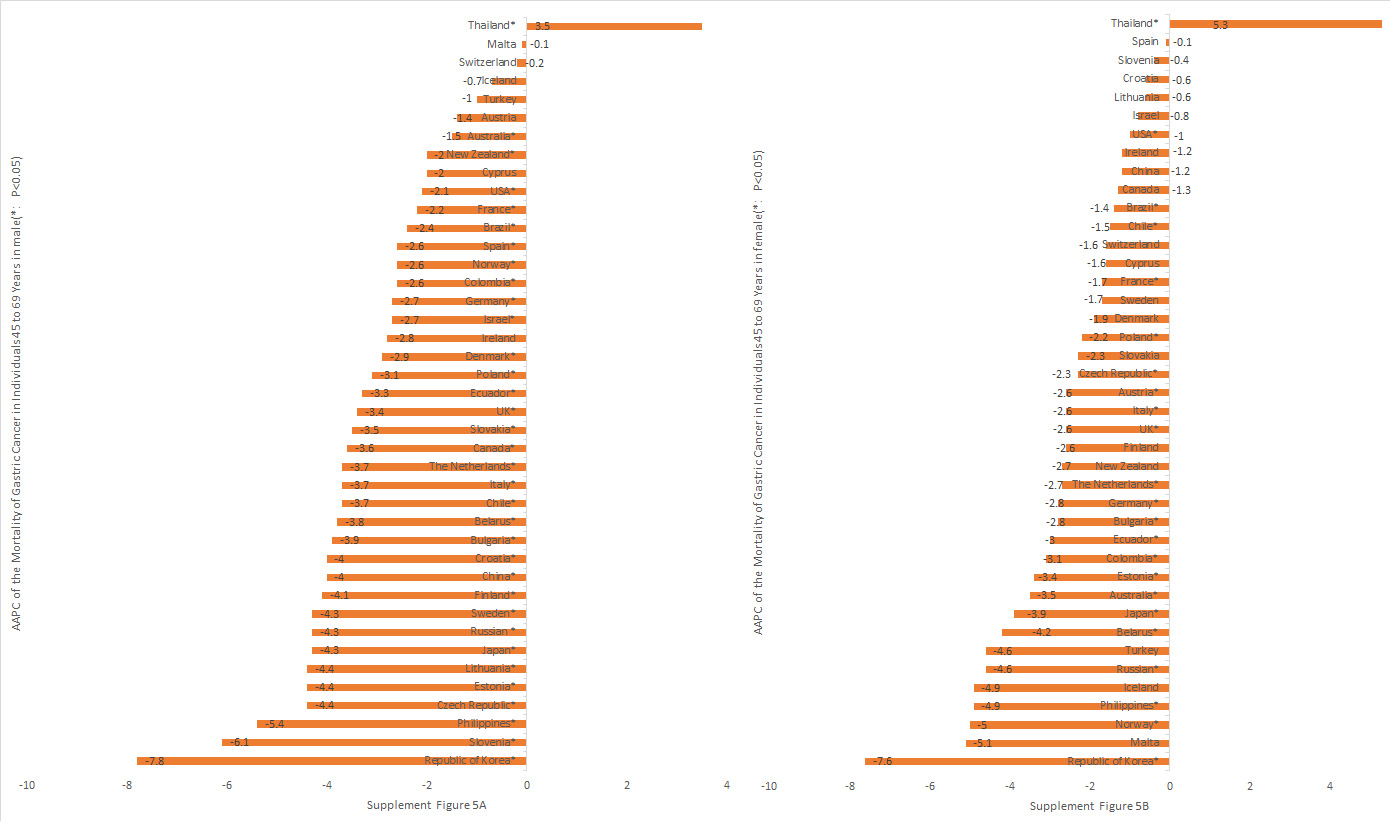

Supplement: Supplementary file 5 — Supplement Figure 5: AAPC of the Incidence of Gastric Cancer in Individuals aged 45-69 in female (*:P<0.05) [file 12889_2024_19104_MOESM5_ESM.jpg]

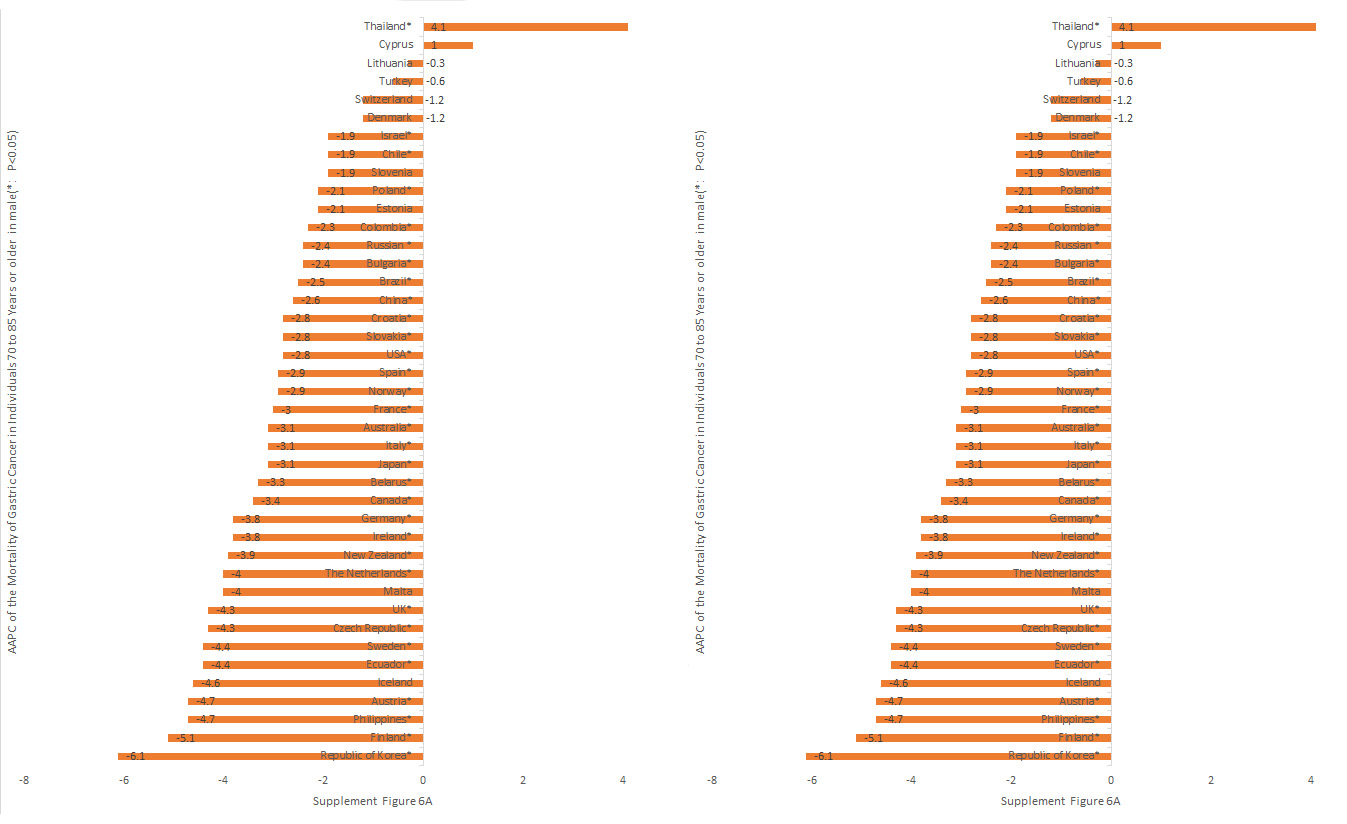

Supplement: Supplementary file 6 — Supplement Figure 6: AAPC of the Incidence of Gastric Cancer in Individuals aged 70-85+ in female (*:P<0.05). [file 12889_2024_19104_MOESM6_ESM.jpg]

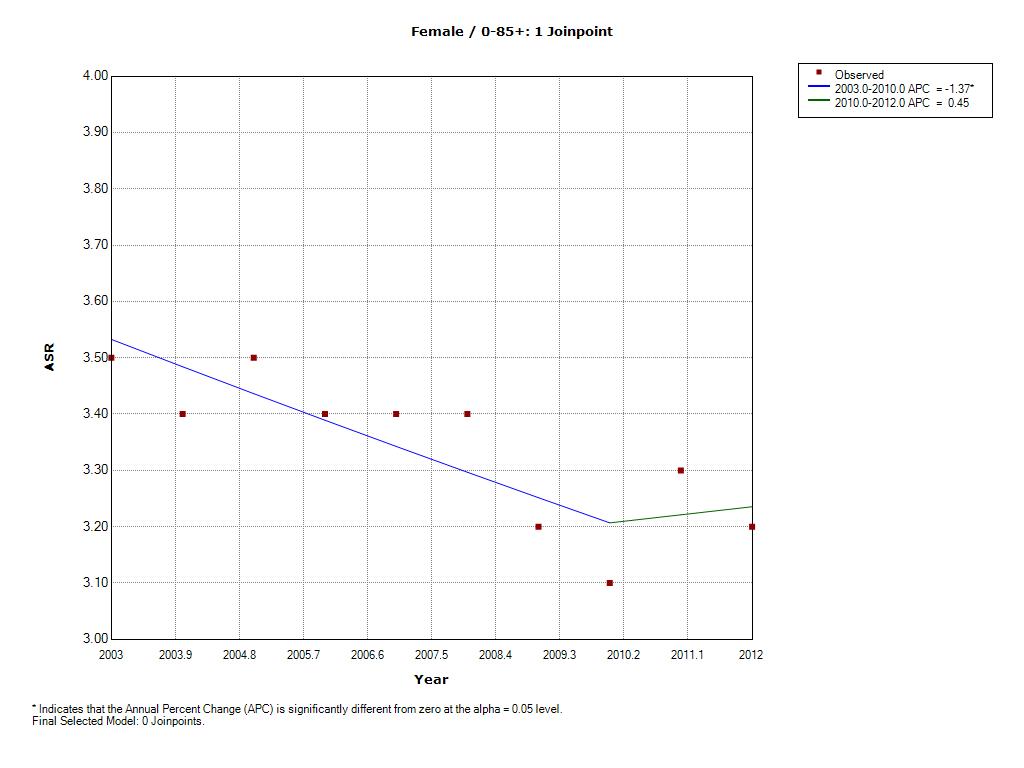

Supplement: Supplementary file 7 — Supplement Figure 7: incidence joinpoint. [file 12889_2024_19104_MOESM7_ESM.zip › Supplement Figure 7 incidence joinpoint/Australia female 0-85+.jpg]

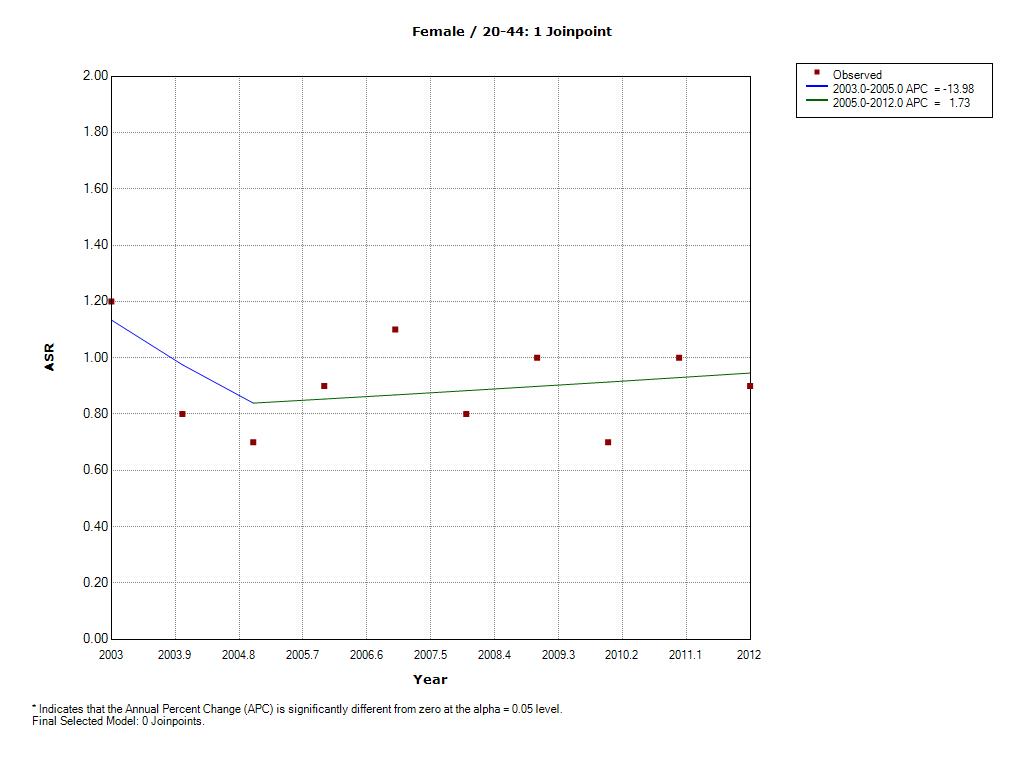

Supplement: Supplementary file 7 — Supplement Figure 7: incidence joinpoint. [file 12889_2024_19104_MOESM7_ESM.zip › Supplement Figure 7 incidence joinpoint/Australia female 20-44.jpg]

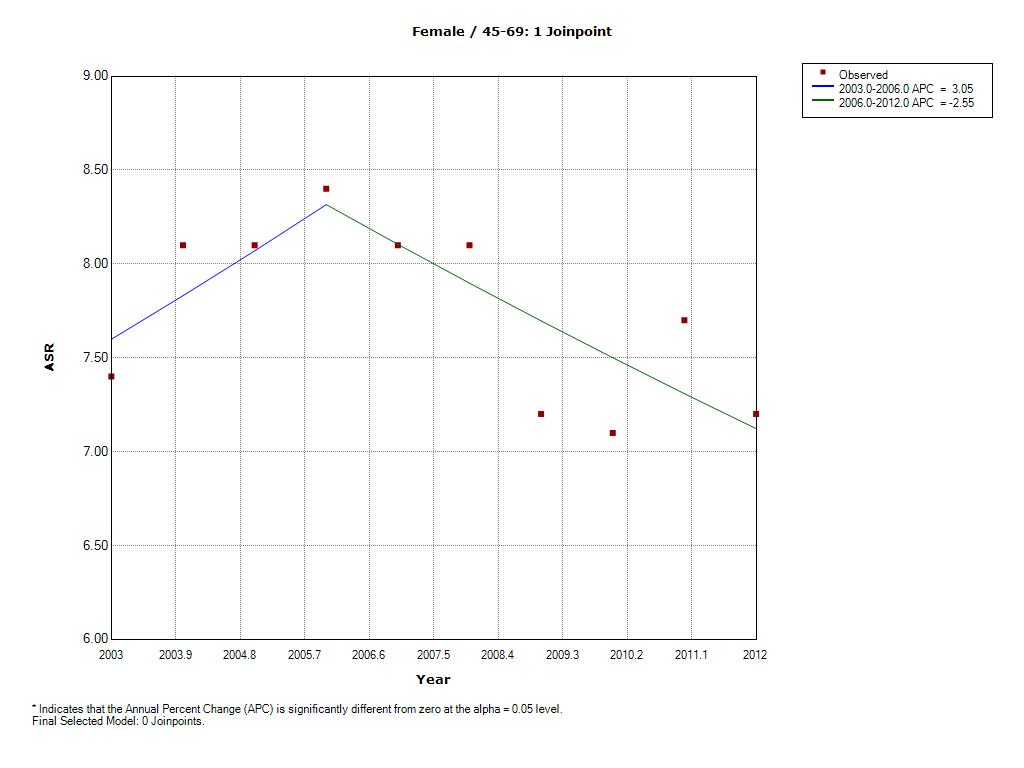

Supplement: Supplementary file 7 — Supplement Figure 7: incidence joinpoint. [file 12889_2024_19104_MOESM7_ESM.zip › Supplement Figure 7 incidence joinpoint/Australia female 45-69.jpg]

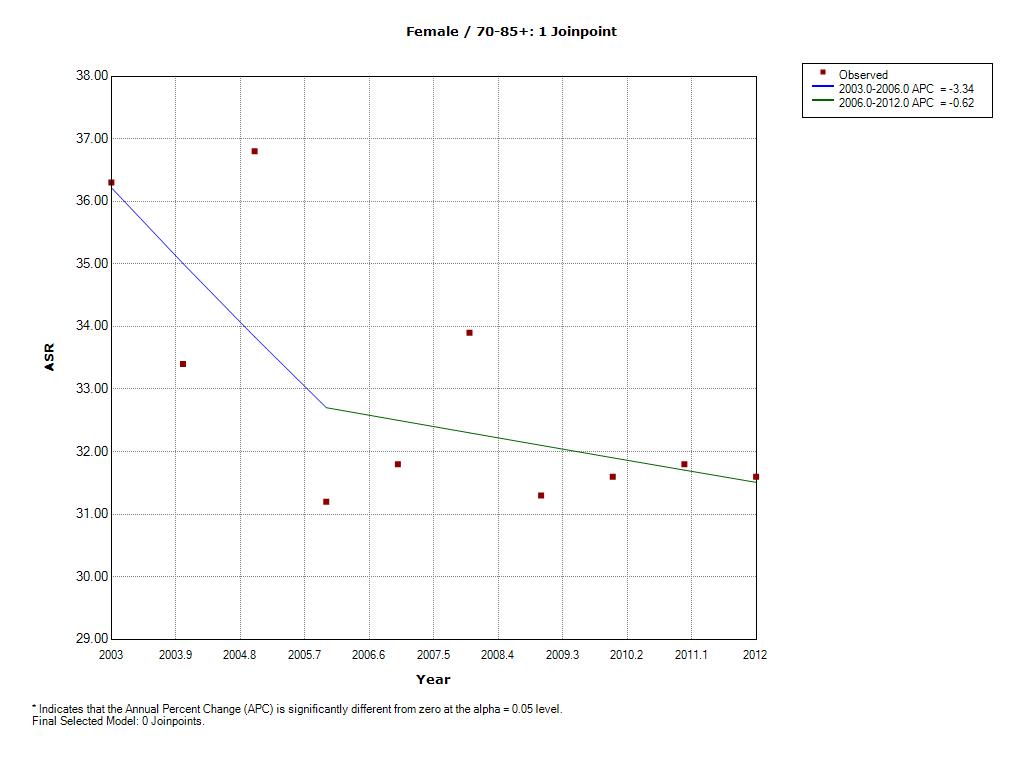

Supplement: Supplementary file 7 — Supplement Figure 7: incidence joinpoint. [file 12889_2024_19104_MOESM7_ESM.zip › Supplement Figure 7 incidence joinpoint/Australia female 70-85+.jpg]

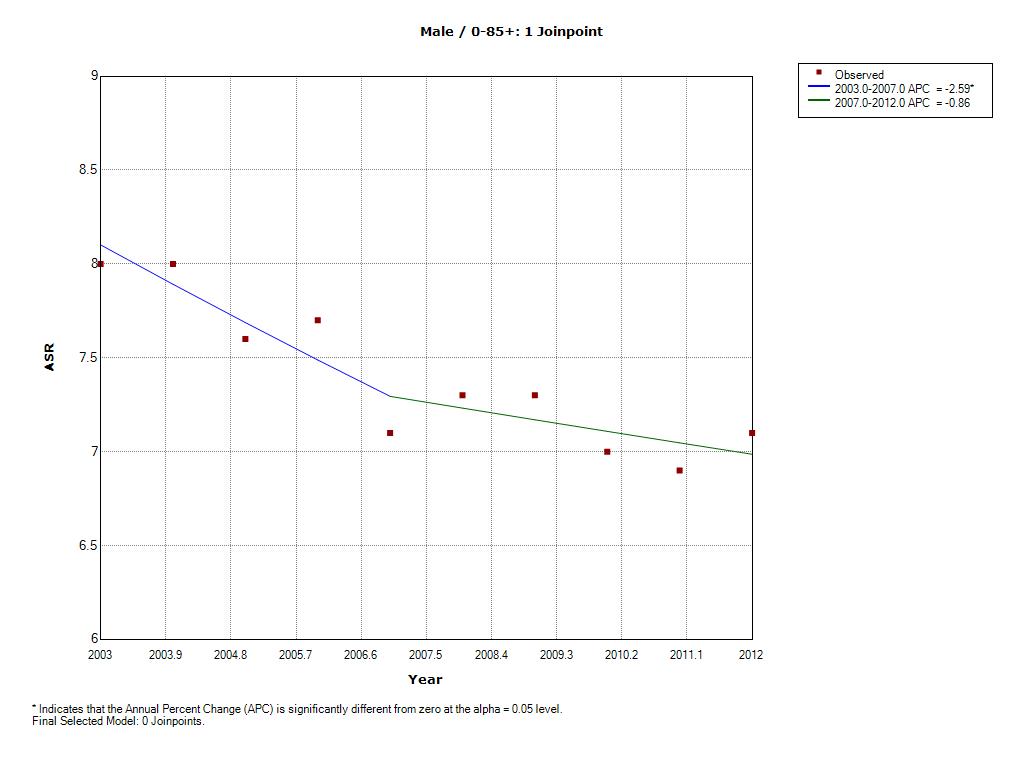

Supplement: Supplementary file 7 — Supplement Figure 7: incidence joinpoint. [file 12889_2024_19104_MOESM7_ESM.zip › Supplement Figure 7 incidence joinpoint/Australia male 0-85+.jpg]

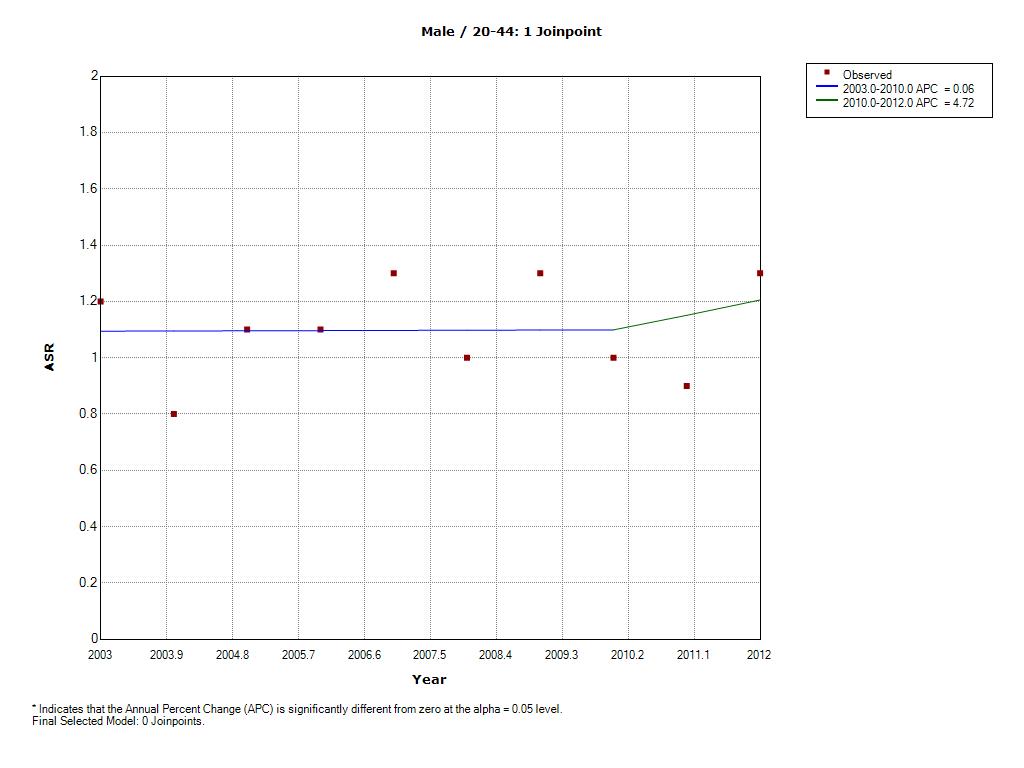

Supplement: Supplementary file 7 — Supplement Figure 7: incidence joinpoint. [file 12889_2024_19104_MOESM7_ESM.zip › Supplement Figure 7 incidence joinpoint/Australia male 20-44.jpg]

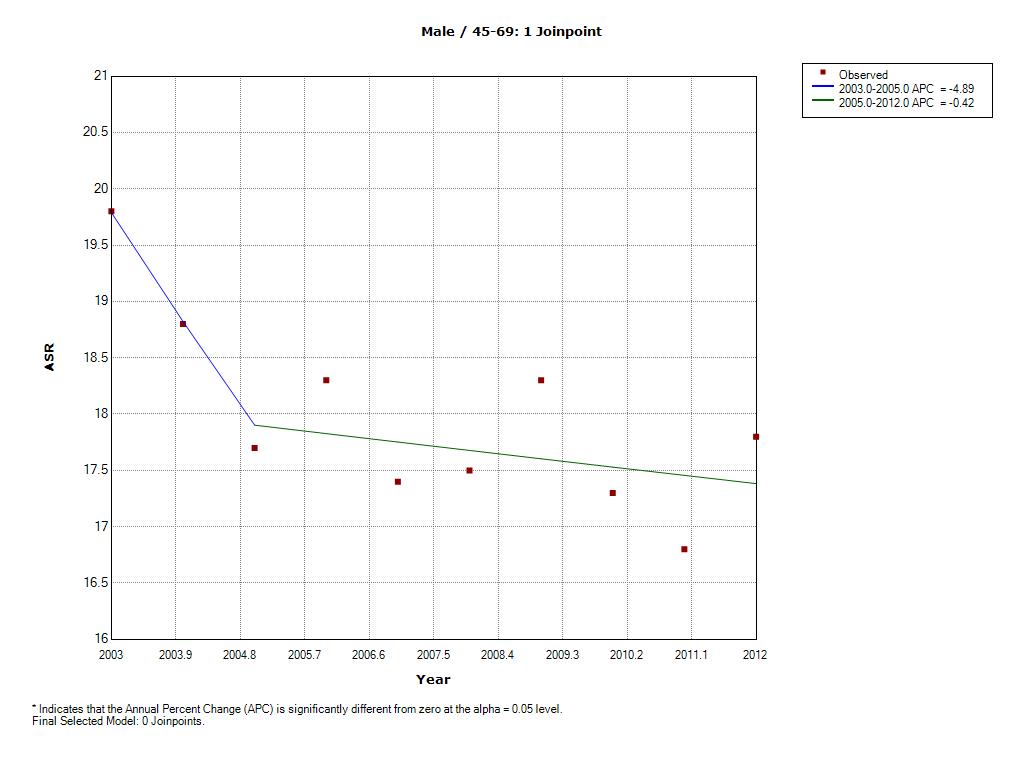

Supplement: Supplementary file 7 — Supplement Figure 7: incidence joinpoint. [file 12889_2024_19104_MOESM7_ESM.zip › Supplement Figure 7 incidence joinpoint/Australia male 45-69.jpg]

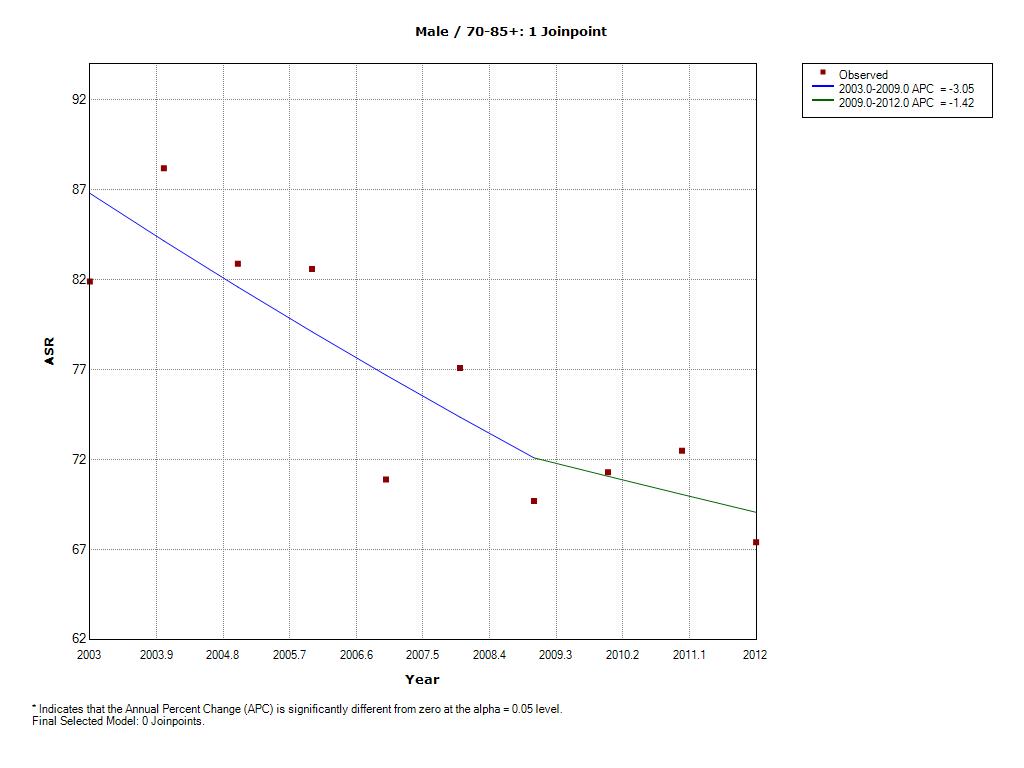

Supplement: Supplementary file 7 — Supplement Figure 7: incidence joinpoint. [file 12889_2024_19104_MOESM7_ESM.zip › Supplement Figure 7 incidence joinpoint/Australia male 70-85+.jpg]

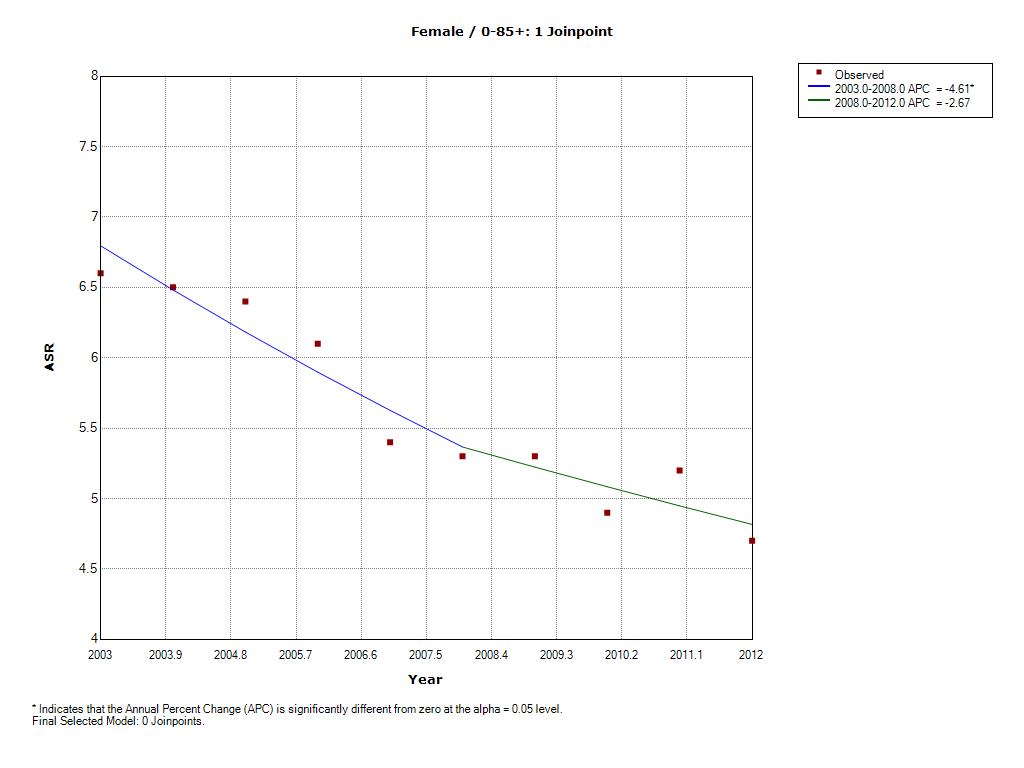

Supplement: Supplementary file 7 — Supplement Figure 7: incidence joinpoint. [file 12889_2024_19104_MOESM7_ESM.zip › Supplement Figure 7 incidence joinpoint/Austria female 0-85+.jpg]

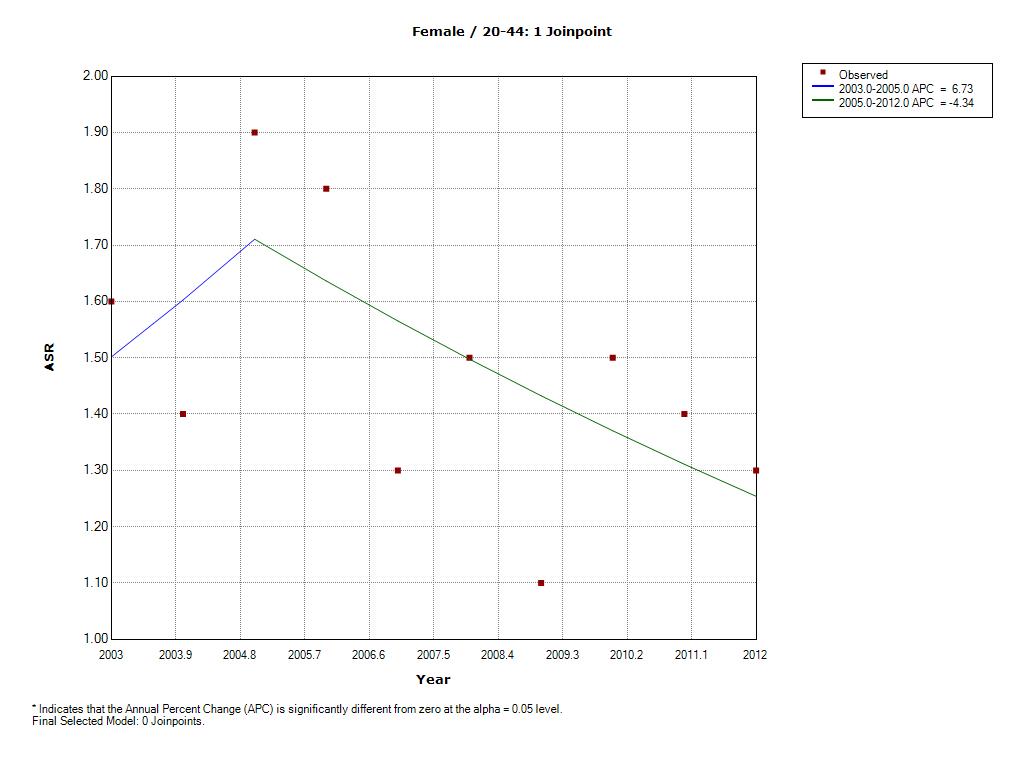

Supplement: Supplementary file 7 — Supplement Figure 7: incidence joinpoint. [file 12889_2024_19104_MOESM7_ESM.zip › Supplement Figure 7 incidence joinpoint/Austria female 20-44.jpg]

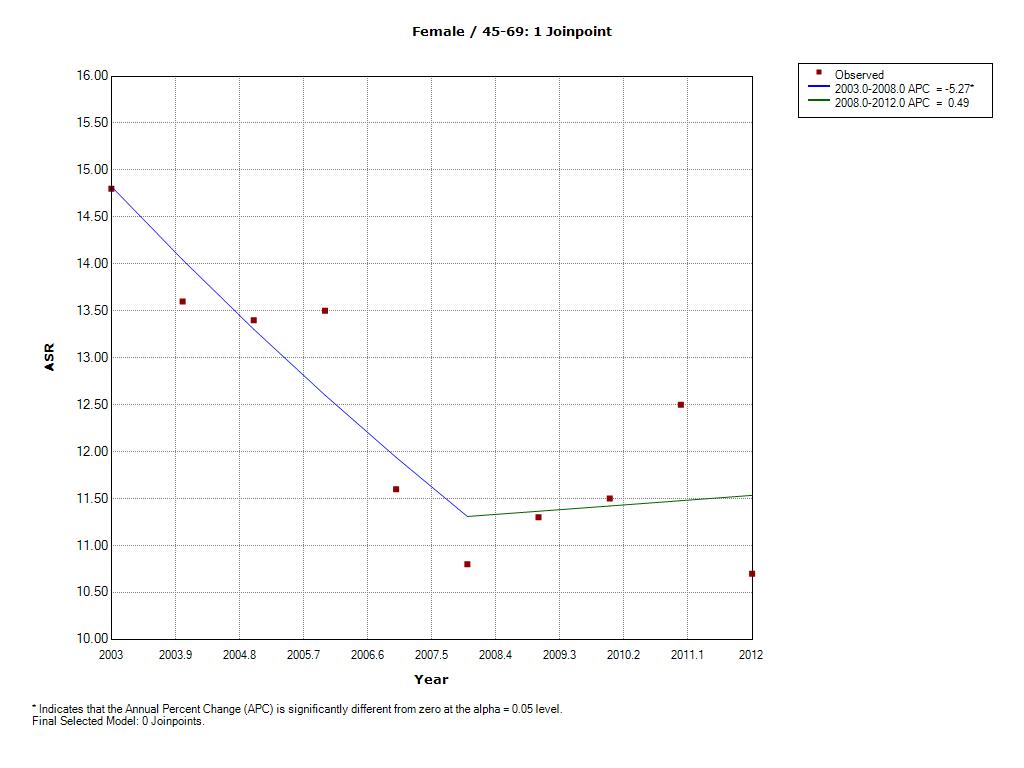

Supplement: Supplementary file 7 — Supplement Figure 7: incidence joinpoint. [file 12889_2024_19104_MOESM7_ESM.zip › Supplement Figure 7 incidence joinpoint/Austria female 45-69.jpg]

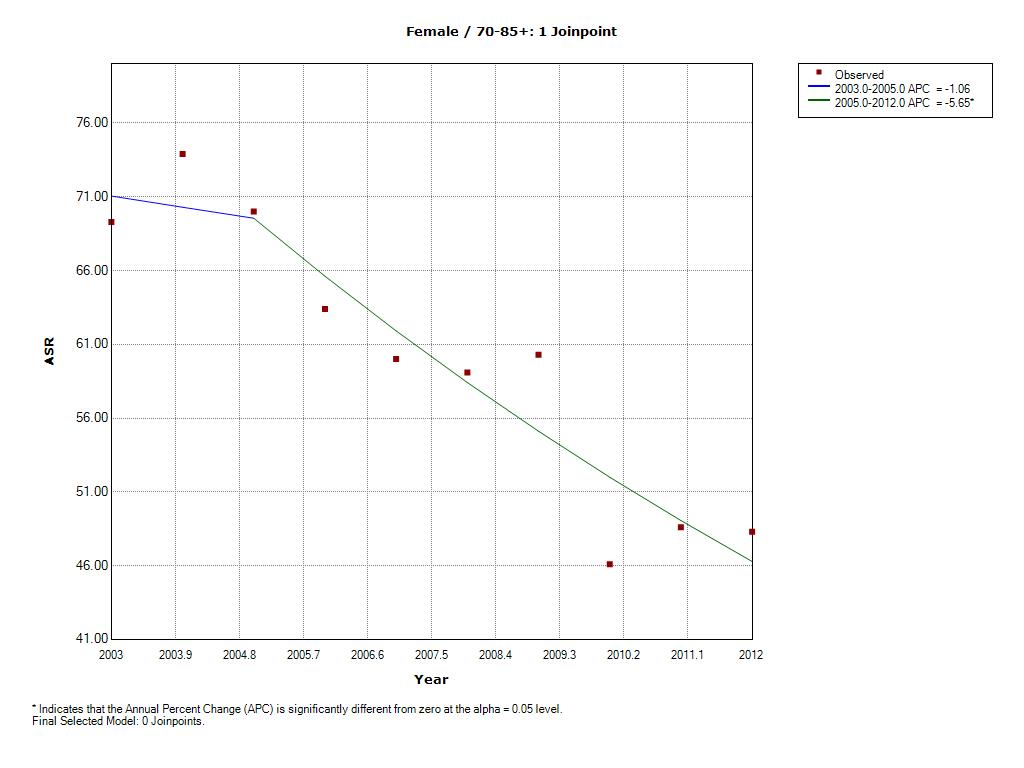

Supplement: Supplementary file 7 — Supplement Figure 7: incidence joinpoint. [file 12889_2024_19104_MOESM7_ESM.zip › Supplement Figure 7 incidence joinpoint/Austria female 70-85+.jpg]

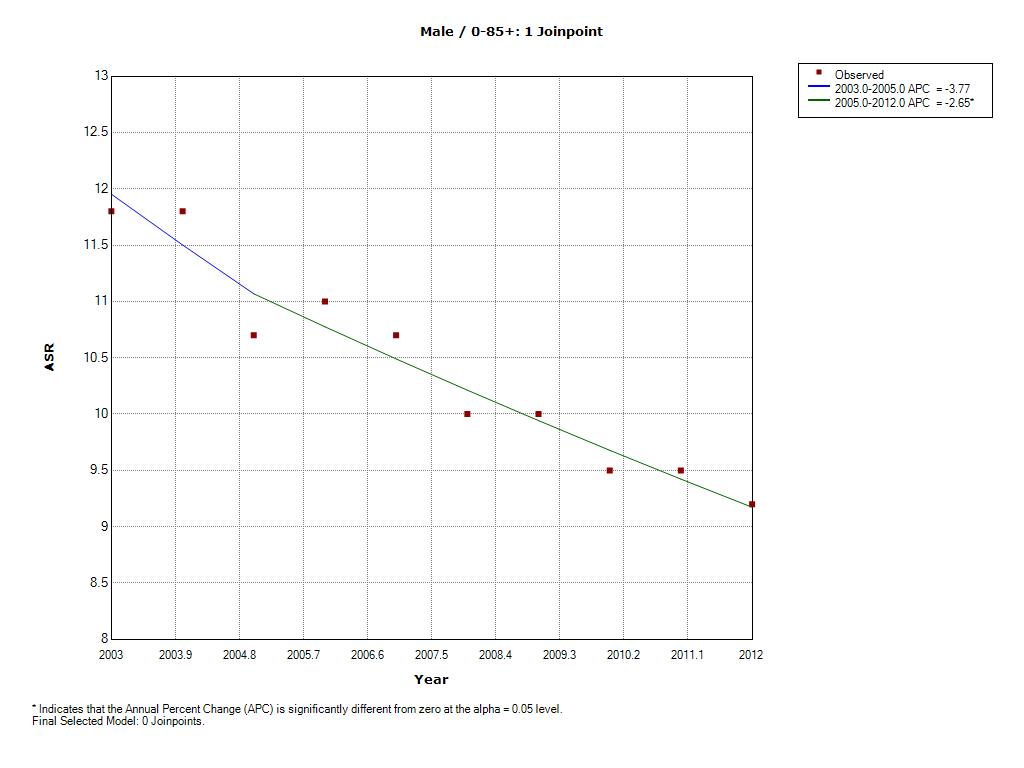

Supplement: Supplementary file 7 — Supplement Figure 7: incidence joinpoint. [file 12889_2024_19104_MOESM7_ESM.zip › Supplement Figure 7 incidence joinpoint/Austria male 0-85+.jpg]

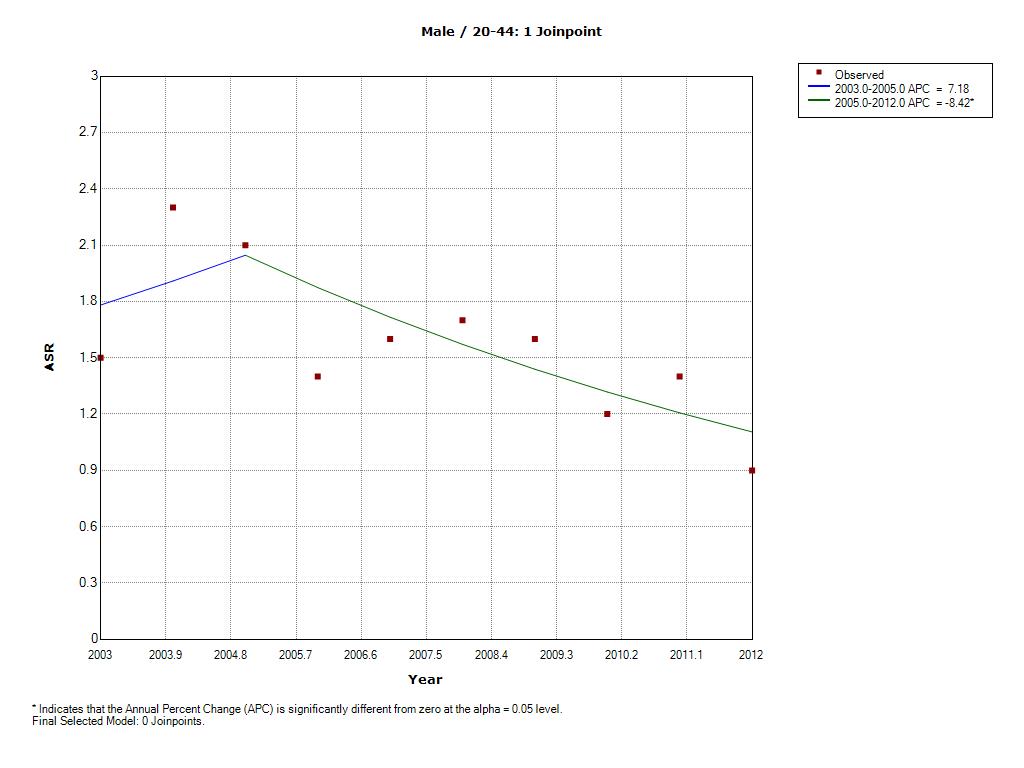

Supplement: Supplementary file 7 — Supplement Figure 7: incidence joinpoint. [file 12889_2024_19104_MOESM7_ESM.zip › Supplement Figure 7 incidence joinpoint/Austria male 20-44.jpg]

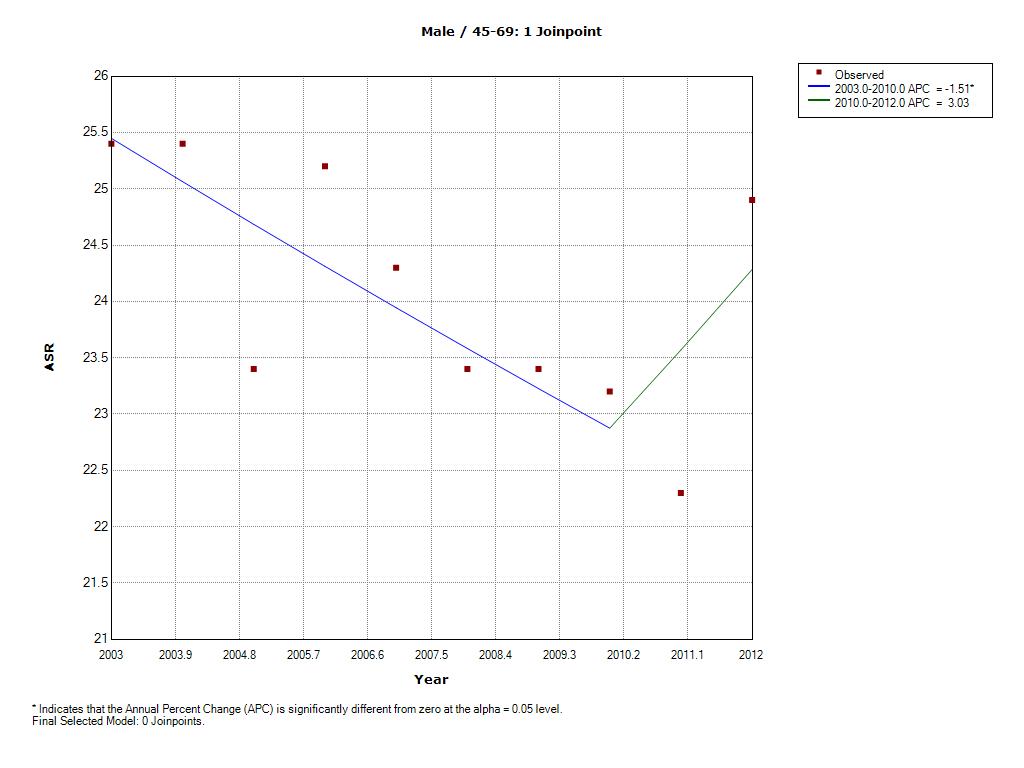

Supplement: Supplementary file 7 — Supplement Figure 7: incidence joinpoint. [file 12889_2024_19104_MOESM7_ESM.zip › Supplement Figure 7 incidence joinpoint/Austria male 45-69.jpg]

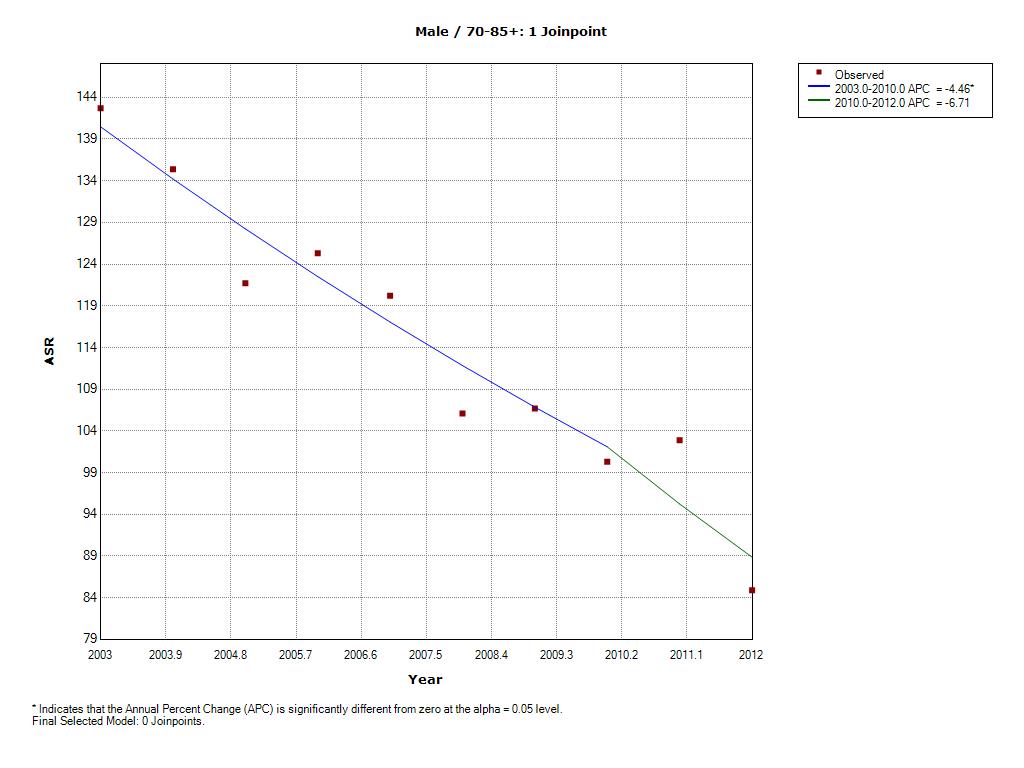

Supplement: Supplementary file 7 — Supplement Figure 7: incidence joinpoint. [file 12889_2024_19104_MOESM7_ESM.zip › Supplement Figure 7 incidence joinpoint/Austria male 70-85+.jpg]

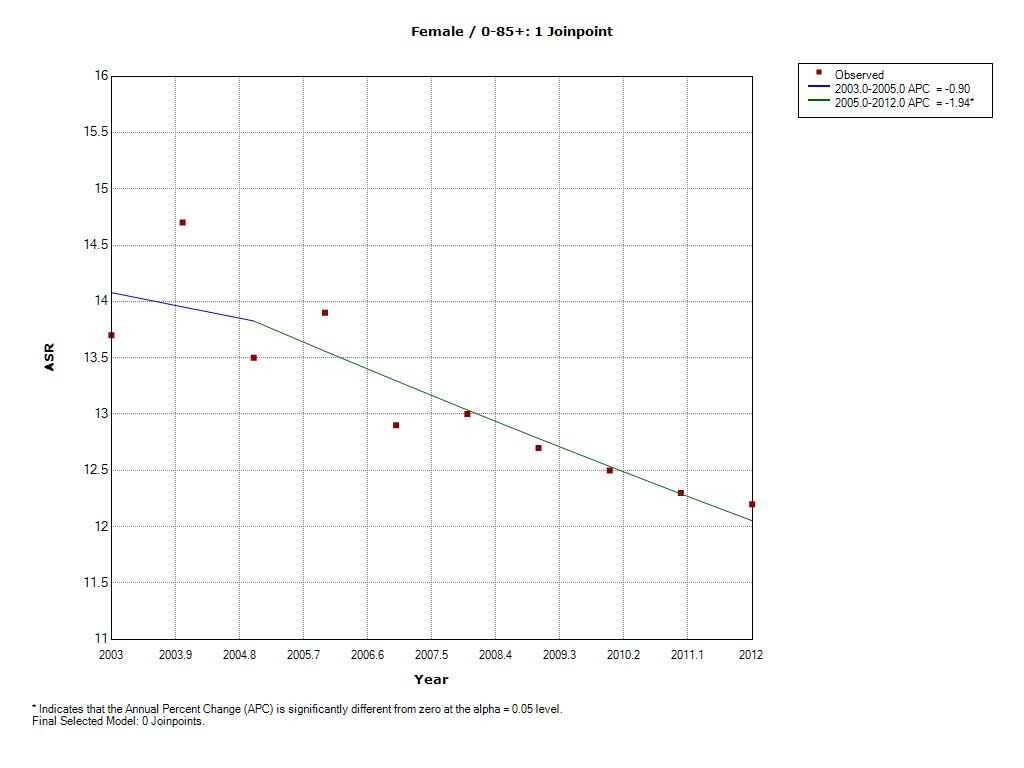

Supplement: Supplementary file 7 — Supplement Figure 7: incidence joinpoint. [file 12889_2024_19104_MOESM7_ESM.zip › Supplement Figure 7 incidence joinpoint/Belarus female 0-85+.jpg]

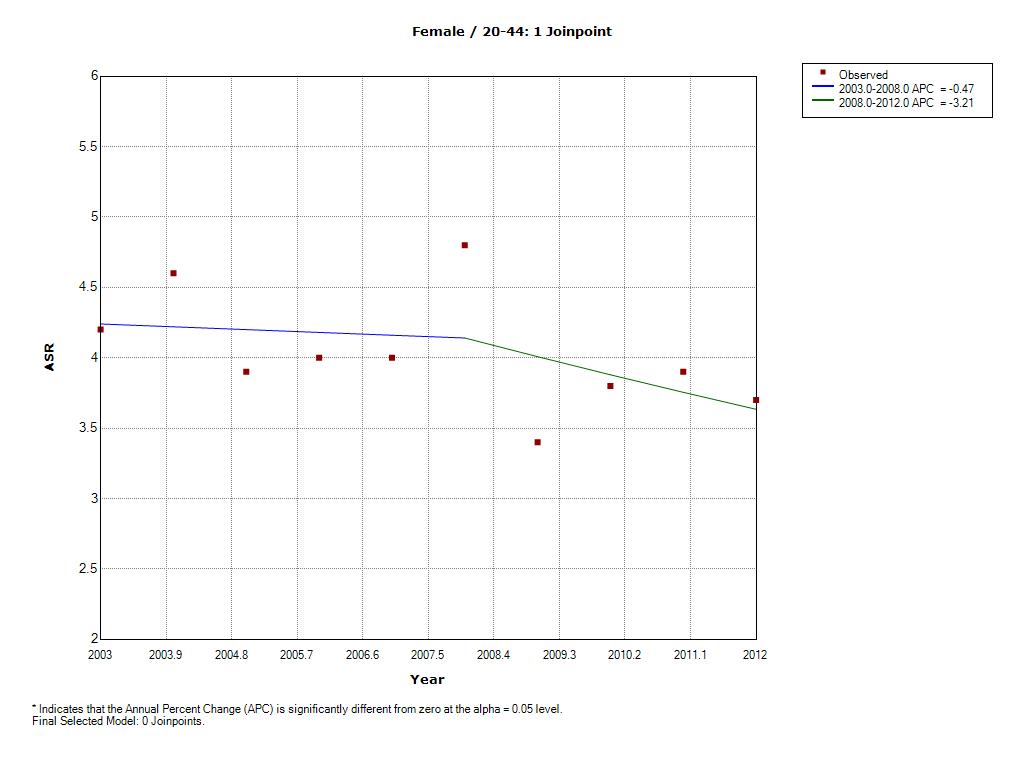

Supplement: Supplementary file 7 — Supplement Figure 7: incidence joinpoint. [file 12889_2024_19104_MOESM7_ESM.zip › Supplement Figure 7 incidence joinpoint/Belarus female 20-44.jpg]

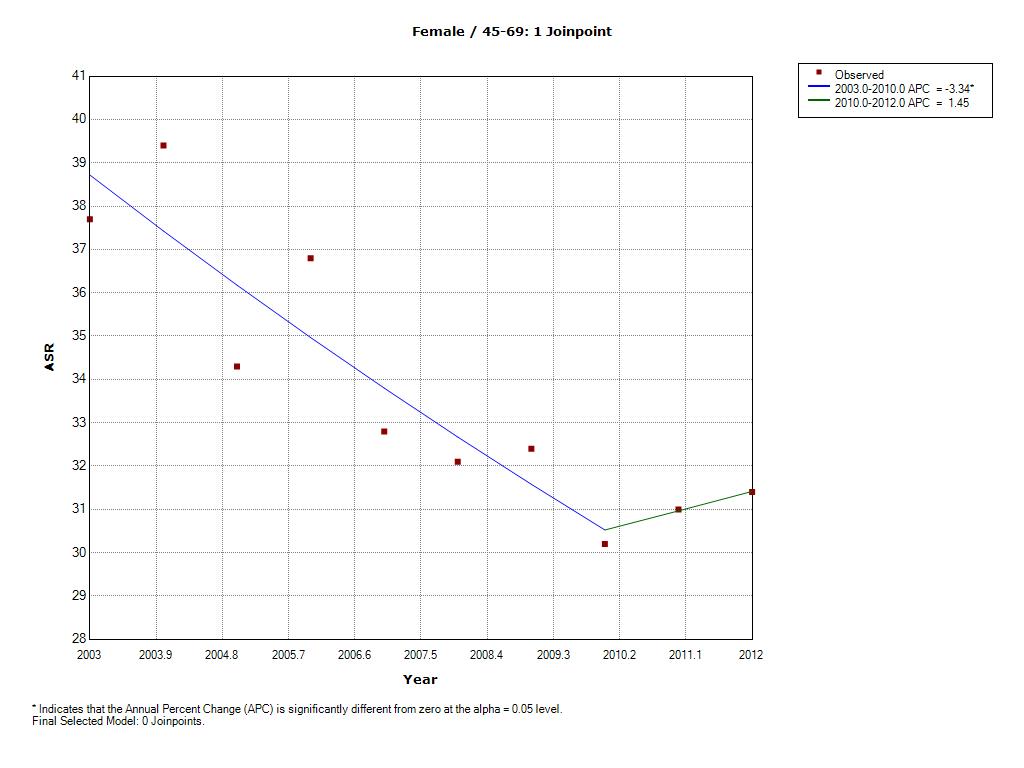

Supplement: Supplementary file 7 — Supplement Figure 7: incidence joinpoint. [file 12889_2024_19104_MOESM7_ESM.zip › Supplement Figure 7 incidence joinpoint/Belarus female 45-69.jpg]

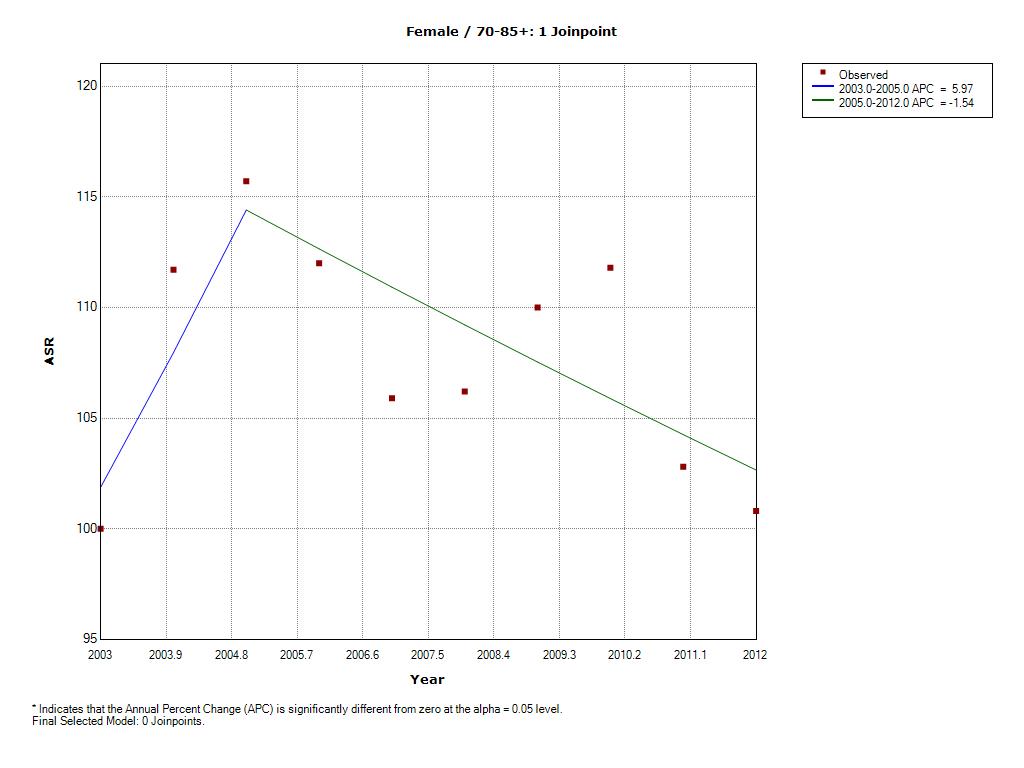

Supplement: Supplementary file 7 — Supplement Figure 7: incidence joinpoint. [file 12889_2024_19104_MOESM7_ESM.zip › Supplement Figure 7 incidence joinpoint/Belarus female 70-85+.jpg]

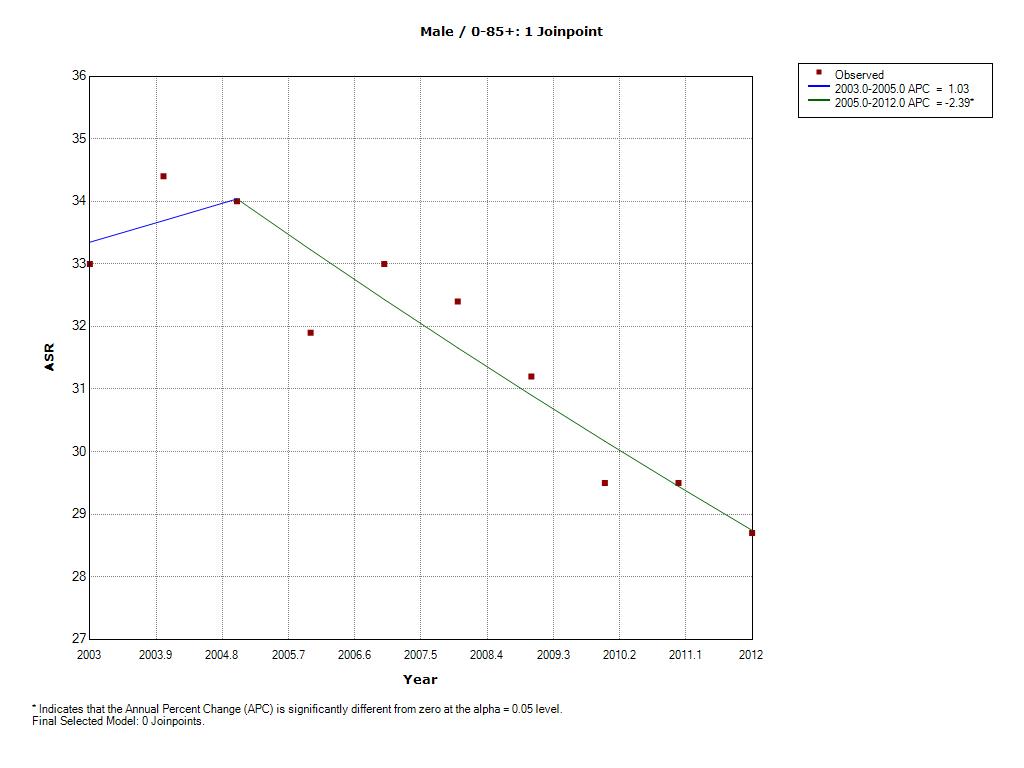

Supplement: Supplementary file 7 — Supplement Figure 7: incidence joinpoint. [file 12889_2024_19104_MOESM7_ESM.zip › Supplement Figure 7 incidence joinpoint/Belarus male 0-85+.jpg]

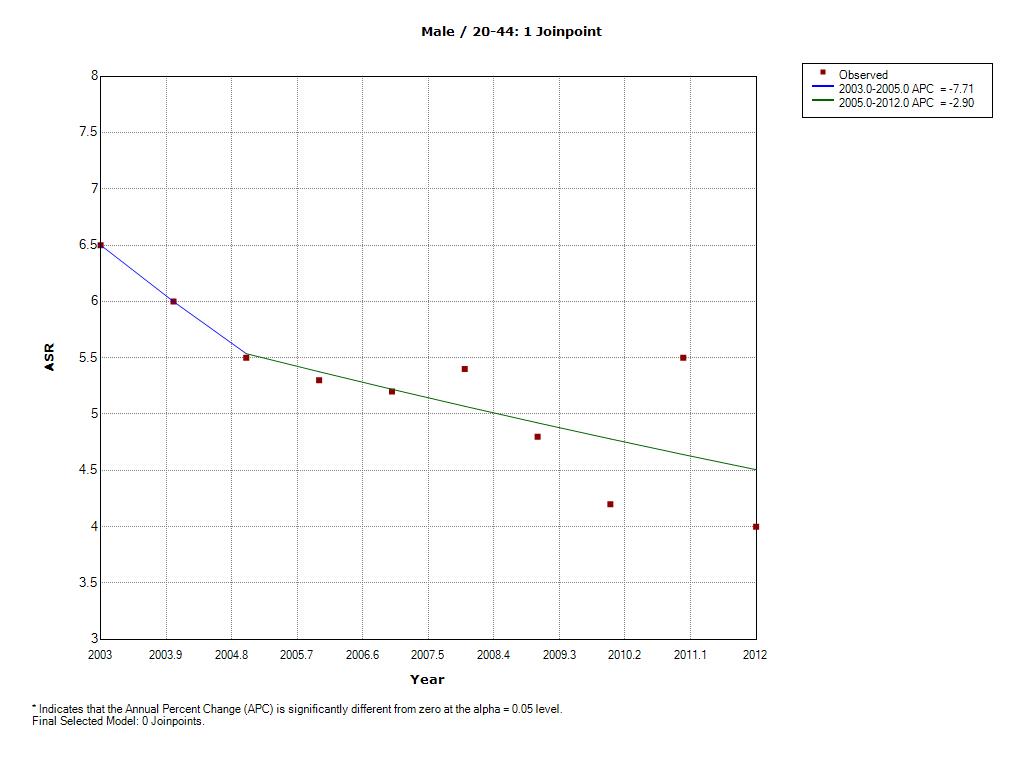

Supplement: Supplementary file 7 — Supplement Figure 7: incidence joinpoint. [file 12889_2024_19104_MOESM7_ESM.zip › Supplement Figure 7 incidence joinpoint/Belarus male 20-44.jpg]

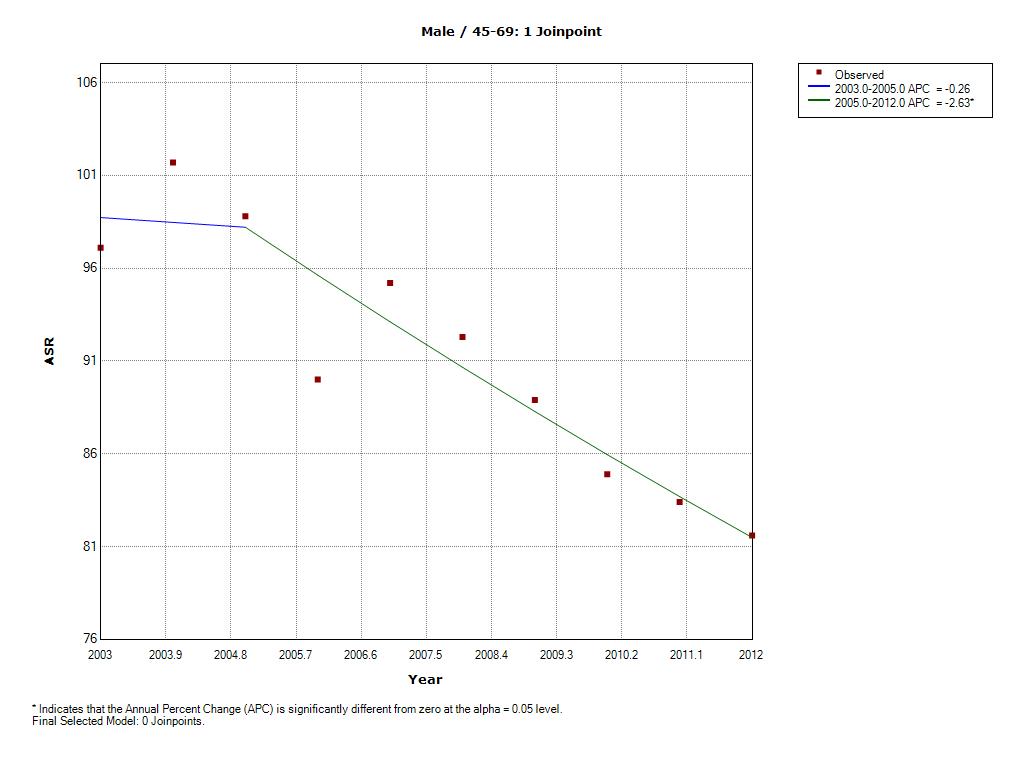

Supplement: Supplementary file 7 — Supplement Figure 7: incidence joinpoint. [file 12889_2024_19104_MOESM7_ESM.zip › Supplement Figure 7 incidence joinpoint/Belarus male 45-69.jpg]

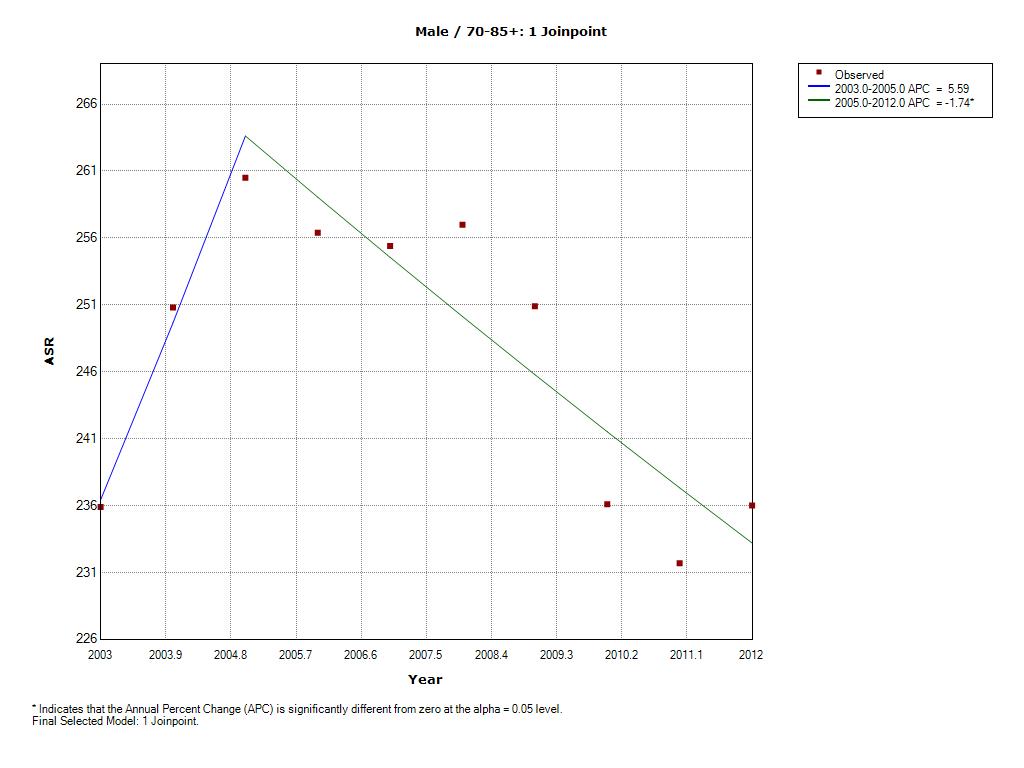

Supplement: Supplementary file 7 — Supplement Figure 7: incidence joinpoint. [file 12889_2024_19104_MOESM7_ESM.zip › Supplement Figure 7 incidence joinpoint/Belarus male 70-85+.jpg]

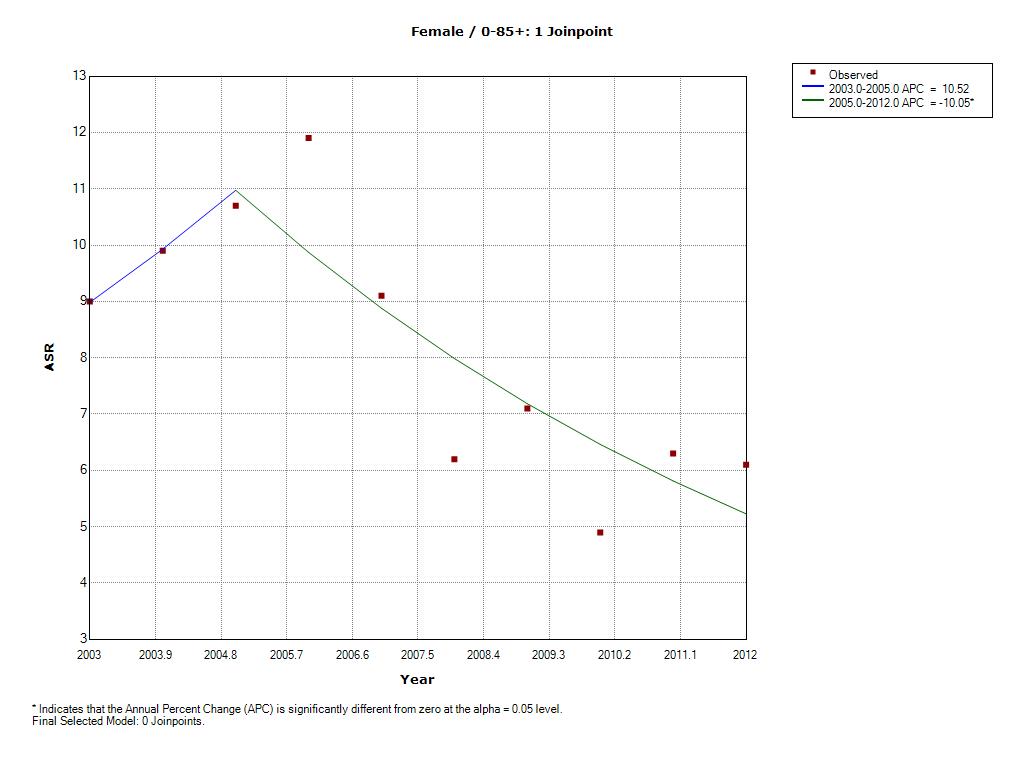

Supplement: Supplementary file 7 — Supplement Figure 7: incidence joinpoint. [file 12889_2024_19104_MOESM7_ESM.zip › Supplement Figure 7 incidence joinpoint/Brazil female 0-85+.jpg]

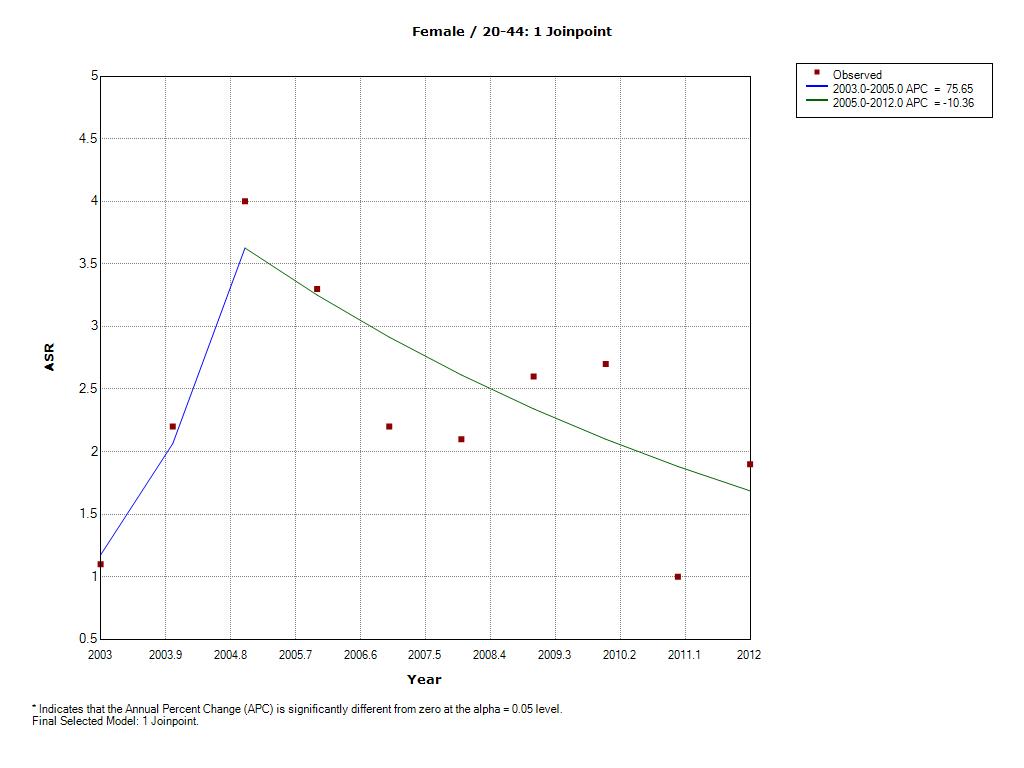

Supplement: Supplementary file 7 — Supplement Figure 7: incidence joinpoint. [file 12889_2024_19104_MOESM7_ESM.zip › Supplement Figure 7 incidence joinpoint/Brazil female 20-44.jpg]

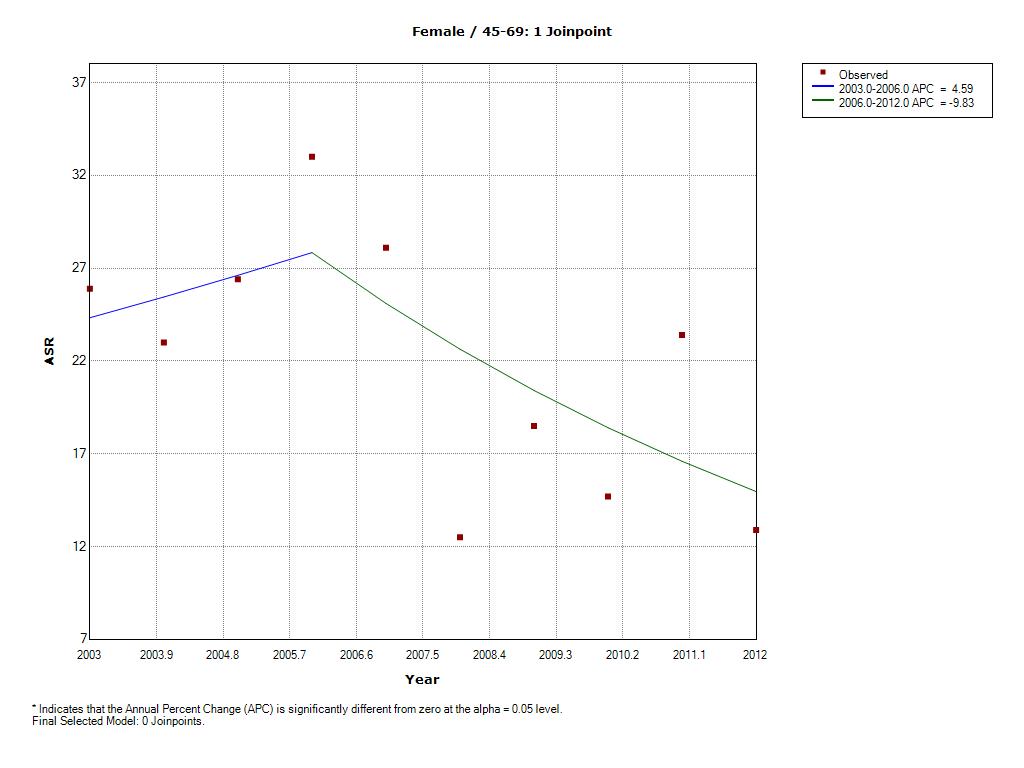

Supplement: Supplementary file 7 — Supplement Figure 7: incidence joinpoint. [file 12889_2024_19104_MOESM7_ESM.zip › Supplement Figure 7 incidence joinpoint/Brazil female 45-69.jpg]

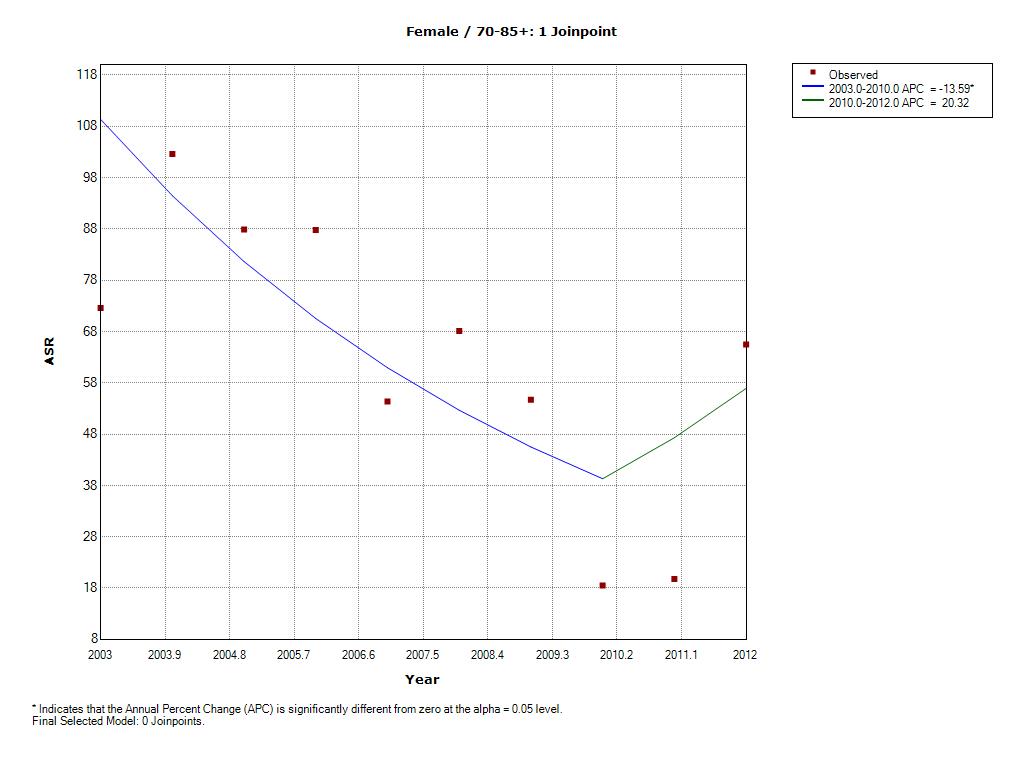

Supplement: Supplementary file 7 — Supplement Figure 7: incidence joinpoint. [file 12889_2024_19104_MOESM7_ESM.zip › Supplement Figure 7 incidence joinpoint/Brazil female 70-85+.jpg]

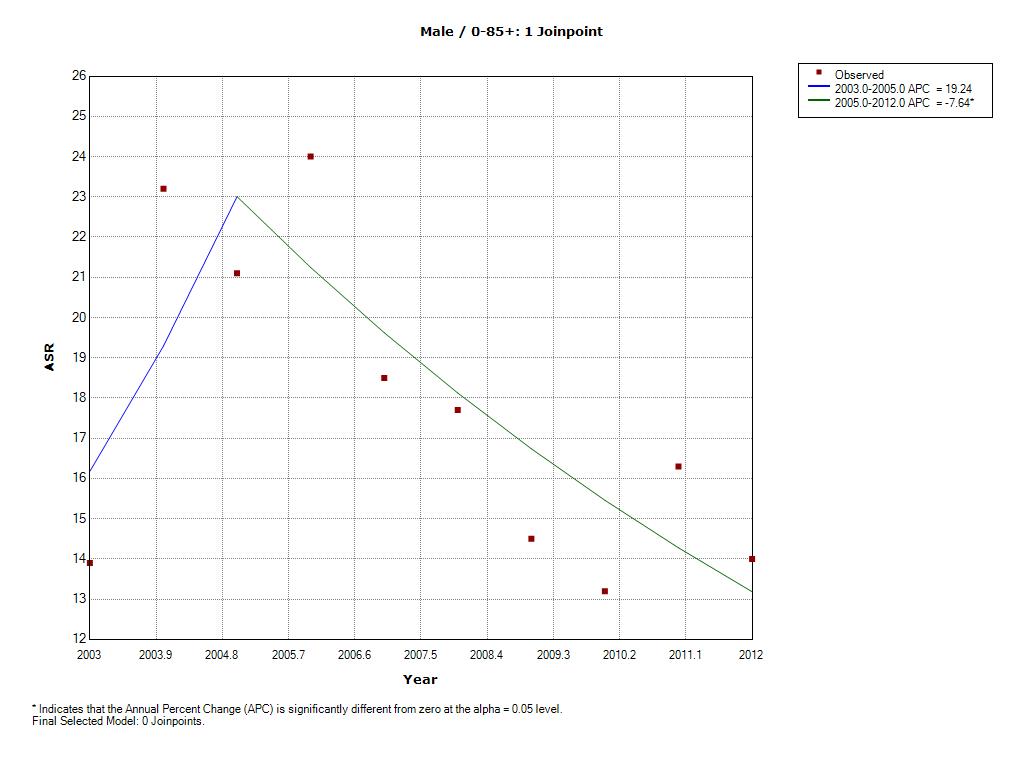

Supplement: Supplementary file 7 — Supplement Figure 7: incidence joinpoint. [file 12889_2024_19104_MOESM7_ESM.zip › Supplement Figure 7 incidence joinpoint/Brazil male 0-85+.jpg]

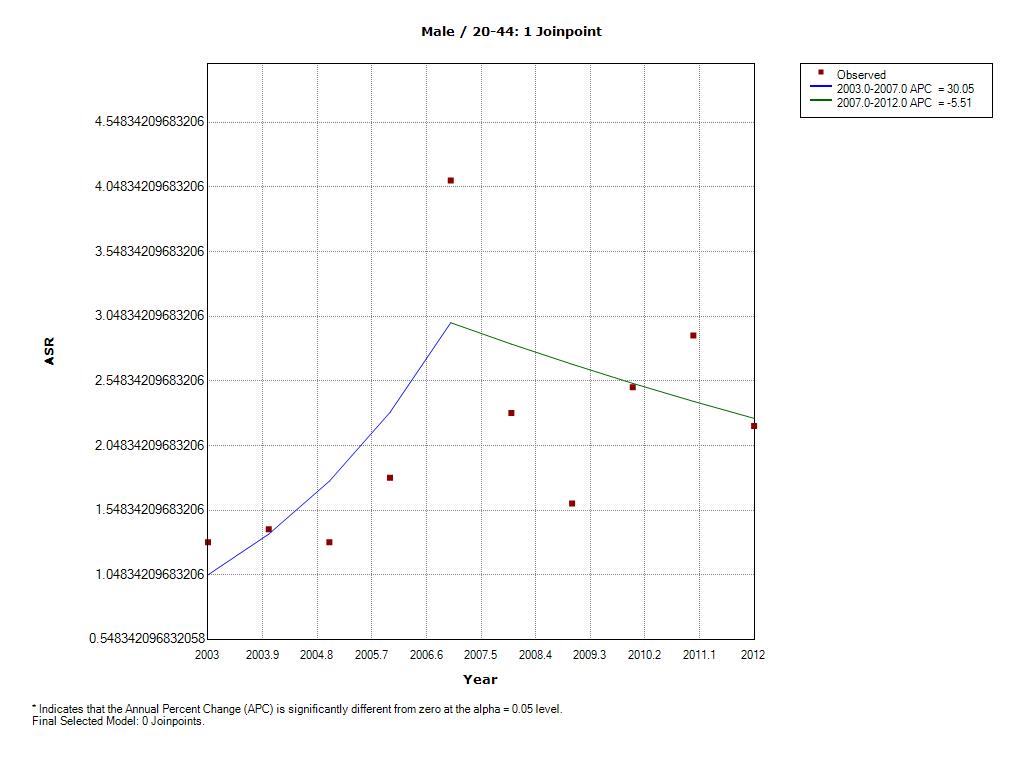

Supplement: Supplementary file 7 — Supplement Figure 7: incidence joinpoint. [file 12889_2024_19104_MOESM7_ESM.zip › Supplement Figure 7 incidence joinpoint/Brazil male 20-44.jpg]

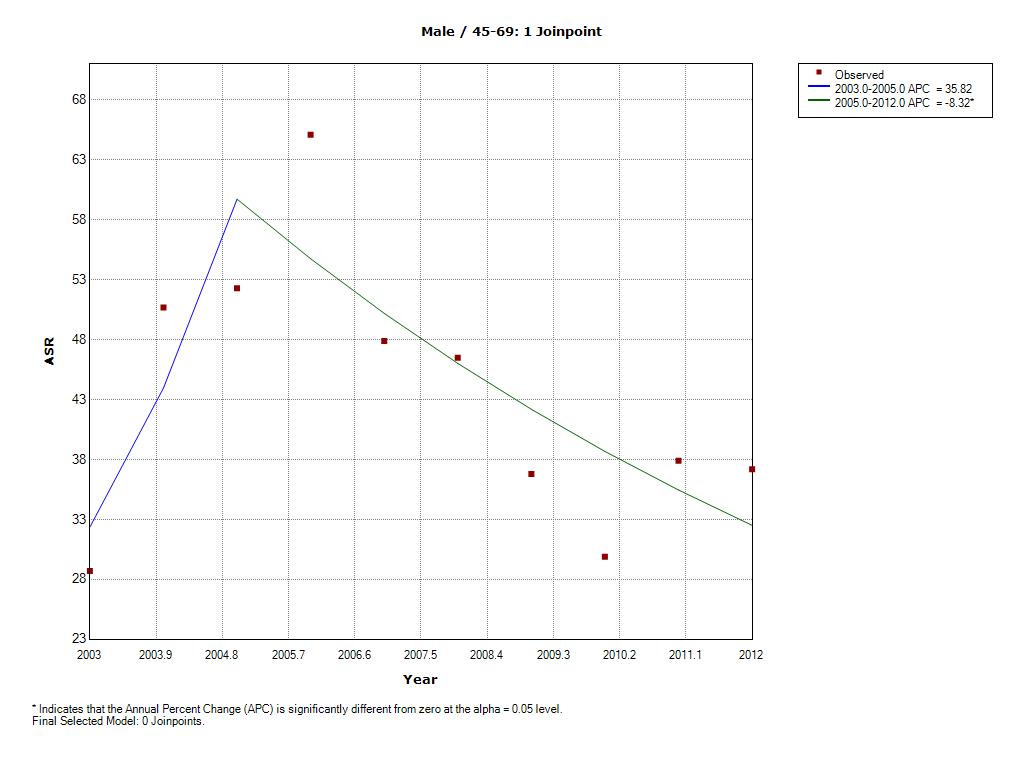

Supplement: Supplementary file 7 — Supplement Figure 7: incidence joinpoint. [file 12889_2024_19104_MOESM7_ESM.zip › Supplement Figure 7 incidence joinpoint/Brazil male 45-69.jpg]

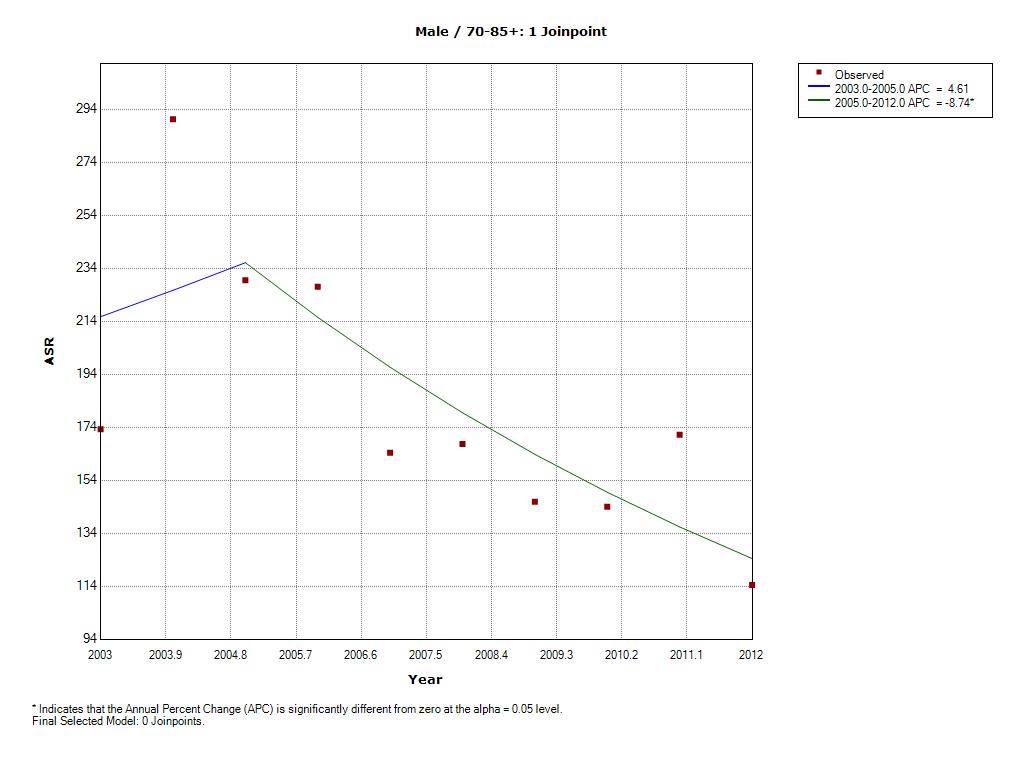

Supplement: Supplementary file 7 — Supplement Figure 7: incidence joinpoint. [file 12889_2024_19104_MOESM7_ESM.zip › Supplement Figure 7 incidence joinpoint/Brazil male 70-85+.jpg]

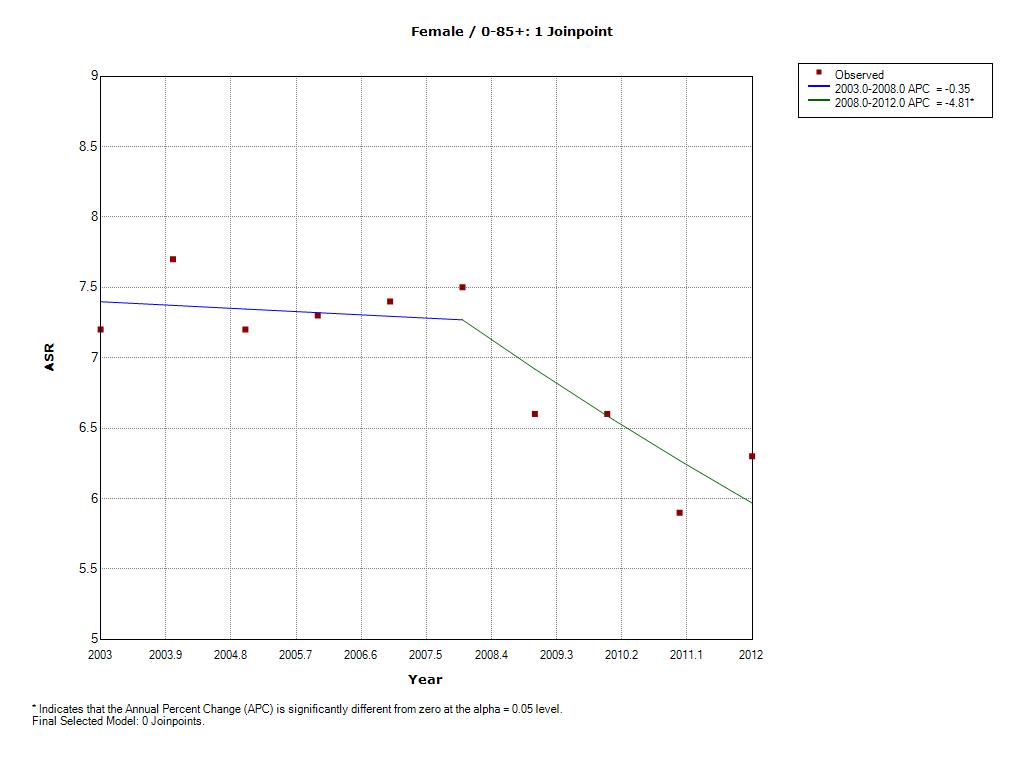

Supplement: Supplementary file 7 — Supplement Figure 7: incidence joinpoint. [file 12889_2024_19104_MOESM7_ESM.zip › Supplement Figure 7 incidence joinpoint/Bulgaria female 0-85+.jpg]

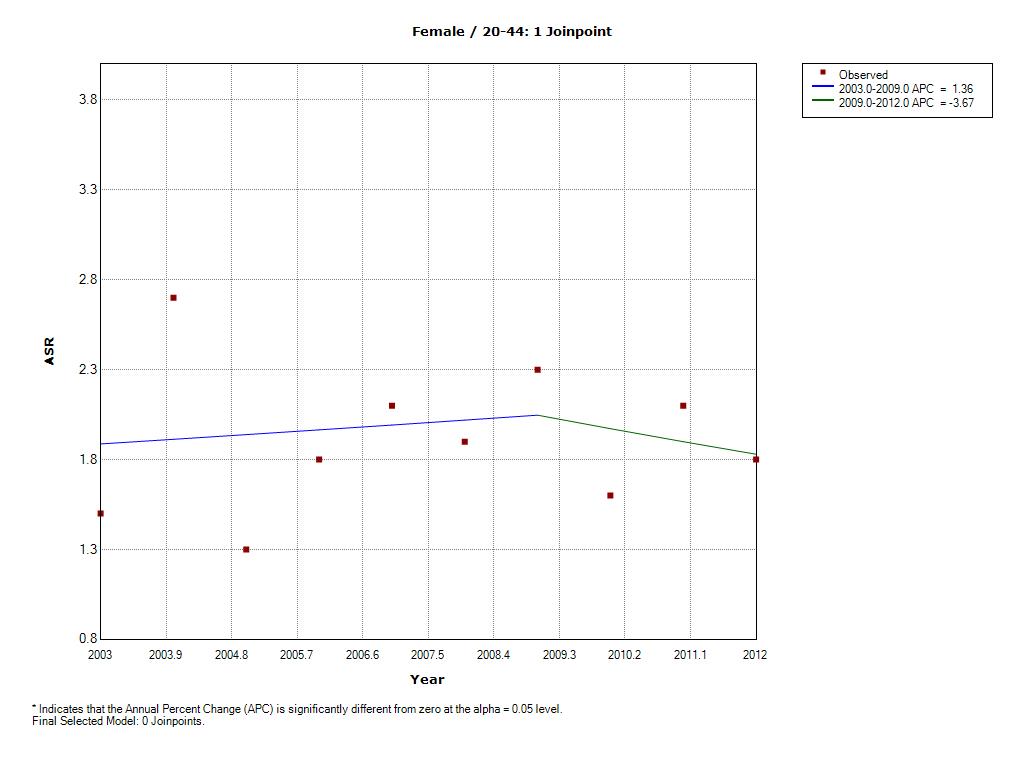

Supplement: Supplementary file 7 — Supplement Figure 7: incidence joinpoint. [file 12889_2024_19104_MOESM7_ESM.zip › Supplement Figure 7 incidence joinpoint/Bulgaria female 20-44.jpg]

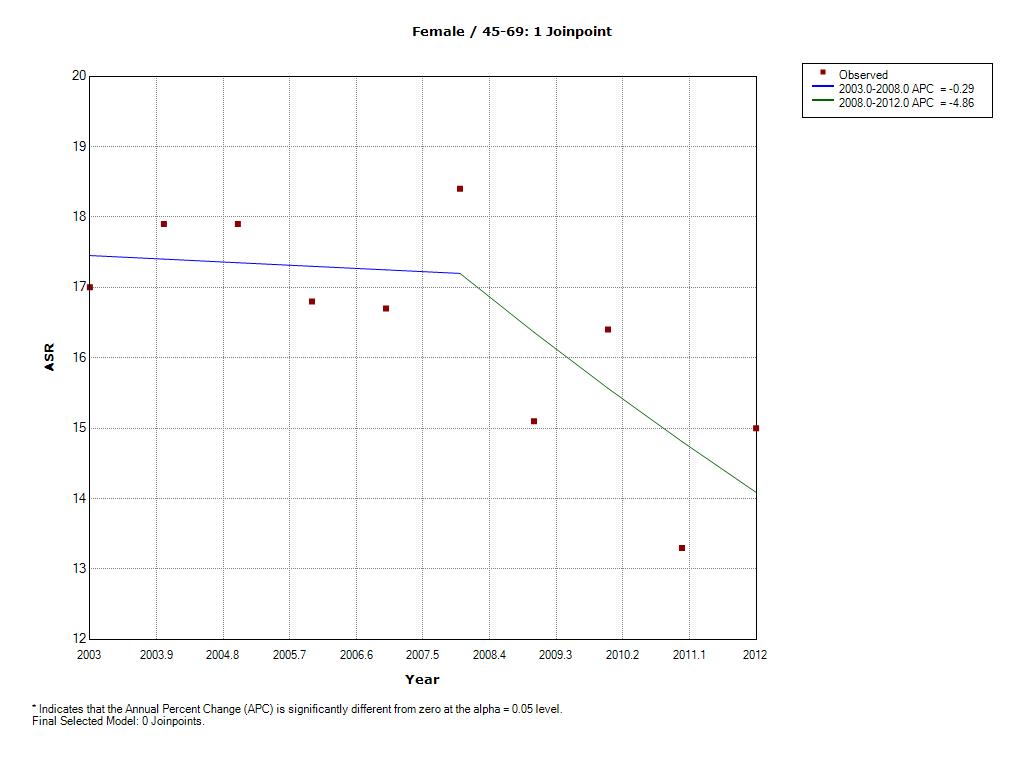

Supplement: Supplementary file 7 — Supplement Figure 7: incidence joinpoint. [file 12889_2024_19104_MOESM7_ESM.zip › Supplement Figure 7 incidence joinpoint/Bulgaria female 45-69.jpg]

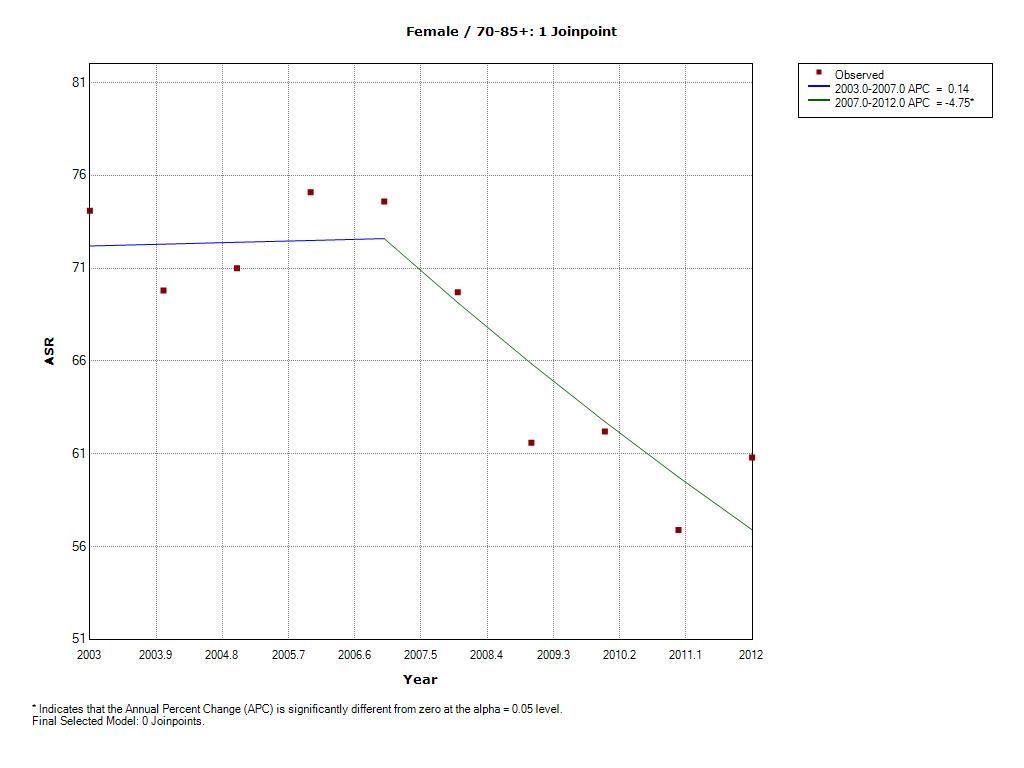

Supplement: Supplementary file 7 — Supplement Figure 7: incidence joinpoint. [file 12889_2024_19104_MOESM7_ESM.zip › Supplement Figure 7 incidence joinpoint/Bulgaria female 70-85+.jpg]

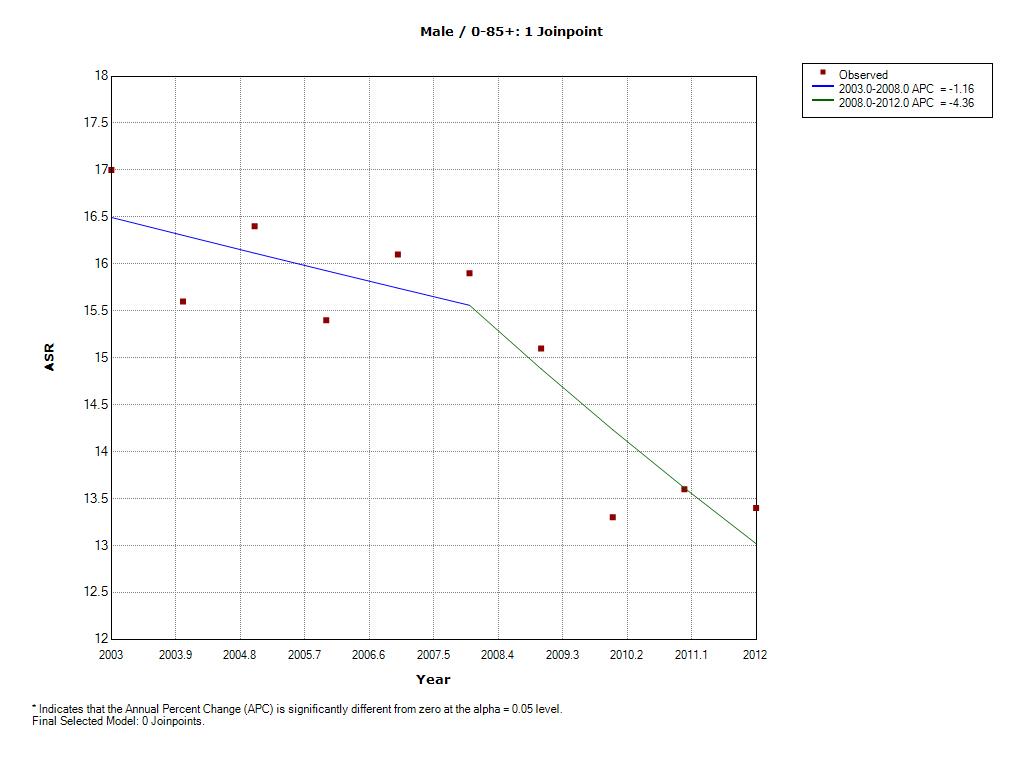

Supplement: Supplementary file 7 — Supplement Figure 7: incidence joinpoint. [file 12889_2024_19104_MOESM7_ESM.zip › Supplement Figure 7 incidence joinpoint/Bulgaria male 0-85+.jpg]

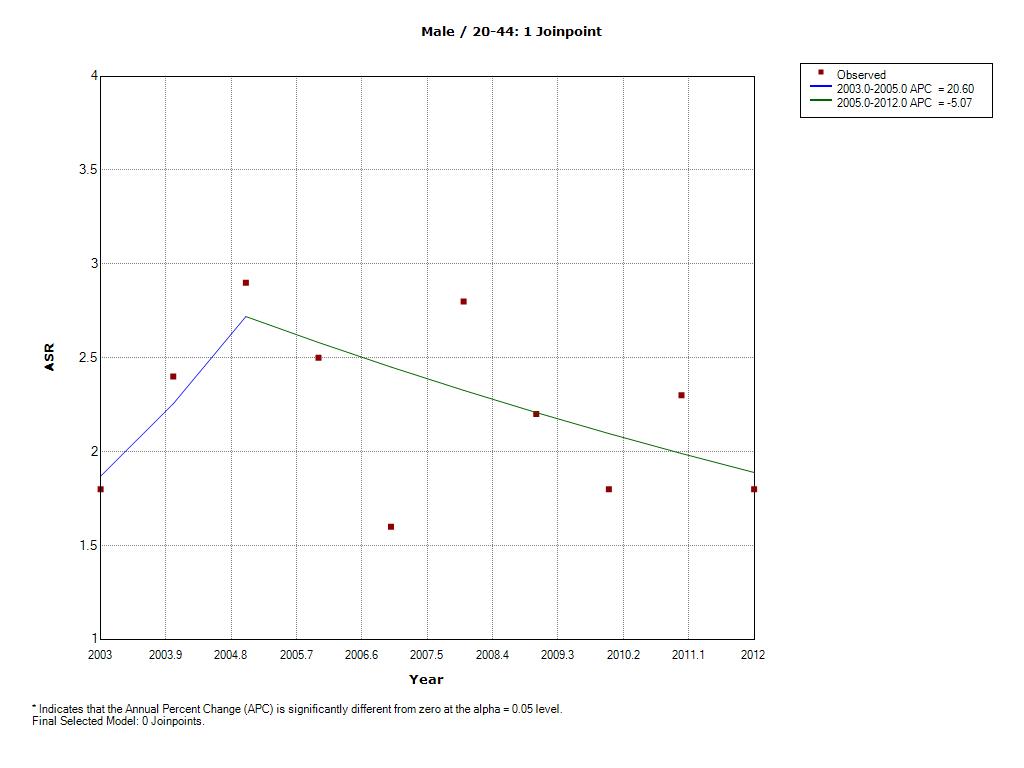

Supplement: Supplementary file 7 — Supplement Figure 7: incidence joinpoint. [file 12889_2024_19104_MOESM7_ESM.zip › Supplement Figure 7 incidence joinpoint/Bulgaria male 20-44.jpg]

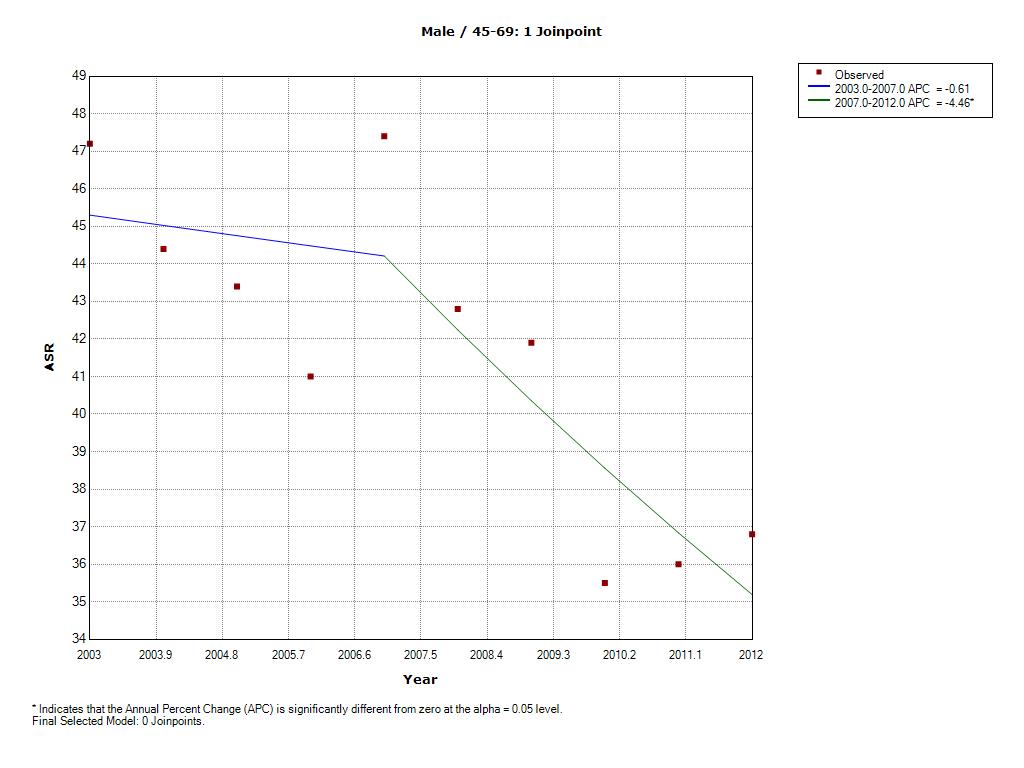

Supplement: Supplementary file 7 — Supplement Figure 7: incidence joinpoint. [file 12889_2024_19104_MOESM7_ESM.zip › Supplement Figure 7 incidence joinpoint/Bulgaria male 45-69.jpg]

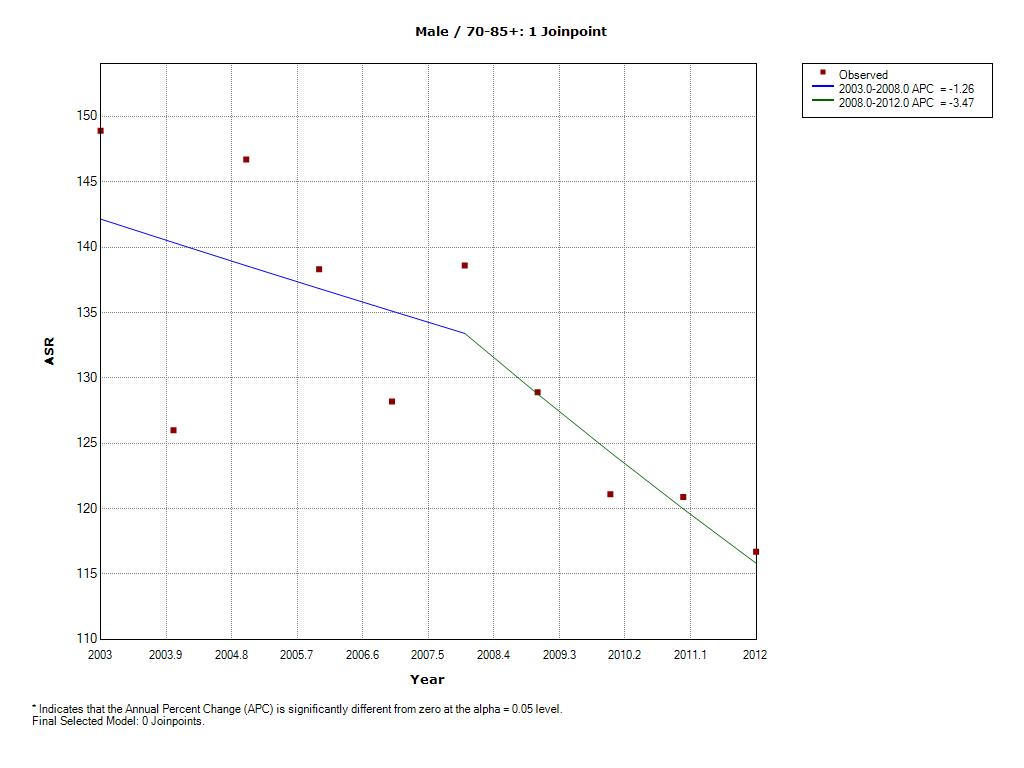

Supplement: Supplementary file 7 — Supplement Figure 7: incidence joinpoint. [file 12889_2024_19104_MOESM7_ESM.zip › Supplement Figure 7 incidence joinpoint/Bulgaria male 70-85+.jpg]

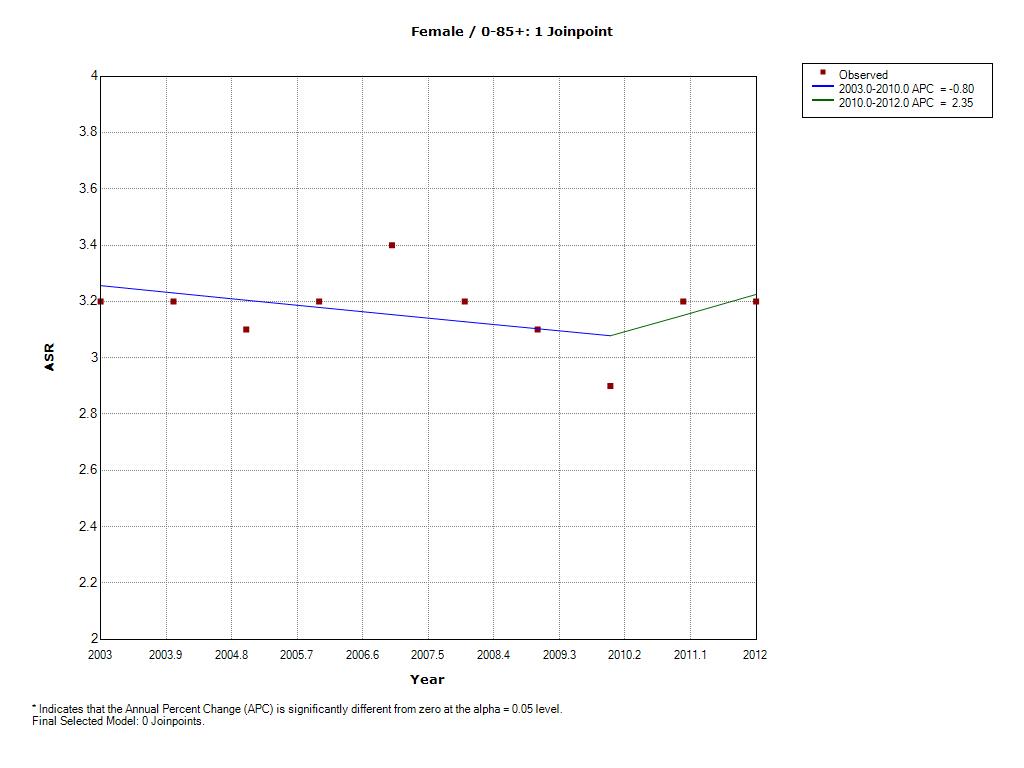

Supplement: Supplementary file 7 — Supplement Figure 7: incidence joinpoint. [file 12889_2024_19104_MOESM7_ESM.zip › Supplement Figure 7 incidence joinpoint/Canada female 0-85+.jpg]

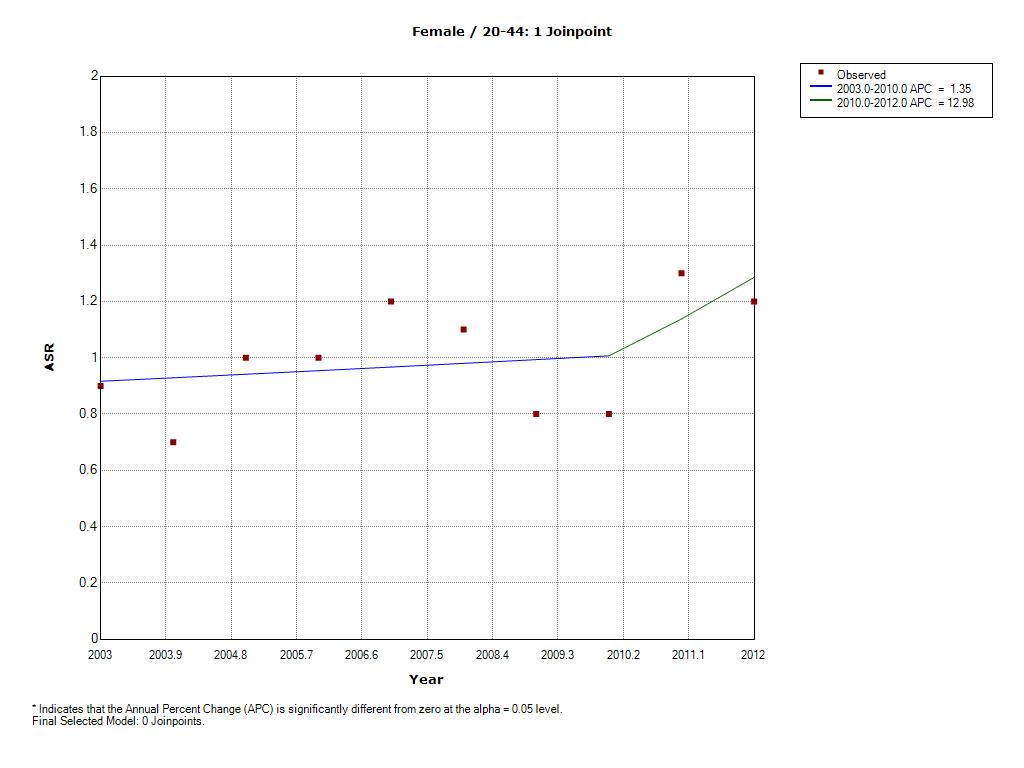

Supplement: Supplementary file 7 — Supplement Figure 7: incidence joinpoint. [file 12889_2024_19104_MOESM7_ESM.zip › Supplement Figure 7 incidence joinpoint/Canada female 20-44.jpg]

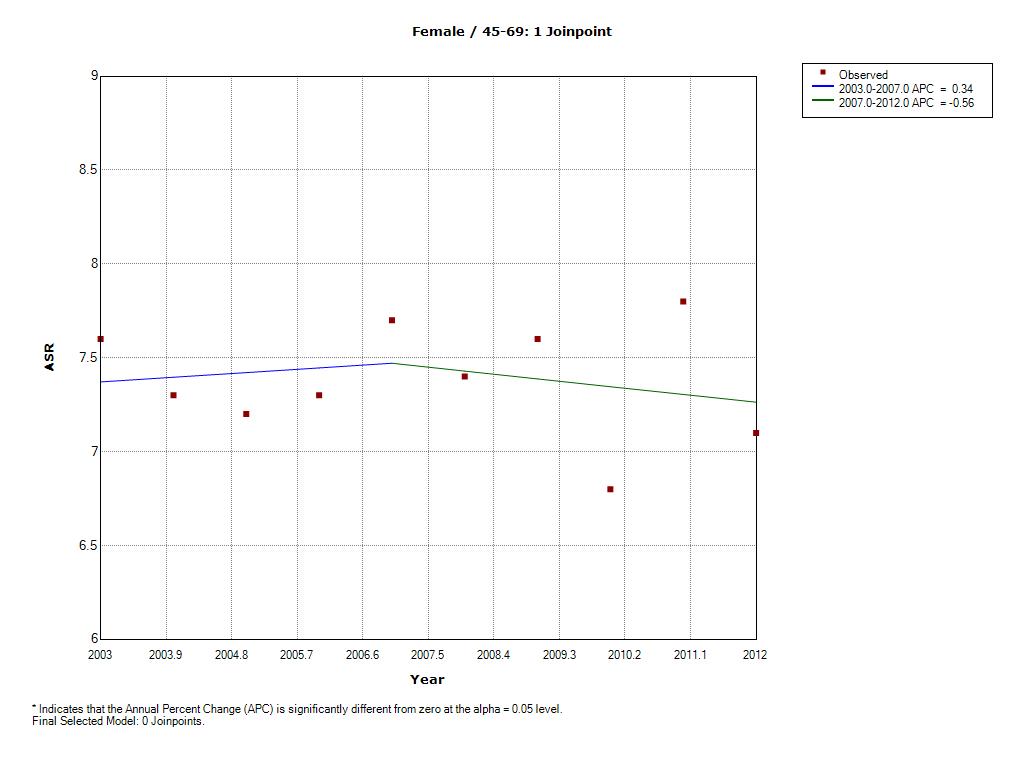

Supplement: Supplementary file 7 — Supplement Figure 7: incidence joinpoint. [file 12889_2024_19104_MOESM7_ESM.zip › Supplement Figure 7 incidence joinpoint/Canada female 45-69.jpg]

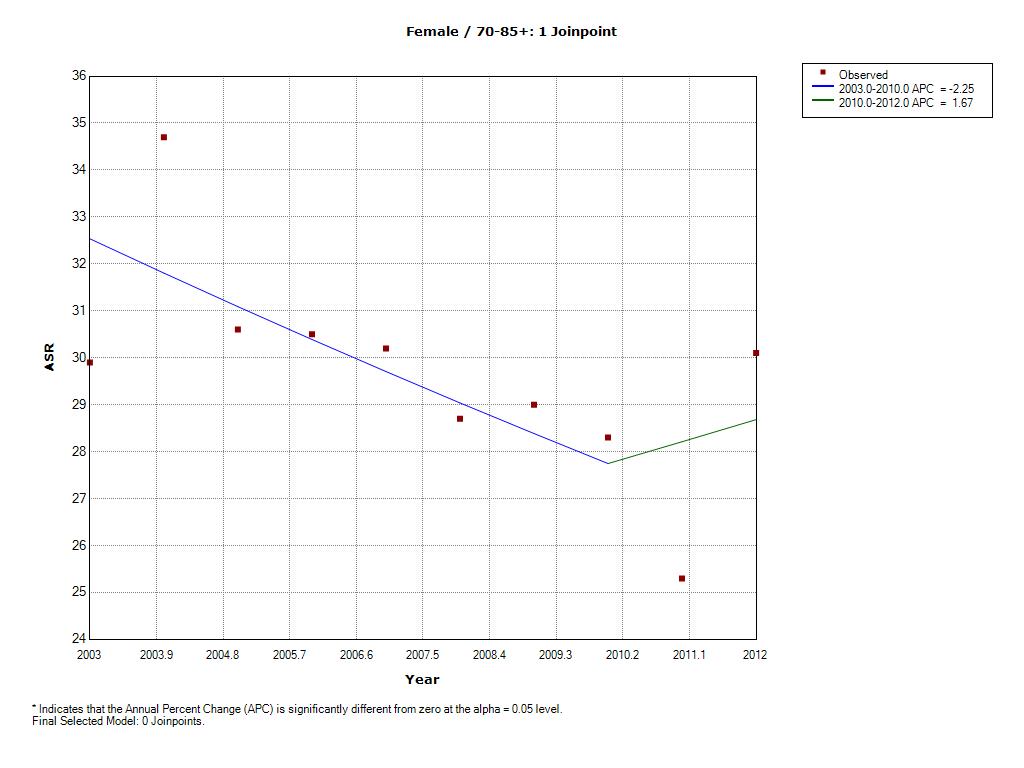

Supplement: Supplementary file 7 — Supplement Figure 7: incidence joinpoint. [file 12889_2024_19104_MOESM7_ESM.zip › Supplement Figure 7 incidence joinpoint/Canada female 70-85+.jpg]

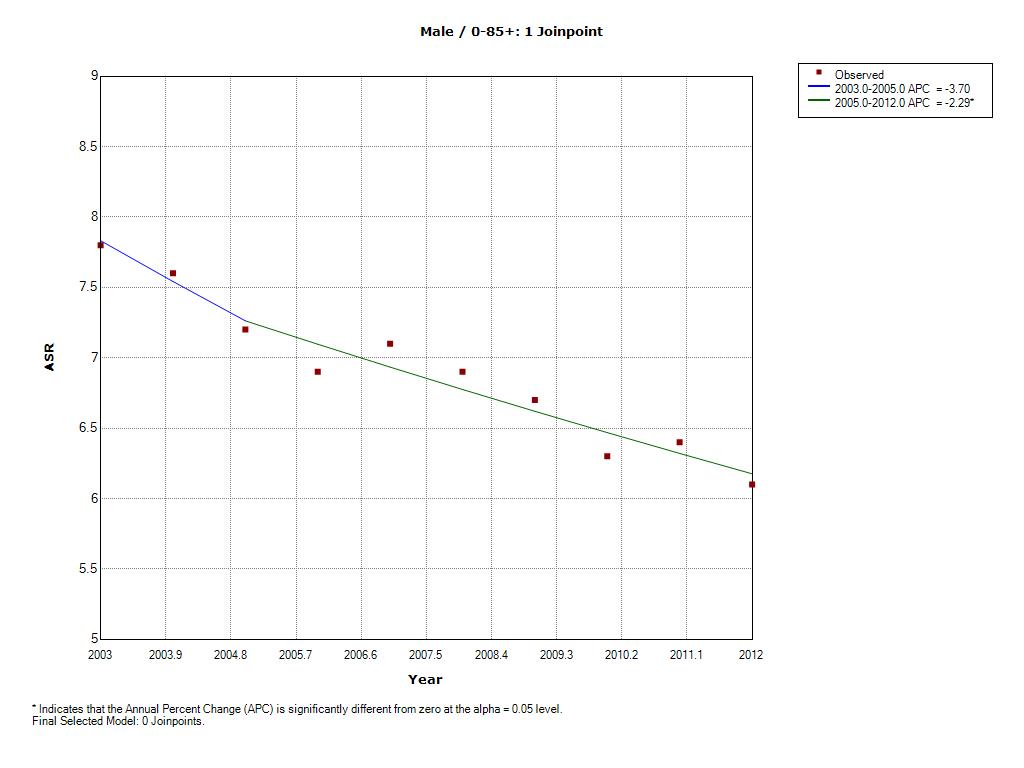

Supplement: Supplementary file 7 — Supplement Figure 7: incidence joinpoint. [file 12889_2024_19104_MOESM7_ESM.zip › Supplement Figure 7 incidence joinpoint/Canada male 0-85+.jpg]

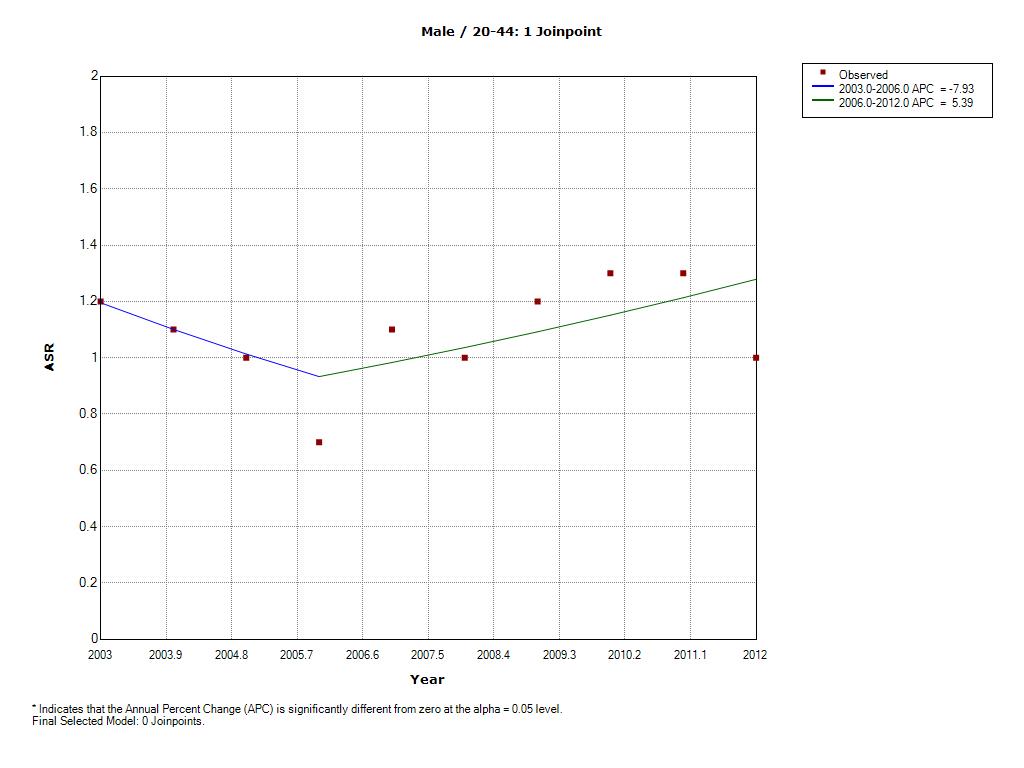

Supplement: Supplementary file 7 — Supplement Figure 7: incidence joinpoint. [file 12889_2024_19104_MOESM7_ESM.zip › Supplement Figure 7 incidence joinpoint/Canada male 20-44.jpg]

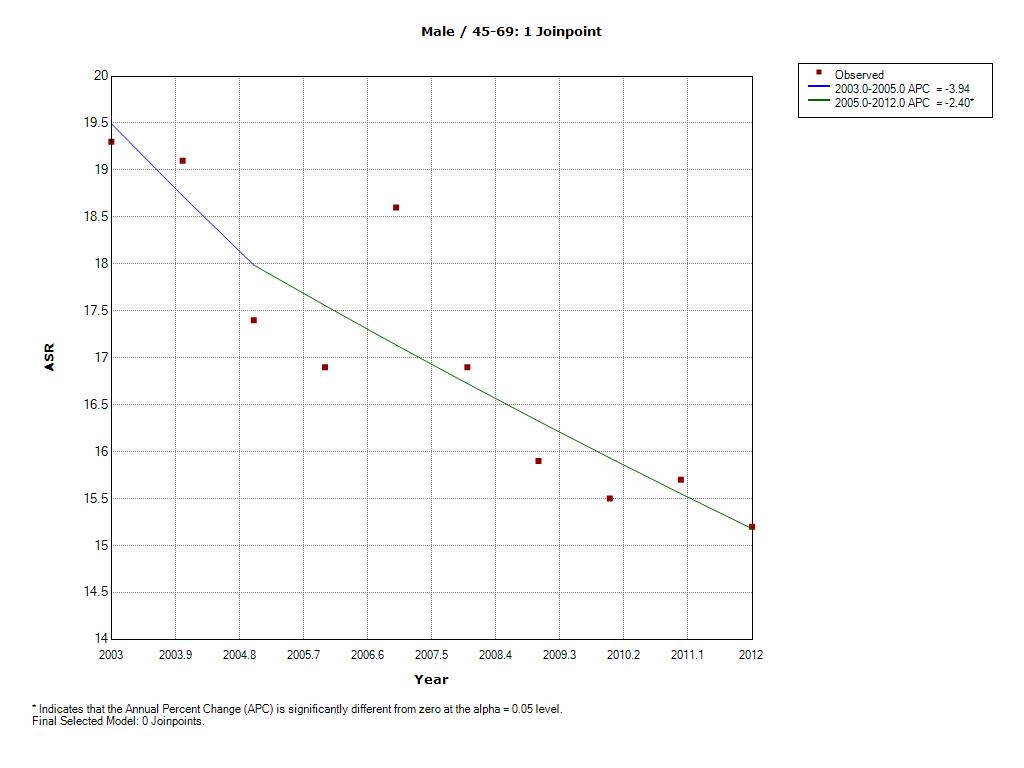

Supplement: Supplementary file 7 — Supplement Figure 7: incidence joinpoint. [file 12889_2024_19104_MOESM7_ESM.zip › Supplement Figure 7 incidence joinpoint/Canada male 45-69.jpg]

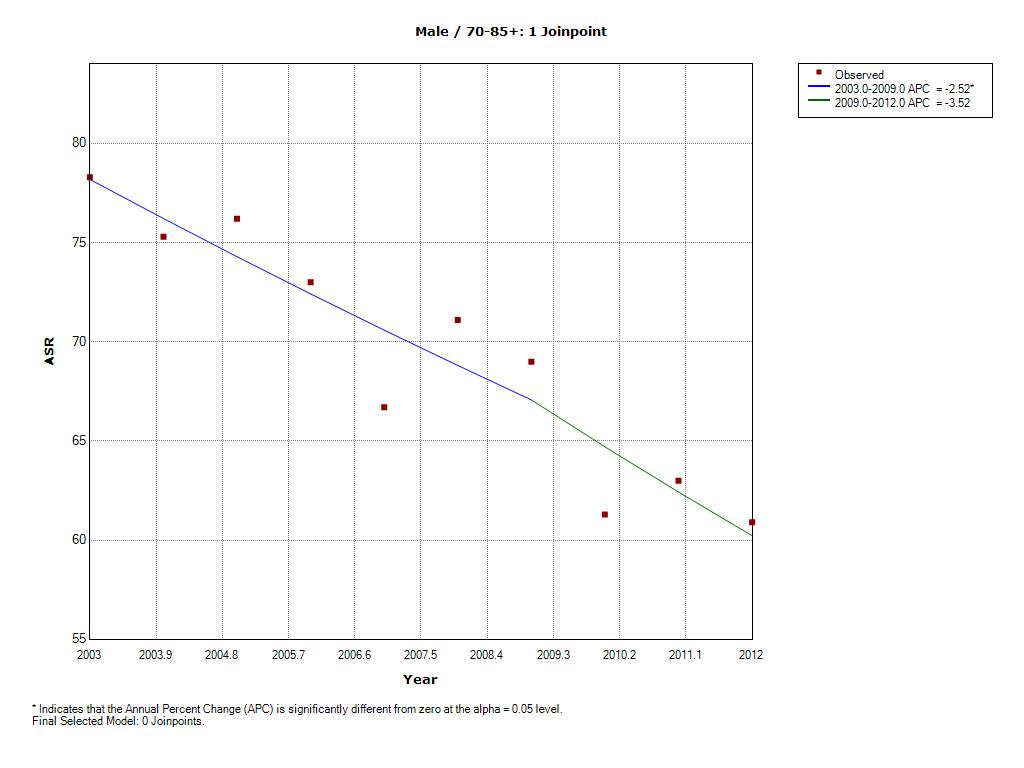

Supplement: Supplementary file 7 — Supplement Figure 7: incidence joinpoint. [file 12889_2024_19104_MOESM7_ESM.zip › Supplement Figure 7 incidence joinpoint/Canada male 70-85+.jpg]

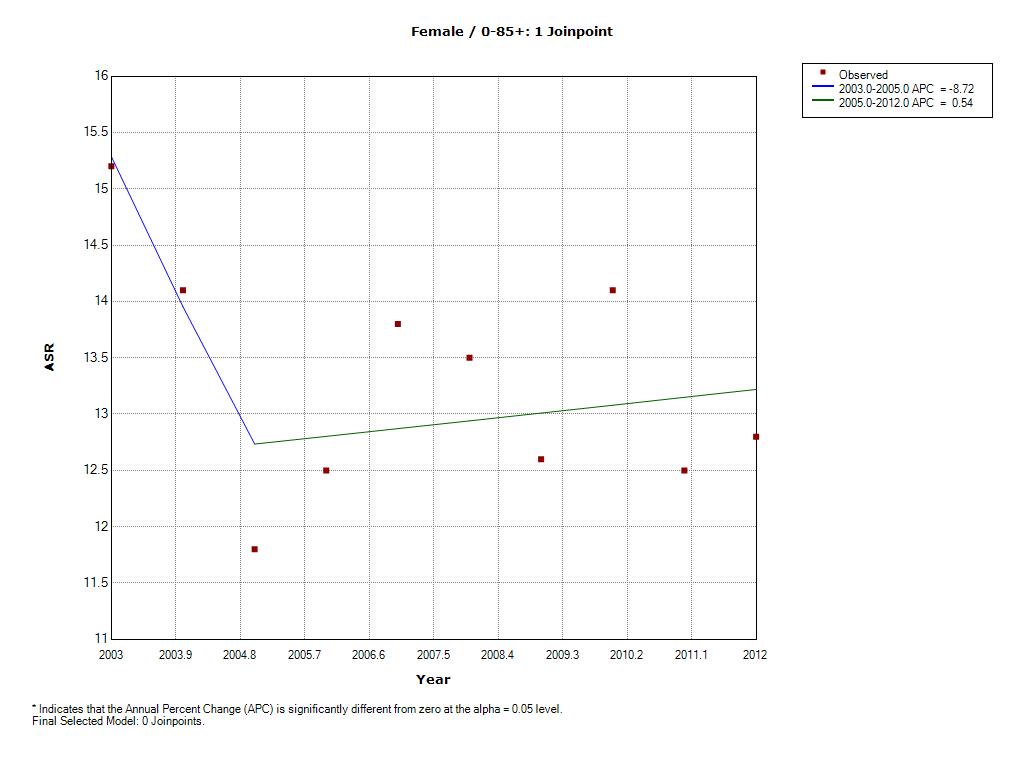

Supplement: Supplementary file 7 — Supplement Figure 7: incidence joinpoint. [file 12889_2024_19104_MOESM7_ESM.zip › Supplement Figure 7 incidence joinpoint/Chile female 0-85+.jpg]

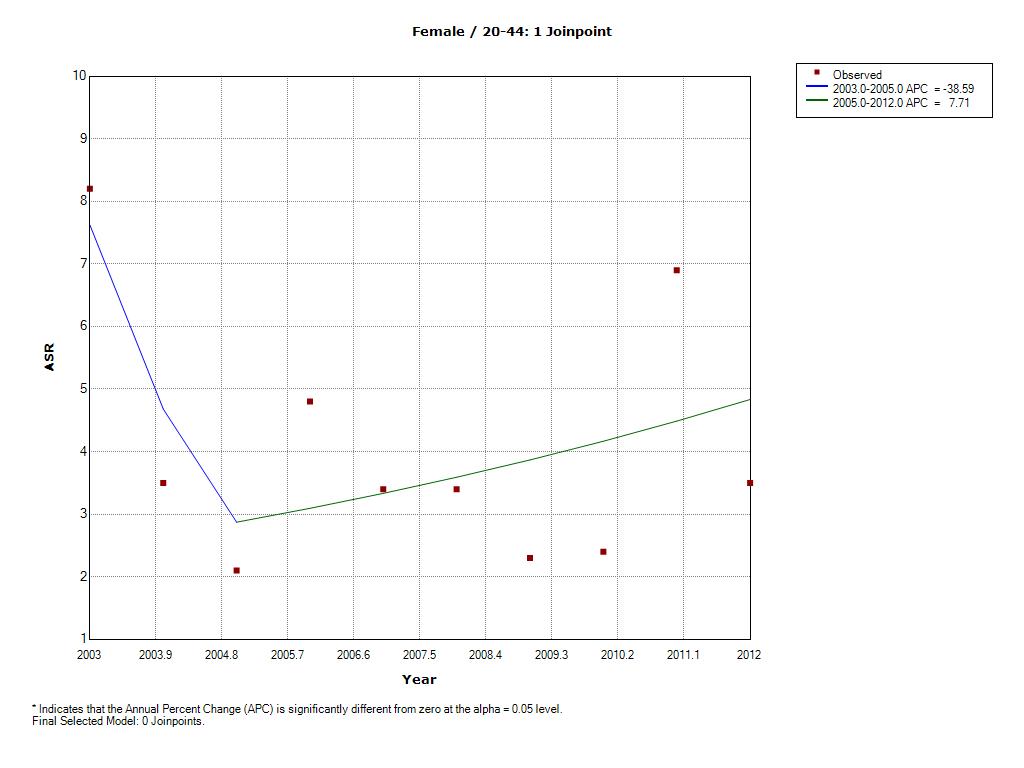

Supplement: Supplementary file 7 — Supplement Figure 7: incidence joinpoint. [file 12889_2024_19104_MOESM7_ESM.zip › Supplement Figure 7 incidence joinpoint/Chile female 20-44.jpg]

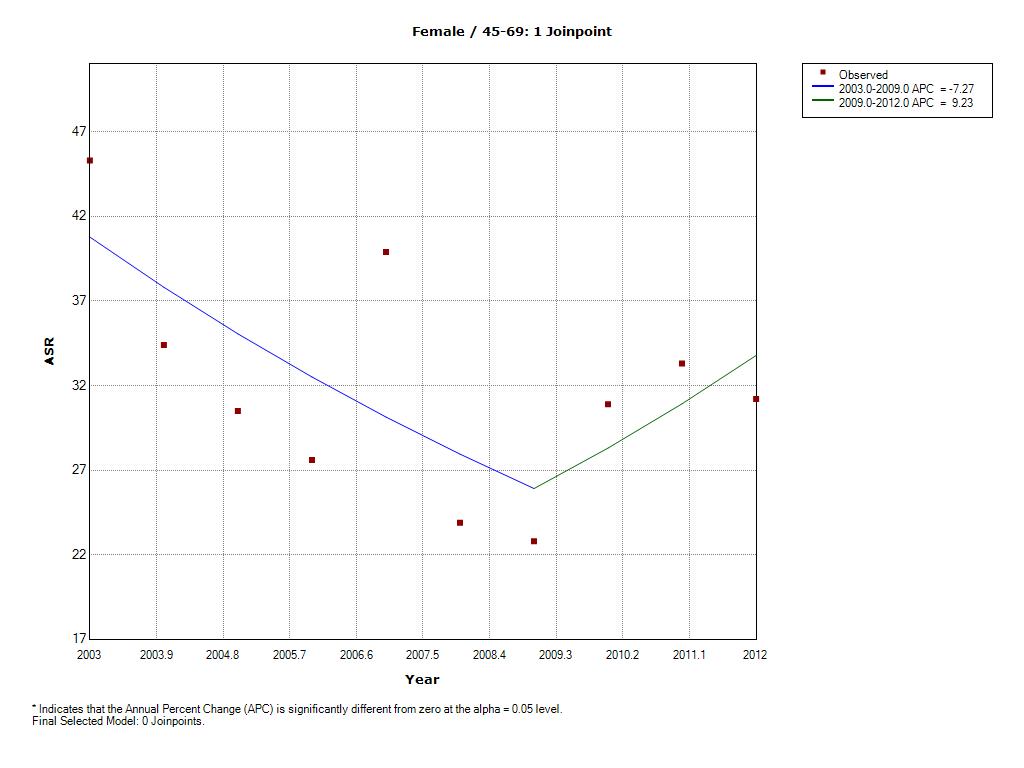

Supplement: Supplementary file 7 — Supplement Figure 7: incidence joinpoint. [file 12889_2024_19104_MOESM7_ESM.zip › Supplement Figure 7 incidence joinpoint/Chile female 45-69.jpg]

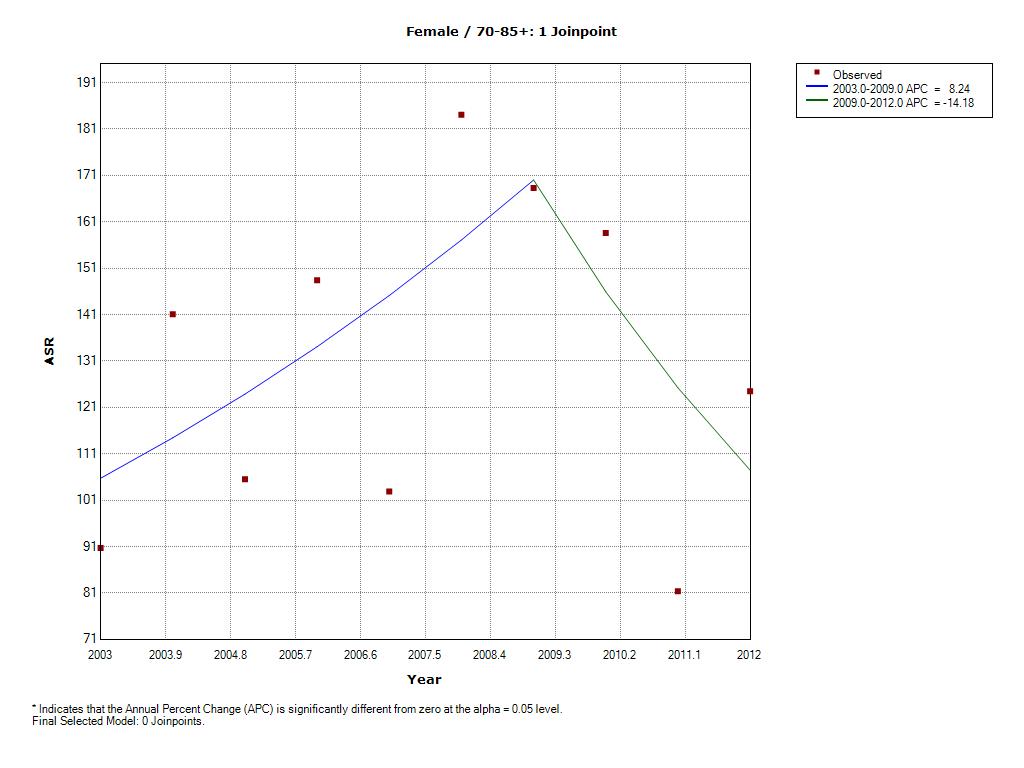

Supplement: Supplementary file 7 — Supplement Figure 7: incidence joinpoint. [file 12889_2024_19104_MOESM7_ESM.zip › Supplement Figure 7 incidence joinpoint/Chile female 70-85+.jpg]

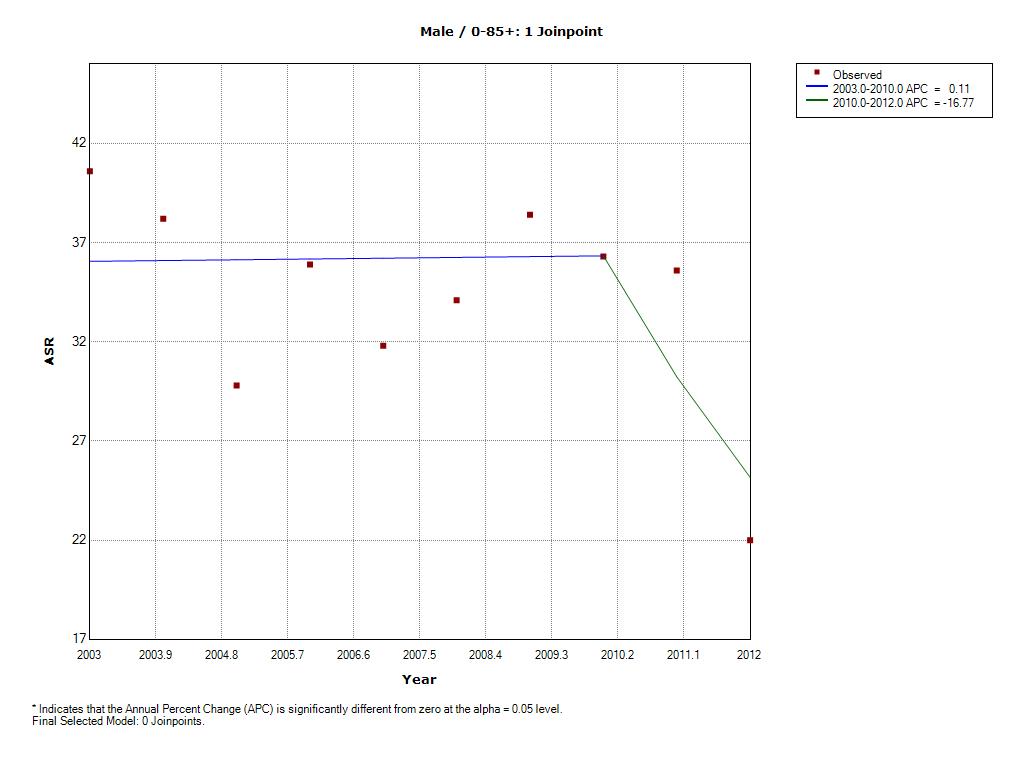

Supplement: Supplementary file 7 — Supplement Figure 7: incidence joinpoint. [file 12889_2024_19104_MOESM7_ESM.zip › Supplement Figure 7 incidence joinpoint/Chile male 0-85+.jpg]

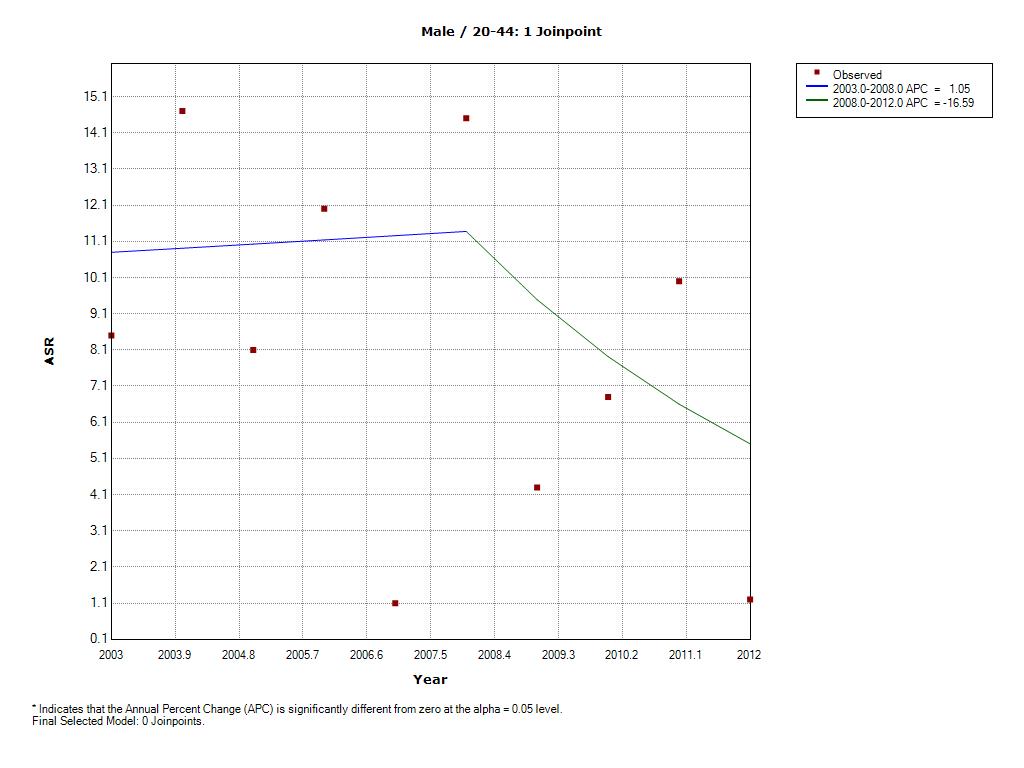

Supplement: Supplementary file 7 — Supplement Figure 7: incidence joinpoint. [file 12889_2024_19104_MOESM7_ESM.zip › Supplement Figure 7 incidence joinpoint/Chile male 20-44.jpg]

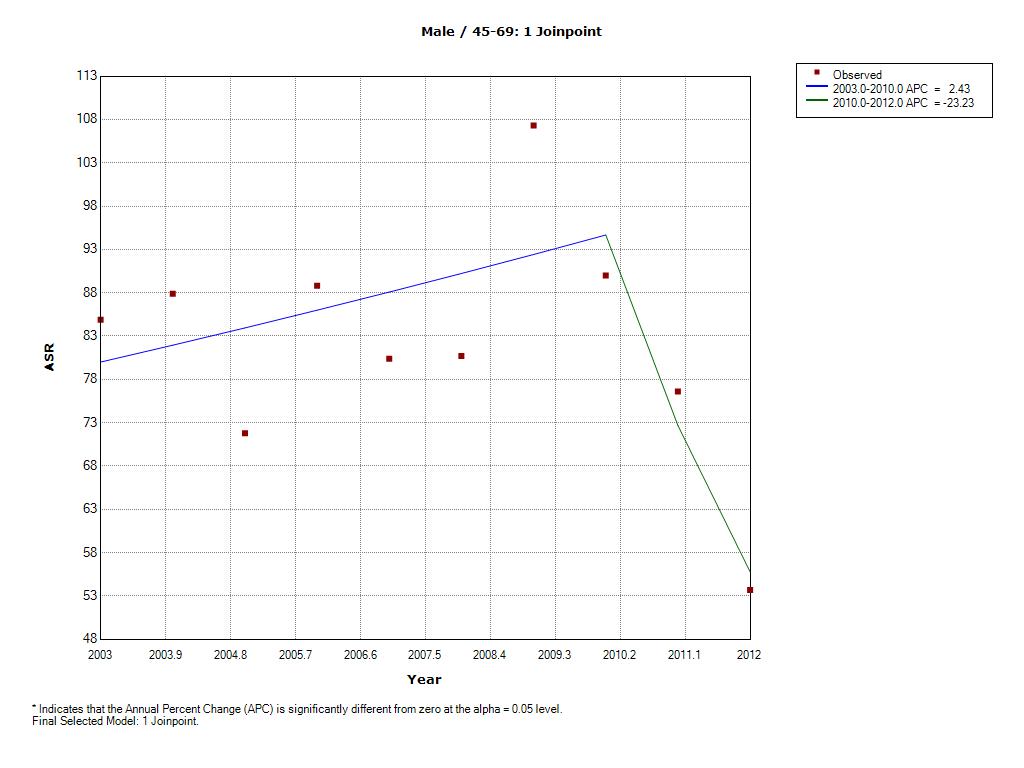

Supplement: Supplementary file 7 — Supplement Figure 7: incidence joinpoint. [file 12889_2024_19104_MOESM7_ESM.zip › Supplement Figure 7 incidence joinpoint/Chile male 45-69.jpg]

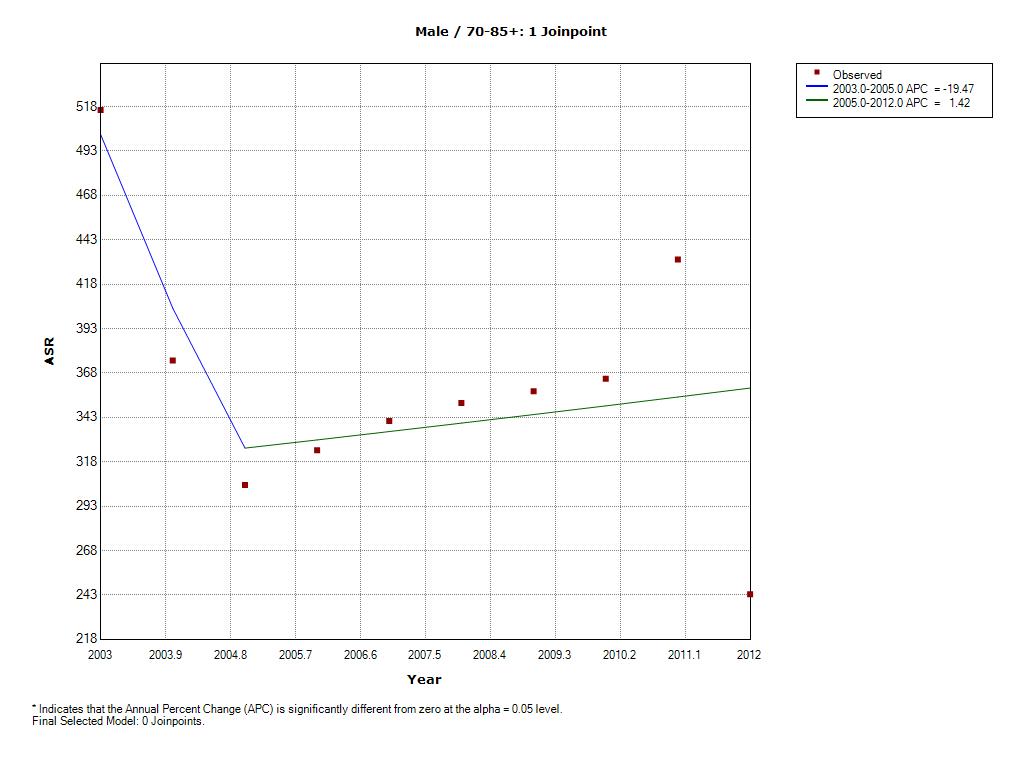

Supplement: Supplementary file 7 — Supplement Figure 7: incidence joinpoint. [file 12889_2024_19104_MOESM7_ESM.zip › Supplement Figure 7 incidence joinpoint/Chile male 70-85+.jpg]

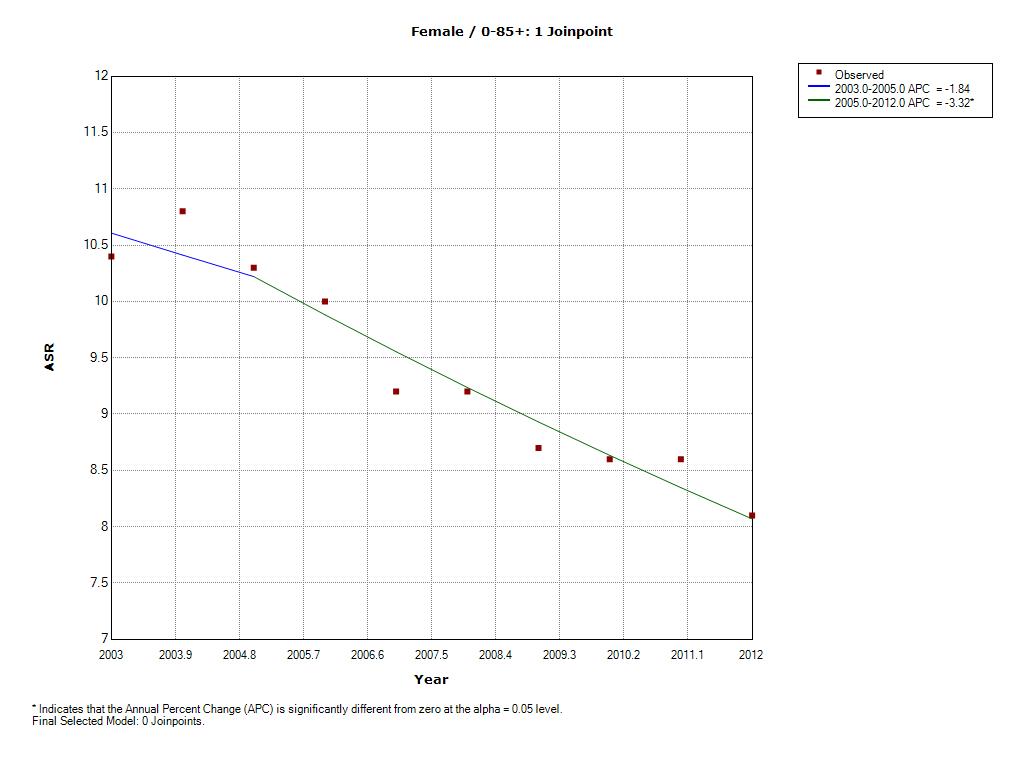

Supplement: Supplementary file 7 — Supplement Figure 7: incidence joinpoint. [file 12889_2024_19104_MOESM7_ESM.zip › Supplement Figure 7 incidence joinpoint/China female 0-85+.jpg]

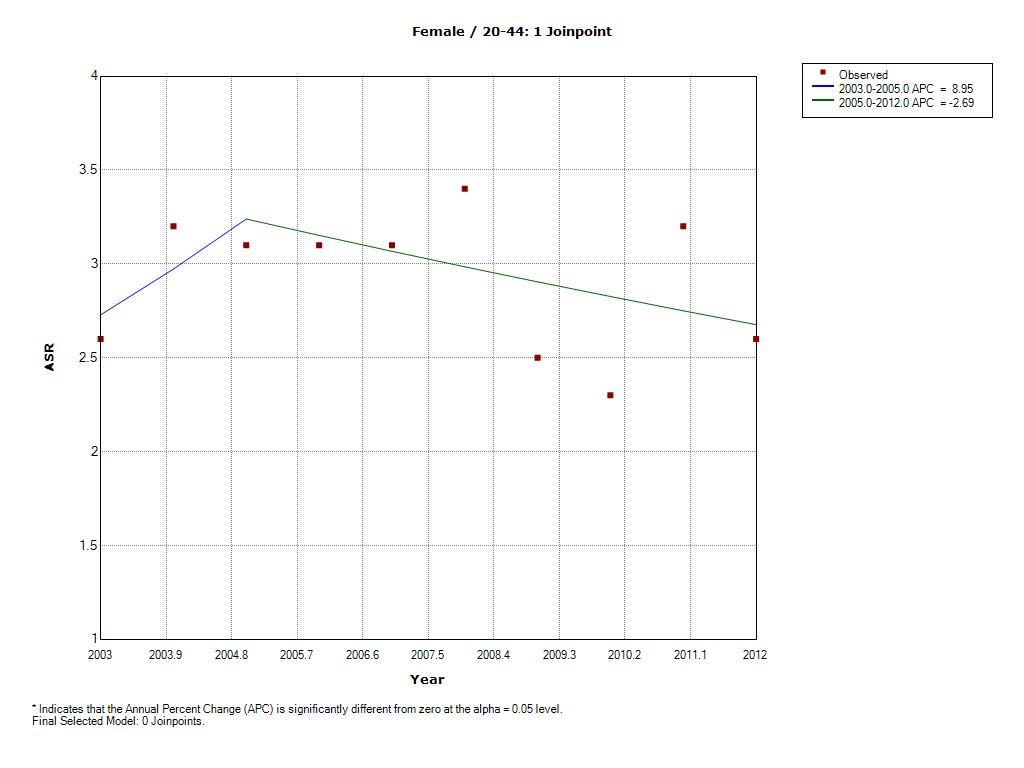

Supplement: Supplementary file 7 — Supplement Figure 7: incidence joinpoint. [file 12889_2024_19104_MOESM7_ESM.zip › Supplement Figure 7 incidence joinpoint/China female 20-44.jpg]

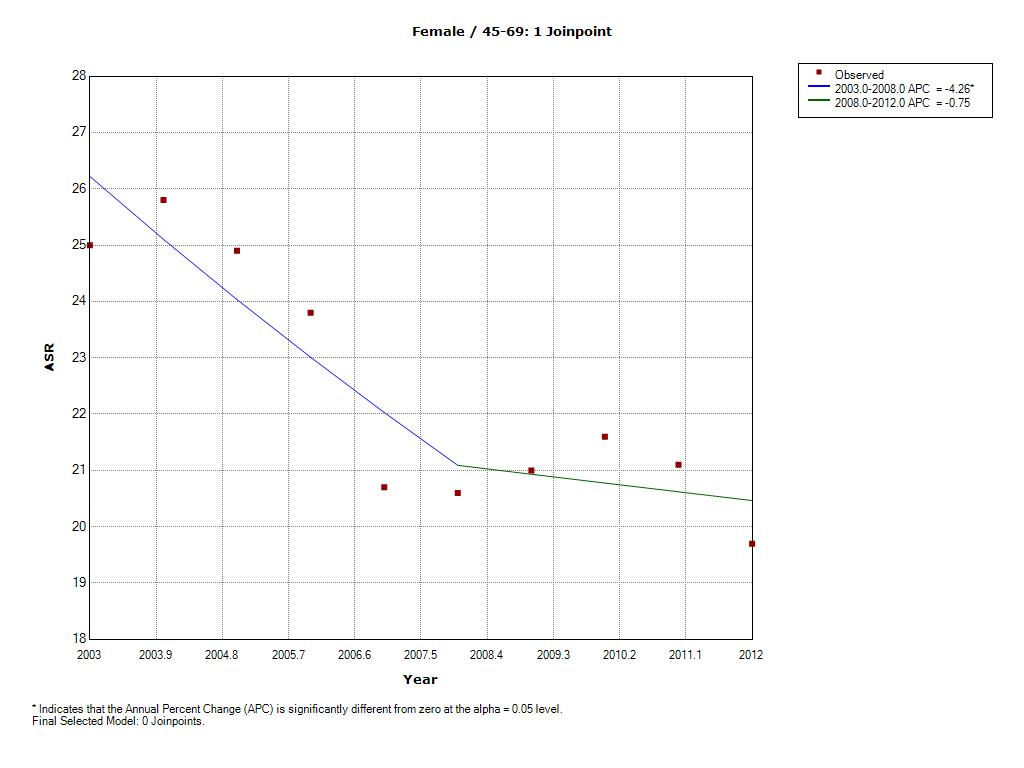

Supplement: Supplementary file 7 — Supplement Figure 7: incidence joinpoint. [file 12889_2024_19104_MOESM7_ESM.zip › Supplement Figure 7 incidence joinpoint/China female 45-69.jpg]

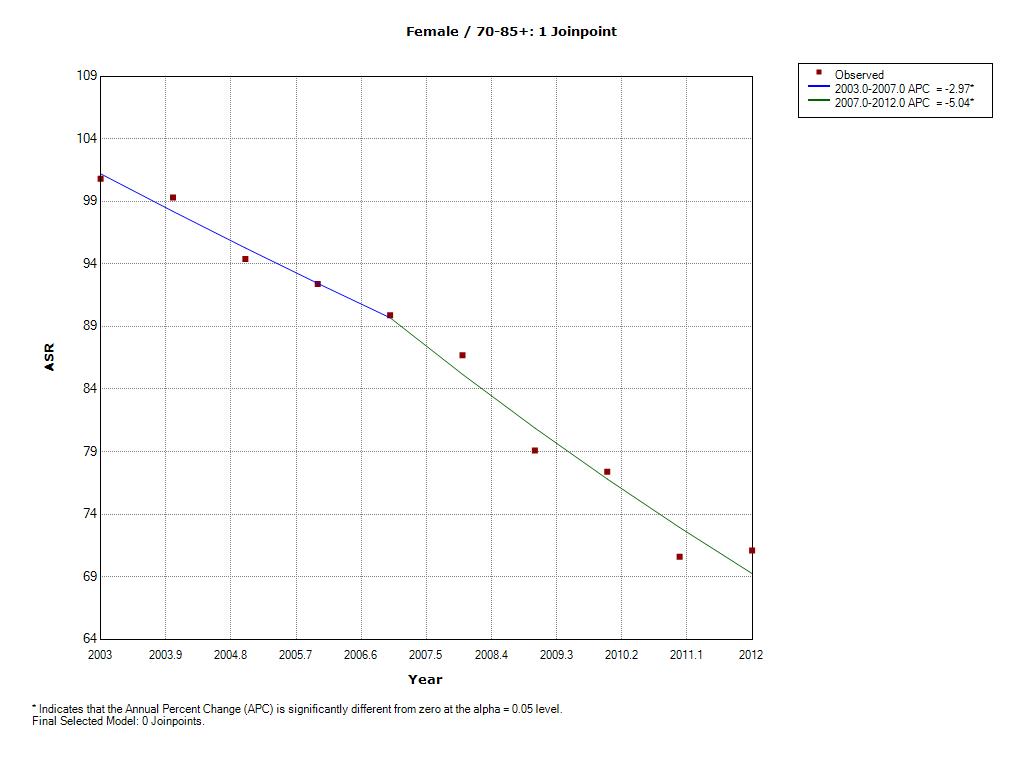

Supplement: Supplementary file 7 — Supplement Figure 7: incidence joinpoint. [file 12889_2024_19104_MOESM7_ESM.zip › Supplement Figure 7 incidence joinpoint/China female 70-85+.jpg]

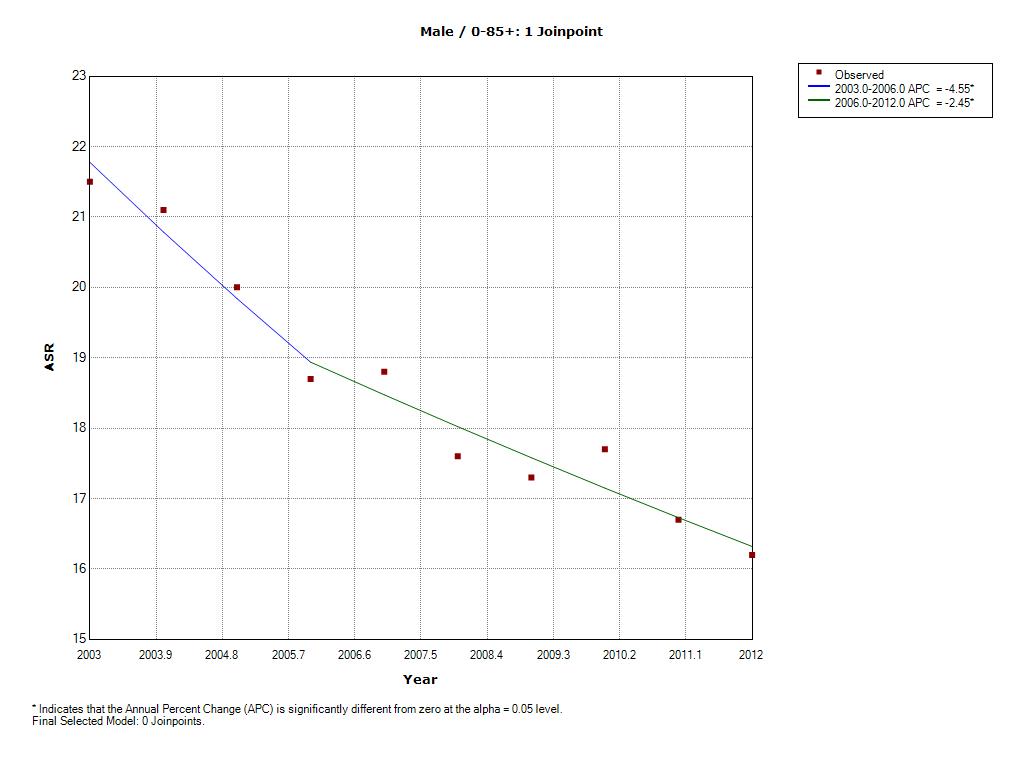

Supplement: Supplementary file 7 — Supplement Figure 7: incidence joinpoint. [file 12889_2024_19104_MOESM7_ESM.zip › Supplement Figure 7 incidence joinpoint/China male 0-85+.jpg]

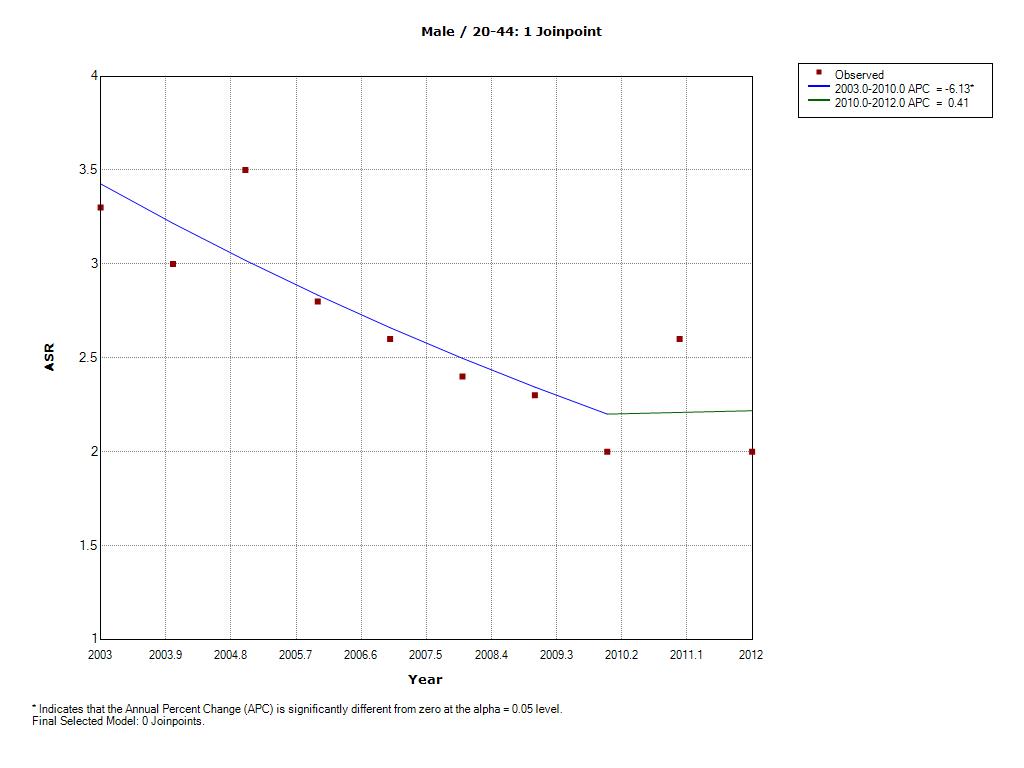

Supplement: Supplementary file 7 — Supplement Figure 7: incidence joinpoint. [file 12889_2024_19104_MOESM7_ESM.zip › Supplement Figure 7 incidence joinpoint/China male 20-44.jpg]

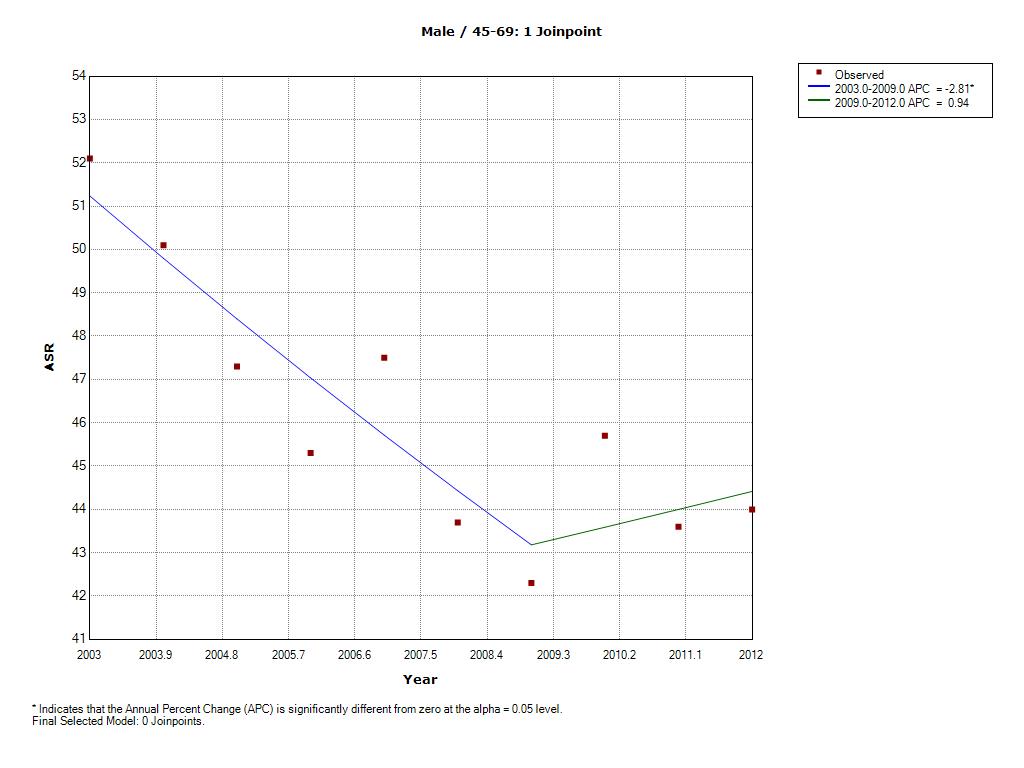

Supplement: Supplementary file 7 — Supplement Figure 7: incidence joinpoint. [file 12889_2024_19104_MOESM7_ESM.zip › Supplement Figure 7 incidence joinpoint/China male 45-69.jpg]

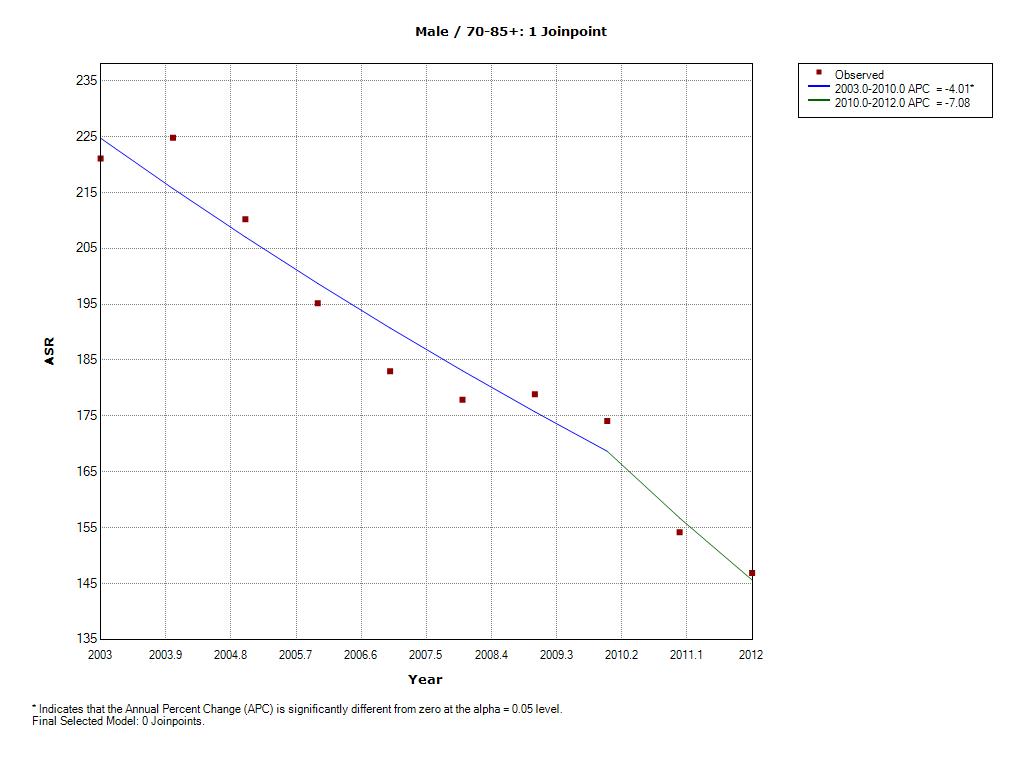

Supplement: Supplementary file 7 — Supplement Figure 7: incidence joinpoint. [file 12889_2024_19104_MOESM7_ESM.zip › Supplement Figure 7 incidence joinpoint/China male 70-85+.jpg]

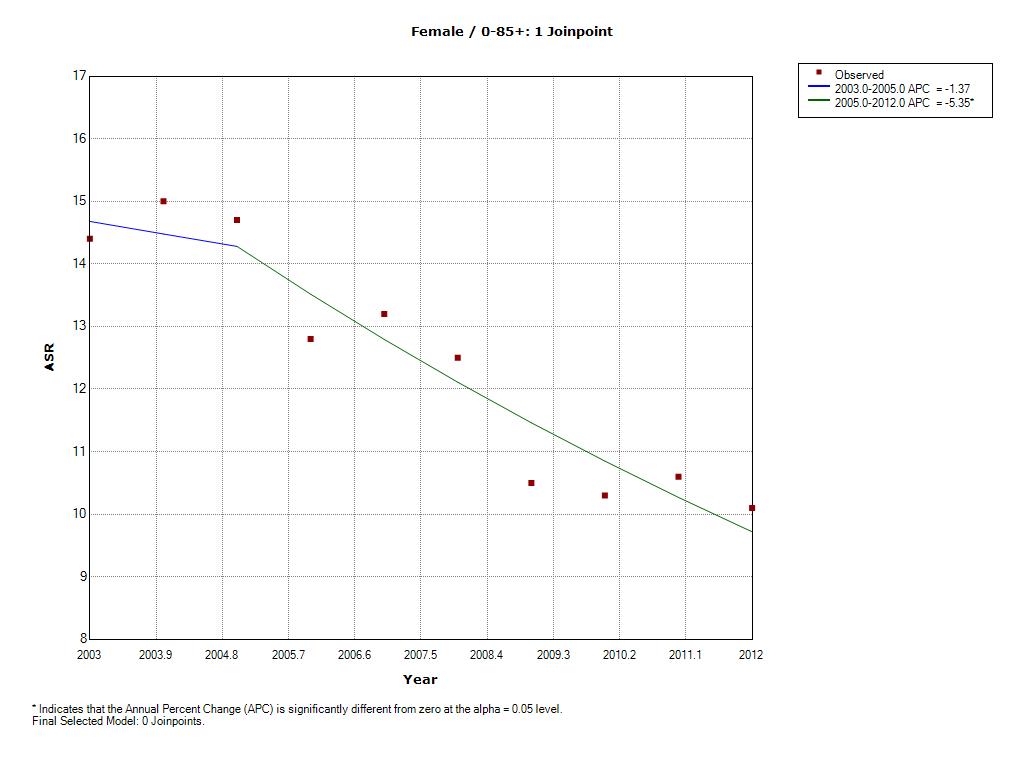

Supplement: Supplementary file 7 — Supplement Figure 7: incidence joinpoint. [file 12889_2024_19104_MOESM7_ESM.zip › Supplement Figure 7 incidence joinpoint/Colombia female 0-85+.jpg]

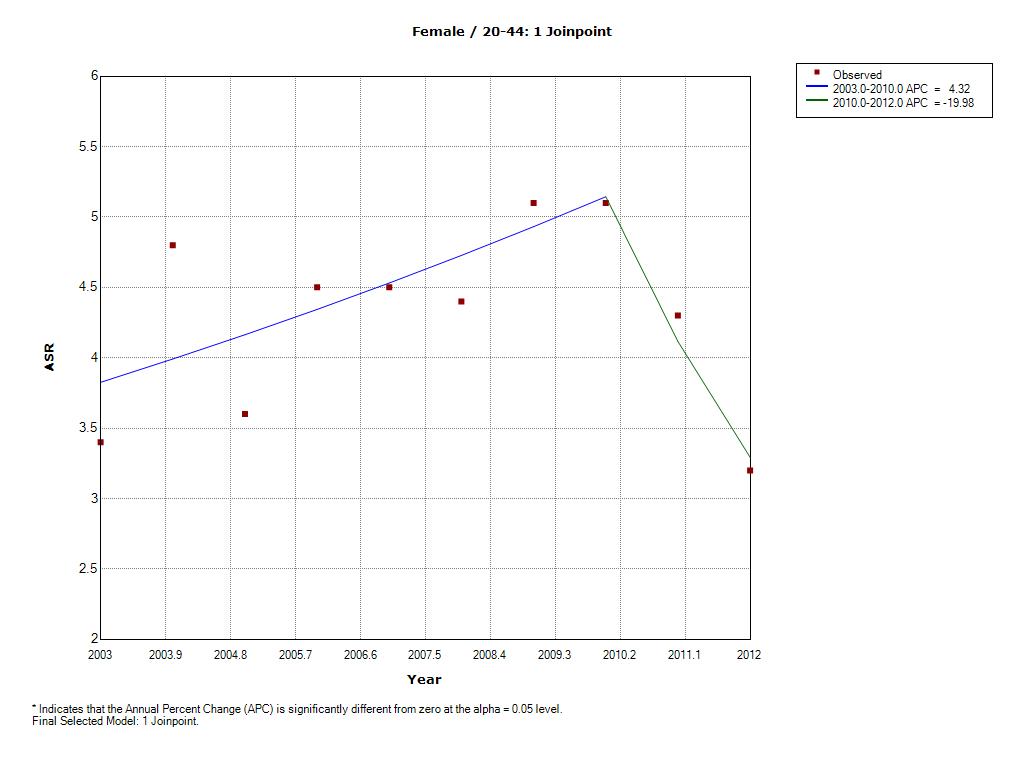

Supplement: Supplementary file 7 — Supplement Figure 7: incidence joinpoint. [file 12889_2024_19104_MOESM7_ESM.zip › Supplement Figure 7 incidence joinpoint/Colombia female 20-44.jpg]

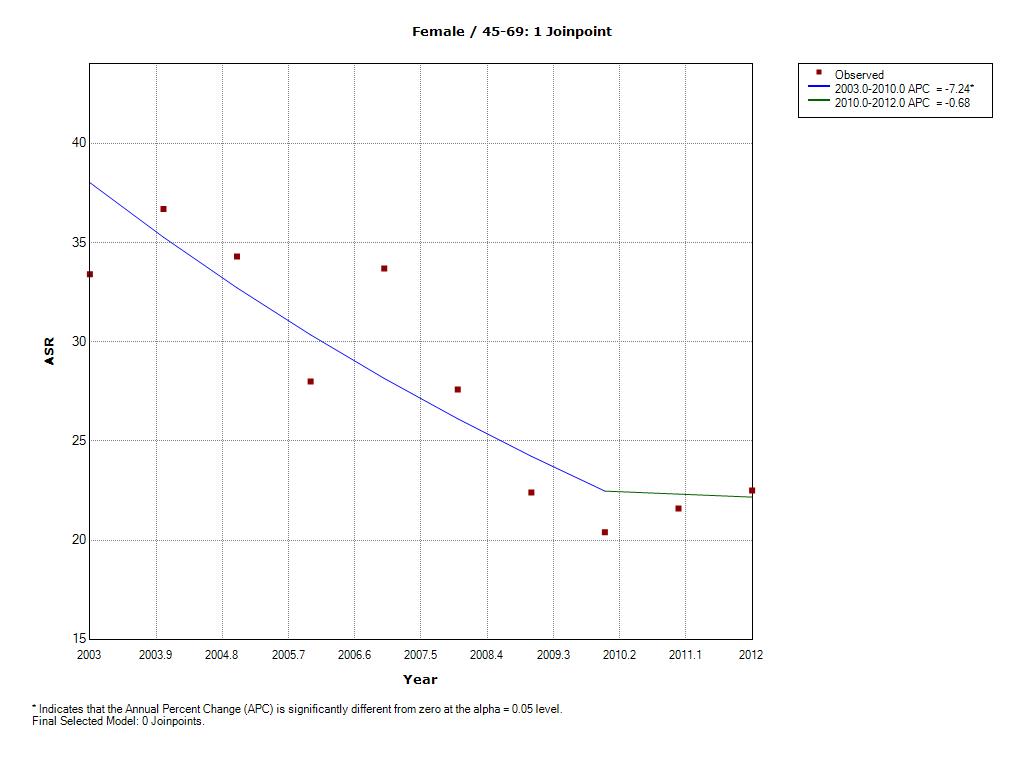

Supplement: Supplementary file 7 — Supplement Figure 7: incidence joinpoint. [file 12889_2024_19104_MOESM7_ESM.zip › Supplement Figure 7 incidence joinpoint/Colombia female 45-69.jpg]

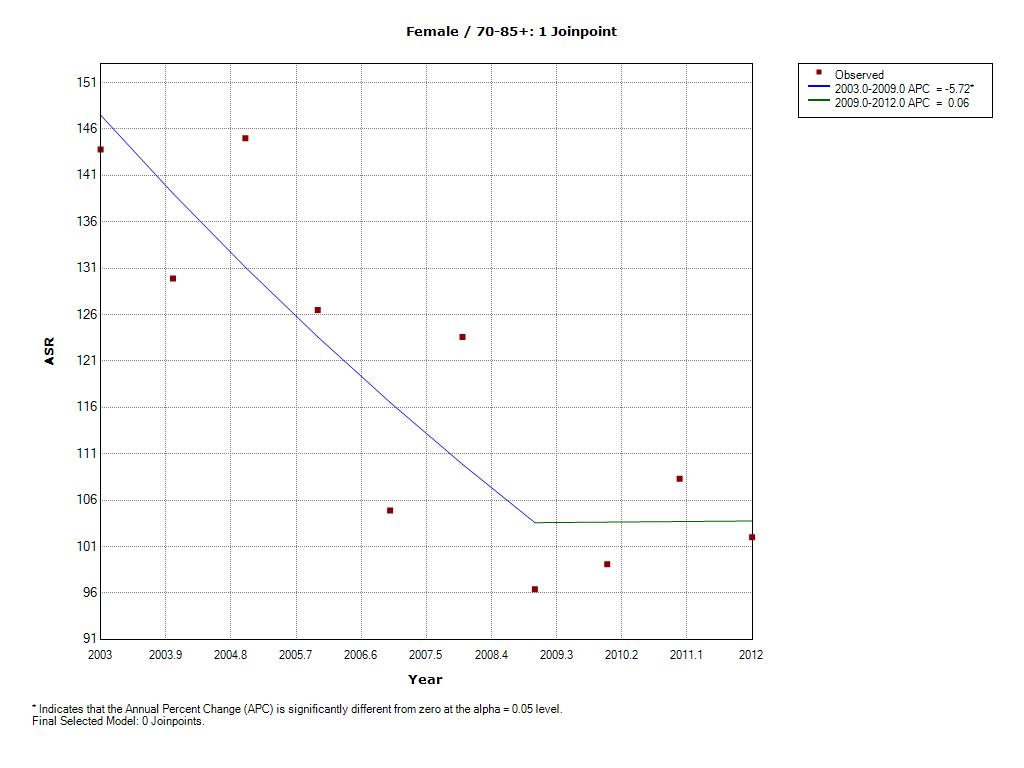

Supplement: Supplementary file 7 — Supplement Figure 7: incidence joinpoint. [file 12889_2024_19104_MOESM7_ESM.zip › Supplement Figure 7 incidence joinpoint/Colombia female 70-85+.jpg]

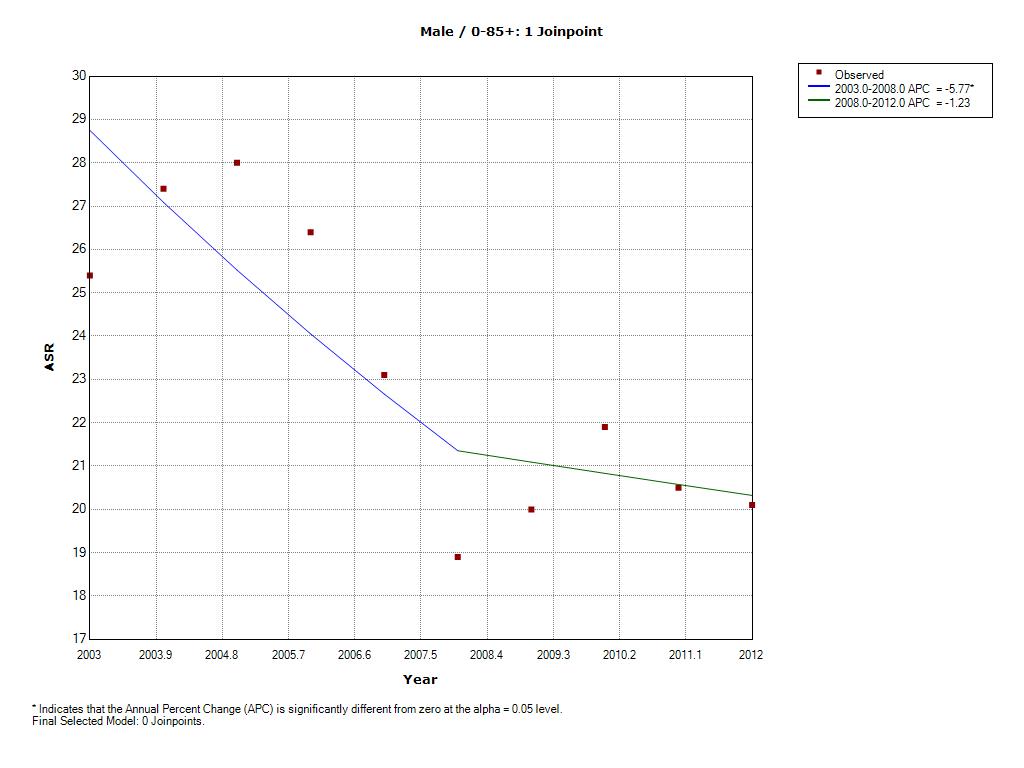

Supplement: Supplementary file 7 — Supplement Figure 7: incidence joinpoint. [file 12889_2024_19104_MOESM7_ESM.zip › Supplement Figure 7 incidence joinpoint/Colombia male 0-85+.jpg]

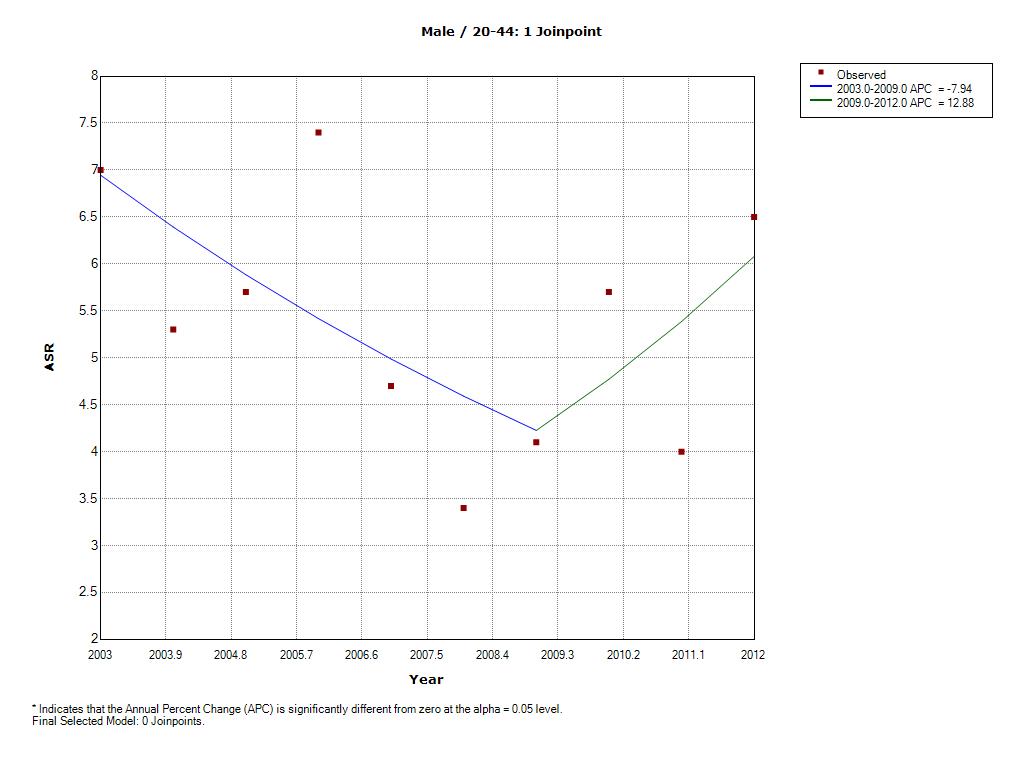

Supplement: Supplementary file 7 — Supplement Figure 7: incidence joinpoint. [file 12889_2024_19104_MOESM7_ESM.zip › Supplement Figure 7 incidence joinpoint/Colombia male 20-44.jpg]

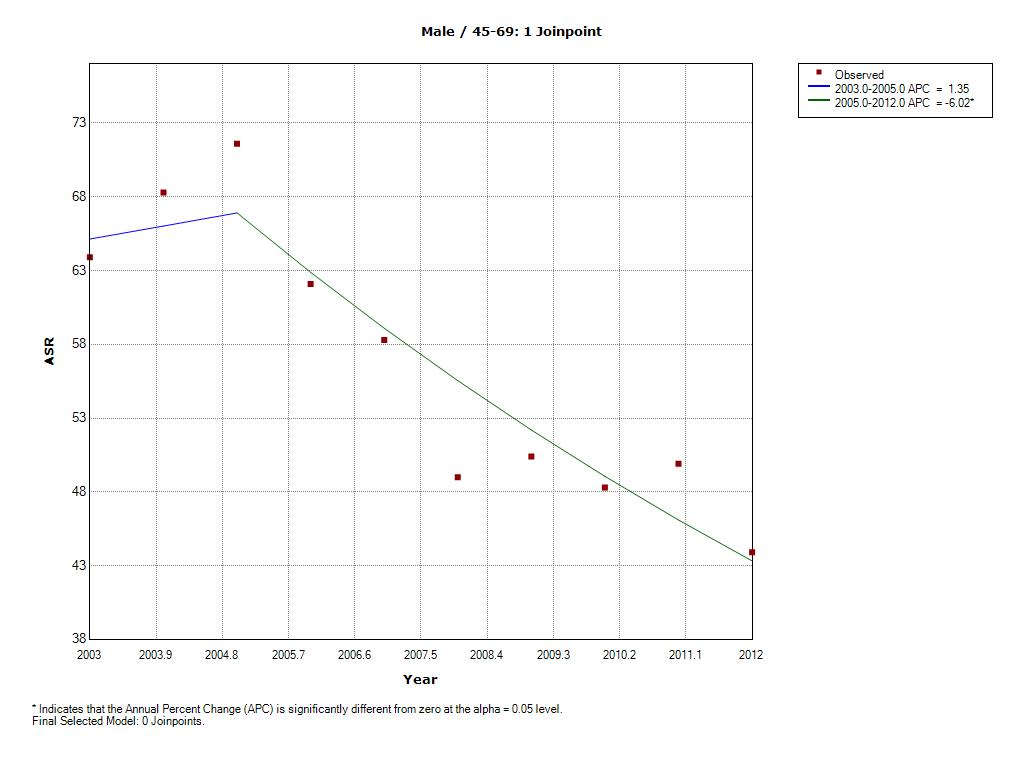

Supplement: Supplementary file 7 — Supplement Figure 7: incidence joinpoint. [file 12889_2024_19104_MOESM7_ESM.zip › Supplement Figure 7 incidence joinpoint/Colombia male 45-69.jpg]

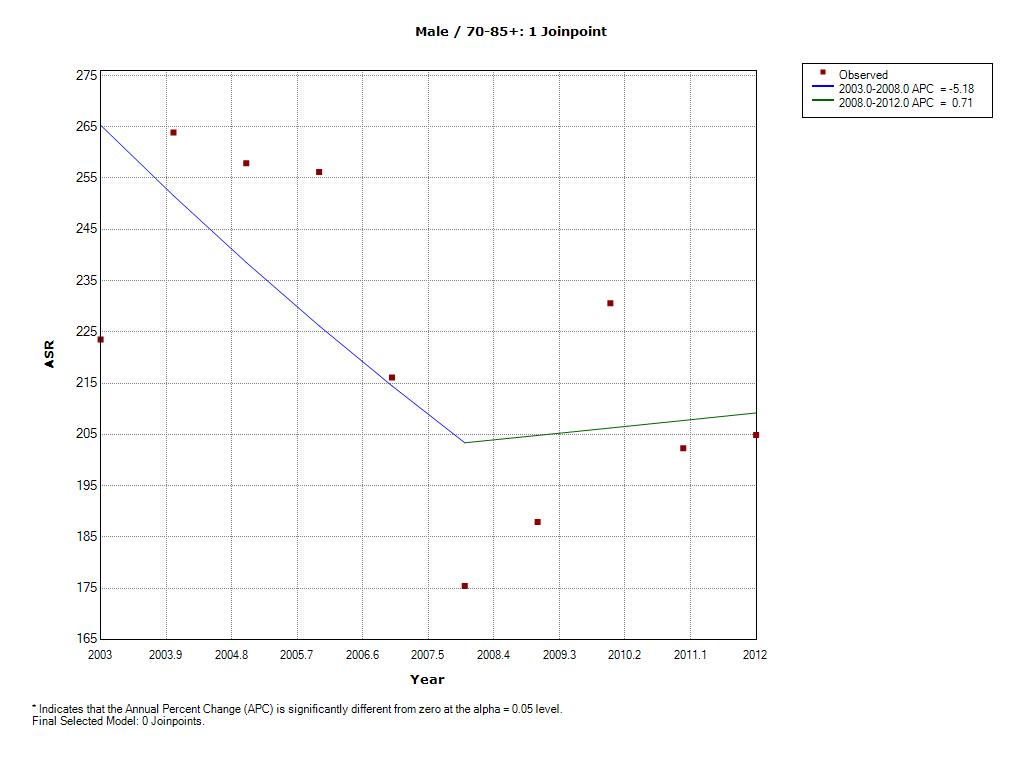

Supplement: Supplementary file 7 — Supplement Figure 7: incidence joinpoint. [file 12889_2024_19104_MOESM7_ESM.zip › Supplement Figure 7 incidence joinpoint/Colombia male 70-85+.jpg]

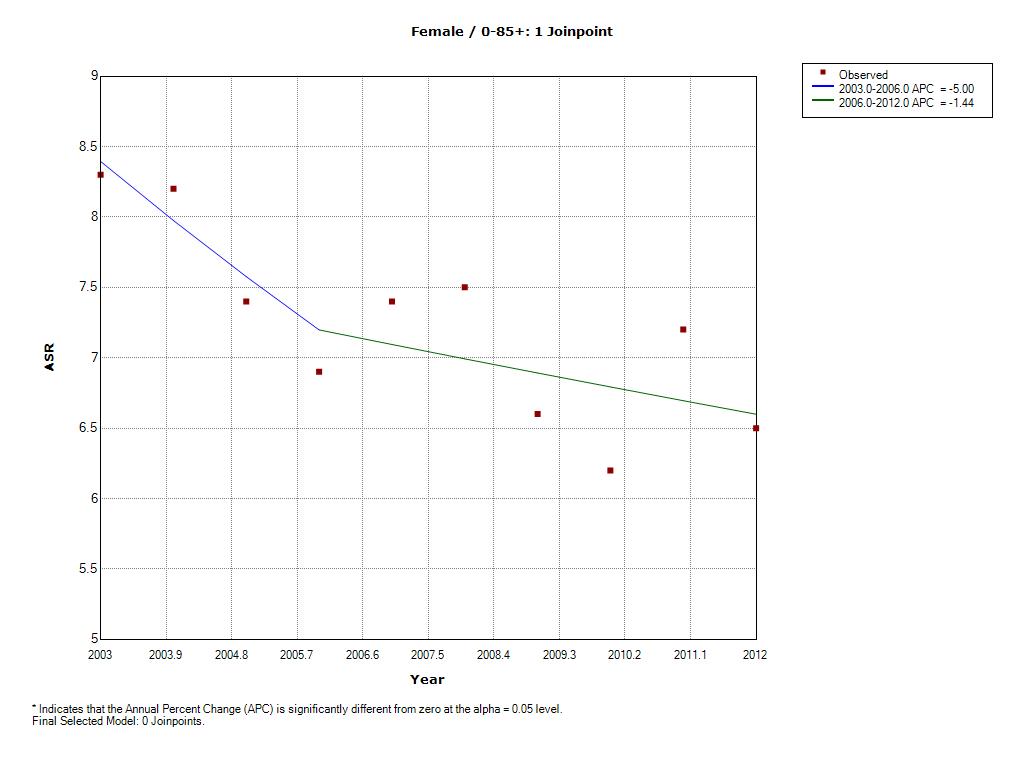

Supplement: Supplementary file 7 — Supplement Figure 7: incidence joinpoint. [file 12889_2024_19104_MOESM7_ESM.zip › Supplement Figure 7 incidence joinpoint/Croatia female 0-85+.jpg]

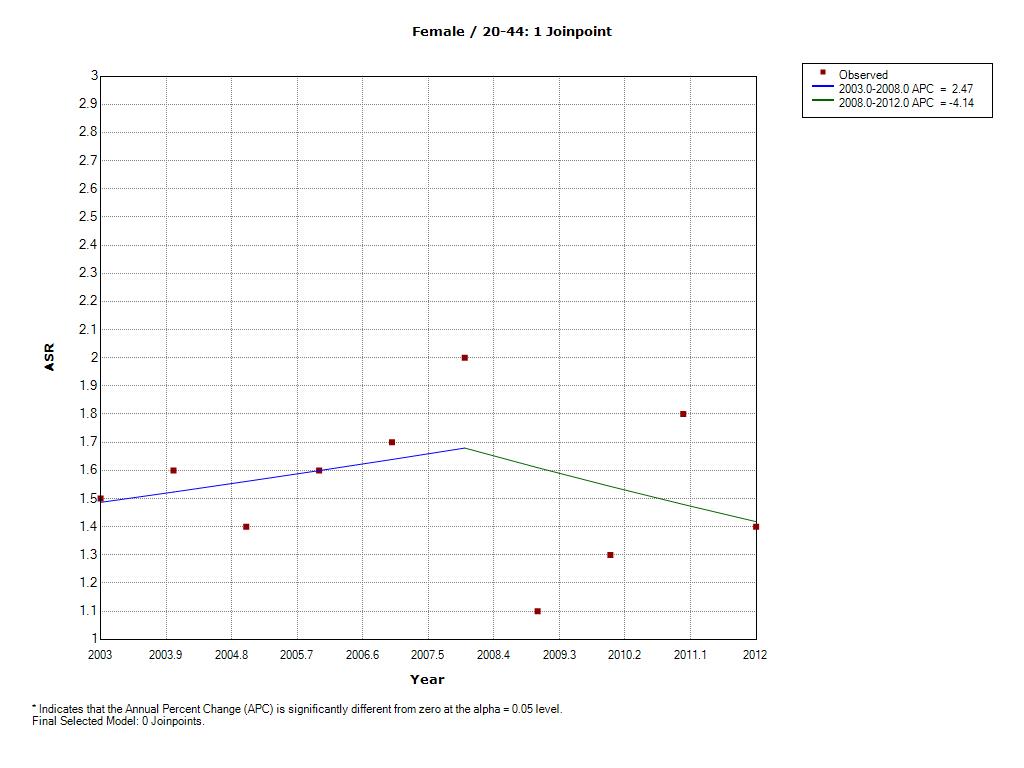

Supplement: Supplementary file 7 — Supplement Figure 7: incidence joinpoint. [file 12889_2024_19104_MOESM7_ESM.zip › Supplement Figure 7 incidence joinpoint/Croatia female 20-44.jpg]

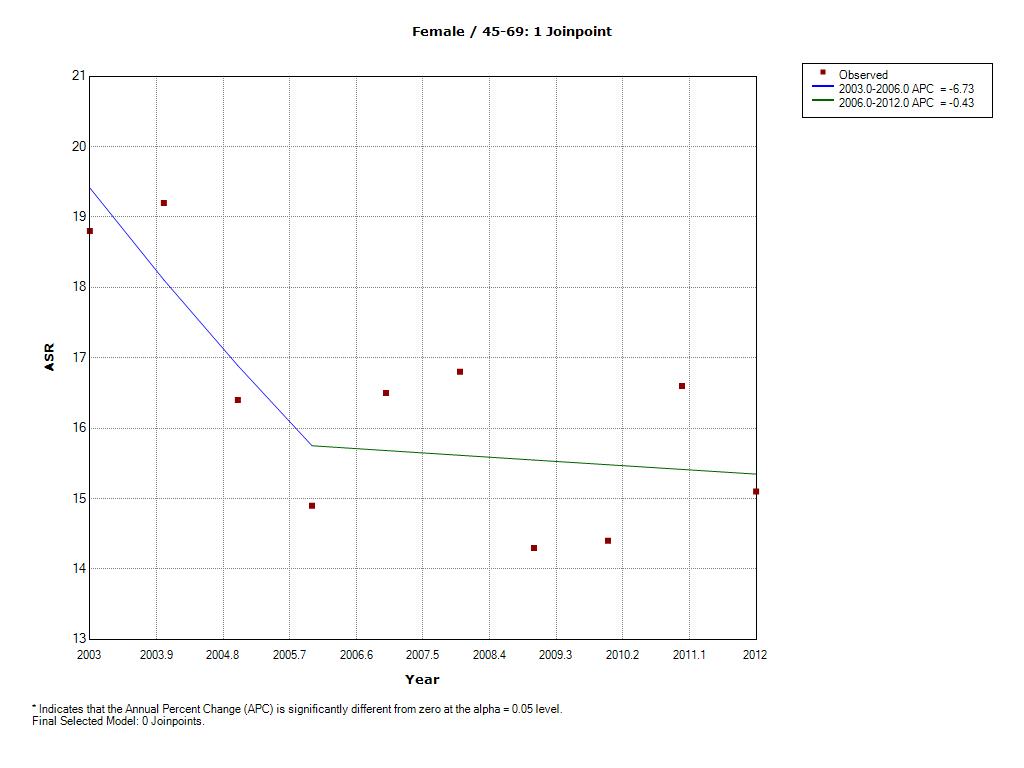

Supplement: Supplementary file 7 — Supplement Figure 7: incidence joinpoint. [file 12889_2024_19104_MOESM7_ESM.zip › Supplement Figure 7 incidence joinpoint/Croatia female 45-69.jpg]

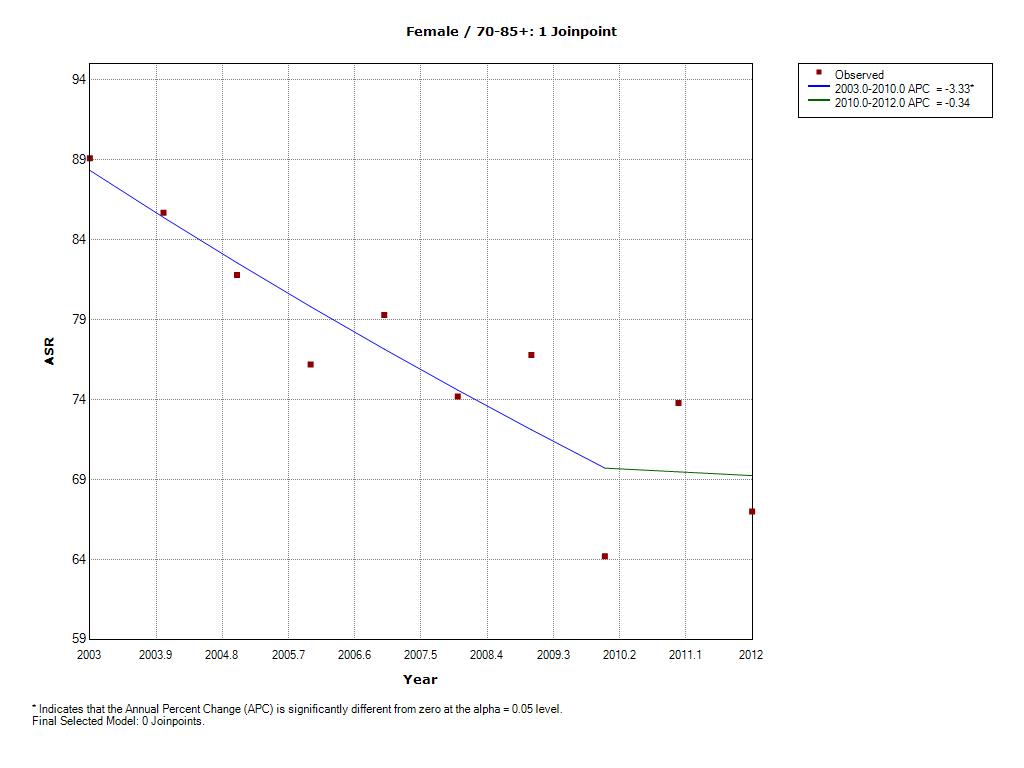

Supplement: Supplementary file 7 — Supplement Figure 7: incidence joinpoint. [file 12889_2024_19104_MOESM7_ESM.zip › Supplement Figure 7 incidence joinpoint/Croatia female 70-85+.jpg]

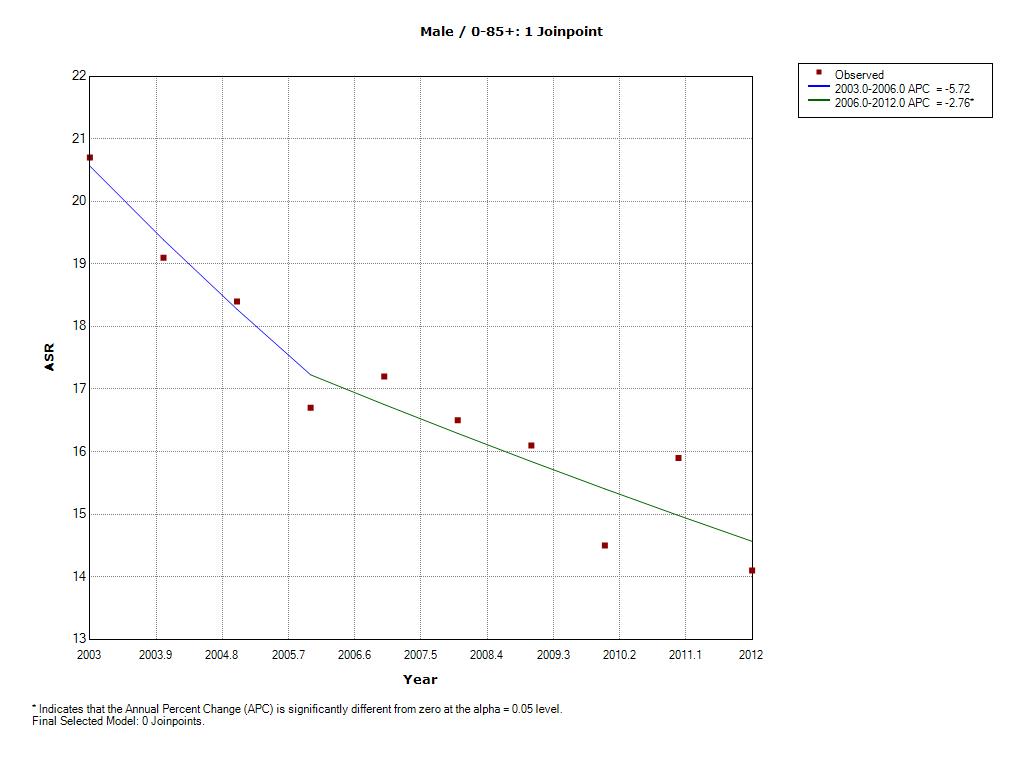

Supplement: Supplementary file 7 — Supplement Figure 7: incidence joinpoint. [file 12889_2024_19104_MOESM7_ESM.zip › Supplement Figure 7 incidence joinpoint/Croatia male 0-85+.jpg]

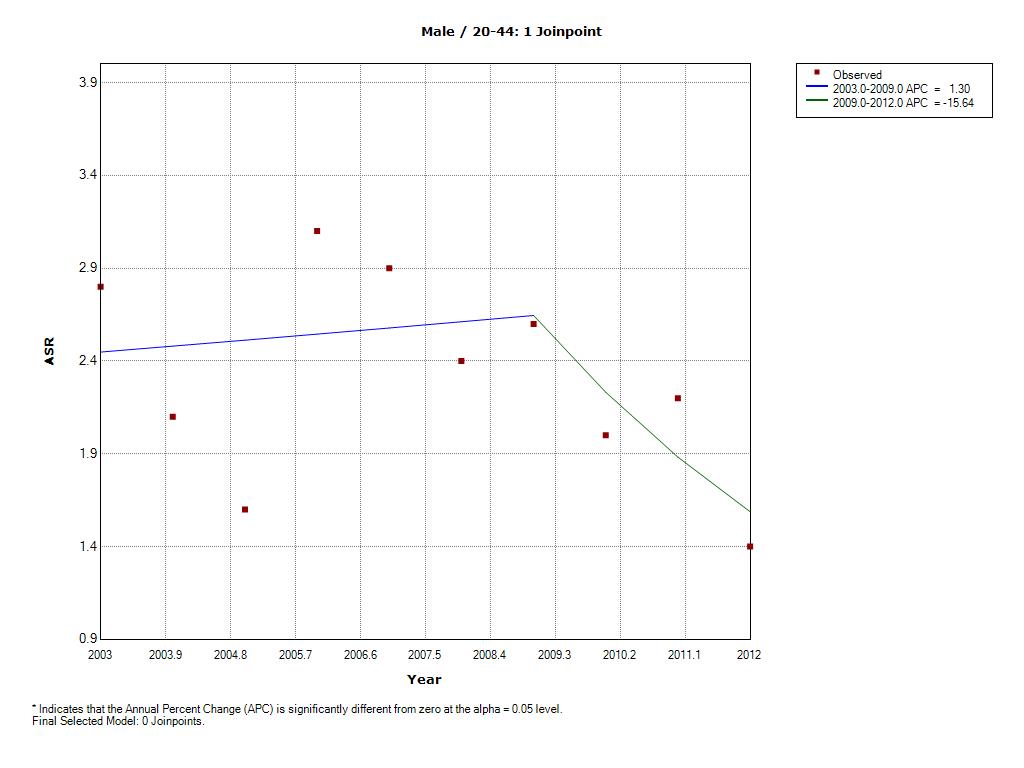

Supplement: Supplementary file 7 — Supplement Figure 7: incidence joinpoint. [file 12889_2024_19104_MOESM7_ESM.zip › Supplement Figure 7 incidence joinpoint/Croatia male 20-44.jpg]

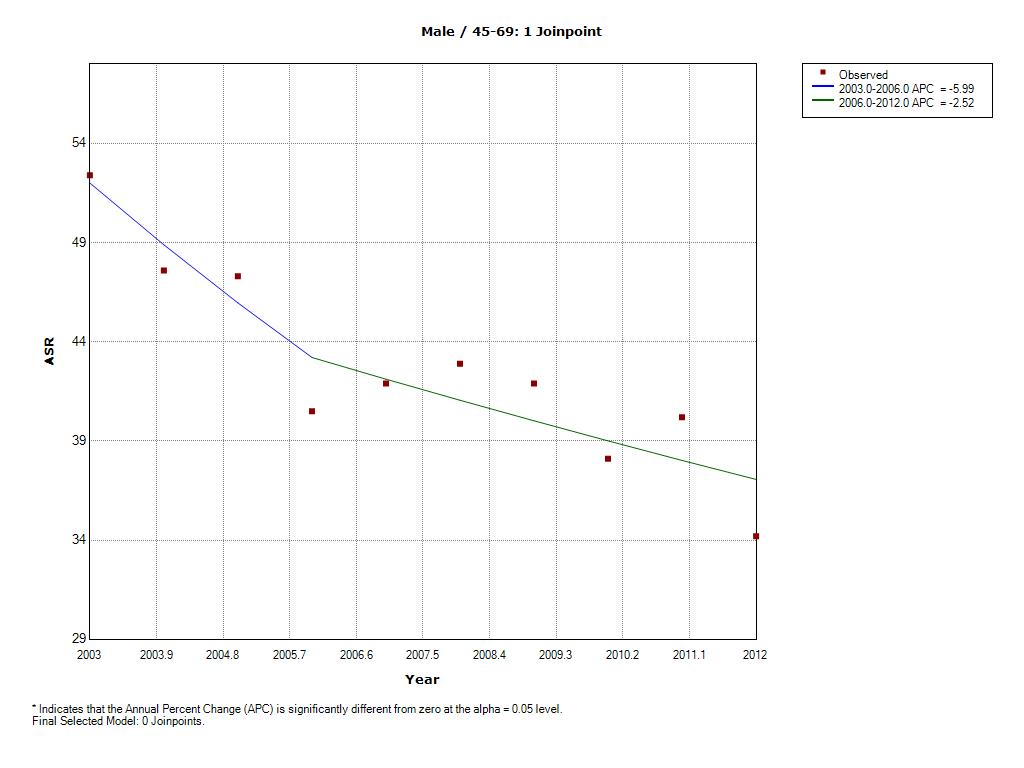

Supplement: Supplementary file 7 — Supplement Figure 7: incidence joinpoint. [file 12889_2024_19104_MOESM7_ESM.zip › Supplement Figure 7 incidence joinpoint/Croatia male 45-69.jpg]

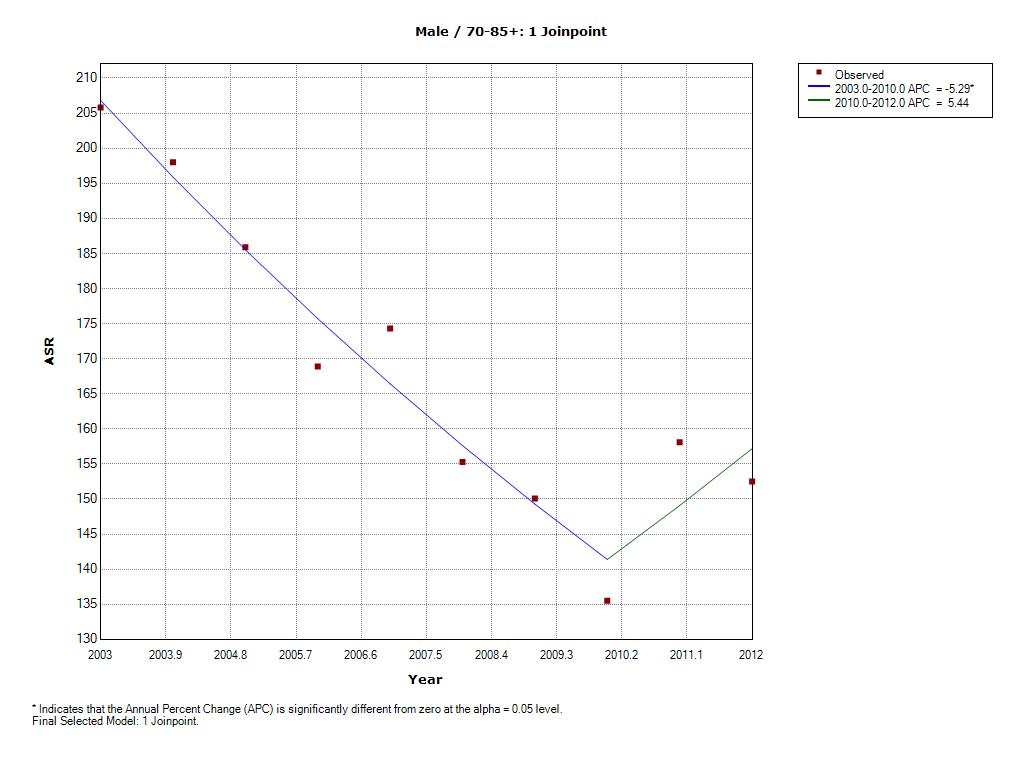

Supplement: Supplementary file 7 — Supplement Figure 7: incidence joinpoint. [file 12889_2024_19104_MOESM7_ESM.zip › Supplement Figure 7 incidence joinpoint/Croatia male 70-85+.jpg]

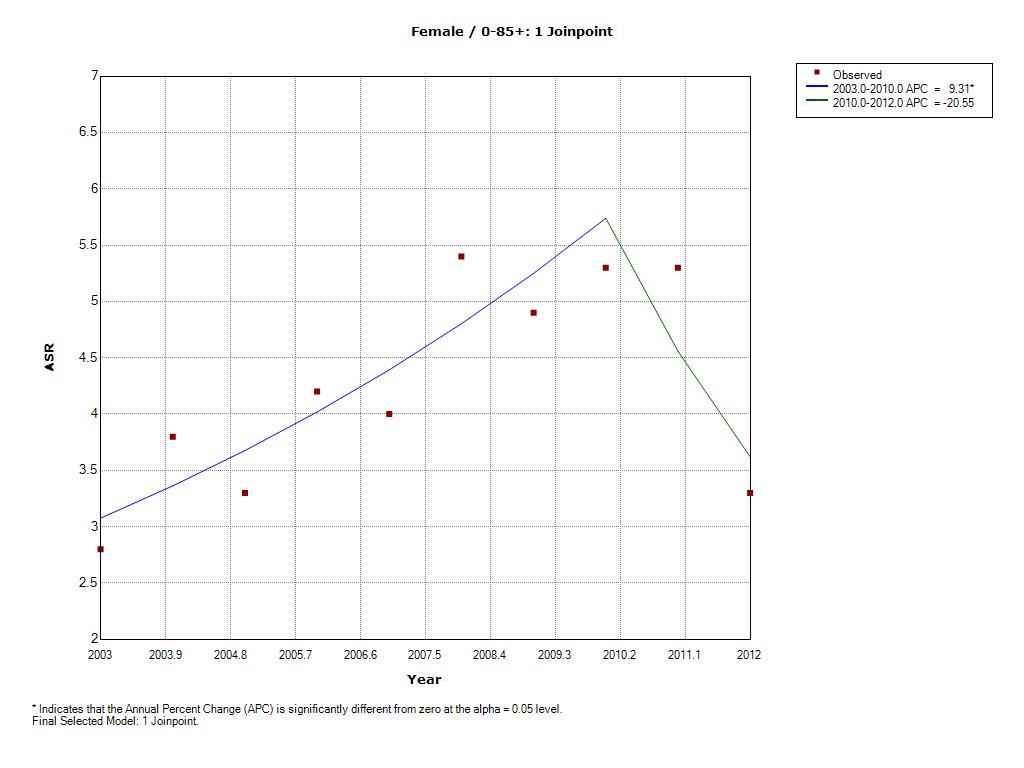

Supplement: Supplementary file 7 — Supplement Figure 7: incidence joinpoint. [file 12889_2024_19104_MOESM7_ESM.zip › Supplement Figure 7 incidence joinpoint/Cyprus female 0-85+.jpg]

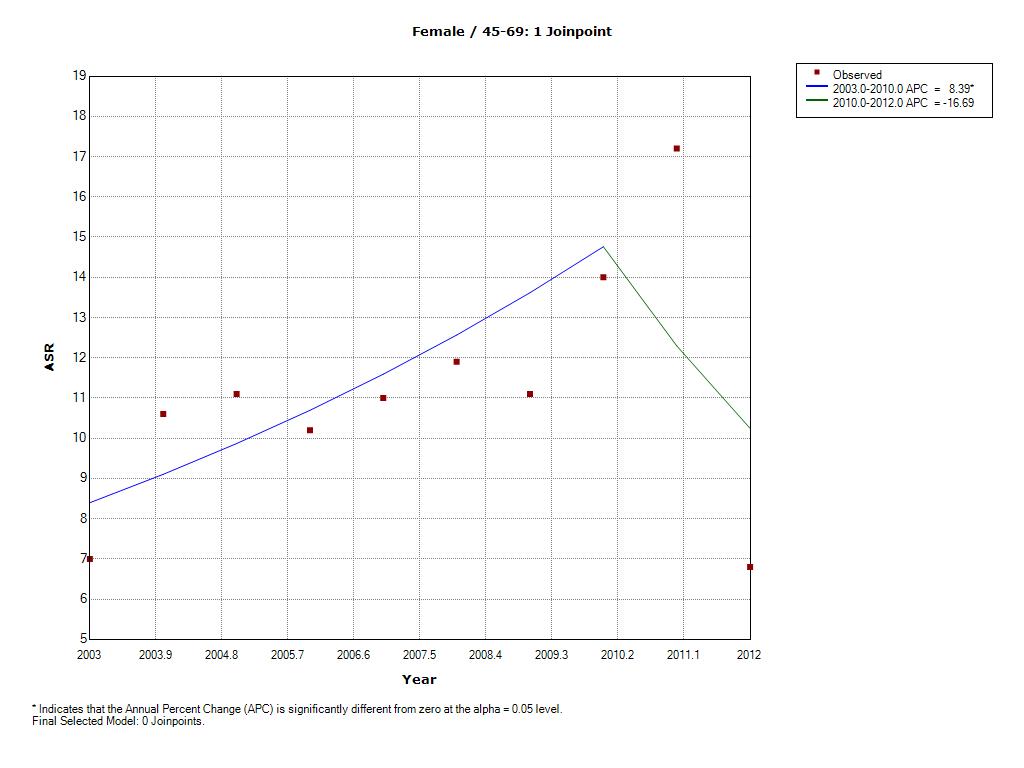

Supplement: Supplementary file 7 — Supplement Figure 7: incidence joinpoint. [file 12889_2024_19104_MOESM7_ESM.zip › Supplement Figure 7 incidence joinpoint/Cyprus female 45-69.jpg]

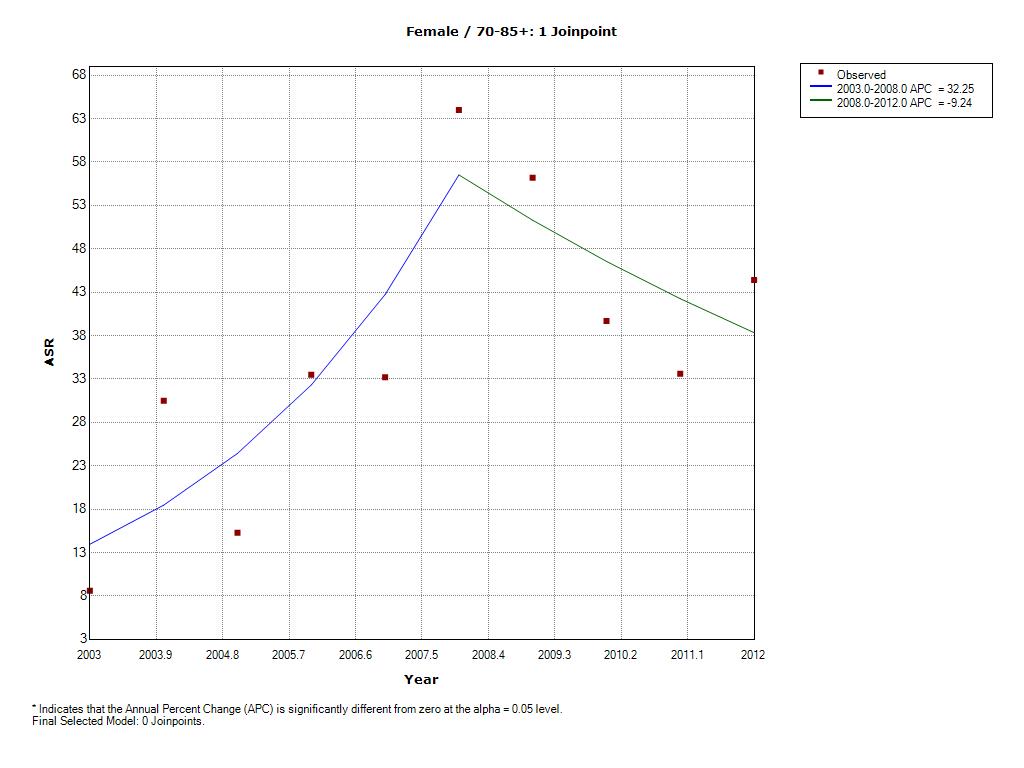

Supplement: Supplementary file 7 — Supplement Figure 7: incidence joinpoint. [file 12889_2024_19104_MOESM7_ESM.zip › Supplement Figure 7 incidence joinpoint/Cyprus female 70-85+.jpg]

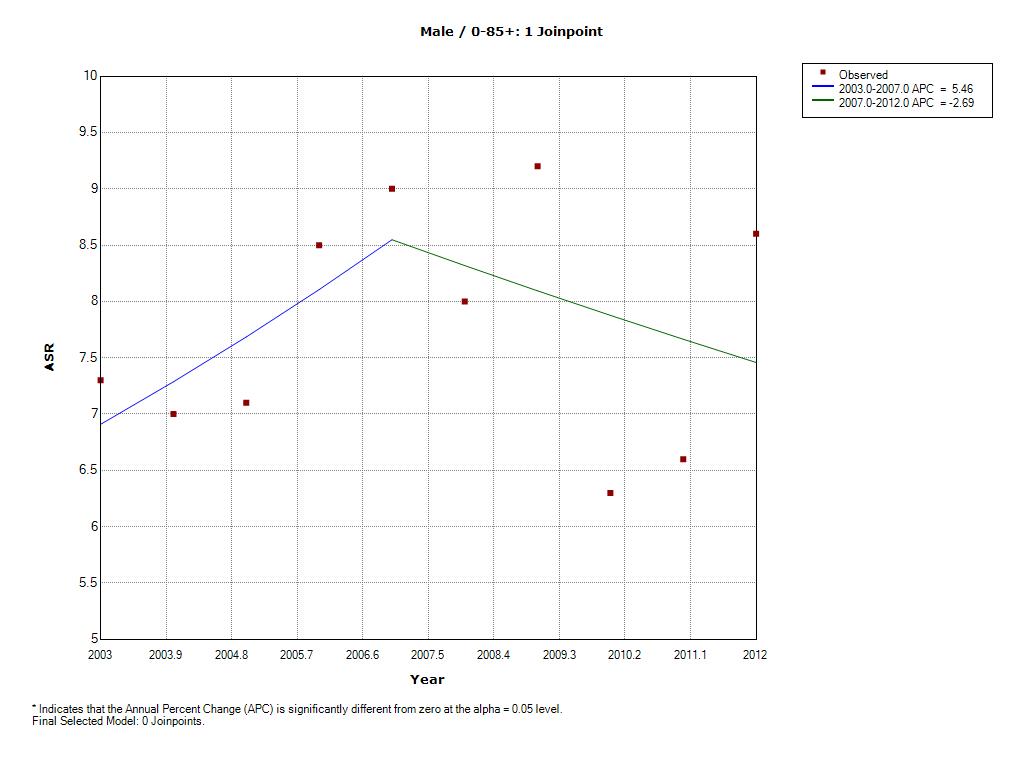

Supplement: Supplementary file 7 — Supplement Figure 7: incidence joinpoint. [file 12889_2024_19104_MOESM7_ESM.zip › Supplement Figure 7 incidence joinpoint/Cyprus male 0-85+.jpg]

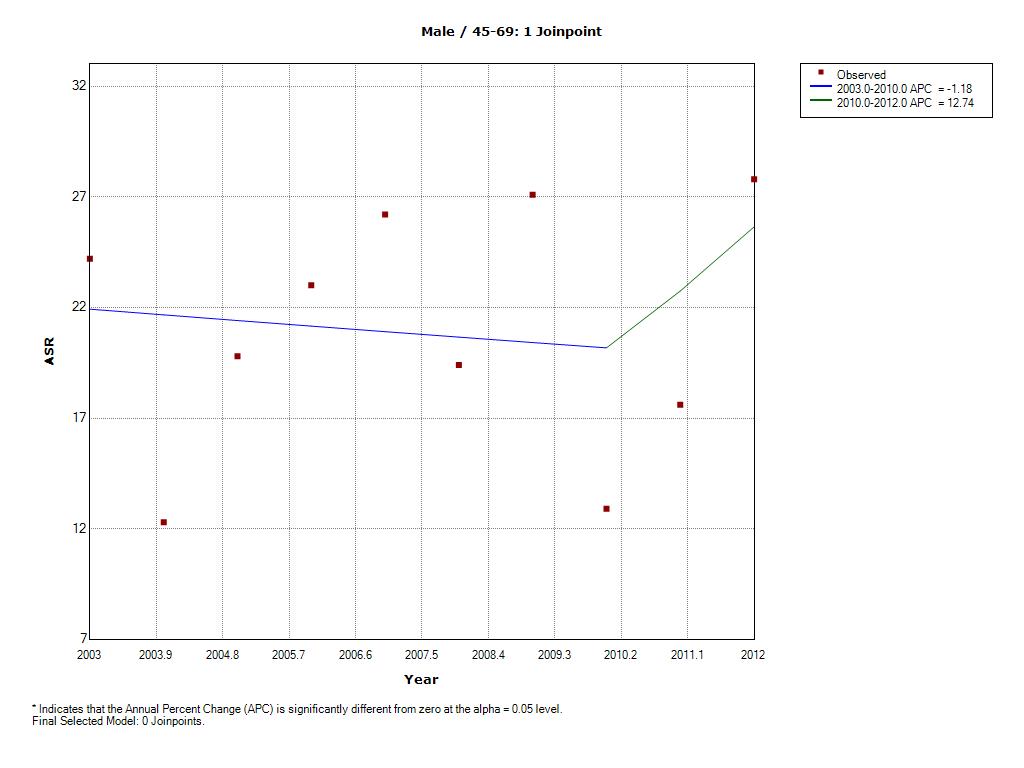

Supplement: Supplementary file 7 — Supplement Figure 7: incidence joinpoint. [file 12889_2024_19104_MOESM7_ESM.zip › Supplement Figure 7 incidence joinpoint/Cyprus male 45-69.jpg]

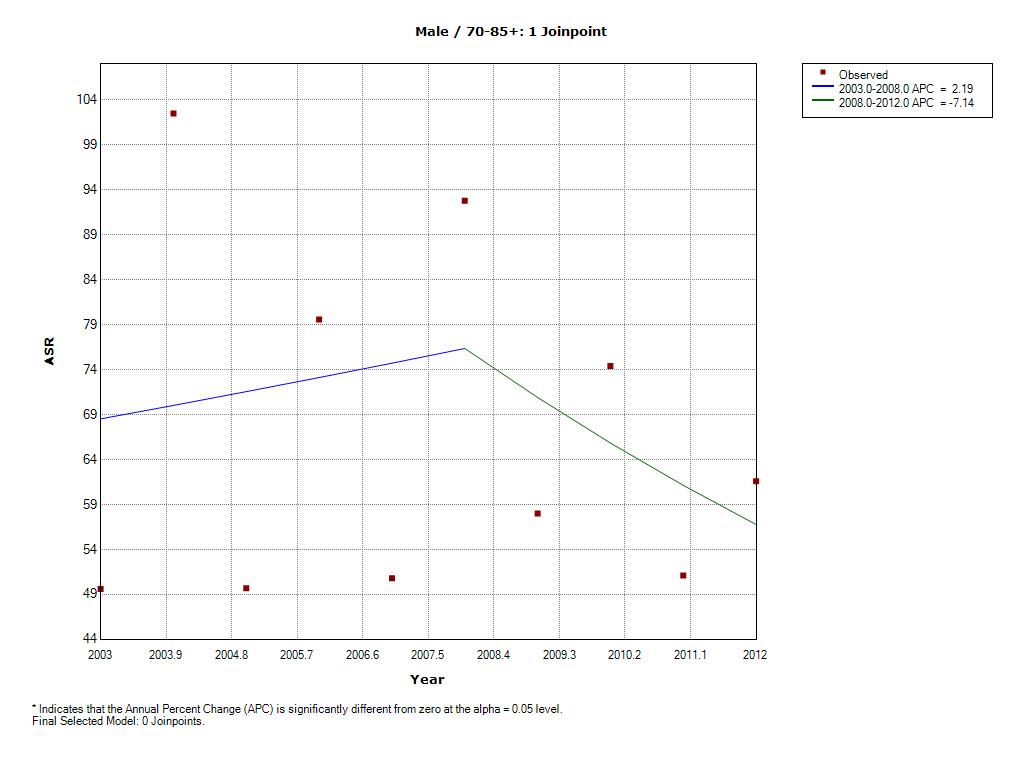

Supplement: Supplementary file 7 — Supplement Figure 7: incidence joinpoint. [file 12889_2024_19104_MOESM7_ESM.zip › Supplement Figure 7 incidence joinpoint/Cyprus male 70-85+.jpg]

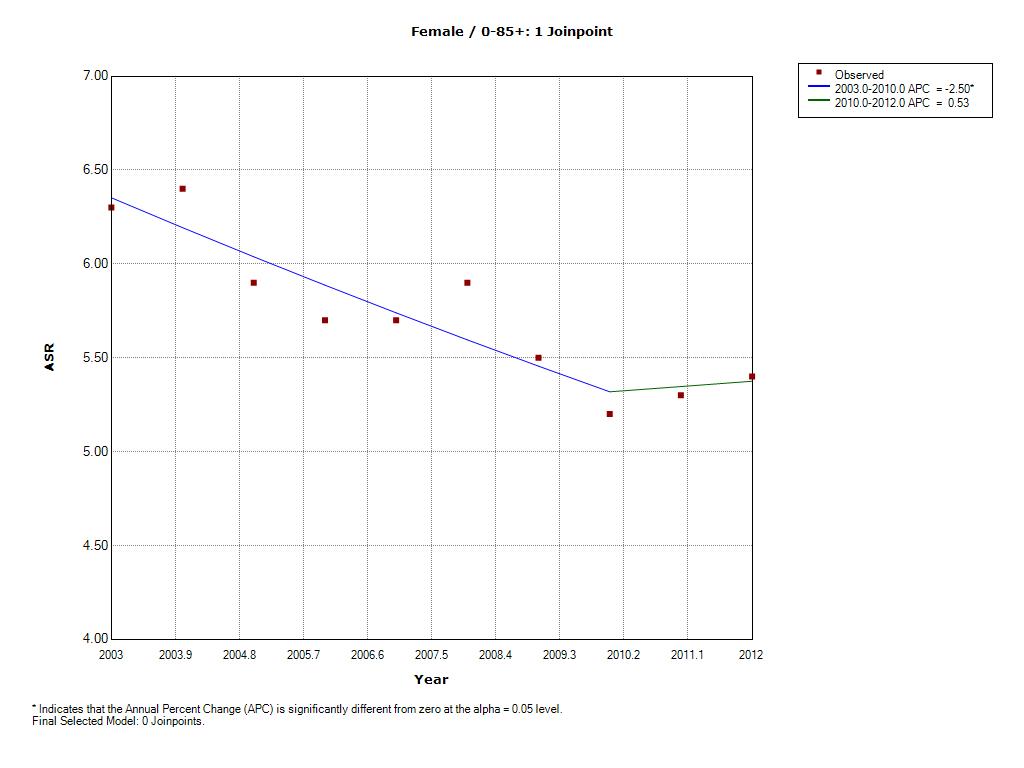

Supplement: Supplementary file 7 — Supplement Figure 7: incidence joinpoint. [file 12889_2024_19104_MOESM7_ESM.zip › Supplement Figure 7 incidence joinpoint/Czech Republic female 0-85+.jpg]

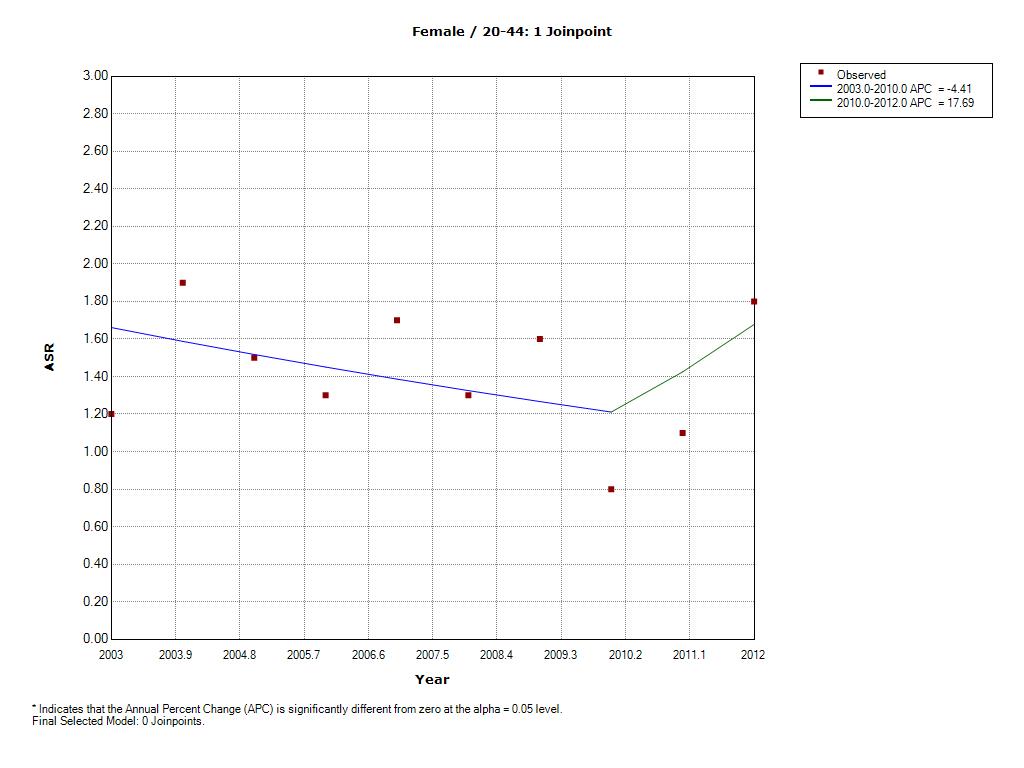

Supplement: Supplementary file 7 — Supplement Figure 7: incidence joinpoint. [file 12889_2024_19104_MOESM7_ESM.zip › Supplement Figure 7 incidence joinpoint/Czech Republic female 20-44.jpg]

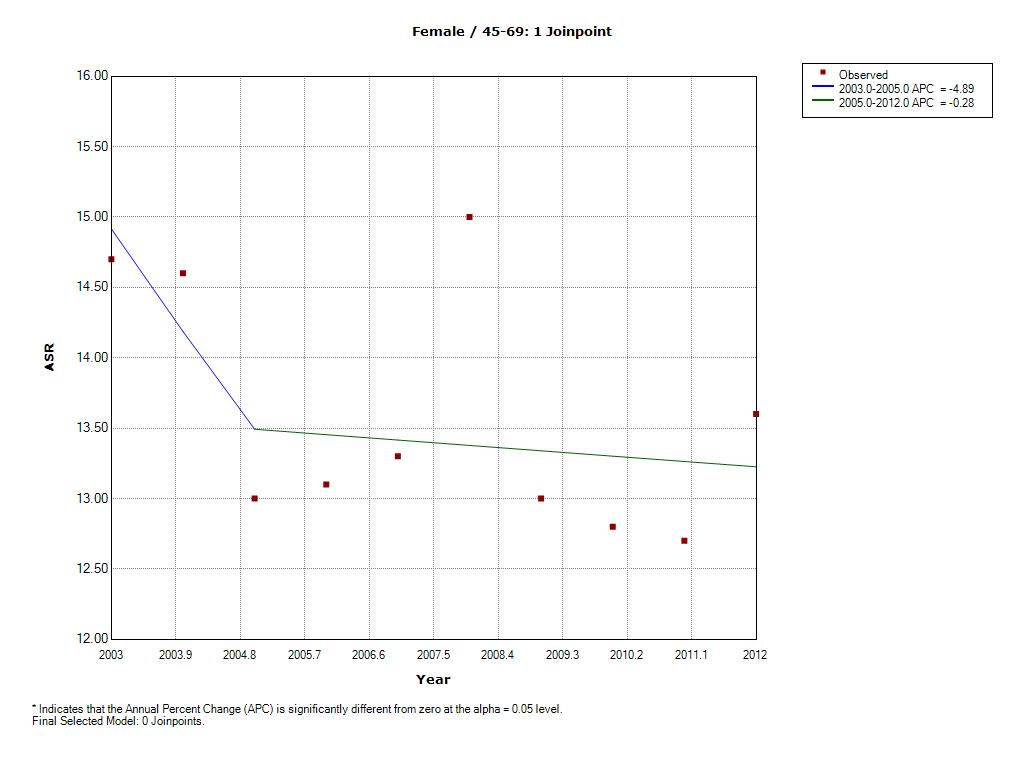

Supplement: Supplementary file 7 — Supplement Figure 7: incidence joinpoint. [file 12889_2024_19104_MOESM7_ESM.zip › Supplement Figure 7 incidence joinpoint/Czech Republic female 45-69.jpg]

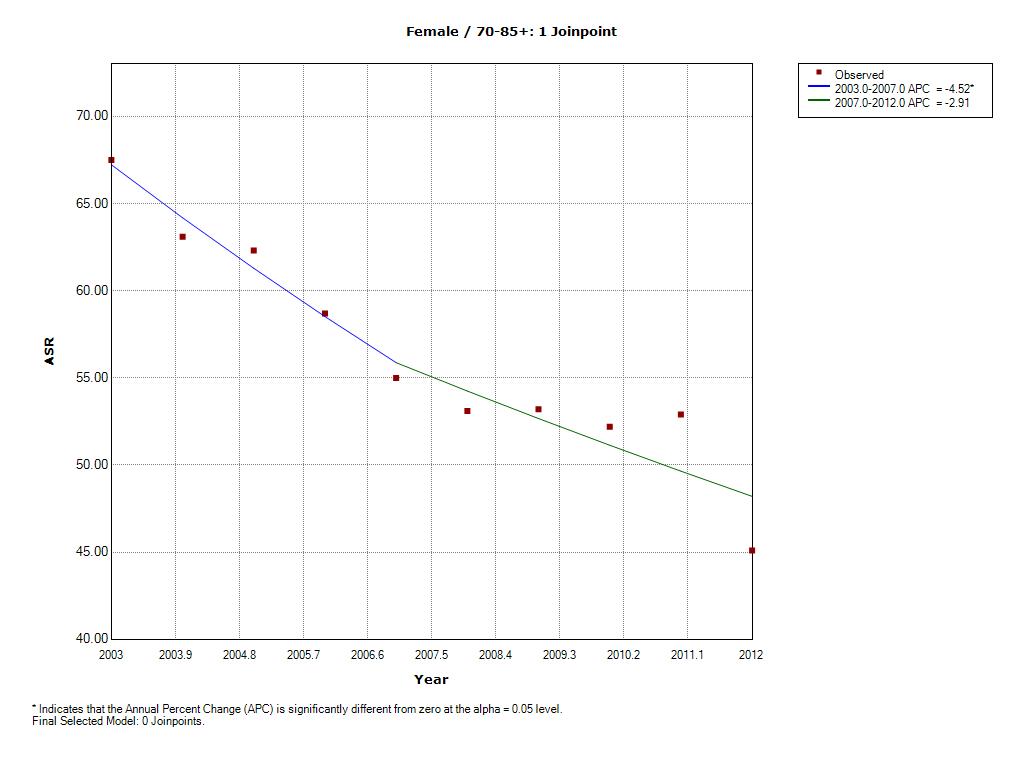

Supplement: Supplementary file 7 — Supplement Figure 7: incidence joinpoint. [file 12889_2024_19104_MOESM7_ESM.zip › Supplement Figure 7 incidence joinpoint/Czech Republic female 70-85+.jpg]

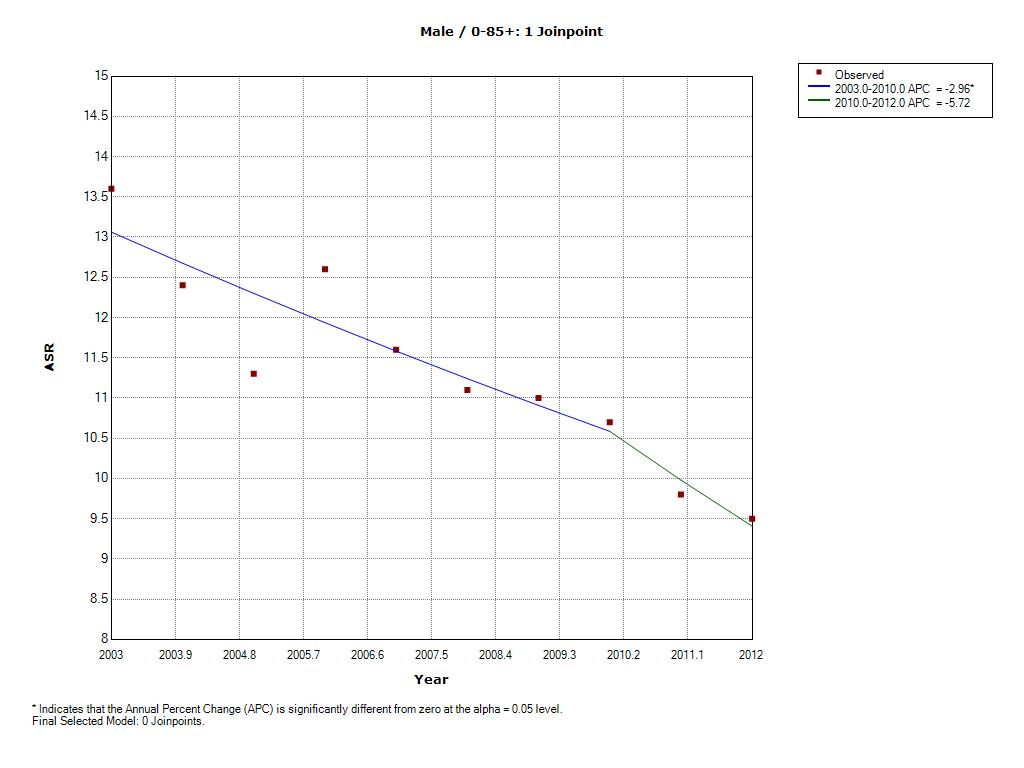

Supplement: Supplementary file 7 — Supplement Figure 7: incidence joinpoint. [file 12889_2024_19104_MOESM7_ESM.zip › Supplement Figure 7 incidence joinpoint/Czech Republic male 0-85+.jpg]

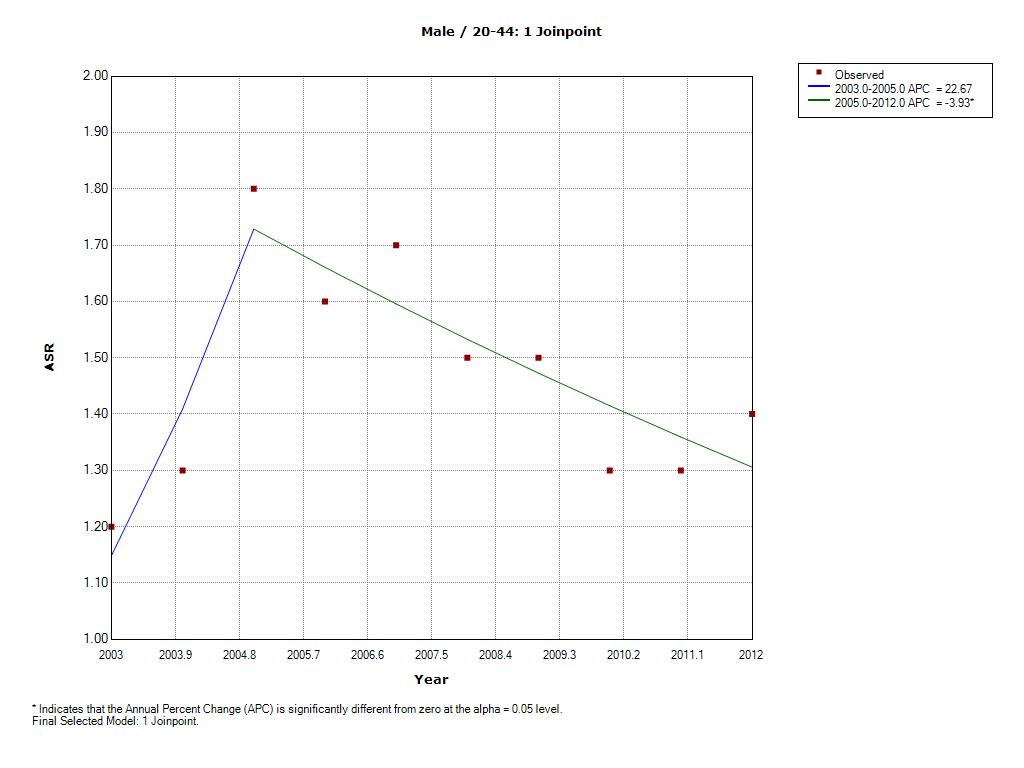

Supplement: Supplementary file 7 — Supplement Figure 7: incidence joinpoint. [file 12889_2024_19104_MOESM7_ESM.zip › Supplement Figure 7 incidence joinpoint/Czech Republic male 20-44.jpg]

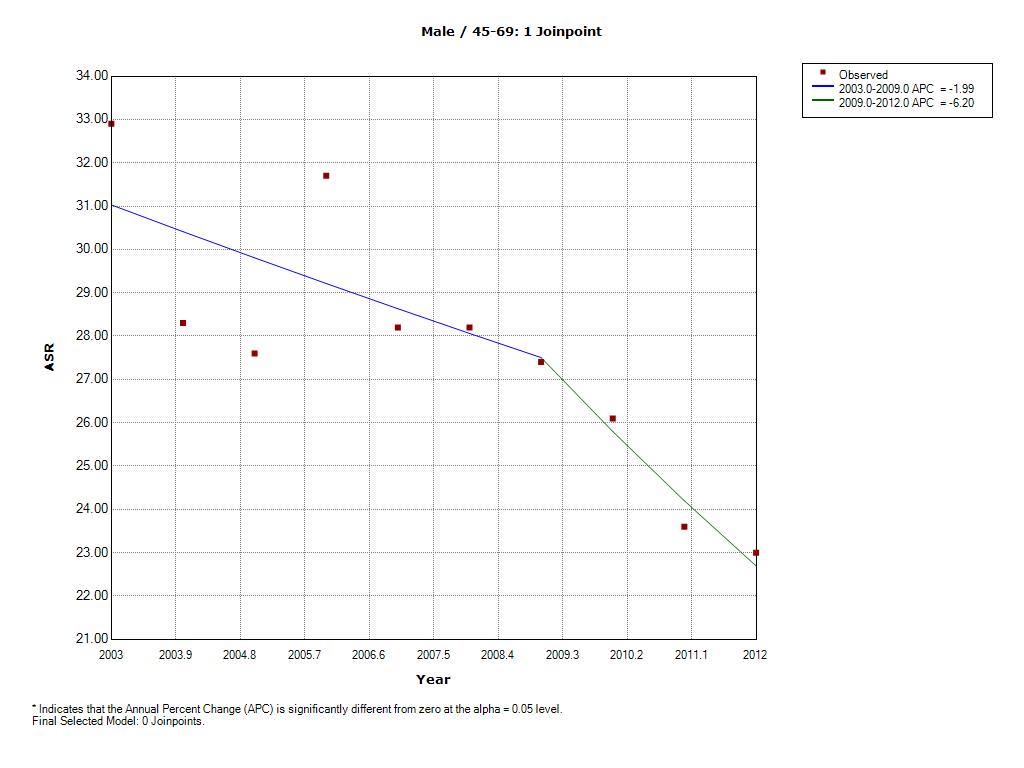

Supplement: Supplementary file 7 — Supplement Figure 7: incidence joinpoint. [file 12889_2024_19104_MOESM7_ESM.zip › Supplement Figure 7 incidence joinpoint/Czech Republic male 45-69.jpg]

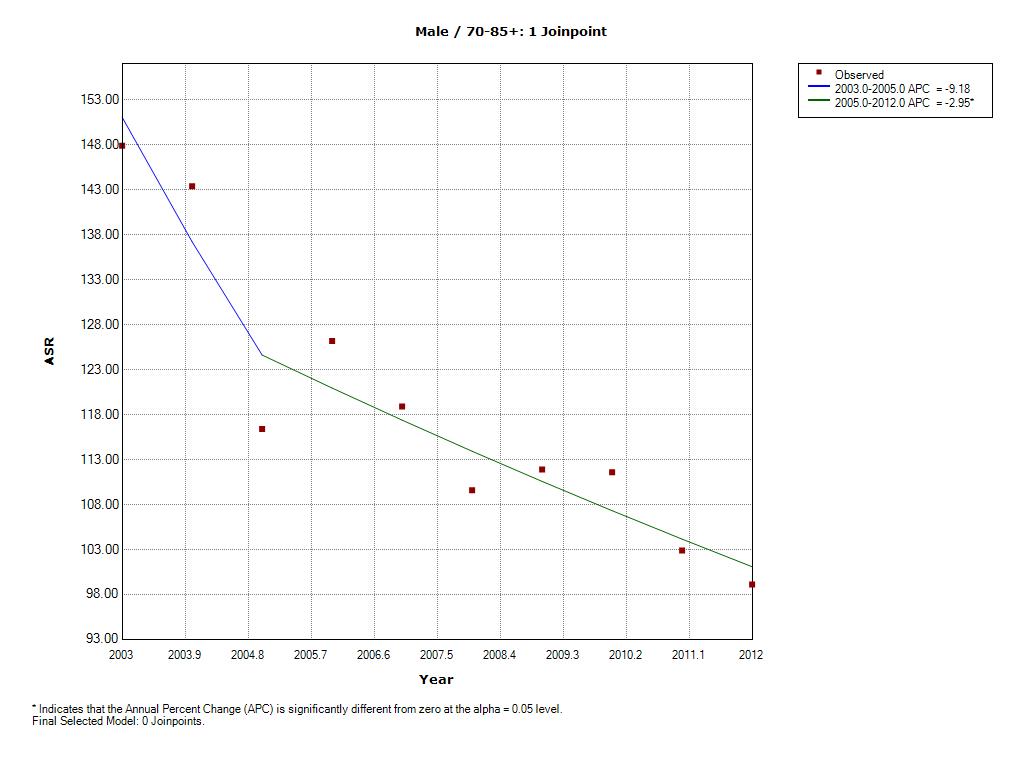

Supplement: Supplementary file 7 — Supplement Figure 7: incidence joinpoint. [file 12889_2024_19104_MOESM7_ESM.zip › Supplement Figure 7 incidence joinpoint/Czech Republic male 70-85+.jpg]
