# Supplementary figures and images for: Global incidence and mortality trends of gastric cancer and predicted mortality of gastric cancer by 2035 (part 3 of 4)
Source: BMC Public Health. 2024 Jul 2;24:1763. doi: 10.1186/s12889-024-19104-6 (PMC11221210; doi:10.1186/s12889-024-19104-6)

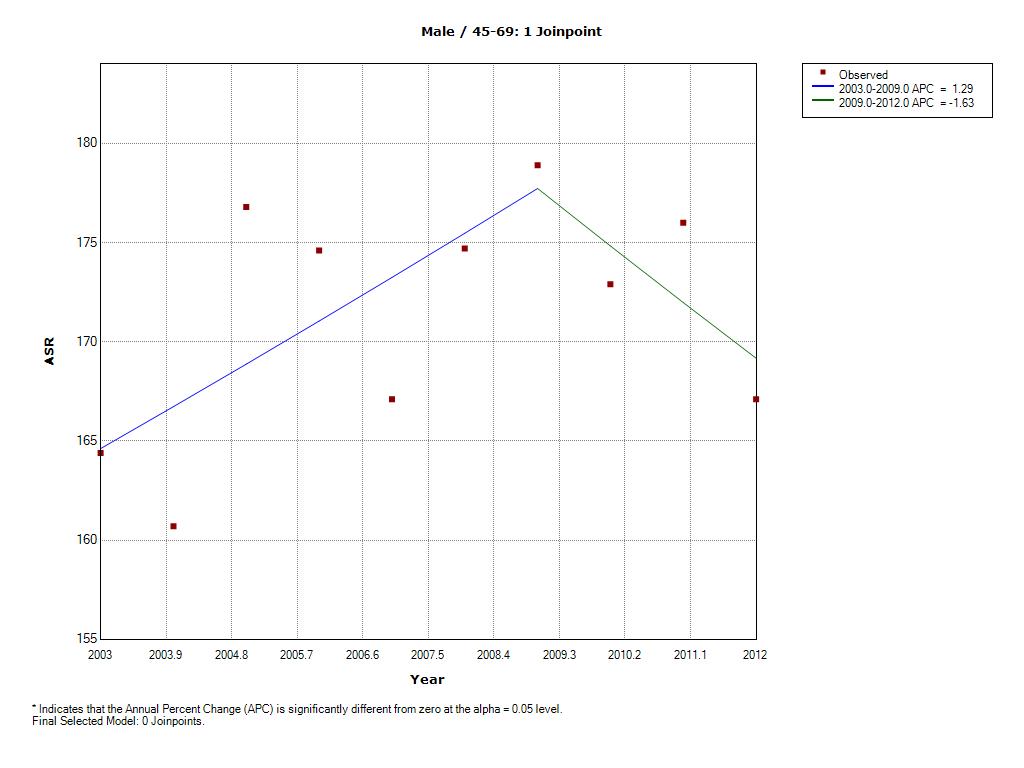

Supplement: Supplementary file 7 — Supplement Figure 7: incidence joinpoint. [file 12889_2024_19104_MOESM7_ESM.zip › Supplement Figure 7 incidence joinpoint/Korea male 45-69.jpg]

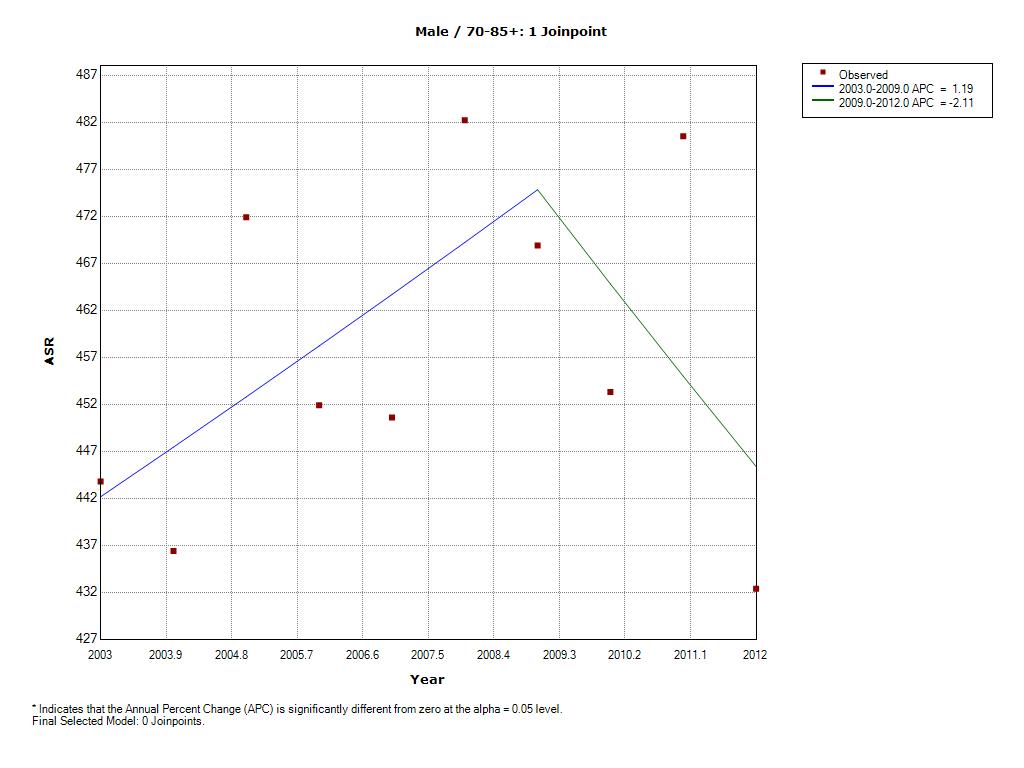

Supplement: Supplementary file 7 — Supplement Figure 7: incidence joinpoint. [file 12889_2024_19104_MOESM7_ESM.zip › Supplement Figure 7 incidence joinpoint/Korea male 70-85+.jpg]

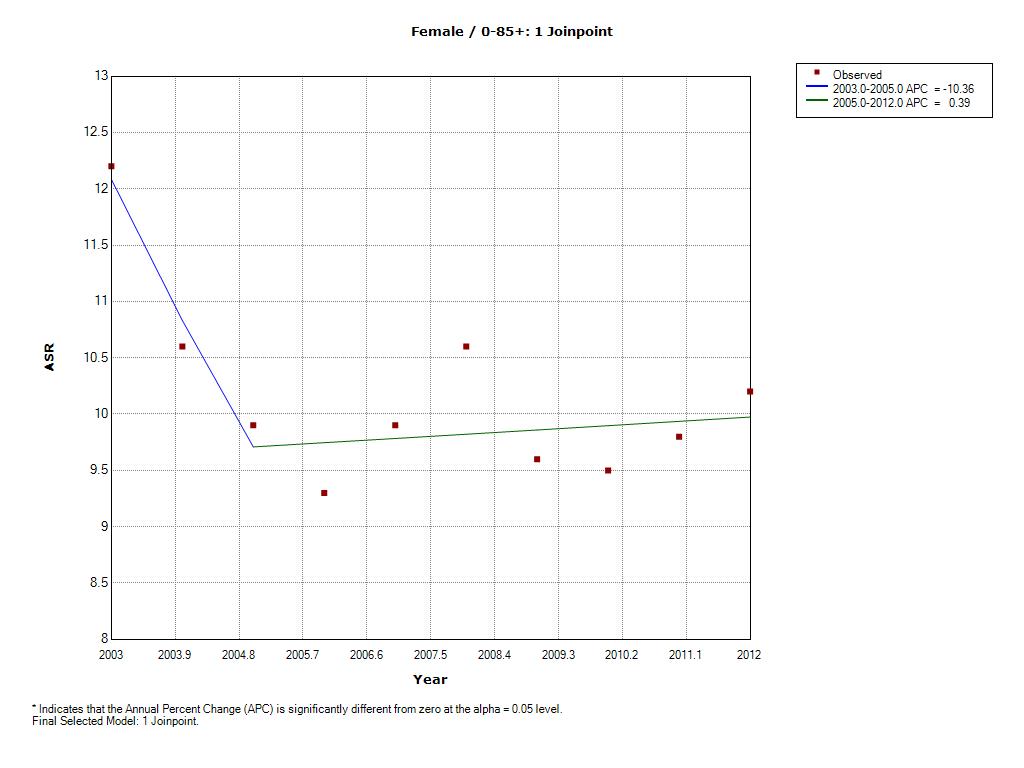

Supplement: Supplementary file 7 — Supplement Figure 7: incidence joinpoint. [file 12889_2024_19104_MOESM7_ESM.zip › Supplement Figure 7 incidence joinpoint/Lithuania female 0-85+.jpg]

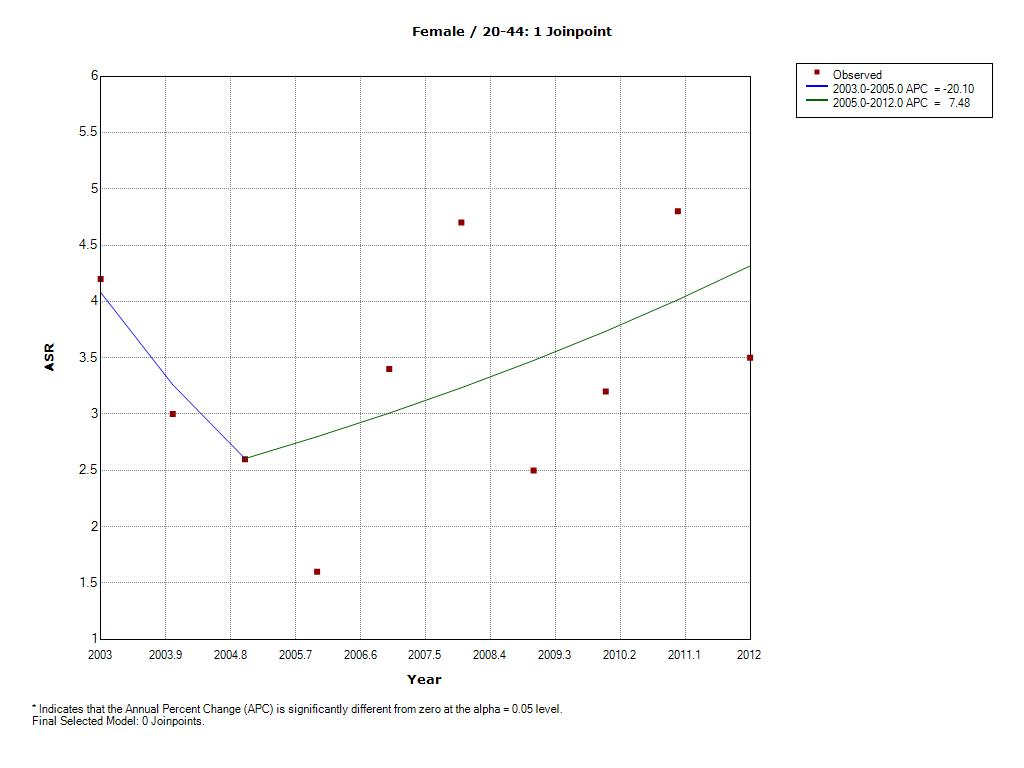

Supplement: Supplementary file 7 — Supplement Figure 7: incidence joinpoint. [file 12889_2024_19104_MOESM7_ESM.zip › Supplement Figure 7 incidence joinpoint/Lithuania female 20-44.jpg]

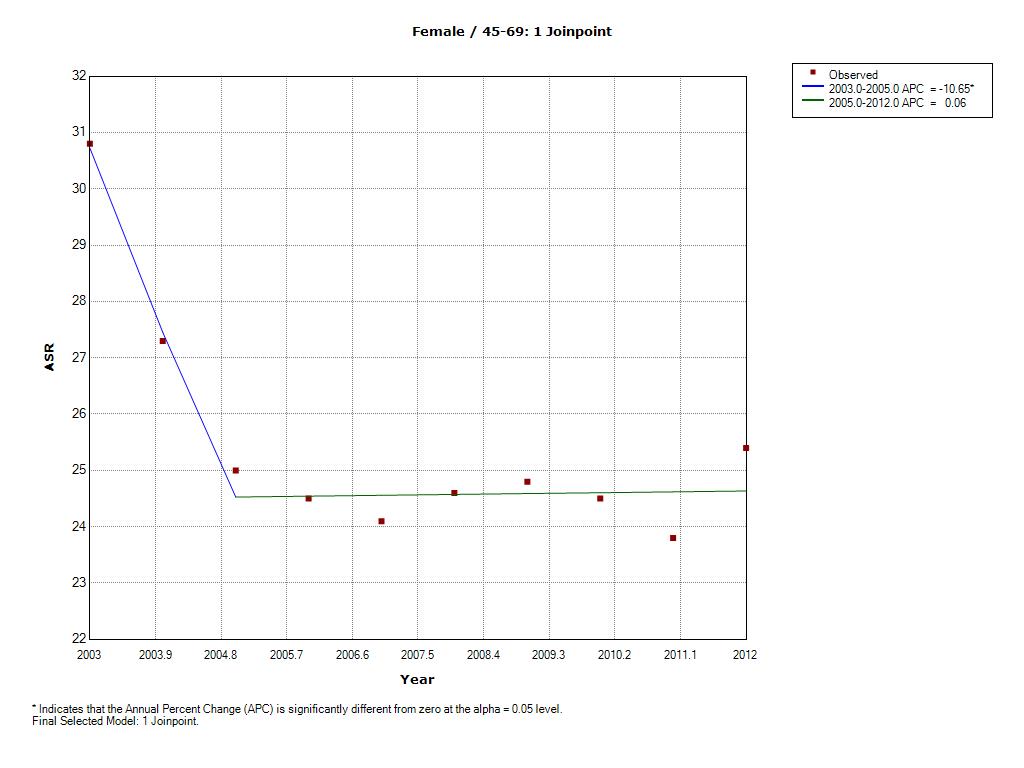

Supplement: Supplementary file 7 — Supplement Figure 7: incidence joinpoint. [file 12889_2024_19104_MOESM7_ESM.zip › Supplement Figure 7 incidence joinpoint/Lithuania female 45-69.jpg]

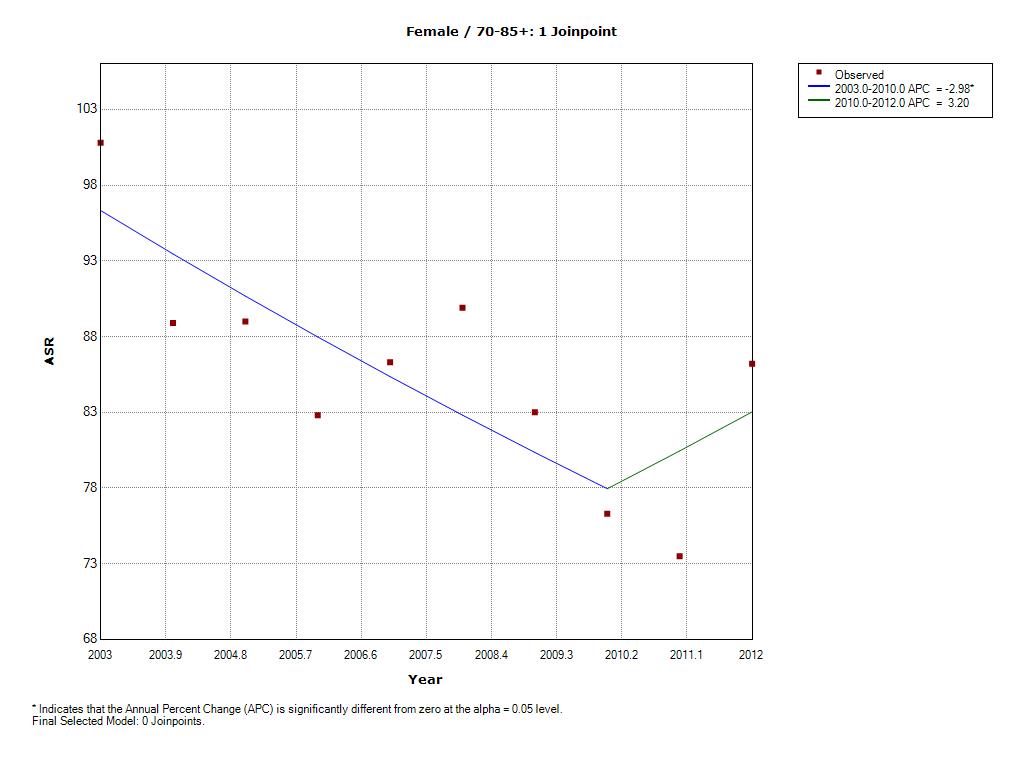

Supplement: Supplementary file 7 — Supplement Figure 7: incidence joinpoint. [file 12889_2024_19104_MOESM7_ESM.zip › Supplement Figure 7 incidence joinpoint/Lithuania female 70-85+.jpg]

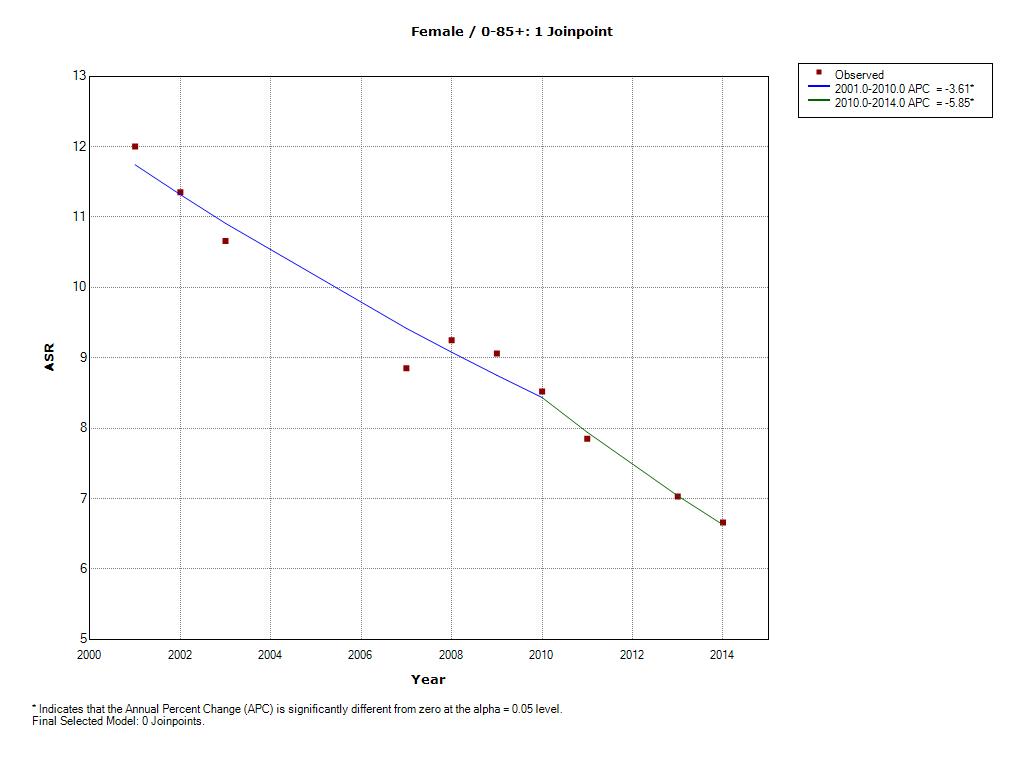

Supplement: Supplementary file 8 — Supplement Figure 8: mortality joinpoint. [file 12889_2024_19104_MOESM8_ESM.zip › Supplement Figure 8 mortality joinpoint/Belarus female 0-85+.jpg]

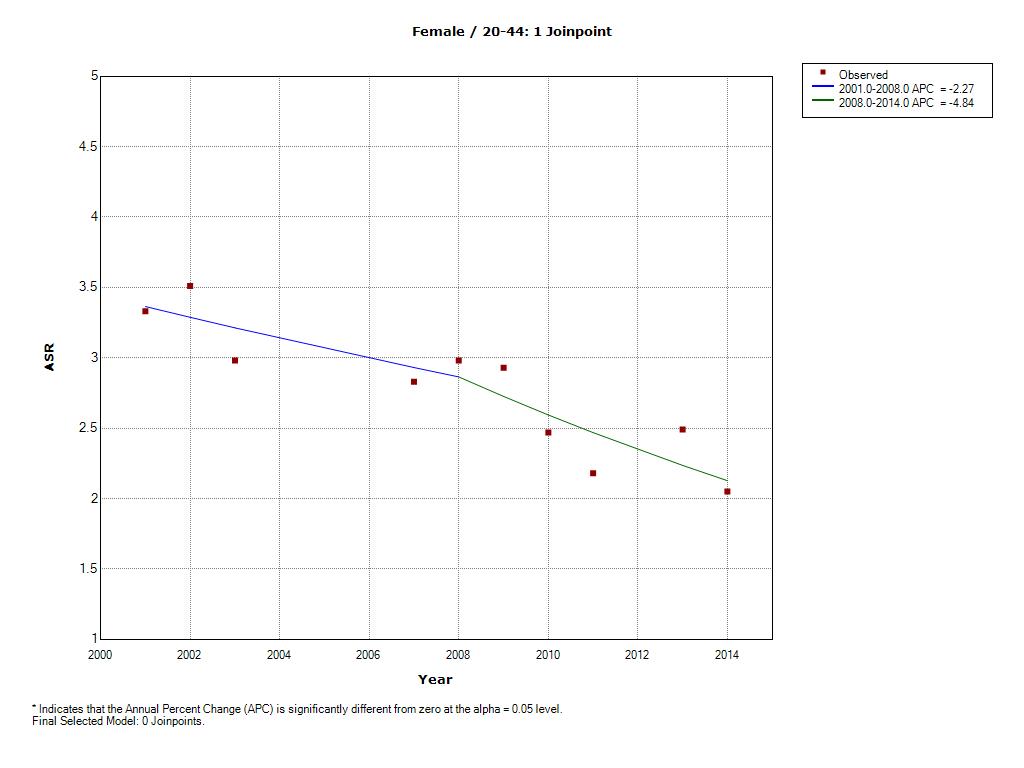

Supplement: Supplementary file 8 — Supplement Figure 8: mortality joinpoint. [file 12889_2024_19104_MOESM8_ESM.zip › Supplement Figure 8 mortality joinpoint/Belarus female 20-44.jpg]

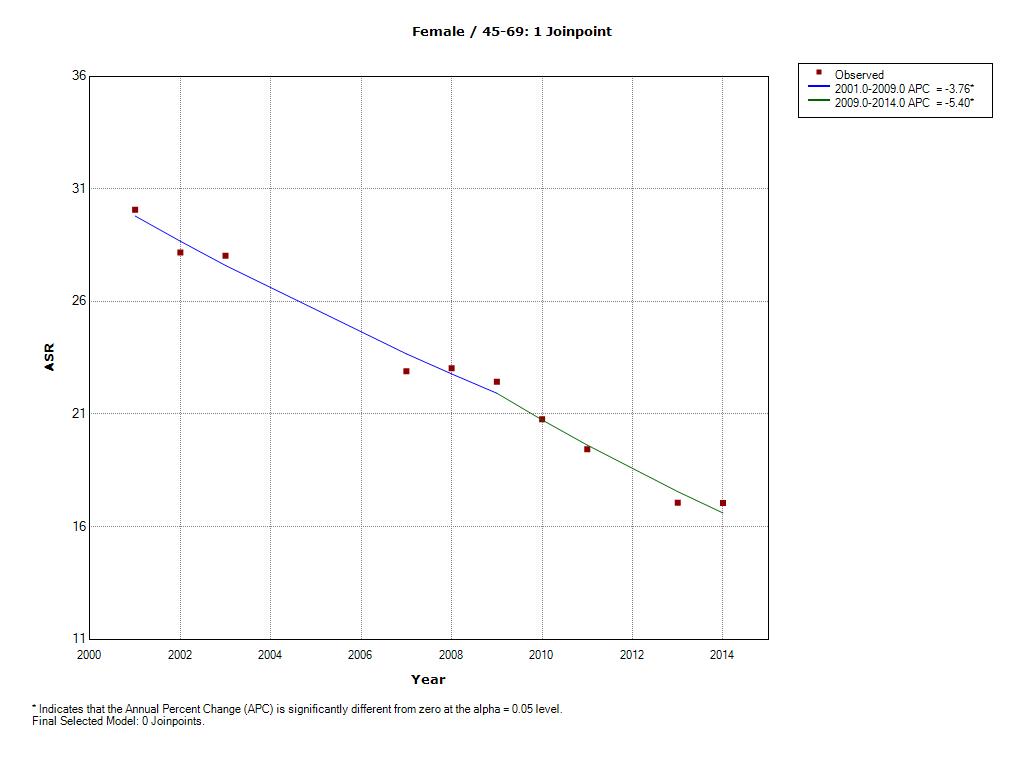

Supplement: Supplementary file 8 — Supplement Figure 8: mortality joinpoint. [file 12889_2024_19104_MOESM8_ESM.zip › Supplement Figure 8 mortality joinpoint/Belarus female 45-69.jpg]

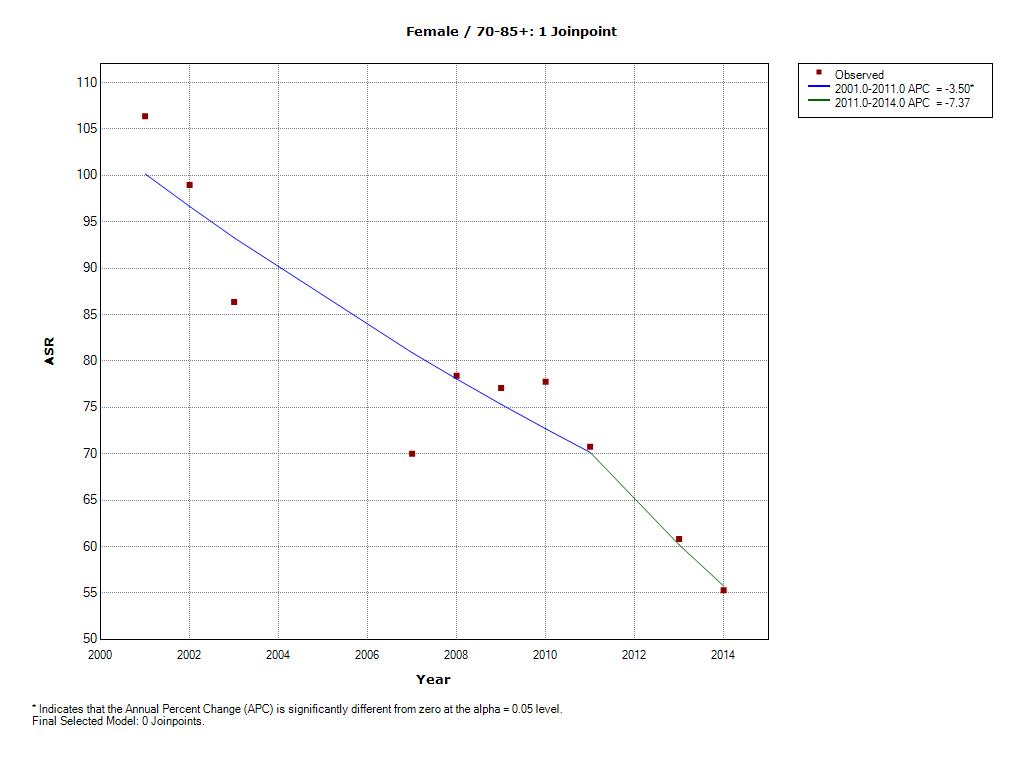

Supplement: Supplementary file 8 — Supplement Figure 8: mortality joinpoint. [file 12889_2024_19104_MOESM8_ESM.zip › Supplement Figure 8 mortality joinpoint/Belarus female 70-85+.jpg]

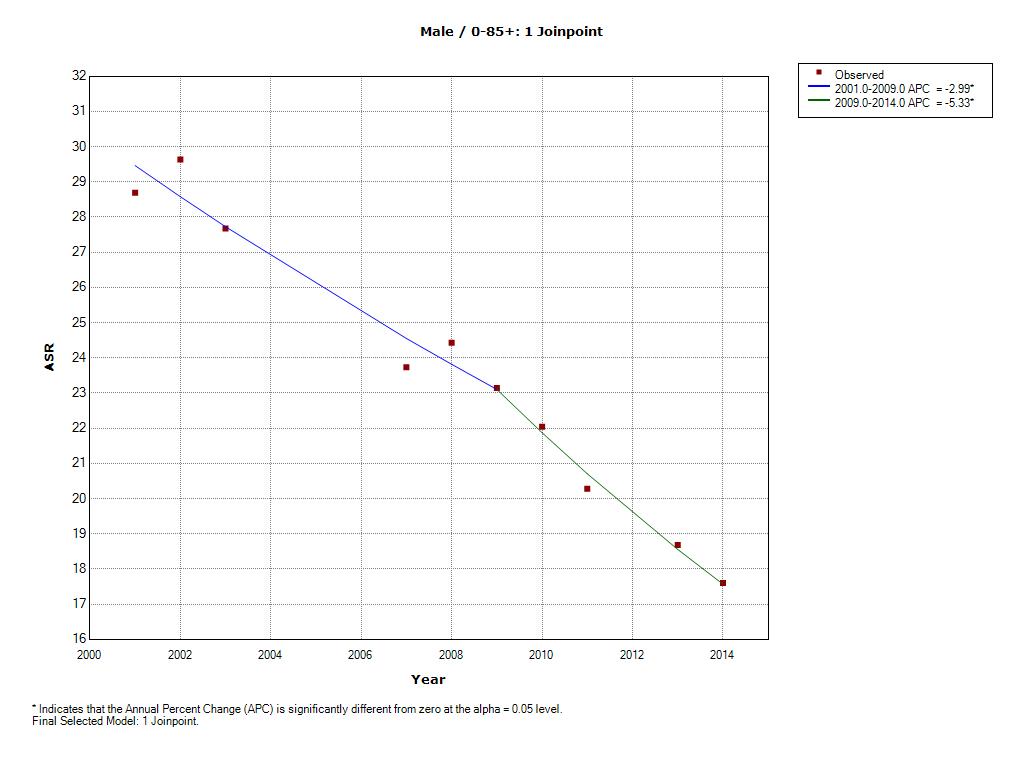

Supplement: Supplementary file 8 — Supplement Figure 8: mortality joinpoint. [file 12889_2024_19104_MOESM8_ESM.zip › Supplement Figure 8 mortality joinpoint/Belarus male 0-85+.jpg]

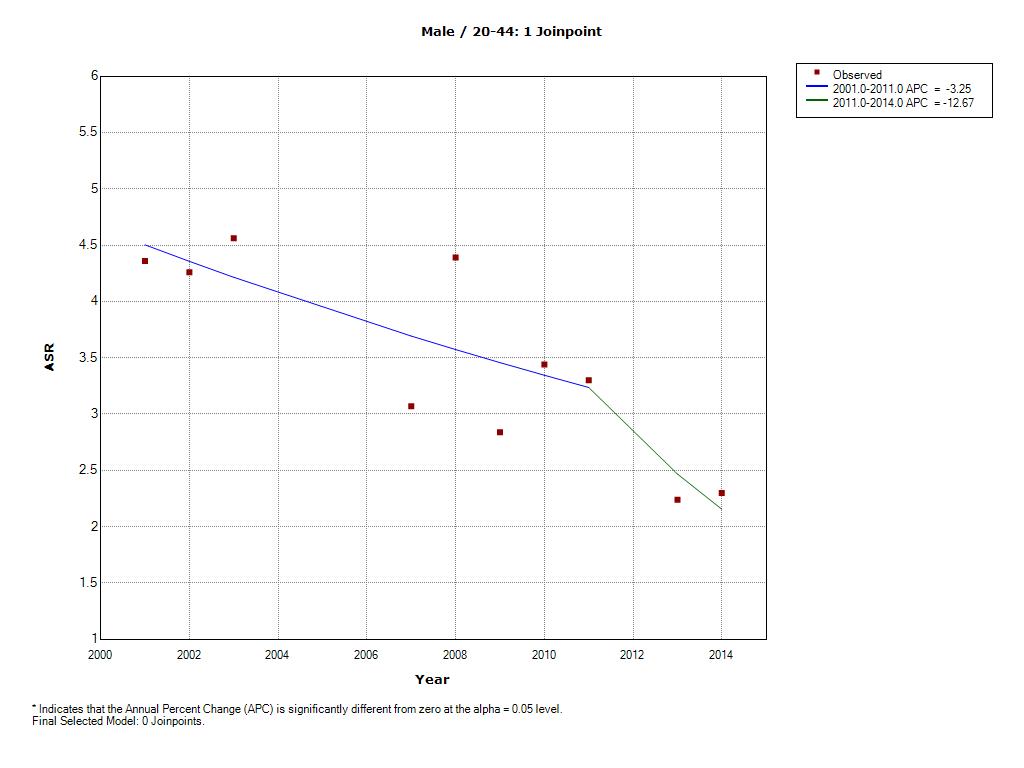

Supplement: Supplementary file 8 — Supplement Figure 8: mortality joinpoint. [file 12889_2024_19104_MOESM8_ESM.zip › Supplement Figure 8 mortality joinpoint/Belarus male 20-45.jpg]

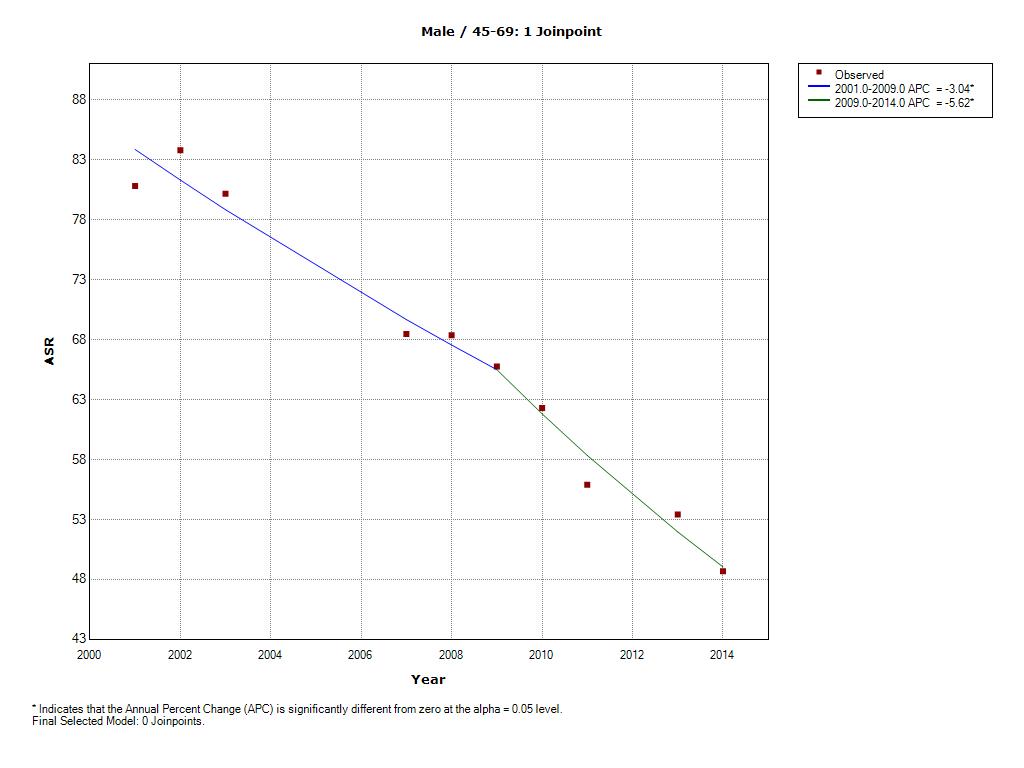

Supplement: Supplementary file 8 — Supplement Figure 8: mortality joinpoint. [file 12889_2024_19104_MOESM8_ESM.zip › Supplement Figure 8 mortality joinpoint/Belarus male 45-69.jpg]

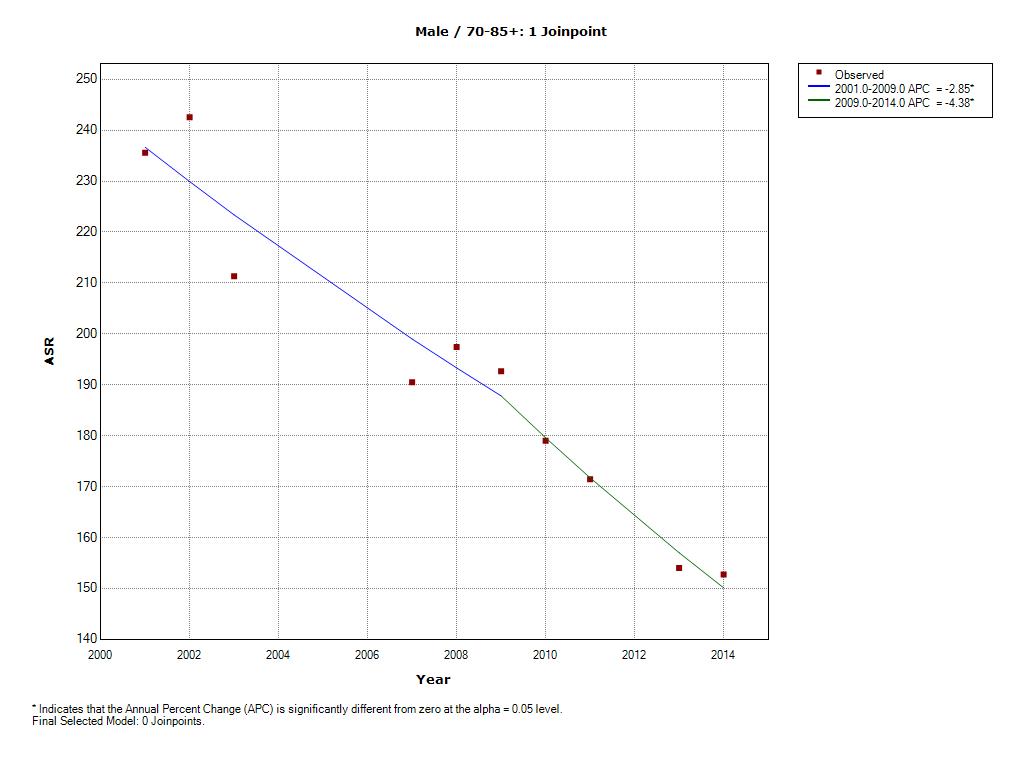

Supplement: Supplementary file 8 — Supplement Figure 8: mortality joinpoint. [file 12889_2024_19104_MOESM8_ESM.zip › Supplement Figure 8 mortality joinpoint/Belarus male 70-85+.jpg]

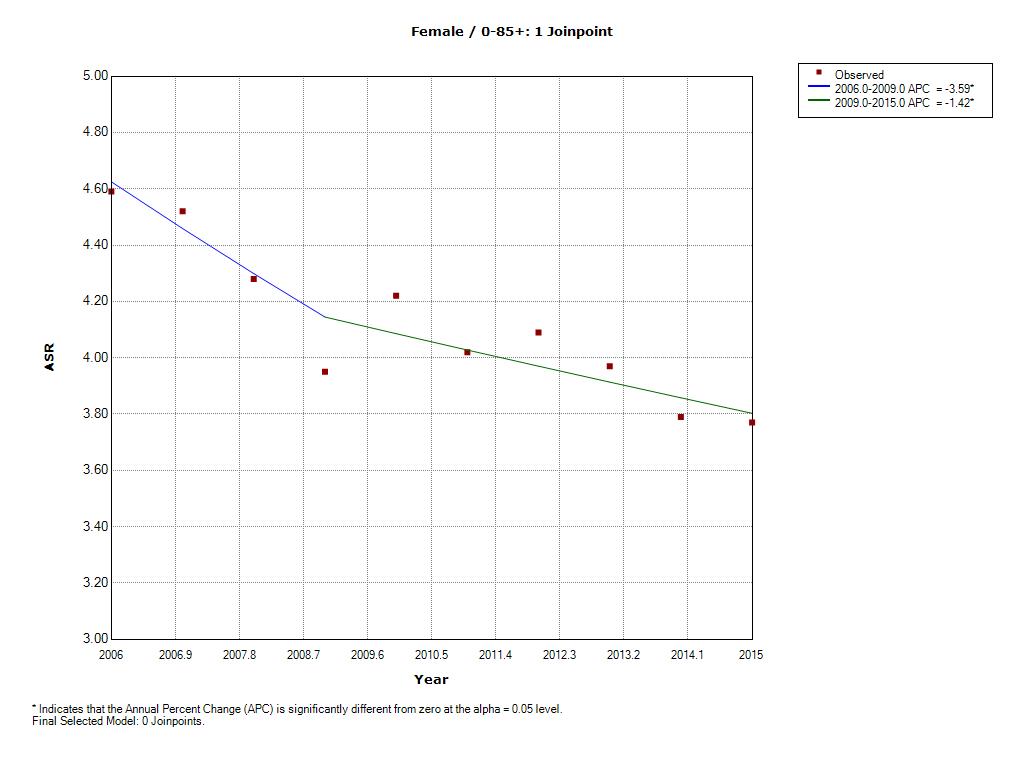

Supplement: Supplementary file 8 — Supplement Figure 8: mortality joinpoint. [file 12889_2024_19104_MOESM8_ESM.zip › Supplement Figure 8 mortality joinpoint/Brazil female 0-85+.jpg]

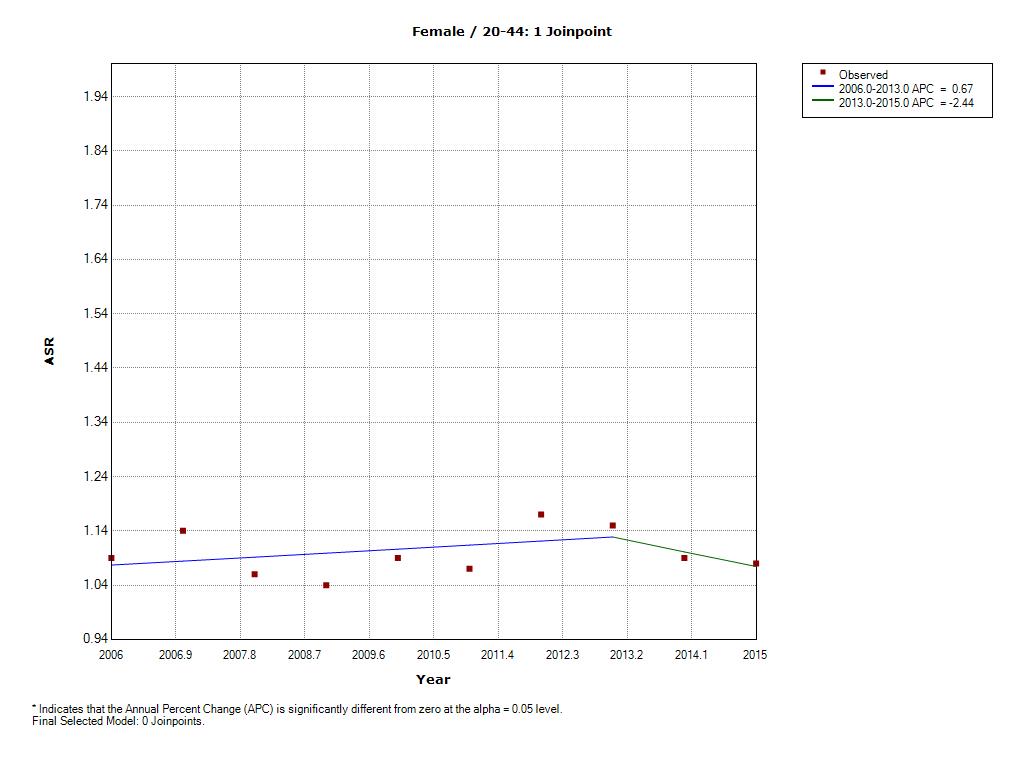

Supplement: Supplementary file 8 — Supplement Figure 8: mortality joinpoint. [file 12889_2024_19104_MOESM8_ESM.zip › Supplement Figure 8 mortality joinpoint/Brazil female 20-44.jpg]

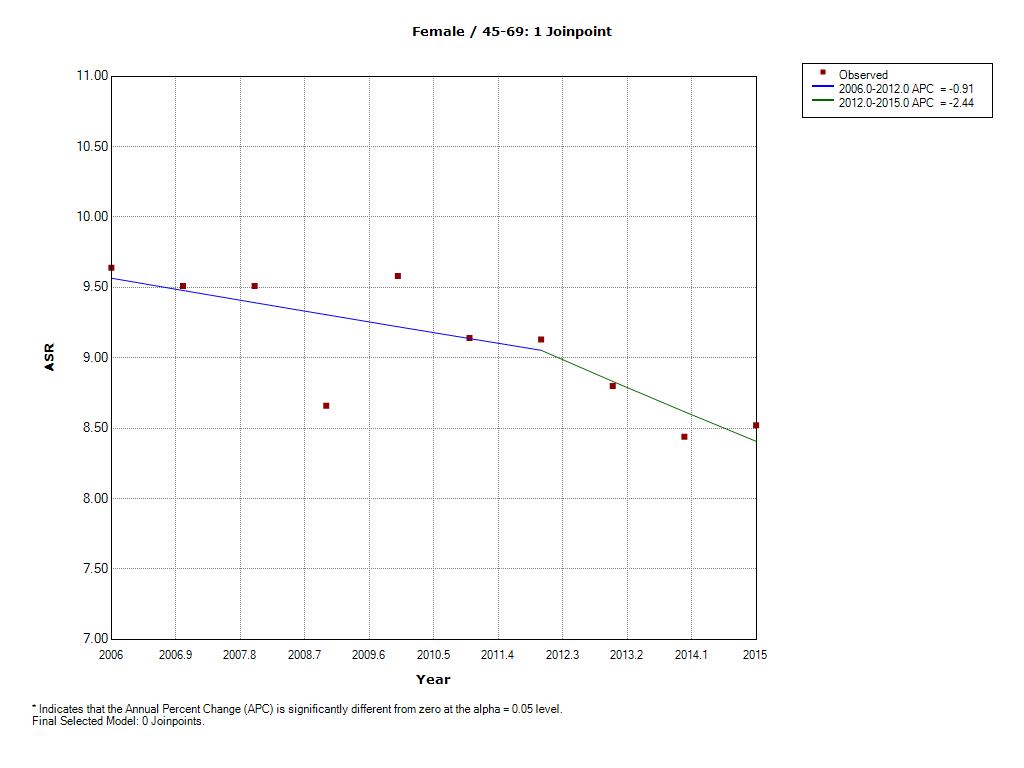

Supplement: Supplementary file 8 — Supplement Figure 8: mortality joinpoint. [file 12889_2024_19104_MOESM8_ESM.zip › Supplement Figure 8 mortality joinpoint/Brazil female 45-69.jpg]

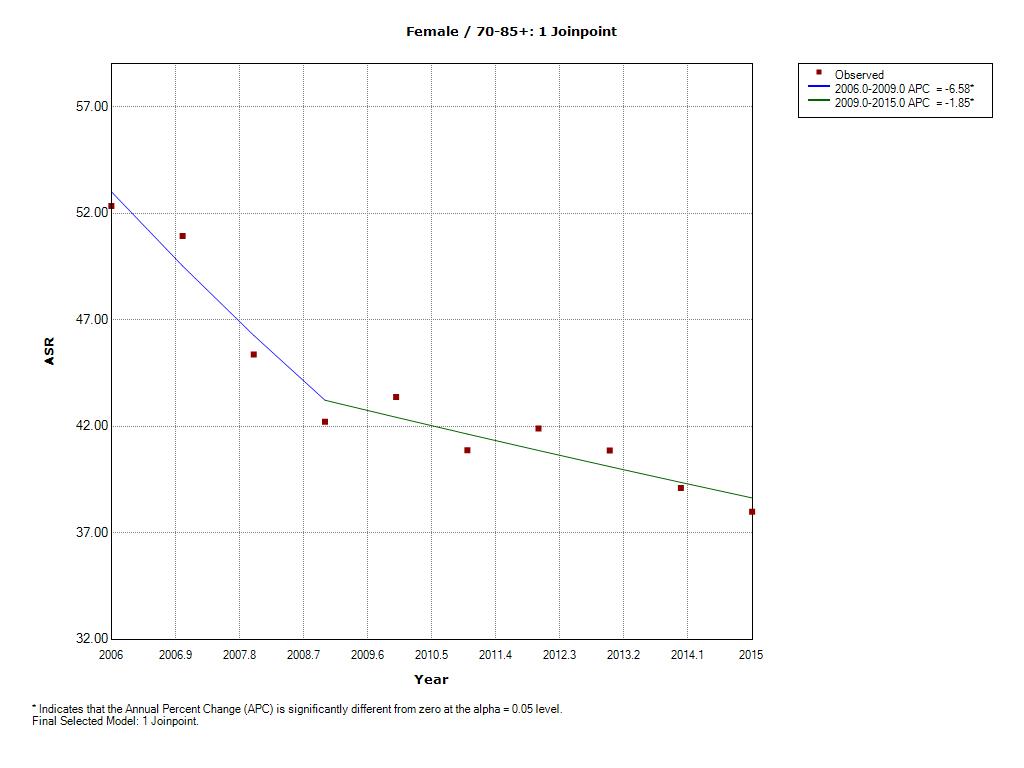

Supplement: Supplementary file 8 — Supplement Figure 8: mortality joinpoint. [file 12889_2024_19104_MOESM8_ESM.zip › Supplement Figure 8 mortality joinpoint/Brazil female 70-85+.jpg]

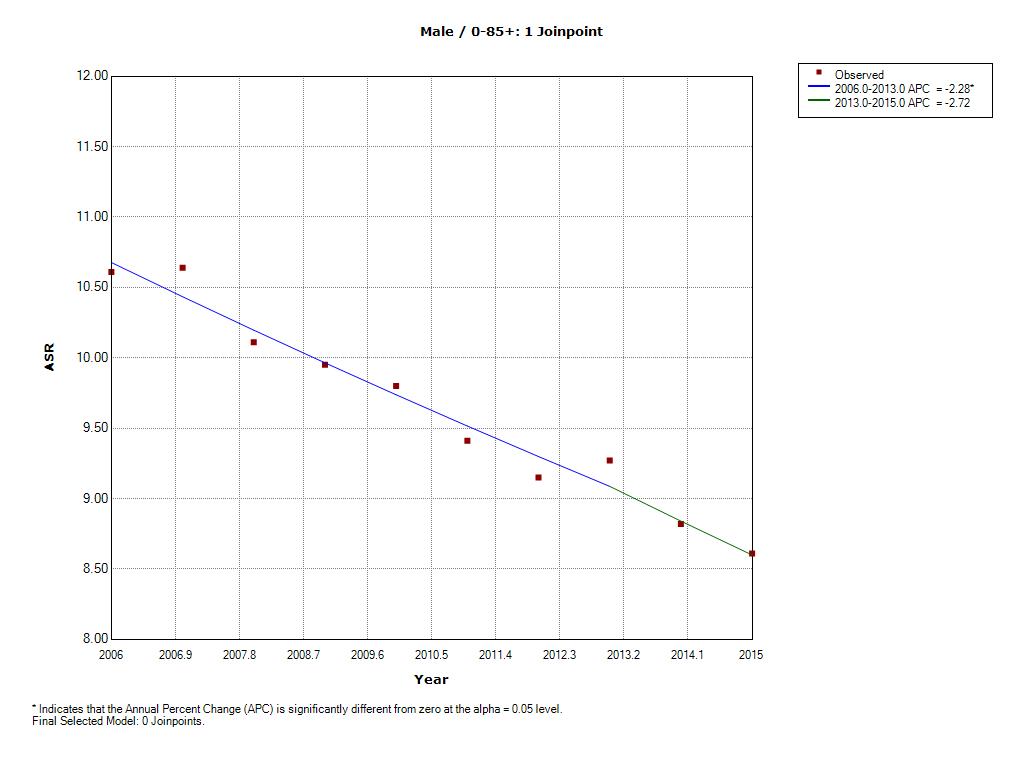

Supplement: Supplementary file 8 — Supplement Figure 8: mortality joinpoint. [file 12889_2024_19104_MOESM8_ESM.zip › Supplement Figure 8 mortality joinpoint/Brazil male 0-85+.jpg]

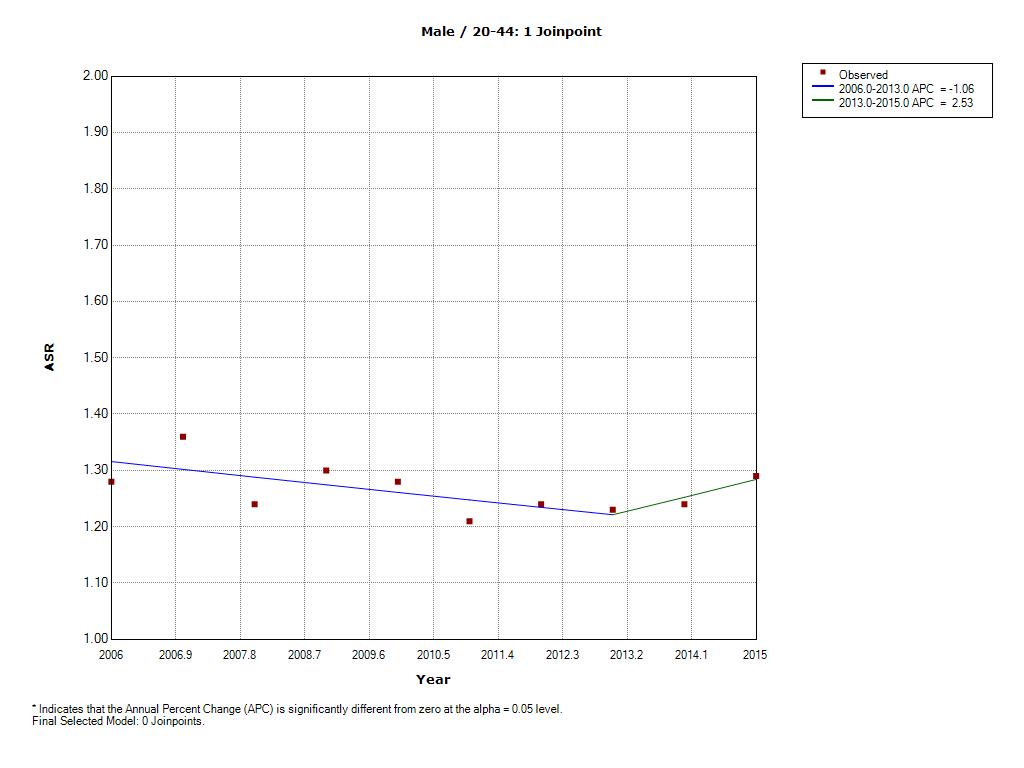

Supplement: Supplementary file 8 — Supplement Figure 8: mortality joinpoint. [file 12889_2024_19104_MOESM8_ESM.zip › Supplement Figure 8 mortality joinpoint/Brazil male 20-44.jpg]

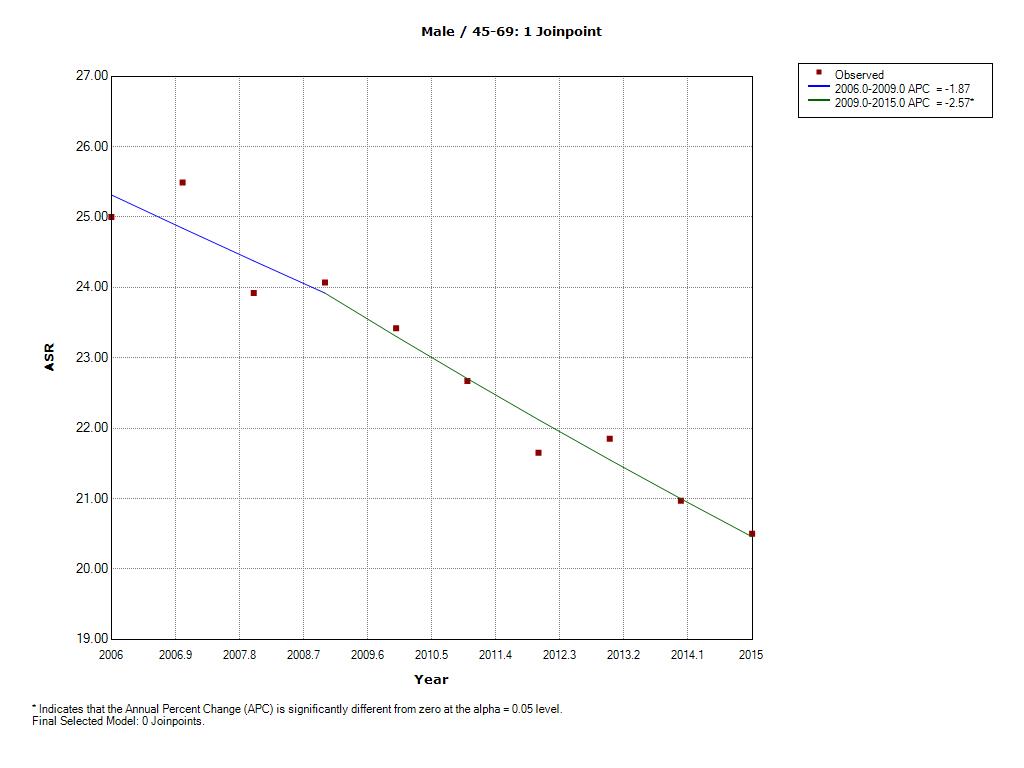

Supplement: Supplementary file 8 — Supplement Figure 8: mortality joinpoint. [file 12889_2024_19104_MOESM8_ESM.zip › Supplement Figure 8 mortality joinpoint/Brazil male 45-69.jpg]

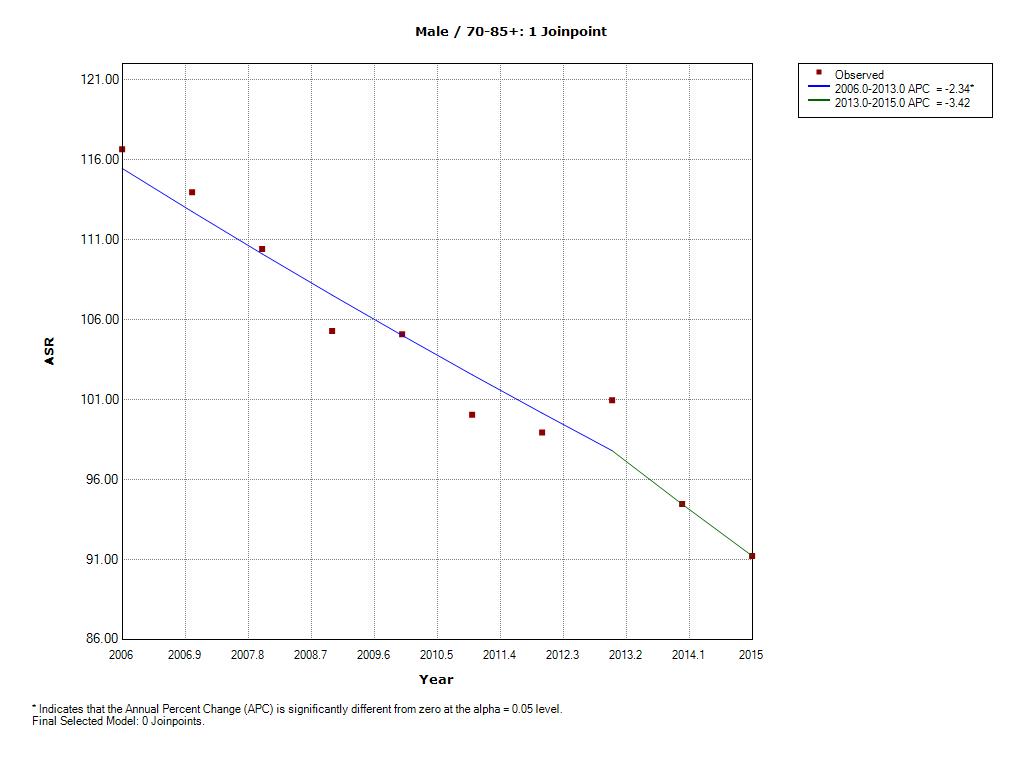

Supplement: Supplementary file 8 — Supplement Figure 8: mortality joinpoint. [file 12889_2024_19104_MOESM8_ESM.zip › Supplement Figure 8 mortality joinpoint/Brazil male 70-85+.jpg]

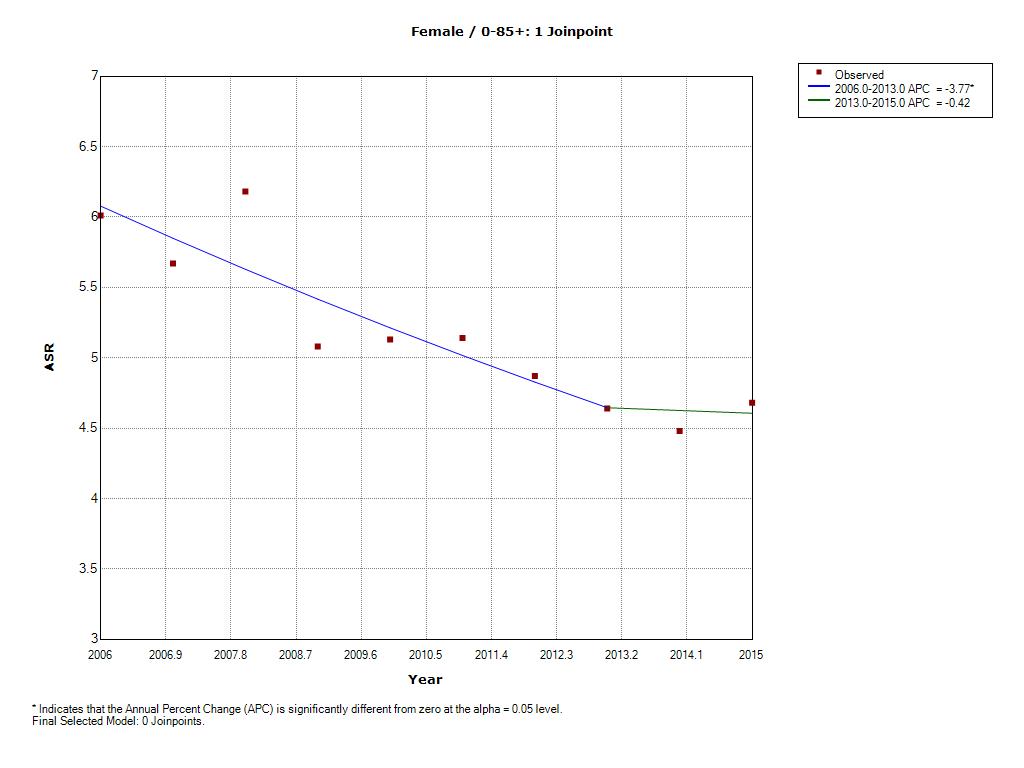

Supplement: Supplementary file 8 — Supplement Figure 8: mortality joinpoint. [file 12889_2024_19104_MOESM8_ESM.zip › Supplement Figure 8 mortality joinpoint/Bulgaria female 0-85+.jpg]

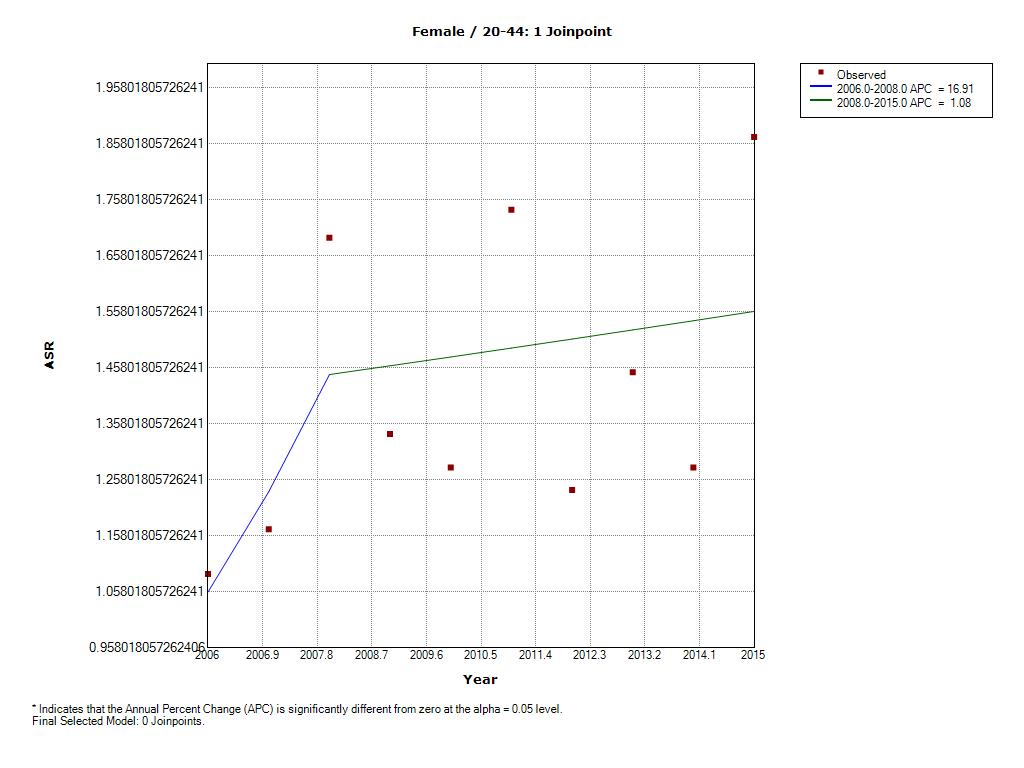

Supplement: Supplementary file 8 — Supplement Figure 8: mortality joinpoint. [file 12889_2024_19104_MOESM8_ESM.zip › Supplement Figure 8 mortality joinpoint/Bulgaria female 20-44.jpg]

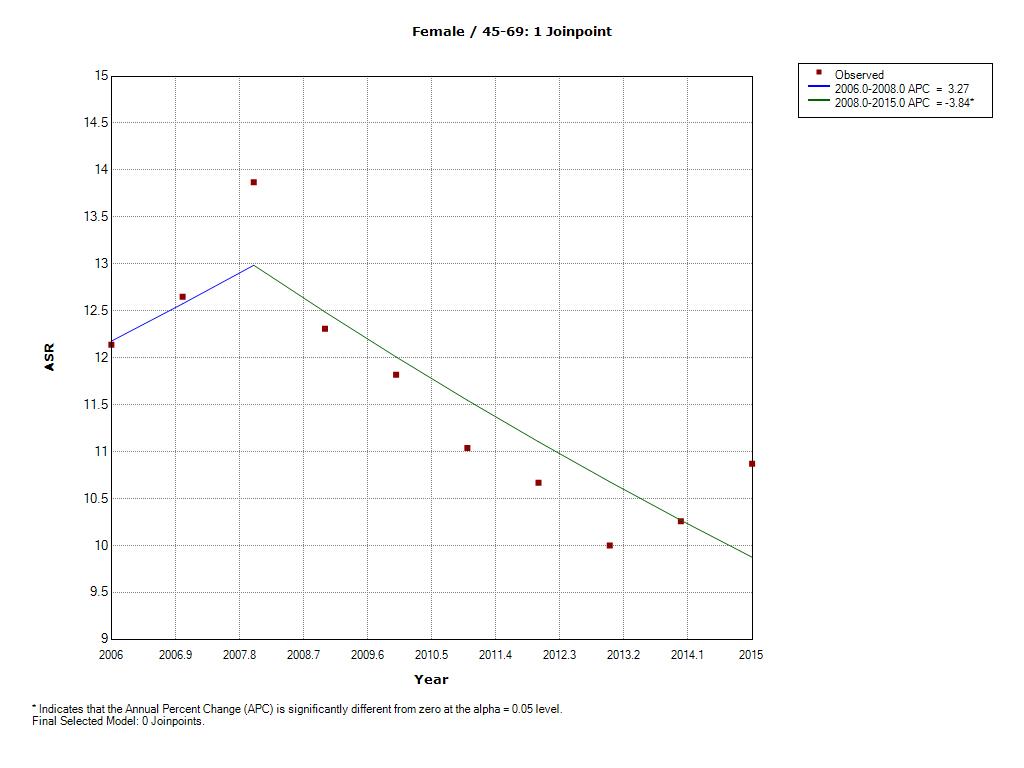

Supplement: Supplementary file 8 — Supplement Figure 8: mortality joinpoint. [file 12889_2024_19104_MOESM8_ESM.zip › Supplement Figure 8 mortality joinpoint/Bulgaria female 45-69.jpg]

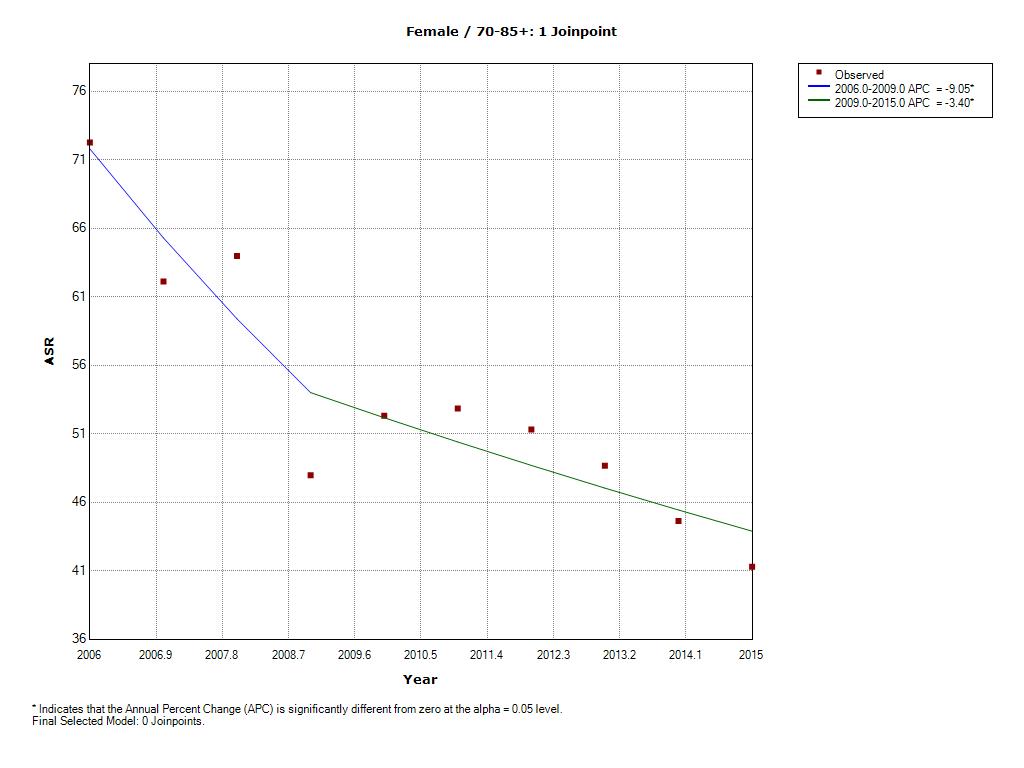

Supplement: Supplementary file 8 — Supplement Figure 8: mortality joinpoint. [file 12889_2024_19104_MOESM8_ESM.zip › Supplement Figure 8 mortality joinpoint/Bulgaria female 70-85+.jpg]

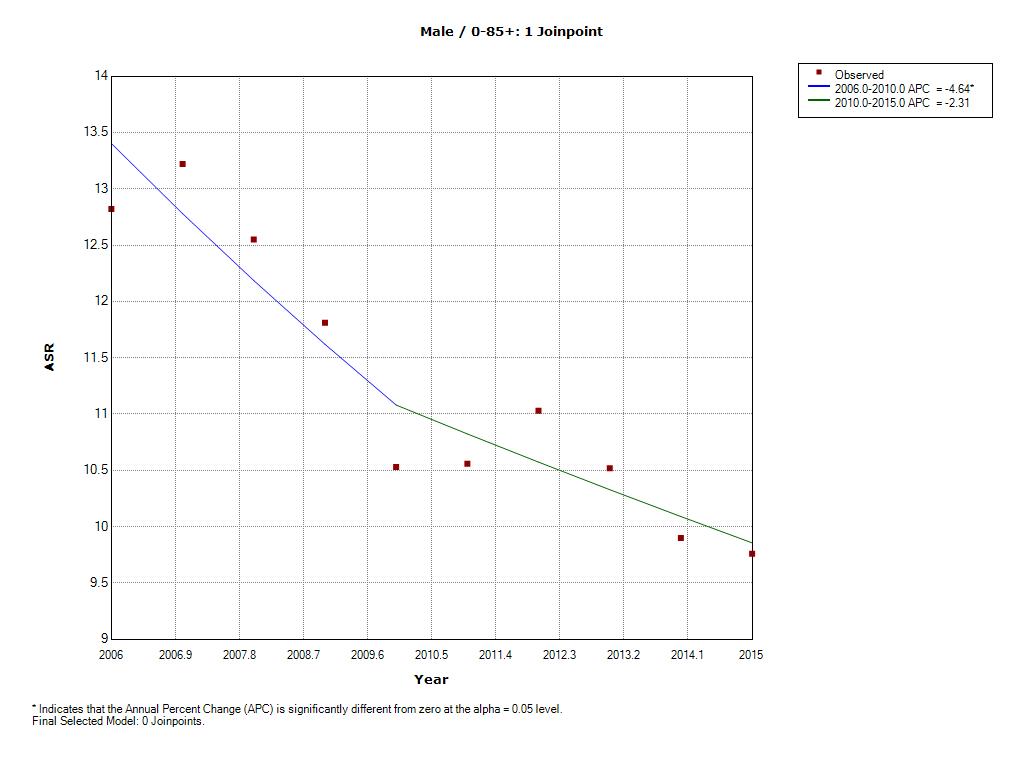

Supplement: Supplementary file 8 — Supplement Figure 8: mortality joinpoint. [file 12889_2024_19104_MOESM8_ESM.zip › Supplement Figure 8 mortality joinpoint/Bulgaria male 0-85+.jpg]

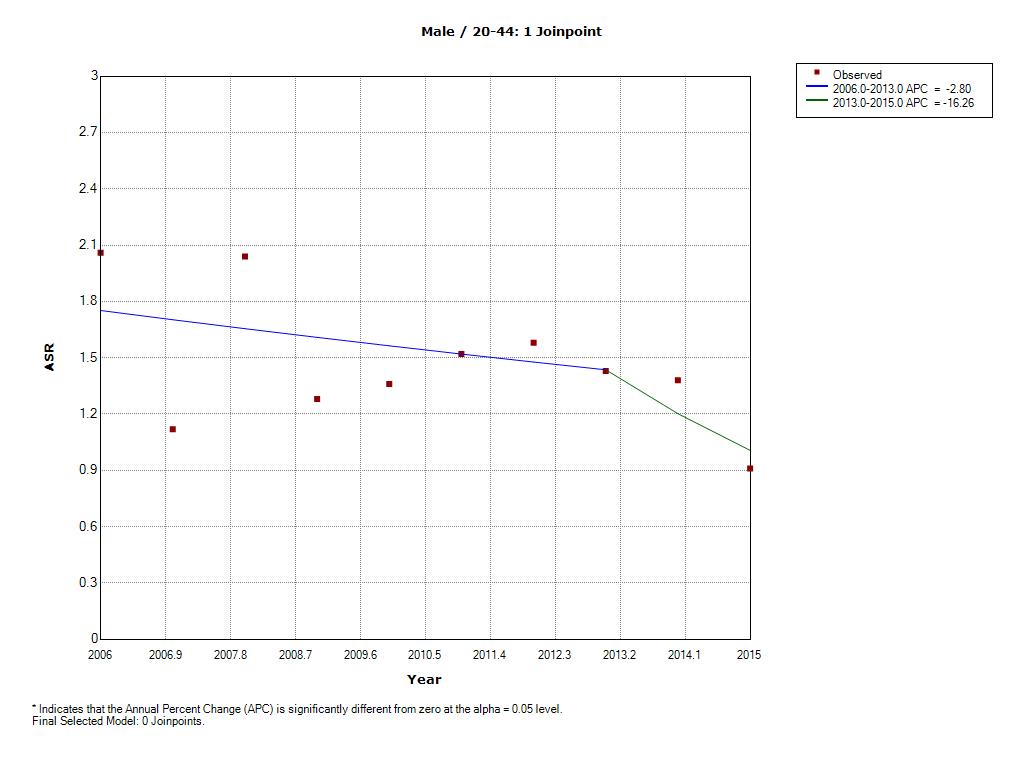

Supplement: Supplementary file 8 — Supplement Figure 8: mortality joinpoint. [file 12889_2024_19104_MOESM8_ESM.zip › Supplement Figure 8 mortality joinpoint/Bulgaria male 20-44.jpg]

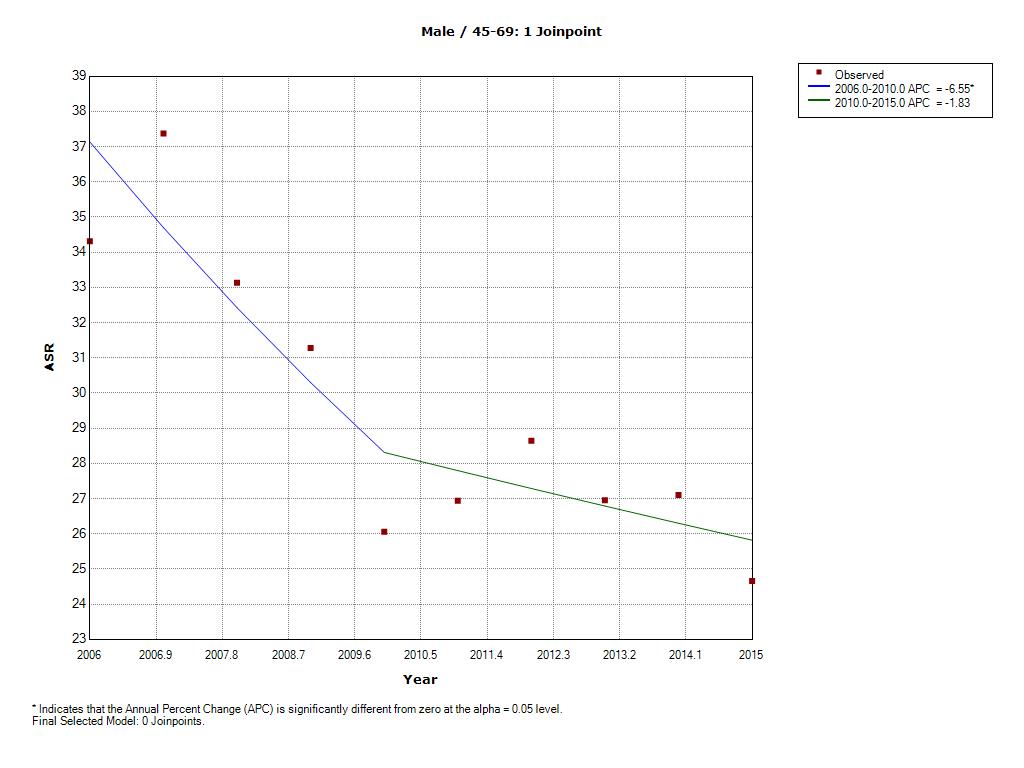

Supplement: Supplementary file 8 — Supplement Figure 8: mortality joinpoint. [file 12889_2024_19104_MOESM8_ESM.zip › Supplement Figure 8 mortality joinpoint/Bulgaria male 45-69.jpg]

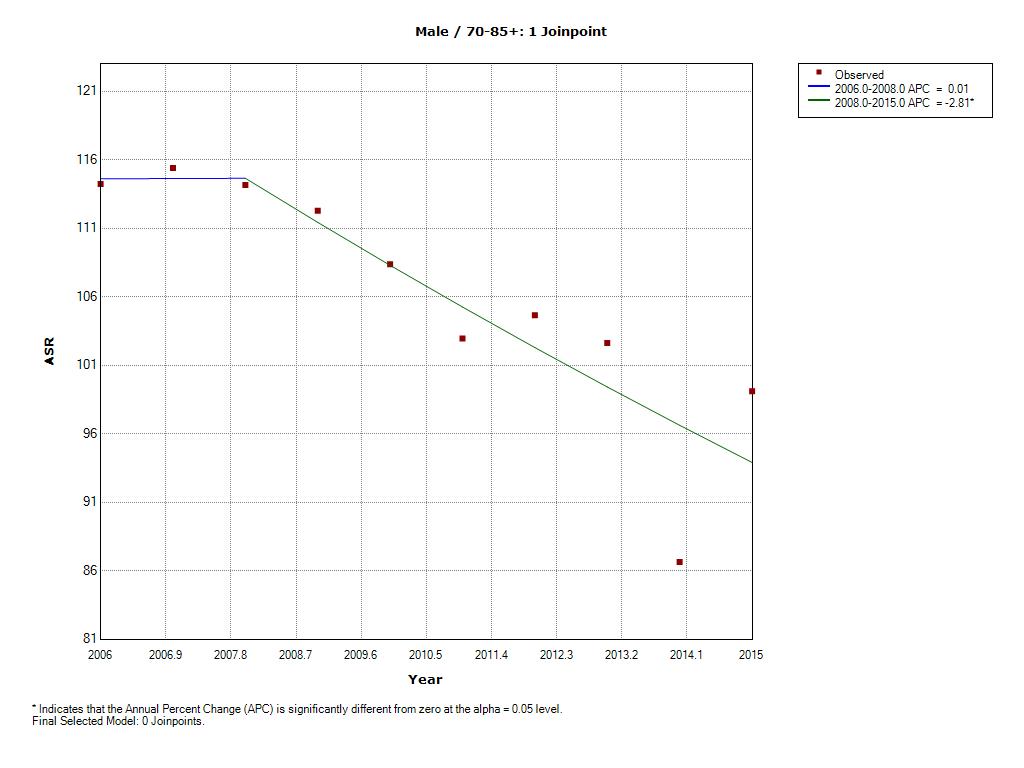

Supplement: Supplementary file 8 — Supplement Figure 8: mortality joinpoint. [file 12889_2024_19104_MOESM8_ESM.zip › Supplement Figure 8 mortality joinpoint/Bulgaria male 70-85+.jpg]

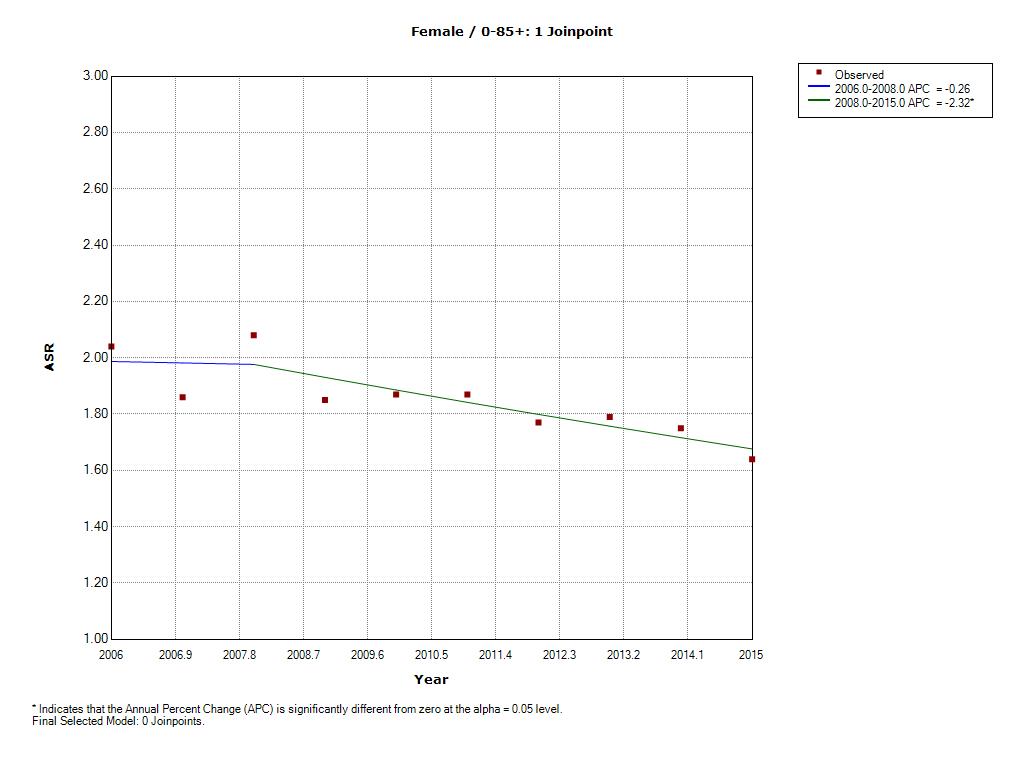

Supplement: Supplementary file 8 — Supplement Figure 8: mortality joinpoint. [file 12889_2024_19104_MOESM8_ESM.zip › Supplement Figure 8 mortality joinpoint/Canada female 0-85+.jpg]

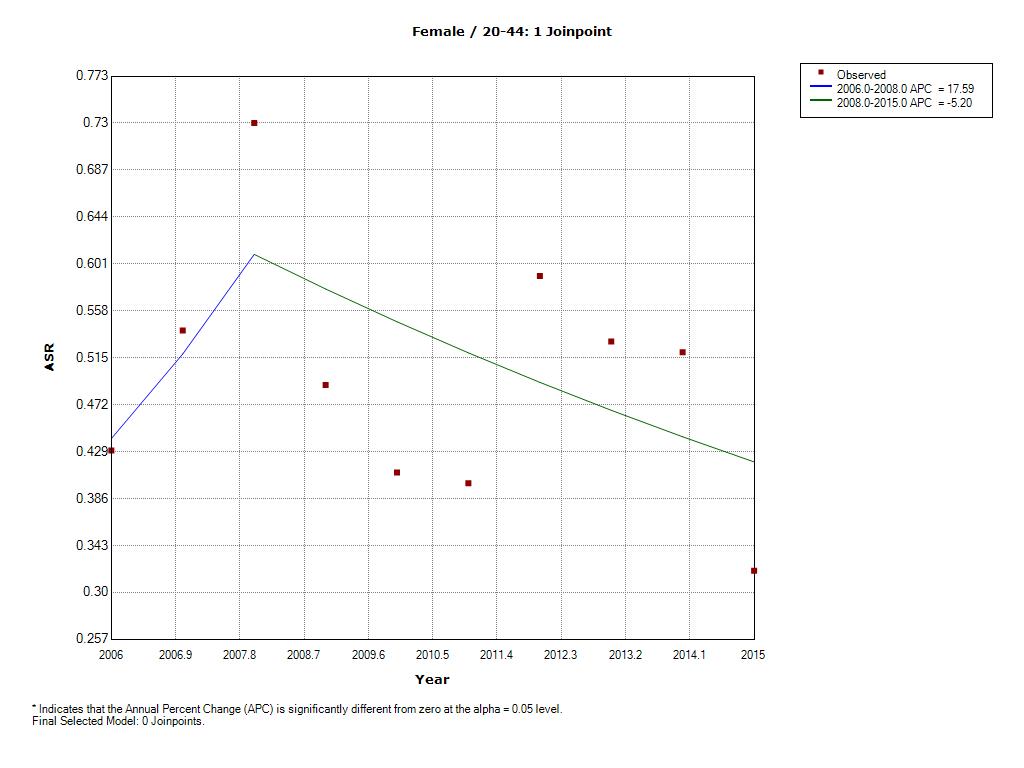

Supplement: Supplementary file 8 — Supplement Figure 8: mortality joinpoint. [file 12889_2024_19104_MOESM8_ESM.zip › Supplement Figure 8 mortality joinpoint/Canada female 20-44.jpg]

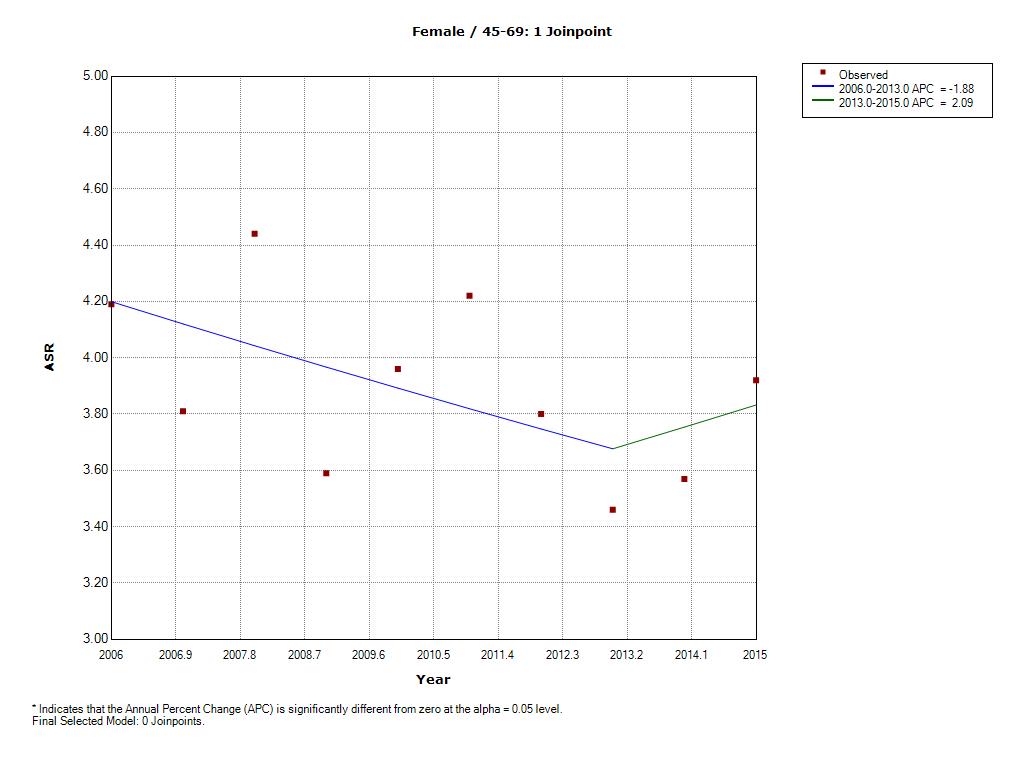

Supplement: Supplementary file 8 — Supplement Figure 8: mortality joinpoint. [file 12889_2024_19104_MOESM8_ESM.zip › Supplement Figure 8 mortality joinpoint/Canada female 45-69.jpg]

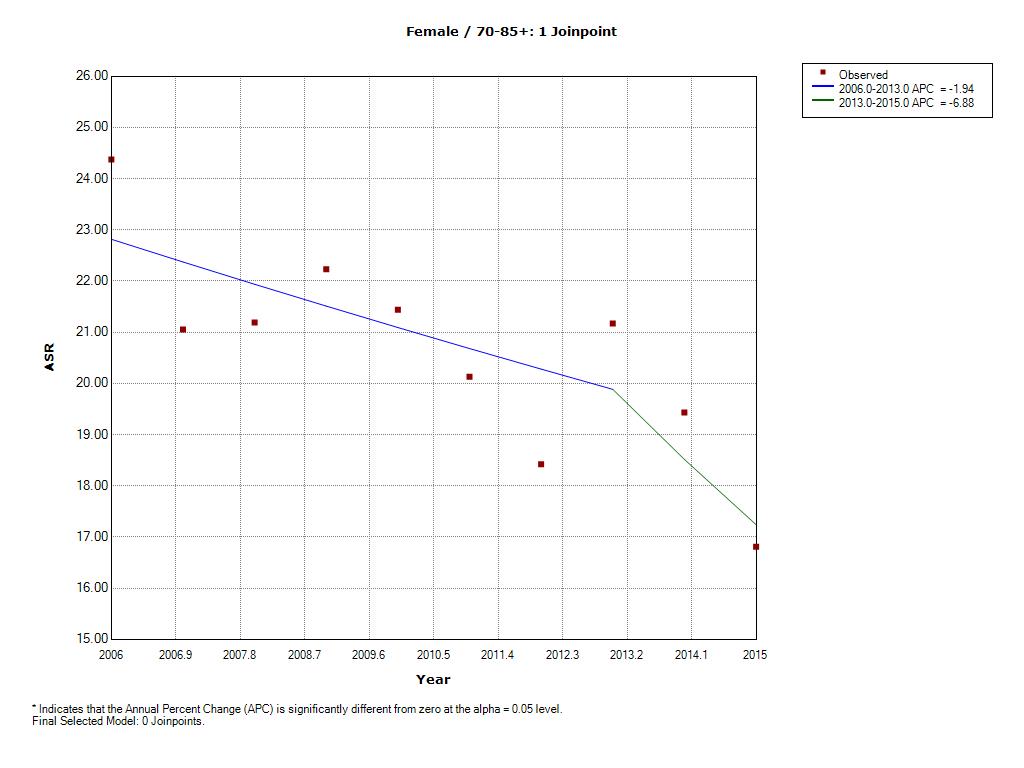

Supplement: Supplementary file 8 — Supplement Figure 8: mortality joinpoint. [file 12889_2024_19104_MOESM8_ESM.zip › Supplement Figure 8 mortality joinpoint/Canada female 70-85+.jpg]

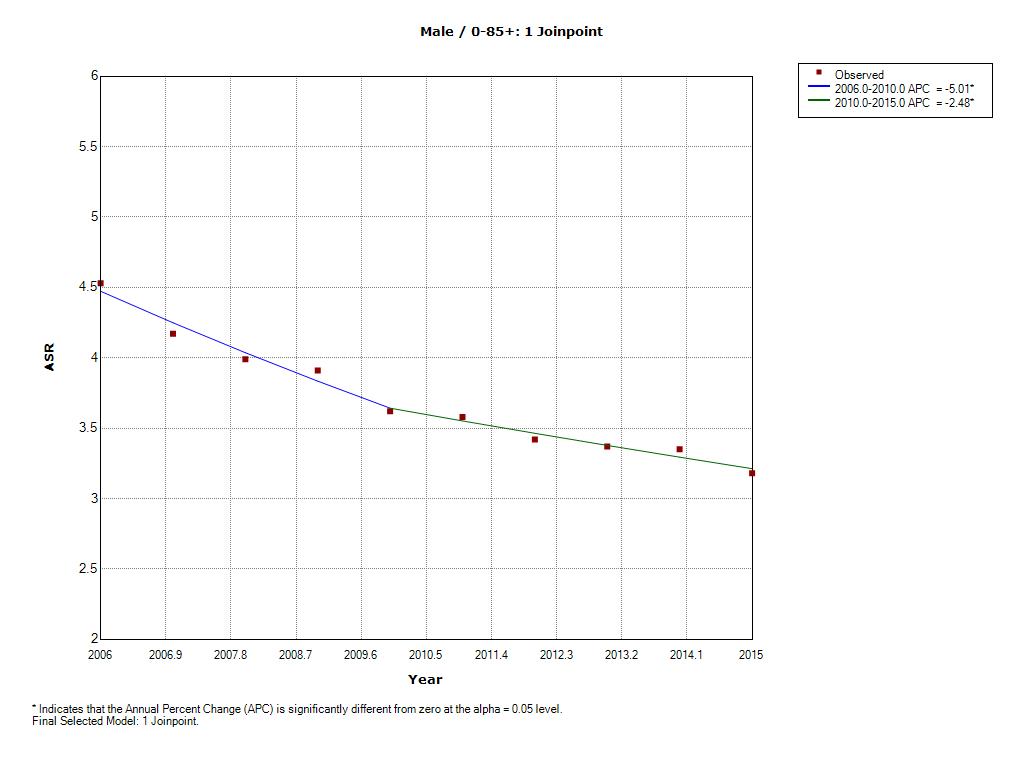

Supplement: Supplementary file 8 — Supplement Figure 8: mortality joinpoint. [file 12889_2024_19104_MOESM8_ESM.zip › Supplement Figure 8 mortality joinpoint/Canada male 0-85+.jpg]

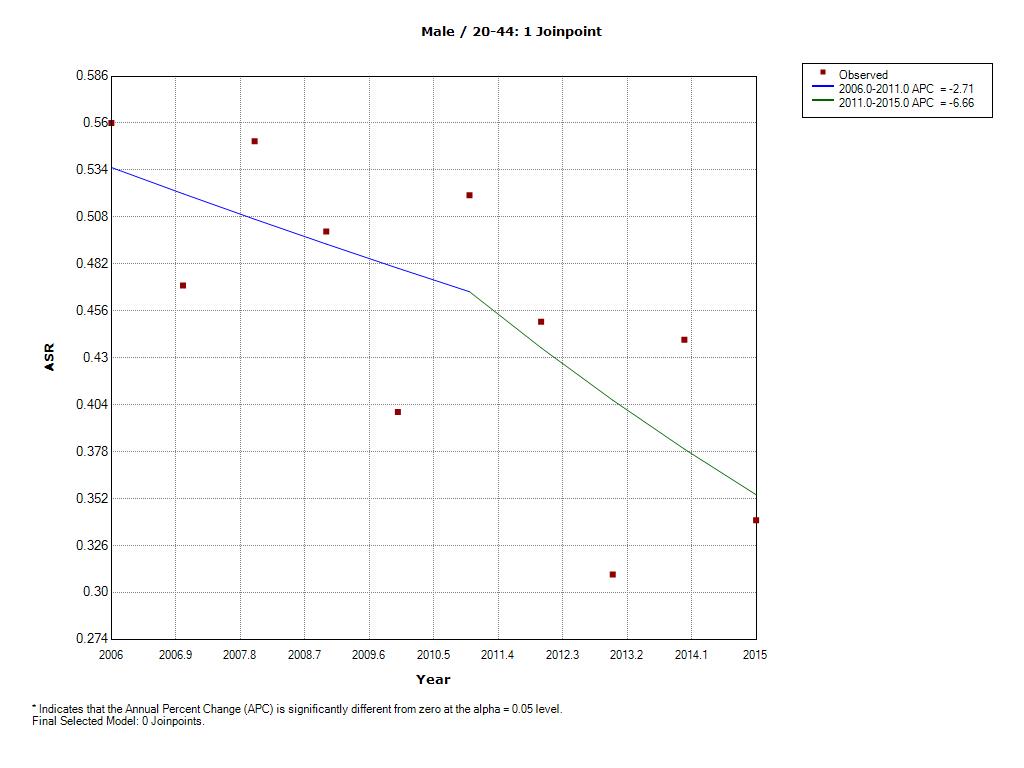

Supplement: Supplementary file 8 — Supplement Figure 8: mortality joinpoint. [file 12889_2024_19104_MOESM8_ESM.zip › Supplement Figure 8 mortality joinpoint/Canada male 20-44.jpg]

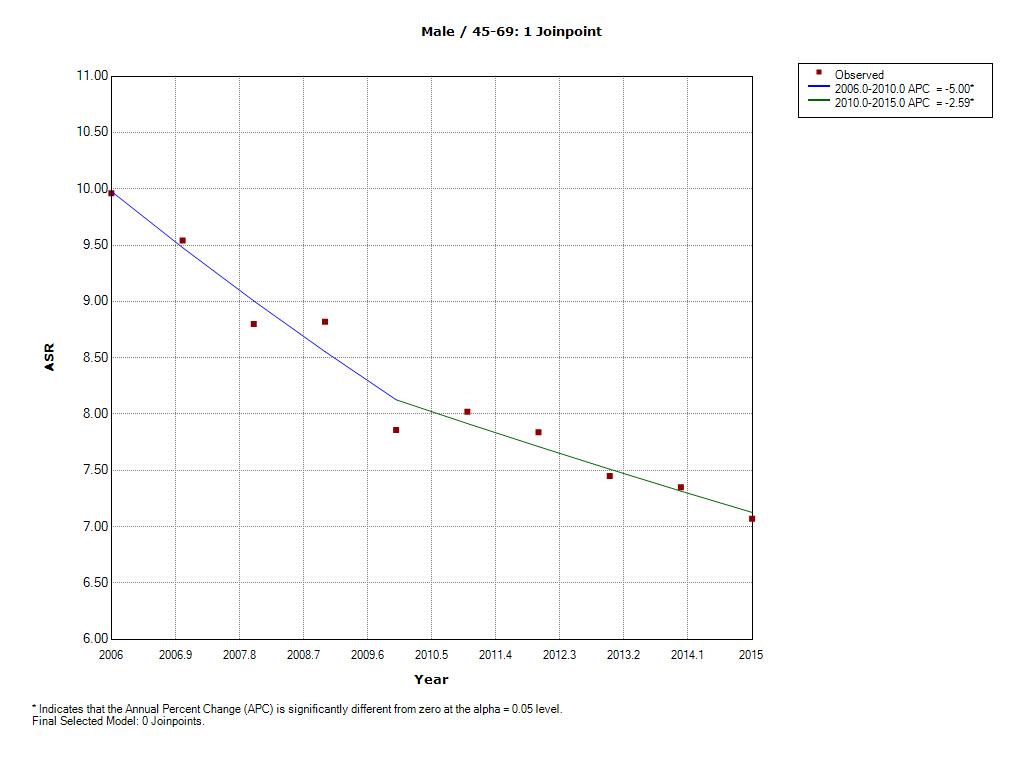

Supplement: Supplementary file 8 — Supplement Figure 8: mortality joinpoint. [file 12889_2024_19104_MOESM8_ESM.zip › Supplement Figure 8 mortality joinpoint/Canada male 45-69.jpg]

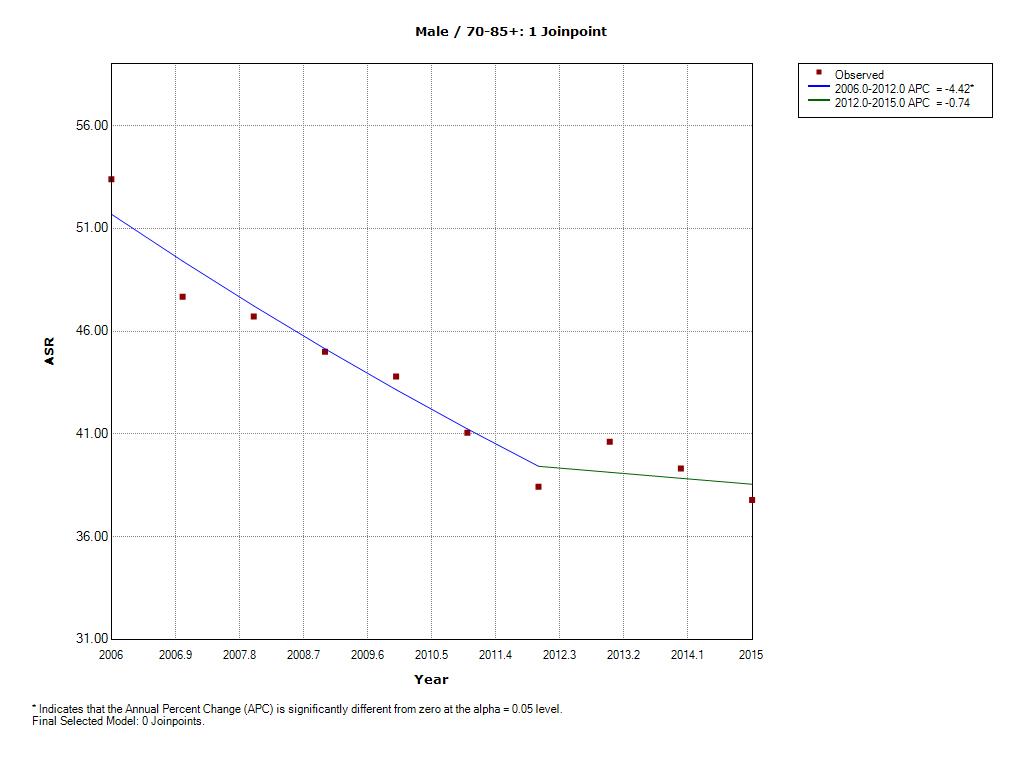

Supplement: Supplementary file 8 — Supplement Figure 8: mortality joinpoint. [file 12889_2024_19104_MOESM8_ESM.zip › Supplement Figure 8 mortality joinpoint/Canada male 70-85+.jpg]

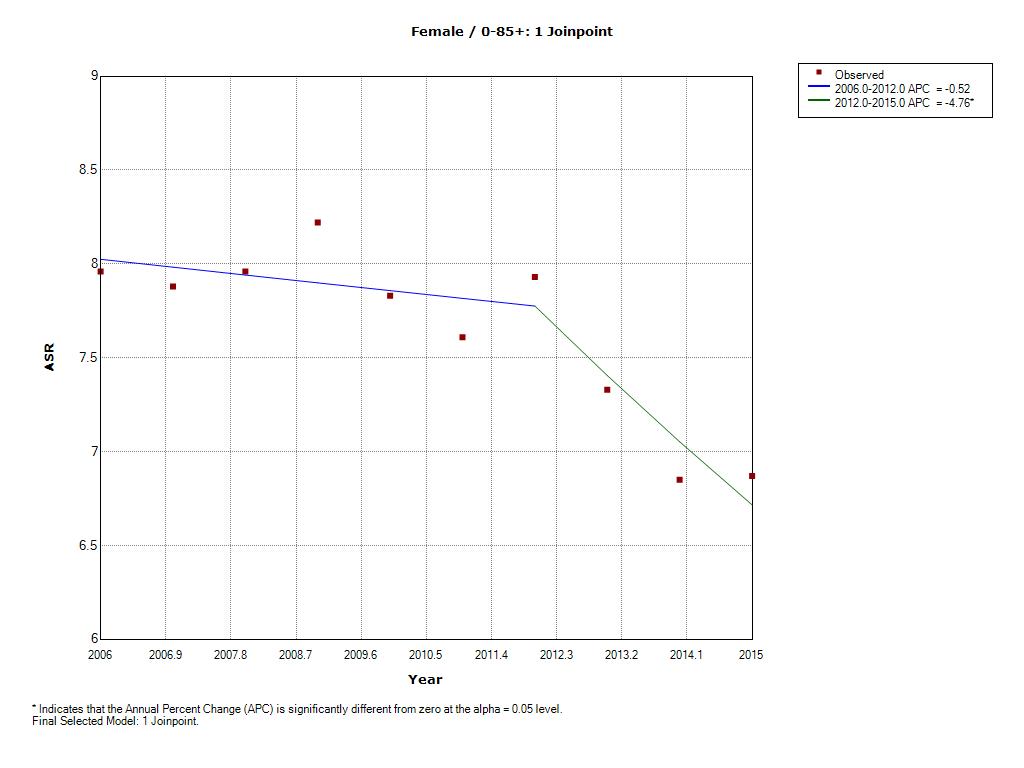

Supplement: Supplementary file 8 — Supplement Figure 8: mortality joinpoint. [file 12889_2024_19104_MOESM8_ESM.zip › Supplement Figure 8 mortality joinpoint/Chile female 0-85+.jpg]

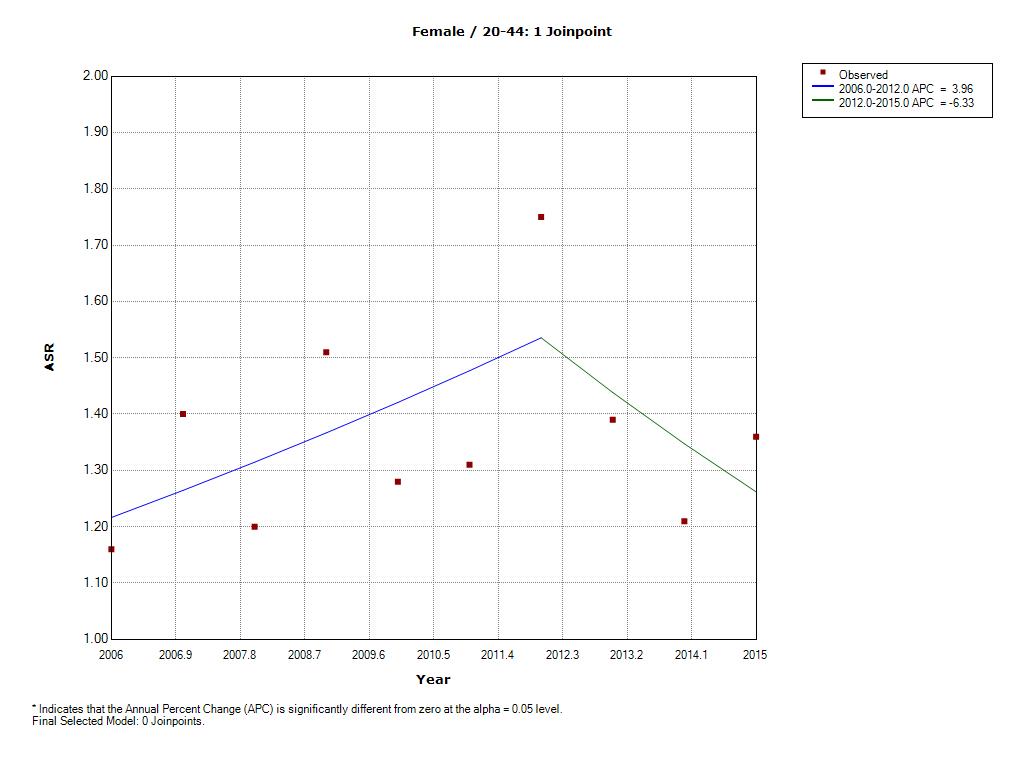

Supplement: Supplementary file 8 — Supplement Figure 8: mortality joinpoint. [file 12889_2024_19104_MOESM8_ESM.zip › Supplement Figure 8 mortality joinpoint/Chile female 20-44.jpg]

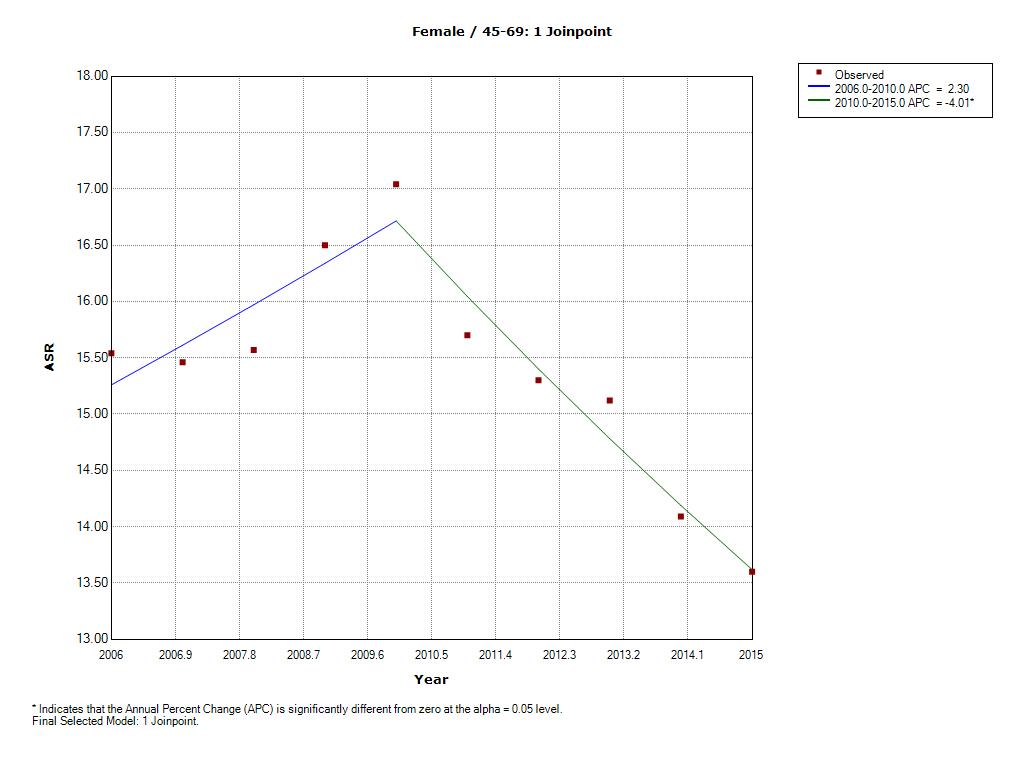

Supplement: Supplementary file 8 — Supplement Figure 8: mortality joinpoint. [file 12889_2024_19104_MOESM8_ESM.zip › Supplement Figure 8 mortality joinpoint/Chile female 45-69.jpg]

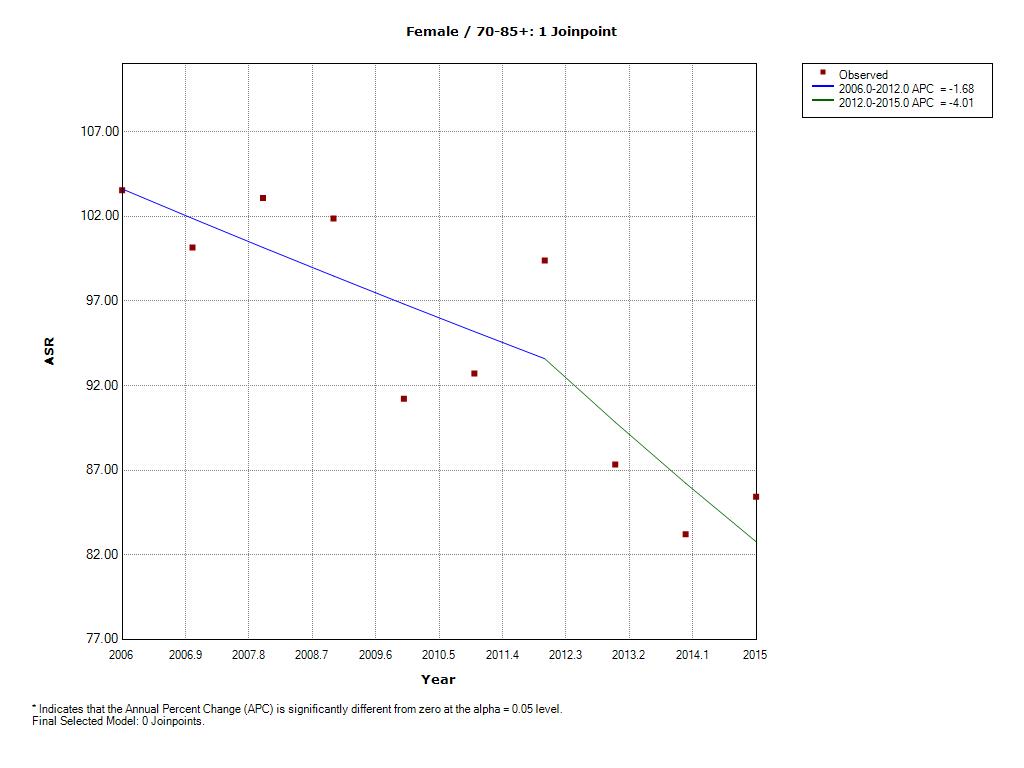

Supplement: Supplementary file 8 — Supplement Figure 8: mortality joinpoint. [file 12889_2024_19104_MOESM8_ESM.zip › Supplement Figure 8 mortality joinpoint/Chile female 70-85+.jpg]

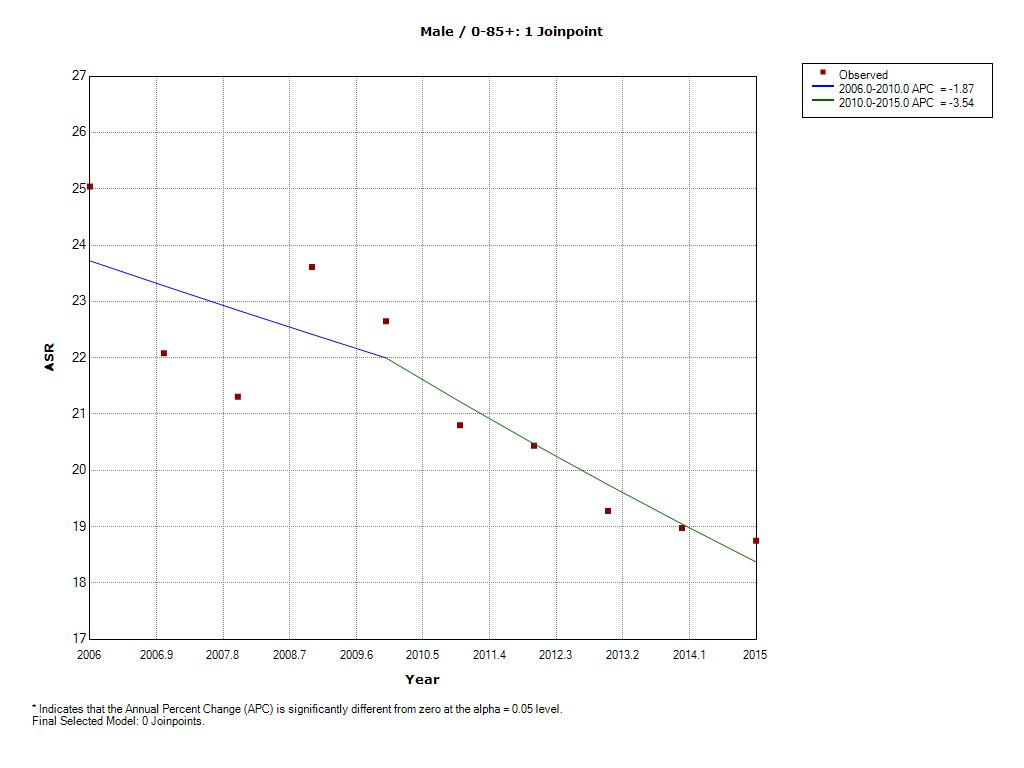

Supplement: Supplementary file 8 — Supplement Figure 8: mortality joinpoint. [file 12889_2024_19104_MOESM8_ESM.zip › Supplement Figure 8 mortality joinpoint/Chile male 0-85+.jpg]

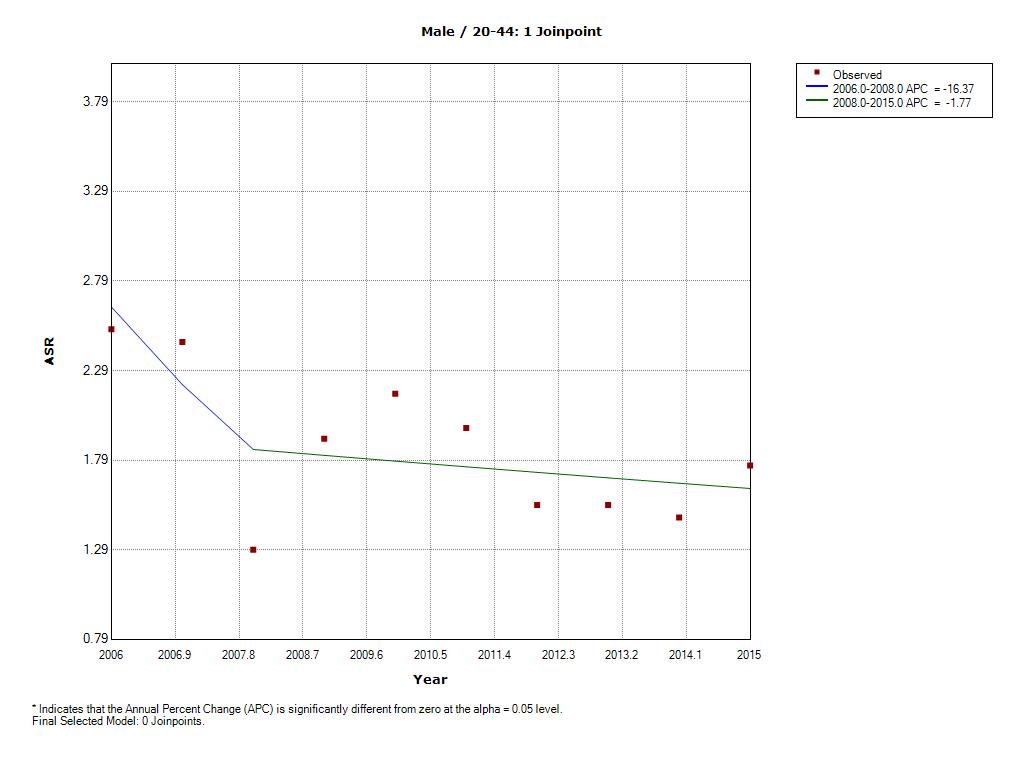

Supplement: Supplementary file 8 — Supplement Figure 8: mortality joinpoint. [file 12889_2024_19104_MOESM8_ESM.zip › Supplement Figure 8 mortality joinpoint/Chile male 20-44.jpg]

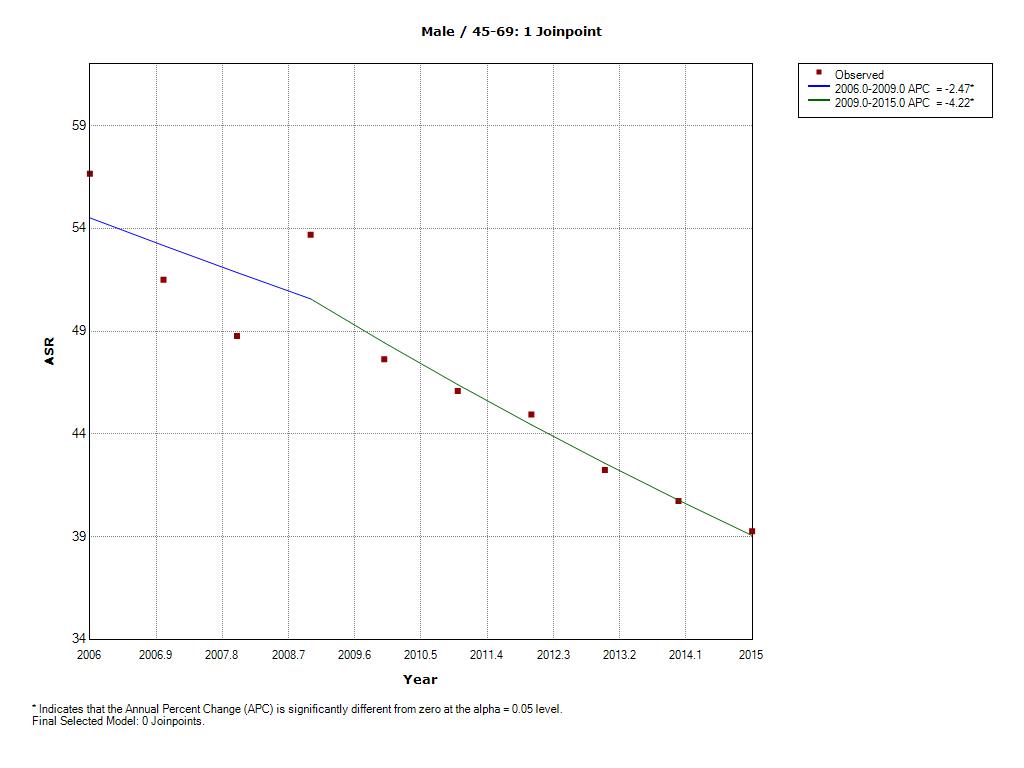

Supplement: Supplementary file 8 — Supplement Figure 8: mortality joinpoint. [file 12889_2024_19104_MOESM8_ESM.zip › Supplement Figure 8 mortality joinpoint/Chile male 45-69.jpg]

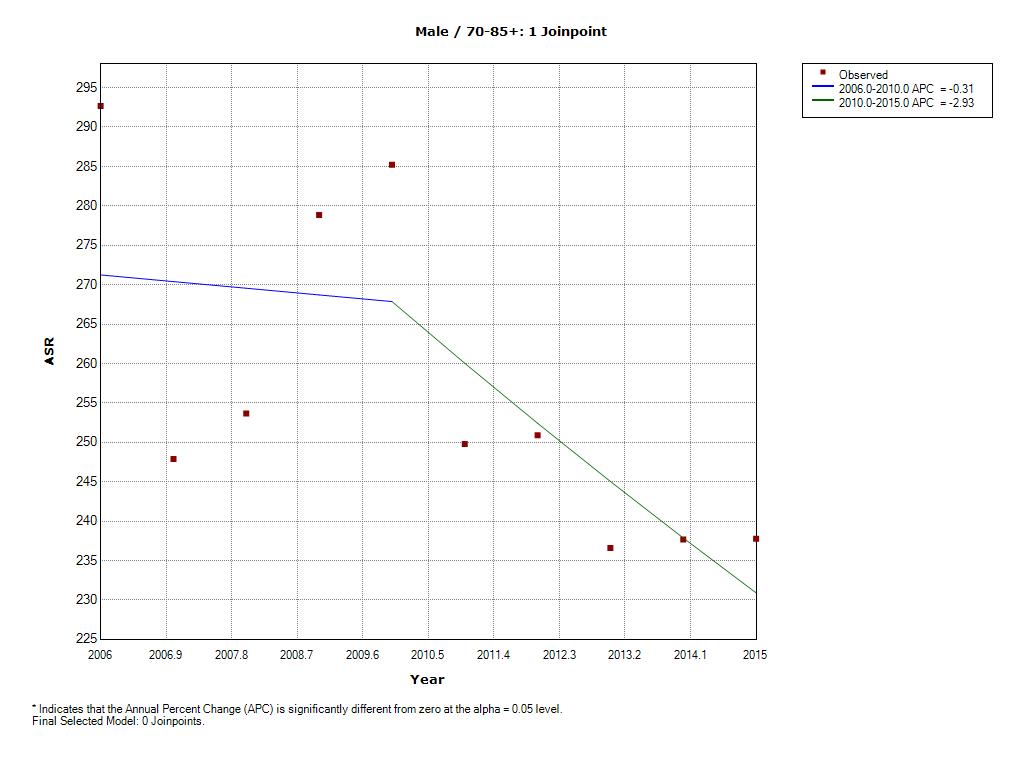

Supplement: Supplementary file 8 — Supplement Figure 8: mortality joinpoint. [file 12889_2024_19104_MOESM8_ESM.zip › Supplement Figure 8 mortality joinpoint/Chile male 70-85+.jpg]

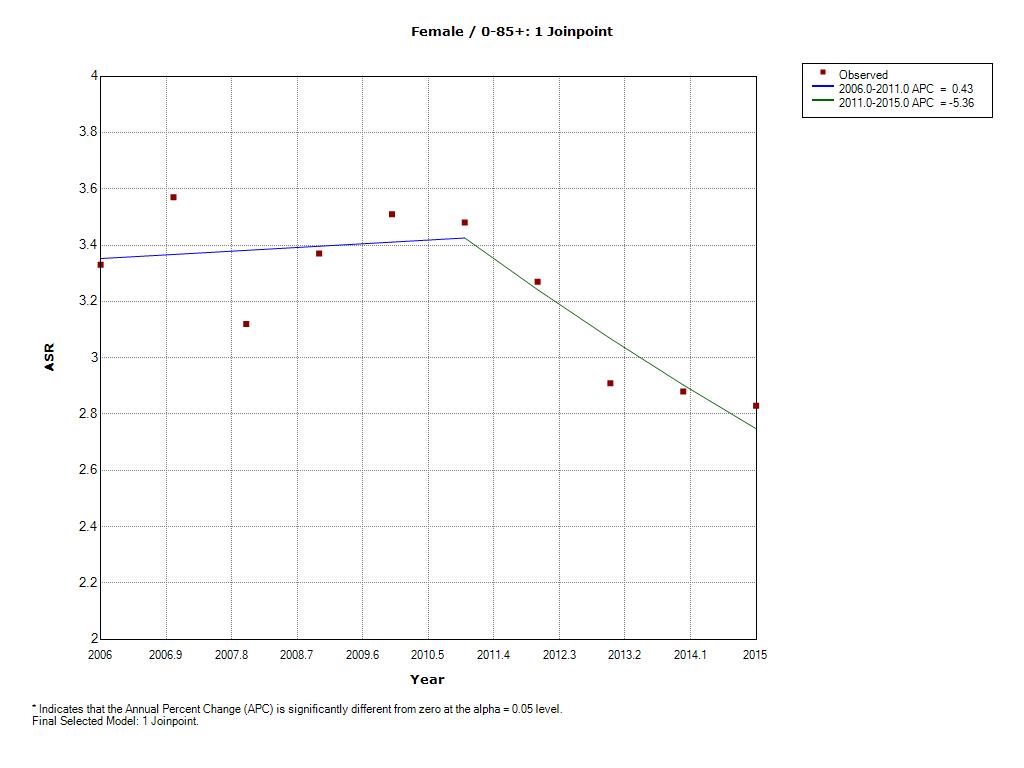

Supplement: Supplementary file 8 — Supplement Figure 8: mortality joinpoint. [file 12889_2024_19104_MOESM8_ESM.zip › Supplement Figure 8 mortality joinpoint/China female 0-85+.jpg]

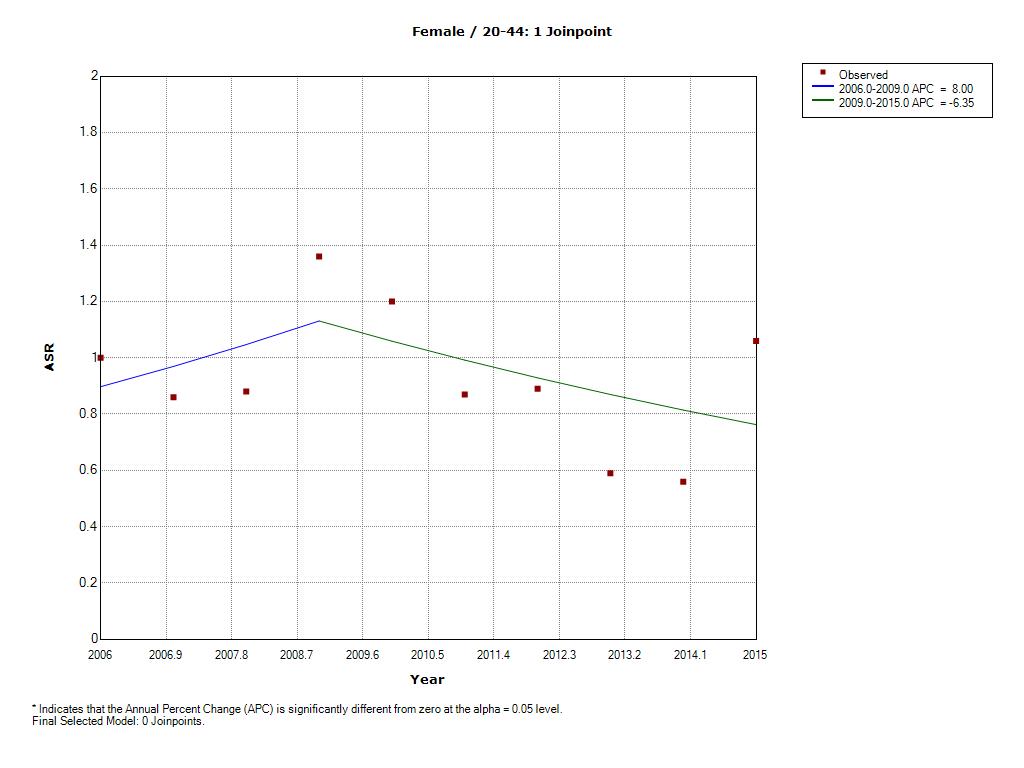

Supplement: Supplementary file 8 — Supplement Figure 8: mortality joinpoint. [file 12889_2024_19104_MOESM8_ESM.zip › Supplement Figure 8 mortality joinpoint/China female 20-44.jpg]

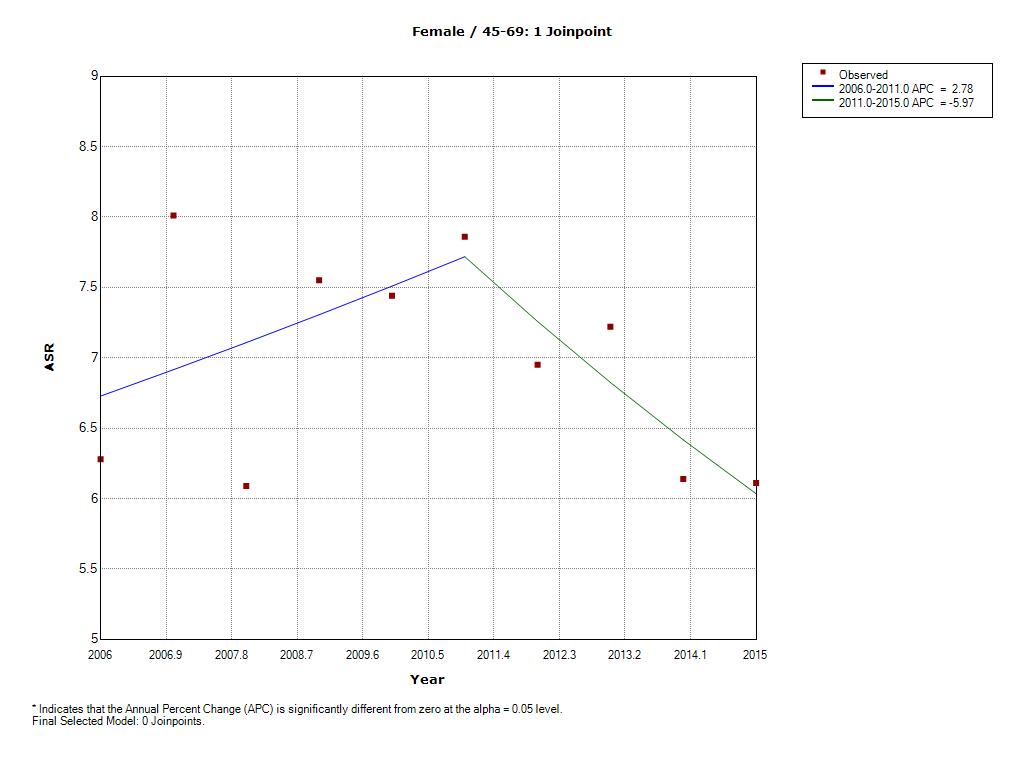

Supplement: Supplementary file 8 — Supplement Figure 8: mortality joinpoint. [file 12889_2024_19104_MOESM8_ESM.zip › Supplement Figure 8 mortality joinpoint/China female 45-69.jpg]

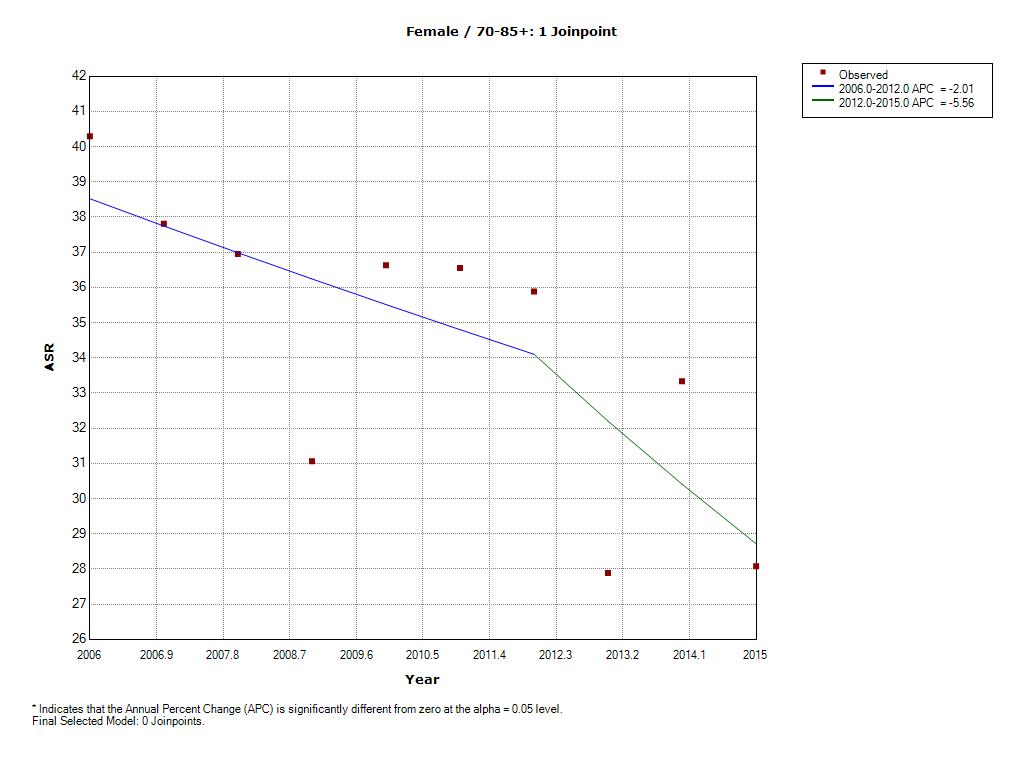

Supplement: Supplementary file 8 — Supplement Figure 8: mortality joinpoint. [file 12889_2024_19104_MOESM8_ESM.zip › Supplement Figure 8 mortality joinpoint/China female 70-85+.jpg]

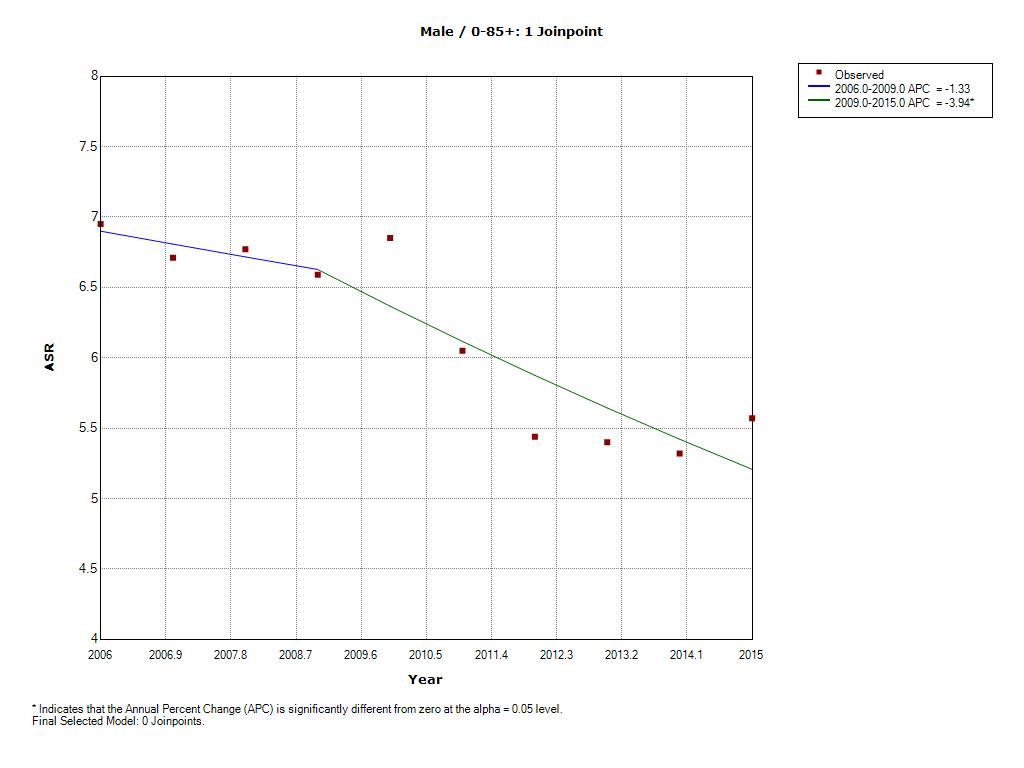

Supplement: Supplementary file 8 — Supplement Figure 8: mortality joinpoint. [file 12889_2024_19104_MOESM8_ESM.zip › Supplement Figure 8 mortality joinpoint/China male 0-85+.jpg]

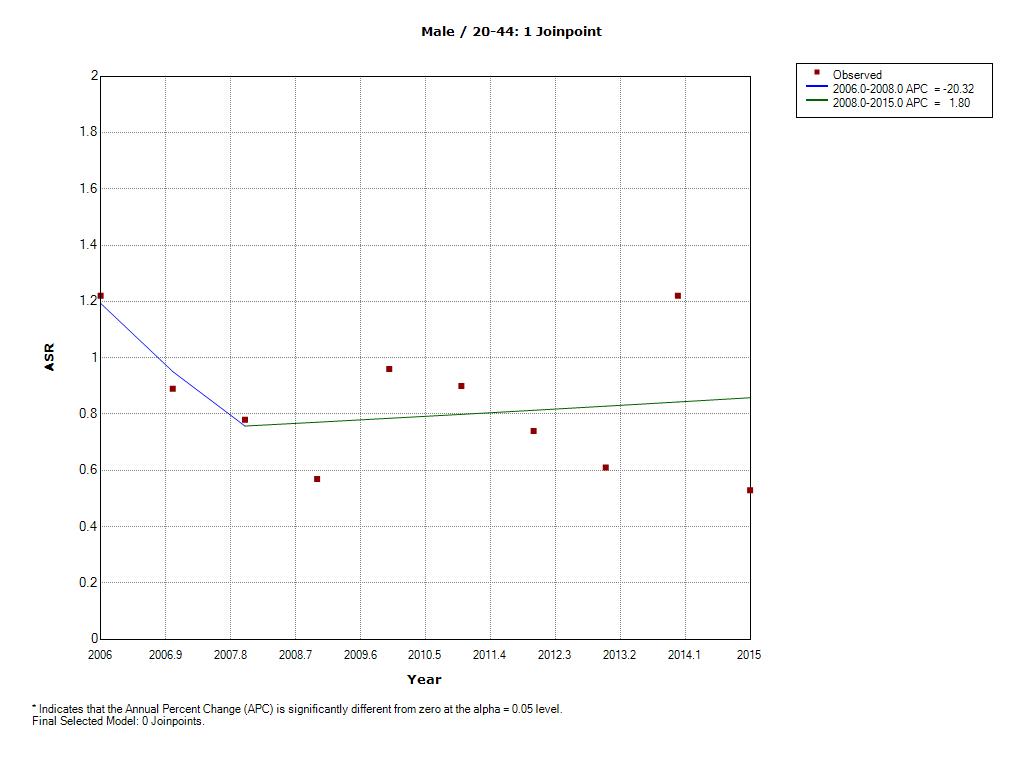

Supplement: Supplementary file 8 — Supplement Figure 8: mortality joinpoint. [file 12889_2024_19104_MOESM8_ESM.zip › Supplement Figure 8 mortality joinpoint/China male 20-44.jpg]

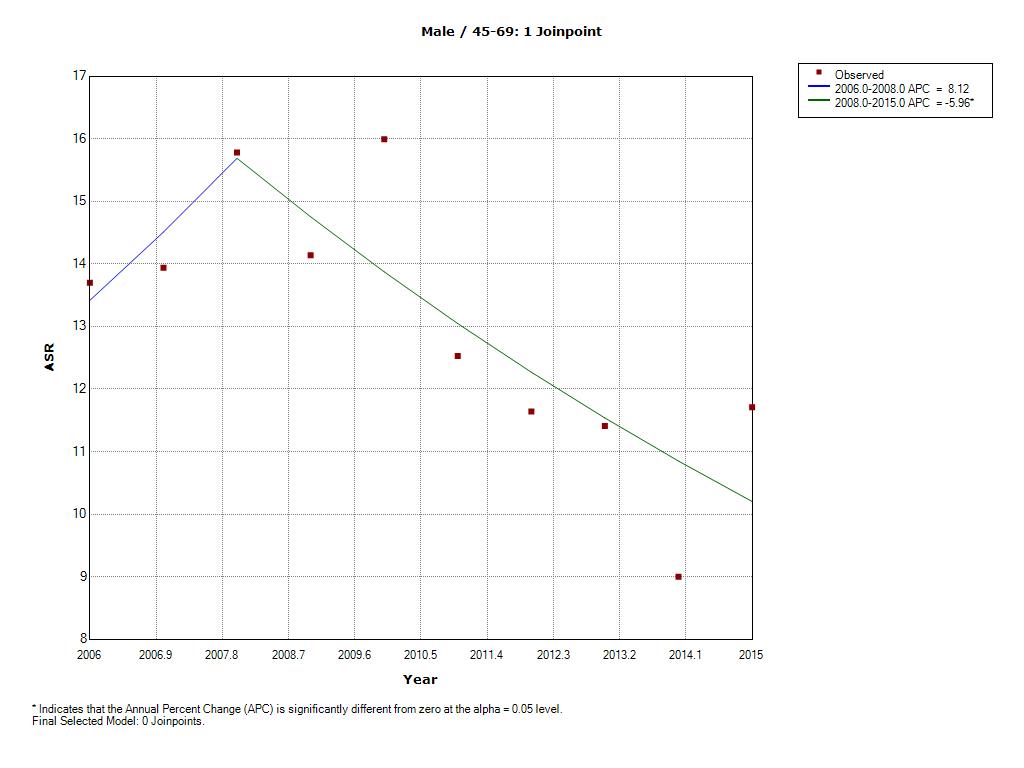

Supplement: Supplementary file 8 — Supplement Figure 8: mortality joinpoint. [file 12889_2024_19104_MOESM8_ESM.zip › Supplement Figure 8 mortality joinpoint/China male 45-69.jpg]

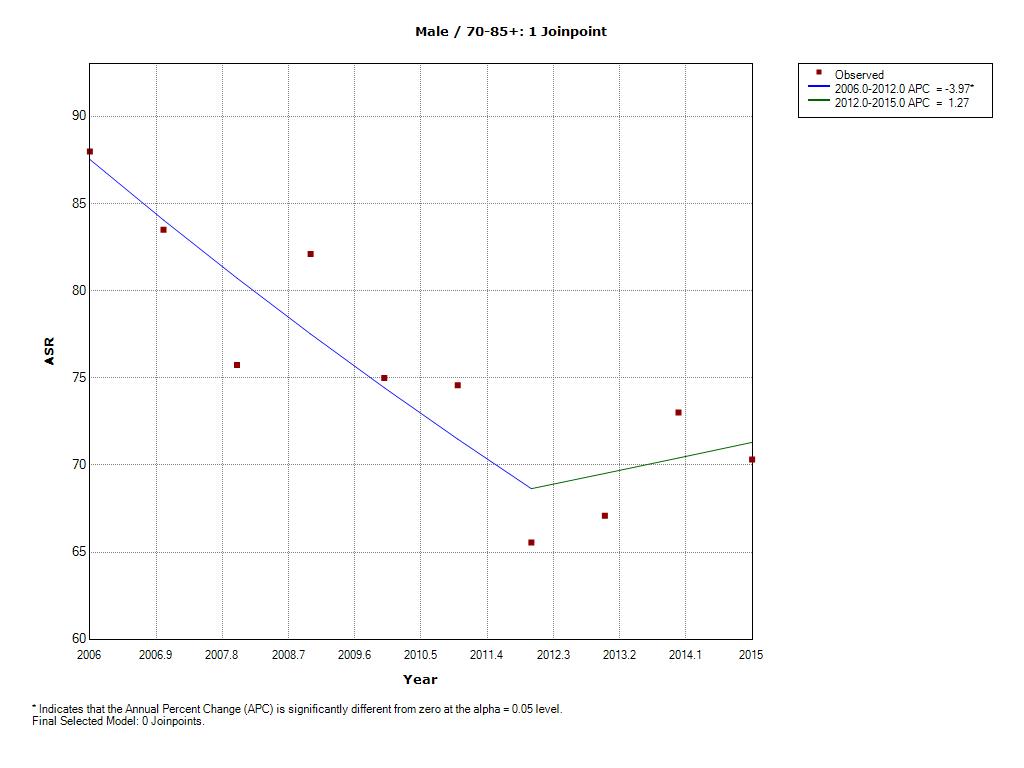

Supplement: Supplementary file 8 — Supplement Figure 8: mortality joinpoint. [file 12889_2024_19104_MOESM8_ESM.zip › Supplement Figure 8 mortality joinpoint/China male 70-85+.jpg]

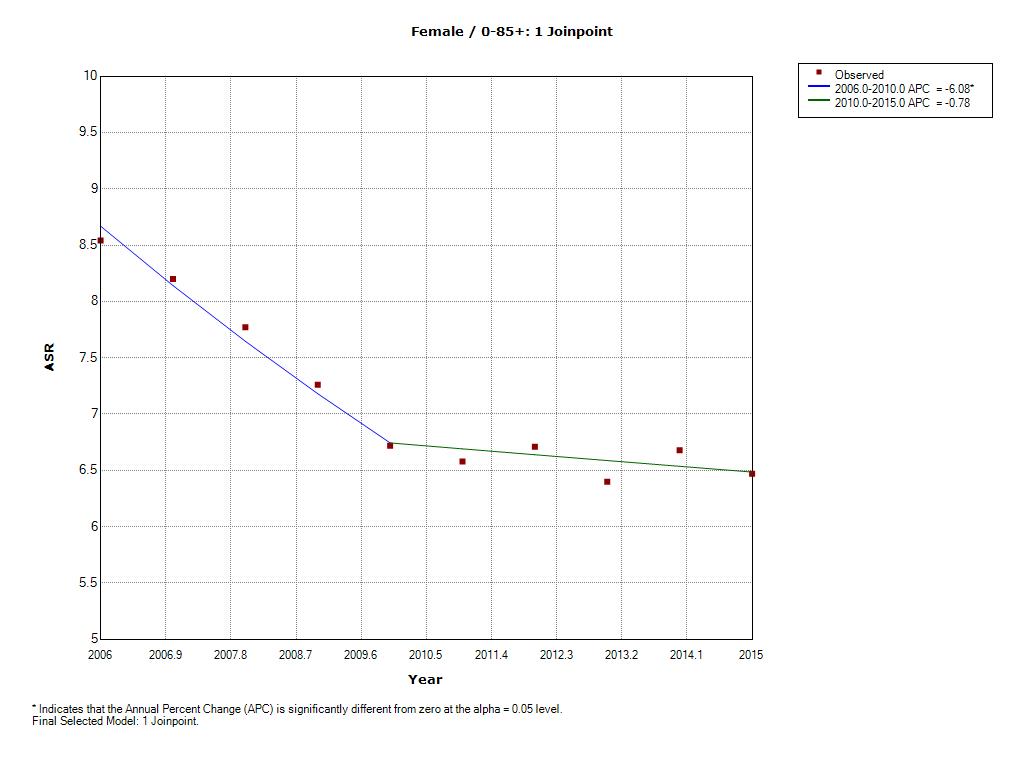

Supplement: Supplementary file 8 — Supplement Figure 8: mortality joinpoint. [file 12889_2024_19104_MOESM8_ESM.zip › Supplement Figure 8 mortality joinpoint/Colombia female 0-85+.jpg]

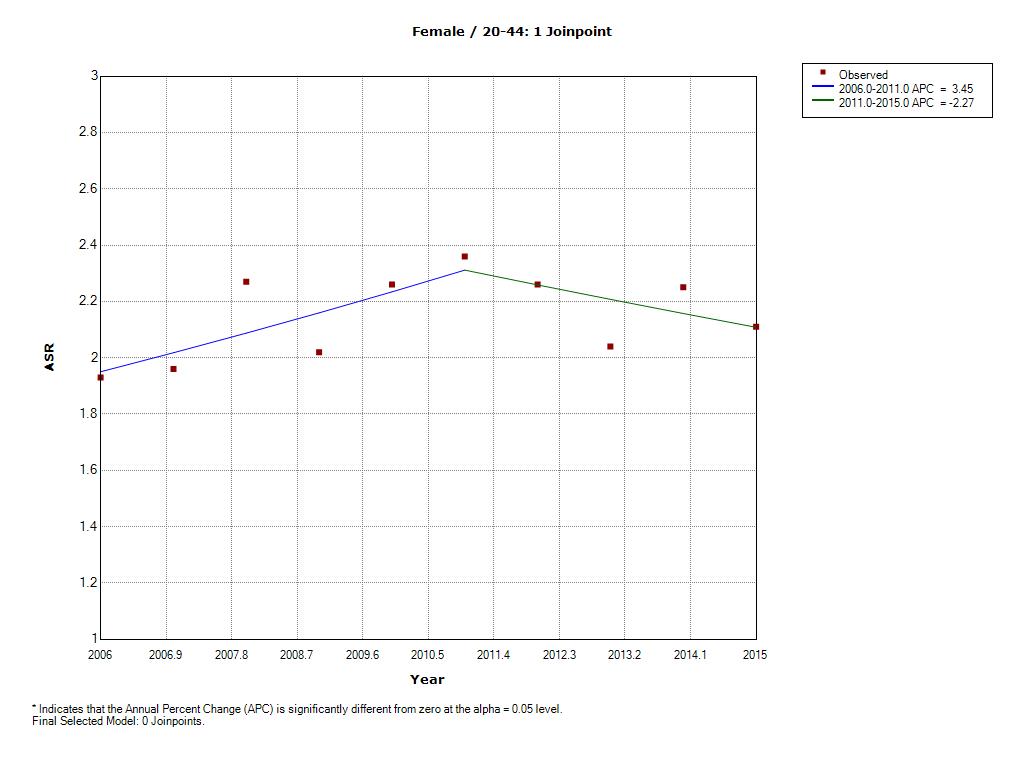

Supplement: Supplementary file 8 — Supplement Figure 8: mortality joinpoint. [file 12889_2024_19104_MOESM8_ESM.zip › Supplement Figure 8 mortality joinpoint/Colombia female 20-44.jpg]

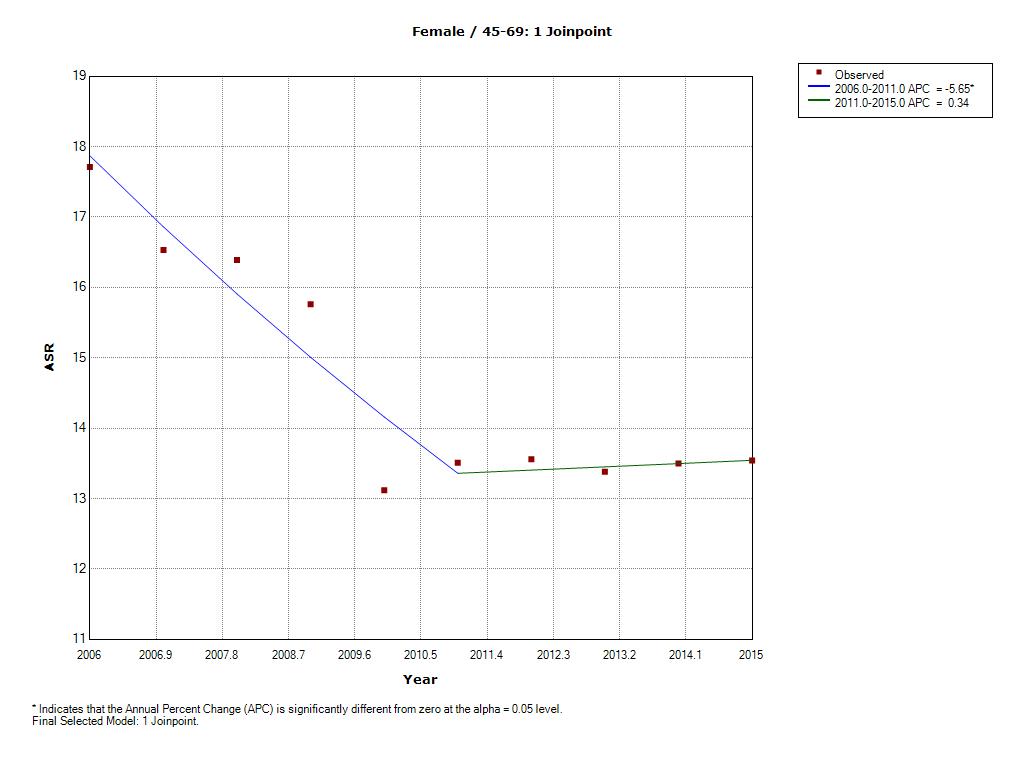

Supplement: Supplementary file 8 — Supplement Figure 8: mortality joinpoint. [file 12889_2024_19104_MOESM8_ESM.zip › Supplement Figure 8 mortality joinpoint/Colombia female 45-69.jpg]

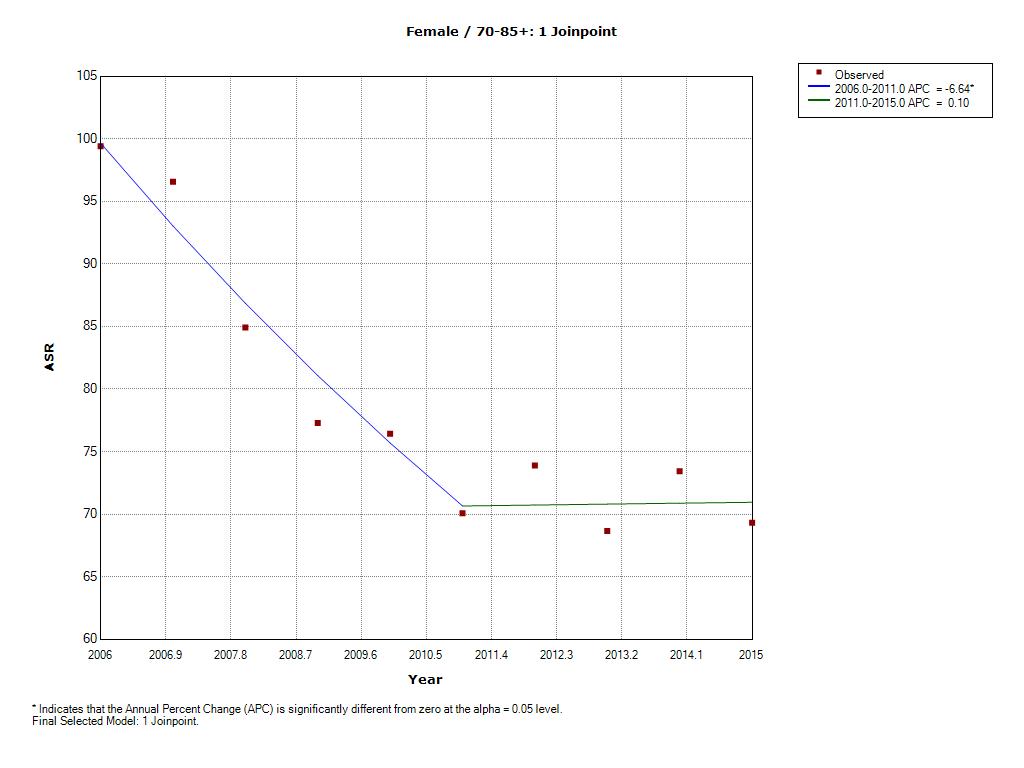

Supplement: Supplementary file 8 — Supplement Figure 8: mortality joinpoint. [file 12889_2024_19104_MOESM8_ESM.zip › Supplement Figure 8 mortality joinpoint/Colombia female 70-85+.jpg]

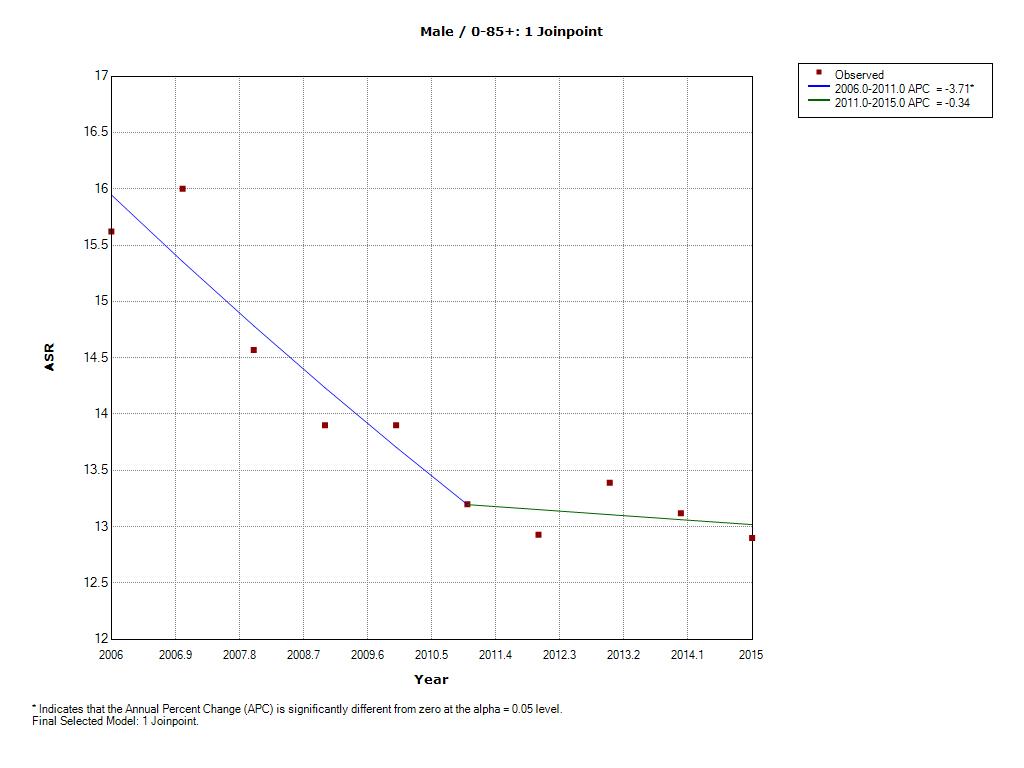

Supplement: Supplementary file 8 — Supplement Figure 8: mortality joinpoint. [file 12889_2024_19104_MOESM8_ESM.zip › Supplement Figure 8 mortality joinpoint/Colombia male 0-85+.jpg]

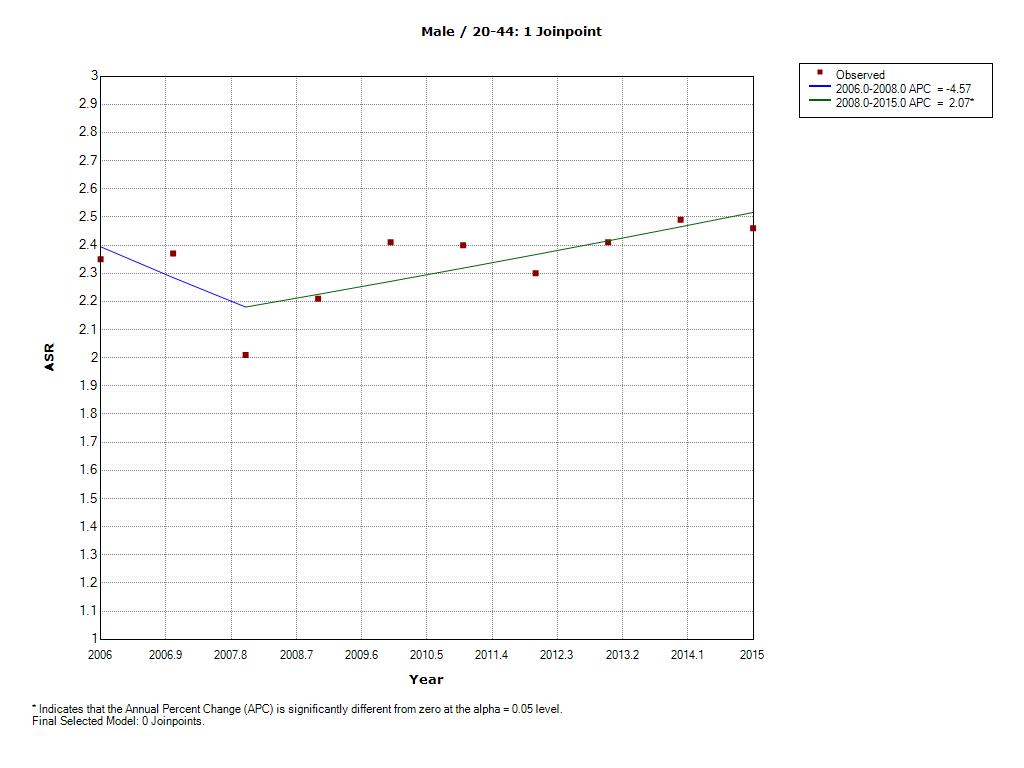

Supplement: Supplementary file 8 — Supplement Figure 8: mortality joinpoint. [file 12889_2024_19104_MOESM8_ESM.zip › Supplement Figure 8 mortality joinpoint/Colombia male 20-44.jpg]

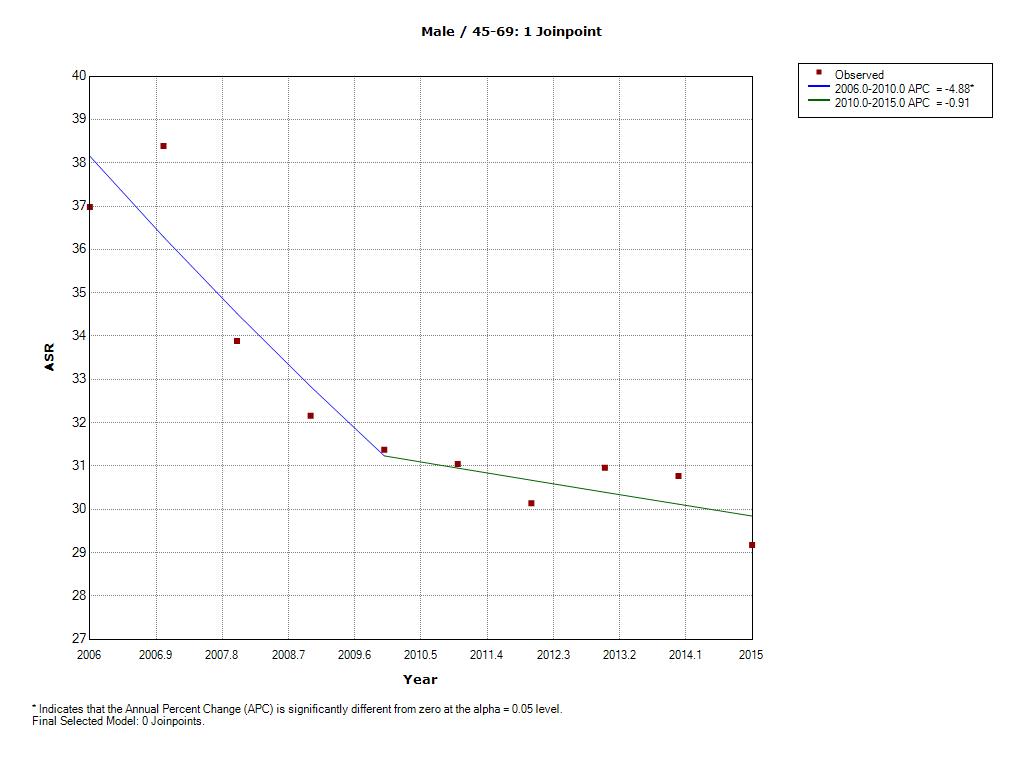

Supplement: Supplementary file 8 — Supplement Figure 8: mortality joinpoint. [file 12889_2024_19104_MOESM8_ESM.zip › Supplement Figure 8 mortality joinpoint/Colombia male 45-69.jpg]

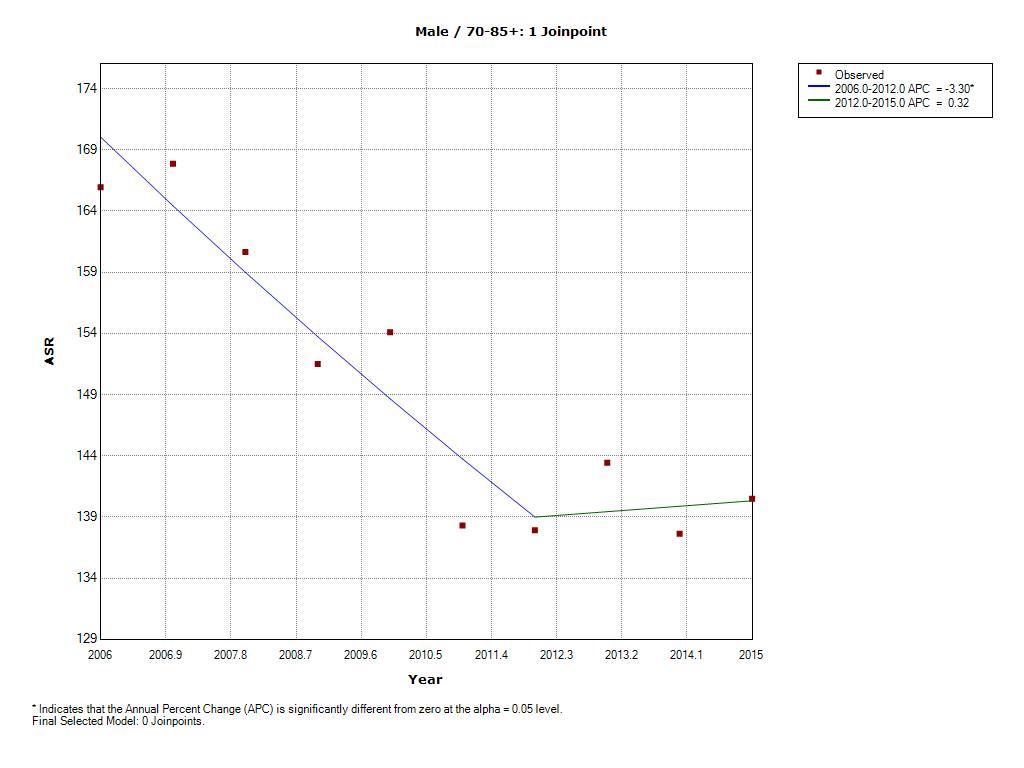

Supplement: Supplementary file 8 — Supplement Figure 8: mortality joinpoint. [file 12889_2024_19104_MOESM8_ESM.zip › Supplement Figure 8 mortality joinpoint/Colombia male 70-85+.jpg]

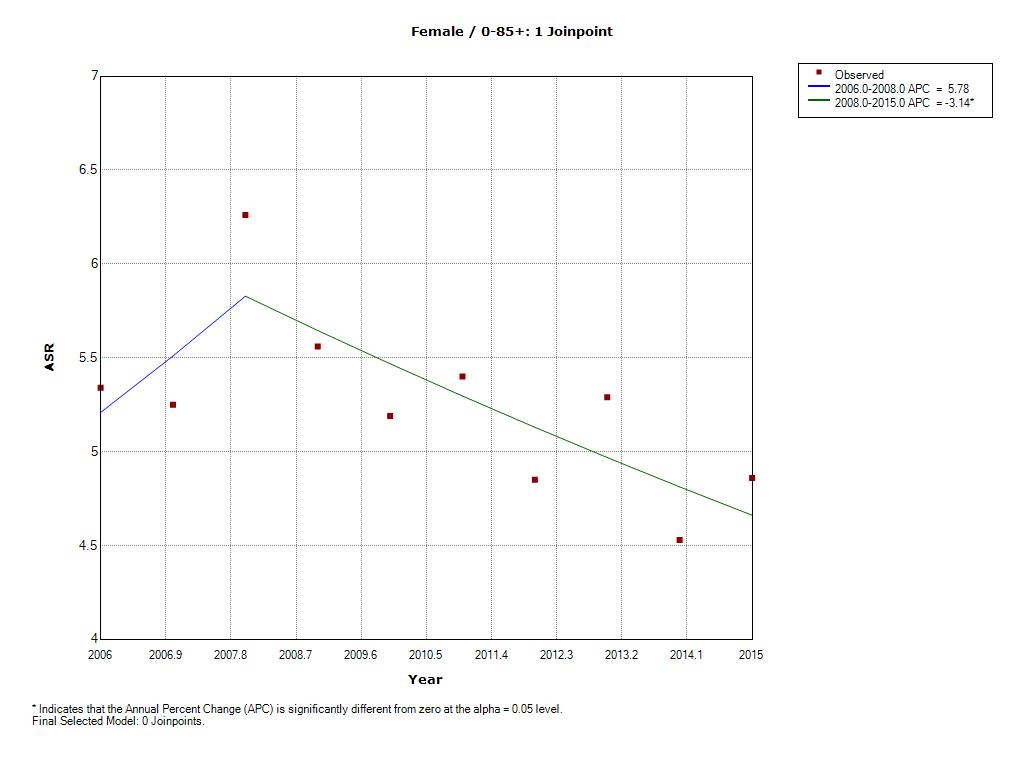

Supplement: Supplementary file 8 — Supplement Figure 8: mortality joinpoint. [file 12889_2024_19104_MOESM8_ESM.zip › Supplement Figure 8 mortality joinpoint/Croatia female 0-85+.jpg]

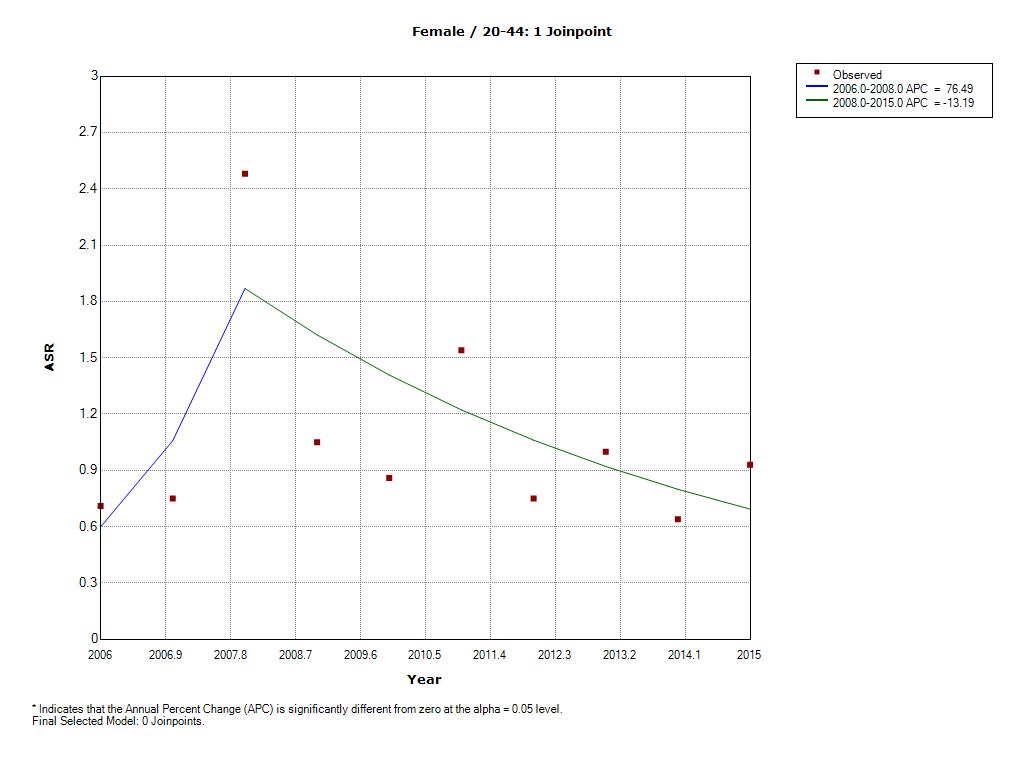

Supplement: Supplementary file 8 — Supplement Figure 8: mortality joinpoint. [file 12889_2024_19104_MOESM8_ESM.zip › Supplement Figure 8 mortality joinpoint/Croatia female 20-44.jpg]

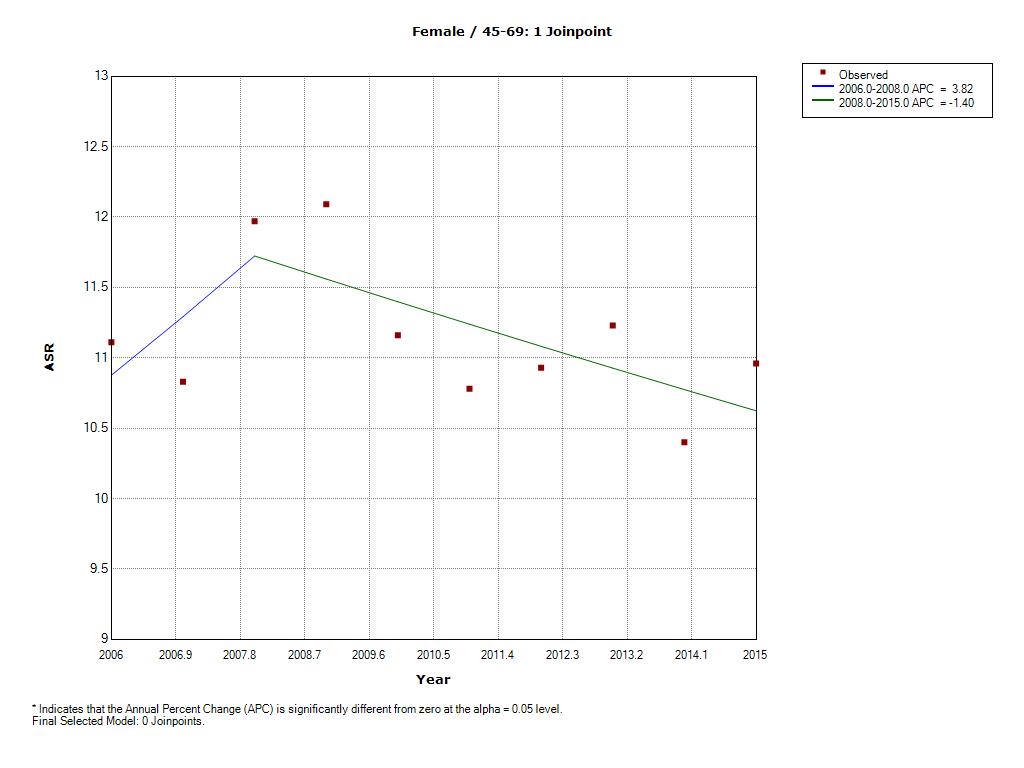

Supplement: Supplementary file 8 — Supplement Figure 8: mortality joinpoint. [file 12889_2024_19104_MOESM8_ESM.zip › Supplement Figure 8 mortality joinpoint/Croatia female 45-69.jpg]

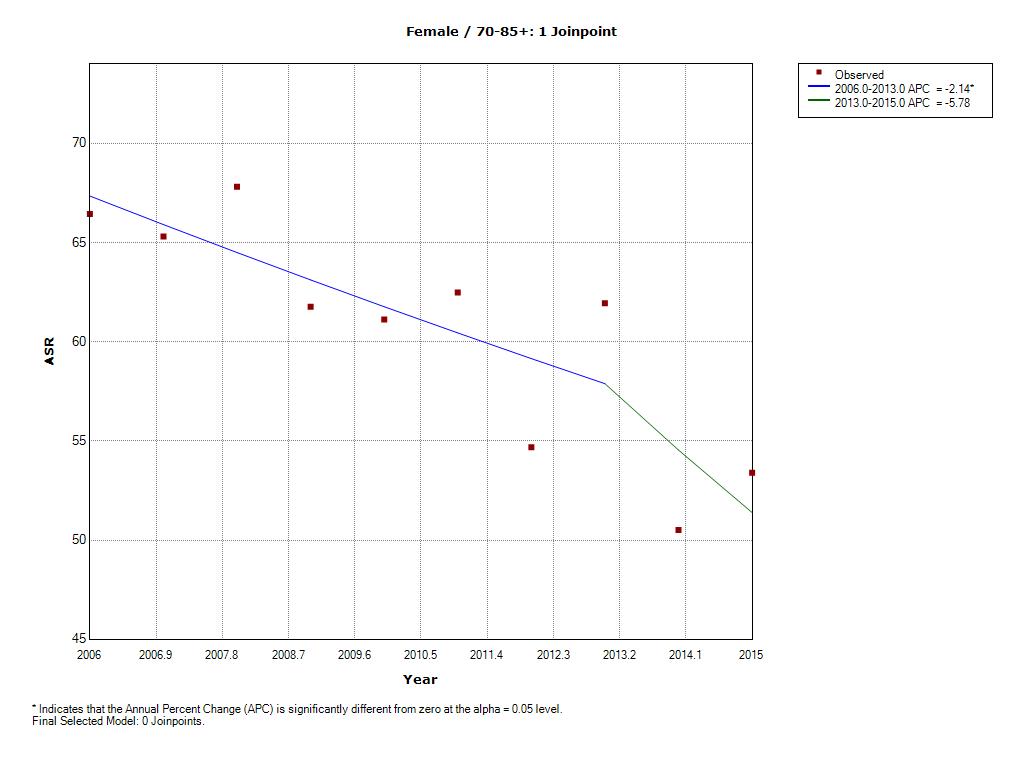

Supplement: Supplementary file 8 — Supplement Figure 8: mortality joinpoint. [file 12889_2024_19104_MOESM8_ESM.zip › Supplement Figure 8 mortality joinpoint/Croatia female 70-85+.jpg]

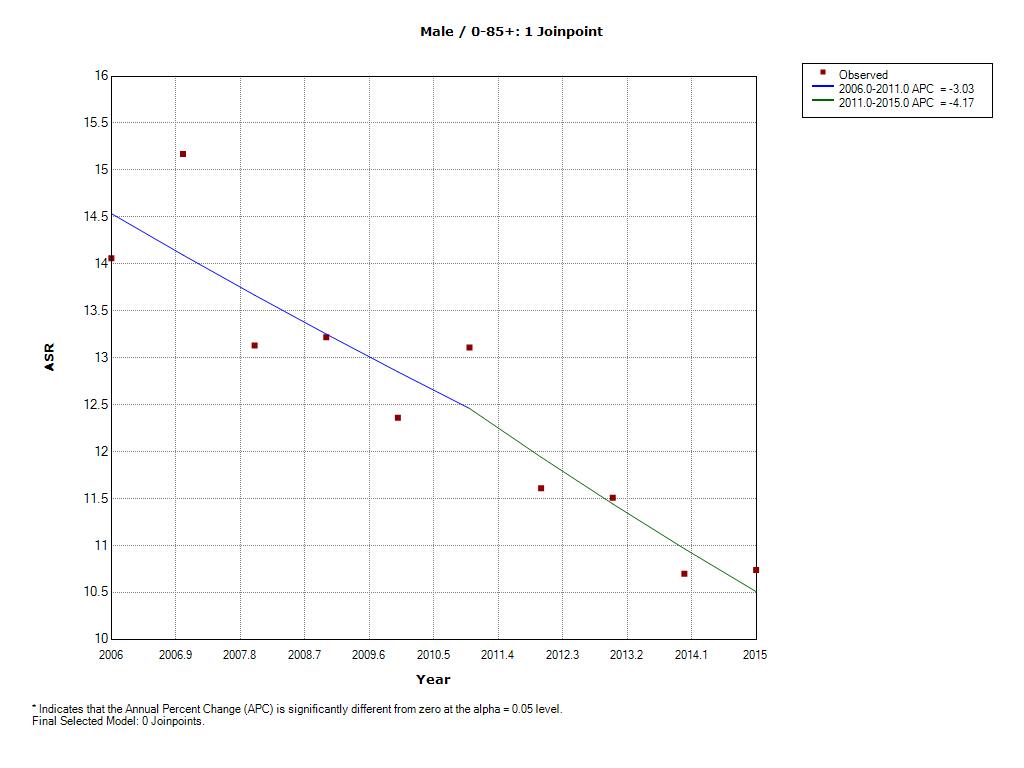

Supplement: Supplementary file 8 — Supplement Figure 8: mortality joinpoint. [file 12889_2024_19104_MOESM8_ESM.zip › Supplement Figure 8 mortality joinpoint/Croatia male 0-85+.jpg]

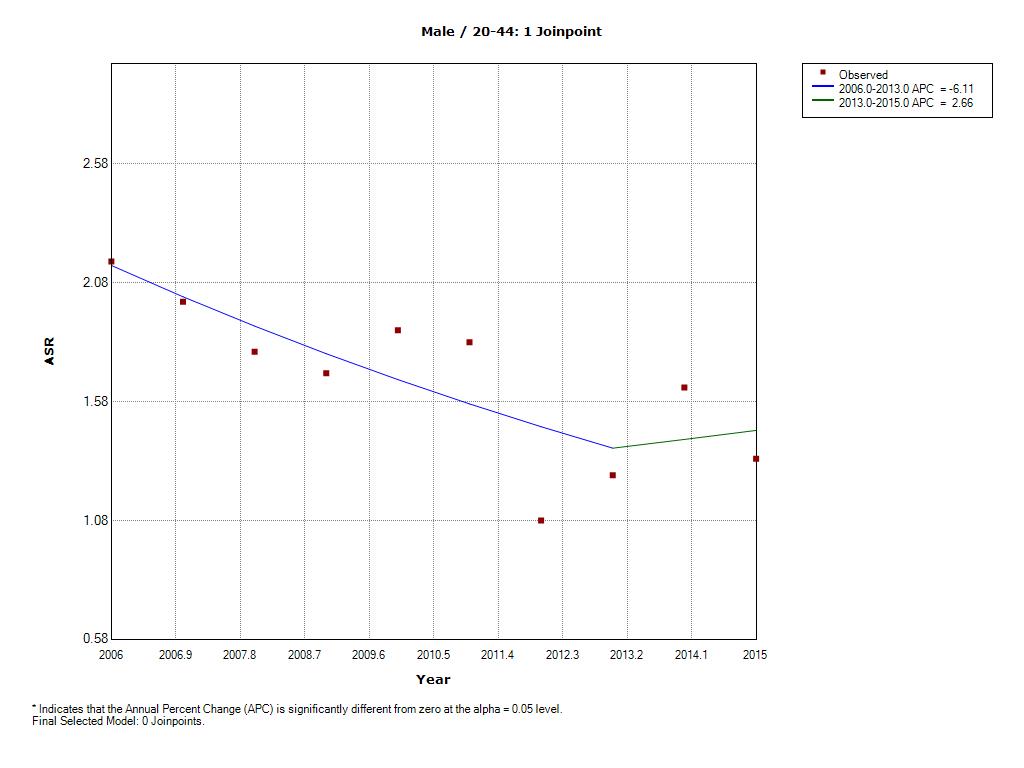

Supplement: Supplementary file 8 — Supplement Figure 8: mortality joinpoint. [file 12889_2024_19104_MOESM8_ESM.zip › Supplement Figure 8 mortality joinpoint/Croatia male 20-44.jpg]

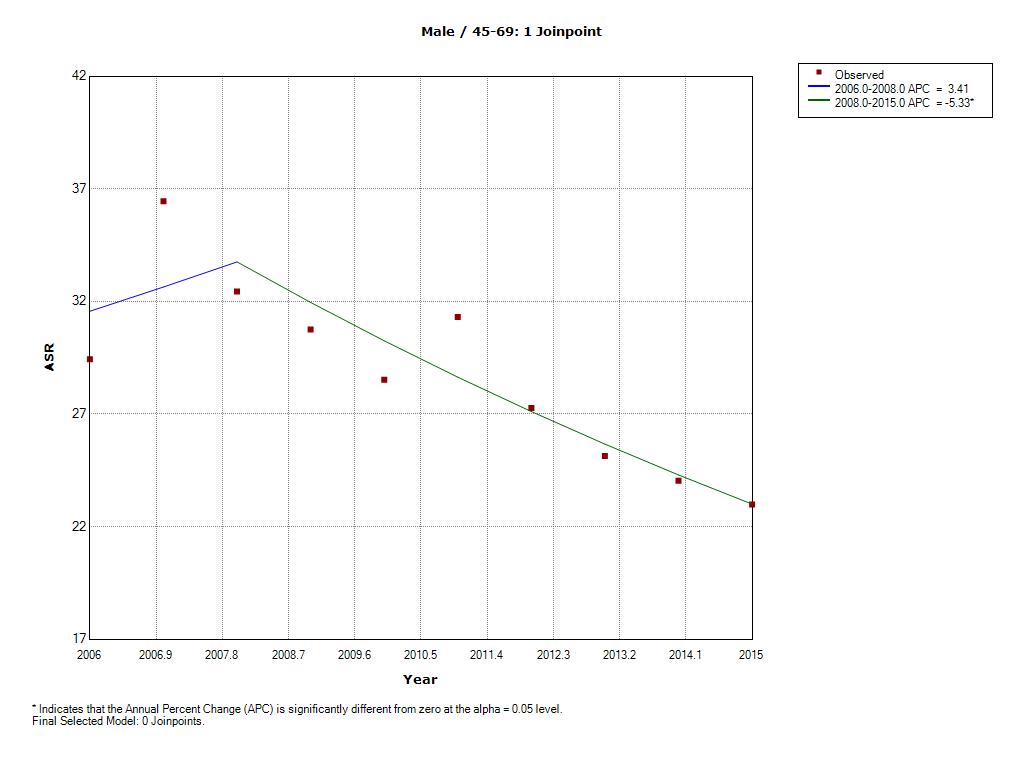

Supplement: Supplementary file 8 — Supplement Figure 8: mortality joinpoint. [file 12889_2024_19104_MOESM8_ESM.zip › Supplement Figure 8 mortality joinpoint/Croatia male 45-69.jpg]

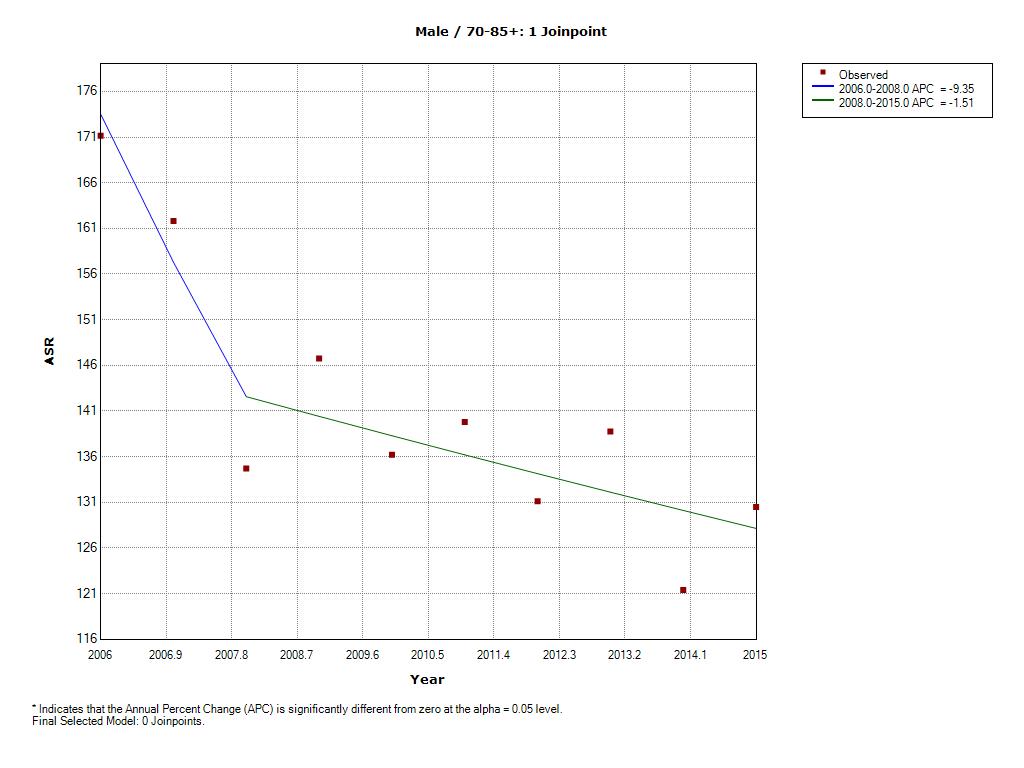

Supplement: Supplementary file 8 — Supplement Figure 8: mortality joinpoint. [file 12889_2024_19104_MOESM8_ESM.zip › Supplement Figure 8 mortality joinpoint/Croatia male 70-85+.jpg]

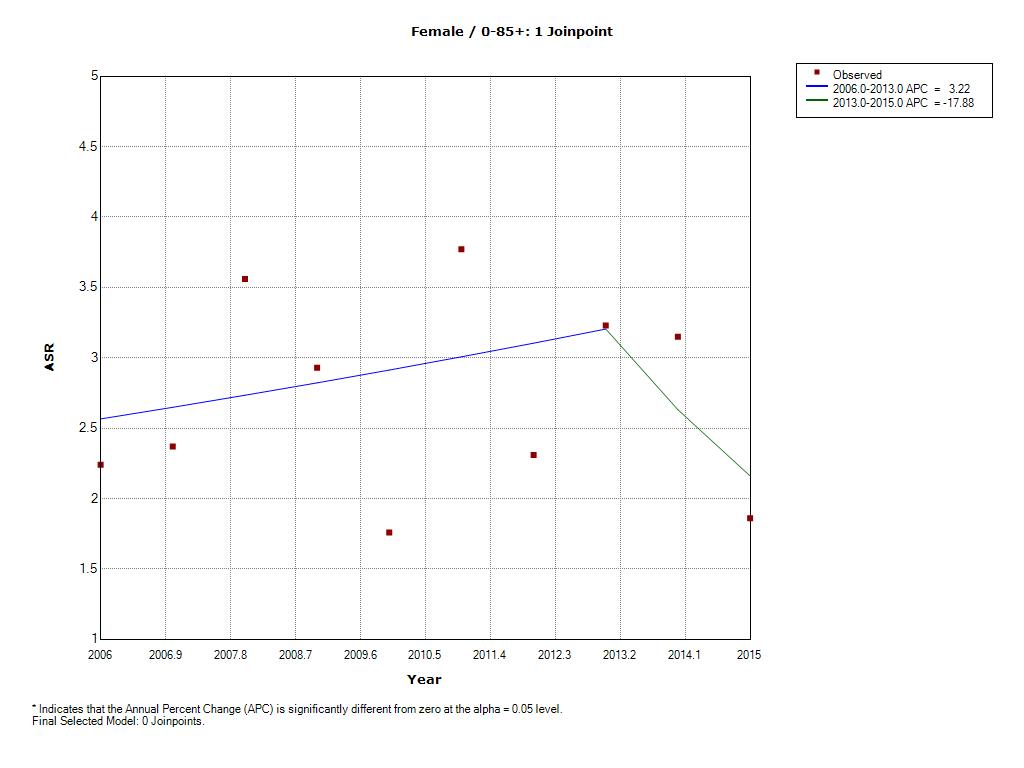

Supplement: Supplementary file 8 — Supplement Figure 8: mortality joinpoint. [file 12889_2024_19104_MOESM8_ESM.zip › Supplement Figure 8 mortality joinpoint/Cyprus female 0-85+.jpg]

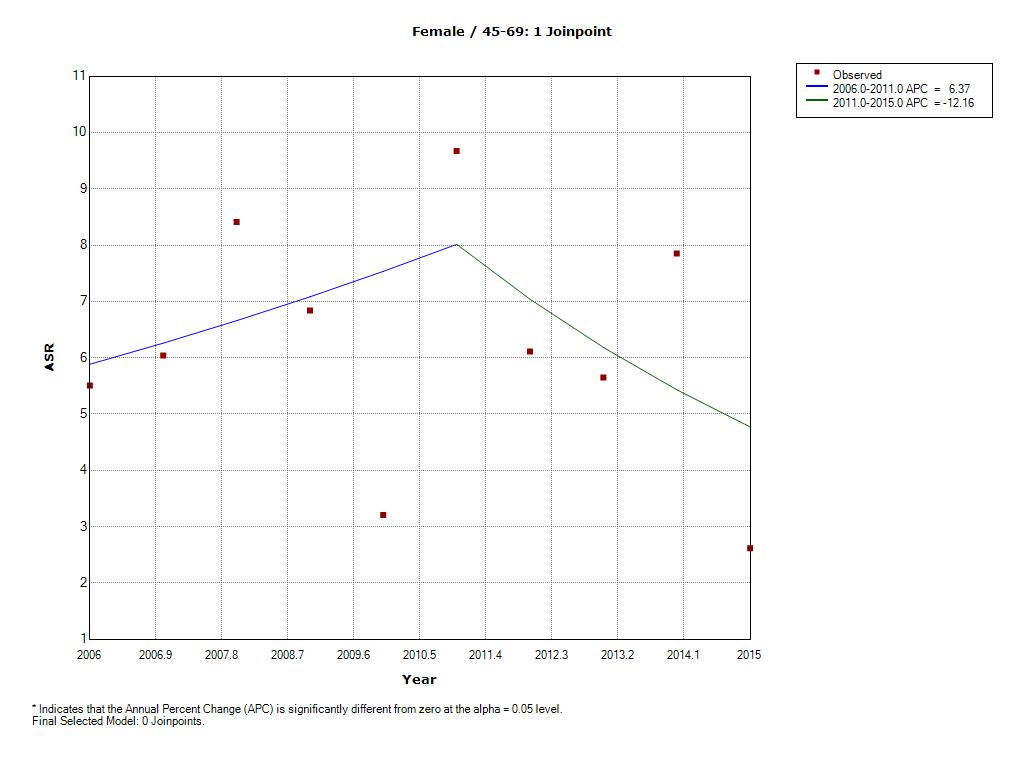

Supplement: Supplementary file 8 — Supplement Figure 8: mortality joinpoint. [file 12889_2024_19104_MOESM8_ESM.zip › Supplement Figure 8 mortality joinpoint/Cyprus female 45-69.jpg]

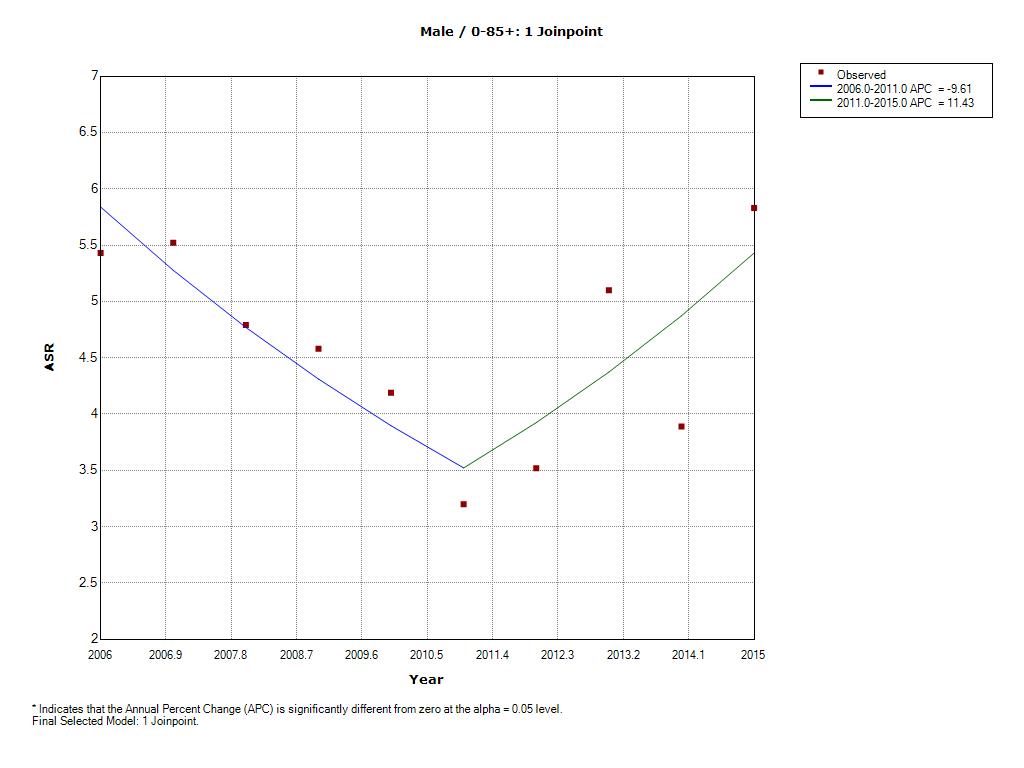

Supplement: Supplementary file 8 — Supplement Figure 8: mortality joinpoint. [file 12889_2024_19104_MOESM8_ESM.zip › Supplement Figure 8 mortality joinpoint/Cyprus male 0-85+.jpg]

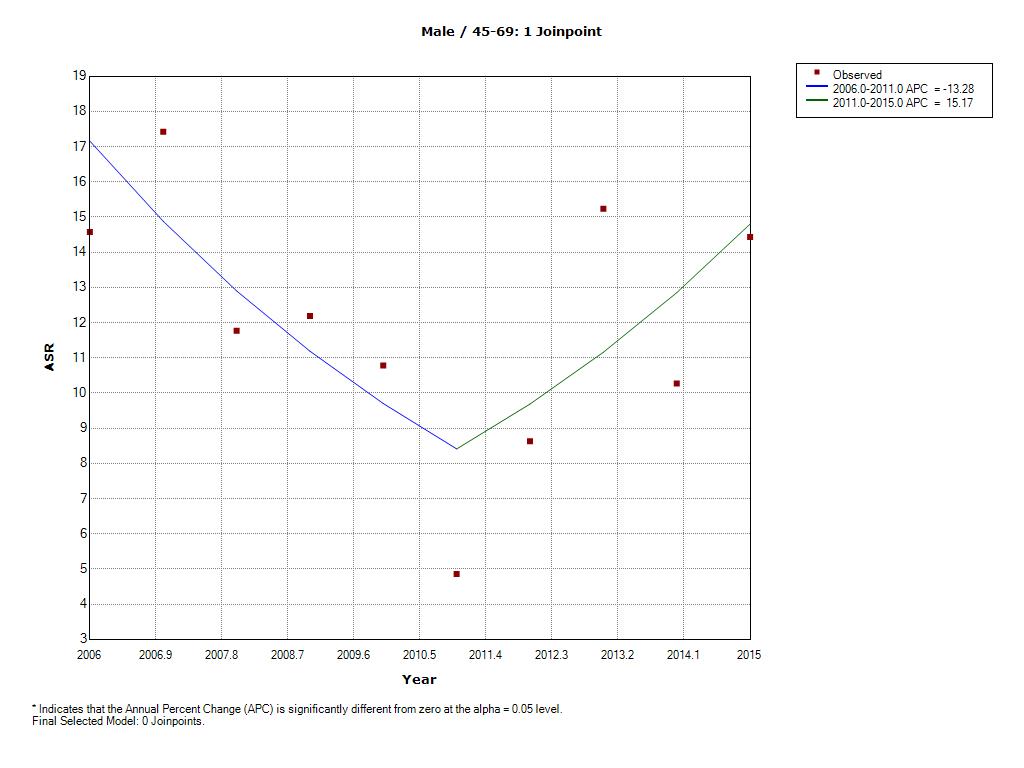

Supplement: Supplementary file 8 — Supplement Figure 8: mortality joinpoint. [file 12889_2024_19104_MOESM8_ESM.zip › Supplement Figure 8 mortality joinpoint/Cyprus male 20-44.jpg]

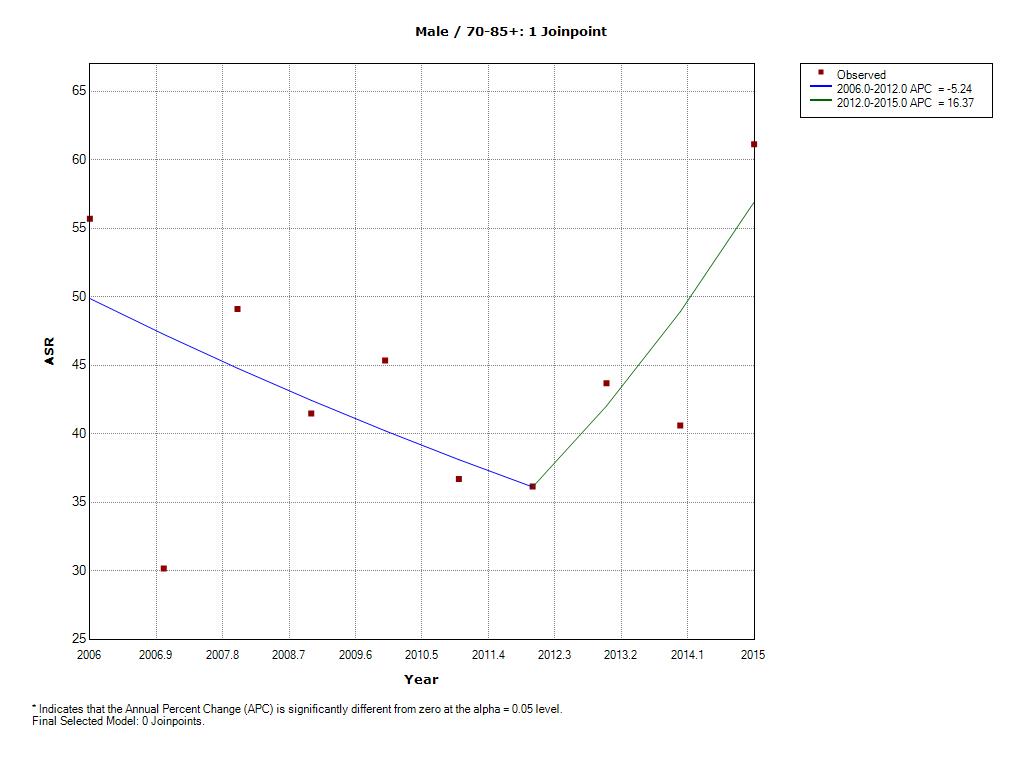

Supplement: Supplementary file 8 — Supplement Figure 8: mortality joinpoint. [file 12889_2024_19104_MOESM8_ESM.zip › Supplement Figure 8 mortality joinpoint/Cyprus male 45-69.jpg]

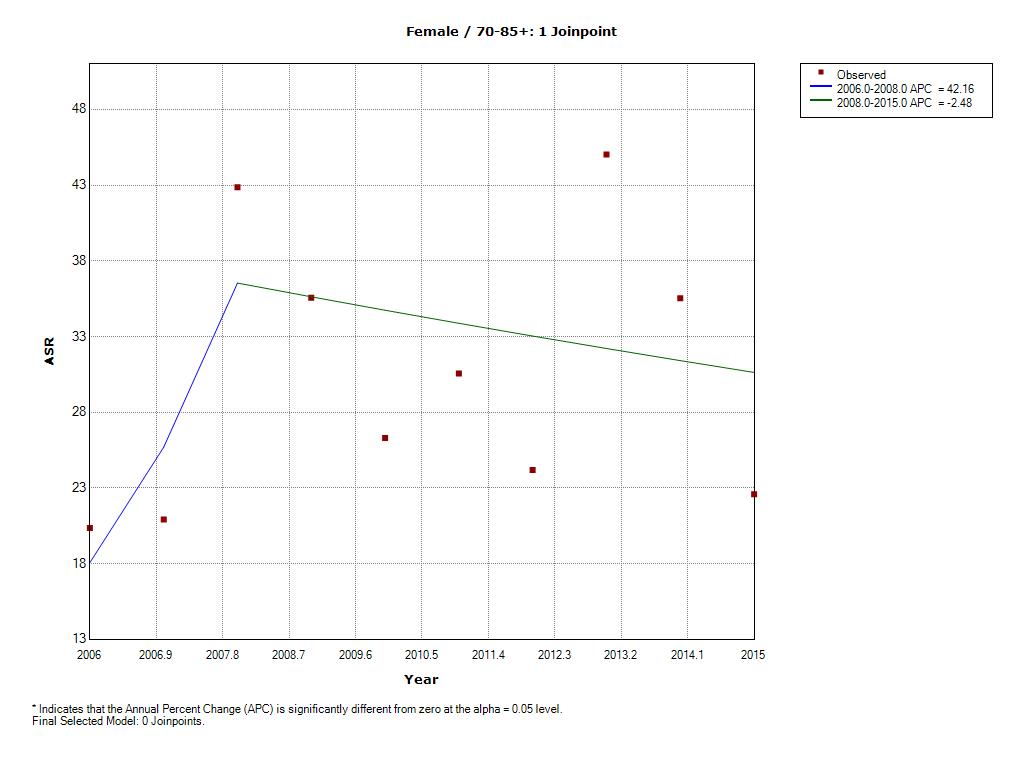

Supplement: Supplementary file 8 — Supplement Figure 8: mortality joinpoint. [file 12889_2024_19104_MOESM8_ESM.zip › Supplement Figure 8 mortality joinpoint/Cyprus male 70-85+.jpg]

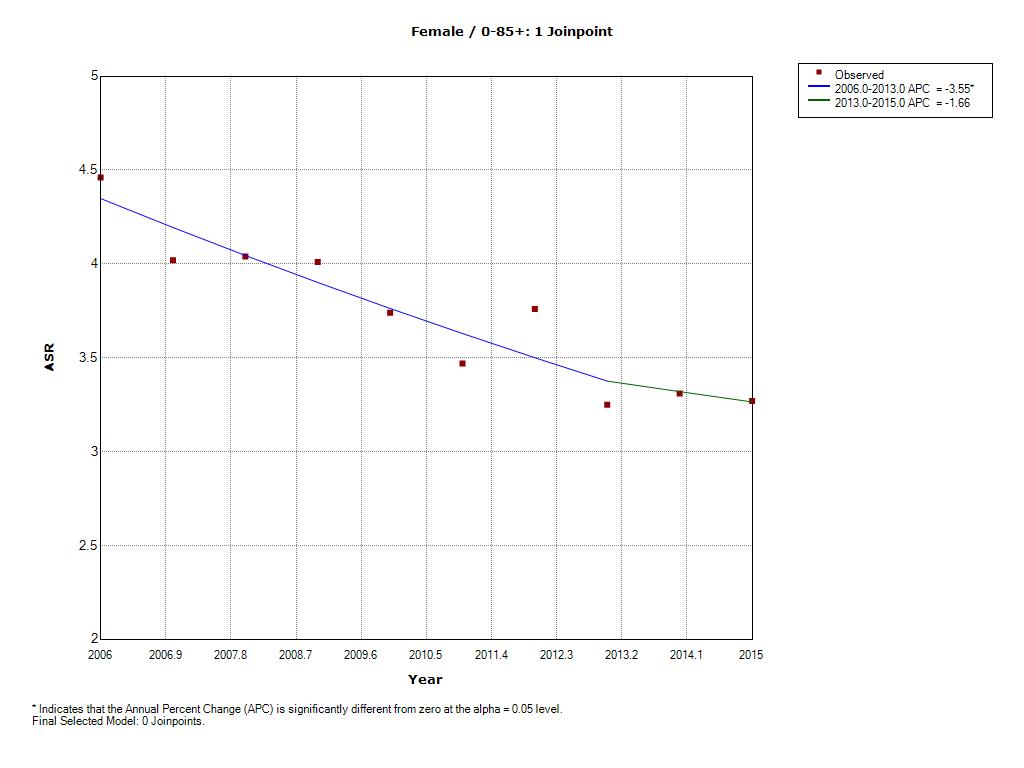

Supplement: Supplementary file 8 — Supplement Figure 8: mortality joinpoint. [file 12889_2024_19104_MOESM8_ESM.zip › Supplement Figure 8 mortality joinpoint/Czech Republic female 0-85+.jpg]

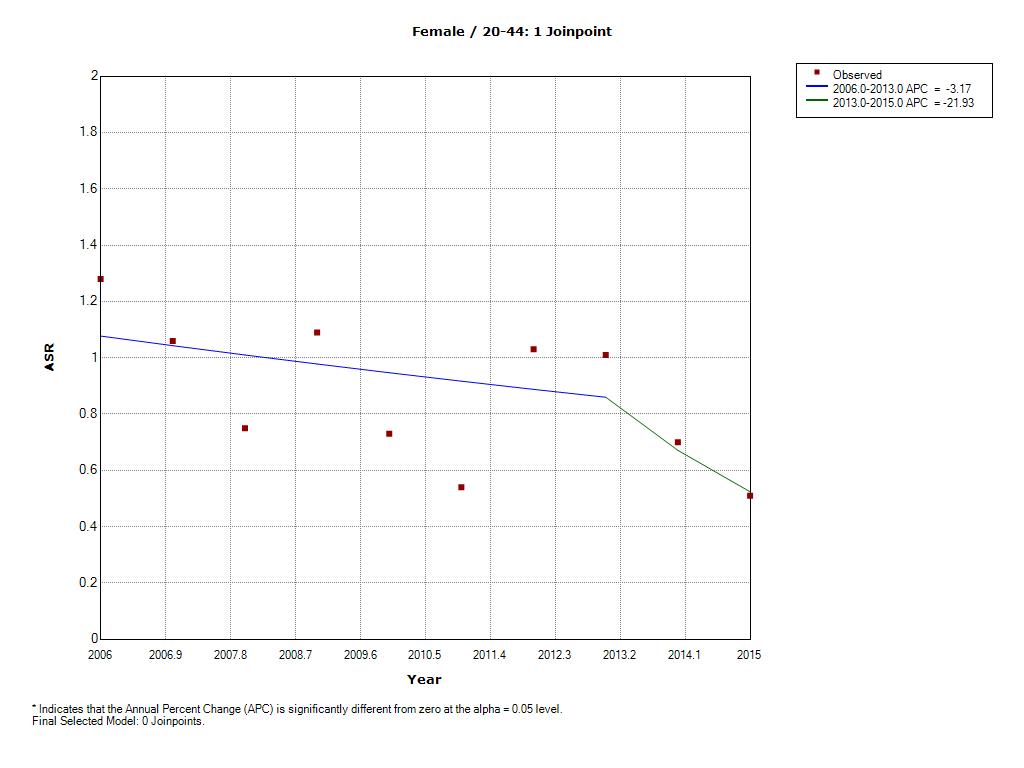

Supplement: Supplementary file 8 — Supplement Figure 8: mortality joinpoint. [file 12889_2024_19104_MOESM8_ESM.zip › Supplement Figure 8 mortality joinpoint/Czech Republic female 20-44.jpg]

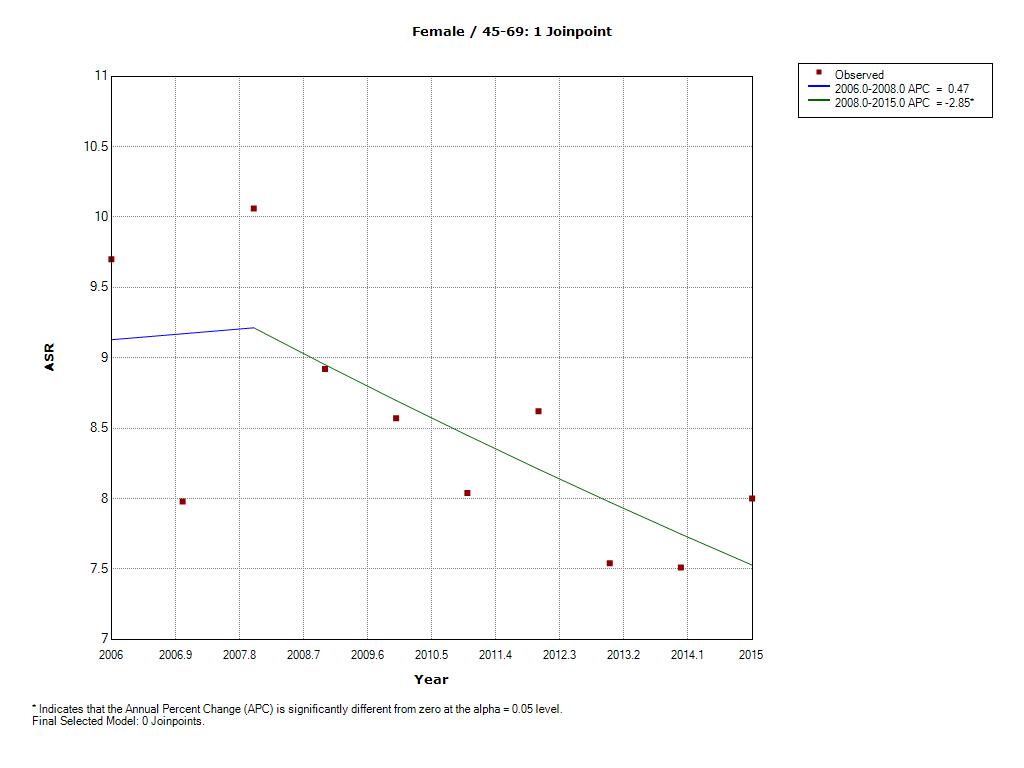

Supplement: Supplementary file 8 — Supplement Figure 8: mortality joinpoint. [file 12889_2024_19104_MOESM8_ESM.zip › Supplement Figure 8 mortality joinpoint/Czech Republic female 45-69.jpg]

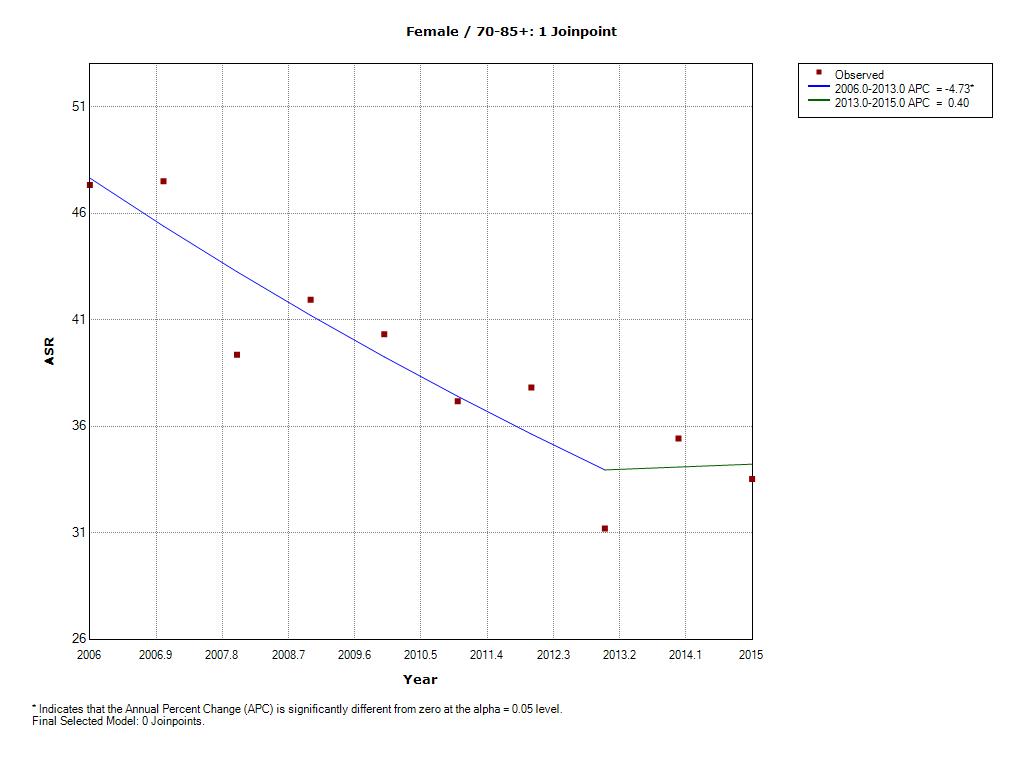

Supplement: Supplementary file 8 — Supplement Figure 8: mortality joinpoint. [file 12889_2024_19104_MOESM8_ESM.zip › Supplement Figure 8 mortality joinpoint/Czech Republic female 70-85+.jpg]

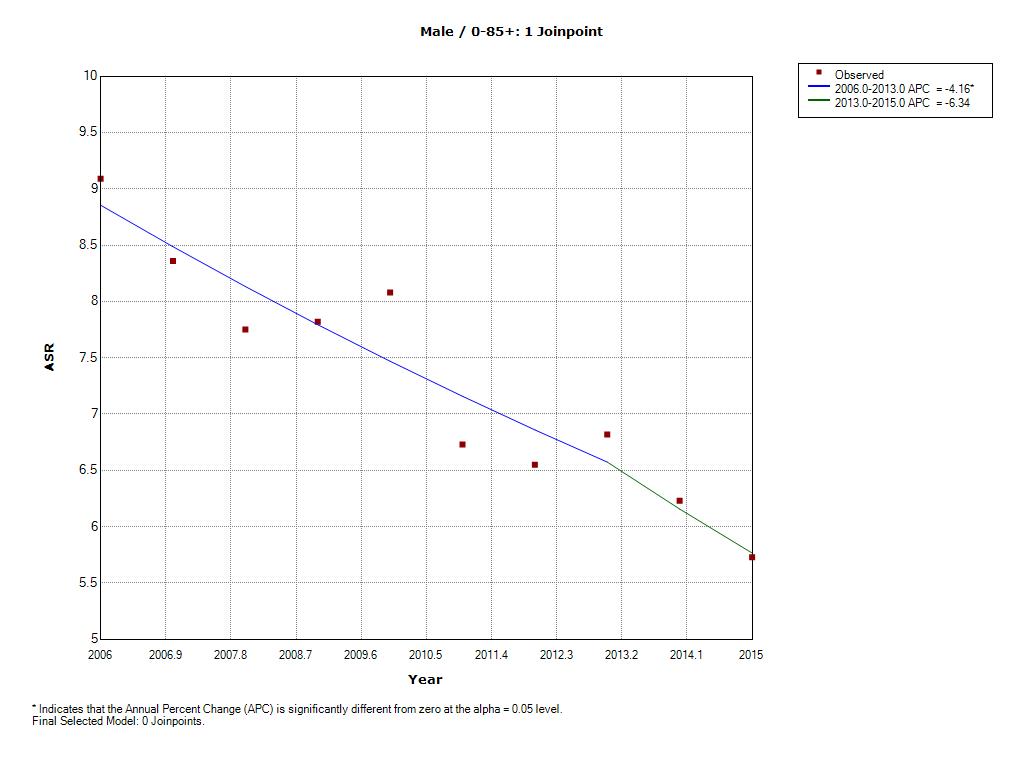

Supplement: Supplementary file 8 — Supplement Figure 8: mortality joinpoint. [file 12889_2024_19104_MOESM8_ESM.zip › Supplement Figure 8 mortality joinpoint/Czech Republic male 0-85+.jpg]

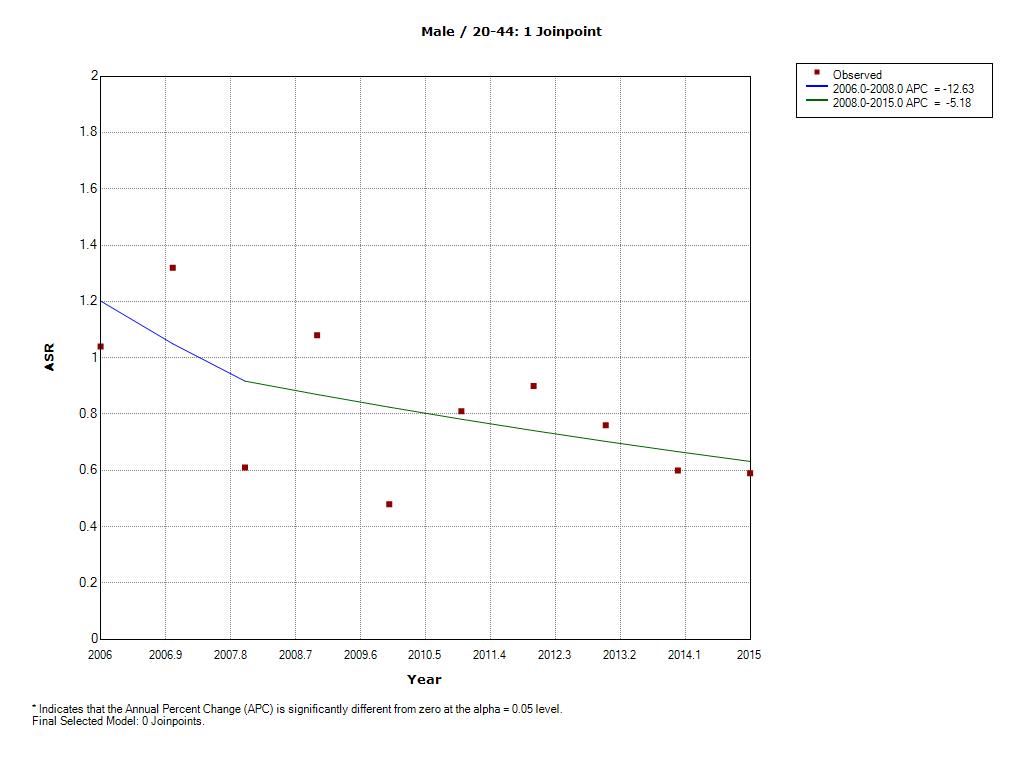

Supplement: Supplementary file 8 — Supplement Figure 8: mortality joinpoint. [file 12889_2024_19104_MOESM8_ESM.zip › Supplement Figure 8 mortality joinpoint/Czech Republic male 20-44.jpg]

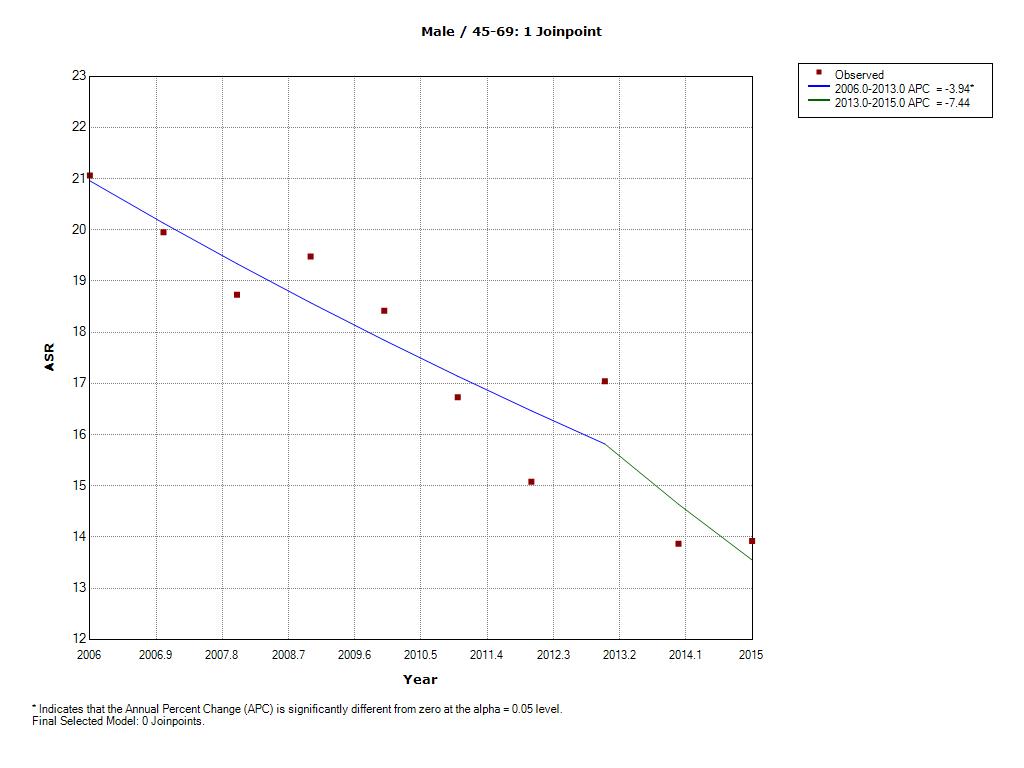

Supplement: Supplementary file 8 — Supplement Figure 8: mortality joinpoint. [file 12889_2024_19104_MOESM8_ESM.zip › Supplement Figure 8 mortality joinpoint/Czech Republic male 45-69.jpg]

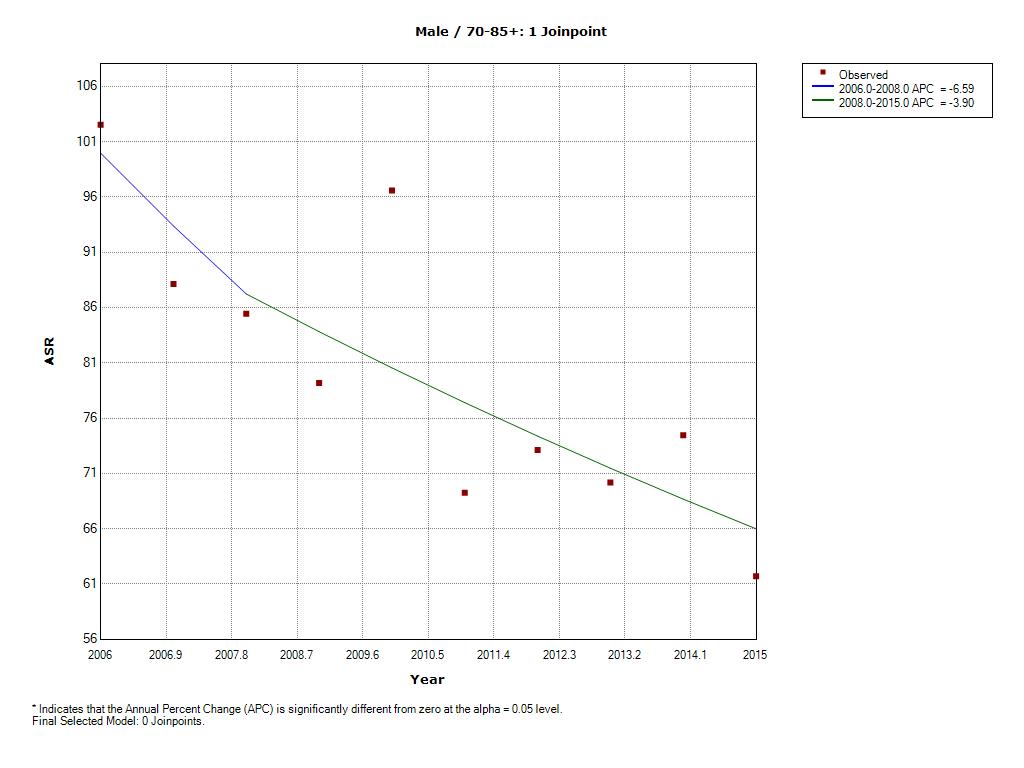

Supplement: Supplementary file 8 — Supplement Figure 8: mortality joinpoint. [file 12889_2024_19104_MOESM8_ESM.zip › Supplement Figure 8 mortality joinpoint/Czech Republic male 70-85+.jpg]

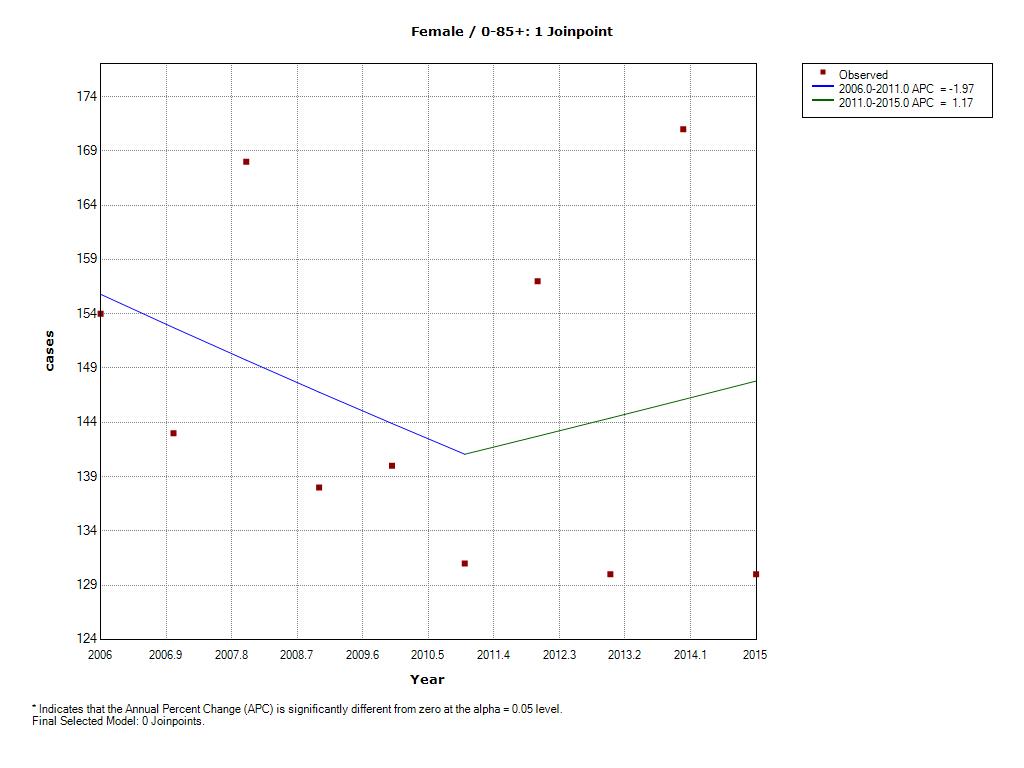

Supplement: Supplementary file 8 — Supplement Figure 8: mortality joinpoint. [file 12889_2024_19104_MOESM8_ESM.zip › Supplement Figure 8 mortality joinpoint/Denmark female 0-85+.jpg]

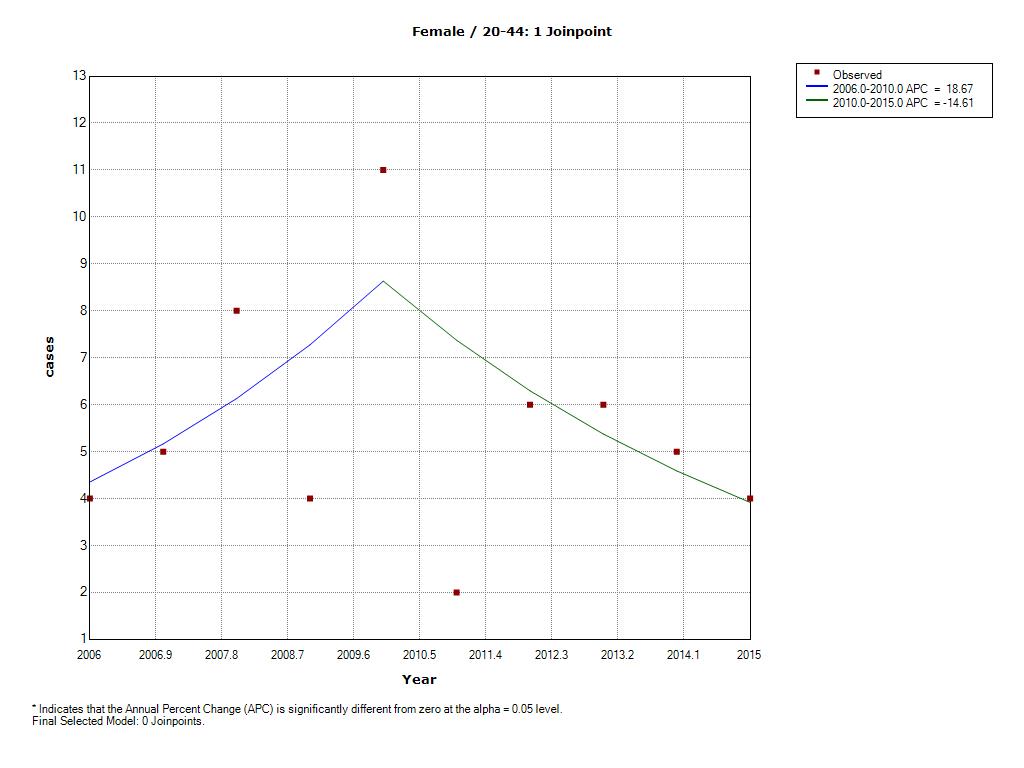

Supplement: Supplementary file 8 — Supplement Figure 8: mortality joinpoint. [file 12889_2024_19104_MOESM8_ESM.zip › Supplement Figure 8 mortality joinpoint/Denmark female 20-44.jpg]

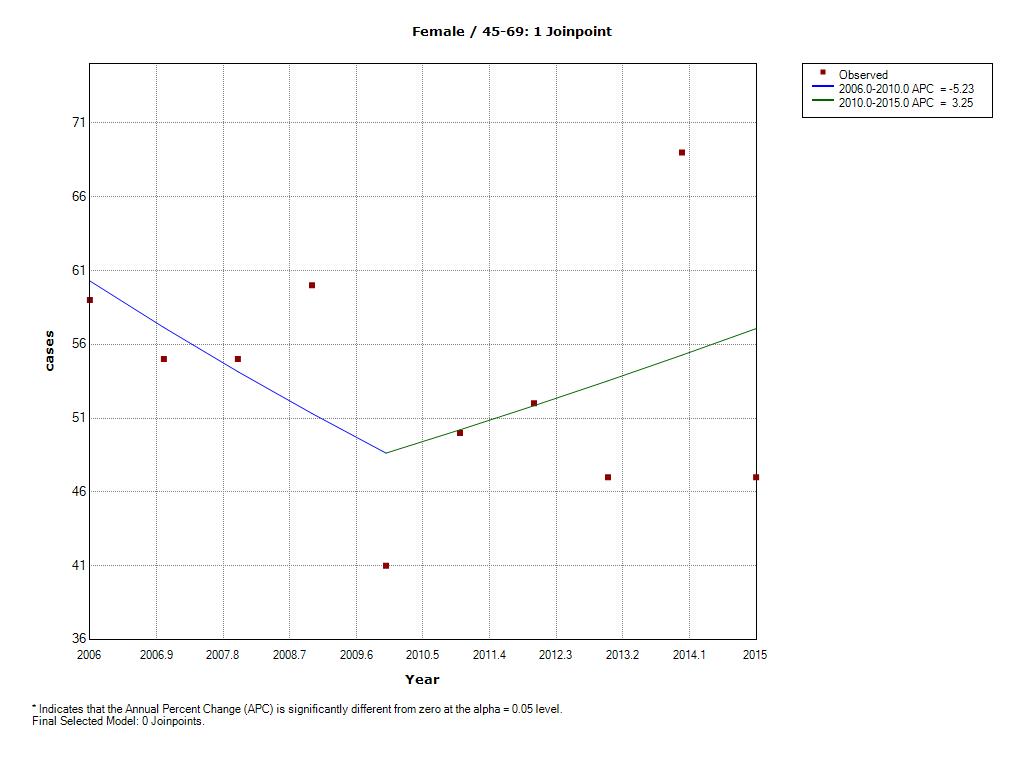

Supplement: Supplementary file 8 — Supplement Figure 8: mortality joinpoint. [file 12889_2024_19104_MOESM8_ESM.zip › Supplement Figure 8 mortality joinpoint/Denmark female 45-69.jpg]

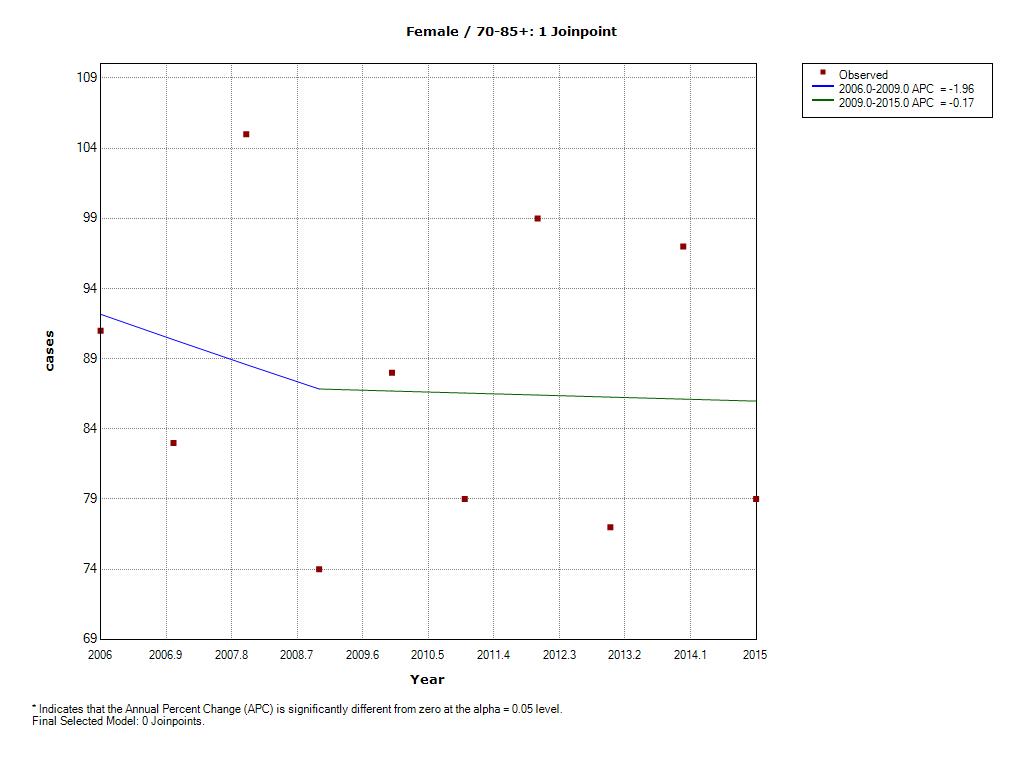

Supplement: Supplementary file 8 — Supplement Figure 8: mortality joinpoint. [file 12889_2024_19104_MOESM8_ESM.zip › Supplement Figure 8 mortality joinpoint/Denmark female 70-85+.jpg]

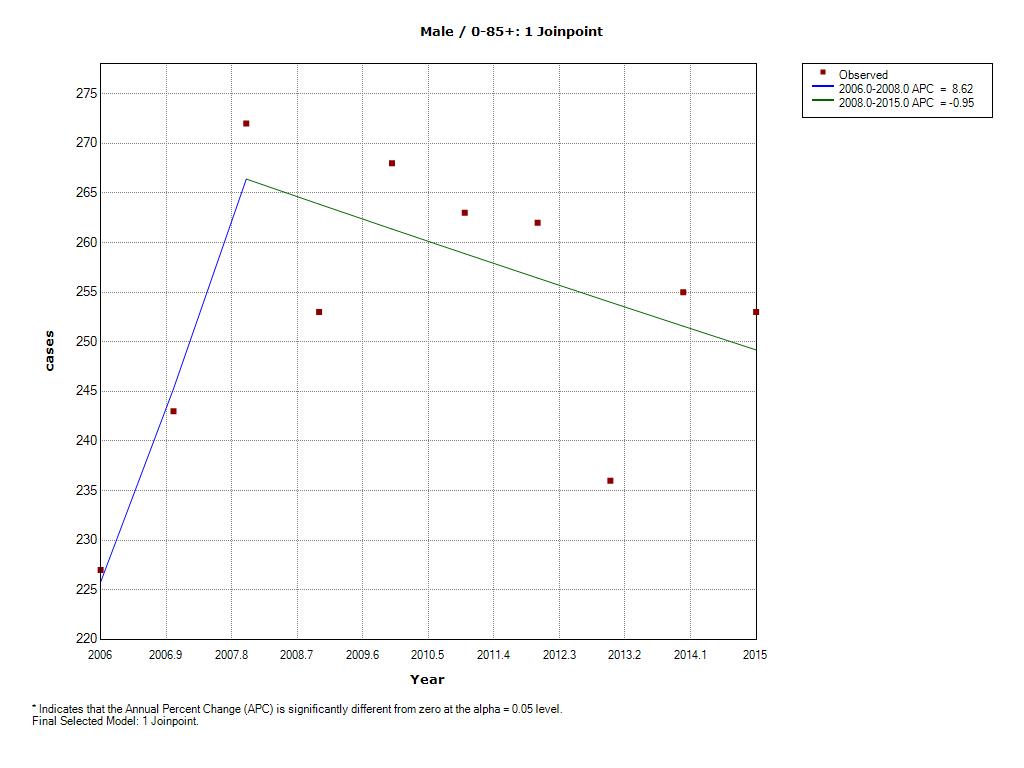

Supplement: Supplementary file 8 — Supplement Figure 8: mortality joinpoint. [file 12889_2024_19104_MOESM8_ESM.zip › Supplement Figure 8 mortality joinpoint/Denmark male 0-85+.jpg]

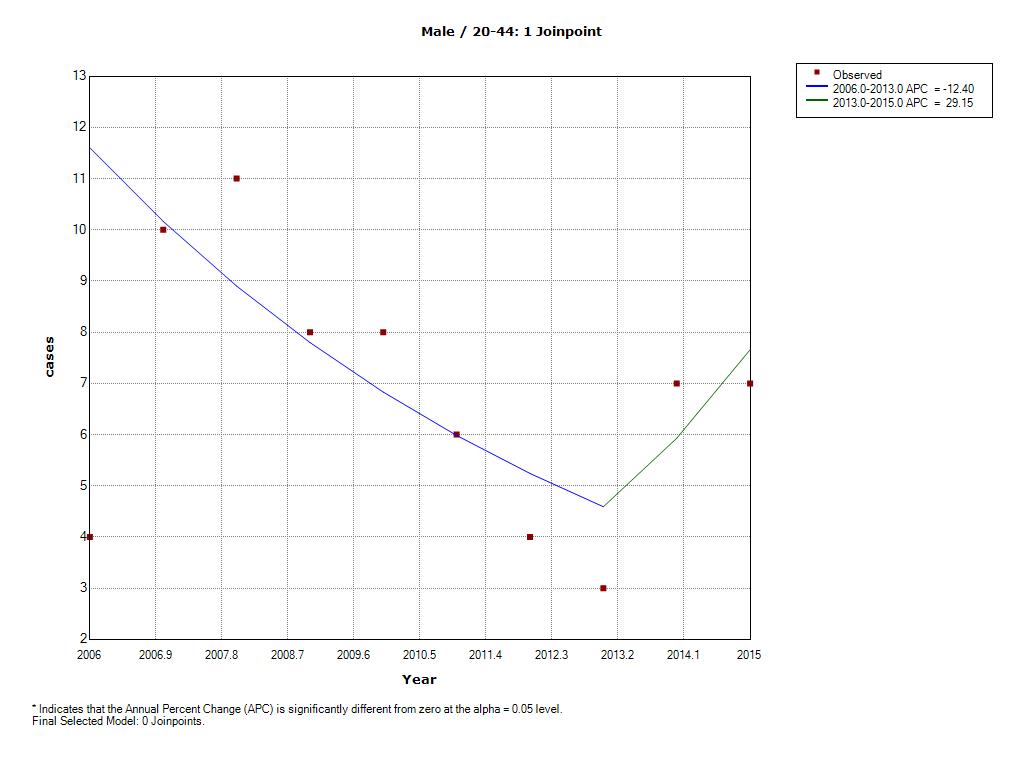

Supplement: Supplementary file 8 — Supplement Figure 8: mortality joinpoint. [file 12889_2024_19104_MOESM8_ESM.zip › Supplement Figure 8 mortality joinpoint/Denmark male 20-44.jpg]

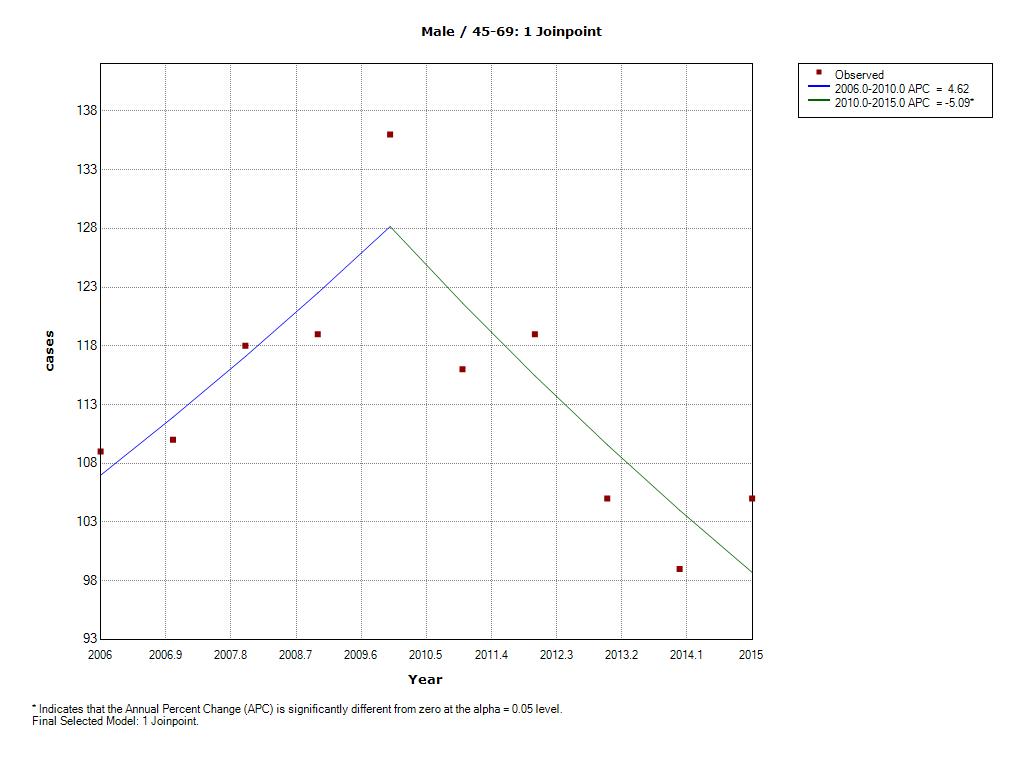

Supplement: Supplementary file 8 — Supplement Figure 8: mortality joinpoint. [file 12889_2024_19104_MOESM8_ESM.zip › Supplement Figure 8 mortality joinpoint/Denmark male 45-69.jpg]

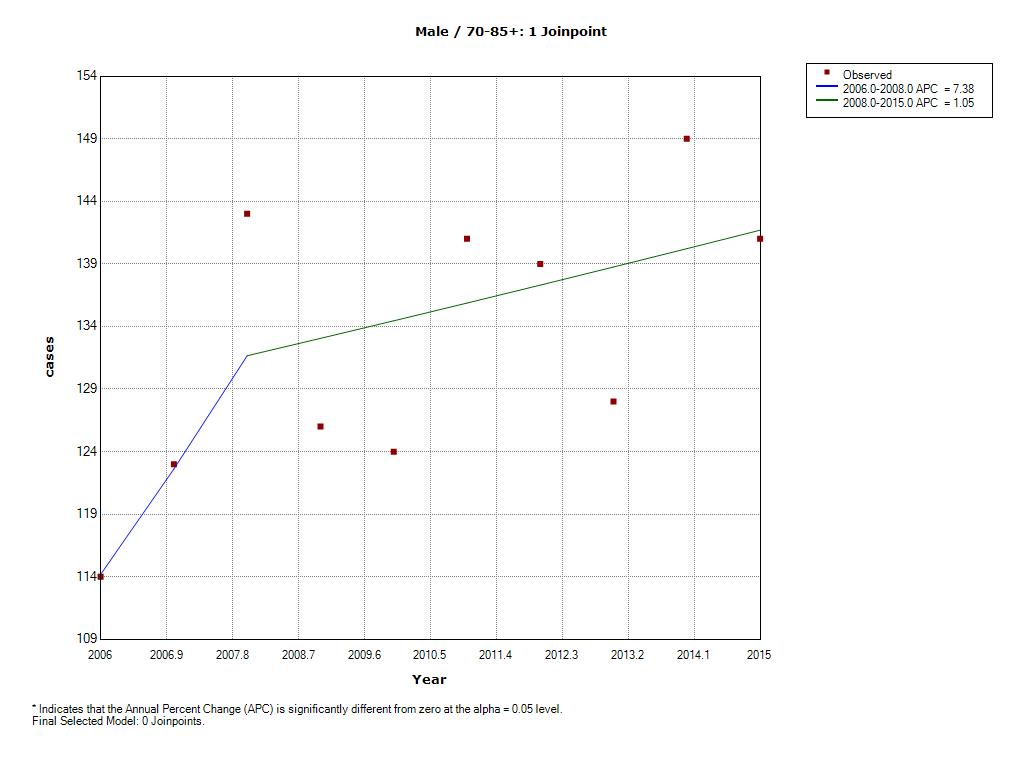

Supplement: Supplementary file 8 — Supplement Figure 8: mortality joinpoint. [file 12889_2024_19104_MOESM8_ESM.zip › Supplement Figure 8 mortality joinpoint/Denmark male 70-85+.jpg]

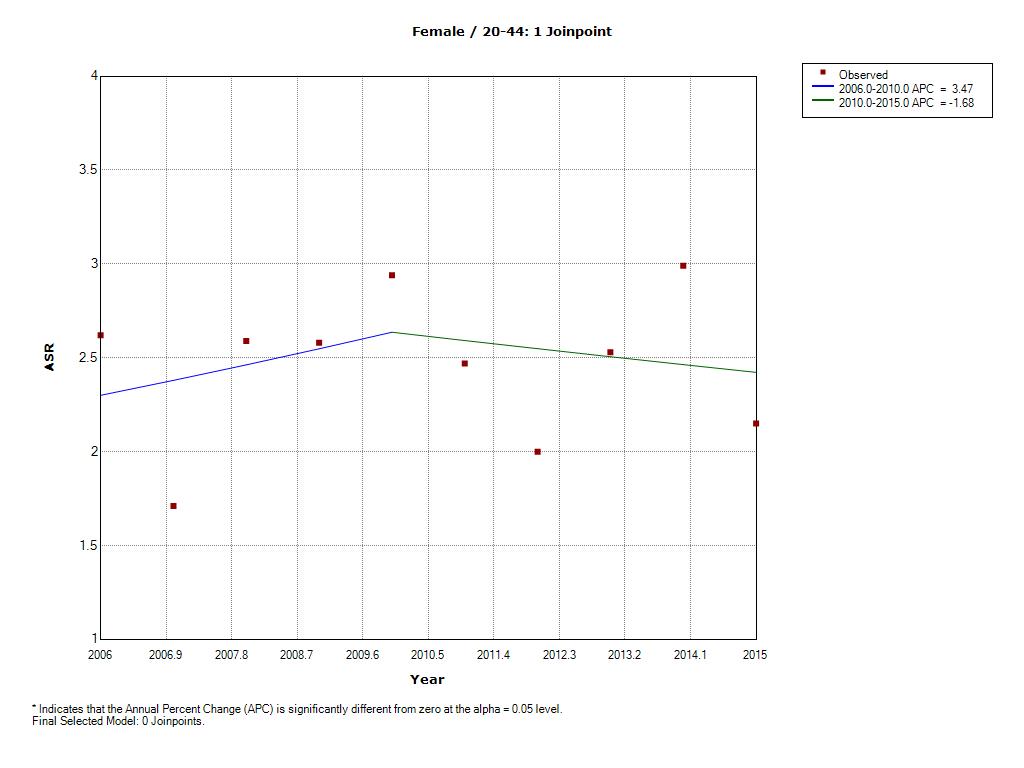

Supplement: Supplementary file 8 — Supplement Figure 8: mortality joinpoint. [file 12889_2024_19104_MOESM8_ESM.zip › Supplement Figure 8 mortality joinpoint/Ecuado female 20-44.jpg]

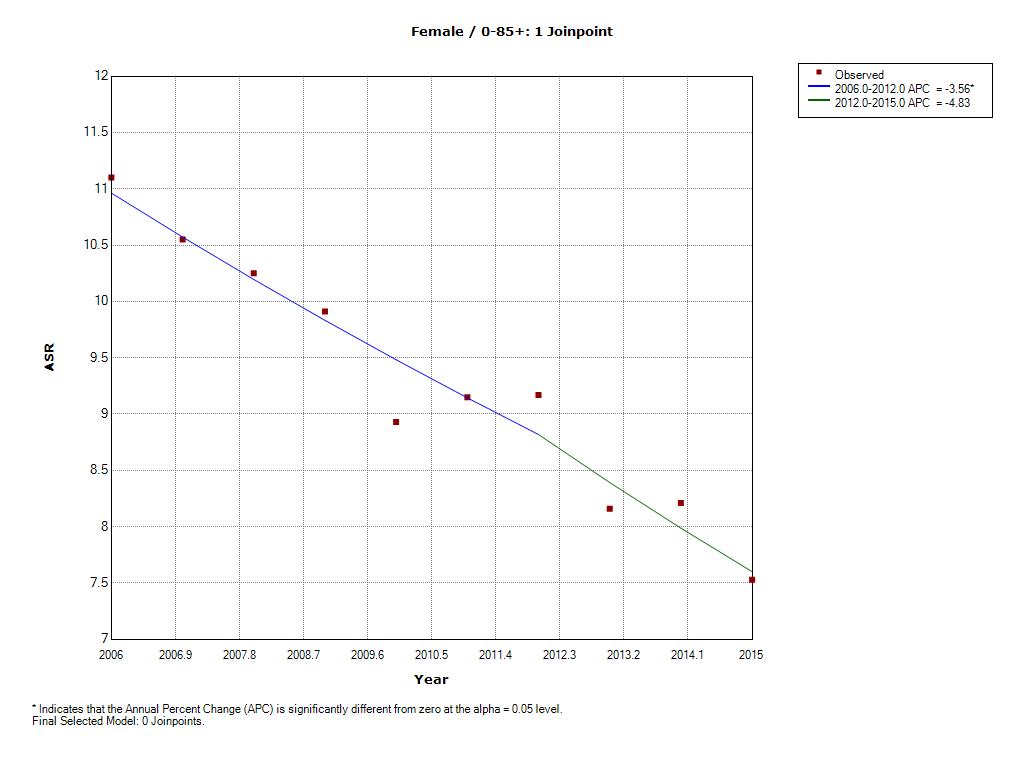

Supplement: Supplementary file 8 — Supplement Figure 8: mortality joinpoint. [file 12889_2024_19104_MOESM8_ESM.zip › Supplement Figure 8 mortality joinpoint/Ecuador female 0-85+.jpg]

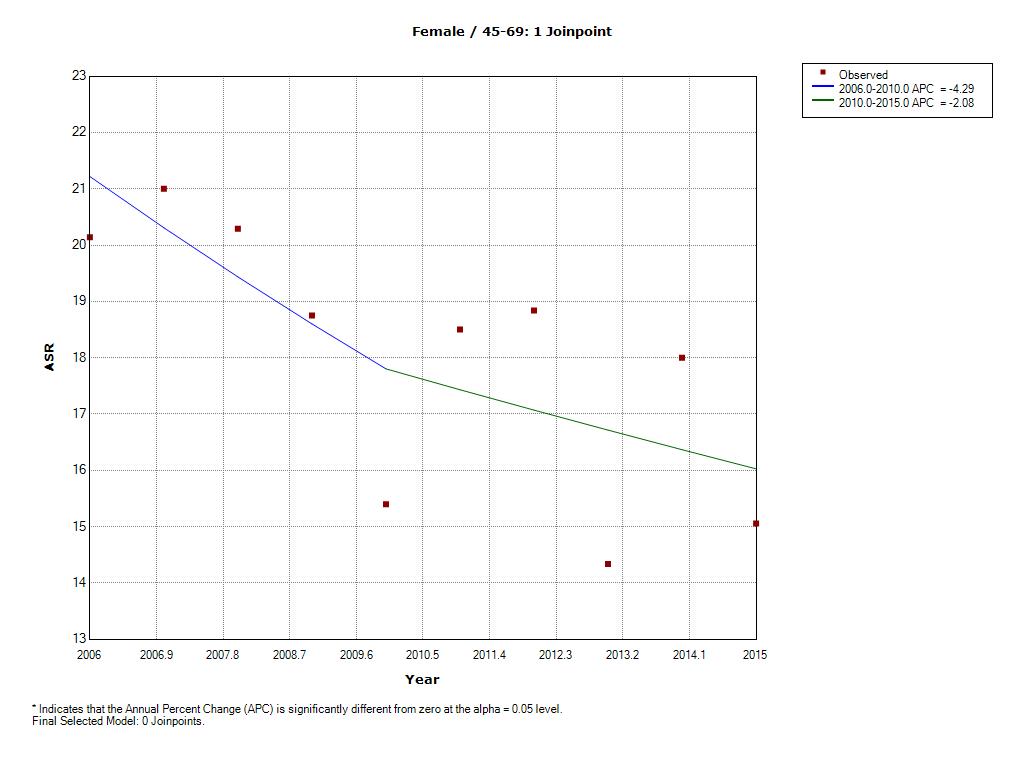

Supplement: Supplementary file 8 — Supplement Figure 8: mortality joinpoint. [file 12889_2024_19104_MOESM8_ESM.zip › Supplement Figure 8 mortality joinpoint/Ecuador female 45-69.jpg]

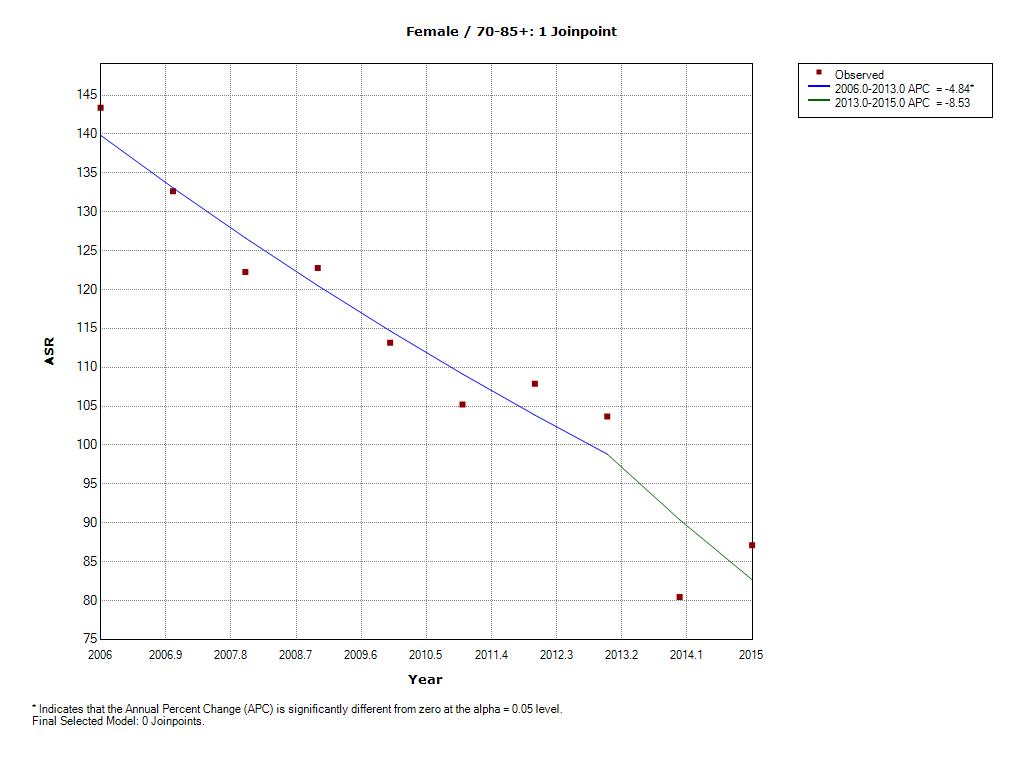

Supplement: Supplementary file 8 — Supplement Figure 8: mortality joinpoint. [file 12889_2024_19104_MOESM8_ESM.zip › Supplement Figure 8 mortality joinpoint/Ecuador female 70-85+.jpg]

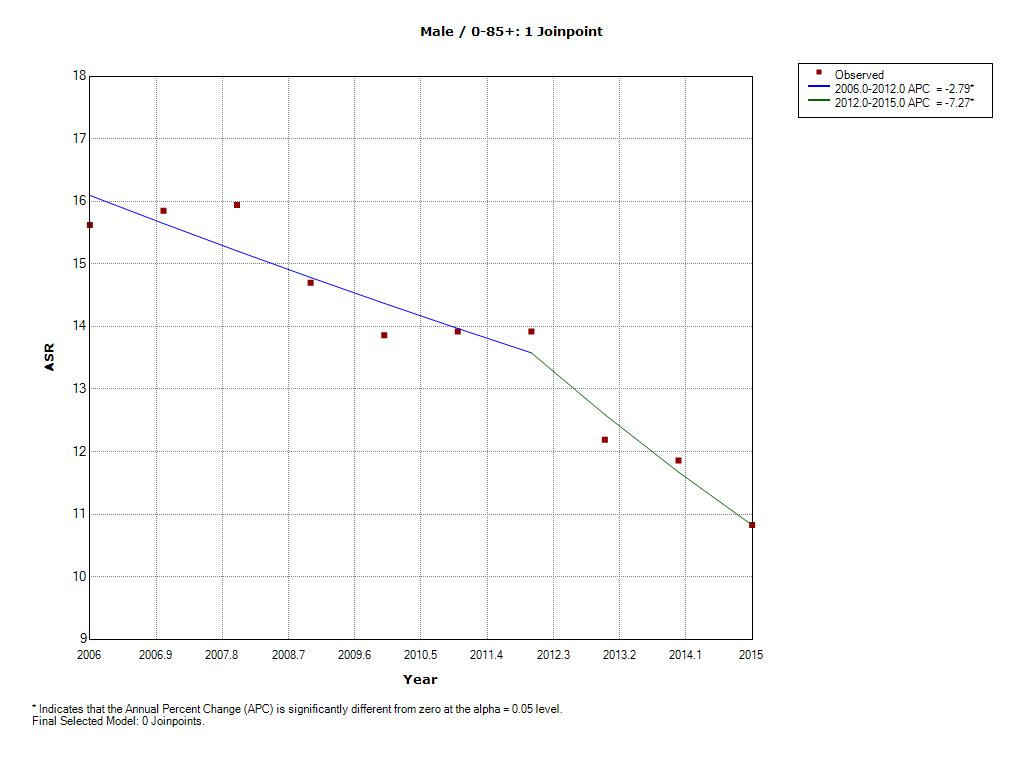

Supplement: Supplementary file 8 — Supplement Figure 8: mortality joinpoint. [file 12889_2024_19104_MOESM8_ESM.zip › Supplement Figure 8 mortality joinpoint/Ecuador male 0-85+.jpg]

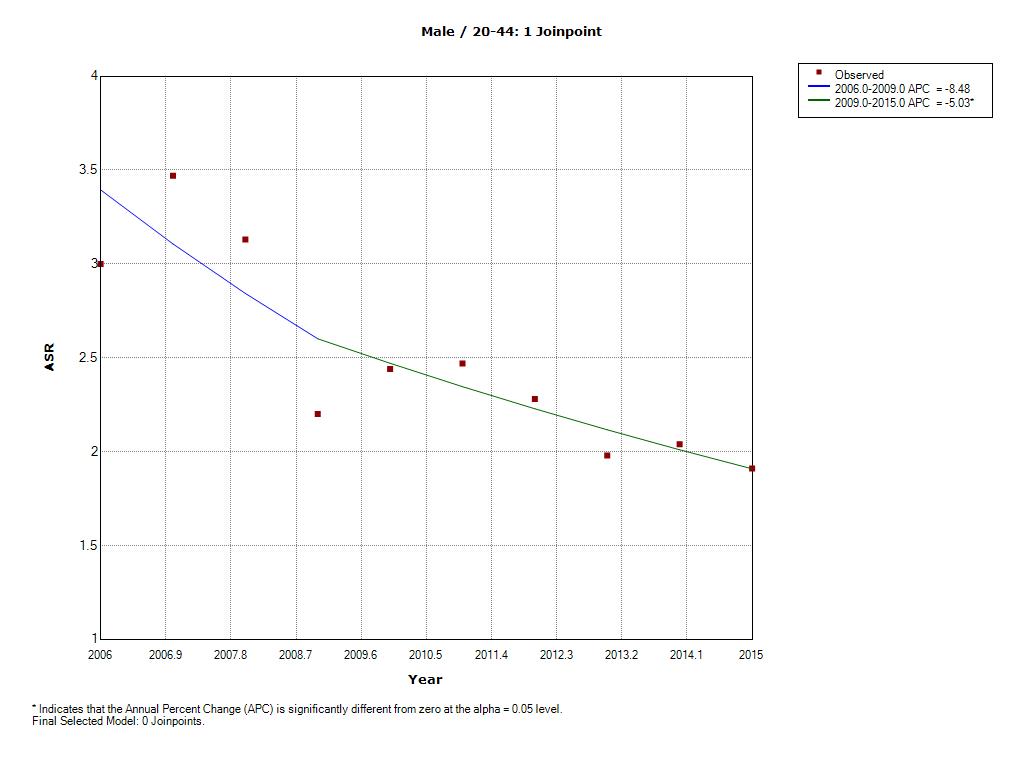

Supplement: Supplementary file 8 — Supplement Figure 8: mortality joinpoint. [file 12889_2024_19104_MOESM8_ESM.zip › Supplement Figure 8 mortality joinpoint/Ecuador male 20-44.jpg]

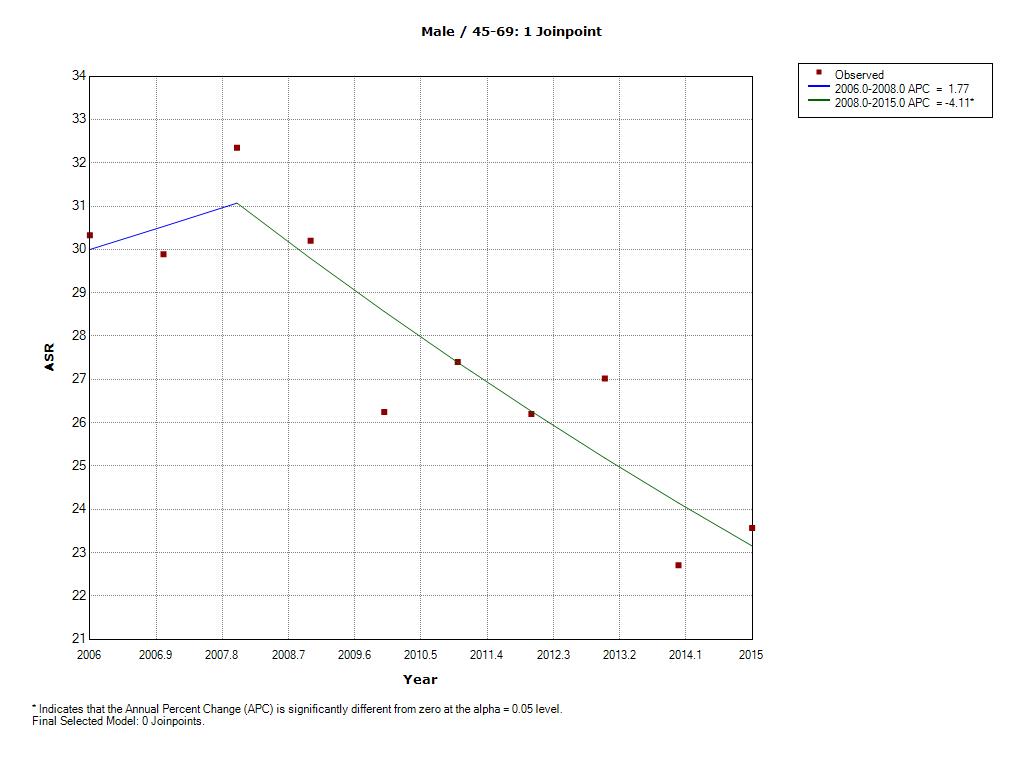

Supplement: Supplementary file 8 — Supplement Figure 8: mortality joinpoint. [file 12889_2024_19104_MOESM8_ESM.zip › Supplement Figure 8 mortality joinpoint/Ecuador male 45-69.jpg]

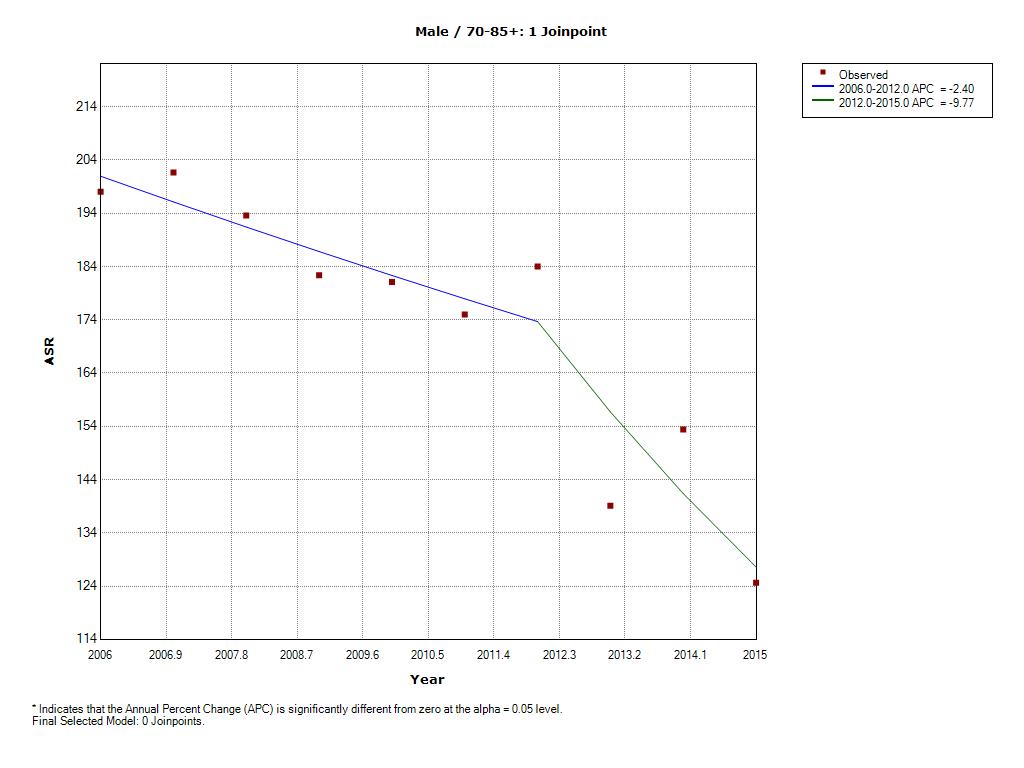

Supplement: Supplementary file 8 — Supplement Figure 8: mortality joinpoint. [file 12889_2024_19104_MOESM8_ESM.zip › Supplement Figure 8 mortality joinpoint/Ecuador male 70-85+.jpg]
