# Supplementary figures and images for: Global incidence and mortality trends of gastric cancer and predicted mortality of gastric cancer by 2035 (part 2 of 4)
Source: BMC Public Health. 2024 Jul 2;24:1763. doi: 10.1186/s12889-024-19104-6 (PMC11221210; doi:10.1186/s12889-024-19104-6)

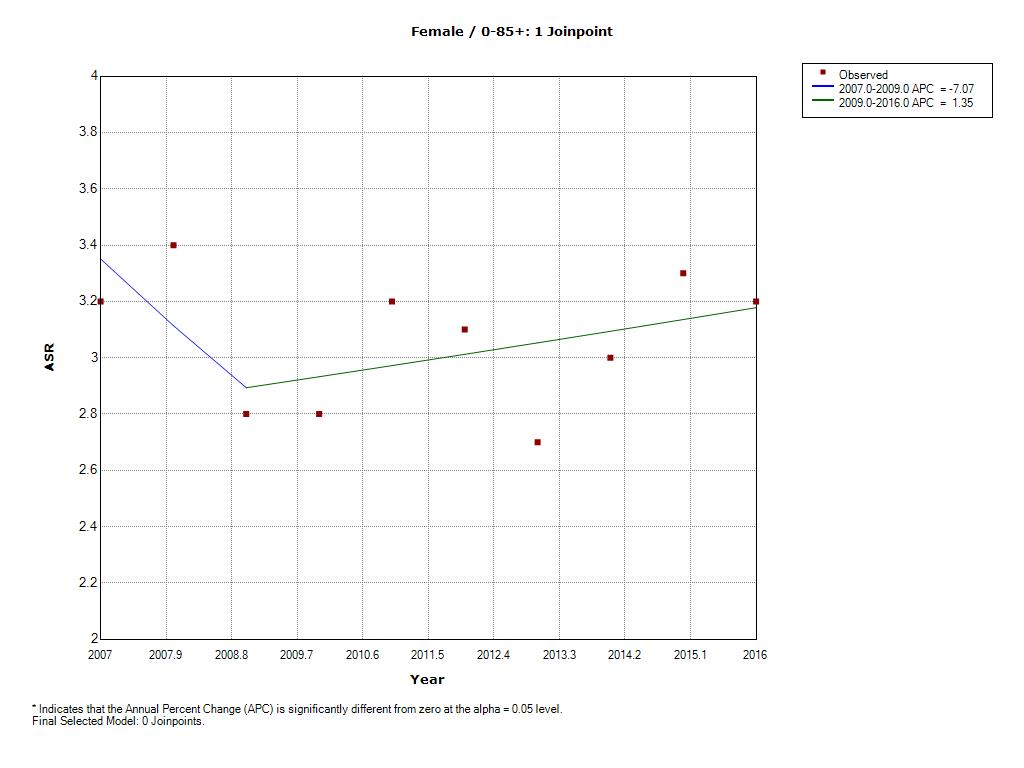

Supplement: Supplementary file 7 — Supplement Figure 7: incidence joinpoint. [file 12889_2024_19104_MOESM7_ESM.zip › Supplement Figure 7 incidence joinpoint/Denmark female 0-85+.jpg]

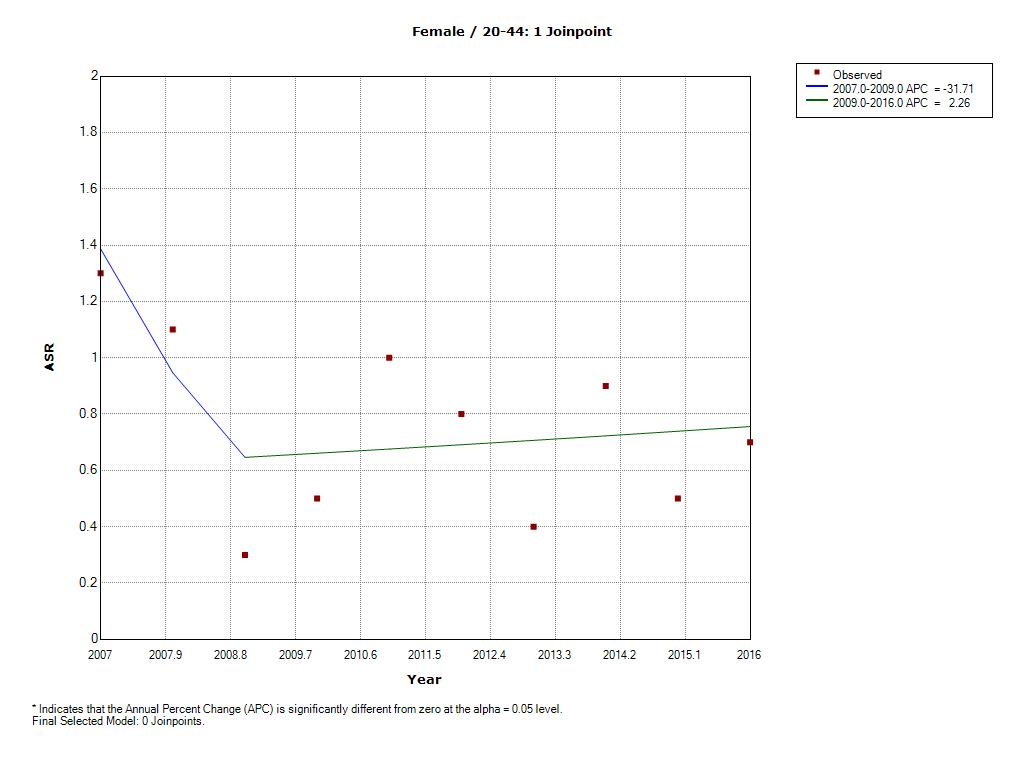

Supplement: Supplementary file 7 — Supplement Figure 7: incidence joinpoint. [file 12889_2024_19104_MOESM7_ESM.zip › Supplement Figure 7 incidence joinpoint/Denmark female 20-44.jpg]

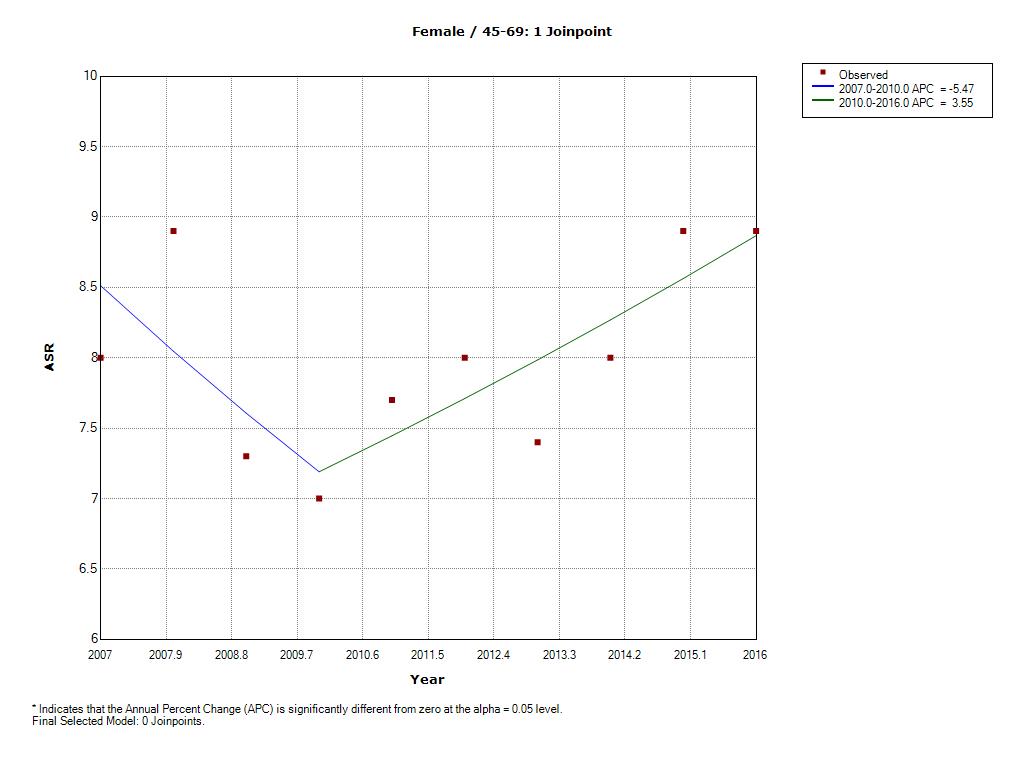

Supplement: Supplementary file 7 — Supplement Figure 7: incidence joinpoint. [file 12889_2024_19104_MOESM7_ESM.zip › Supplement Figure 7 incidence joinpoint/Denmark female 45-69.jpg]

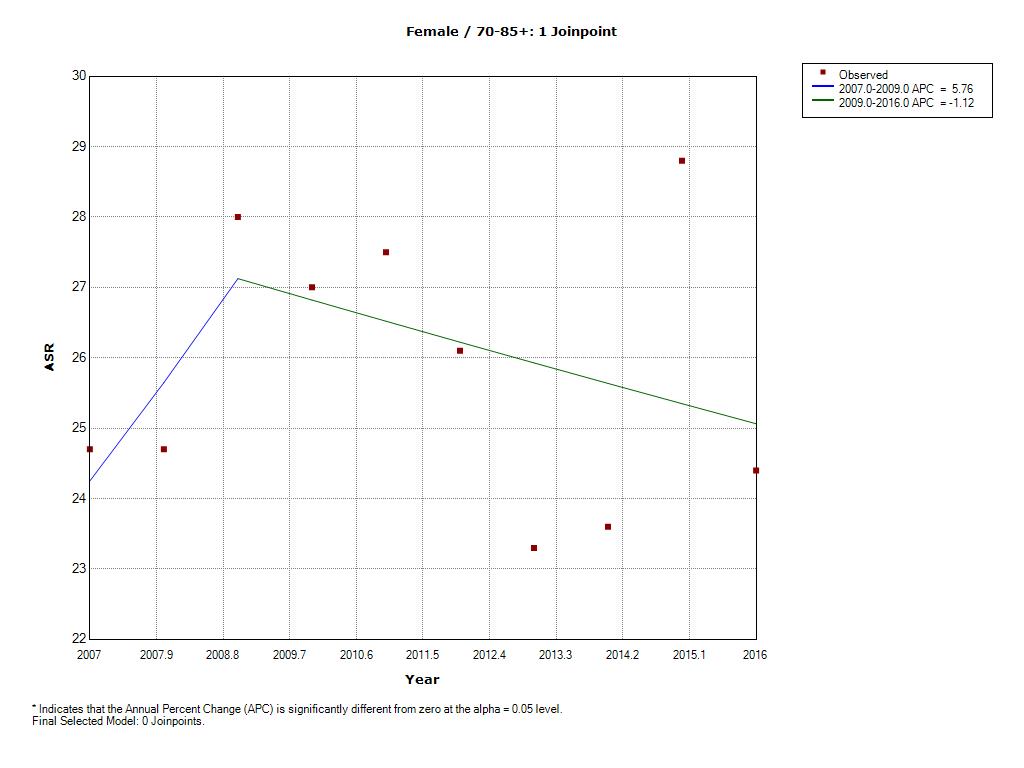

Supplement: Supplementary file 7 — Supplement Figure 7: incidence joinpoint. [file 12889_2024_19104_MOESM7_ESM.zip › Supplement Figure 7 incidence joinpoint/Denmark female 70-85+.jpg]

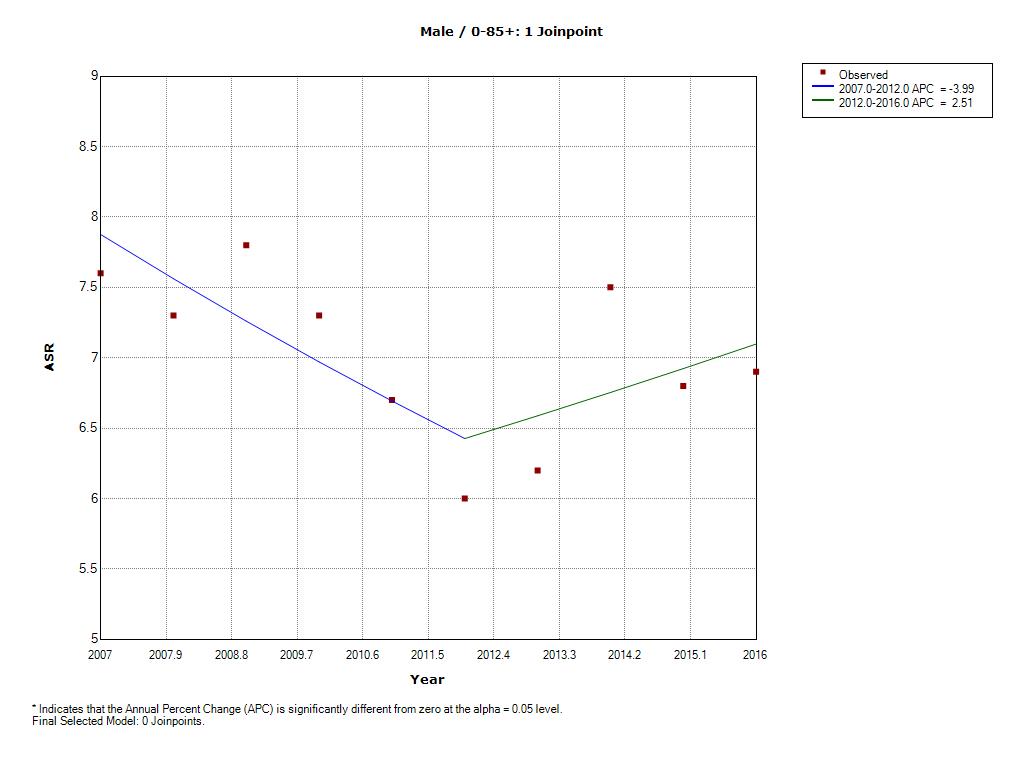

Supplement: Supplementary file 7 — Supplement Figure 7: incidence joinpoint. [file 12889_2024_19104_MOESM7_ESM.zip › Supplement Figure 7 incidence joinpoint/Denmark male 0-85+.jpg]

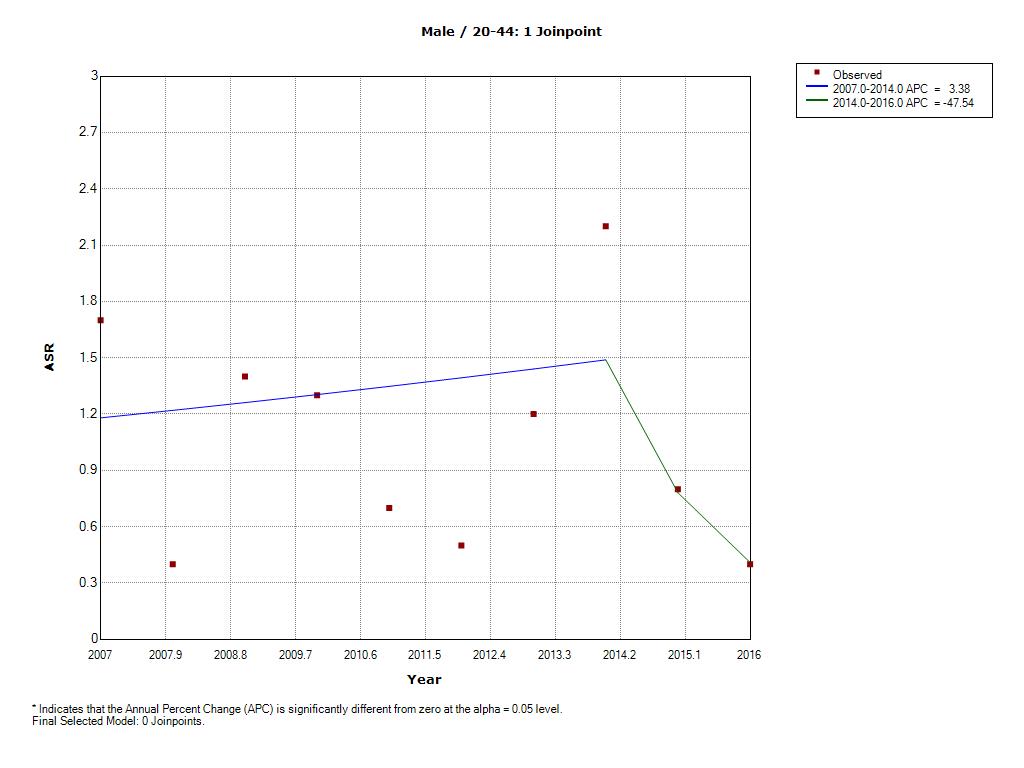

Supplement: Supplementary file 7 — Supplement Figure 7: incidence joinpoint. [file 12889_2024_19104_MOESM7_ESM.zip › Supplement Figure 7 incidence joinpoint/Denmark male 20-44.jpg]

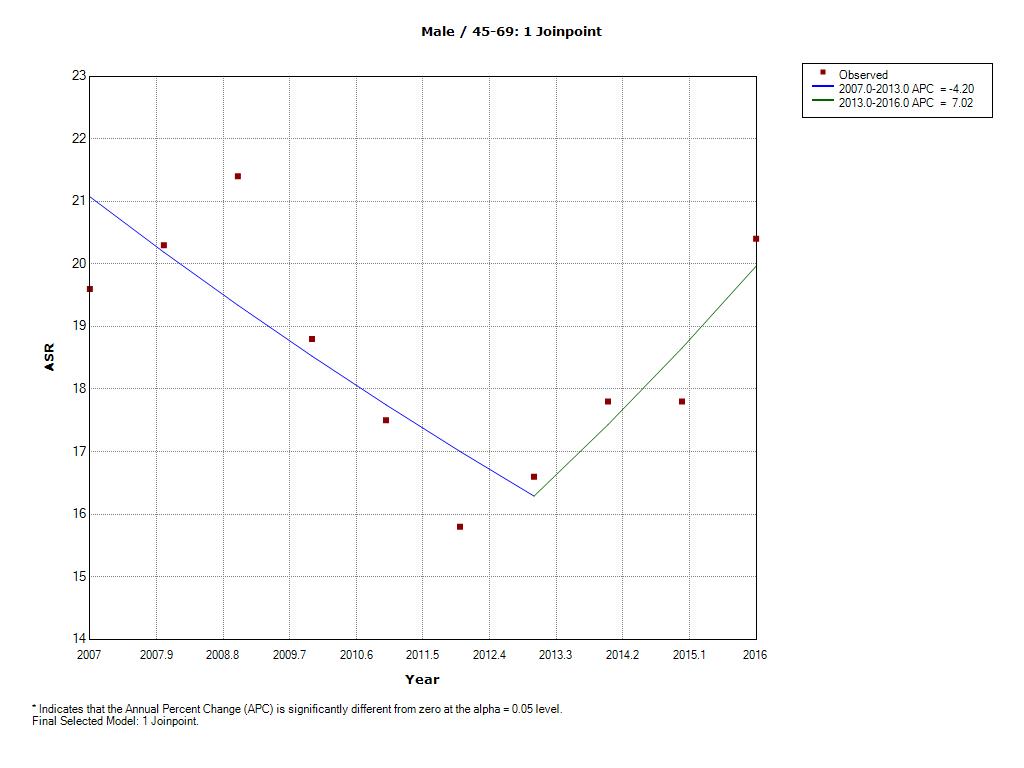

Supplement: Supplementary file 7 — Supplement Figure 7: incidence joinpoint. [file 12889_2024_19104_MOESM7_ESM.zip › Supplement Figure 7 incidence joinpoint/Denmark male 45-69.jpg]

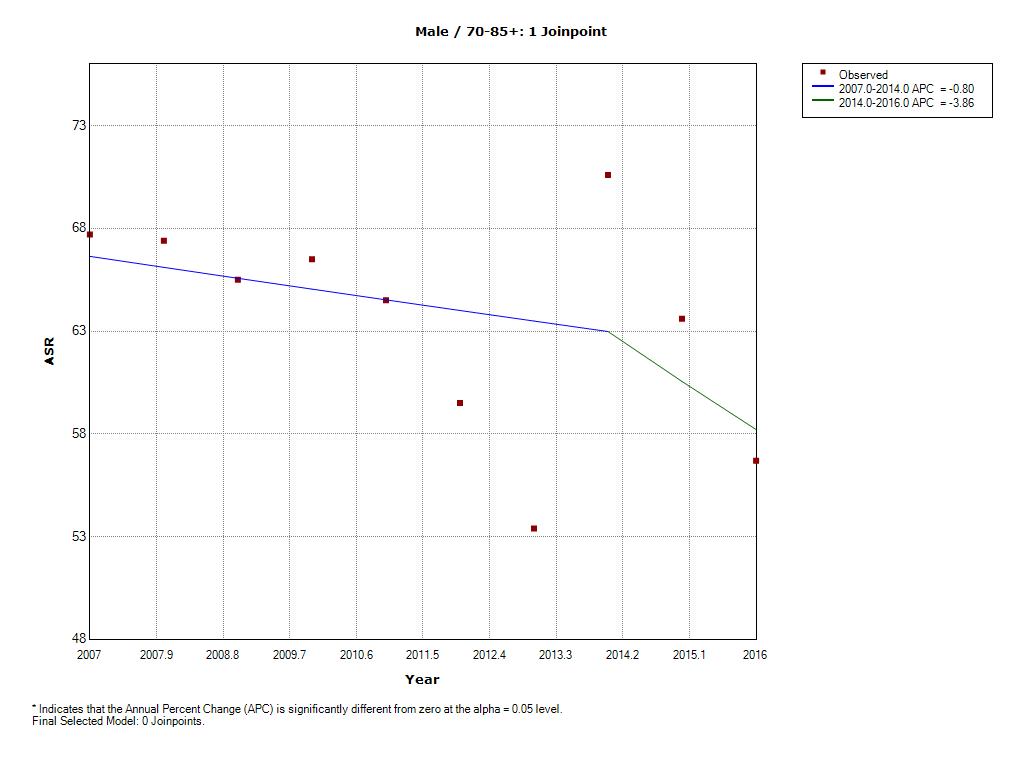

Supplement: Supplementary file 7 — Supplement Figure 7: incidence joinpoint. [file 12889_2024_19104_MOESM7_ESM.zip › Supplement Figure 7 incidence joinpoint/Denmark male 70-85+.jpg]

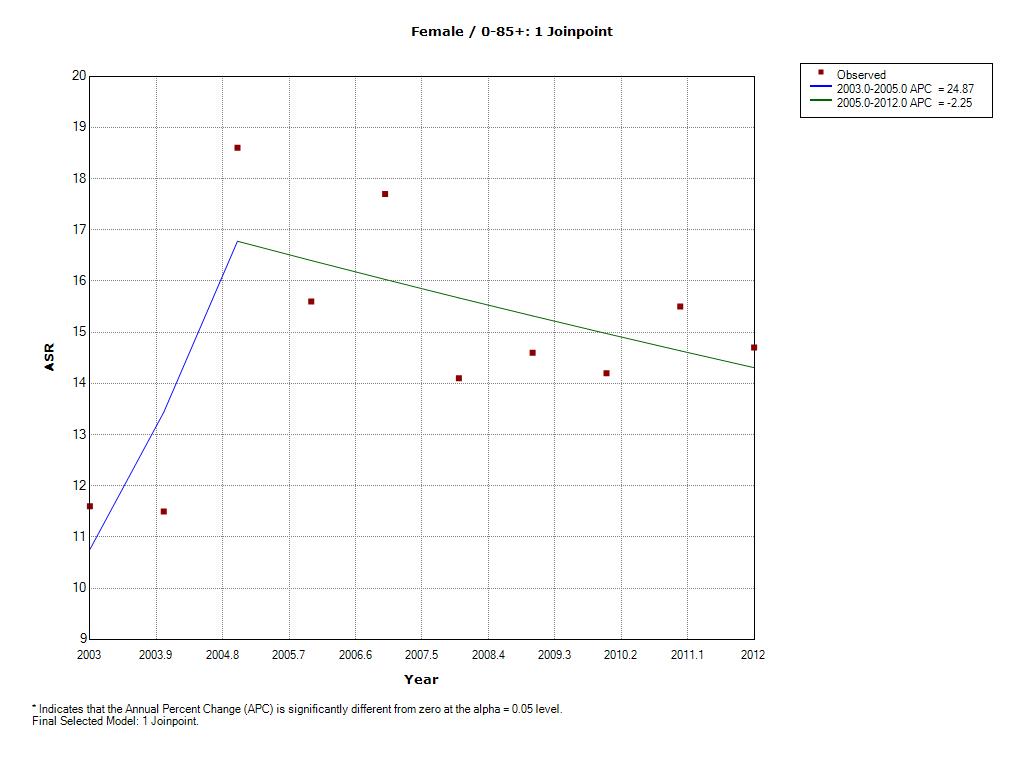

Supplement: Supplementary file 7 — Supplement Figure 7: incidence joinpoint. [file 12889_2024_19104_MOESM7_ESM.zip › Supplement Figure 7 incidence joinpoint/Ecuador female 0-85+.jpg]

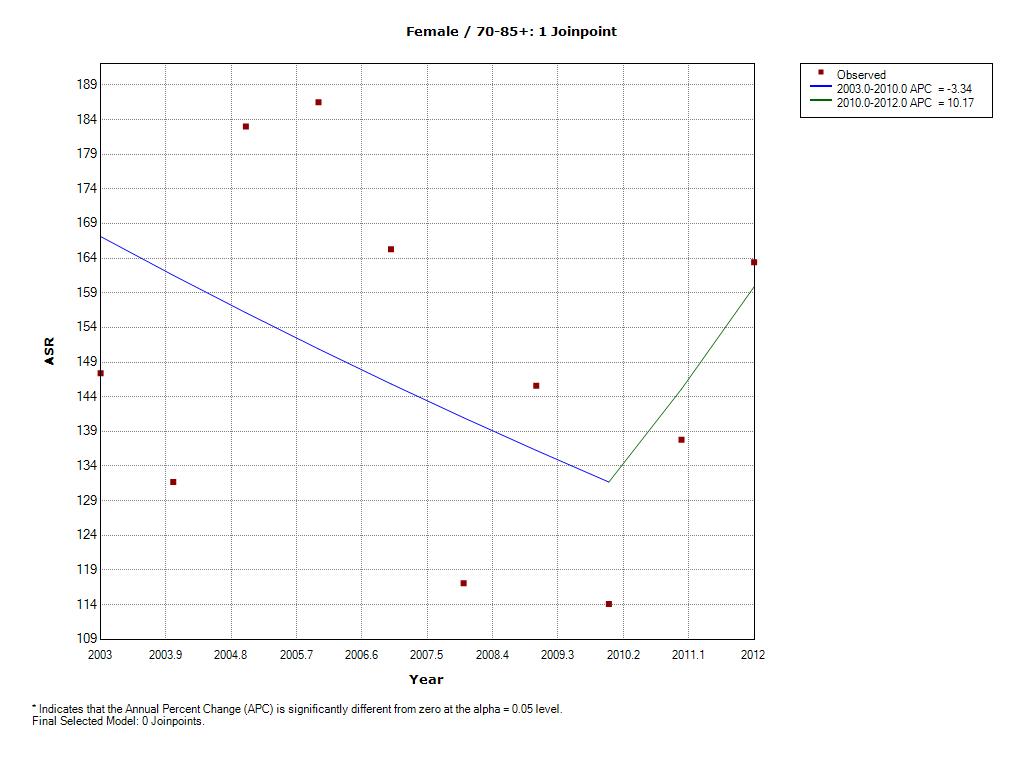

Supplement: Supplementary file 7 — Supplement Figure 7: incidence joinpoint. [file 12889_2024_19104_MOESM7_ESM.zip › Supplement Figure 7 incidence joinpoint/Ecuador female 120-85+.jpg]

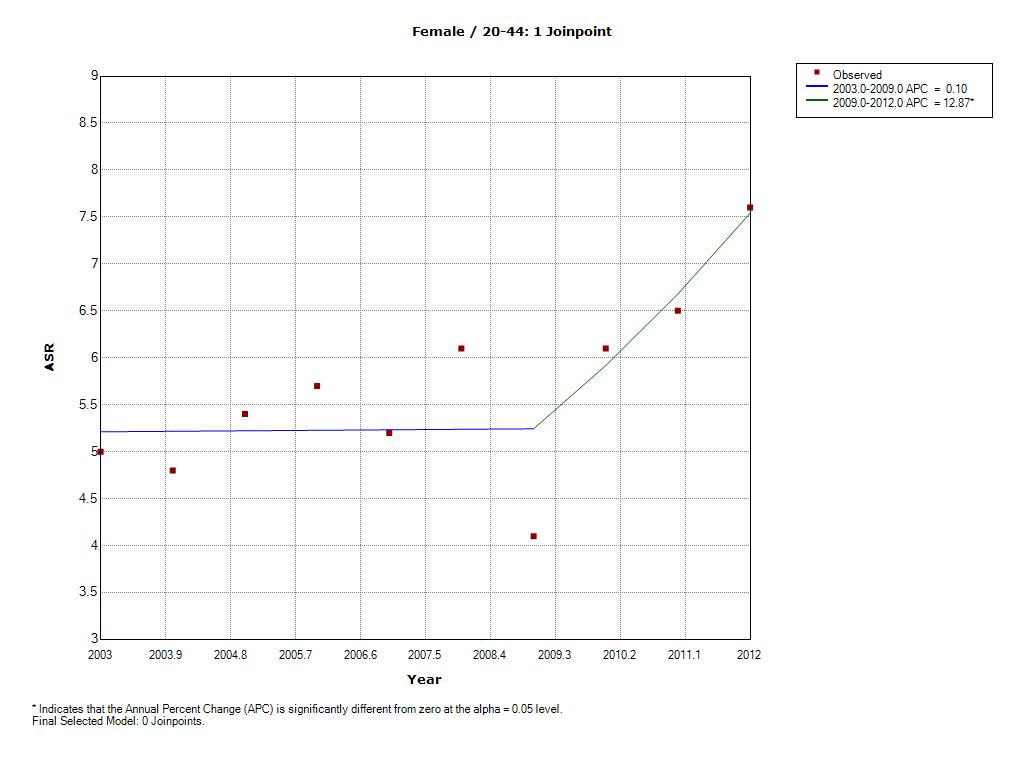

Supplement: Supplementary file 7 — Supplement Figure 7: incidence joinpoint. [file 12889_2024_19104_MOESM7_ESM.zip › Supplement Figure 7 incidence joinpoint/Ecuador female 20-44.jpg]

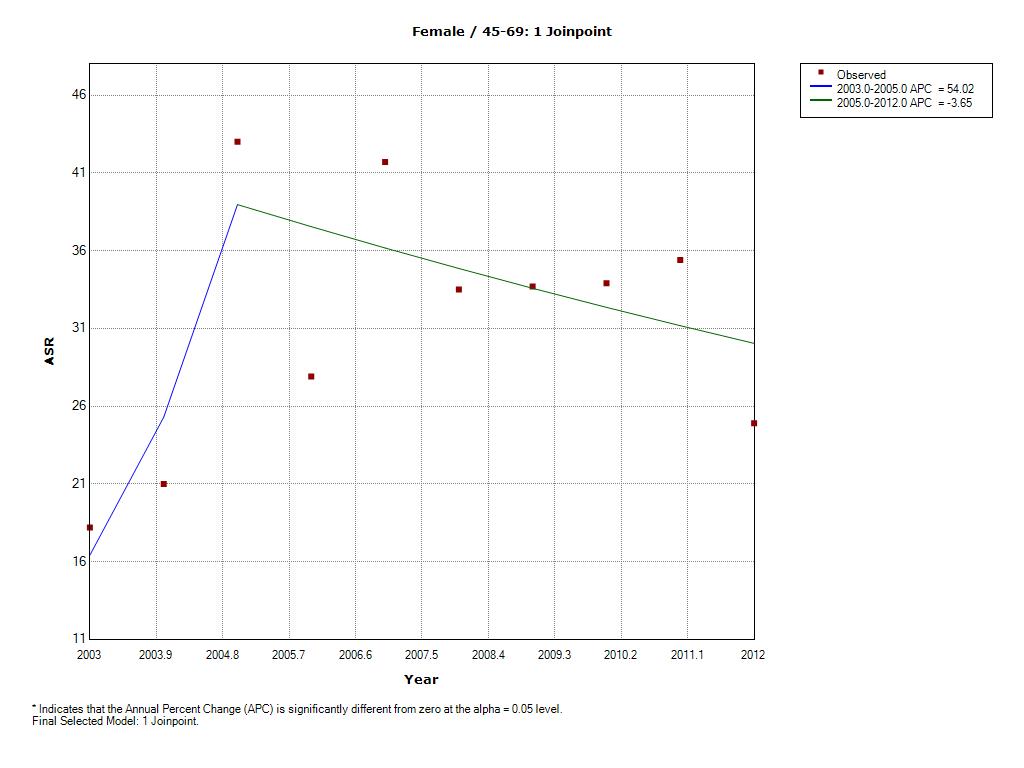

Supplement: Supplementary file 7 — Supplement Figure 7: incidence joinpoint. [file 12889_2024_19104_MOESM7_ESM.zip › Supplement Figure 7 incidence joinpoint/Ecuador female 45-69.jpg]

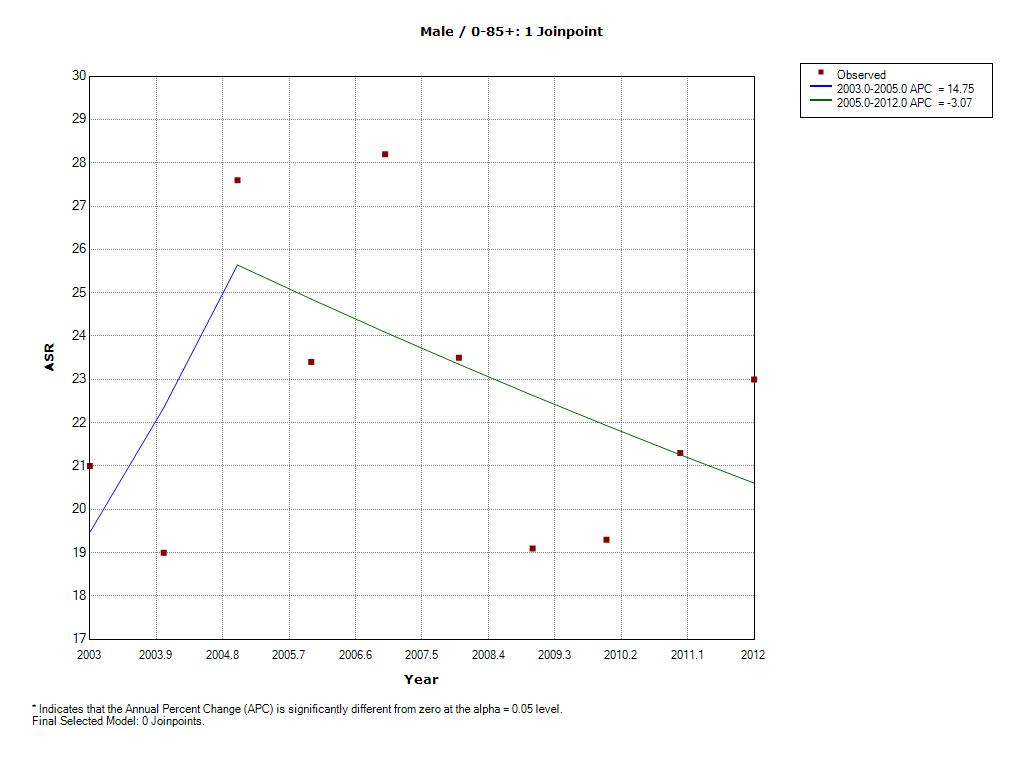

Supplement: Supplementary file 7 — Supplement Figure 7: incidence joinpoint. [file 12889_2024_19104_MOESM7_ESM.zip › Supplement Figure 7 incidence joinpoint/Ecuador male 0-85+.jpg]

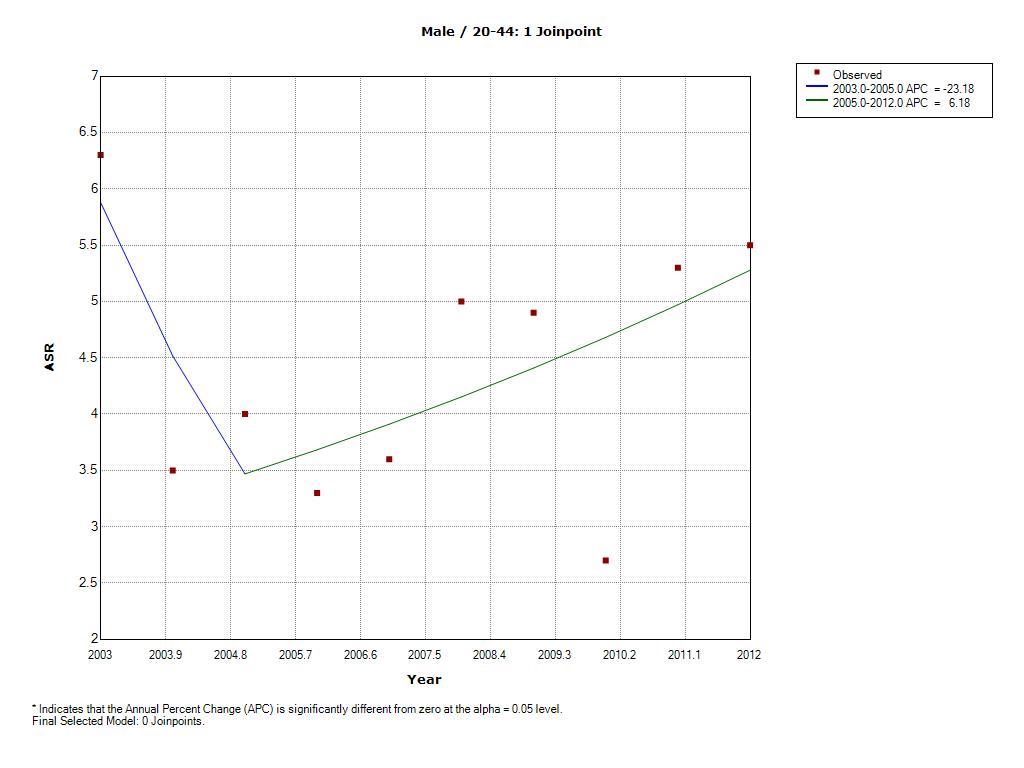

Supplement: Supplementary file 7 — Supplement Figure 7: incidence joinpoint. [file 12889_2024_19104_MOESM7_ESM.zip › Supplement Figure 7 incidence joinpoint/Ecuador male 20-44.jpg]

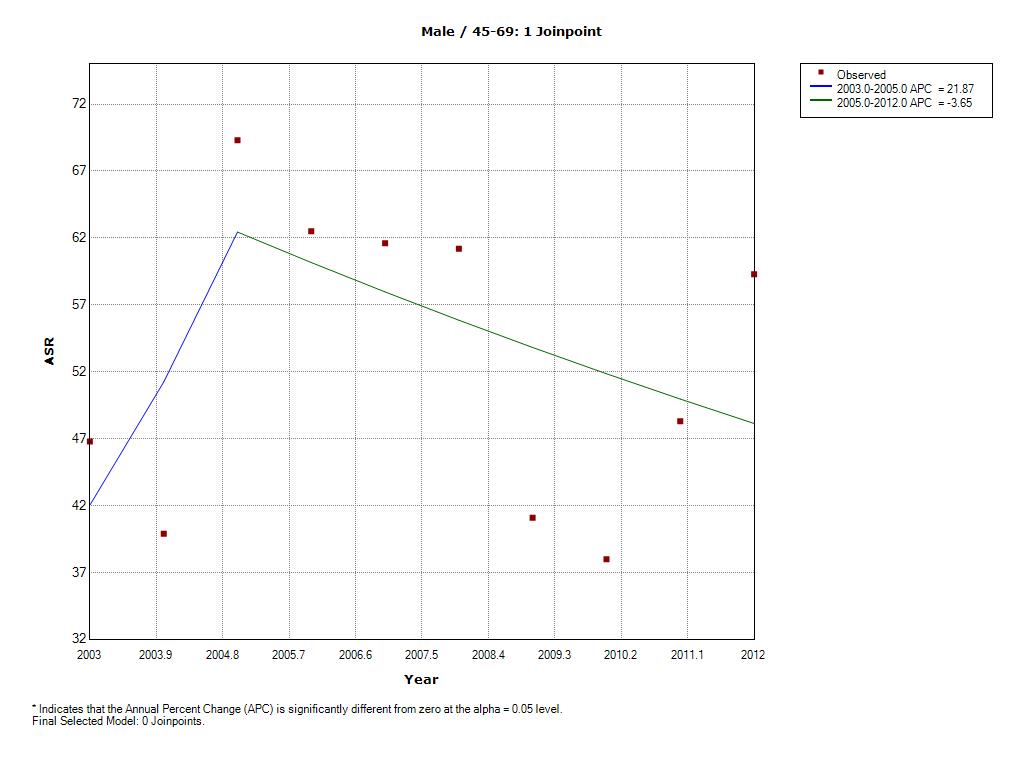

Supplement: Supplementary file 7 — Supplement Figure 7: incidence joinpoint. [file 12889_2024_19104_MOESM7_ESM.zip › Supplement Figure 7 incidence joinpoint/Ecuador male 45-69.jpg]

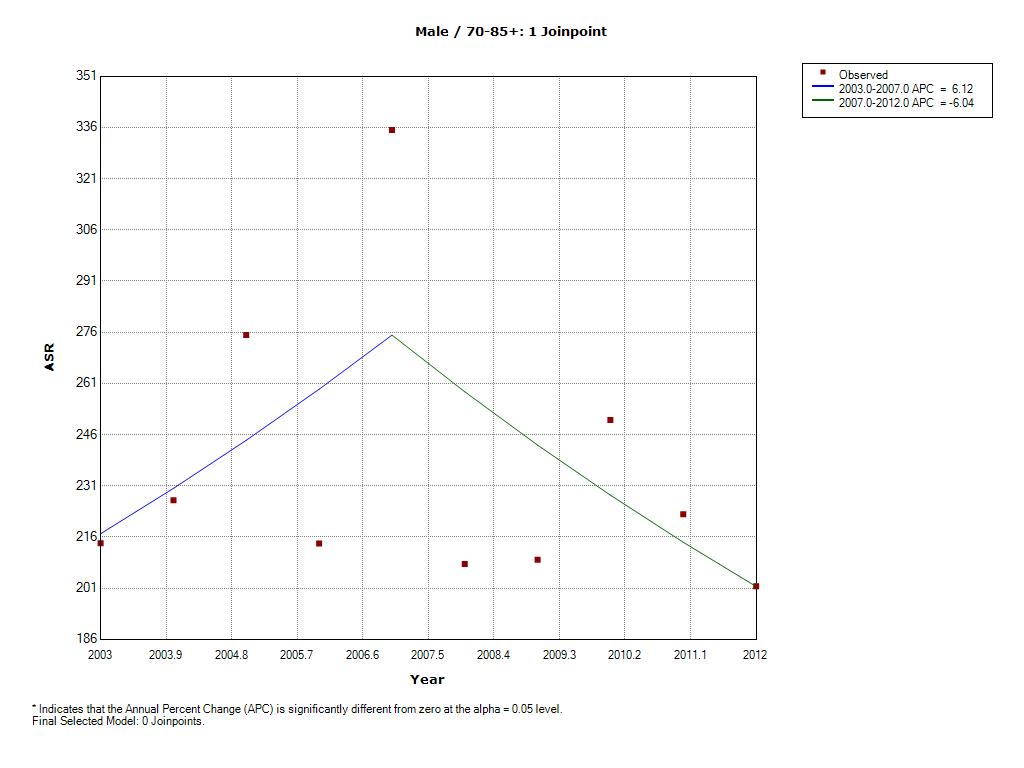

Supplement: Supplementary file 7 — Supplement Figure 7: incidence joinpoint. [file 12889_2024_19104_MOESM7_ESM.zip › Supplement Figure 7 incidence joinpoint/Ecuador male 70-85+.jpg]

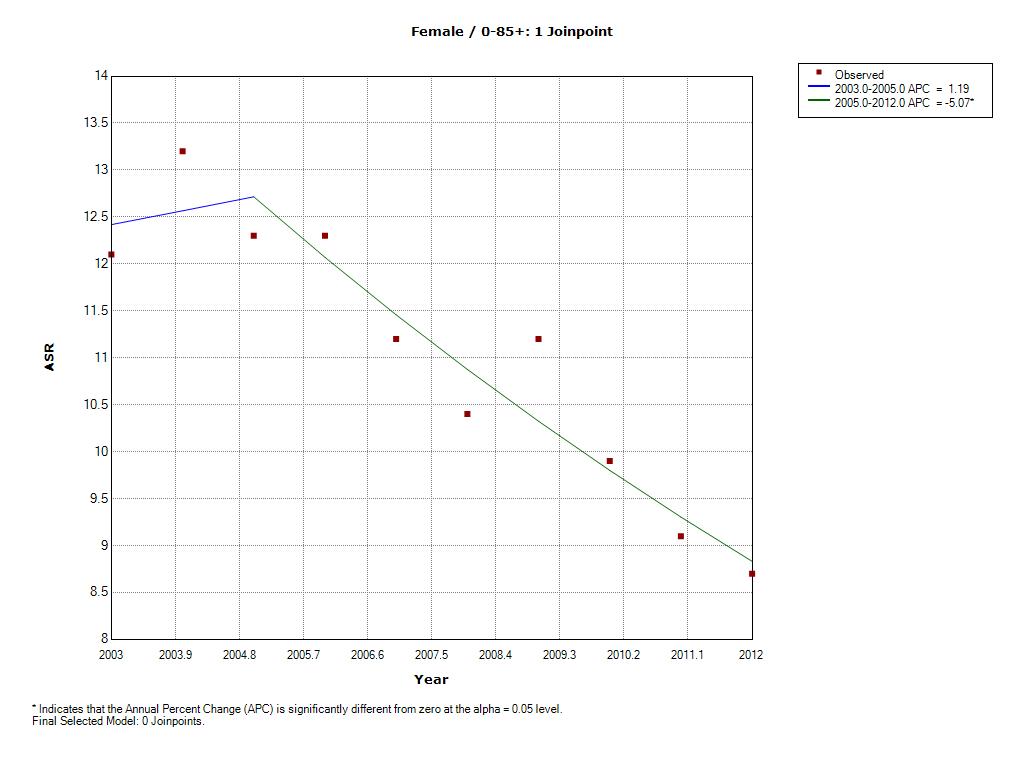

Supplement: Supplementary file 7 — Supplement Figure 7: incidence joinpoint. [file 12889_2024_19104_MOESM7_ESM.zip › Supplement Figure 7 incidence joinpoint/Estonia female 0-85+.jpg]

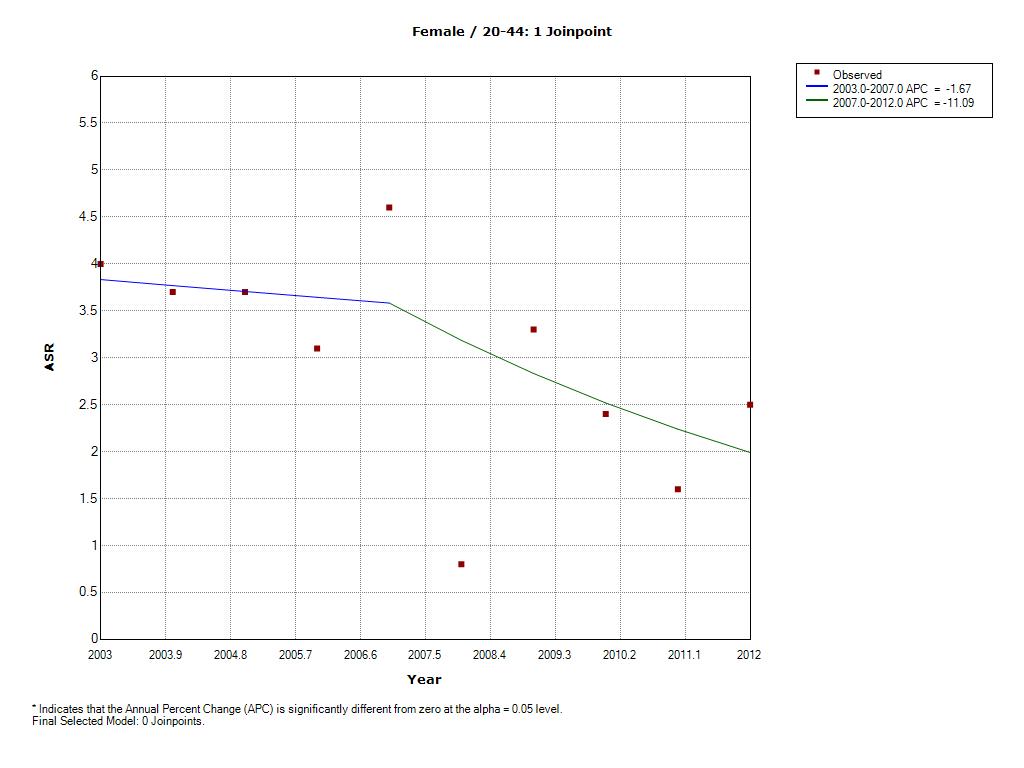

Supplement: Supplementary file 7 — Supplement Figure 7: incidence joinpoint. [file 12889_2024_19104_MOESM7_ESM.zip › Supplement Figure 7 incidence joinpoint/Estonia female 20-44.jpg]

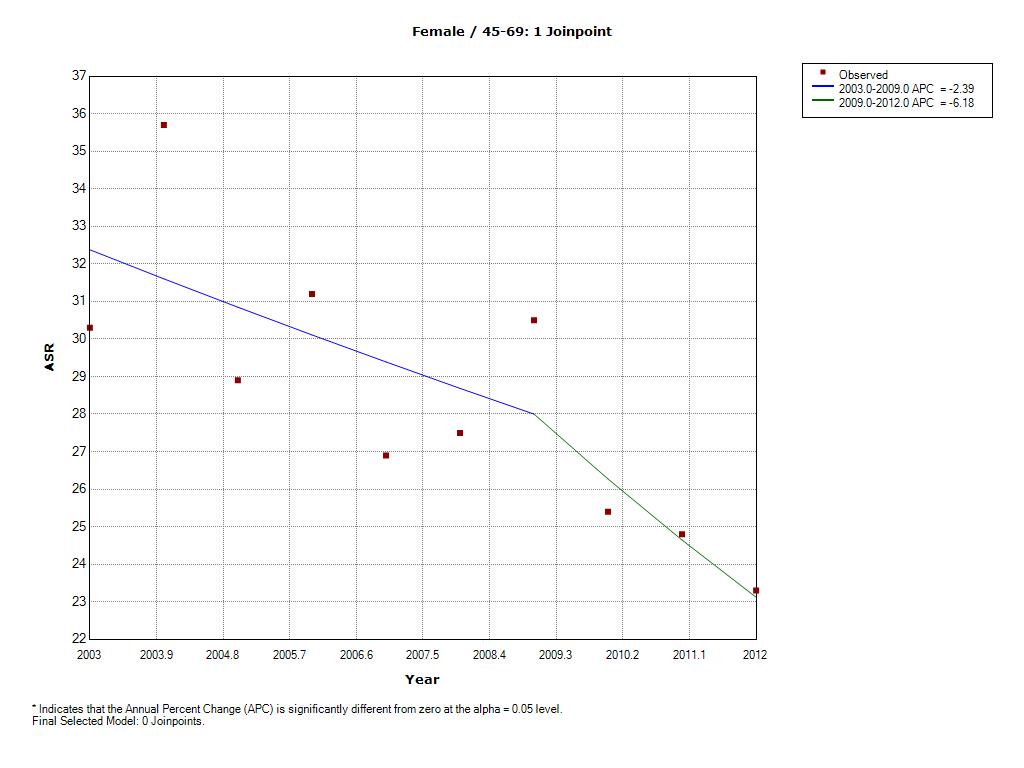

Supplement: Supplementary file 7 — Supplement Figure 7: incidence joinpoint. [file 12889_2024_19104_MOESM7_ESM.zip › Supplement Figure 7 incidence joinpoint/Estonia female 45-69.jpg]

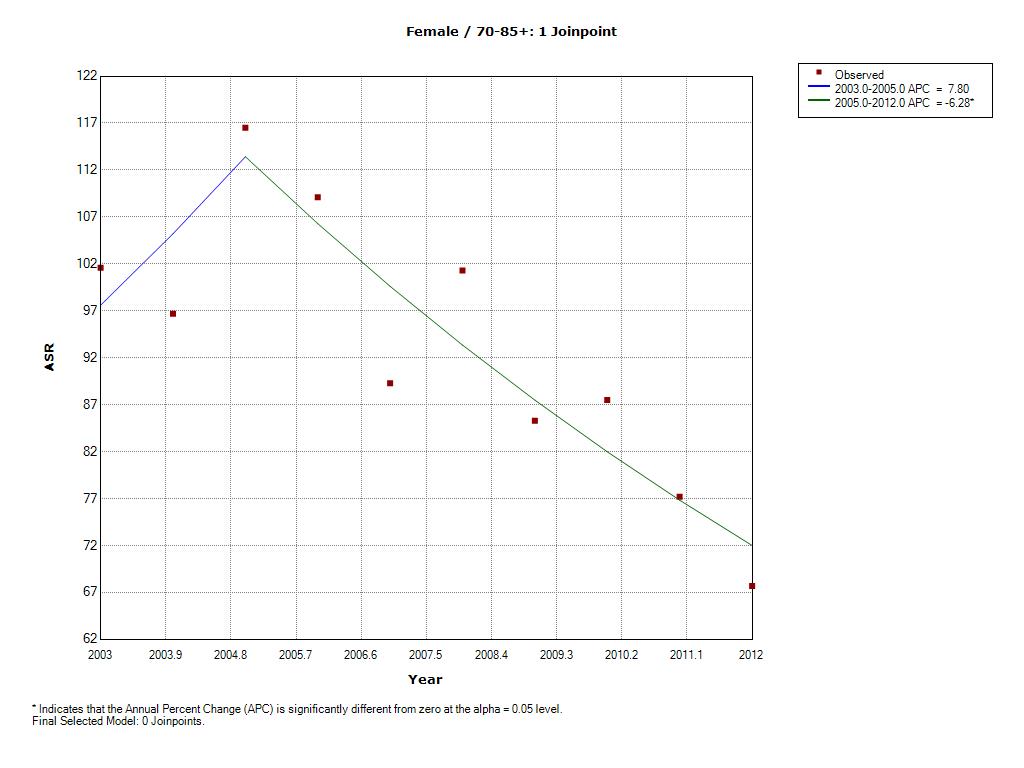

Supplement: Supplementary file 7 — Supplement Figure 7: incidence joinpoint. [file 12889_2024_19104_MOESM7_ESM.zip › Supplement Figure 7 incidence joinpoint/Estonia female 70-85+.jpg]

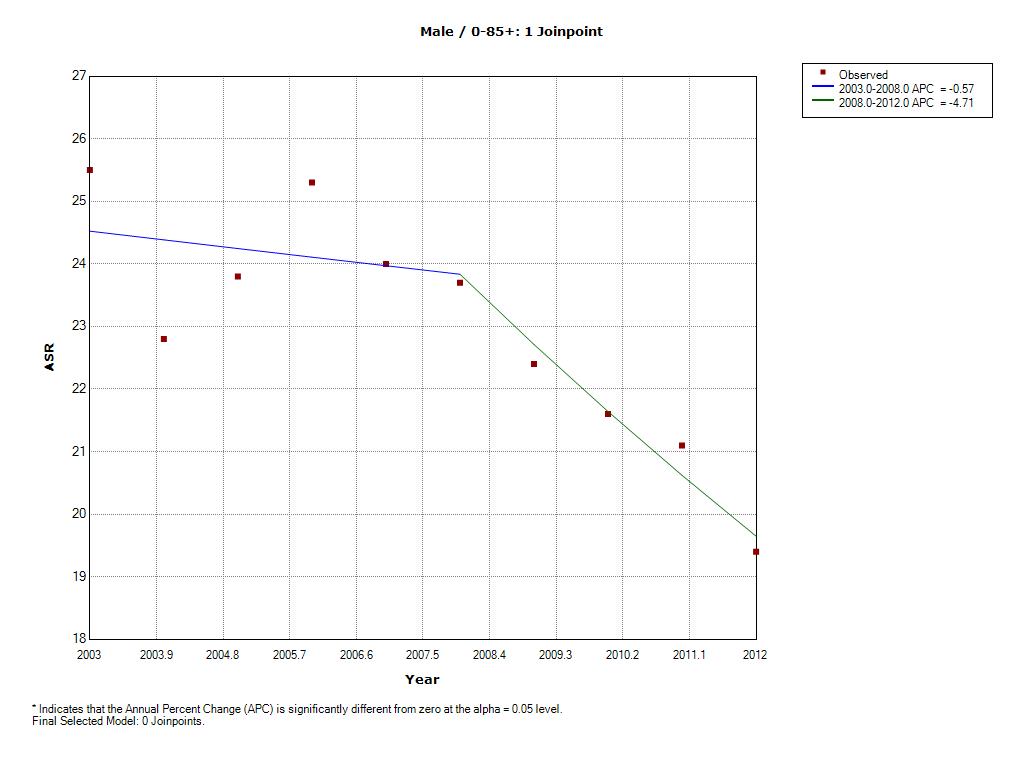

Supplement: Supplementary file 7 — Supplement Figure 7: incidence joinpoint. [file 12889_2024_19104_MOESM7_ESM.zip › Supplement Figure 7 incidence joinpoint/Estonia male 0-85+.jpg]

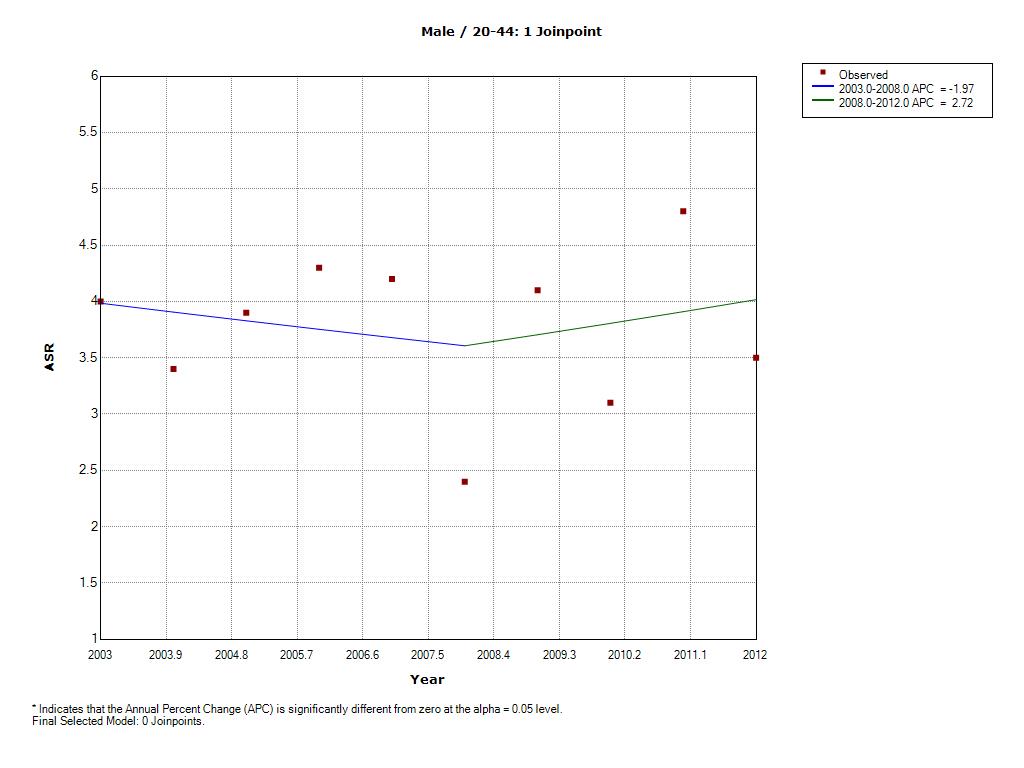

Supplement: Supplementary file 7 — Supplement Figure 7: incidence joinpoint. [file 12889_2024_19104_MOESM7_ESM.zip › Supplement Figure 7 incidence joinpoint/Estonia male 20-44.jpg]

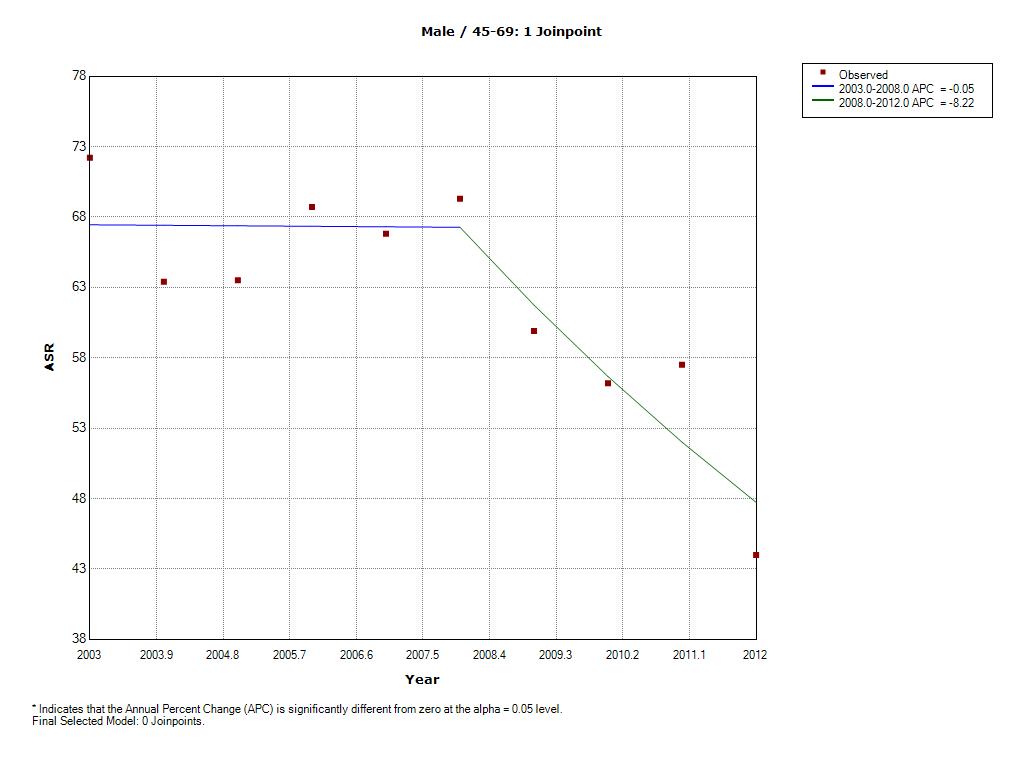

Supplement: Supplementary file 7 — Supplement Figure 7: incidence joinpoint. [file 12889_2024_19104_MOESM7_ESM.zip › Supplement Figure 7 incidence joinpoint/Estonia male 45-69.jpg]

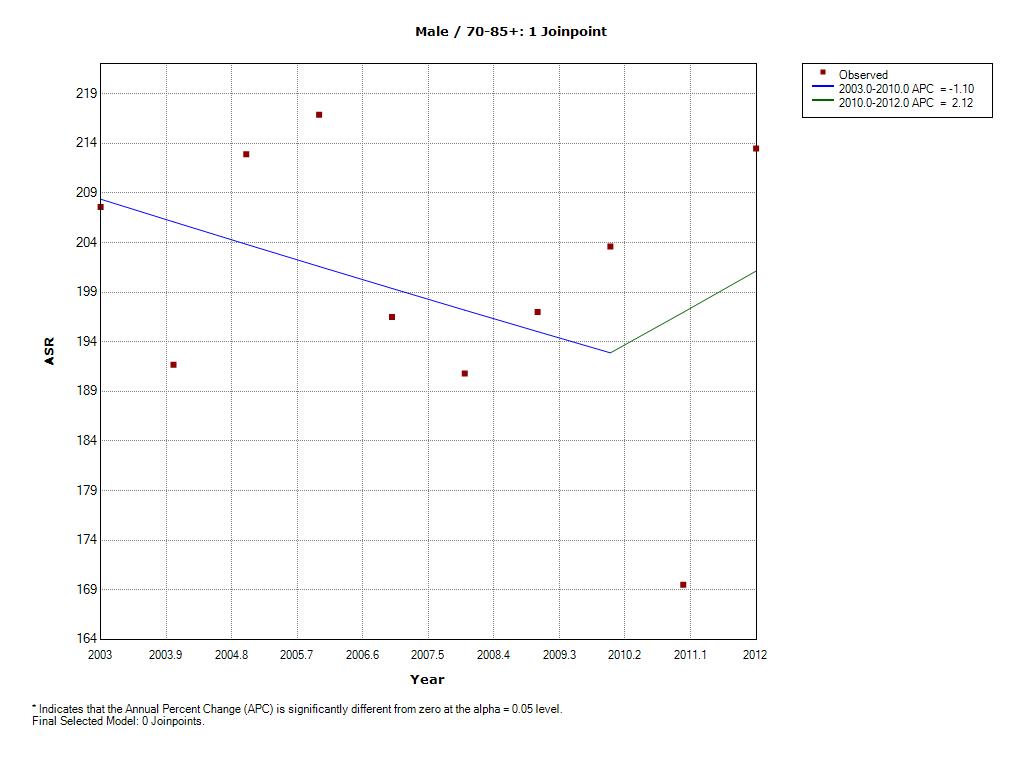

Supplement: Supplementary file 7 — Supplement Figure 7: incidence joinpoint. [file 12889_2024_19104_MOESM7_ESM.zip › Supplement Figure 7 incidence joinpoint/Estonia male 70-85+.jpg]

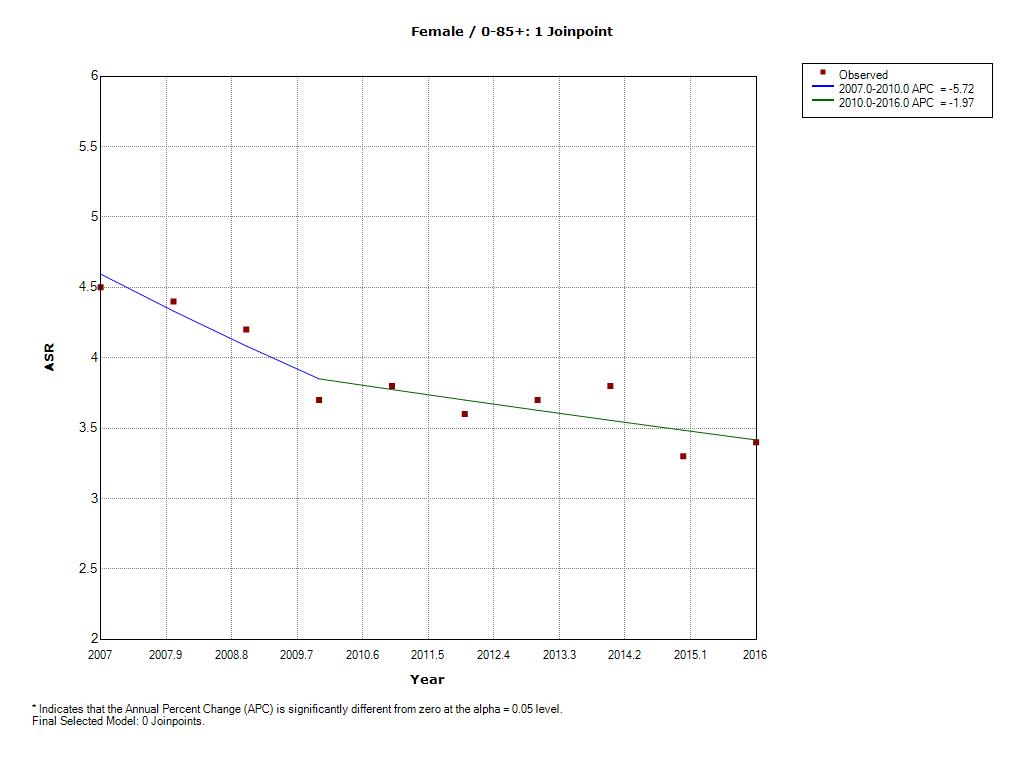

Supplement: Supplementary file 7 — Supplement Figure 7: incidence joinpoint. [file 12889_2024_19104_MOESM7_ESM.zip › Supplement Figure 7 incidence joinpoint/Finland female 0-85+.jpg]

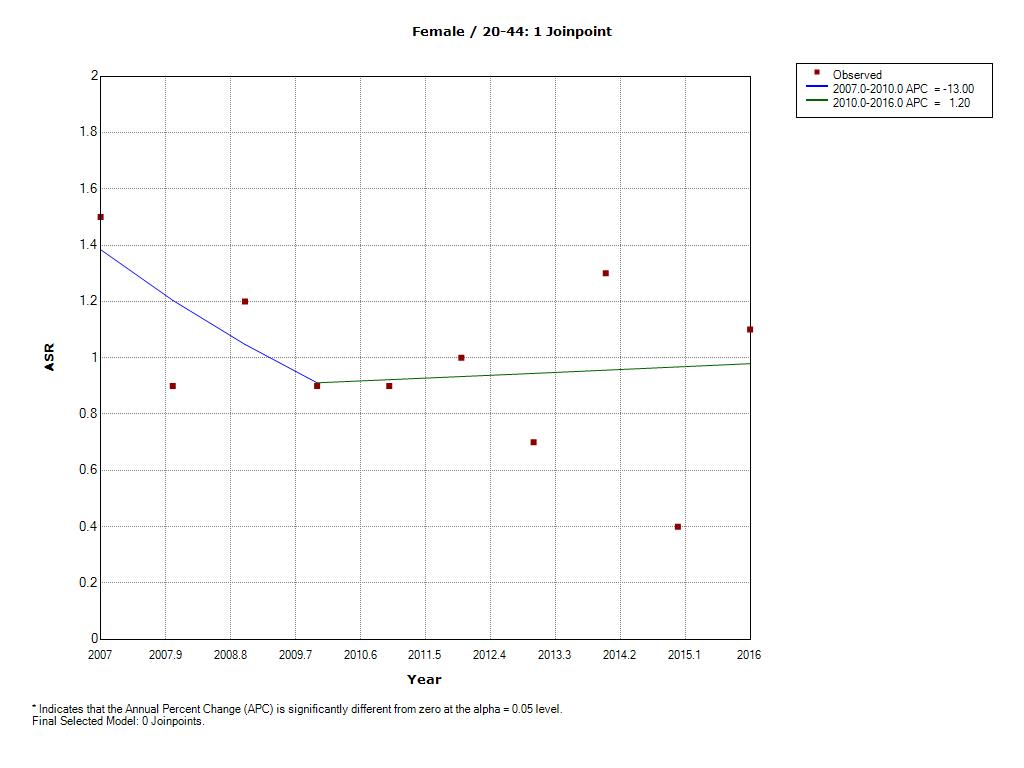

Supplement: Supplementary file 7 — Supplement Figure 7: incidence joinpoint. [file 12889_2024_19104_MOESM7_ESM.zip › Supplement Figure 7 incidence joinpoint/Finland female 20-44.jpg]

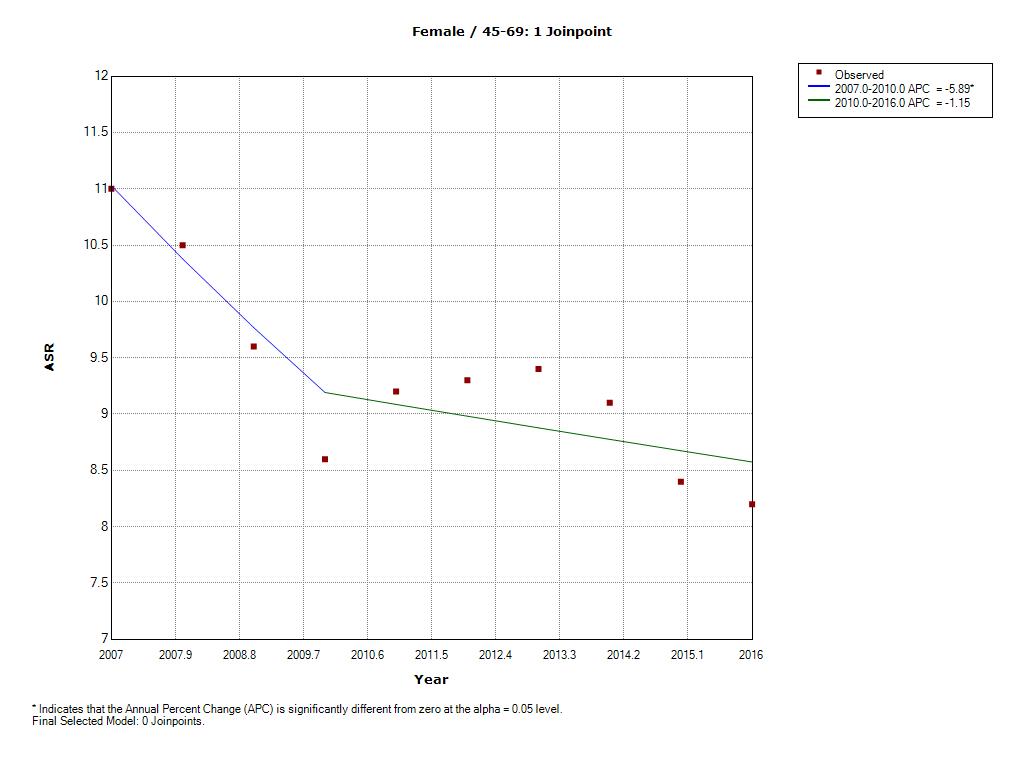

Supplement: Supplementary file 7 — Supplement Figure 7: incidence joinpoint. [file 12889_2024_19104_MOESM7_ESM.zip › Supplement Figure 7 incidence joinpoint/Finland female 45-69.jpg]

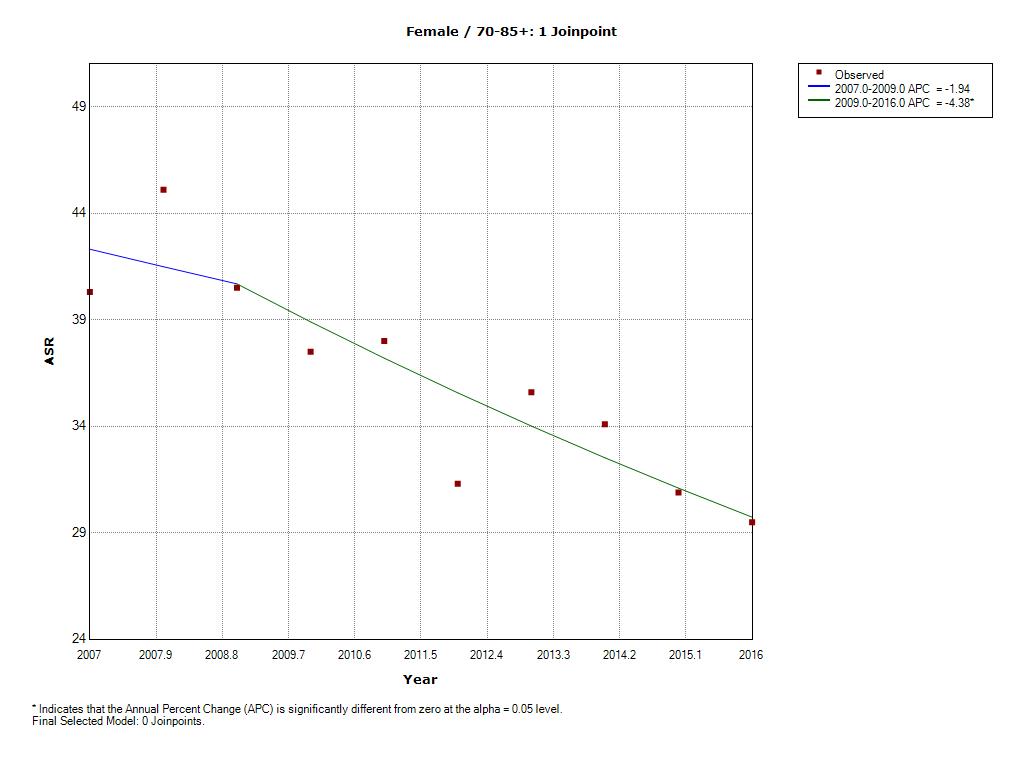

Supplement: Supplementary file 7 — Supplement Figure 7: incidence joinpoint. [file 12889_2024_19104_MOESM7_ESM.zip › Supplement Figure 7 incidence joinpoint/Finland female 70-85+.jpg]

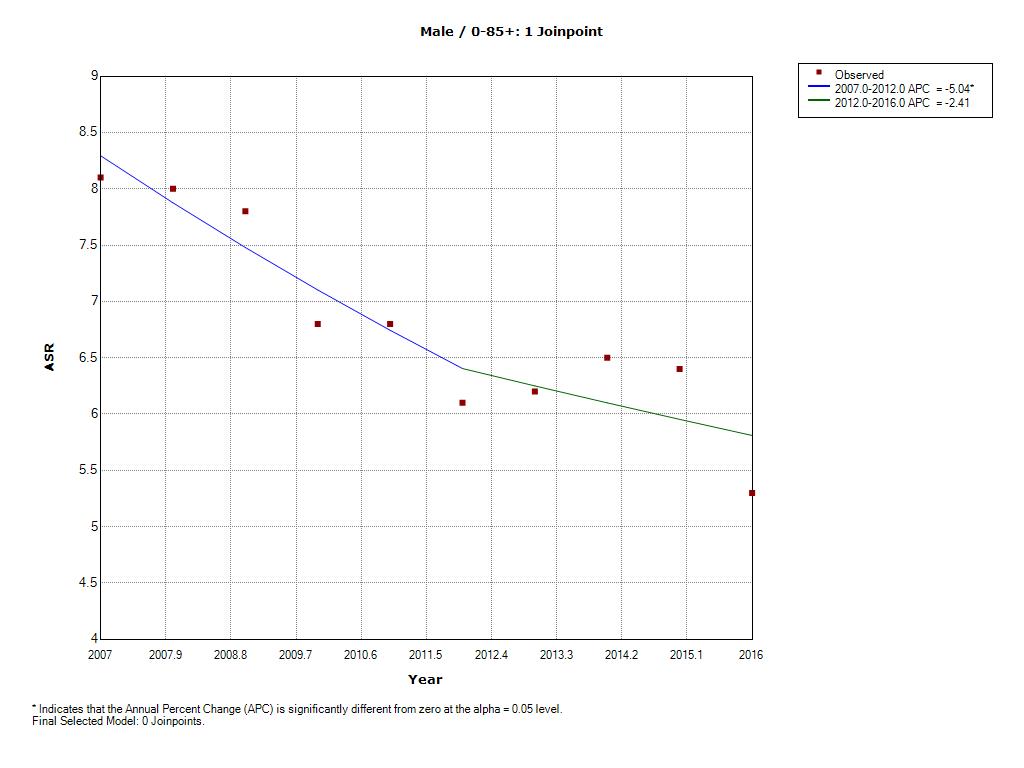

Supplement: Supplementary file 7 — Supplement Figure 7: incidence joinpoint. [file 12889_2024_19104_MOESM7_ESM.zip › Supplement Figure 7 incidence joinpoint/Finland male 0-85+.jpg]

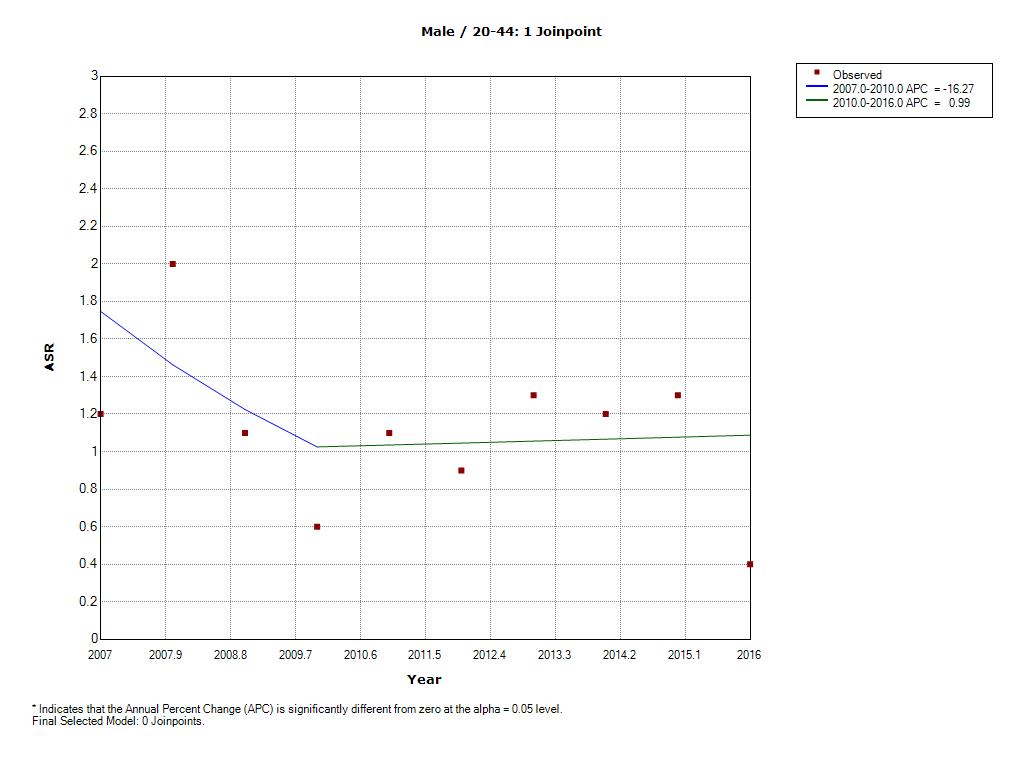

Supplement: Supplementary file 7 — Supplement Figure 7: incidence joinpoint. [file 12889_2024_19104_MOESM7_ESM.zip › Supplement Figure 7 incidence joinpoint/Finland male 20-44.jpg]

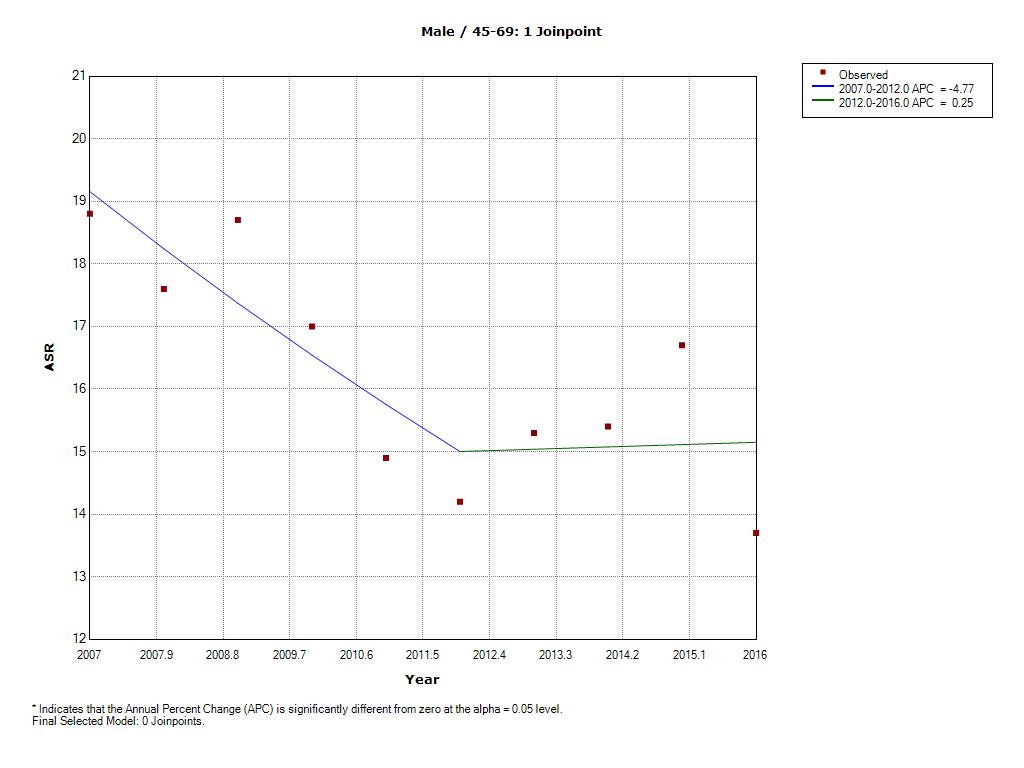

Supplement: Supplementary file 7 — Supplement Figure 7: incidence joinpoint. [file 12889_2024_19104_MOESM7_ESM.zip › Supplement Figure 7 incidence joinpoint/Finland male 45-69.jpg]

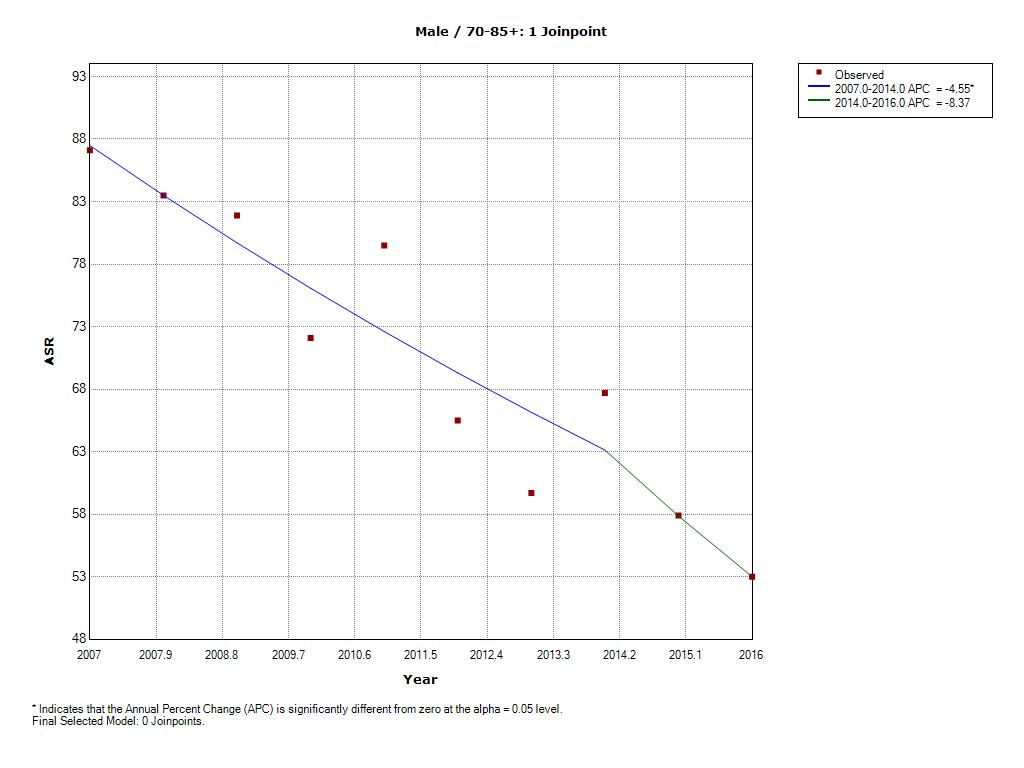

Supplement: Supplementary file 7 — Supplement Figure 7: incidence joinpoint. [file 12889_2024_19104_MOESM7_ESM.zip › Supplement Figure 7 incidence joinpoint/Finland male 70-85+.jpg]

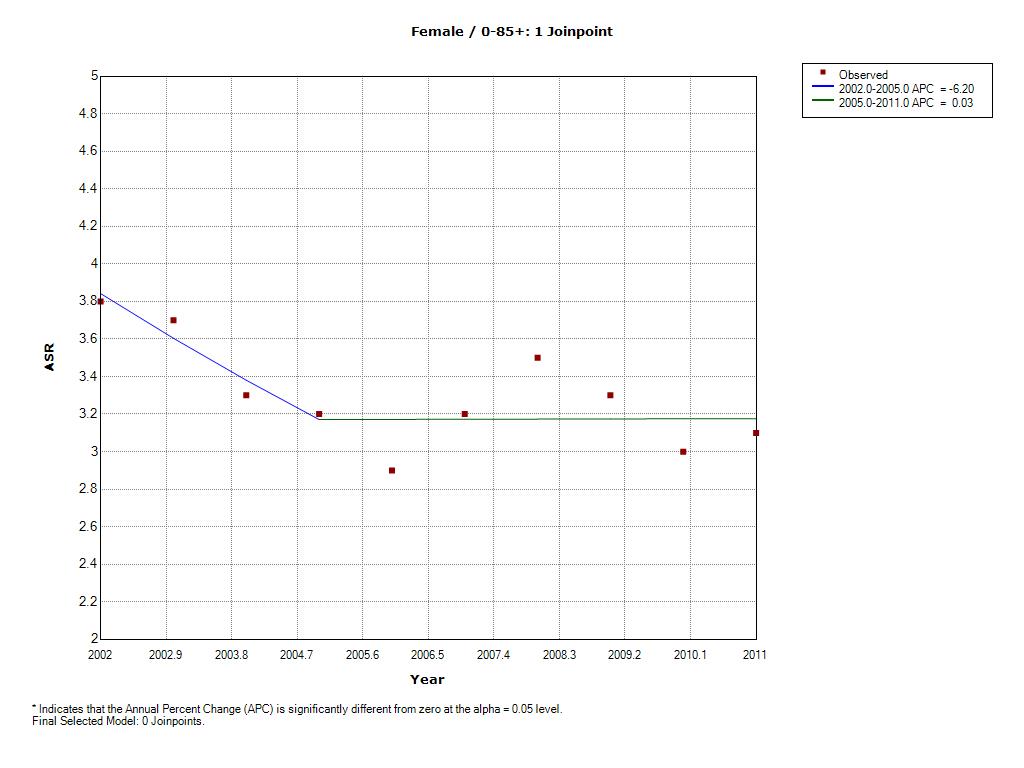

Supplement: Supplementary file 7 — Supplement Figure 7: incidence joinpoint. [file 12889_2024_19104_MOESM7_ESM.zip › Supplement Figure 7 incidence joinpoint/France female 0-85+.jpg]

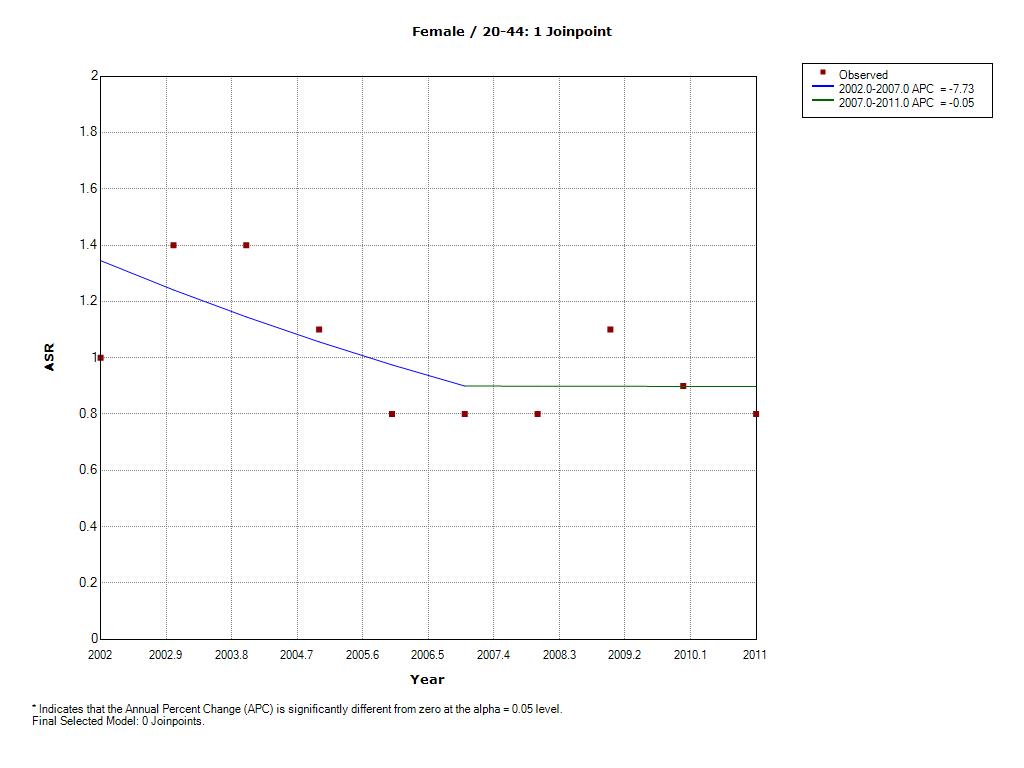

Supplement: Supplementary file 7 — Supplement Figure 7: incidence joinpoint. [file 12889_2024_19104_MOESM7_ESM.zip › Supplement Figure 7 incidence joinpoint/France female 20-44.jpg]

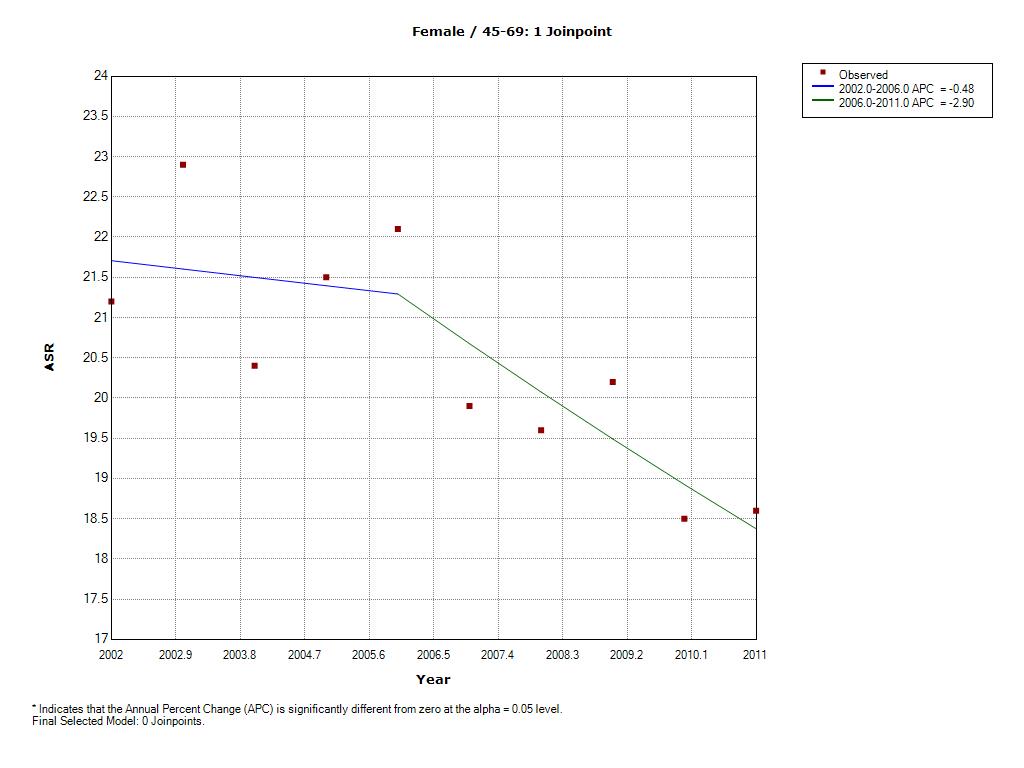

Supplement: Supplementary file 7 — Supplement Figure 7: incidence joinpoint. [file 12889_2024_19104_MOESM7_ESM.zip › Supplement Figure 7 incidence joinpoint/France female 45-69.jpg]

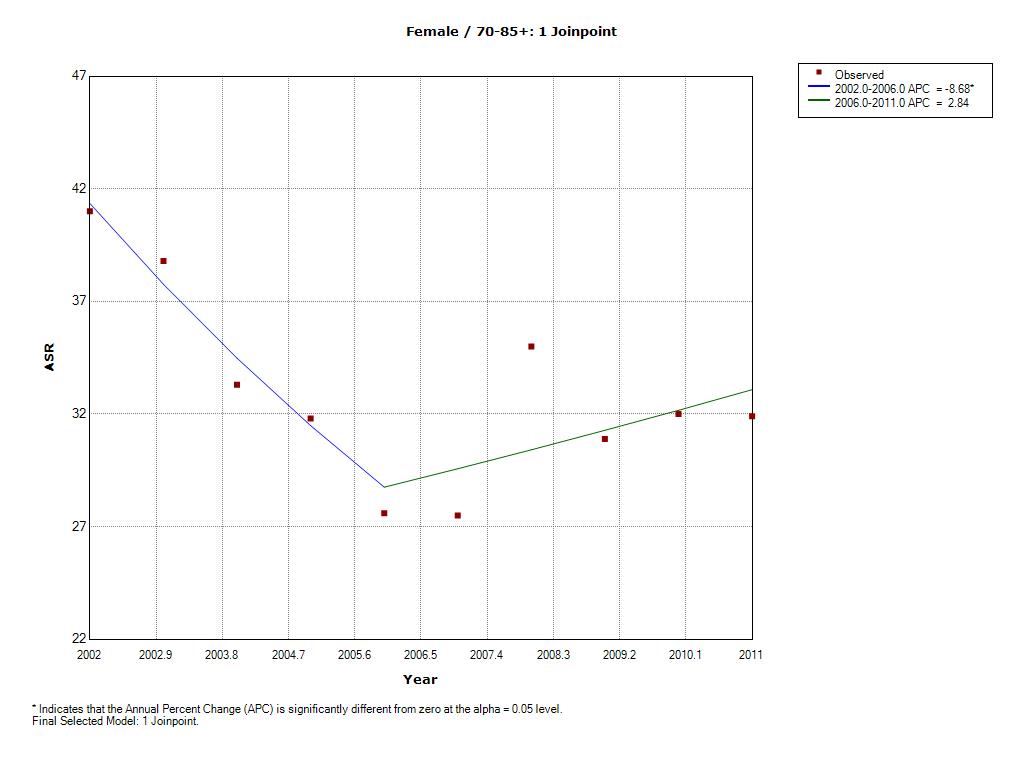

Supplement: Supplementary file 7 — Supplement Figure 7: incidence joinpoint. [file 12889_2024_19104_MOESM7_ESM.zip › Supplement Figure 7 incidence joinpoint/France female 70-85+.jpg]

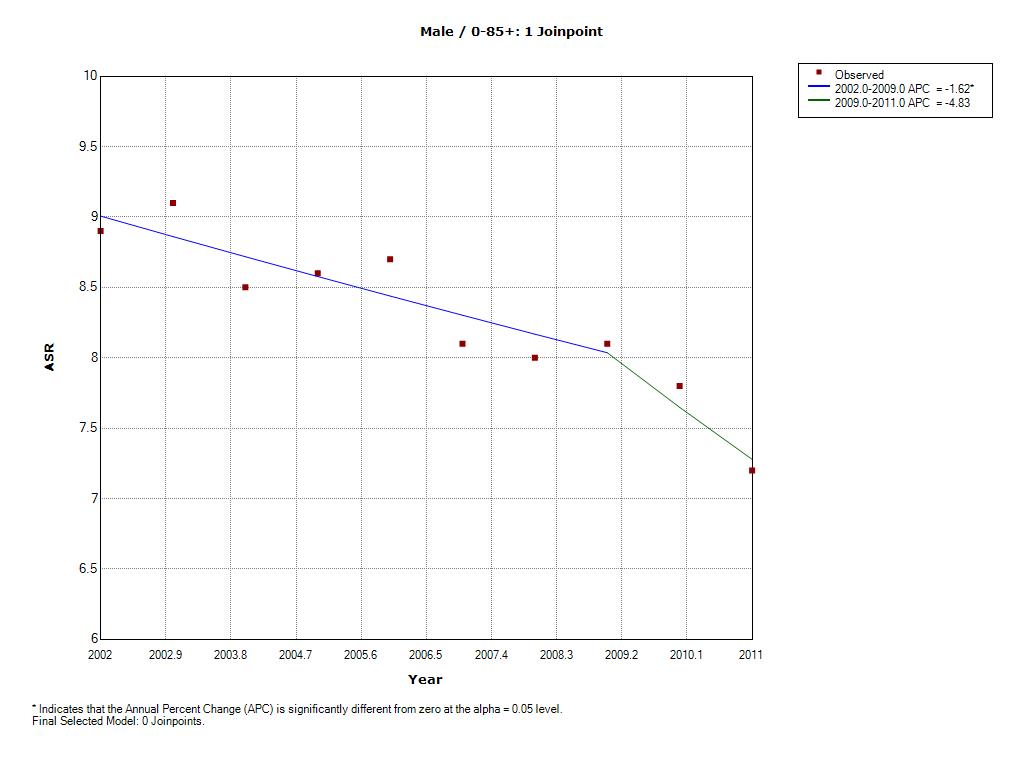

Supplement: Supplementary file 7 — Supplement Figure 7: incidence joinpoint. [file 12889_2024_19104_MOESM7_ESM.zip › Supplement Figure 7 incidence joinpoint/France male 0-85+.jpg]

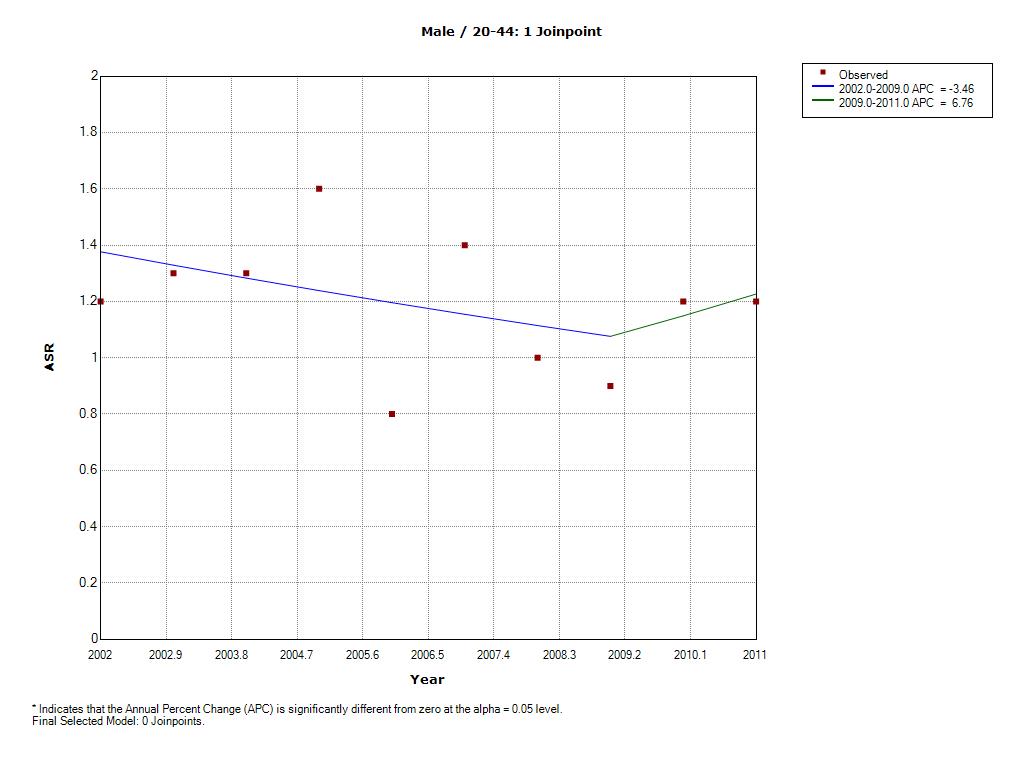

Supplement: Supplementary file 7 — Supplement Figure 7: incidence joinpoint. [file 12889_2024_19104_MOESM7_ESM.zip › Supplement Figure 7 incidence joinpoint/France male 20-44.jpg]

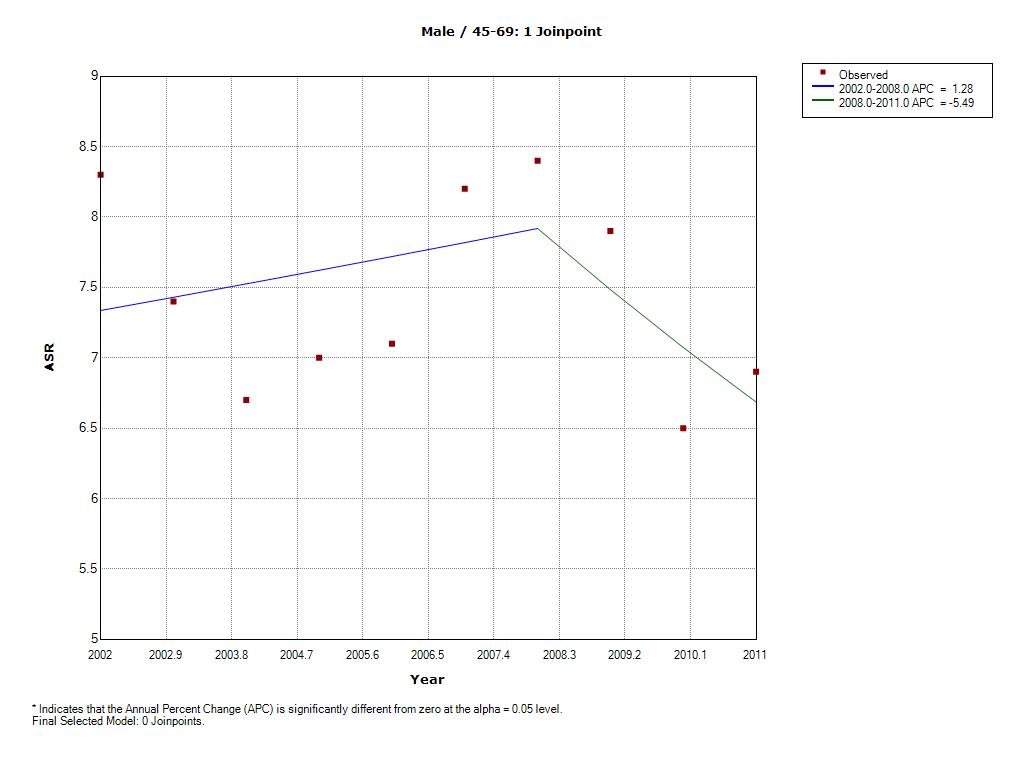

Supplement: Supplementary file 7 — Supplement Figure 7: incidence joinpoint. [file 12889_2024_19104_MOESM7_ESM.zip › Supplement Figure 7 incidence joinpoint/France male 45-69.jpg]

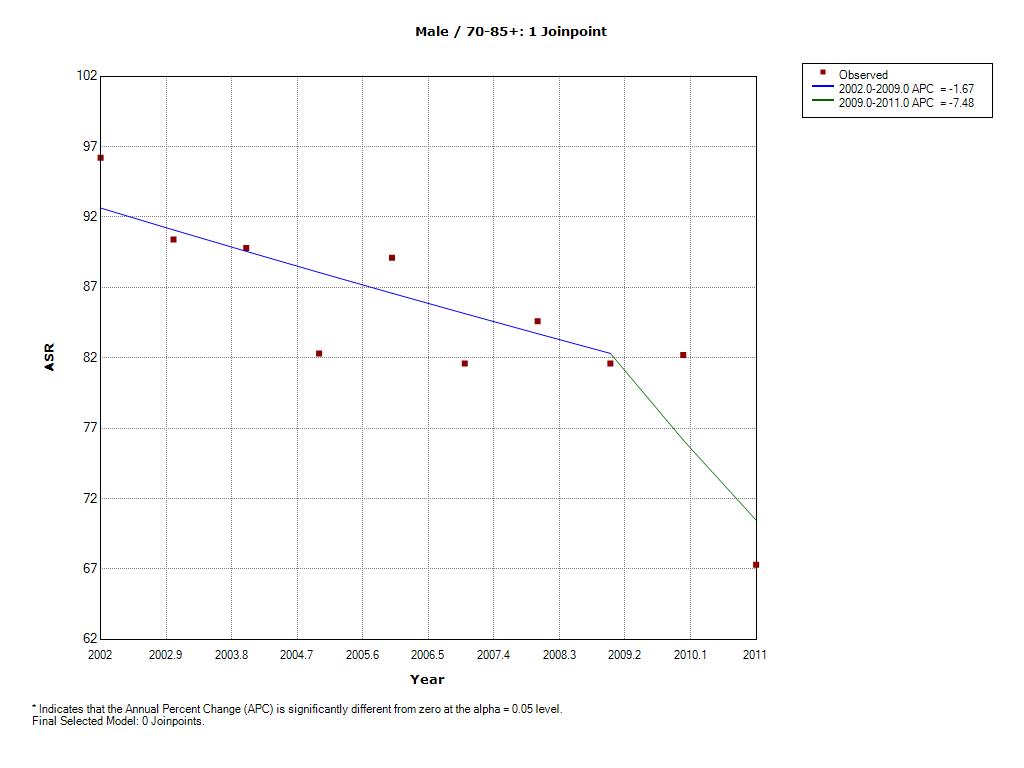

Supplement: Supplementary file 7 — Supplement Figure 7: incidence joinpoint. [file 12889_2024_19104_MOESM7_ESM.zip › Supplement Figure 7 incidence joinpoint/France male 70-85+.jpg]

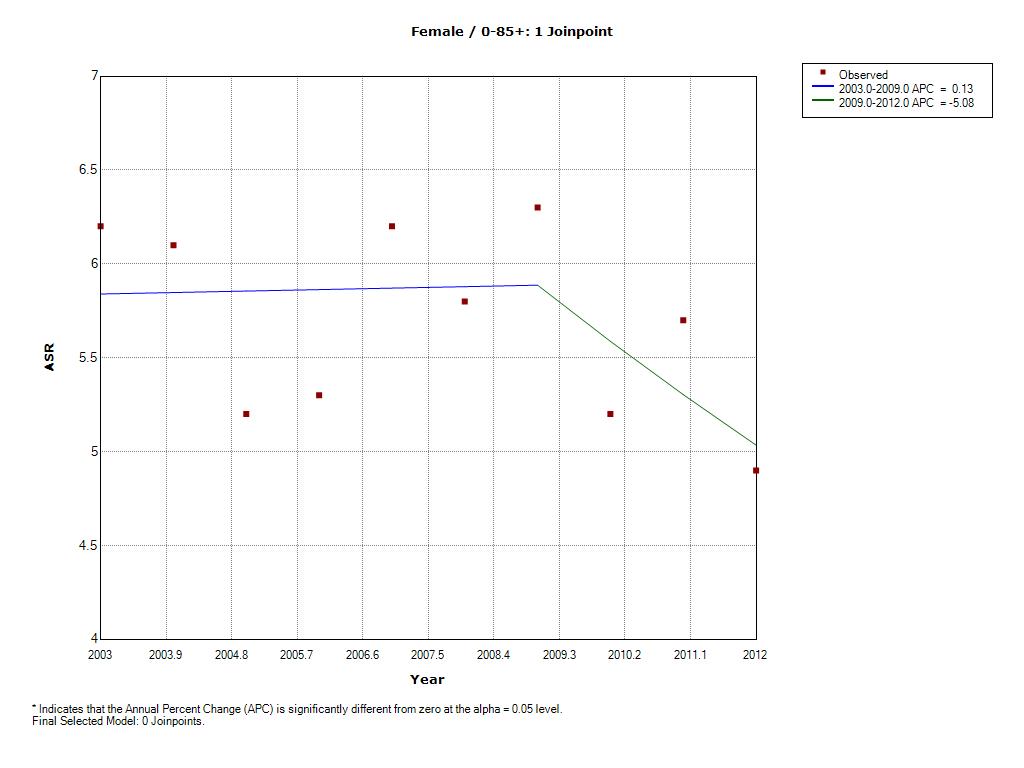

Supplement: Supplementary file 7 — Supplement Figure 7: incidence joinpoint. [file 12889_2024_19104_MOESM7_ESM.zip › Supplement Figure 7 incidence joinpoint/Germany female 0-85+.jpg]

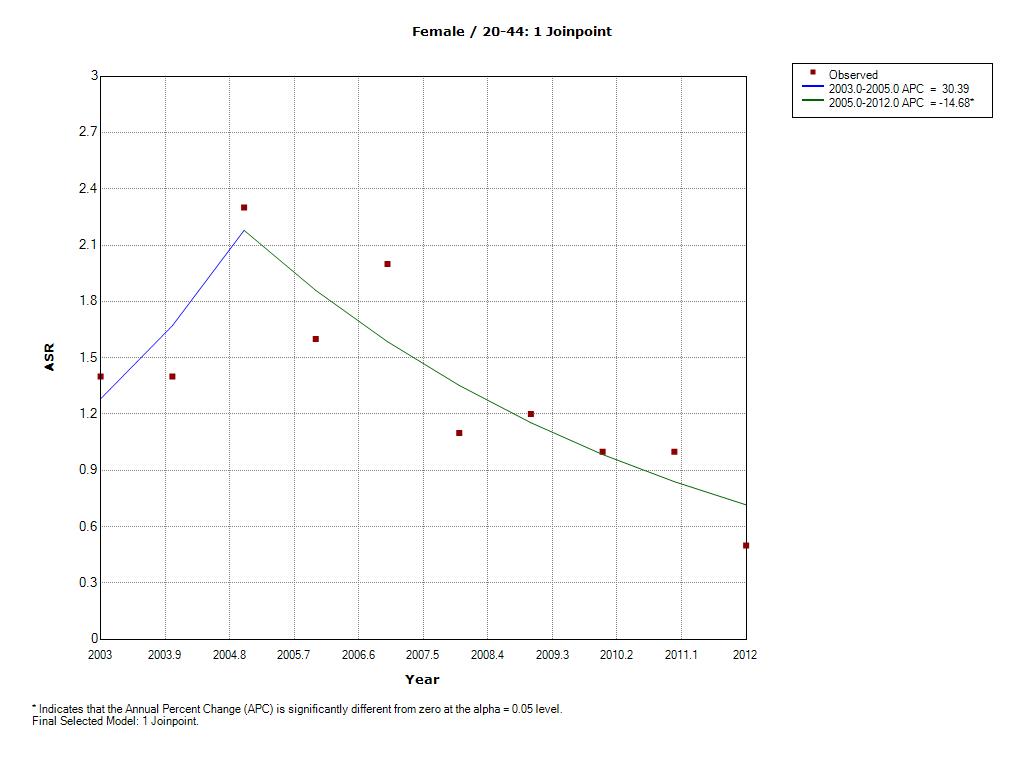

Supplement: Supplementary file 7 — Supplement Figure 7: incidence joinpoint. [file 12889_2024_19104_MOESM7_ESM.zip › Supplement Figure 7 incidence joinpoint/Germany female 20-44.jpg]

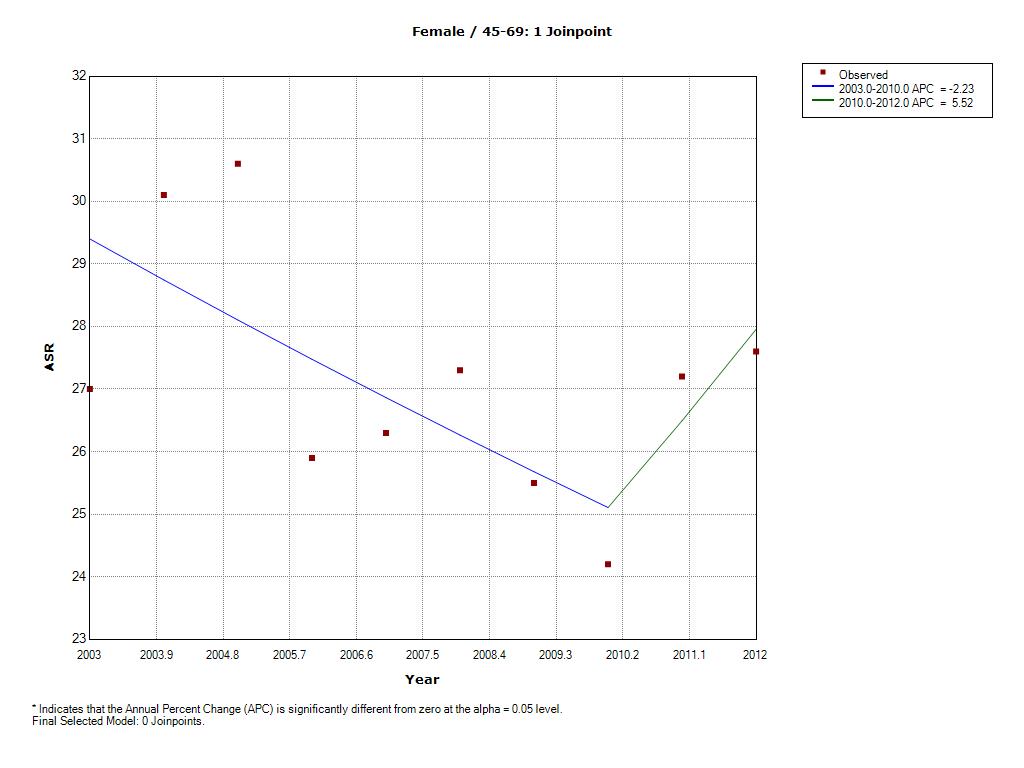

Supplement: Supplementary file 7 — Supplement Figure 7: incidence joinpoint. [file 12889_2024_19104_MOESM7_ESM.zip › Supplement Figure 7 incidence joinpoint/Germany female 45-69.jpg]

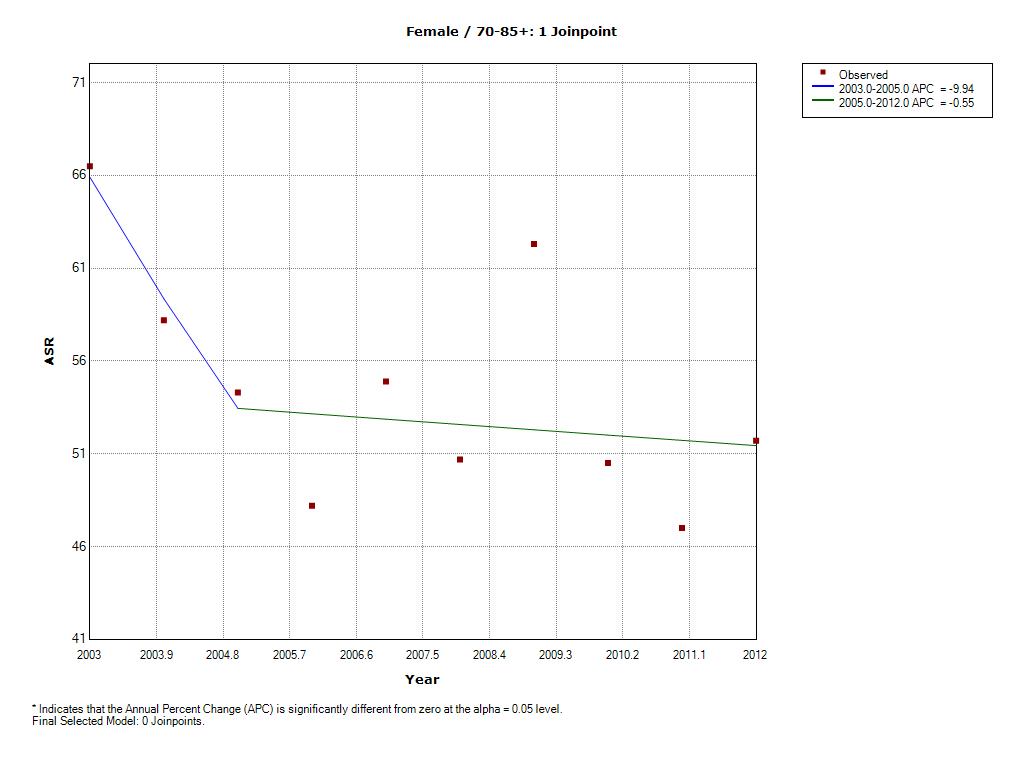

Supplement: Supplementary file 7 — Supplement Figure 7: incidence joinpoint. [file 12889_2024_19104_MOESM7_ESM.zip › Supplement Figure 7 incidence joinpoint/Germany female 70-85+.jpg]

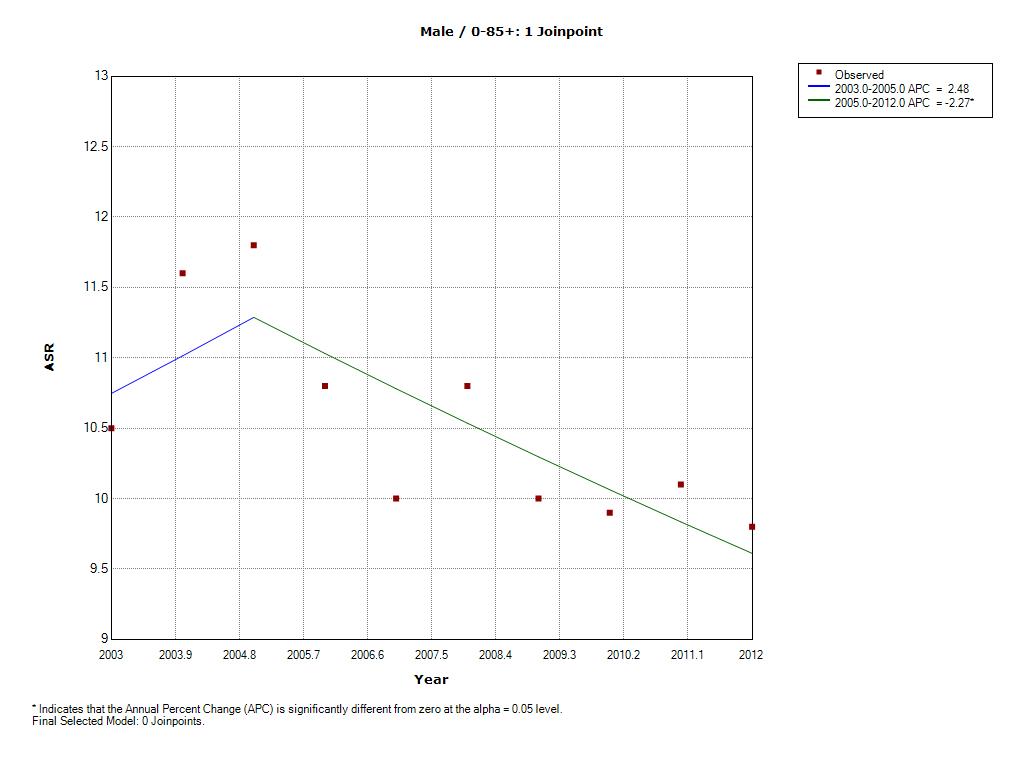

Supplement: Supplementary file 7 — Supplement Figure 7: incidence joinpoint. [file 12889_2024_19104_MOESM7_ESM.zip › Supplement Figure 7 incidence joinpoint/Germany male 0-85+.jpg]

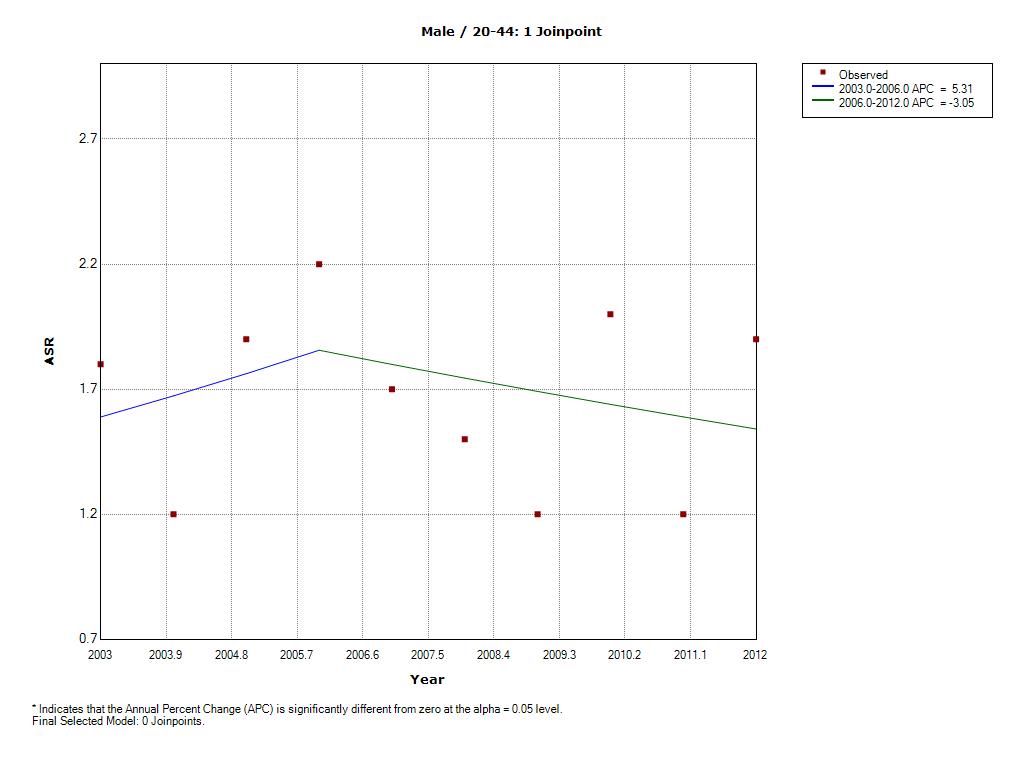

Supplement: Supplementary file 7 — Supplement Figure 7: incidence joinpoint. [file 12889_2024_19104_MOESM7_ESM.zip › Supplement Figure 7 incidence joinpoint/Germany male 20-44.jpg]

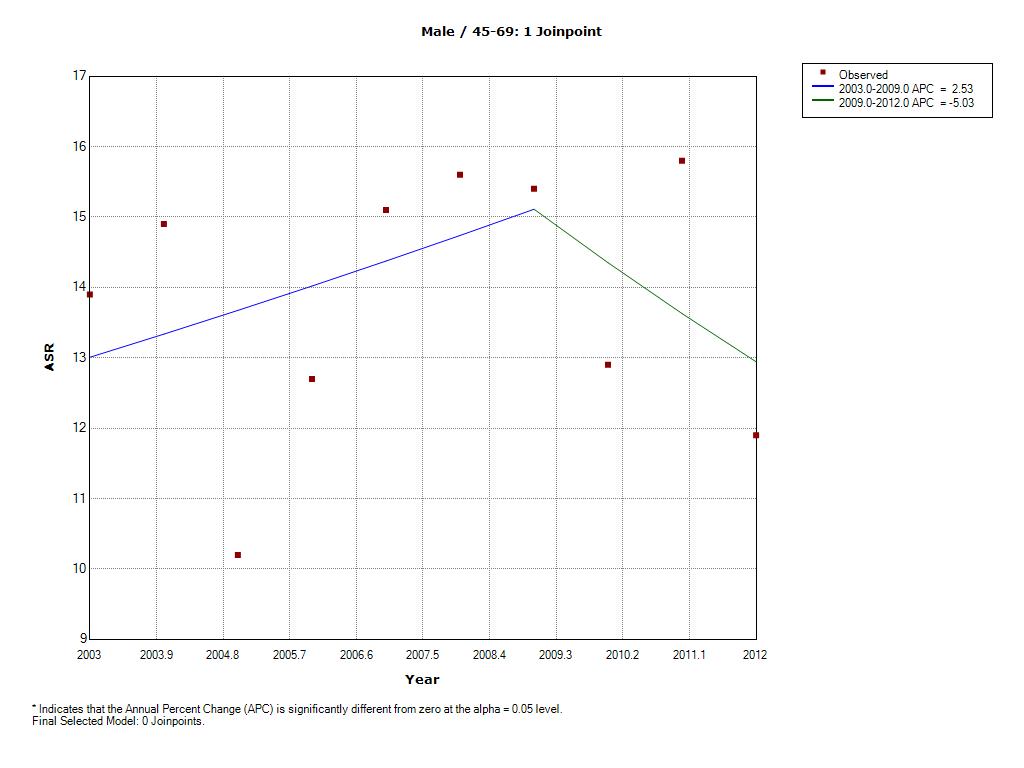

Supplement: Supplementary file 7 — Supplement Figure 7: incidence joinpoint. [file 12889_2024_19104_MOESM7_ESM.zip › Supplement Figure 7 incidence joinpoint/Germany male 45-69.jpg]

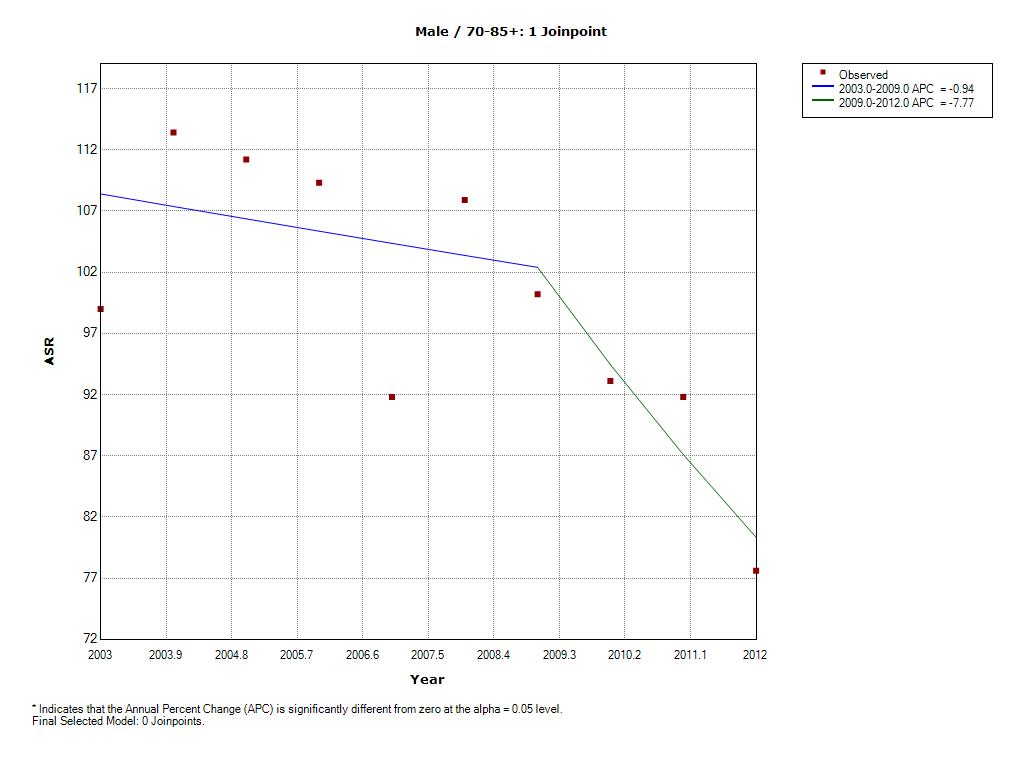

Supplement: Supplementary file 7 — Supplement Figure 7: incidence joinpoint. [file 12889_2024_19104_MOESM7_ESM.zip › Supplement Figure 7 incidence joinpoint/Germany male 70-85+.jpg]

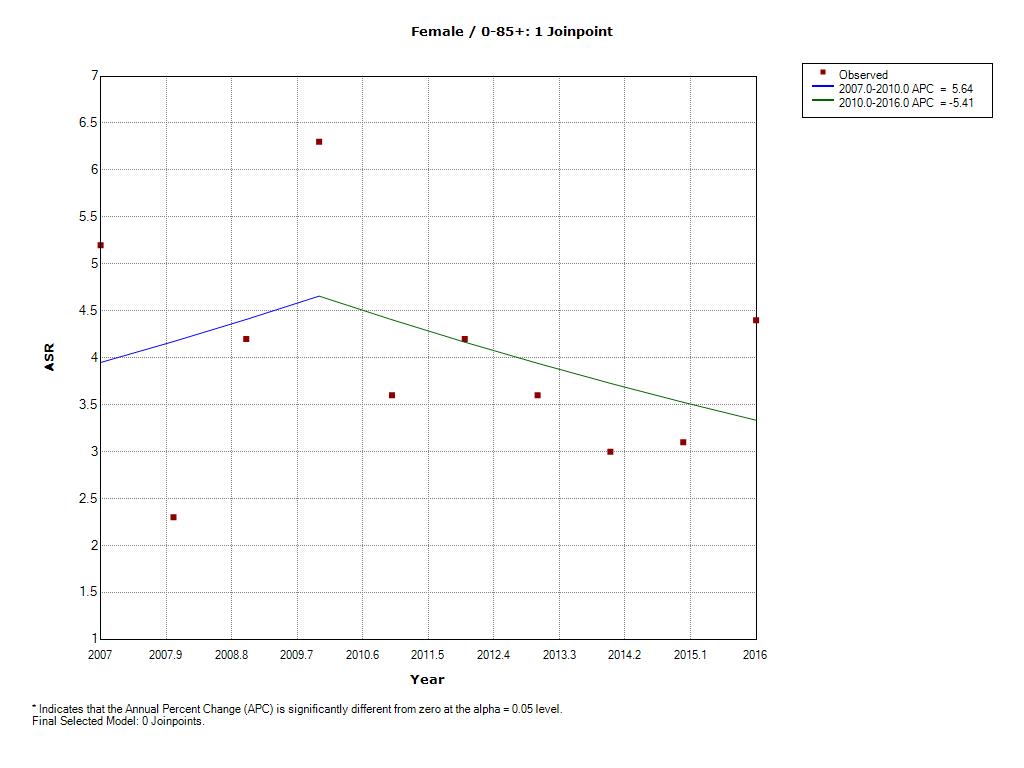

Supplement: Supplementary file 7 — Supplement Figure 7: incidence joinpoint. [file 12889_2024_19104_MOESM7_ESM.zip › Supplement Figure 7 incidence joinpoint/Iceland female 0-85+.jpg]

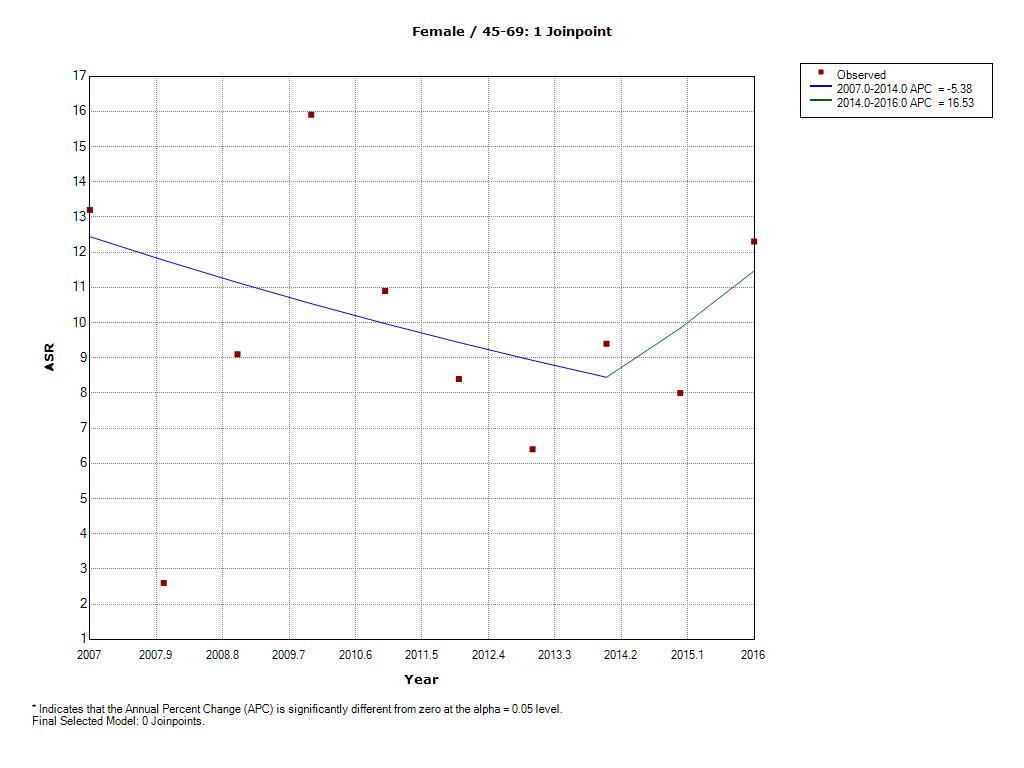

Supplement: Supplementary file 7 — Supplement Figure 7: incidence joinpoint. [file 12889_2024_19104_MOESM7_ESM.zip › Supplement Figure 7 incidence joinpoint/Iceland female 20-44.jpg]

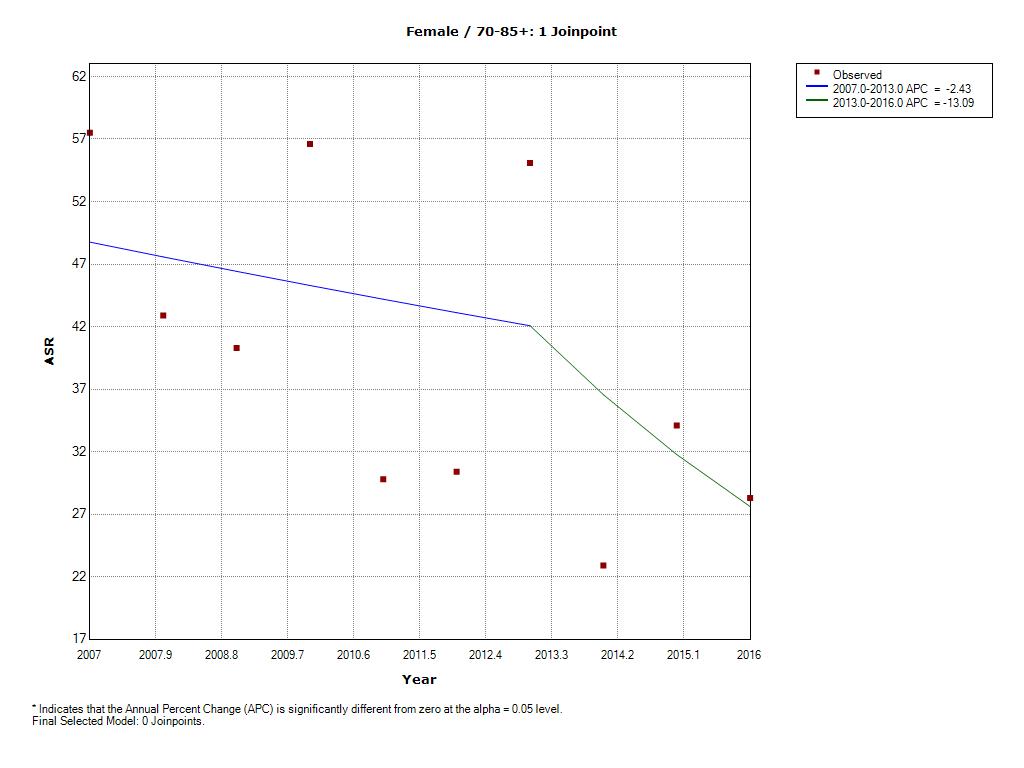

Supplement: Supplementary file 7 — Supplement Figure 7: incidence joinpoint. [file 12889_2024_19104_MOESM7_ESM.zip › Supplement Figure 7 incidence joinpoint/Iceland female 70-85+.jpg]

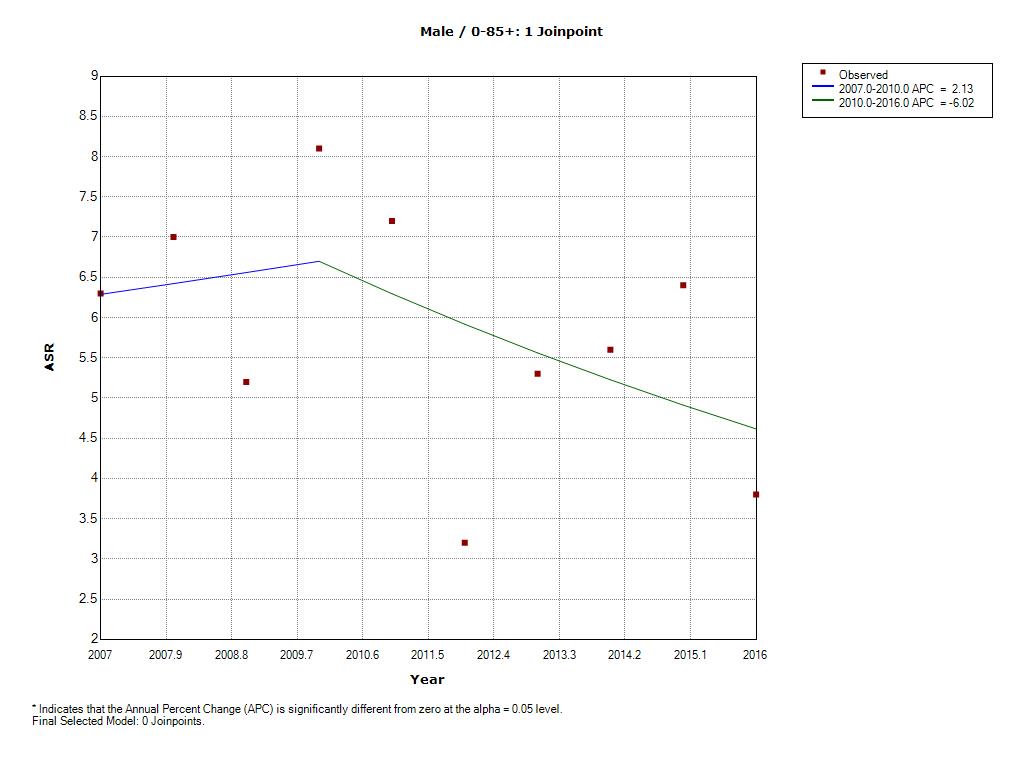

Supplement: Supplementary file 7 — Supplement Figure 7: incidence joinpoint. [file 12889_2024_19104_MOESM7_ESM.zip › Supplement Figure 7 incidence joinpoint/Iceland male 0-85+.jpg]

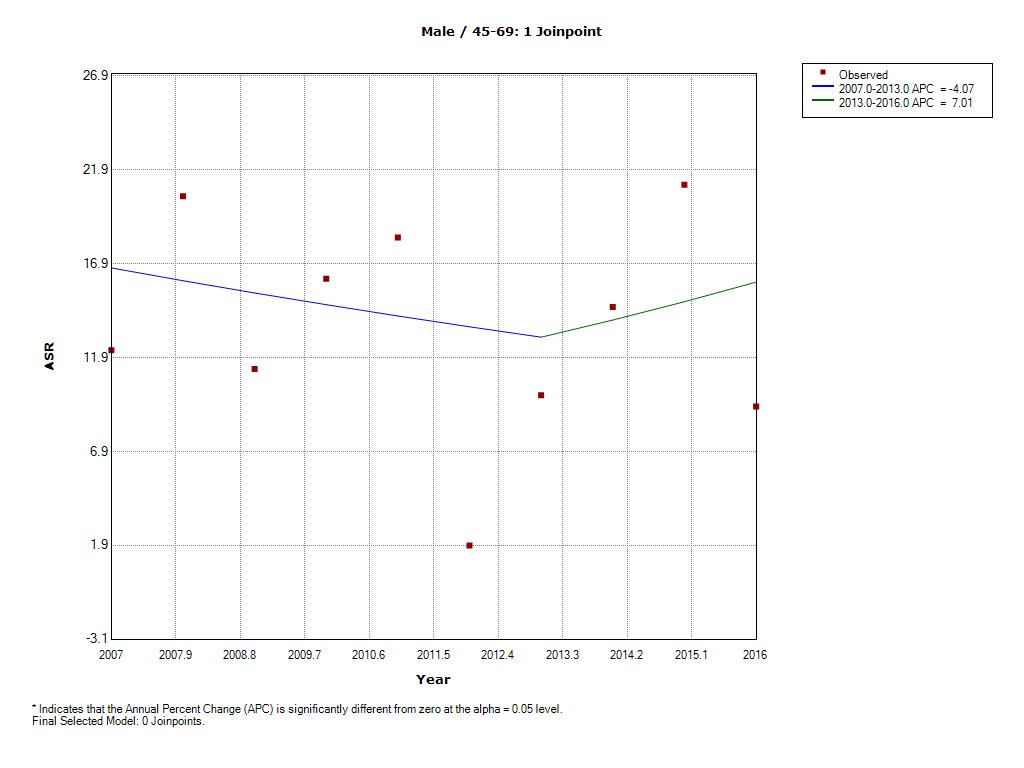

Supplement: Supplementary file 7 — Supplement Figure 7: incidence joinpoint. [file 12889_2024_19104_MOESM7_ESM.zip › Supplement Figure 7 incidence joinpoint/Iceland male 20-44.jpg]

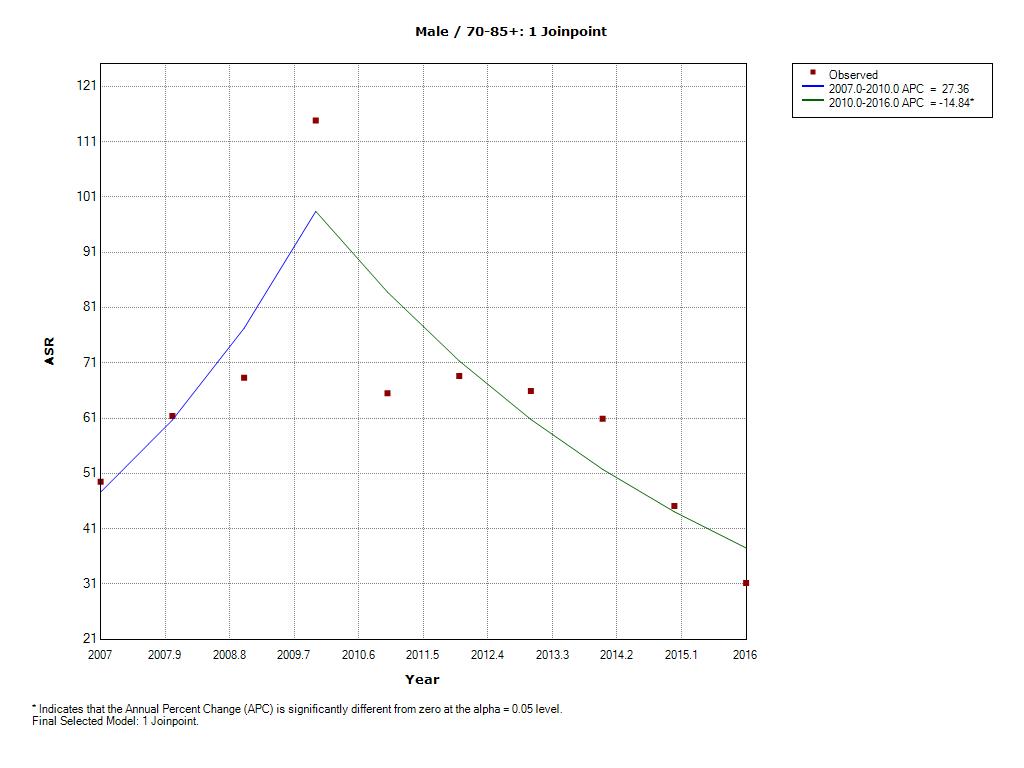

Supplement: Supplementary file 7 — Supplement Figure 7: incidence joinpoint. [file 12889_2024_19104_MOESM7_ESM.zip › Supplement Figure 7 incidence joinpoint/Iceland male 70-85+.jpg]

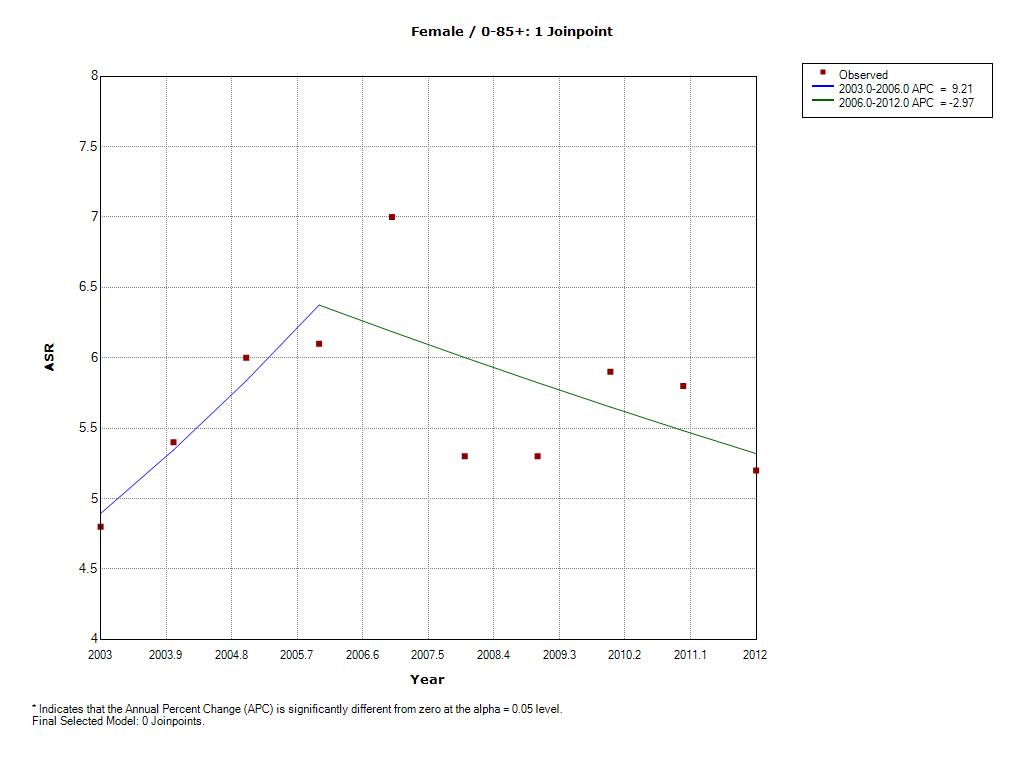

Supplement: Supplementary file 7 — Supplement Figure 7: incidence joinpoint. [file 12889_2024_19104_MOESM7_ESM.zip › Supplement Figure 7 incidence joinpoint/India female 0-85+.jpg]

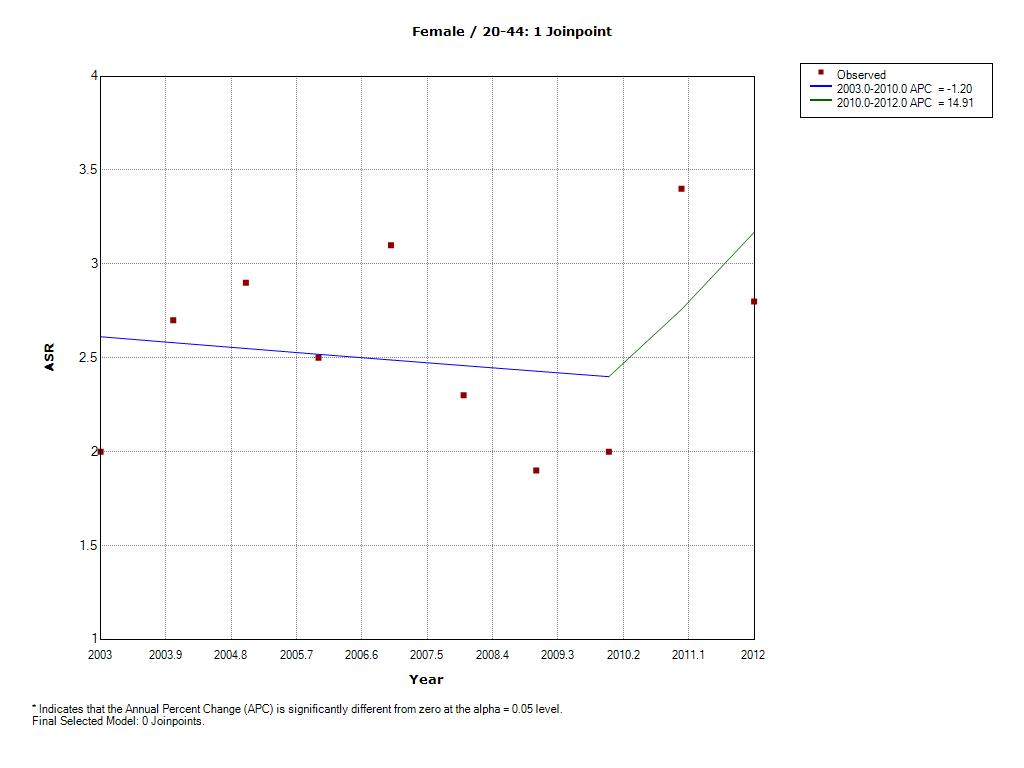

Supplement: Supplementary file 7 — Supplement Figure 7: incidence joinpoint. [file 12889_2024_19104_MOESM7_ESM.zip › Supplement Figure 7 incidence joinpoint/India female 20-44.jpg]

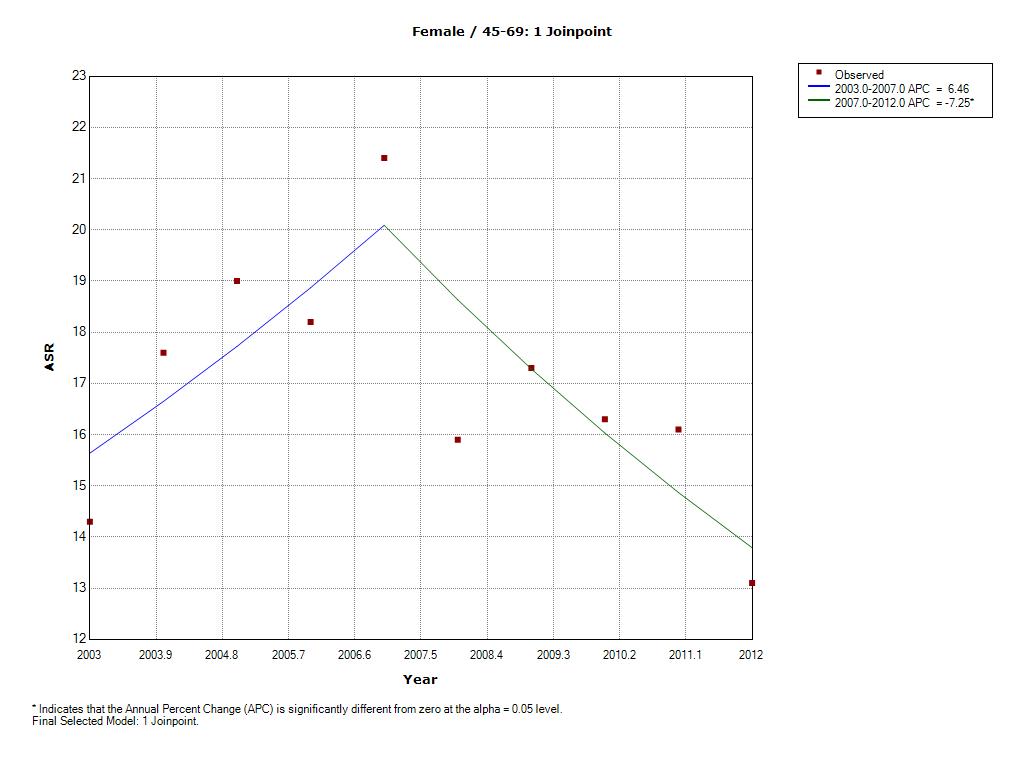

Supplement: Supplementary file 7 — Supplement Figure 7: incidence joinpoint. [file 12889_2024_19104_MOESM7_ESM.zip › Supplement Figure 7 incidence joinpoint/India female 45-69.jpg]

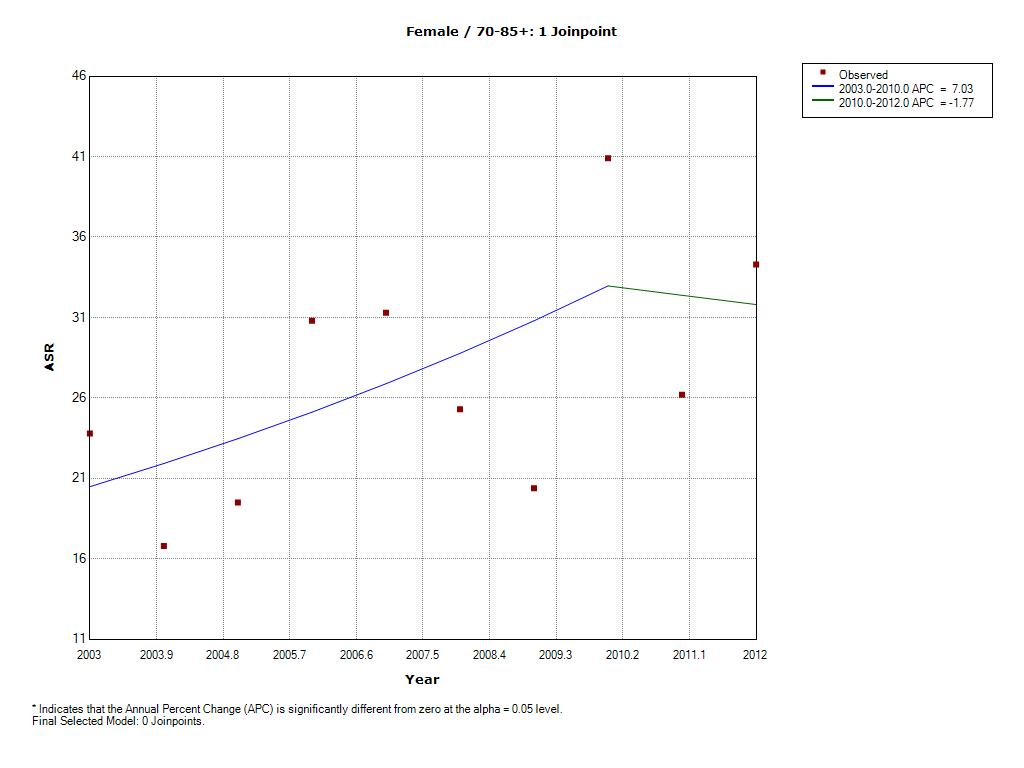

Supplement: Supplementary file 7 — Supplement Figure 7: incidence joinpoint. [file 12889_2024_19104_MOESM7_ESM.zip › Supplement Figure 7 incidence joinpoint/India female 70-85+.jpg]

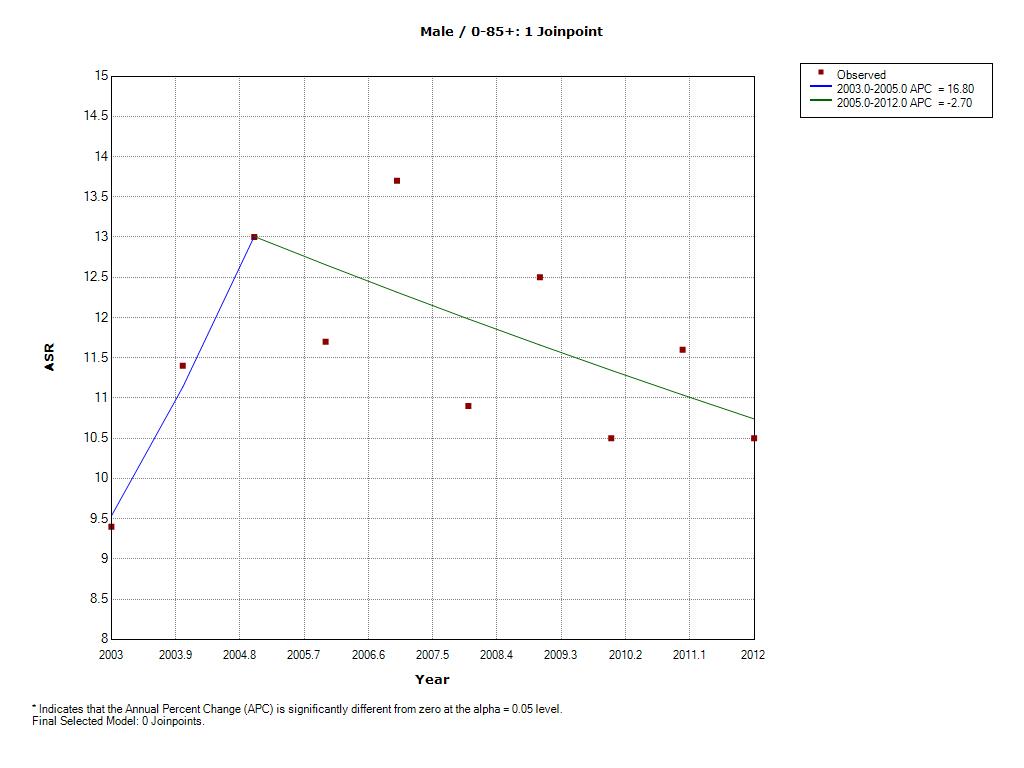

Supplement: Supplementary file 7 — Supplement Figure 7: incidence joinpoint. [file 12889_2024_19104_MOESM7_ESM.zip › Supplement Figure 7 incidence joinpoint/India male 0-85+.jpg]

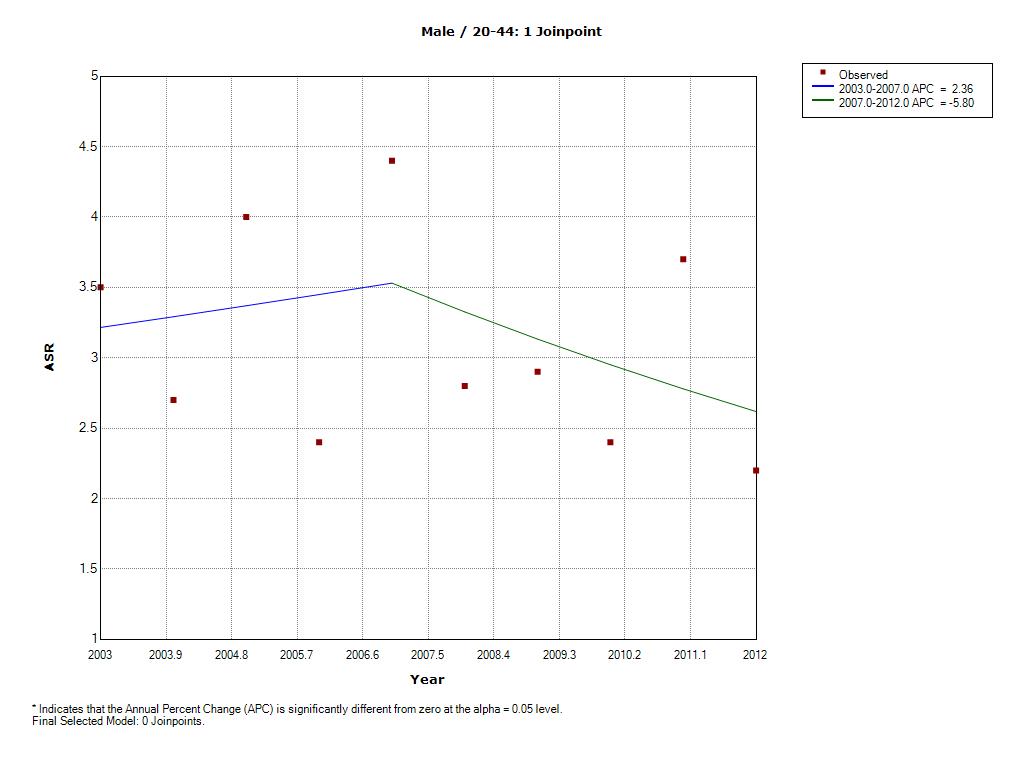

Supplement: Supplementary file 7 — Supplement Figure 7: incidence joinpoint. [file 12889_2024_19104_MOESM7_ESM.zip › Supplement Figure 7 incidence joinpoint/India male 20-44.jpg]

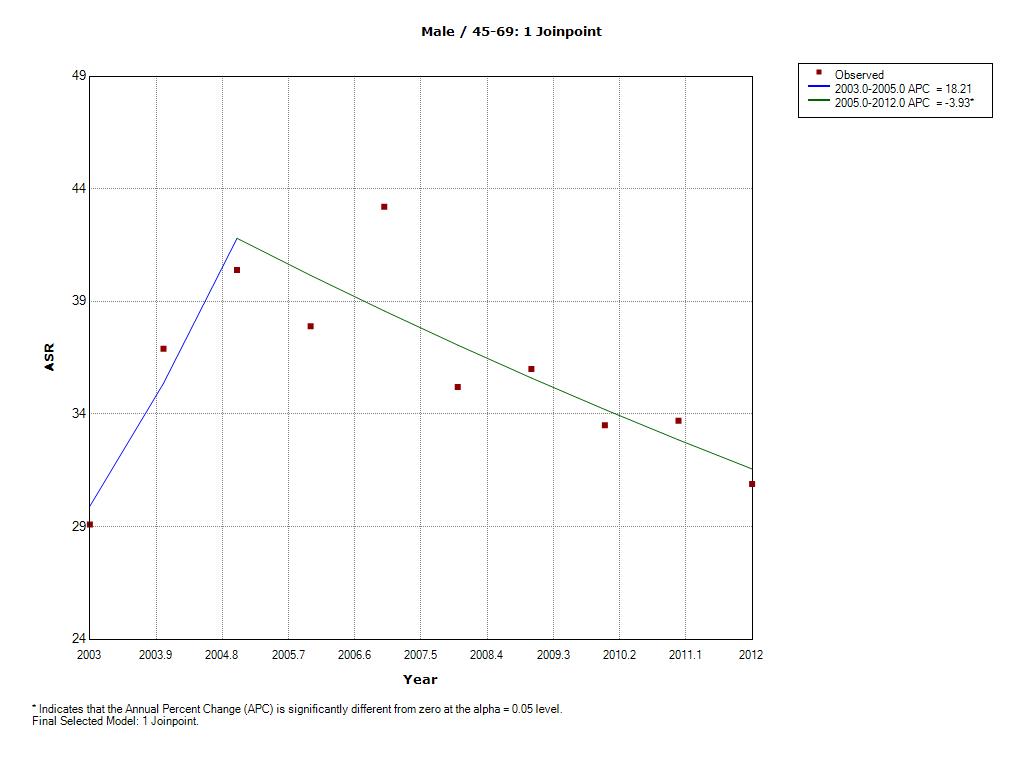

Supplement: Supplementary file 7 — Supplement Figure 7: incidence joinpoint. [file 12889_2024_19104_MOESM7_ESM.zip › Supplement Figure 7 incidence joinpoint/India male 45-69.jpg]

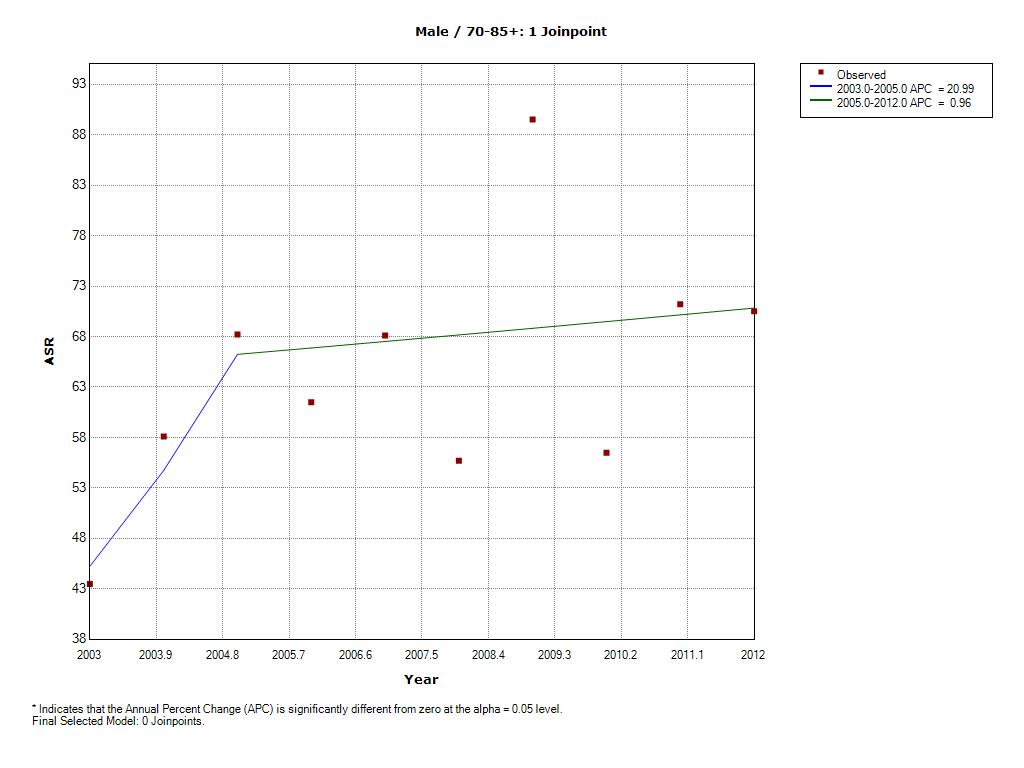

Supplement: Supplementary file 7 — Supplement Figure 7: incidence joinpoint. [file 12889_2024_19104_MOESM7_ESM.zip › Supplement Figure 7 incidence joinpoint/India male 70-85+.jpg]

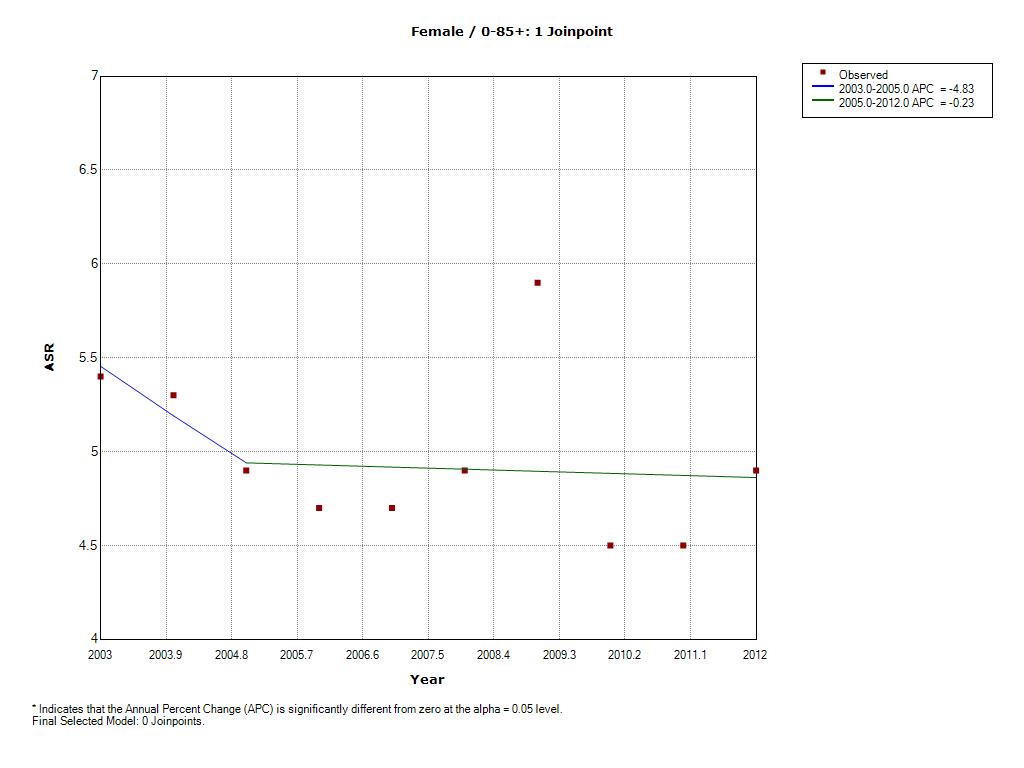

Supplement: Supplementary file 7 — Supplement Figure 7: incidence joinpoint. [file 12889_2024_19104_MOESM7_ESM.zip › Supplement Figure 7 incidence joinpoint/Ireland female 0-85+.jpg]

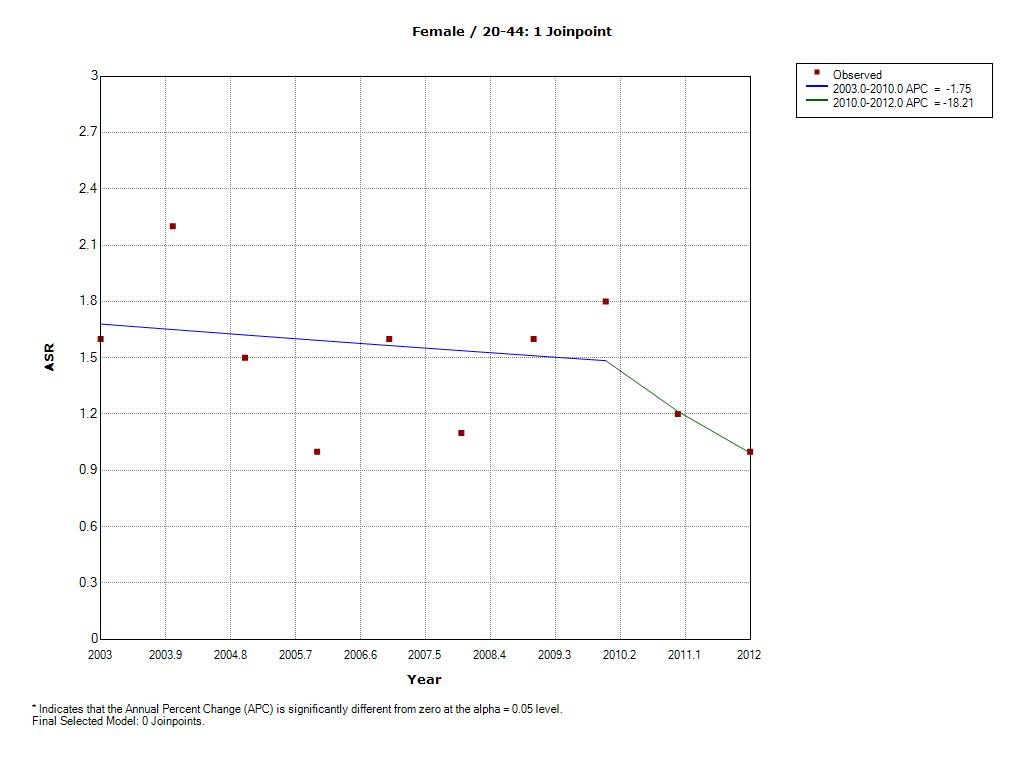

Supplement: Supplementary file 7 — Supplement Figure 7: incidence joinpoint. [file 12889_2024_19104_MOESM7_ESM.zip › Supplement Figure 7 incidence joinpoint/Ireland female 20-44.jpg]

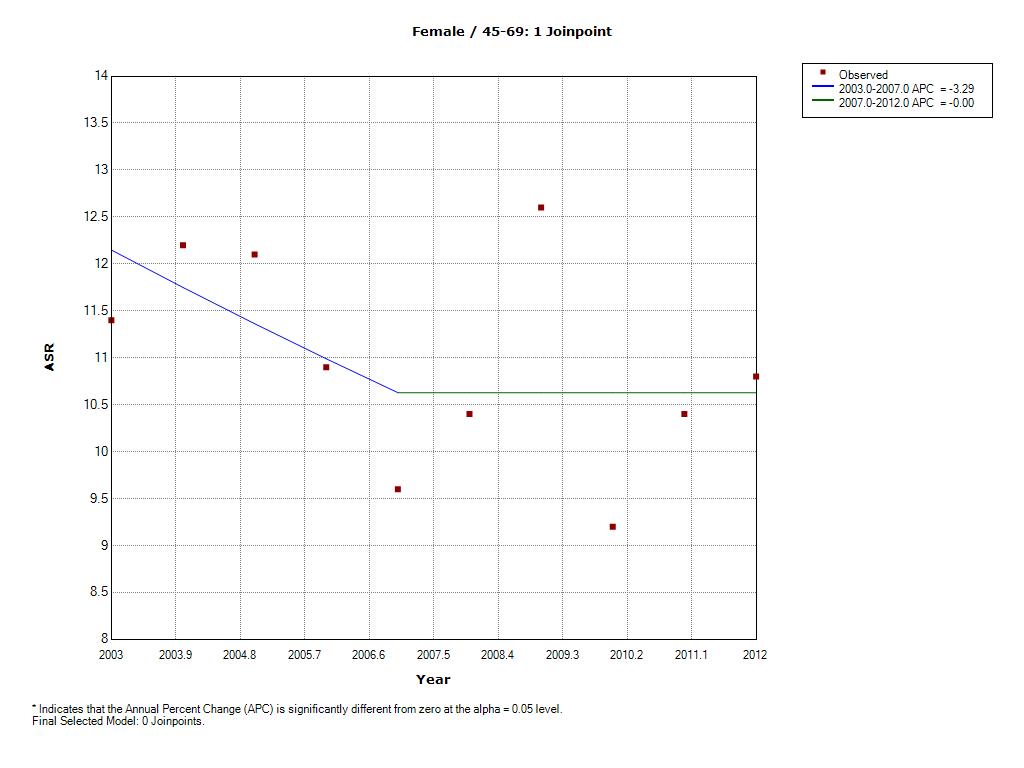

Supplement: Supplementary file 7 — Supplement Figure 7: incidence joinpoint. [file 12889_2024_19104_MOESM7_ESM.zip › Supplement Figure 7 incidence joinpoint/Ireland female 45-69.jpg]

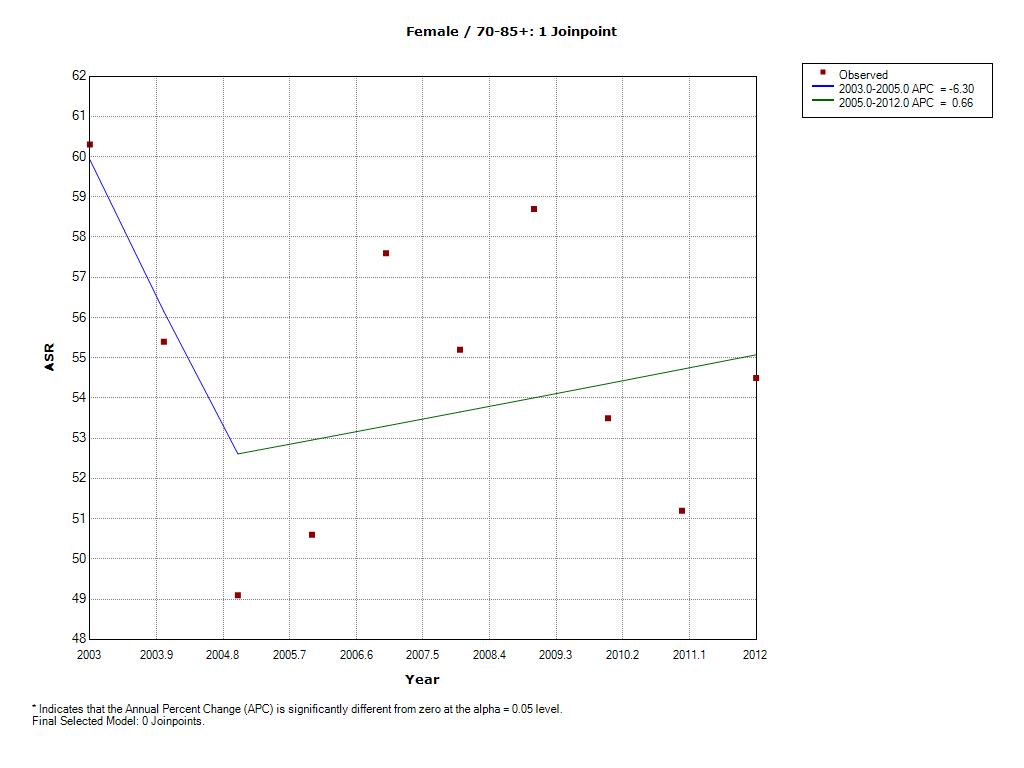

Supplement: Supplementary file 7 — Supplement Figure 7: incidence joinpoint. [file 12889_2024_19104_MOESM7_ESM.zip › Supplement Figure 7 incidence joinpoint/Ireland female 70-85+.jpg]

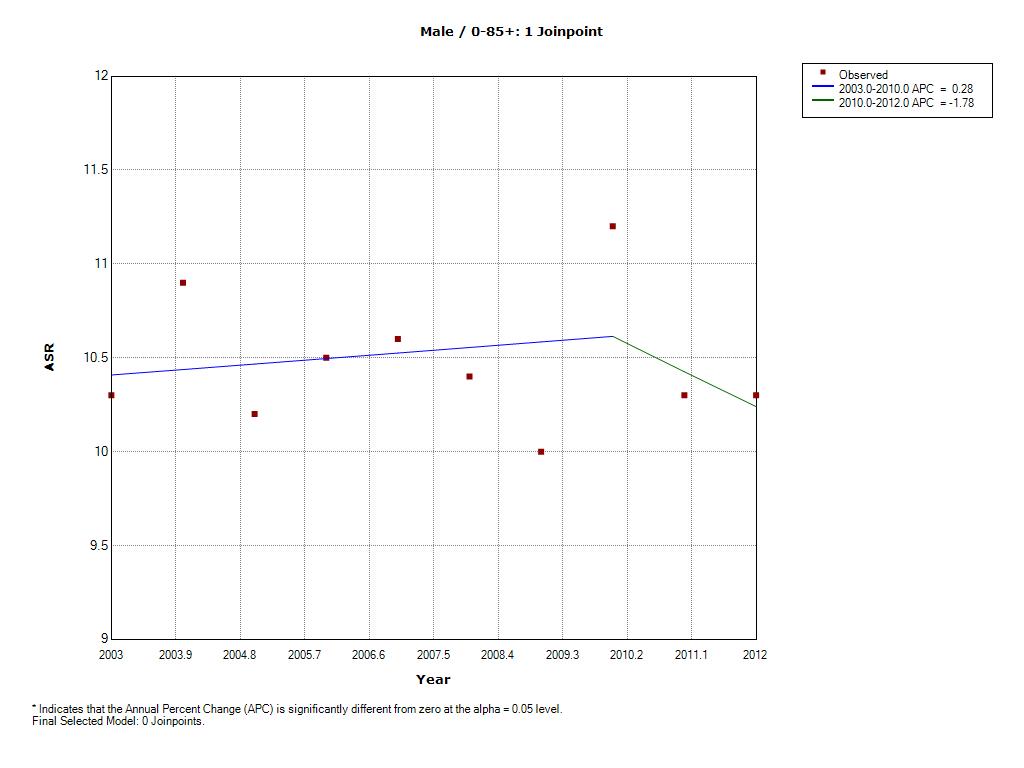

Supplement: Supplementary file 7 — Supplement Figure 7: incidence joinpoint. [file 12889_2024_19104_MOESM7_ESM.zip › Supplement Figure 7 incidence joinpoint/Ireland male 0-85+.jpg]

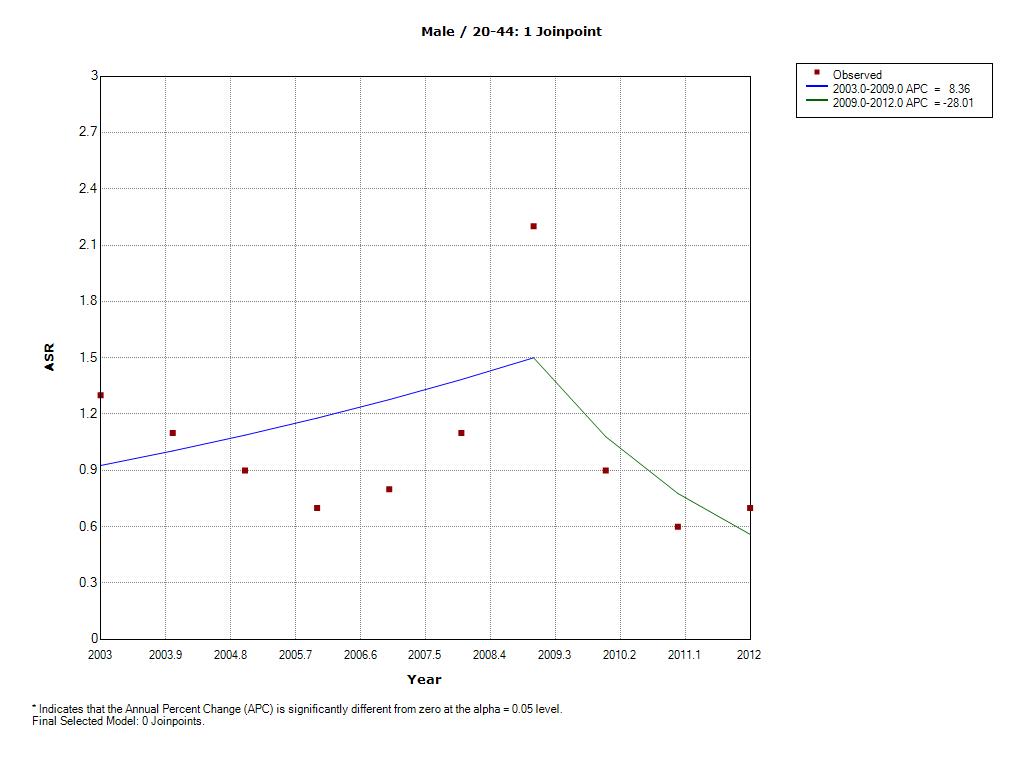

Supplement: Supplementary file 7 — Supplement Figure 7: incidence joinpoint. [file 12889_2024_19104_MOESM7_ESM.zip › Supplement Figure 7 incidence joinpoint/Ireland male 20-44.jpg]

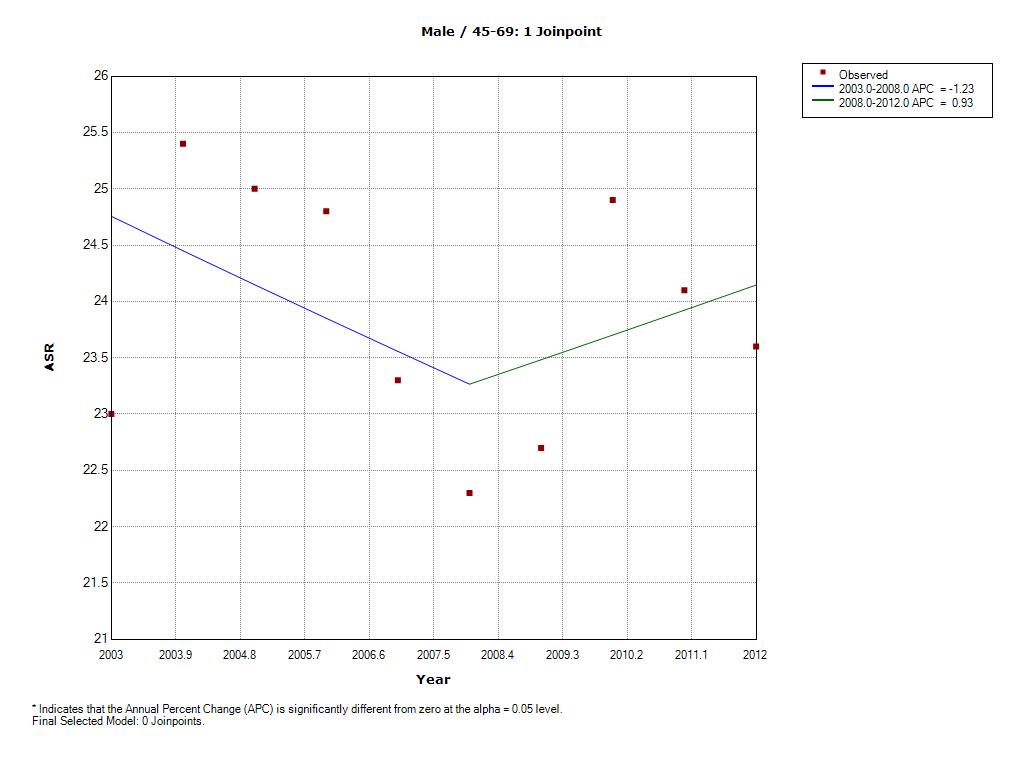

Supplement: Supplementary file 7 — Supplement Figure 7: incidence joinpoint. [file 12889_2024_19104_MOESM7_ESM.zip › Supplement Figure 7 incidence joinpoint/Ireland male 45-69.jpg]

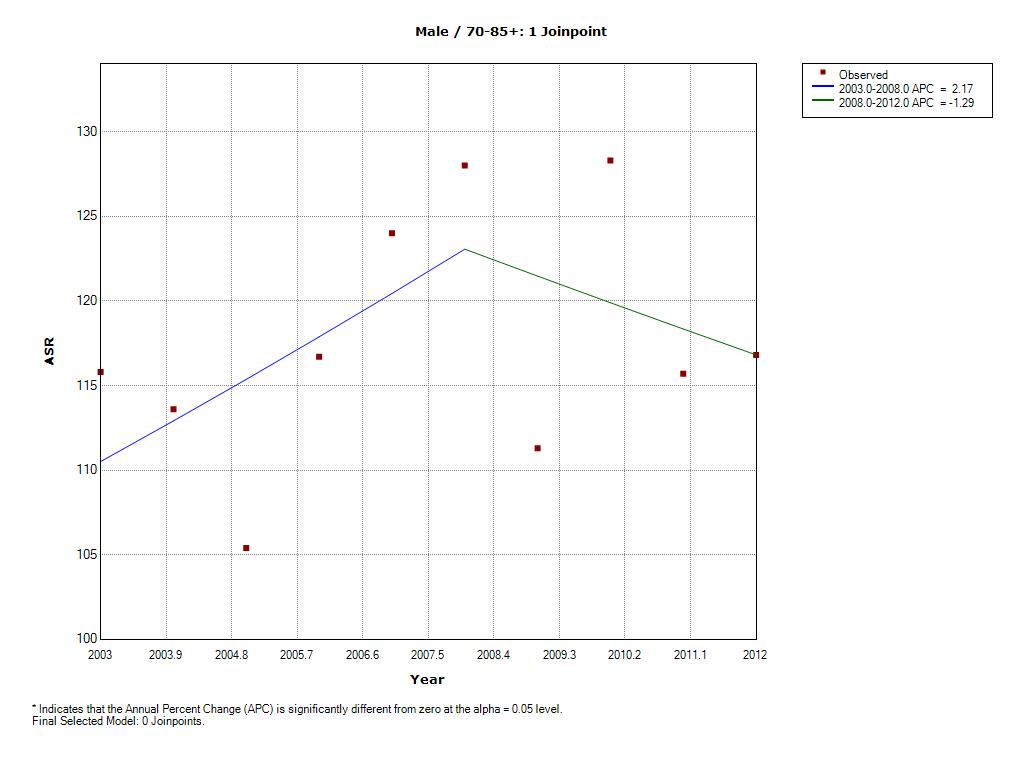

Supplement: Supplementary file 7 — Supplement Figure 7: incidence joinpoint. [file 12889_2024_19104_MOESM7_ESM.zip › Supplement Figure 7 incidence joinpoint/Ireland male 70-85+.jpg]

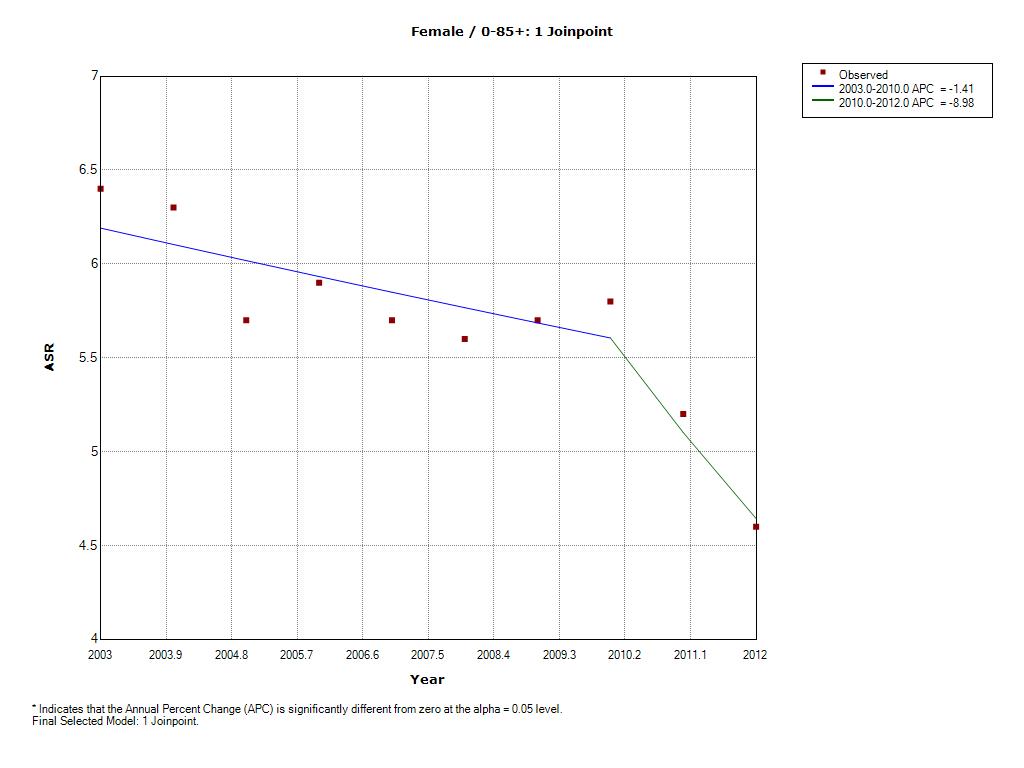

Supplement: Supplementary file 7 — Supplement Figure 7: incidence joinpoint. [file 12889_2024_19104_MOESM7_ESM.zip › Supplement Figure 7 incidence joinpoint/Israel female 0-85+.jpg]

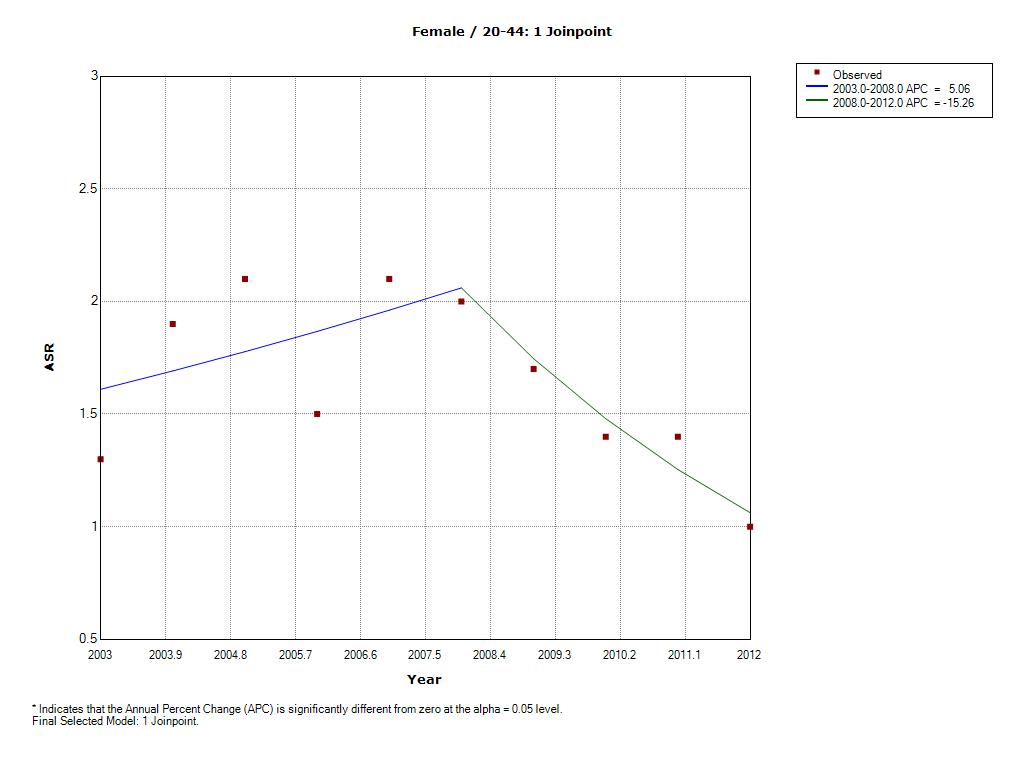

Supplement: Supplementary file 7 — Supplement Figure 7: incidence joinpoint. [file 12889_2024_19104_MOESM7_ESM.zip › Supplement Figure 7 incidence joinpoint/Israel female 20-44.jpg]

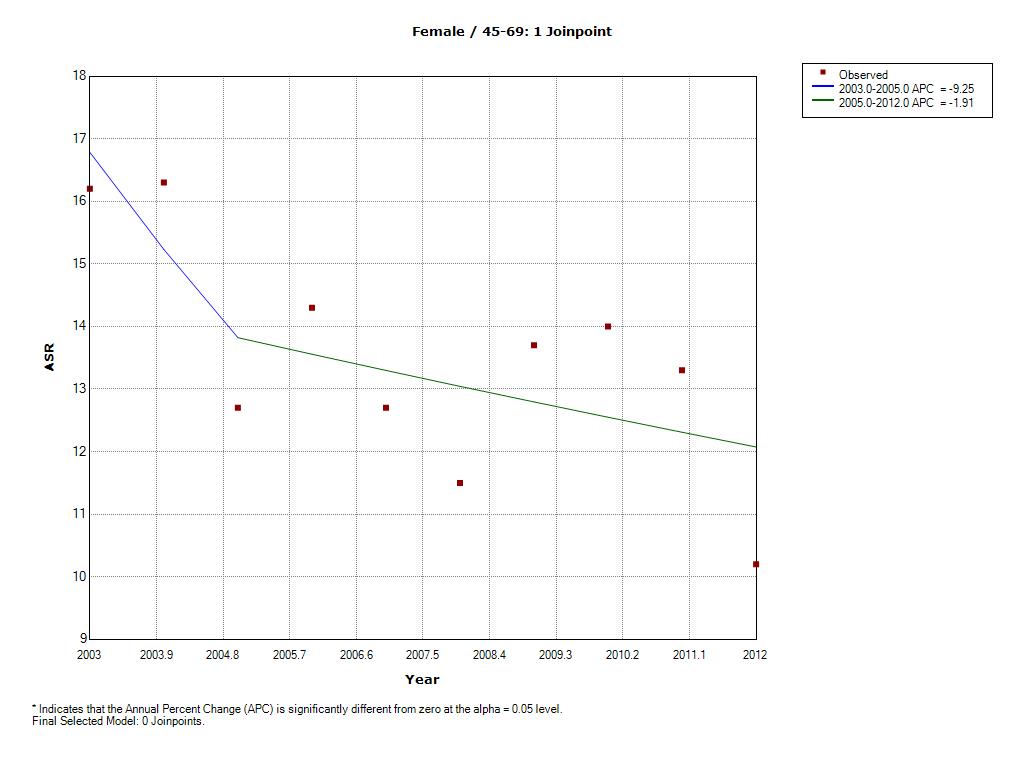

Supplement: Supplementary file 7 — Supplement Figure 7: incidence joinpoint. [file 12889_2024_19104_MOESM7_ESM.zip › Supplement Figure 7 incidence joinpoint/Israel female 45-69.jpg]

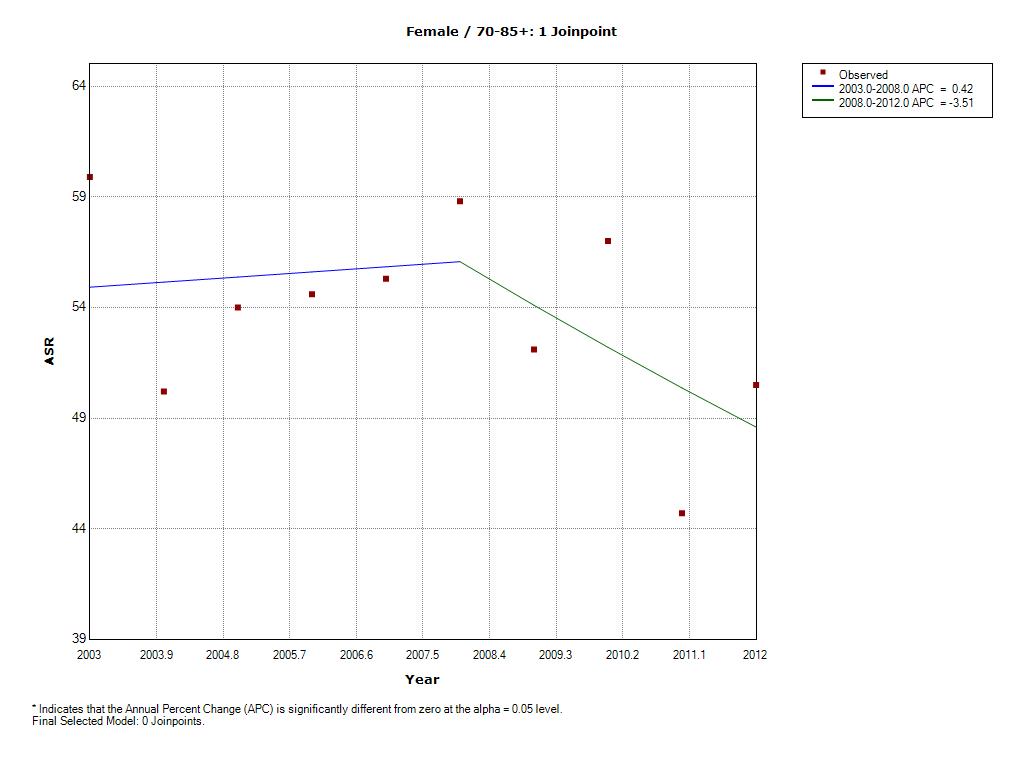

Supplement: Supplementary file 7 — Supplement Figure 7: incidence joinpoint. [file 12889_2024_19104_MOESM7_ESM.zip › Supplement Figure 7 incidence joinpoint/Israel female 70-85+.jpg]

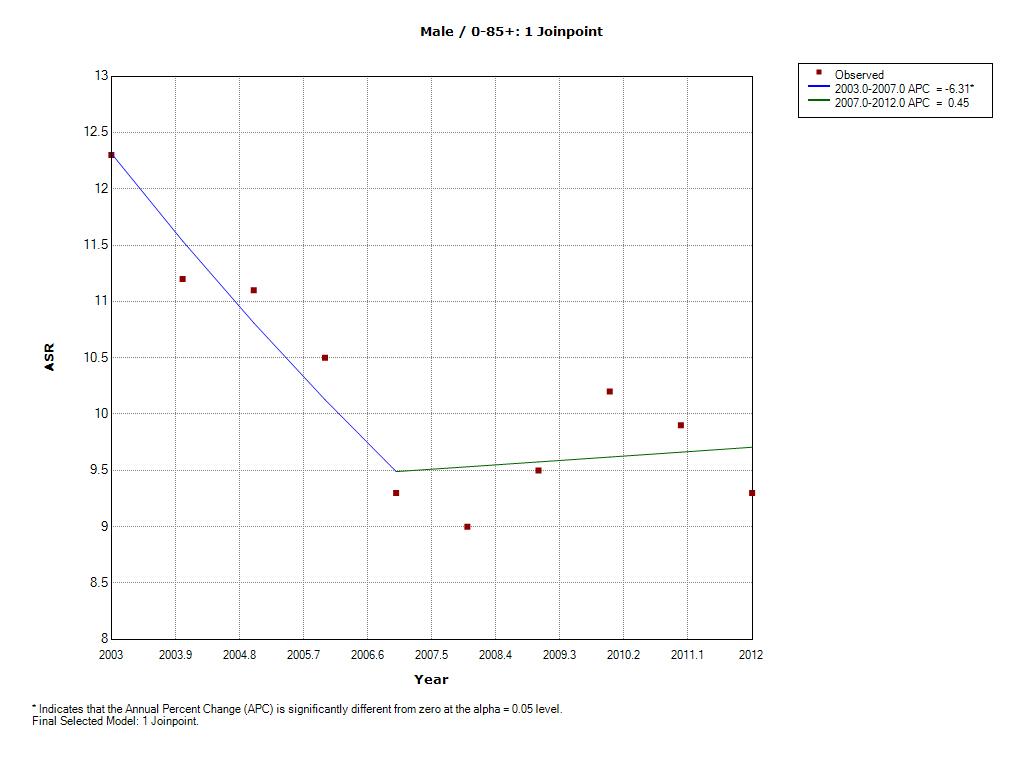

Supplement: Supplementary file 7 — Supplement Figure 7: incidence joinpoint. [file 12889_2024_19104_MOESM7_ESM.zip › Supplement Figure 7 incidence joinpoint/Israel male 0-85+.jpg]

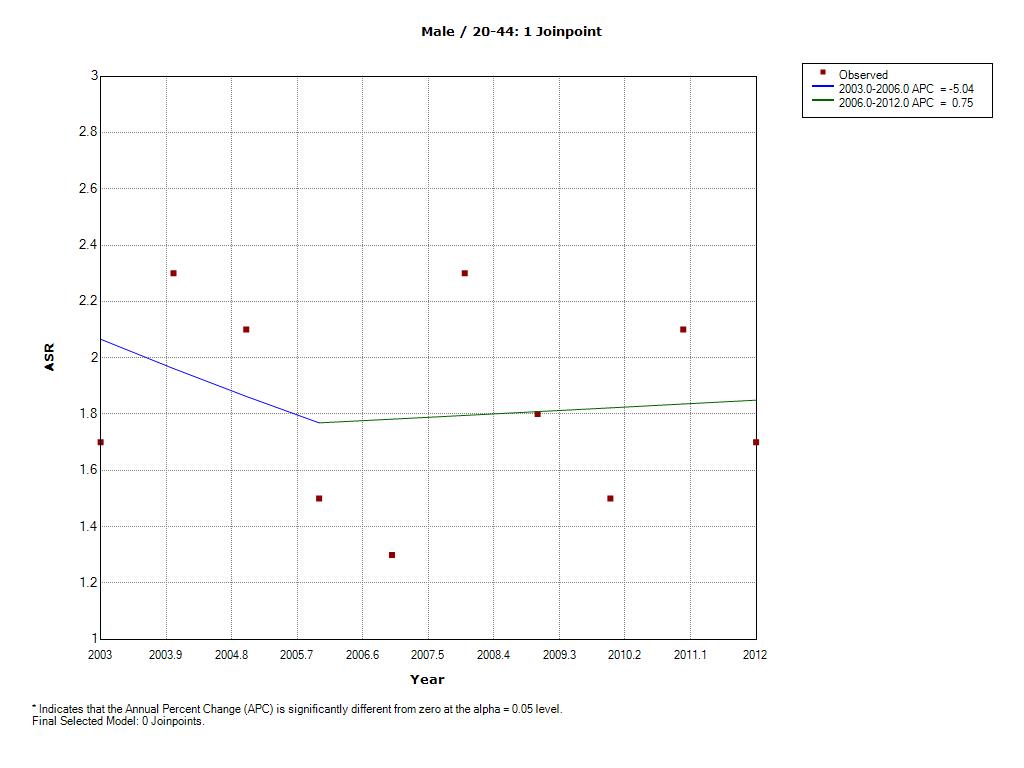

Supplement: Supplementary file 7 — Supplement Figure 7: incidence joinpoint. [file 12889_2024_19104_MOESM7_ESM.zip › Supplement Figure 7 incidence joinpoint/Israel male 20-44.jpg]

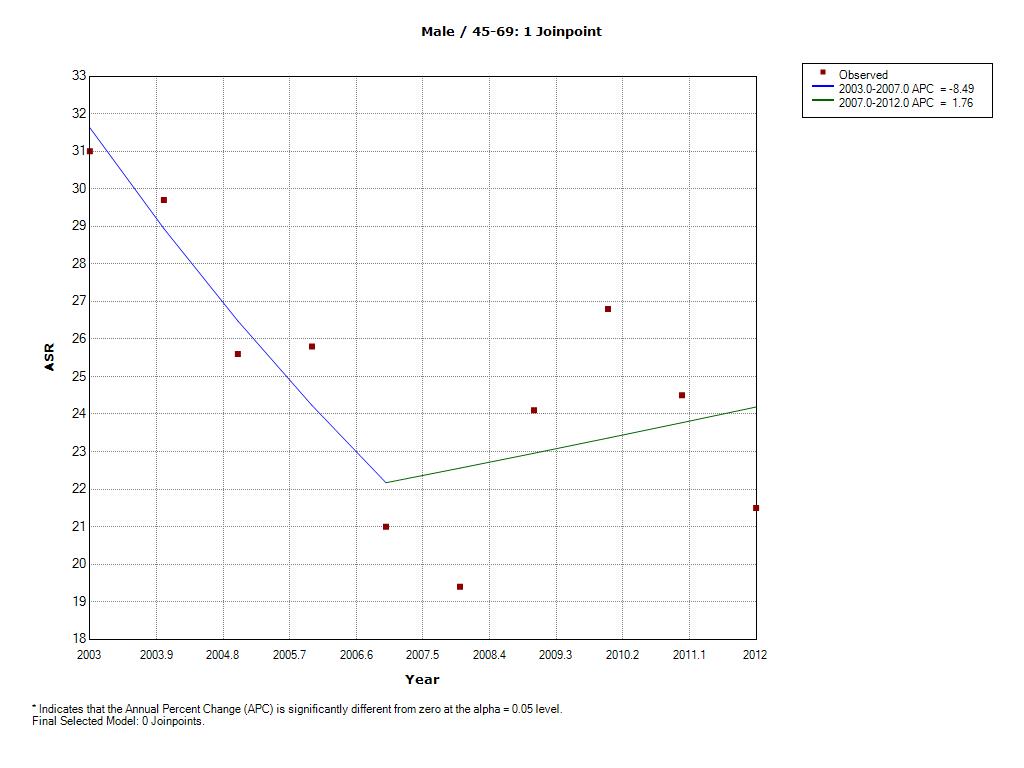

Supplement: Supplementary file 7 — Supplement Figure 7: incidence joinpoint. [file 12889_2024_19104_MOESM7_ESM.zip › Supplement Figure 7 incidence joinpoint/Israel male 45-69.jpg]

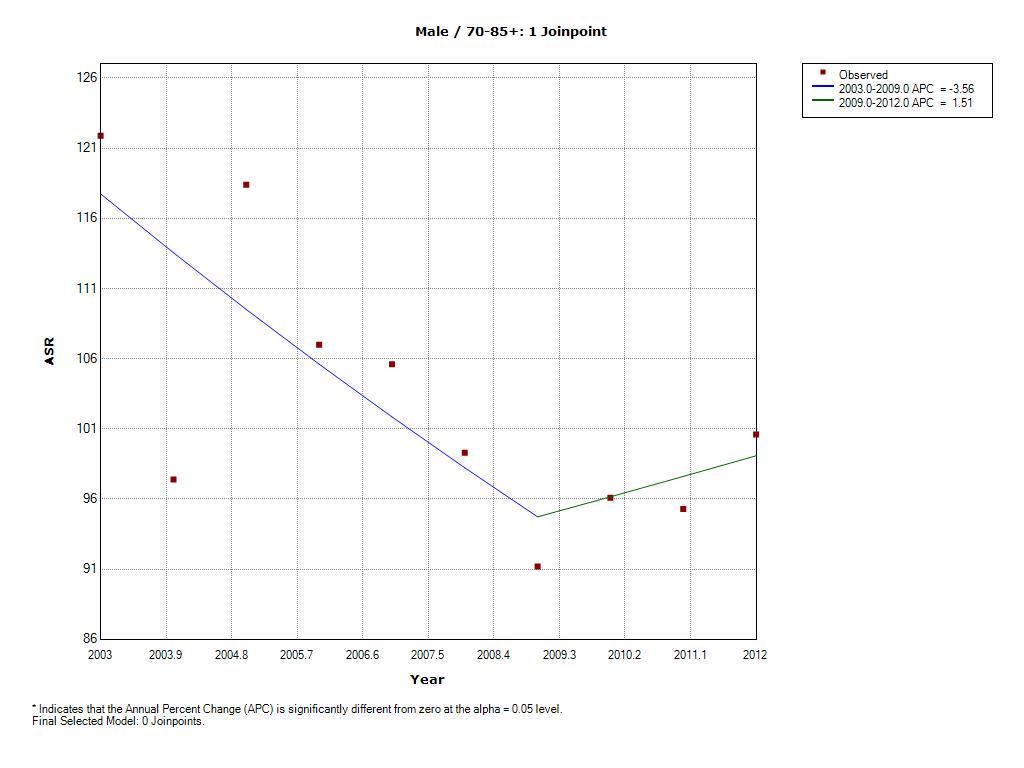

Supplement: Supplementary file 7 — Supplement Figure 7: incidence joinpoint. [file 12889_2024_19104_MOESM7_ESM.zip › Supplement Figure 7 incidence joinpoint/Israel male 70-85+.jpg]

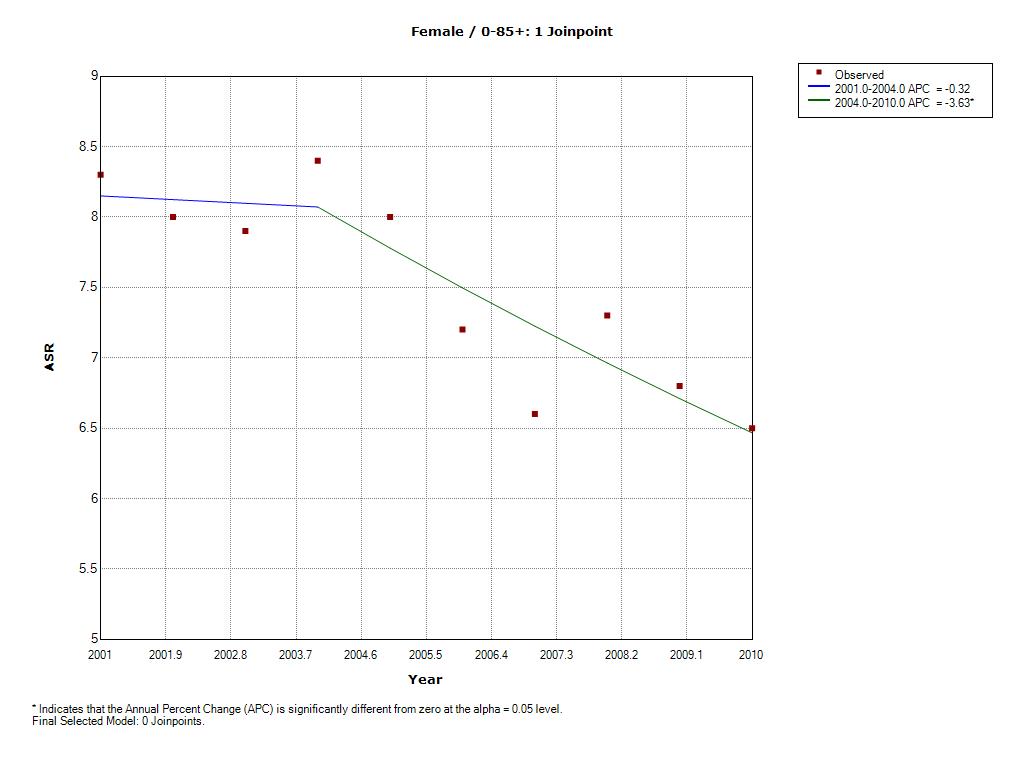

Supplement: Supplementary file 7 — Supplement Figure 7: incidence joinpoint. [file 12889_2024_19104_MOESM7_ESM.zip › Supplement Figure 7 incidence joinpoint/Italy female 0-85+.jpg]

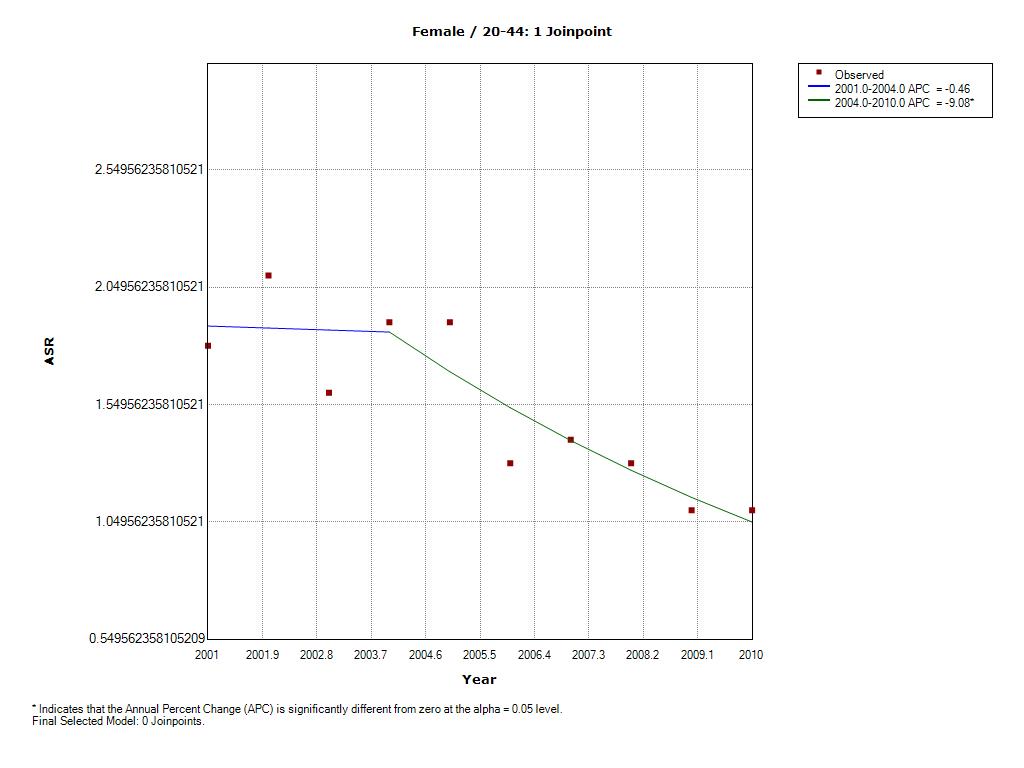

Supplement: Supplementary file 7 — Supplement Figure 7: incidence joinpoint. [file 12889_2024_19104_MOESM7_ESM.zip › Supplement Figure 7 incidence joinpoint/Italy female 20-44.jpg]

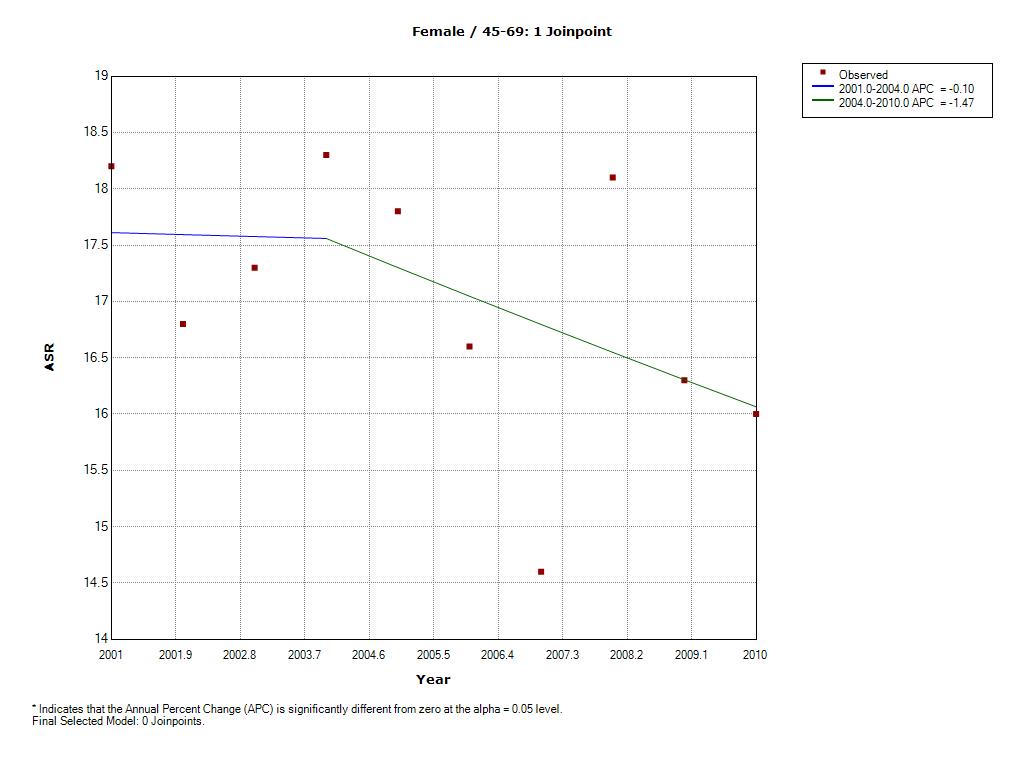

Supplement: Supplementary file 7 — Supplement Figure 7: incidence joinpoint. [file 12889_2024_19104_MOESM7_ESM.zip › Supplement Figure 7 incidence joinpoint/Italy female 45-69.jpg]

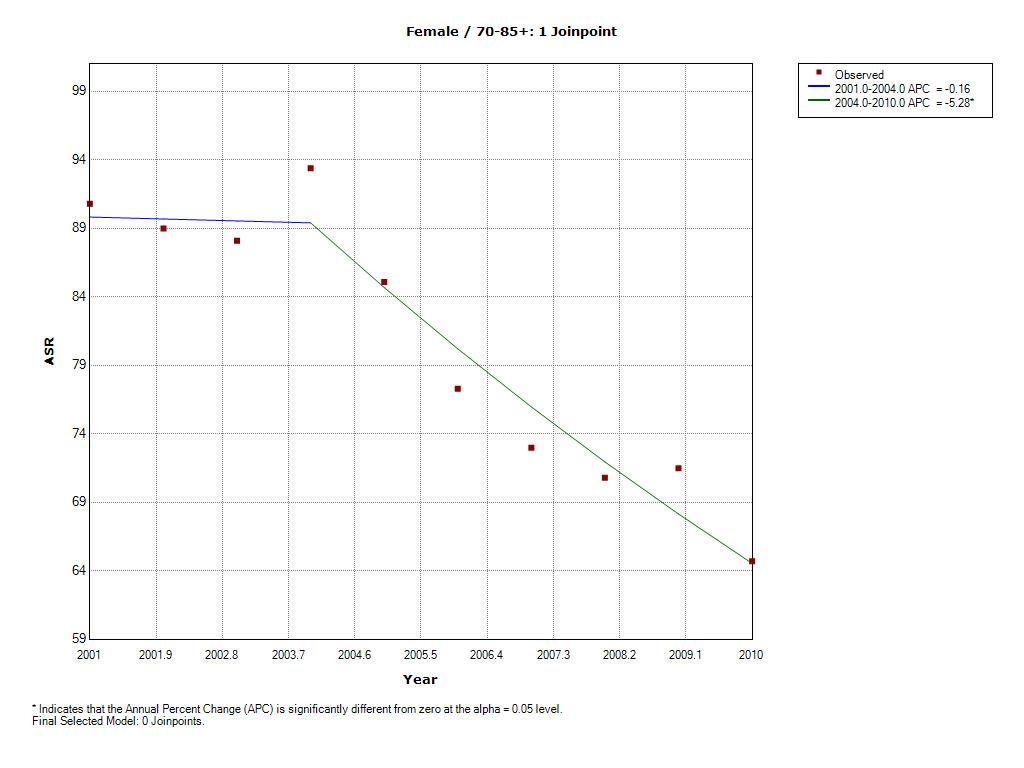

Supplement: Supplementary file 7 — Supplement Figure 7: incidence joinpoint. [file 12889_2024_19104_MOESM7_ESM.zip › Supplement Figure 7 incidence joinpoint/Italy female 70-85+.jpg]

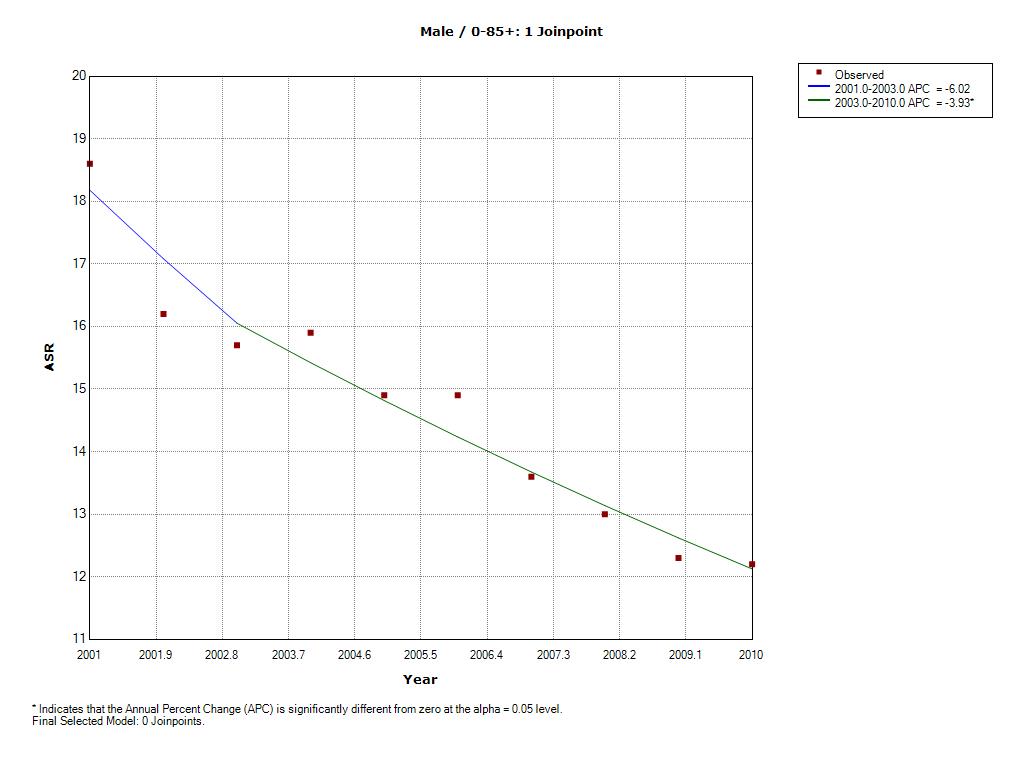

Supplement: Supplementary file 7 — Supplement Figure 7: incidence joinpoint. [file 12889_2024_19104_MOESM7_ESM.zip › Supplement Figure 7 incidence joinpoint/Italy male 0-85+.jpg]

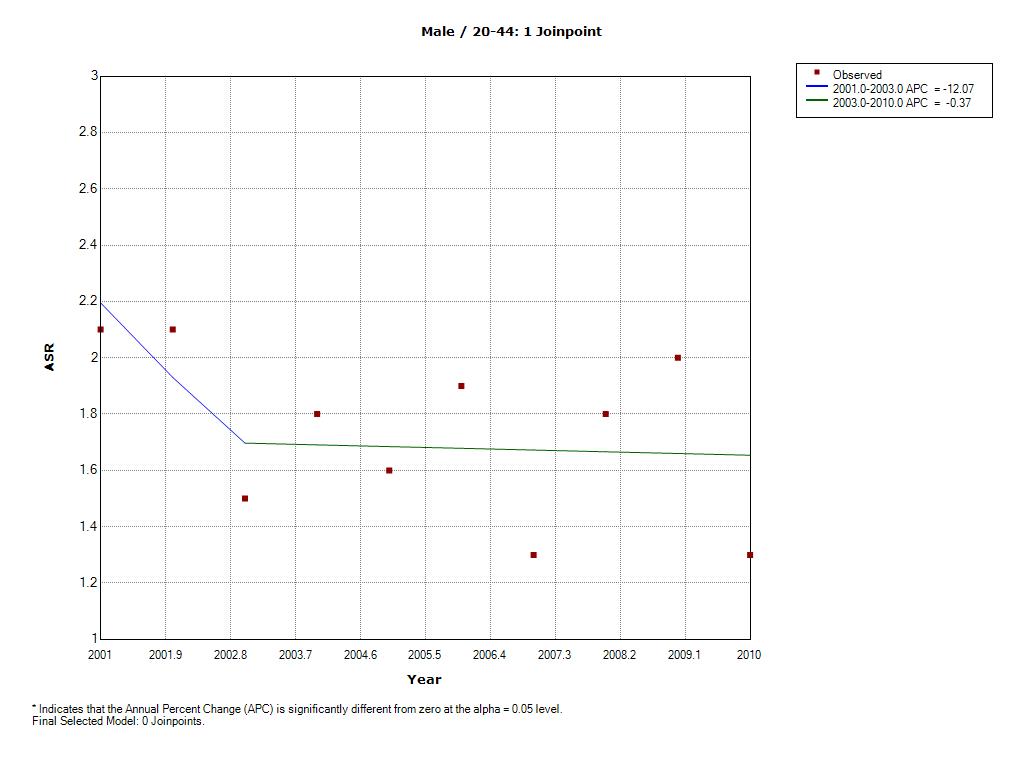

Supplement: Supplementary file 7 — Supplement Figure 7: incidence joinpoint. [file 12889_2024_19104_MOESM7_ESM.zip › Supplement Figure 7 incidence joinpoint/Italy male 20-44.jpg]

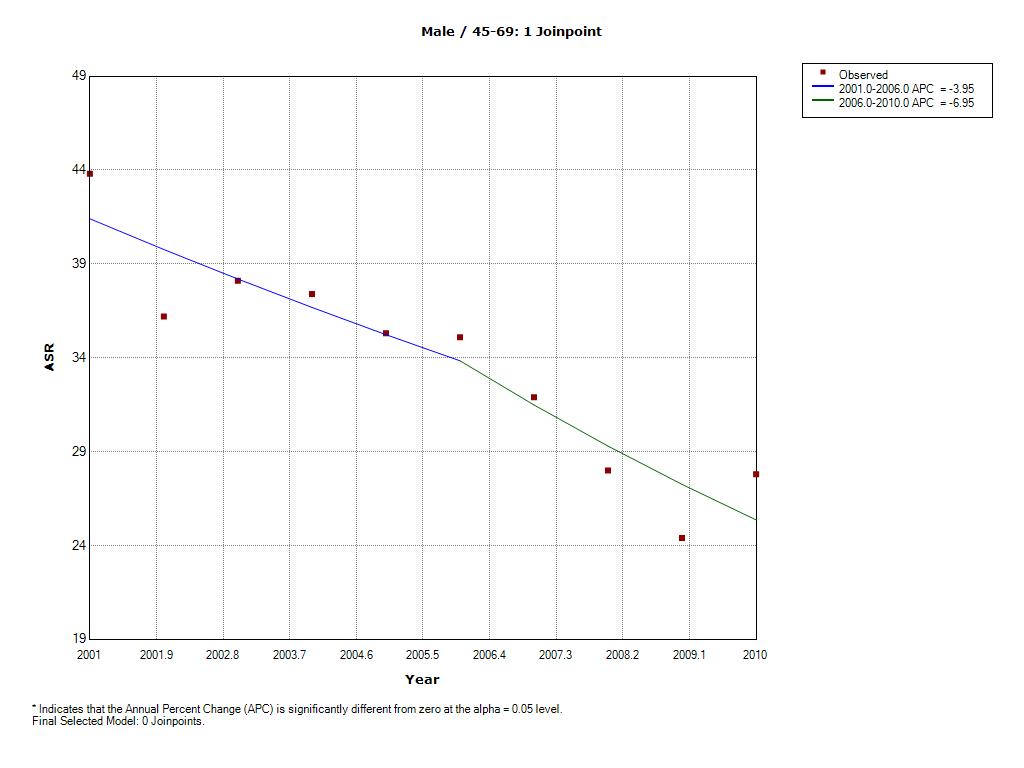

Supplement: Supplementary file 7 — Supplement Figure 7: incidence joinpoint. [file 12889_2024_19104_MOESM7_ESM.zip › Supplement Figure 7 incidence joinpoint/Italy male 45-69.jpg]

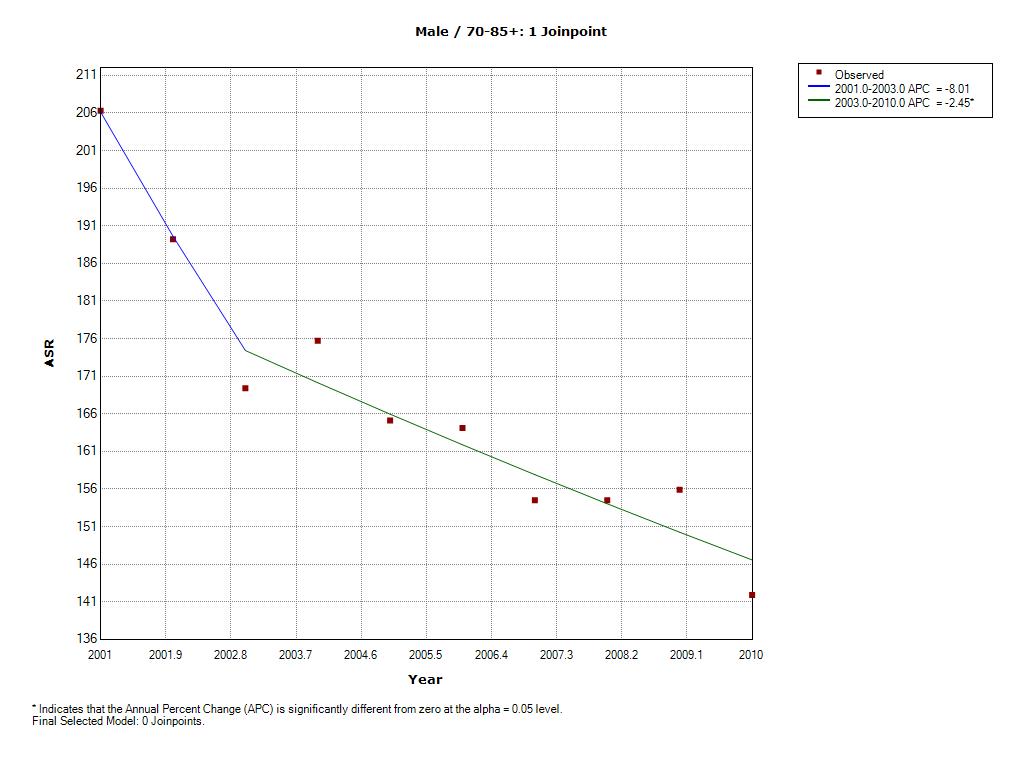

Supplement: Supplementary file 7 — Supplement Figure 7: incidence joinpoint. [file 12889_2024_19104_MOESM7_ESM.zip › Supplement Figure 7 incidence joinpoint/Italy male 70-85+.jpg]

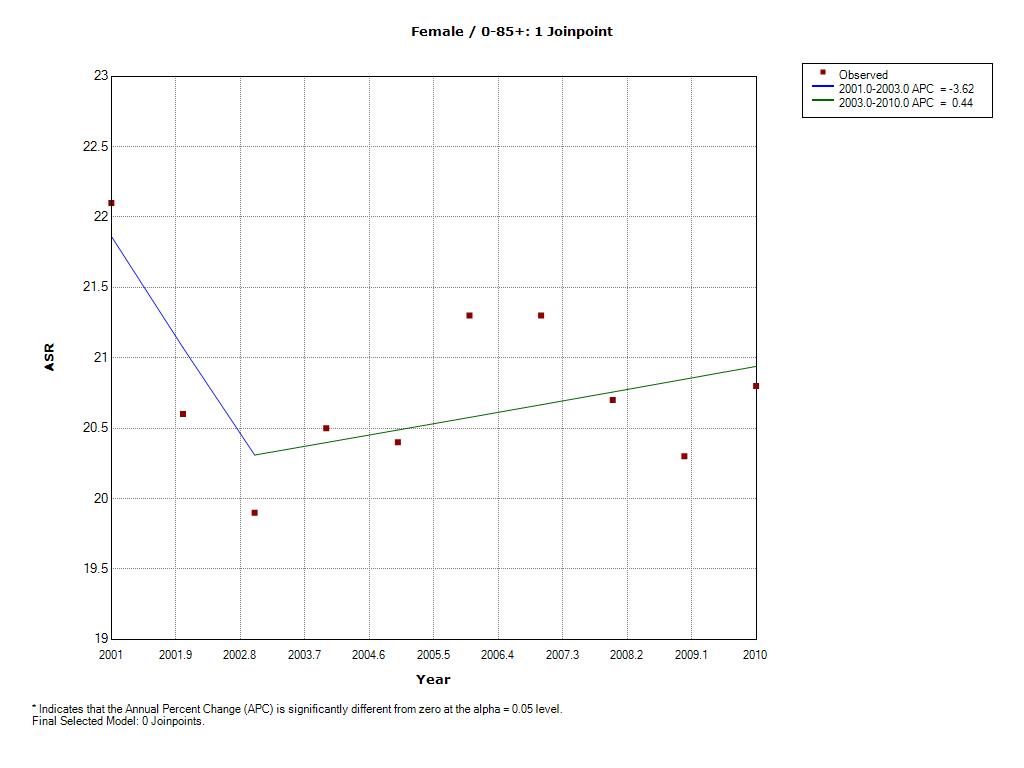

Supplement: Supplementary file 7 — Supplement Figure 7: incidence joinpoint. [file 12889_2024_19104_MOESM7_ESM.zip › Supplement Figure 7 incidence joinpoint/Japan female 0-85+.jpg]

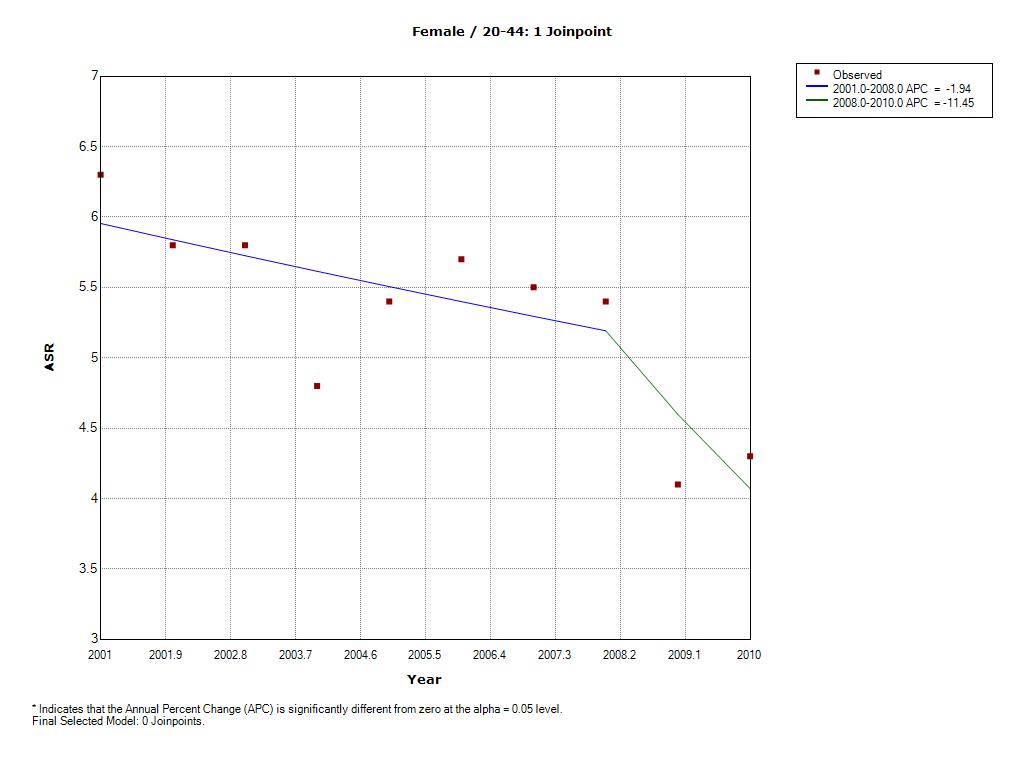

Supplement: Supplementary file 7 — Supplement Figure 7: incidence joinpoint. [file 12889_2024_19104_MOESM7_ESM.zip › Supplement Figure 7 incidence joinpoint/Japan female 20-44.jpg]

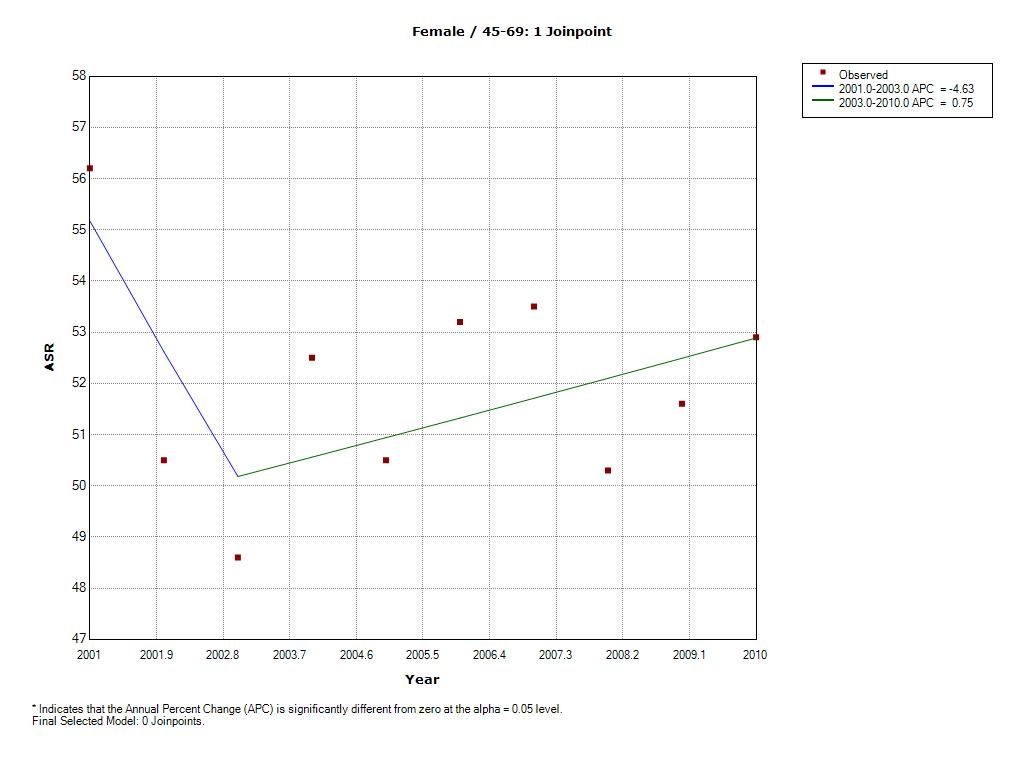

Supplement: Supplementary file 7 — Supplement Figure 7: incidence joinpoint. [file 12889_2024_19104_MOESM7_ESM.zip › Supplement Figure 7 incidence joinpoint/Japan female 45-69.jpg]

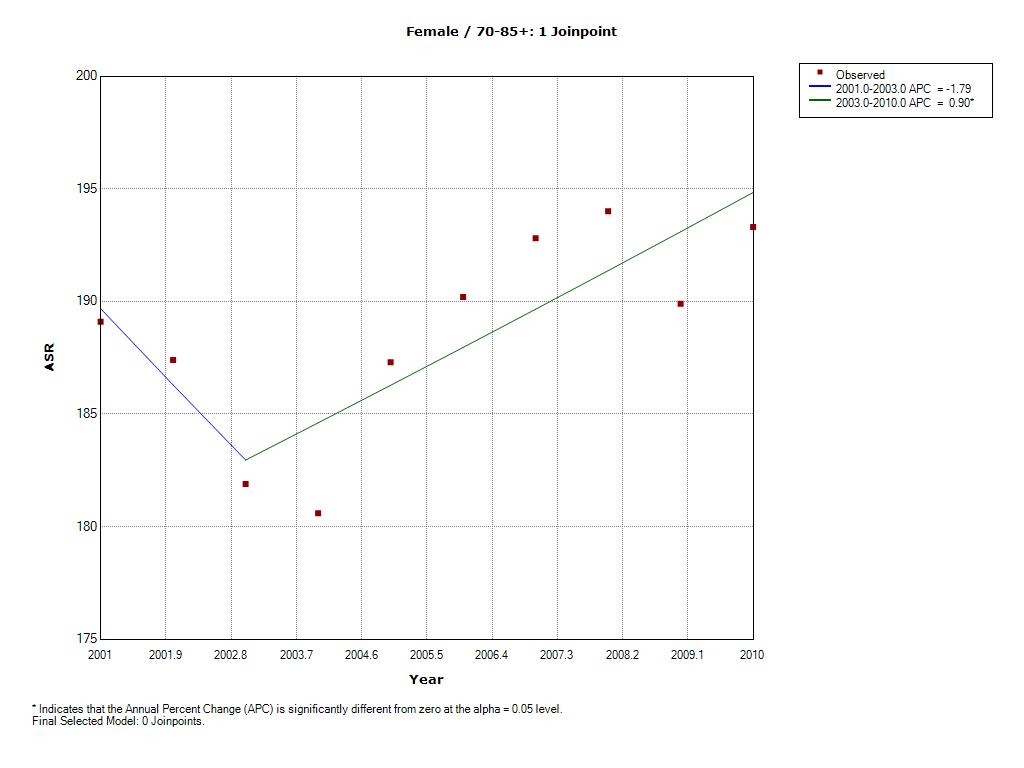

Supplement: Supplementary file 7 — Supplement Figure 7: incidence joinpoint. [file 12889_2024_19104_MOESM7_ESM.zip › Supplement Figure 7 incidence joinpoint/Japan female 70-85+.jpg]

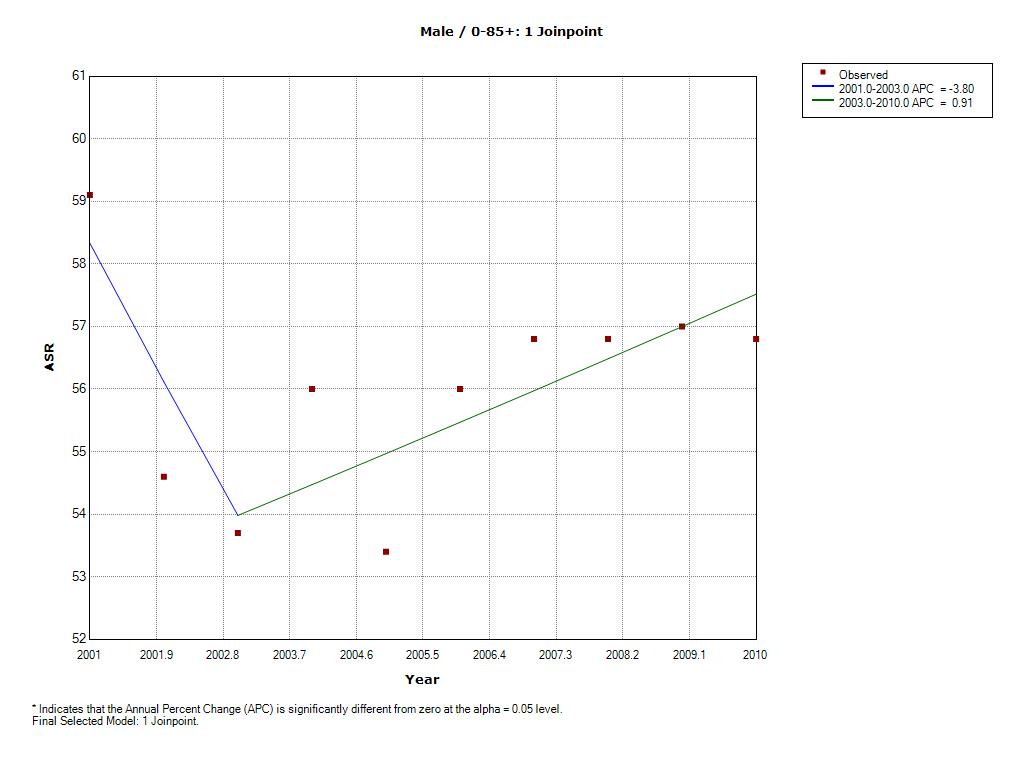

Supplement: Supplementary file 7 — Supplement Figure 7: incidence joinpoint. [file 12889_2024_19104_MOESM7_ESM.zip › Supplement Figure 7 incidence joinpoint/Japan male 0-85+.jpg]

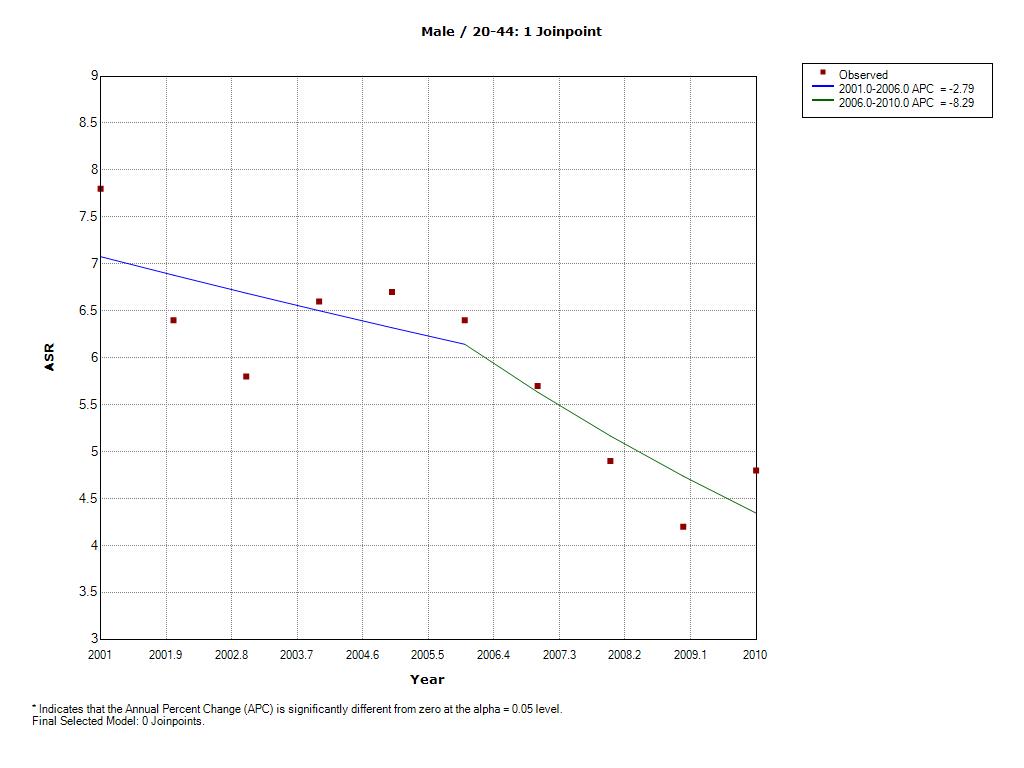

Supplement: Supplementary file 7 — Supplement Figure 7: incidence joinpoint. [file 12889_2024_19104_MOESM7_ESM.zip › Supplement Figure 7 incidence joinpoint/Japan male 20-44.jpg]

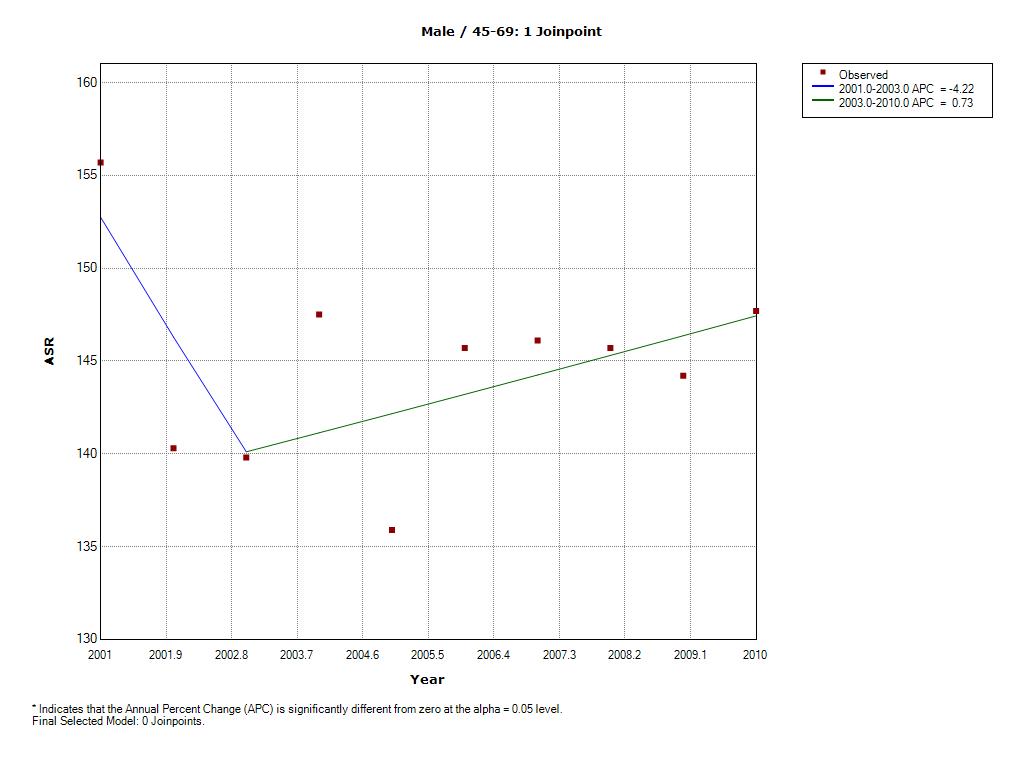

Supplement: Supplementary file 7 — Supplement Figure 7: incidence joinpoint. [file 12889_2024_19104_MOESM7_ESM.zip › Supplement Figure 7 incidence joinpoint/Japan male 45-69.jpg]

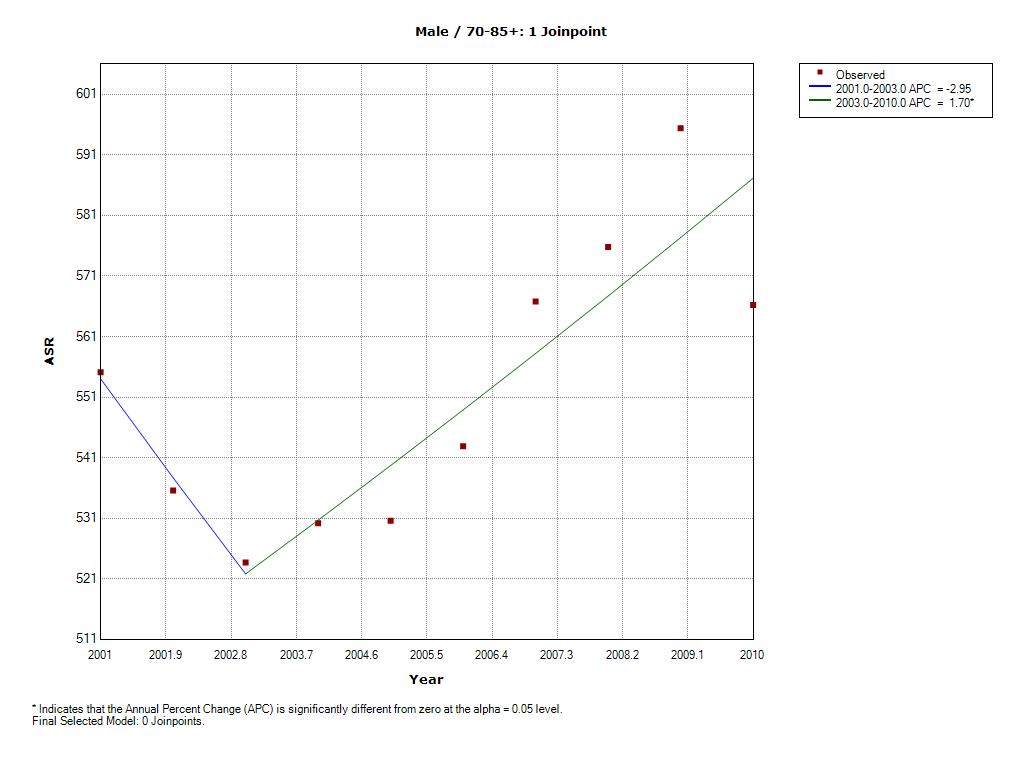

Supplement: Supplementary file 7 — Supplement Figure 7: incidence joinpoint. [file 12889_2024_19104_MOESM7_ESM.zip › Supplement Figure 7 incidence joinpoint/Japan male 70-85+.jpg]

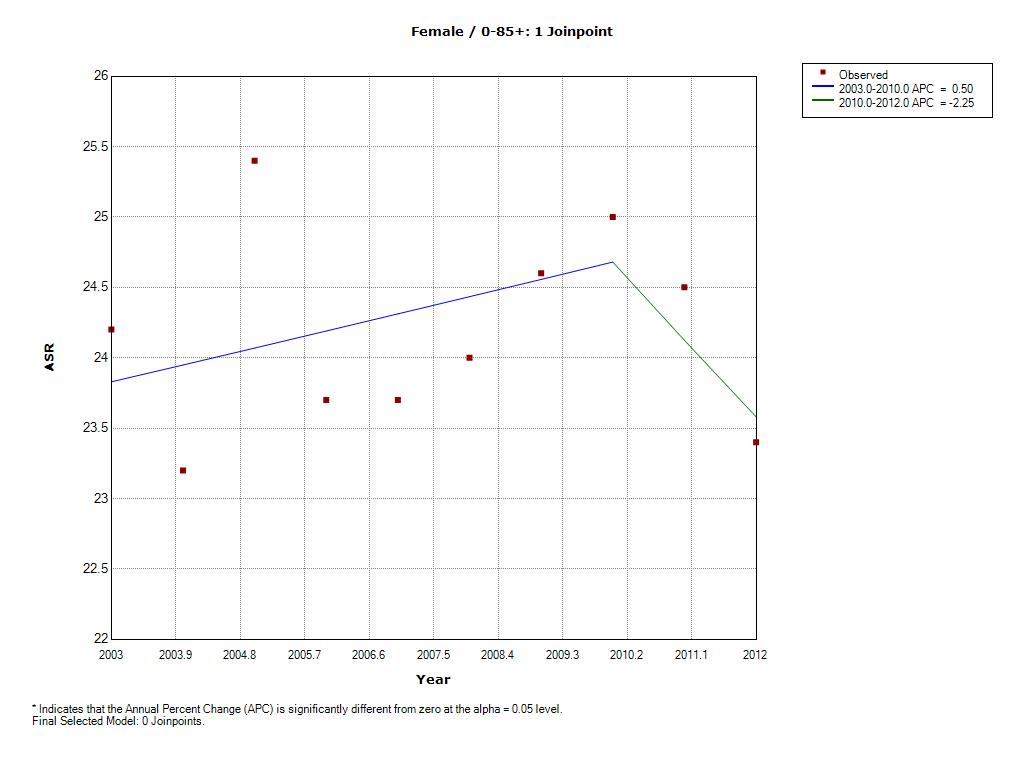

Supplement: Supplementary file 7 — Supplement Figure 7: incidence joinpoint. [file 12889_2024_19104_MOESM7_ESM.zip › Supplement Figure 7 incidence joinpoint/Korea female 0-85+.jpg]

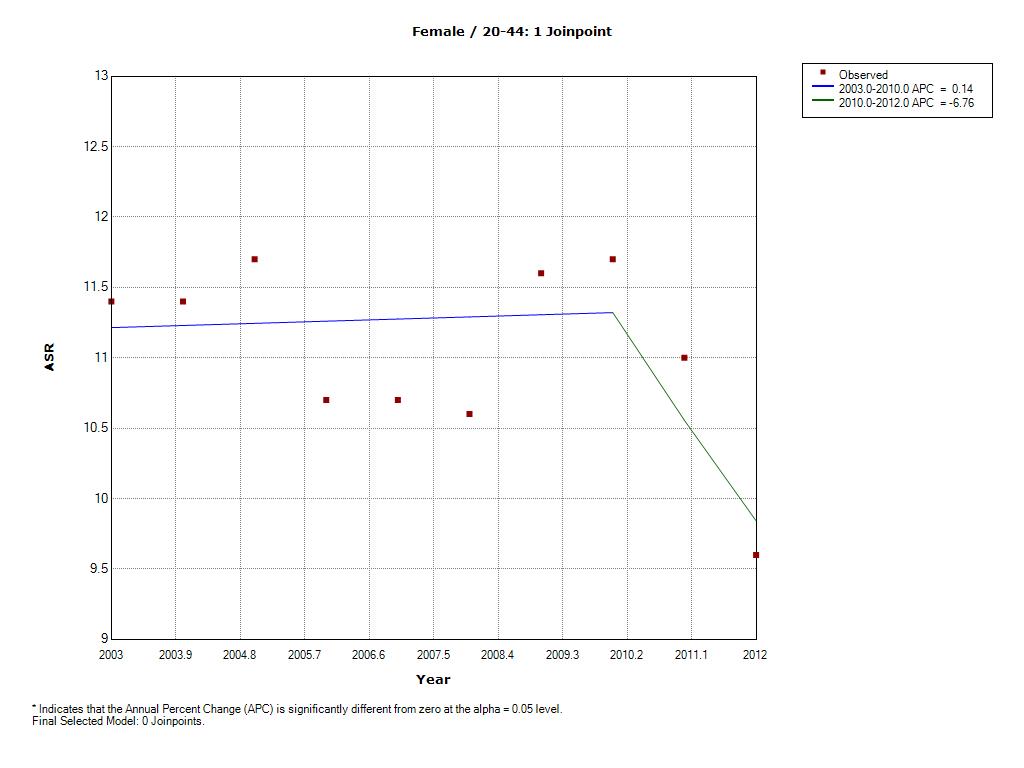

Supplement: Supplementary file 7 — Supplement Figure 7: incidence joinpoint. [file 12889_2024_19104_MOESM7_ESM.zip › Supplement Figure 7 incidence joinpoint/Korea female 20-44.jpg]

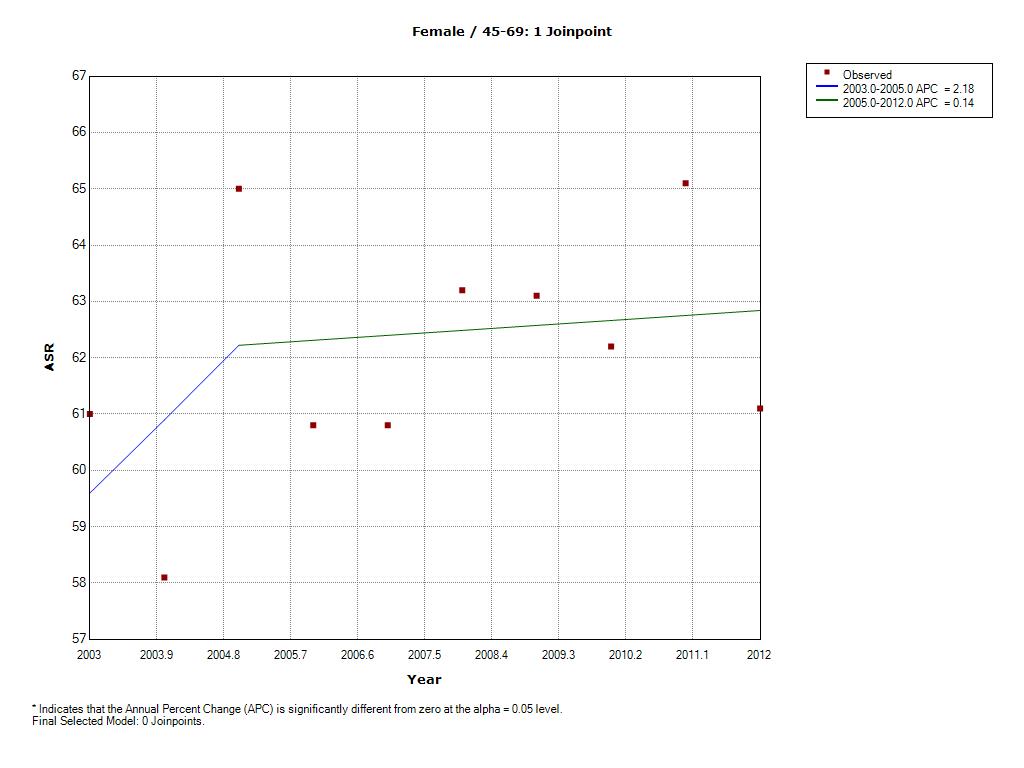

Supplement: Supplementary file 7 — Supplement Figure 7: incidence joinpoint. [file 12889_2024_19104_MOESM7_ESM.zip › Supplement Figure 7 incidence joinpoint/Korea female 45-69.jpg]

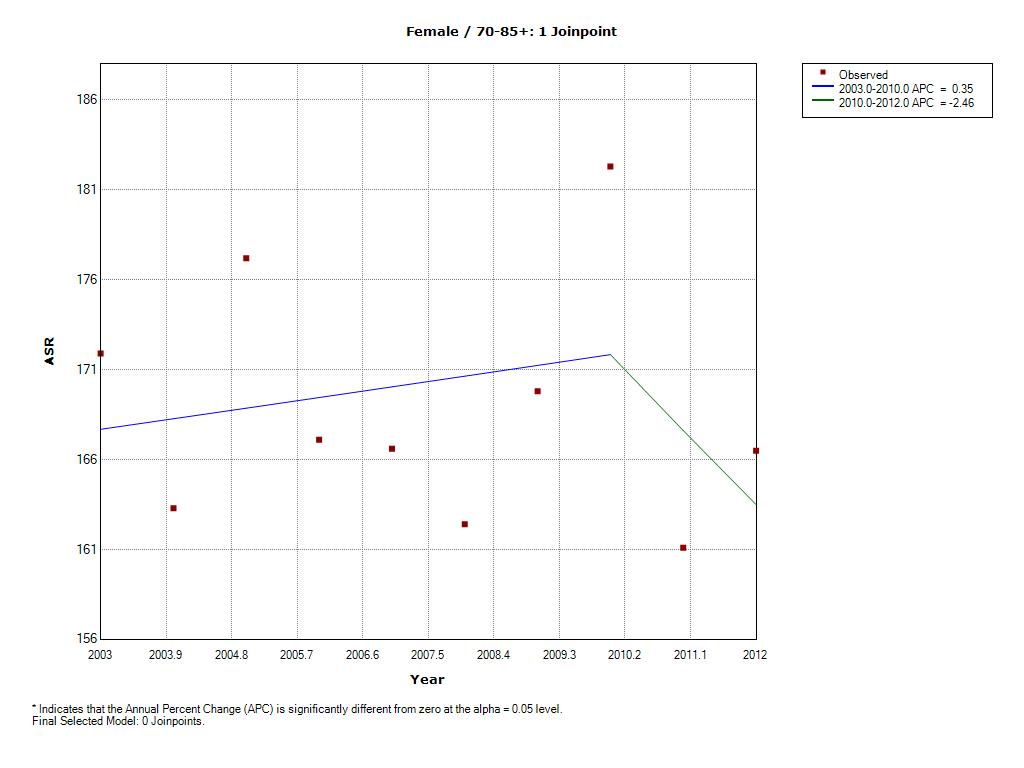

Supplement: Supplementary file 7 — Supplement Figure 7: incidence joinpoint. [file 12889_2024_19104_MOESM7_ESM.zip › Supplement Figure 7 incidence joinpoint/Korea female 70-85+.jpg]

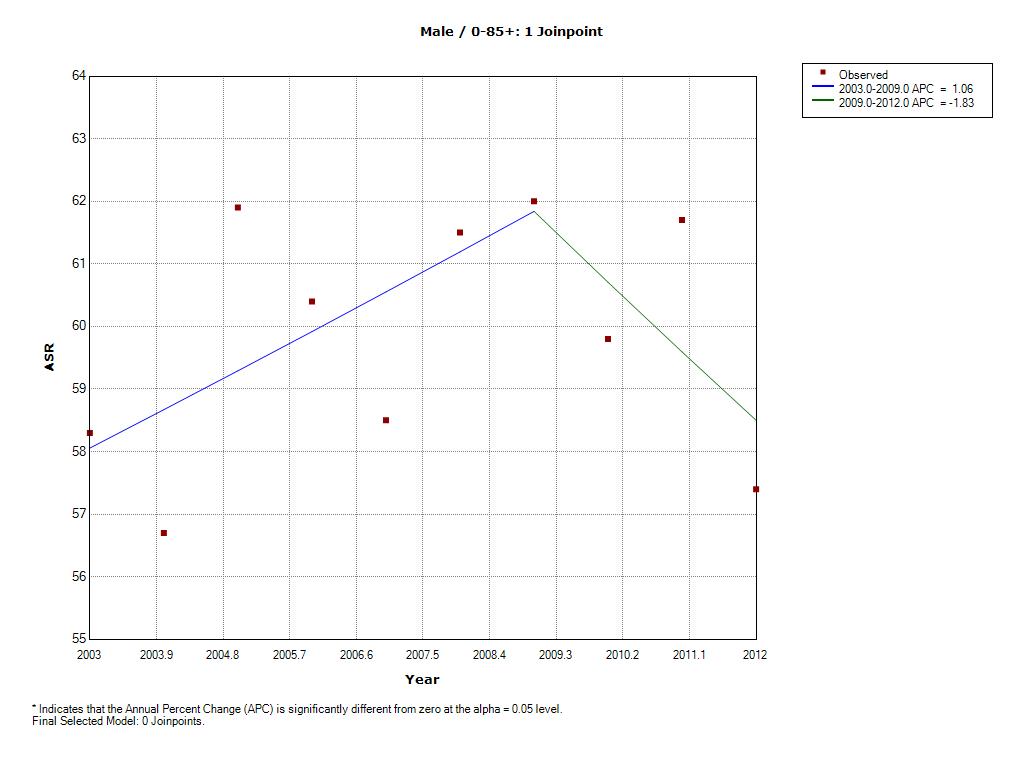

Supplement: Supplementary file 7 — Supplement Figure 7: incidence joinpoint. [file 12889_2024_19104_MOESM7_ESM.zip › Supplement Figure 7 incidence joinpoint/Korea male 0-85+.jpg]

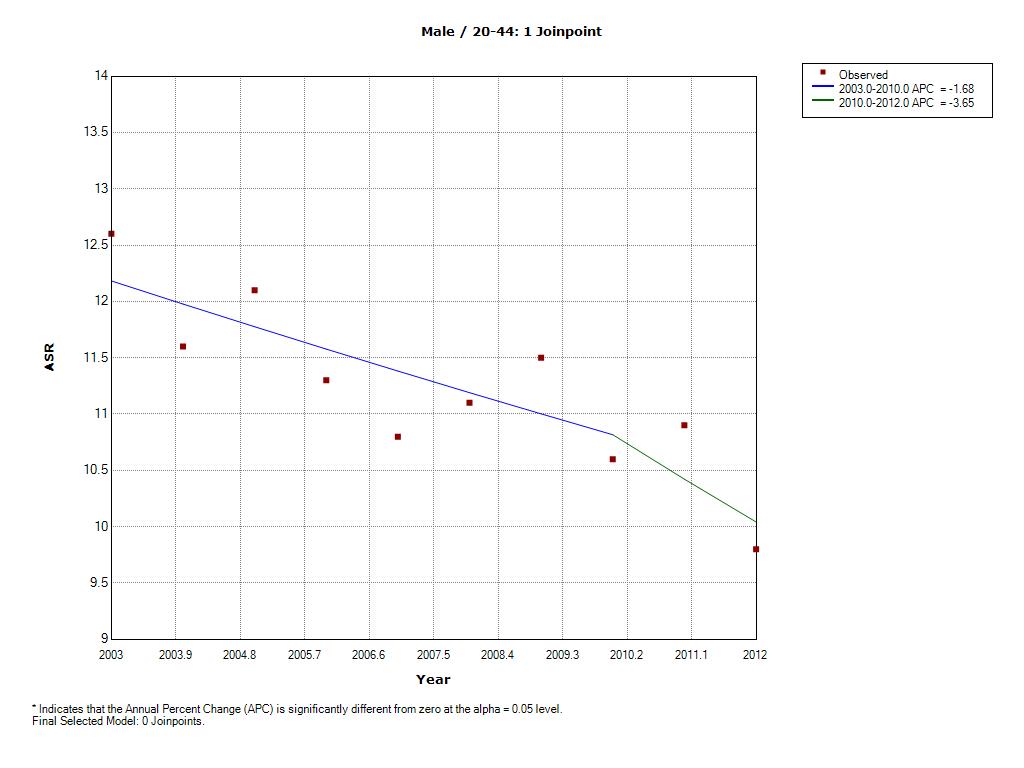

Supplement: Supplementary file 7 — Supplement Figure 7: incidence joinpoint. [file 12889_2024_19104_MOESM7_ESM.zip › Supplement Figure 7 incidence joinpoint/Korea male 20-44.jpg]
