# Supplementary material for: Global incidence and mortality trends of gastric cancer and predicted mortality of gastric cancer by 2035
Source: BMC Public Health. 2024 Jul 2;24:1763. doi: 10.1186/s12889-024-19104-6 (PMC11221210; doi:10.1186/s12889-024-19104-6)
Supplement: Supplementary file 9 — Supplementary material 9. [file 12889_2024_19104_MOESM9_ESM.docx]

| Supplement Table 1 AAPC of the Incidence of Gastric Cancer in Individuals 20 to 44 Years | | | | | | |
| --- | --- | --- | --- | --- | --- | --- |
| Region | Males | |  | Females | |  |
|  | APCC | -95% CI | 95% CI | APCC | -95% CI | 95% CI |
| South America |  |  |  |  |  |  |
| Brazil | 5.5 | -5 | 17.1 | -4.1 | -13.7 | 6.7 |
| Chile | -5.5 | -18 | 8.9 | -3 | -13.6 | 8.9 |
| Colombia | -2.3 | -8 | 3.8 | 0.6 | -3.8 | 5.1 |
| Ecuador | 0.6 | -5.8 | 7.4 | 4.0* | 0.5 | 7.6 |
| Northern America |  |  |  |  |  |  |
| Canada | -0.7 | -4.3 | 3 | 2.1 | -1.8 | 6.2 |
| USA | 0.5 | -1.1 | 2.1 | 2.0* | 0.8 | 3.2 |
| Eastern Asia |  |  |  |  |  |  |
| China | -5.3* | -7.8 | -2.7 | -1.1 | -4.3 | 2.3 |
| Japan | -5.0* | -7.5 | -2.5 | -3.3* | -5.6 | -1 |
| Philippines | -5.0* | -9.3 | -0.5 | -4.2 | -11.8 | 4.2 |
| India | -2.4 | -8.4 | 4 | 1.5 | -3.6 | 6.8 |
| South Korea | -2.0* | -2.9 | -1 | -0.9 | -2.3 | 0.6 |
| Southeastern Asia |  |  |  |  |  |  |
| Thailand | -0.7 | -7 | 6.1 | 4.5 | -2 | 11.4 |
| Eastern Europe |  |  |  |  |  |  |
| Belarus | -3.7* | -5.9 | -1.6 | -1.6 | -3.9 | 0.8 |
| Bulgaria | -1.8 | -7 | 3.7 | 0 | -5.8 | 6.1 |
| Czech Republic | -0.8 | -4.3 | 2.8 | -0.8 | -6.8 | 5.7 |
| Poland | -0.7 | -10.1 | 9.8 | 6.1 | -6.1 | 20 |
| Slovakia | -0.6 | -3.6 | 2.6 | 0.3 | -5.4 | 6.3 |
| Northern Europe |  |  |  |  |  |  |
| Denmark | -2 | -15.2 | 13.1 | -5.8 | -14.6 | 3.9 |
| Estonia | 0.1 | -7.9 | 8.7 | -7 | -22.9 | 12.1 |
| Iceland |  |  |  |  |  |  |
| Ireland | -1.1 | -12.8 | 12.1 | -4 | -9.9 | 2.3 |
| Lithuania | 0.6 | -5.2 | 6.6 | 1.9 | -5.7 | 10.1 |
| Sweden | 2.2 | -0.9 | 5.5 | -4.8 | -12.5 | 3.7 |
| Norway | -0.6 | -7.7 | 7.1 | 9 | -6.7 | 27.2 |
| Finland | -4.9 | -13.6 | 4.6 | -3.6 | -10.4 | 3.8 |
| UK | 0.8 | -2 | 3.6 | -0.1 | -3.8 | 3.8 |
| Western Asia |  |  |  |  |  |  |
| Cyprus |  |  |  |  |  |  |
| Southern Europe |  |  |  |  |  |  |
| Croatia | -2.9 | -8.8 | 3.3 | -0.3 | -4.7 | 4.3 |
| Italy | -2.4 | -6.5 | 1.9 | -6.6* | -9.6 | -3.5 |
| Malta |  |  |  |  |  |  |
| Slovenia | -1.2 | -9.6 | 8 | -1.7 | -7.1 | 4 |
| Spain | -3.8 | -8.6 | 1.2 | -2.3 | -8.1 | 3.9 |
| Turkey | -2 | -7.7 | 4.1 | 0.9 | -4.3 | 6.5 |
| Western Europe |  |  |  |  |  |  |
| Austria | -6.2* | -10.8 | -1.4 | -2.8 | -6.4 | 1 |
| France | -1.9 | -6.7 | 3.2 | -4.6 | -9.1 | 0.1 |
| Germany | -0.9 | -6.8 | 5.3 | -8.2 | -15.7 | 0 |
| The Netherlands | 0.2 | -3.4 | 3.9 | 3 | -3.2 | 9.6 |
| Switzerland | 4.3 | -6.2 | 16 | -4 | -13.8 | 6.9 |
| Oceania |  |  |  |  |  |  |
| Australia | 0.8 | -3.3 | 5.1 | -1.1 | -5.6 | 3.6 |
| New Zealand | 0.6 | -4.5 | 6.1 | -1.5 | -8.3 | 5.8 |
| *：P<0.05 |  |  |  |  |  |  |

| Supplement Table 2 AAPC of the Incidence of Gastric Cancer in Individuals 44 to 69 Years | | | | | | |
| --- | --- | --- | --- | --- | --- | --- |
|  | Males | |  | Females | |  |
| Country | APCC | -95% CI | 95% CI | APCC | -95% CI | 95% CI |
| South America |  |  |  |  |  |  |
| Brazil | -4 | -9.9 | 2.3 | -6.1 | -12.8 | 1 |
| Chile | -1.4 | -6 | 3.4 | -2.8 | -7.4 | 2 |
| Colombia | -5.0* | -6.7 | -3.2 | -6.2* | -8.8 | -3.6 |
| Ecuador | -0.8 | -6.3 | 5 | 1.3 | -5.7 | 8.8 |
| Northern America |  |  |  |  |  |  |
| Canada | 0.9* | 0 | 1.9 | 3.1* | 1.7 | 4.5 |
| USA | -0.7* | -1.2 | -0.2 | -0.3 | -0.9 | 0.3 |
| Eastern Asia |  |  |  |  |  |  |
| China | -1.7* | -2.7 | -0.7 | -2.8* | -4 | -1.5 |
| Japan | 0 | -1 | 0.9 | -0.1 | -1.2 | 1 |
| Philippines | -9.0* | -11.2 | -6.6 | -8.1* | -11.9 | -4.2 |
| India | -1.2 | -4.2 | 1.8 | -1.9 | -5.3 | 1.8 |
| South Korea | 0.4 | -0.5 | 1.3 | 0.4 | -0.4 | 1.3 |
| Southeastern Asia |  |  |  |  |  |  |
| Thailand | 0.9 | -2.7 | 4.5 | 1.7 | -2 | 5.6 |
| Eastern Europe |  |  |  |  |  |  |
| Belarus | -2.3* | -3 | -1.5 | -2.7* | -3.8 | -1.5 |
| Bulgaria | -2.8* | -4.4 | -1.2 | -2.2* | -4 | -0.3 |
| Czech Republic | -3.1* | -4.7 | -1.6 | -1 | -2.5 | 0.5 |
| Poland | -4.0* | -6 | -1.9 | -1.3 | -7.1 | 4.9 |
| Slovakia | -3.1* | -4.9 | -1.3 | 4.3 | -7.5 | 17.5 |
| Northern Europe |  |  |  |  |  |  |
| Denmark | -1.1 | -3.5 | 1.3 | 1 | -1.1 | 3.2 |
| Estonia | -3.8 | -7.5 | 0.1 | -3.7 | -8.3 | 1.2 |
| Iceland | -1 | -11.1 | 10.2 | -1.8 | -10.6 | 7.8 |
| Ireland | -0.3 | -1.5 | 0.9 | -1.4 | -3.8 | 1.1 |
| Lithuania | -2.9* | -4.4 | -1.4 | -1.8* | -3.3 | -0.2 |
| Sweden | -1.4 | -3.4 | 0.7 | -1.9* | -3.2 | -0.7 |
| Norway | -2.2 | -4.8 | 0.4 | -3.9* | -6 | -1.7 |
| Finland | -2.7* | -4.6 | -0.8 | -2.6* | -3.9 | -1.2 |
| UK | -3.7* | -4.4 | -3 | -3.3* | -4.4 | -2.2 |
| Western Asia |  |  |  |  |  |  |
| Cyprus | 1.3 | -5.5 | 8.6 | 4.3 | -2.9 | 11.9 |
| Southern Europe |  |  |  |  |  |  |
| Croatia | -3.6* | -4.9 | -2.2 | -2.3* | -4.3 | -0.4 |
| Italy | -5.2* | -6.8 | -3.5 | -1.1 | -2.7 | 0.5 |
| Malta | 0.9 | -3.3 | 5.2 | -0.3 | -9.6 | 10 |
| Slovenia | -2.8* | -4.5 | -1 | 0.3 | -2.3 | 3.1 |
| Spain | -2.4* | -4 | -0.8 | -2.7* | -4.5 | -0.9 |
| Israel | -2.7 | -5.6 | 0.3 | -3.1* | -5.7 | -0.4 |
| Turkey | -0.3 | -1.8 | 1.2 | -0.4 | -2.5 | 1.8 |
| Western Europe |  |  |  |  |  |  |
| Austria | -0.8 | -1.8 | 0.2 | -2.9* | -4.6 | -1.2 |
| France | -0.6 | -3.1 | 1.9 | -1.9* | -3 | -0.8 |
| Germany | 0.4 | -3.2 | 4.2 | -1.1 | -2.8 | 0.7 |
| The Netherlands | -2.7* | -3.9 | -1.4 | -1.1 | -2.5 | 0.3 |
| Switzerland | 1.2 | -0.9 | 3.4 | 3.7 | 0 | 7.6 |
| Oceania |  |  |  |  |  |  |
| Australia | -1.1* | -2 | -0.2 | -1.1 | -2.5 | 0.3 |
| New Zealand | -2.9* | -4.6 | -1.1 | -1.1 | -3 | 0.9 |
| *：P<0.05 |  |  |  |  |  |  |

| Supplement Table 3 AAPC of the Incidence of Gastric Cancer in Individuals 70 to 85 Years or Older | | | | | | |
| --- | --- | --- | --- | --- | --- | --- |
|  | Males | |  | Females | |  |
| Country | APCC | -95% CI | 95% CI | APCC | -95% CI | 95% CI |
| South America |  |  |  |  |  |  |
| Brazil | -7.0* | -11.3 | -2.4 | -8.2 | -16.8 | 1.2 |
| Chile | -2.5 | -7.2 | 2.3 | 1.1 | -6.1 | 8.9 |
| Colombia | -2.6 | -5.5 | 0.3 | -4.1* | -6.3 | -1.9 |
| Ecuador | -1.1 | -5.5 | 3.5 | -1.2 | -5.4 | 3.2 |
| Northern America |  |  |  |  |  |  |
| Canada | -0.3 | -1 | 0.5 | 0 | -1.4 | 1.4 |
| USA | -1.5* | -1.9 | -1.1 | -1.8* | -2.4 | -1.1 |
| Eastern Asia |  |  |  |  |  |  |
| China | -4.5* | -5.3 | -3.6 | -4.1* | -4.8 | -3.5 |
| Japan | 1.0* | 0.2 | 1.9 | 0.5* | 0 | 1 |
| Philippines | -8.6* | -13.3 | -3.8 | -7.1* | -10.1 | -4 |
| India | 3.2 | -1.4 | 8 | 5.4 | -0.9 | 12.1 |
| South Korea | 0.1 | -1 | 1.2 | -0.1 | -1.2 | 1 |
| Southeastern Asia |  |  |  |  |  |  |
| Thailand | -0.2 | -5.1 | 4.9 | 1.8 | -2.7 | 6.4 |
| Eastern Europe |  |  |  |  |  |  |
| Belarus | -0.7 | -1.7 | 0.4 | -0.5 | -1.7 | 0.8 |
| Bulgaria | -2.2* | -3.6 | -0.8 | -2.7* | -4.1 | -1.2 |
| Czech Republic | -4.0* | -5.3 | -2.7 | -3.6* | -4.5 | -2.7 |
| Poland | -4.4* | -7.4 | -1.3 | -5.3* | -8.9 | -1.6 |
| Slovakia | -1.3 | -3 | 0.5 | -1.2 | -3.4 | 0.9 |
| Northern Europe |  |  |  |  |  |  |
| Denmark | -1.3 | -3.3 | 0.8 | -0.2 | -2.2 | 1.8 |
| Estonia | -0.4 | -3.7 | 3 | -3.3 | -8 | 1.6 |
| Iceland | -4.6 | -12.9 | 4.4 | -5.4 | -11.9 | 1.5 |
| Ireland | 0.6 | -1 | 2.2 | -0.5 | -2.2 | 1.3 |
| Lithuania | -1.3* | -2.4 | -0.3 | -2.1* | -3.7 | -0.4 |
| Sweden | -3.0* | -5.1 | -0.9 | -3.2* | -4.9 | -1.5 |
| Norway | -2.8* | -5.1 | -0.4 | -5.4* | -8.2 | -2.4 |
| Finland | -5.1* | -6.7 | -3.5 | -4.0* | -5.5 | -2.4 |
| UK | -3.4* | -3.9 | -2.9 | -3.1* | -3.8 | -2.4 |
| Western Asia |  |  |  |  |  |  |
| Cyprus | -2 | -9 | 5.5 | 8.3 | -3.7 | 21.9 |
| Southern Europe |  |  |  |  |  |  |
| Croatia | -3.7* | -5.3 | -2.1 | -2.9* | -4.1 | -1.6 |
| Italy | -3.4* | -4.3 | -2.4 | -3.9* | -5.1 | -2.6 |
| Malta | -0.8 | -5.6 | 4.4 | -4.7 | -9.9 | 0.8 |
| Slovenia | -3.1* | -4.9 | -1.3 | -3.8* | -6.2 | -1.3 |
| Spain | -2.6* | -3.5 | -1.6 | -0.9 | -2.7 | 0.9 |
| Israel | -2.1* | -3.9 | -0.3 | -1.3 | -3.3 | 0.8 |
| Turkey | -0.4 | -2.6 | 1.9 | -3.1 | -6.7 | 0.5 |
| Western Europe |  |  |  |  |  |  |
| Austria | -4.8* | -5.9 | -3.7 | -4.9* | -6.5 | -3.4 |
| France | -2.5* | -3.9 | -1.1 | -2.4 | -5 | 0.4 |
| Germany | -2.9* | -5 | -0.8 | -2.1 | -4.5 | 0.4 |
| The Netherlands | -2.8* | -3.4 | -2.2 | -2.4* | -3.6 | -1.1 |
| Switzerland | 6.7 | -5.8 | 20.7 | -5.7 | -11.3 | 0.1 |
| Oceania |  |  |  |  |  |  |
| Australia | -2.6* | -3.8 | -1.4 | -1.4* | -2.6 | -0.2 |
| New Zealand | -1.4 | -4.1 | 1.5 | -2.9 | -5.9 | 0.2 |
| *：P<0.05 |  |  |  |  |  |  |

| Supplement Table 4 AAPC of the mortality of Gastric Cancer in Individuals 20 to 44 Years | | | | | | |
| --- | --- | --- | --- | --- | --- | --- |
| Region | Males | |  | Females | |  |
|  | APCC | -95% CI | 95% CI | APCC | -95% CI | 95% CI |
| South America |  |  |  |  |  |  |
| Brazil | -0.5 | -1.3 | 0.3 | 0.2 | -0.8 | 1.2 |
| Chile | -4.5* | -8.7 | -0.1 | 1 | -2.5 | 4.6 |
| Colombia | 1 | -0.3 | 2.4 | 0.9 | -0.9 | 2.7 |
| Ecuador | -2.800 | -10.7 | 5.9 | 0.4 | -3.9 | 4.9 |
| Northern America |  |  |  |  |  |  |
| Canada | -4.3* | -7.5 | -1 | -2.1 | -7.8 | 3.9 |
| USA | 0.4 | -0.3 | 1.2 | -1 | -2.8 | 0.9 |
| Eastern Asia |  |  |  |  |  |  |
| China | -2.9 | -9.6 | 4.3 | -2.5 | -8.8 | 4.2 |
| Japan | -4.4* | -5.8 | -3 | -4.1* | -5.6 | -2.5 |
| South Korea | -6.7* | -7.9 | -5.5 | -4.7* | -6.1 | -3.3 |
| Southeastern Asia |  |  |  |  |  |  |
| Philippines | -5.3* | -6.8 | -3.8 | -5.1* | -7.7 | -2.4 |
| Thailand | 3.4* | 1.3 | 5.6 | 2.7 | -1.8 | 7.4 |
| Eastern Europe |  |  |  |  |  |  |
| Belarus | -4.4* | -6.8 | -1.9 | -3.3* | -4.6 | -1.9 |
| Bulgaria | -4.6 | -9.7 | 0.8 | 3.1 | -1.4 | 7.7 |
| Czech Republic | -6.5* | -12.3 | -0.3 | -5.5 | -11.3 | 0.6 |
| Russian | -4.4* | -5.2 | -3.6 | -3.8* | -4.7 | -2.8 |
| Poland | -2.2 | -4.5 | 0.1 | -2.6 | -5.5 | 0.3 |
| Slovakia | 1.7 | -3 | 6.7 | 2.4 | -5.5 | 11 |
| Northern Europe |  |  |  |  |  |  |
| Denmark | -3.8 | -11.2 | 4.2 | -0.2 | -12 | 13.1 |
| Estonia | -2.8 | -10.7 | 5.9 | -10.8 | -20.5 | 0 |
| Finland | -2.6 | -12.9 | 9 | -4.7 | -11.6 | 2.7 |
| Iceland |  |  |  |  |  |  |
| Ireland | -2.6 | -8.7 | 4 | -2.4 | -10.1 | 6 |
| Lithuania | 0.5 | -6.9 | 8.5 | 2.9 | -7.2 | 14.1 |
| Sweden | -5 | -10.5 | 0.9 | 0.4 | -8.3 | 10 |
| Norway | 3.1 | -4.7 | 11.4 | -1.4 | -11.4 | 9.7 |
| UK | -1.2 | -4.5 | 2.1 | 1.5 | -2.3 | 5.5 |
| Western Asia |  |  |  |  |  |  |
| Cyprus |  |  |  |  |  |  |
| Israel | -2.2 | -8.1 | 4.1 | -3.2 | -9.2 | 3.2 |
| Turkey | -2.8 | -9.3 | 4.2 | -0.2 | -2.9 | 2.6 |
| Southern Europe |  |  |  |  |  |  |
| Croatia | -4.9* | -8.1 | -1.6 | -5.8 | -17.9 | 8.1 |
| Italy | -0.5 | -3.4 | 2.5 | -3.6* | -5.1 | -2 |
| Malta |  |  |  |  |  |  |
| Slovenia | -9.0* | -13.5 | -4.3 | -2.2 | -12.9 | 9.9 |
| Spain | -2.4 | -4.9 | 0.2 | -4.5* | -6.1 | -2.8 |
| Western Europe |  |  |  |  |  |  |
| Austria | -4.6 | -11.3 | 2.7 | -3.5 | -9.4 | 2.7 |
| France | 1.1 | -2.4 | 4.6 | 0.6 | -1.9 | 3.2 |
| Germany | -3.4* | -5.3 | -1.5 | -4.1* | -6.7 | -1.4 |
| The Netherlands | 1.9 | -1.4 | 5.3 | -2.5 | -8.5 | 4 |
| Switzerland | -5 | -12.8 | 3.4 | 0.9 | -9.1 | 12 |
| Oceania |  |  |  |  |  |  |
| Australia | 0 | -3.5 | 3.7 | 1.4 | -3.2 | 6.1 |
| New Zealand | 2.8 | -3.8 | 9.8 | -3.3 | -11.4 | 5.5 |
| *：P<0.05 |  |  |  |  |  |  |

| Supplement Table 5 AAPC of the mortality of Gastric Cancer in Individuals 45 to 69 Years | | | | | | |
| --- | --- | --- | --- | --- | --- | --- |
| Region | Males | |  | Females | |  |
|  | APCC | -95% CI | 95% CI | APCC | -95% CI | 95% CI |
| South America |  |  |  |  |  |  |
| Brazil | -2.4* | -2.8 | -2 | -1.4* | -2.1 | -0.6 |
| Chile | -3.7* | -4.7 | -2.8 | -1.5* | -2.8 | -0.1 |
| Colombia | -2.6* | -3.6 | -1.5 | -3.1* | -4.5 | -1.7 |
| Ecuador | -3.3* | -4.7 | -1.9 | -3.0* | -5.4 | -0.6 |
| Northern America |  |  |  |  |  |  |
| Canada | -3.6* | -4.3 | -2.9 | -1.3 | -3.1 | 0.7 |
| USA | -2.1* | -2.6 | -1.6 | -1.0* | -1.6 | -0.3 |
| Eastern Asia |  |  |  |  |  |  |
| China | -4.0* | -7.1 | -0.9 | -1.2 | -3.9 | 1.6 |
| Japan | -4.3* | -4.7 | -3.9 | -3.9* | -4.2 | -3.6 |
| South Korea | -7.8* | -8.3 | -7.4 | -7.6* | -8.3 | -7 |
| Southeastern Asia |  |  |  |  |  |  |
| Philippines | -5.4* | -6.2 | -4.6 | -4.9* | -6 | -3.8 |
| Thailand | 3.5* | 1.9 | 5.1 | 5.3* | 3.8 | 6.9 |
| Eastern Europe |  |  |  |  |  |  |
| Belarus | -3.8* | -4.5 | -3.1 | -4.2* | -4.7 | -3.8 |
| Bulgaria | -3.9* | -5.6 | -2.2 | -2.8* | -4.4 | -1.2 |
| Czech Republic | -4.4* | -5.8 | -3.1 | -2.3* | -4.2 | -0.4 |
| Russian | -4.3* | -4.9 | -3.6 | -4.6* | -5.3 | -3.8 |
| Poland | -3.1* | -3.6 | -2.7 | -2.2* | -3.4 | -1 |
| Slovakia | -3.5* | -4.6 | -2.5 | -2.3 | -5.3 | 0.7 |
| Northern Europe |  |  |  |  |  |  |
| Denmark | -2.9* | -5 | -0.7 | -1.9 | -5.6 | 2.1 |
| Estonia | -4.4* | -7 | -1.8 | -3.4* | -6.4 | -0.4 |
| Finland | -4.1* | -5.8 | -2.3 | -2.6 | -6.2 | 1.2 |
| Iceland | -0.7 | -6 | 5 | -4.9 | -14.9 | 6.3 |
| Ireland | -2.8 | -6.7 | 1.1 | -1.2 | -7 | 4.9 |
| Lithuania | -4.4* | -5.8 | -3 | -0.6 | -3.6 | 2.6 |
| Sweden | -4.3* | -5.9 | -2.7 | -1.7 | -3.7 | 0.4 |
| Norway | -2.6* | -4 | -1.2 | -5.0* | -8.9 | -0.8 |
| UK | -3.4* | -4.3 | -2.4 | -2.6* | -4.2 | -1 |
| Western Asia |  |  |  |  |  |  |
| Cyprus | -2 | -8.9 | 5.5 | -1.6 | -10.9 | 8.6 |
| Israel | -2.7* | -5 | -0.3 | -0.8 | -3.5 | 2 |
| Turkey | -1 | -5.9 | 4.2 | -4.6 | -9.7 | 0.7 |
| Southern Europe |  |  |  |  |  |  |
| Croatia | -4.0* | -6 | -2 | -0.6 | -1.8 | 0.5 |
| Italy | -3.7* | -4.5 | -2.9 | -2.6* | -3.6 | -1.6 |
| Malta | -0.1 | -9.3 | 10 | -5.1 | -14.5 | 5.4 |
| Slovenia | -6.1* | -9.7 | -2.4 | -0.4 | -5.3 | 4.7 |
| Spain | -2.6* | -3.9 | -1.4 | -0.1 | -1.1 | 0.9 |
| Western Europe |  |  |  |  |  |  |
| Austria | -1.4 | -3.4 | 0.7 | -2.6* | -5 | -0.1 |
| France | -2.2* | -3.1 | -1.3 | -1.7* | -2.7 | -0.6 |
| Germany | -2.7* | -3.2 | -2.2 | -2.8* | -3.5 | -2.1 |
| The Netherlands | -3.7* | -6 | -1.4 | -2.7* | -4.4 | -1.1 |
| Switzerland | -0.2 | -1.7 | 1.3 | -1.6 | -4.8 | 1.7 |
| Oceania |  |  |  |  |  |  |
| Australia | -1.5* | -2.8 | -0.2 | -3.5* | -5.2 | -1.7 |
| New Zealand | -2.0* | -3.4 | -0.6 | -2.7 | -5.4 | 0 |
| *：P<0.05 |  |  |  |  |  |  |

| Supplement Table 6 AAPC of the mortality of Gastric Cancer in Individuals 70 to 85 Years or Older | | | | | | |
| --- | --- | --- | --- | --- | --- | --- |
| Region | Males | |  | Females | |  |
|  | APCC | -95% CI | 95% CI | APCC | -95% CI | 95% CI |
| South America |  |  |  |  |  |  |
| Brazil | -2.5* | -3 | -2 | -3.2* | -4.2 | -2.2 |
| Chile | -1.9* | -3.3 | -0.4 | -2.3* | -3.4 | -1.2 |
| Colombia | -2.3* | -3.2 | -1.4 | -3.8* | -5.4 | -2.3 |
| Ecuador | -4.4* | -6.4 | -2.5 | -4.0* | -5.4 | -2.5 |
| Northern America |  |  |  |  |  |  |
| Canada | -3.4* | -4.3 | -2.5 | -2.7* | -4.3 | -1.1 |
| USA | -2.8* | -3.3 | -2.3 | -2.7* | -3 | -2.3 |
| Eastern Asia |  |  |  |  |  |  |
| China | -2.6* | -3.9 | -1.1 | -2.9* | -5.1 | -0.7 |
| Japan | -3.1* | -3.3 | -2.8 | -3.5* | -3.7 | -3.3 |
| South Korea | -6.1* | -6.5 | -5.7 | -5.7* | -6.3 | -5 |
| Southeastern Asia |  |  |  |  |  |  |
| Philippines | -4.7* | -5.5 | -3.9 | -4.9* | -6 | -3.7 |
| Thailand | 4.1* | 1 | 7.2 | 4.5* | 2.6 | 6.4 |
| Eastern Europe |  |  |  |  |  |  |
| Belarus | -3.3* | -4 | -2.6 | -4.0* | -5.2 | -2.7 |
| Bulgaria | -2.4* | -3.5 | -1.2 | -5.2* | -7 | -3.4 |
| Czech Republic | -4.3* | -6.6 | -2 | -4.0* | -5.4 | -2.5 |
| Russian | -2.4* | -3.2 | -1.7 | -2.6* | -3 | -2.2 |
| Poland | -2.1* | -2.5 | -1.8 | -2.8* | -3.8 | -1.8 |
| Slovakia | -2.8* | -4.5 | -1.1 | -3.0* | -5.3 | -0.7 |
| Northern Europe |  |  |  |  |  |  |
| Denmark | -1.2 | -3.1 | 0.6 | -2.1 | -5.5 | 1.5 |
| Estonia | -2.1 | -5.6 | 1.5 | -3.3* | -5.6 | -1 |
| Finland | -5.1* | -7.3 | -2.9 | -4.5* | -6.3 | -2.6 |
| Iceland | -4.6 | -11.6 | 3 | -1.2 | -11.5 | 10.2 |
| Ireland | -3.8* | -6.8 | -0.8 | -3.3* | -5.4 | -1.2 |
| Lithuania | -0.3 | -2.3 | 1.7 | -3.0* | -5.5 | -0.5 |
| Sweden | -4.4* | -5.6 | -3.1 | -3.5* | -5.6 | -1.3 |
| Norway | -2.9* | -5.2 | -0.5 | -5.1* | -6.6 | -3.6 |
| UK | -4.3* | -4.7 | -3.9 | -4.3* | -5.1 | -3.5 |
| Western Asia |  |  |  |  |  |  |
| Cyprus | 1 | -4.2 | 6.6 | 1.3 | -6.8 | 10 |
| Israel | -1.9* | -3.1 | -0.7 | -4.1* | -6.3 | -1.9 |
| Turkey | -0.6 | -6.8 | 5.9 | 1.5 | -2.9 | 6.1 |
| Southern Europe |  |  |  |  |  |  |
| Croatia | -2.8* | -4.3 | -1.3 | -2.7* | -4 | -1.3 |
| Italy | -3.1* | -3.6 | -2.7 | -3.4* | -3.9 | -2.8 |
| Malta | -4 | 9.9 | 0.9 | 2.5 | -3.3 | 8.7 |
| Slovenia | -1.9 | -4.9 | 1.2 | -3.2* | -5.4 | -0.9 |
| Spain | -2.9* | -3.4 | -2.3 | -2.8* | -3.4 | -2.1 |
| Western Europe |  |  |  |  |  |  |
| Austria | -4.7* | -5.6 | -3.7 | -5.3* | -6.2 | -4.3 |
| France | -3.0* | -3.6 | -2.4 | -3.1* | -3.9 | -2.2 |
| Germany | -3.8* | -4.3 | -3.3 | -4.1* | -4.7 | -3.5 |
| The Netherlands | -4.0* | -4.9 | -3.1 | -3.8* | -5.6 | -1.9 |
| Switzerland | -1.2 | -2.9 | 0.6 | -2.6* | -4.8 | -0.3 |
| Oceania |  |  |  |  |  |  |
| Australia | -3.1* | -4.7 | -1.4 | -2.4* | -4 | -0.7 |
| New Zealand | -3.9 | -8.9 | 1.5 | -1 | -4.4 | 2.5 |
| *：P<0.05 |  |  |  |  |  |  |
