# Supplementary material for: Global incidence and mortality trends of gastric cancer and predicted mortality of gastric cancer by 2035
Source: BMC Public Health. 2024 Jul 2;24:1763. doi: 10.1186/s12889-024-19104-6 (PMC11221210; doi:10.1186/s12889-024-19104-6)
Supplement: Supplementary file 10 — Supplementary material 10. [file 12889_2024_19104_MOESM10_ESM.docx]

**Supplement Results**

**Global incidence and mortality in 2020**

Among the population aged 20-44, Sao Tome and Principe, Mongolia, South Korea, Samoa, and France have the highest incidence rate (Figure 2A).

Among the population aged 45-69, Mongolia, Japan, South Korea, China, and Tajikistan have the highest incidence rate (Figure 2A).

Among the population aged 70-85+, Japan, Mongolia, Tajikistan, Iran, and South Korea have the highest incidence rate (Figure 2A).

Among the population aged 20-44, Sao Tome and Principe, Samoa, Mongolia, Tajikistan, and Lao People's Democratic Republic have the highest mortality rates (Figure 2B).

Among the population aged 45-69, Mongolia, Bhutan, Tajikistan, Kyrgyzstan, and Sao Tome and Principe have the highest mortality rates (Figure 2B).

Among the population aged 70-85+, Mongolia, Tajikistan, Iran, Cape Verde, and China have the highest mortality rates (Figure 2B).

**Average annual percent change in the incidence of gastric cancer from 2003 to 2012**

Among the male population aged 20-44, Brazil, Sweden, Switzerland, UK, Australia, New Zealand, Lithuania, Ecuador, USA, the Netherlands and Estonia had an increasing trend (P>0.05); 24 countries had a decreasing trend (P>0.05); Other 6 countries had a decreasing trend (P<0.05); Among women population aged 20-44, USA [AAPC, 2.0; (95%Cl, 0.8-3.2), P<0.05] and Ecuador [AAPC, 4.0; (95%Cl, 0.5-7.6), P<0.05] had an increasing trend; Norway, Poland, Thailand, the Netherlands, Canada, Lithuania, Turkey, Colombia and Slovakia had an increasing trend (P>0.05); 23 countries had a decreasing trend (P>0.05); 2 countries had a decreasing trend (P<0.05) (Supplement Table 1 and Supplement Figure 1).

Among the male population aged 45-69, Canada [AAPC, 0.9; (95%cl, 0-1.9), P<0.05] showed an increasing trend; Cyprus, Switzerland, Thailand, Malta, South Korea, and Germany had an increasing trend (P>0.05); 14 countries had a decreasing trend (P>0.05); 19 countries had a decreasing trend (P<0.05); among the female population aged 45-69, Canada [AAPC, 3.1; (95%Cl, 1.7-4.5), P<0.05] had an increasing trend; Slovakia, Cyprus, Switzerland, Thailand, Ecuador, Denmark, South Korea, and Slovenia showed an increasing trend (P>0.05); 17 countries had a decreasing trend (P>0.05); 15 countries had a decreasing trend (P<0.05) (Supplement Table 2 and Supplement Figure 2).

Among the male population aged 70-85+, Japanese males [AAPC, 1; (95%Cl, 0.2-1.9), P<0.05] had an increasing trend; Switzerland, India, Ireland, and South Korea had an increasing trend (P>0.05); 22 countries had a decreasing trend (P>0.05); 14 countries had a decreasing trend (P<0.05); among the female population aged 70-85+, Japan women [AAPC, 0.5; (95%Cl, 0-1), P<0.05] had an increasing trend; Slovakia, India, Thailand, and Chile had an increasing trend (P>0.05); 18 countries had a decreasing trend (P>0.05); 17 countries had a decreasing trend (P<0.05) (Supplement Table 3 and Supplement Figure 3).

**Average annual percent change in the mortality of gastric cancer from 2006 to 2015**

Among the male population aged 20-44, Thailand [AAPC, 3.4; (95%Cl, 1.3-5.4), P<0.05] showed an increasing trend; Norway, New Zealand, The Netherlands, Slovakia, France, Colombia, Lithuania, and the USA showed an increasing trend (P>0.05); 17 countries had a decreasing trend (P>0.05); 11 countries had a decreasing trend (P<0.05); among women population aged 20-44, Bulgaria, Lithuania, Thailand, Slovakia, UK, Australia, Chile, Switzerland, Colombia, France, Sweden, Ecuador, and Brazil had an increasing trend (P>0.05); 17 countries had an increasing trend (P>0.05); eight countries had an increasing trend (P<0.05) (Supplement Table 4 and Supplement Figure 4).

Among the male population aged 45-69, Thailand [AAPC, 3.5; (95%Cl, 1.9-5.1), P<0.05] had an increasing trend; 7 countries had an increasing trend (P>0.05); 33 countries had a decreasing trend (P<0.05); among the female population aged 45-69, Thailand [AAPC, 5.3; (95%Cl, 3.8-6.9), P<0.05] had an increasing trend; 17 countries had an increasing trend (P>0.05); 8 countries had a decreasing trend (P<0.05) (Supplement Table 5 and Supplement Figure 5).

Among the male population 70-85+, Thailand [AAPC, 4.1; (95%Cl, 1 -7.2), P<0.05] had an increasing trend; Cyprus had an increasing trend (P>0.05); 7 countries had a decreasing trend (P>0.05); 32 countries had a decreasing trend (P<0.05); among women population aged 70-85 +, Thailand [AAPC, 4.5; (95%cl, 2.6-6.4), P<0.05] had an increasing trend; Malta, Turkey, and Cyprus had an increasing trend (P>0.05); 3 countries had a decreasing trend (P>0.05); 34 countries had a decreasing trend (P<0.05) (Supplementary Table 6 and Supplement Figure 6).
